# A CHK1-mediated phosphorylation switch suppresses human Topoisomerase 1-associated genomic instability

**DOI:** 10.1038/s44318-026-00783-3

**Published:** 2026-05-13

**Authors:** Ananda Guha Majumdar, Nitish Chauhan, Pooja Gupta, Mahesh Subramanian, Birija Sankar Patro

**Affiliations:** 1 Bio-Organic Division, Bhabha Atomic Research Centre, Mumbai, India; 2 Homi Bhabha National Institute, Mumbai, India

**Keywords:** DNA Replication, Recombination & Repair

## Abstract

Topoisomerase 1 (TOP1) is essential for relieving DNA supercoils during replication and transcription. However, its transient reaction intermediates (TOP1 cleavage complexes or TOP1-DNA covalent complexes, *i.e.,* TOP1ccs) become highly genotoxic when stabilized. While mechanisms that resolve chemotherapy-induced TOP1ccs are well-characterized, how cells prevent their accumulation under physiological conditions for securing genomic stability has remained elusive. Here, we elucidate a novel regulatory pathway in which CHK1-mediated phosphorylation of TOP1 at Serine-320 regulates its religation activity and hence limits steady-state TOP1cc levels during unperturbed cellular metabolism. We further demonstrate a distinct mechanism of TOP1cc stabilization, which escapes recognition by proteasomal and autophagic machineries, while being susceptible to CtIP, SPRTN, and p97-mediated removal. Defective phosphorylation of TOP1 at S320 impairs replication-fork progression, leading to replication- and transcription-associated DSBs, R-loop stabilization, genomic instability, and hypersensitivity to TOP1 poisons. Overall, our study assigns a new function to CHK1 in direct regulation of human TOP1cc dynamics, with critical implications for genomic integrity and combinatorial chemotherapy.

## Synopsis

While resolution of chemotherapy-induced TOP1-DNA covalent complexes (TOP1cc’s) has been well-studied, their control under physiological conditions remains unclear. Here, CHK1 is found to phosphorylate TOP1, preventing persistent TOP1cc’s, replication-transcription conflicts and R-loop stabilization that all lead to genomic instability.

- CHK1 phosphorylates TOP1 at S320 and directly regulates the religation step of the TOP1 catalytic cycle.
- CHK1-mediated TOP1-S320 phosphorylation suppresses spontaneous accumulation of TOP1cc’s on whole genome.
- CHK1 inhibition generates persistent TOP1cc’s, which are targeted and removed by CtIP, SPRTN and p97.
- CHK1-mediated TOP1 phosphorylation suppresses R-loop accumulation, transcription-replication conflicts, double strand breaks and genome instability.

## Abstract (web summary)

TOP1 S320 phosphorylation prevents TOP1-DNA covalent-complex accumulation under normal physiological conditions.

## Graphical abstract

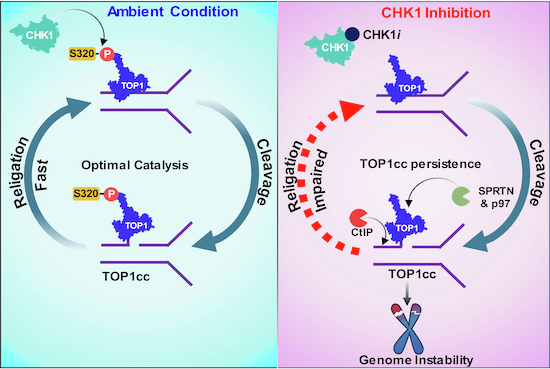

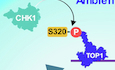

## Introduction

Topoisomerase 1 (TOP1) performs indispensable functions in replication and transcription through relaxation of DNA supercoils employing a processive cleavage-rotation-religation cycle (Pommier et al, 2022, 2016b) involving the formation of a transient Topoisomerase 1 cleavage complex (also referred to as TOP1-DNA covalent complex, or TOP1cc) intermediate (Pommier et al, 2016b). Under ambient conditions, the significantly faster catalytic kinetics of religation compared to cleavage ensure transience of TOP1ccs (Pommier et al, 2022). However, various conditions, including DNA damage of spontaneous origin or exposure to TOP1 poisons (*e.g.,* camptothecin, CPT), may stabilize these intermediates. Once stabilized, TOP1ccs function as sources of DSBs (due to conflicts with replication or transcription machinery) as well as RNA/DNA hybrids (R-loops) (Pommier et al, 2022, 2016b; Cristini et al, 2019). Consequently, cells deploy extensive countermeasures to rapidly resolve such structures (Cristini et al, 2019). Although multiple mechanisms involved in resolving TOP1ccs in response to TOP1 poisons have been elucidated, regulatory processes that prevent TOP1cc persistence under unperturbed physiological conditions [such as SPRTN-mediated TOP1cc removal orchestrated by TEX-264 and p97 (Fielden et al, 2020)] are only beginning to be unraveled.

Removal of genotoxic TOP1ccs may be orchestrated by proteasomal/non-proteasomal processing of TOP1ccs followed by their removal by TDP1/TDP2, or *via* nuclease-mediated removal of a TOP1cc-containing oligonucleotide segment involving a battery of context-specific nucleases (involving, but not limited to, the XPF-ERCC complex, MUS81/EME1, MRE11, CtIP, etc.) (Lin et al, 2009; Fielden et al, 2020; Yaneva et al, 2023; Gupta et al, 2022). More recently, TEX264-driven selective autophagy has been demonstrated to play a critical role in the repair of TOP1cc-associated DNA lesions (Lascaux et al, 2024). Further, we have recently unveiled a Werner Syndrome (WRN) helicase-mediated pathway that triggers TOP1cc removal *via* TDP1 and CtIP (Gupta et al, 2022). These responses are synchronized into a highly efficient and robust process, which is vital to the maintenance of genomic stability (Pommier et al, 2006; Crewe and Madabhushi, 2021).

Post-translational modifications (PTMs) have been shown to play a pivotal role in the removal of TOP1ccs. CPT exposure has been shown to trigger rapid DNA-PK-mediated phosphorylation of TOP1 at Serine 10, which is essential for its proteasomal degradation (Ando et al, 2017). Ubiquitination (Lin et al, 2008), SUMOylation (Sun et al, 2020a), and NEDDylation (Meroni et al, 2022) have been shown to regulate various aspects of proteasomal as well as non-proteasomal degradation of TOP1ccs (Fielden et al, 2020; Sun et al, 2020b). PARylation operates at multiple levels, ranging from regulation of TOP1cc degradation to recruitment of TDP1 and fork remodeling factors, as well as regulation of nucleolar localization of TOP (Das et al, 2016; Sun et al, 2021; Chowdhuri and Das, 2021; Berti et al, 2013). Phosphorylation of TOP1 at Tyrosine 268 (by c-ABL kinase), Serine 10 (by CK-2), Serine 21 (by protein kinase Cα), Serine 112, and Serine 394 (by CDK1) has been shown to regulate TOP1 catalytic activity (Yu et al, 2004; Hackbarth et al, 2008). Similarly, CK-2 regulates TOP1 catalytic activity, CPT sensitivity, and interaction with p14/ARF through phosphorylation of TOP1 at Tyrosine 506 (Bandyopadhyay et al, 2012; Bandyopadhyay and Gjerset, 2011). Although phosphorylation has been established as a key regulator of TOP1 dynamics, its role in the regulation of steady-state TOP1cc abundance in cells under unperturbed conditions remains poorly understood.

CHK1, a key cell cycle checkpoint kinase and DNA damage response effector, plays a crucial role in the downstream cellular events following TOP1 inhibition (Gupta et al, 2022; Patro et al, 2011). We have previously demonstrated that CHK1 is rapidly activated in response to TOP1ccs in a manner dependent on WRN helicase (Gupta et al, 2022; Patro et al, 2011), thereby rendering indispensable functions in the enforcement of cell cycle checkpoints (Neizer-Ashun and Bhattacharya, 2021; Flatten et al, 2005). Canonical activation of CHK1 occurs through ATR-mediated phosphorylation upon induction of DNA damage or replication stress (Neizer-Ashun and Bhattacharya, 2021). Replication stress (of which sources might range from endogenous DNA damage to genomic regions posing conditions inclement to replication) results in uncoupling of the DNA polymerase from the replicative helicase, resulting in the accumulation of long stretches of single-stranded DNA. This in turn activates the ATR and pathway, finally culminating in CHK1 activation (Zeman and Cimprich, 2014; Iyer and Rhind, 2017). Consequently, drug-induced TOP1cc stabilization has been shown to trigger CHK1 activation (owing to TOP1cc-induced replisome stalling or collapse), resulting in intra-S-phase arrest (Jossé et al, 2014; Wang et al, 2002). Interestingly, it has been demonstrated that cells maintain a basal CHK1 activity in the absence of replication stress. This ATR-dependent mechanism (Saldivar et al, 2018), which relies on CHK1-autoactivation (Michelena et al, 2019), performs a variety of critical functions ranging from maintenance of CHK1 stability (thus preserving S-phase checkpoint integrity) (Michelena et al, 2019), to maintenance of optimal replication origin firing (Moiseeva et al, 2019). The mechanism of ATR/CHK1 activation under unperturbed replication remains an actively investigated question. However, it is proposed that basal ATR/CHK1 activation may be initiated by short stretches of single-stranded DNA generated by the action of the CMG helicase or at unligated Okazaki fragments (Moiseeva et al, 2019). While multiple roles played by basal cellular CHK1 activity pertaining to checkpoint enforcement and replication fork integrity have been well documented, there has been no direct demonstration of its role in the regulation of TOP1 catalysis.

In this study, we identified CHK1 as a novel regulator of cellular TOP1cc levels by performing a focused screen using a library of kinase inhibitors. Our findings demonstrate that pharmacological inhibition of CHK1 results in extensive stabilization of TOP1ccs on the genome. A combination of biochemistry, cell biology, mass spectrometry, and computational analysis reveals that CHK1 phosphorylates TOP1 and Serine 320, thereby regulating the religation step of the TOP1 catalytic cycle. Loss of this phosphorylation leads to TOP1cc persistence on the genome, resulting in replication and transcription-associated DNA damage, chromosomal aberrations, transcription–replication collisions, and accumulation of R-loops. Collectively, our findings suggest a previously unrecognized role of basal cellular CHK1 kinase activity in suppression of genomic instability through regulation of the TOP1 catalytic cycle under physiological conditions.

## Results

### A focused kinase screen reveals CHK1 as a regulator of TOP1 dynamics

**Figure 1:**
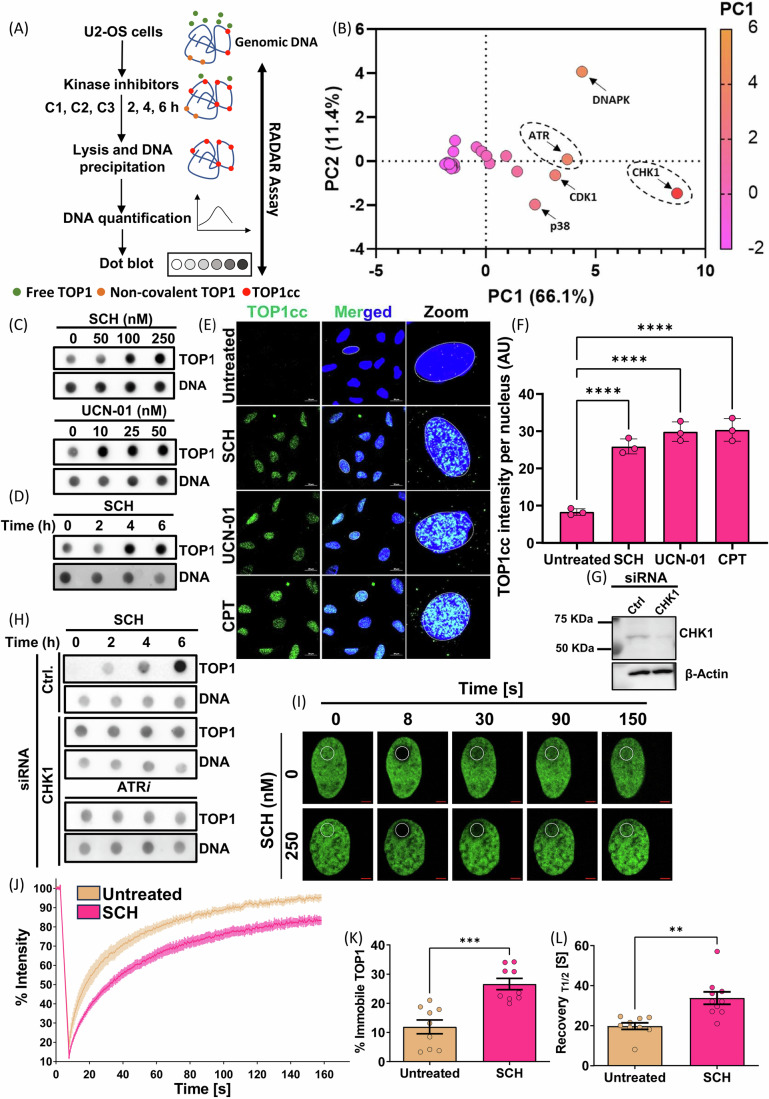
Pharmacological inhibition of CHK1 stabilizes TOP1ccs. (**A**) Schematic representation of the RADAR assay screen. (**B**) Principal component analysis (PCA) of RADAR screen data. (**C**) Representative RADAR assay showing TOP1cc stabilization in U2-OS cells treated with CHK1 inhibitors, SCH900776 (SCH; 50, 100, and 250 nM) and UCN-01 (10, 25, and 50 nM) for 6 h. (**D**) Representative time-course RADAR assay of TOP1cc stabilization in U2-OS cells subjected to SCH treatment (250 nM) for 2, 4, or 6 h. (**E**, **F**) Representative TOP1cc immunofluorescence microscopy (and quantification across three replicates) in U2-OS cells treated with SCH (250 nM), UCN-01 (50 nM) for 2 h, or CPT (100 nM) for 1 h. Detection was performed with anti-TOP1cc monoclonal antibody. Zoomed nuclei are marked with white dotted lines. Scale bars: 10 µm. *P* values are Untreated *vs.* SCH: 0.000158, Untreated *vs.* UCN-01: 0.000173, Untreated *vs.* CPT: 0.000267 (data from three independent experiments with 150 cells per sample). (**G**) Representative immunoblot of CHK1 from U2-OS cells transfected with control siRNA or siRNA targeting CHK1. (**H**) Representative RADAR assay showing TOP1cc stabilization in U2-OS cells treated either with control siRNA or CHK1 siRNA for 24 h, followed by treatment with SCH (250 nM) or ATR inhibitor AZD6738 (ATR*i*). ATR*i* treatment (10 µM) was performed 2 h prior to SCH treatment. (**I**) Representative microscopic images of FRAP analysis of EGFP-TOP1 dynamics in U2-OS cells treated with SCH (250 nM) for 2 h. Scale bars: 1 µm. (**K**, **L**) Quantification of fluorescence recovery kinetics (percentage population of immobile EGFP-TOP1 and t_1/2_ (of fluorescence recovery) of the experiment shown in (**J**). *P* values are 0.000213 (**K**) and 0.001427 (**L**) (representative data of the replicate shown). See Appendix (Appendix Fig. S3A–C,K,L) for densitometric quantification and data from all replicates. Error bars represent SEM. (Kruskal–Wallis test with Dunn’s post hoc analysis). Source data are available online for this figure.

The regulation of TOP1 dynamics in living cells is nascently understood. To uncover novel physiological regulators of TOP1 dynamics, we screened a curated panel of 25 small-molecule kinase inhibitors (Appendix Table S1) using RADAR (Rapid Approach to DNA Adduct Recovery) (Kiianitsa and Maizels, 2013) assay (Fig. 1A). Inhibitors were selected for maximal isoform coverage with minimal reported off-target activity. The screen was estimated to interrogate a total of ~42 kinases linked to various aspects of the DNA damage response. Given the dose- and time-dependent nature of TOP1cc formation with agents such as CPT (Gupta et al, 2022; Sun et al, 2020a), we systematically profiled each inhibitor at three concentrations (C1 < C2 < C3) and three time points (2, 4, and 6 h). The screen successfully retrieved known TOP1 regulators, including CDK1 and DNA-PK (Hackbarth et al, 2008; Ando et al, 2017), validating the approach. Novel hits, including CHK1, ATR, and p38, were also obtained (Fig. 1B; Appendix Figs. S1 and S2). The capture of both CHK1 as well as it’s upstream kinase, *i.e.,* ATR in the screen was intriguing, signaling at a previously unrecognized role of the ATR-CHK1 axis in TOP1 homeostasis. CHK1, being the highest ranked novel hit (Fig. 1B) was pursued for mechanistic characterization.

Our group has previously demonstrated that CHK1 activates the pro-survival NF-κB pathway in response to nanomolar concentrations of CPT (Gupta et al, 2022). This, in conjunction with multiple other reports have established a downstream effector function of CHK1 kinase in cellular response to TOP1 poisoning. However, prevalent reports (including ours) have exclusively elucidated effector functions of CHK1 post stress-triggered activation. Our kinase inhibitor screen, however, pointed to an unreported phenomenon: basal CHK1 activity appeared to influence TOP1cc levels even in the absence of exogenous stress. To test this, we inhibited CHK1 in U2-OS cells using two structurally different ATP-competitive small molecules: SCH900776 (SCH; employed in the initial screen) and UCN-01, and quantified TOP1ccs by RADAR assay. Both inhibitors produced a striking accumulation of TOP1ccs (Fig. 1C,D; Appendix Fig. S3A–C), without affecting overall cellular TOP1 levels (Appendix Fig. S3D). Immunofluorescence microscopy with a monoclonal TOP1cc-specific antibody (Patel et al, 2016) independently confirmed SCH and UCN-01-induced TOP1cc stabilization (Fig. 1E,F).

Given its superior selectivity for CHK1 (Montano et al, 2012), SCH was chosen for subsequent investigation. This effect was reproducible across four different cancer cell lines of diverse origin, [pancreatic (PANC-1), lung (A549), colorectal (HCT116), and breast (MDA-MB-231)] (Appendix Fig. S3E,F), as well as non-transformed human WI-38 cells (Appendix Fig. S3G,H), demonstrating that CHK1-dependent control of TOP1ccs is not cell type-restricted. We also corroborated these findings employing siRNA-mediated depletion of CHK1. siCHK1 triggered robust TOP1cc stabilization, which was not significantly enhanced further upon CHK1*i* treatment (Fig. 1G,H; Appendix Fig. S3I). We also treated CHK1-depleted cells with an ATR-specific inhibitor (AZD6738; ATR*i*) to investigate the possible involvement of ATR kinase in the regulation of TOP1cc levels. ATR*i* failed to enhance TOP1cc stabilization in siCHK1-treated cells (Fig. 1H; Appendix Fig. S3I), strongly suggesting that the role of ATR is limited to maintenance of basal CHK1 activity, which then regulates TOP1cc levels. Further, induction of DNA damage (triggering secondary TOP1cc stabilization) and activation of the positive-feedback loop of CHK1 (where CHK1 inhibition leads to ssDNA accumulation, which elicits further CHK1 activation) upon exposure to a low concentration of CHK1 inhibitor were explored. In this regard, our results demonstrated that DNA damage markers, *e.g.,* phospho-KAP-1 (S824) and γ-H2AX levels, remained unaltered under our experimental conditions in response to CHK1 inhibitors (Appendix Figs. S3J and S4A–D). Similarly, although CPT treatment rapidly induced the formation of RPA and RPA32 foci (markers of ssDNA), treatment with CHKI inhibitors failed to elicit RPA/RPA32 foci (Appendix Fig. S5A–D), which ruled out any possibility of activation of the ssDNA-mediated positive feedback loop of CHK1 activation in response to our experimental conditions.

We next examined TOP1 dynamics in live cells using the FRAP (fluorescence recovery after photobleaching) assay (Das et al, 2016). Using FRAP assay in U2-OS cells stably expressing EGFP-TOP1 (U2-OS^EGFP-TOP1WT^), we found that untreated nuclei contained ~12–15% immobile TOP1, with the remainder highly mobile (Fig. 1I–K; Appendix Fig. S3K,L). SCH treatment (250 nM, 4 h) increased the immobile fraction to ~25–28% (Fig. 1K; Appendix Fig. S3K) and prolonged the t_1/2_ of fluorescence recovery from ~20 to ~35 s (Fig. 1L; Appendix Fig. S3L). Taken together, our results revealed that CHK1 inhibition induces a pronounced shift in TOP1 dynamics, revealing, for the first time, that basal CHK1 activity actively maintains TOP1 mobility under physiological conditions. Having established this unexpected role, we next sought to define its mechanistic basis.

### CHK1 modulates endogenous TOP1cc levels independently of replication and transcription

**Figure 2:**
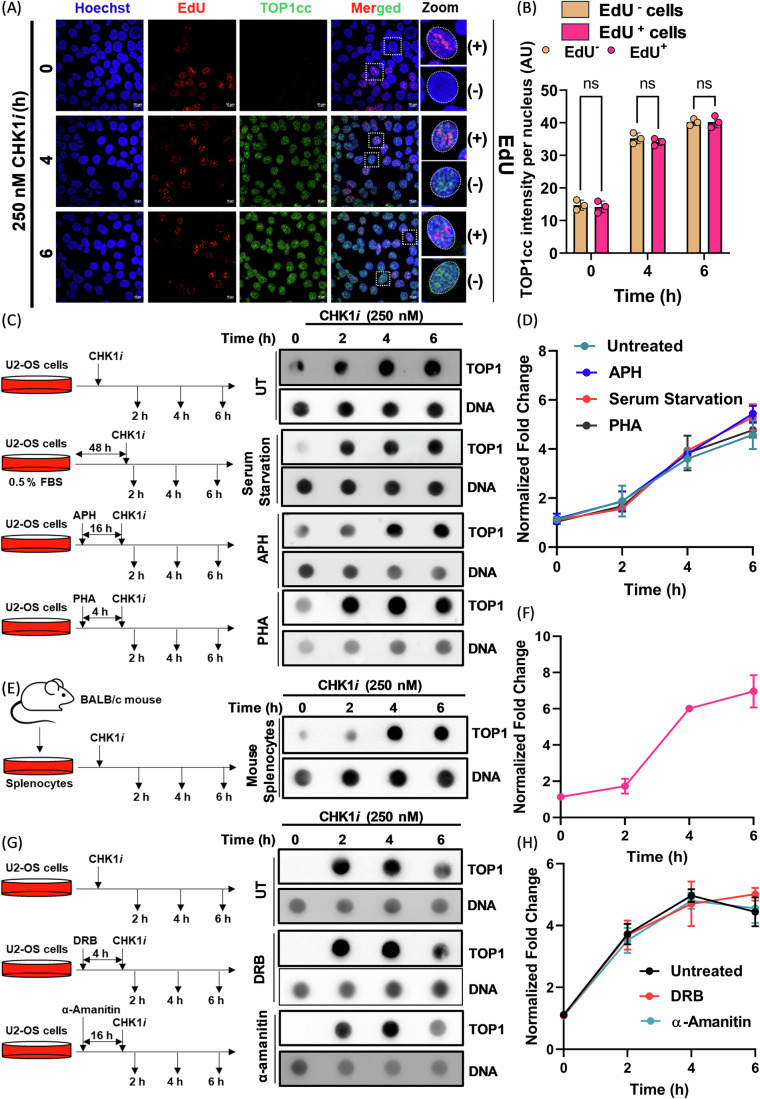
CHK1 inhibition stabilizes TOP1ccs independently of replication and transcription. (**A**, **B**) Representative microscopic images (and quantification) of dual EdU/TOP1cc staining in U2-OS cells treated with CHK1*i* (250 nM; 4 or 6 h). Cells were pulse-labeled with EdU (20 min) prior to fixation. TOP1ccs were detected through immunofluorescence employing an anti-TOP1cc monoclonal antibody; EdU incorporation was detected through click chemistry. Cells in white dotted boxes are shown in the zoomed panel (data from three independent replicates with 200 cells per condition). *P* values are (EdU^+^ *vs.* EdU^-^) 0 h: 0.717796, 4 h: 0.386922, 6 h: 0.954769 (Kruskal–Wallis test with Dunn’s post hoc analysis). Scale bars: 10 µm. (**C**, **D**) Representative RADAR assay in U2-OS cells treated with CHK1*i* (2, 4, or 6 h) alone (UT) or following replication inhibition by serum starvation (0.5% fetal bovine serum, *i.e.,* FBS for 48 h), Aphidicolin (APH; 250 nM, 16 h), or CDC7 inhibitor PHA-767491 (PHA; 5 µM, 4 h). (**E**, **F**) Representative RADAR assay in freshly isolated mouse splenocytes treated with CHK1*i*. (**G**, **H**) Representative RADAR assay showing TOP1cc stabilization by CHK1*i* in U2-OS cells alone (UT) or following prior transcription inhibition with DRB (100 µM, 4 h) or α-Amanitin (40 µg/mL, 16 h). Densitometric data in (**D**, **E**, **H**) shown from three independent experiments. Source data are available online for this figure.

**Figure EV1:**
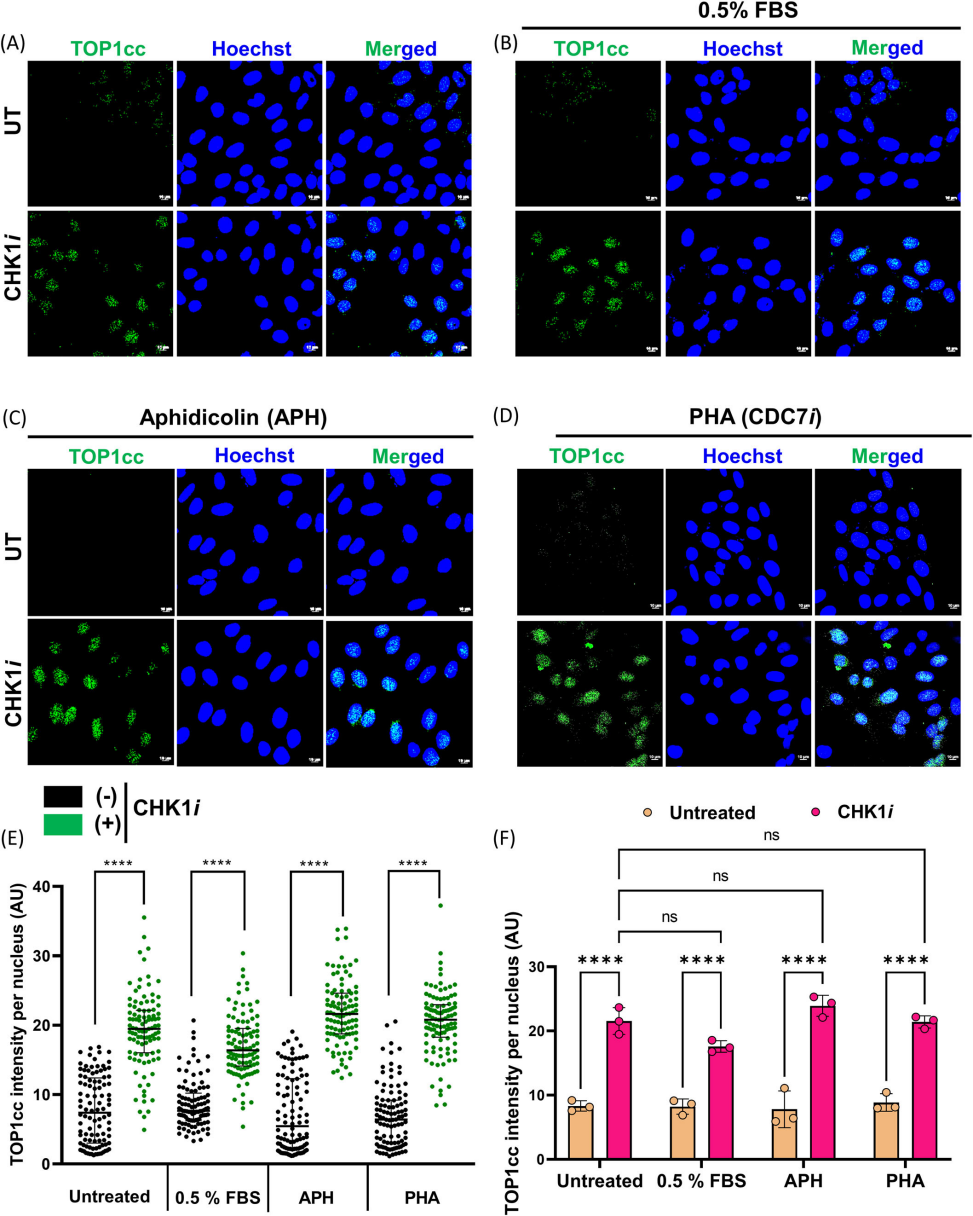
Replication-independent stabilization of TOP1ccs in response to CHK1*i.* (**A**–**D**) Representative immunofluorescence microscopy images of TOP1cc levels in U2-OS cells subjected to CHK1*i*-treatment in the absence (**A**) or presence of replication inhibition. U2-OS cells were subjected to serum starvation (0.5% fetal bovine serum/FBS, 48 h) (**B**), Aphidicolin (APH, 200 nM, 16 h) (**C**), or CDC7i (PHA, 5 µM, 4 h) (**D**), followed by 250 nM CHK1*i* treatment for 2 h. Cellular TOP1cc levels were detected using anti-TOP1cc monoclonal antibody. Scale bars: 10 µm for (**A**–**D**). (**E**) Quantitative representation of the replicates shown in (**A**–**D**). ****P < 0.0001. Error bars represent median ± interquartile range. Significance was determined using the Kruskal–Wallis test with Dunn’s post hoc analysis. (**F**) Quantification across three replicates of (**A**–**D**). *P* values are: <0.0001 (Untreated: No CHK1*i* *vs.* CHK1*i*), <0.0001 (0.5% FBS: No CHK1*i* *vs.* CHK1*i*), <0.0001 (APH: No CHK1*i* *vs.* CHK1*i*), <0.0001 (PHA: No CHK1*i* *vs.* CHK1*i*), 0.176245 (Untreated+CHK1*i* *vs.* 0.5% FBS+ CHK1*i*), 0.265347 (Untreated+CHK1*i* *vs.* APH+ CHK1*i*), 0.314577 (Untreated+CHK1*i* *vs.* PHA+CHK1*i*). 200 cells were scored per condition. Error bars represent SEM. Significance was determined using Kruskal–Wallis test with Dunn’s post hoc analysis. Source data are available online for this figure.

TOP1 is essential in the context of replication as well as transcription. Since CHK1 plays critical regulatory roles in both processes (Zhang and Hunter, 2014), we first considered whether SCH (referred to CHK1*i*)-mediated TOP1cc stabilization might simply reflect disruption of these processes. To address replication, we employed EdU (5-Ethynyl-2’-deoxyuridine) incorporation assay to differentially detect TOP1cc stabilization in replicating *versus* non-replicating cells upon exposure to CHK1*i*. TOP1cc stabilization occurred irrespective of EdU incorporation status of cells (Fig. 2A,B). We next suppressed replication using aphidicolin (DNA polymerase α inhibitor), PHA-767491 (CDC7 inhibitor; blocks origin firing), or serum starvation before CHK1*i* treatment. Each condition markedly reduced EdU incorporation (Appendix Fig. S6A–C) without altering CHK1*i*-induced TOP1cc stabilization (Fig. 2C,D). These observations were also confirmed using immunofluorescence microscopy employing TOP1cc-specific monoclonal antibody (Fig. EV1A–F). Consistently, CHK1*i* treatment led to stabilization of TOP1ccs in isolated mouse splenocytes (which show negligible replication under unstimulated conditions), further excluding replication perturbation as the primary driver (Fig. 2E,F).

To evaluate the contribution of transcription, we pre-treated cells with either 5,6-dichloro-1-β-D-ribofuranosylbenzimidazole (DRB) or α-amanitin to globally inhibit RNA synthesis prior to CHK1*i* exposure. Both inhibitors substantially reduced 5-ethynyl uridine (EU) incorporation (Appendix Fig. S6D,E) without affecting CHK1*i*-mediated TOP1cc stabilization (Fig. 2G,H). Together, these results indicate that CHK1*i*-induced TOP1cc stabilization arises from modulation of constitutive TOP1 engagement with chromatin, rather than from replication or transcriptional perturbations.

### CHK1*i*-induced TOP1cc persistence is mechanistically distinct from canonical TOP1 poisoning

Cellular TOP1cc levels reflect a dynamic balance between their formation and removal. Disruption of either (a) the TOP1 catalytic cycle or (b) TOP1cc clearance can elevate steady-state TOP1ccs. We therefore examined whether CHK1 influences TOP1cc homeostasis *via* altered degradation or clearance, using an alkaline lysis assay for TOP1 downregulation (Appendix Fig. S6F) (Lin et al, 2008; Desai et al, 1997).

**Figure 3:**
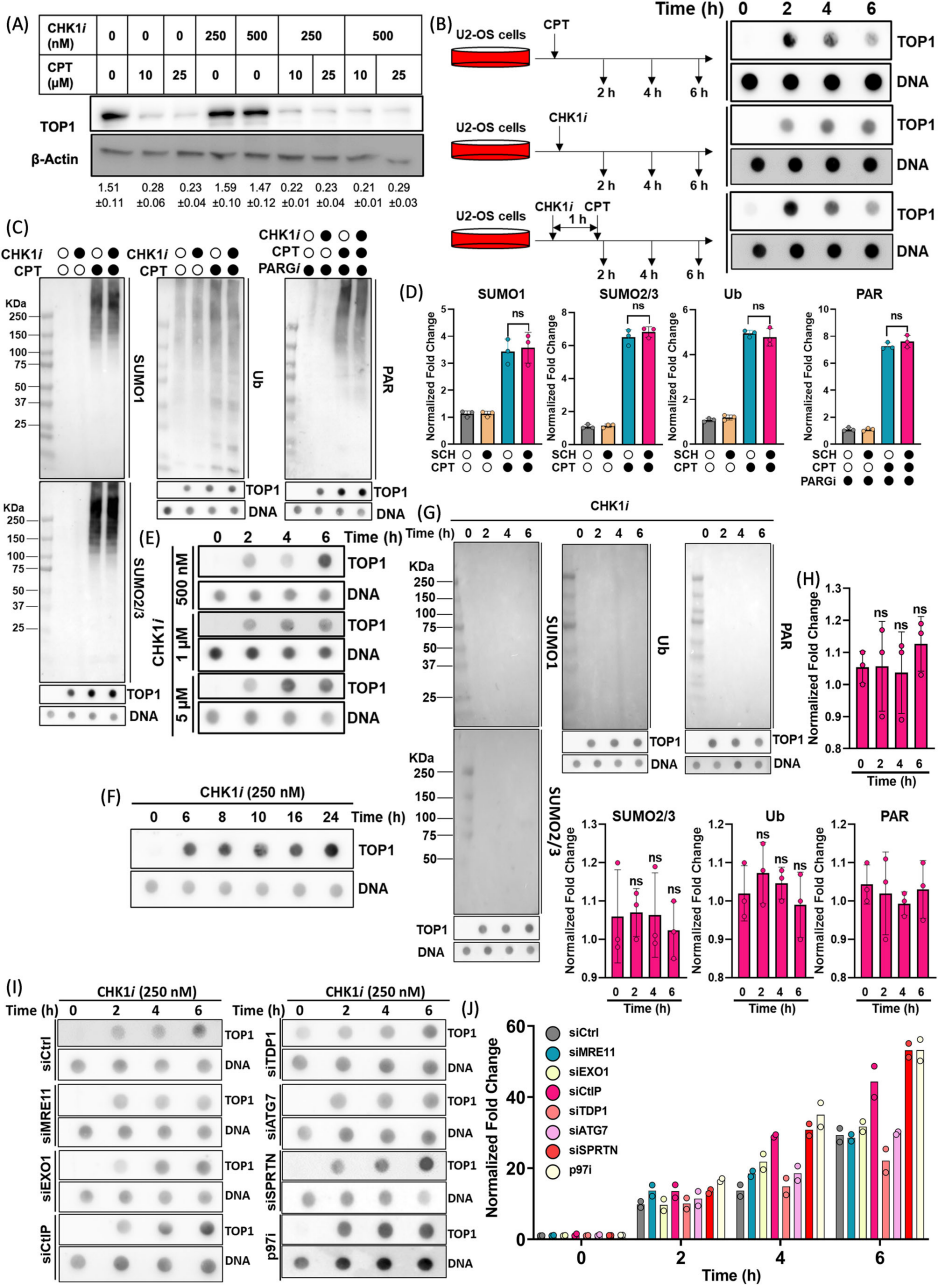
CHK1*i* stabilizes TOP1ccs through a distinct mechanism. (**A**) Global TOP1 levels in U2-OS cells treated with CPT (10 or 25 µM; 1 h) with or without CHK1*i* pre-treatment (250 or 500 nM; 1 h), assessed through TOP1 downregulation assay (alkaline lysis method). (**B**) RADAR assay of U2-OS cells treated with CPT (1 µM; 1 h) with or without CHK1*i* pre-treatment (250 nM; 1 h). See Appendix Fig. S6G for densitometric quantification. (**C**, **D**) Representative DUST assay of U2-OS cells treated with CPT (10 µM) for 30 min with or without pre-treatment with CHK1*i* (250 nM; 1 h). To detect TOP1cc PARylation, cells were pre-treated with PARG inhibitor PDD00017273 (10 µM, 1 h) prior to CHK1*i* treatment. DNA loading controls and TOP1cc levels are shown beneath each blot. SUMO1 and SUMO2/3 blots were developed from the same membrane through stripping and re-probing. ns: non-significant (data from three independent experiments; significance was determined using one-way ANOVA). (**E**) Representative RADAR assay showing TOP1cc accumulation in U2-OS cells treated with CHK1*i* (500 nM, 1 µM or 5 µM) for 2, 4, or 6 h. (**F**) Representative RADAR assay showing TOP1cc kinetics in U2-OS cells treated with CHK1*i* (250 nM) for 6, 8, 10, 16, or 24 h. See Appendix Fig. S6H,I for densitometric quantification. (**G**, **H**) Representative DUST assay of U2-OS cells treated with CHK1*i* (250 nM) for 2, 4, or 6 h. Detection of PARylation and SUMOylation was performed as described for (**C**). ns: non-significant (data from three independent experiments; significance was determined using one-way ANOVA). (**I**, **J**) Representative RADAR assay showing TOP1cc kinetics in U2-OS cells transfected with siRNAs or subjected to pharmacological inhibition of p97 prior to CHK1*i* treatment. siRNA transfections (control siRNA or siRNAs against MRE11, EXO1, CtIP, TDP1, ATG7 or SPRTN) were conducted 48 h prior to CHK1*i* treatment. p97i (CB-5083; 5 µM) was employed 2 h prior to CHK1*i* treatment (data from two independent replicates). Source data are available online for this figure.

CPT-mediated TOP1 poisoning is known to trigger global TOP1 downregulation. If CHK1*i* interfered with TOP1cc/TOP1 degradation pathways, we reasoned that it would alter the cellular response to CPT. However, pre-treatment with CHK1*i* (1 h) did not prevent CPT-induced TOP1 downregulation (Fig. 3A) or clearance of TOP1ccs (Fig. 3B; Appendix Fig. S6G), indicating that CHK1*i* does not impair CPT-driven TOP1 proteolysis or TOP1cc removal. Next, we employed DUST assay (Sun et al, 2020a) to probe post-translational modifications on TOP1ccs in CPT-treated cells in the absence or presence of CHK1*i*. DUST assays confirmed that canonical CPT-triggered PTMs on TOP1ccs, *i.e.,* SUMOylation, ubiquitination, and PARylation (Sun et al, 2020a) remained intact in the presence of CHK1*i* (Fig. 3C,D). Taken together, these observations pointed against a direct role played by CHK1 in TOP1cc degradation.

These observations also raised an intriguing question: CHK1*i* robustly stabilizes TOP1ccs, yet fails to trigger degradative PTMs, TOP1cc clearance, or TOP1 downregulation (Fig. 3A–D). Given that mammalian cells can engage multiple, context-specific TOP1cc removal pathways, including differential autophagic responses under nanomolar CPT stress (Lascaux et al, 2024), we explored whether altering CHK1*i* treatment conditions could induce clearance. Neither high-dose CHK1*i* treatment (up to 5 μM; Fig. 3E; Appendix Fig. S6H) nor extended exposure to 250 nM CHK1*i* for 24 h (Fig. 3F; Appendix Fig. S6I) promoted detectable TOP1cc removal, suggesting a general inability to efficiently process CHK1*i*-stabilized TOP1ccs.

We further aimed to conclusively rule out PTM induction on CHK1*i*-stabilized TOP1ccs. To this end, we performed DUST assays in U2-OS cells exposed to CHK1*i* for 2, 4, or 6 h. Our results suggested no appreciable SUMOylation, ubiquitination, or PARylation of CHK1*i*-stabilized TOP1ccs (Fig. 3G,H). We then tested whether nuclease-, non-proteasomal- or autophagy-mediated pathways contribute to their removal. We conducted siRNA-mediated depletion of key proteins involved in these pathways in U2-OS cells and measured TOP1cc kinetics upon CHK1*i* exposure. siRNA-mediated depletion of MRE11 or EXO1 did not alter TOP1cc kinetics in response to CHK1*i* (Fig. 3I,J; Appendix Fig. S6J). However, CtIP depletion resulted in a moderate but significant elevation in TOP1cc levels upon CHK1*i* treatment (Fig. 3I,J), indicating that CHK1*i*-stabilized TOP1ccs may be partially targeted by CtIP. Interestingly, SPRTN depletion or pharmacological inhibition of p97 (p97i) resulted in significant enhancement of CHK1*i*-induced TOP1cc accumulation, indicating that these TOP1ccs may be targeted for removal by SPRTN/p97 (Fig. 3I,J). This observation is interesting in the light of the recently demonstrated capability of TEX264 to recruit SPRTN/p97 at both unmodified and SUMOylated TOP1ccs (Fielden et al, 2020), thus explaining the SPRTN/p97-mediated targeting of CHK1*i*-stabilized TOP1ccs in the absence of degradative PTMs. Finally, depletion of TDP1 or ATG7 had no detectable influence on TOP1cc accumulation in the presence of CHK1*i* (Fig. 3I,J; Appendix Fig. S6J), effectively ruling out targeting of these TOP1ccs by the TDP1 or the autophagic pathways.

Together, these results reveal that TOP1ccs stabilized by CHK1*i* are mechanistically distinct from CPT-induced complexes: persisting without degradative PTMs and largely evading proteasomal and autophagic processing, while only being partially amenable to non-proteasomal (SPRTN/p97) and CtIP-mediated degradation, pointing to a previously unrecognized pathway of CHK1-dependent TOP1cc regulation.

### TOP1 is a bona fide CHK1 substrate

**Figure 4:**
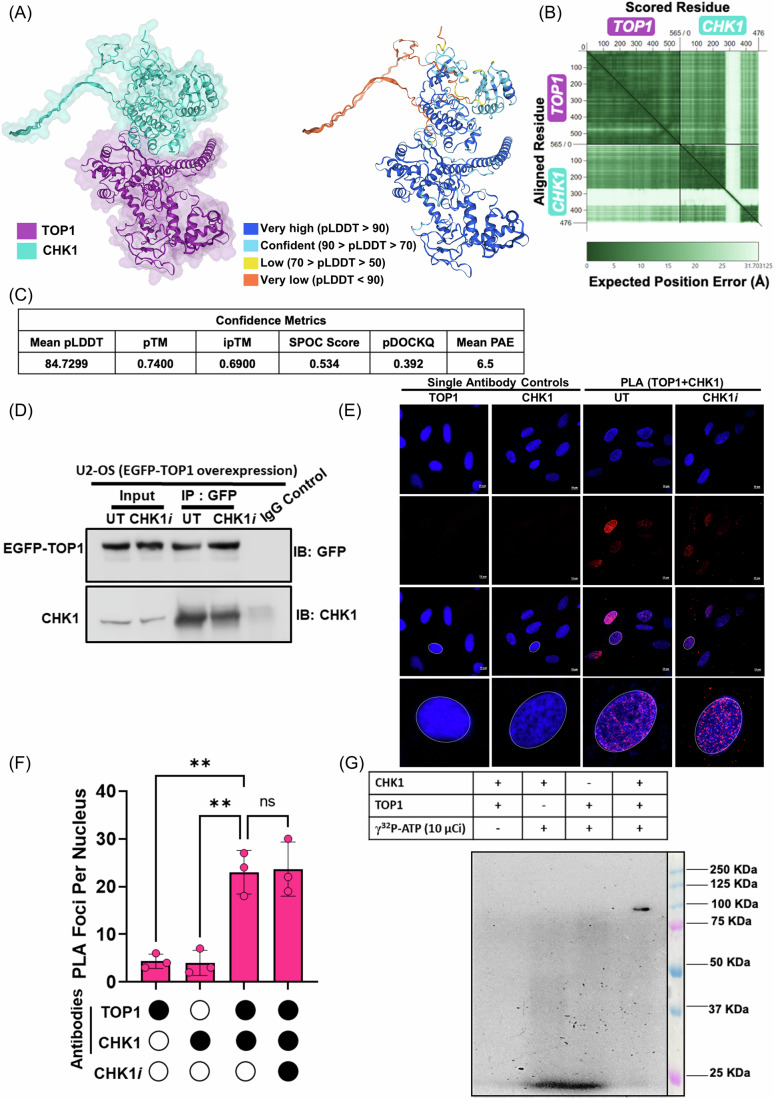
Characterization of CHK1–TOP1 interaction. (**A**) AlphaFold multimer prediction of CHK1–TOP1 interaction. The top-ranked model has been reproduced (subunit view and pLDDT views). (**B**) PAE plot of the top-ranked model. (**C**) Confidence metrics of the predicted interaction. pLDDT, pTM, and ipTM were calculated from the top-ranked model, while SPOC, pDOCKQ, and mean PAE were averaged across the three top-ranked models. Cutoffs: pLDDT<75, pTM>0.7, ipTM>0.55, SPOC > 0.3, pDOCKQ>0.23, PAE < 15. (**D**) Representative immunoblot showing co-immunoprecipitation of EGFP-TOP1 with endogenous CHK1 in U2-OS cells. U2-OS cells were transiently transfected with EGFP-TOP1 followed by immunoprecipitation from untreated, or CHK1*i*-treated (250 nM, 2 h) cells with anti-GFP antibody. (**E**, **F**) Representative microscopic images (and quantification across three replicates) of Proximity Ligation Assay (PLA) demonstrating endogenous CHK1–TOP1 interaction in U2-OS cells with or without CHK1*i* treatment (250 nM, 2 h). Zoomed nuclei are marked with white dotted lines. *P* values are: TOP1 antibody *vs.* TOP1 + CHK1 antibody: 0.012634, CHK1 antibody *vs.* TOP1 + CHK1 antibody: 0.006911, TOP1 + CHK1 antibody *vs.* TOP1 + CHK1 antibody + CHK1*i* 0.882028 (data from three independent experiments with 100 cells per condition; statistical significance was determined using Kruskal–Wallis test with Dunn’s post hoc analysis). Scale bars: 10 µm. (**G**) Representative autoradiograph of in vitro kinase assay with recombinant TOP1 and CHK1. Pre-stained molecular weight markers (run in parallel) were superimposed on the autoradiograph for reference. pLDDT Predicted Local Distance Difference Test, pTM Predicted Template Modeling score, ipTM Interface Template Modeling score, SPOC Structure Prediction and Omics Classifier, PAE Predicted Aligned Error. Error bars represent SEM. Source data are available online for this figure.

To explore how CHK1 regulates TOP1cc levels, we first examined whether CHK1 physically interacts with TOP1. Using AlphaFold Multimer (AFM) for theoretical modeling, we probed the interaction between TOP1 (excluding the disordered N-terminus) and CHK1. AFM predicted a moderately confident TOP1–CHK1 interaction, with two of five models meeting quality benchmarks (Fig. 4A–C; Appendix Fig. S7A–F). Domain-specific modeling revealed that the CHK1 kinase domain (residues 1–265), but not the KA1 domain (residues 391–476), was predicted to bind TOP1 (Appendix Figs. S7G–L and S8A–C).

We next validated this interaction in cellulo. We transiently overexpressed EGFP-TOP1 in U2-OS cells and performed immunoprecipitation with anti-GFP antibody. CHK1 was prominently co-immunoprecipitated with EGFP-TOP1 (Fig. 4D). This was further confirmed using proximity ligation assay (PLA) (Fig. 4E,F), which revealed nuclear interaction between TOP1 and CHK1. In co-immunoprecipitation as well as PLA, TOP1-CHK1 interaction was unabated in the presence of CHK1*i*, which is in line with previously reported non-interference of ATP-competitive CHK1 inhibitors with CHK1-substrate interactions (Sampath et al, 2002). Finally, we asked whether CHK1 can directly phosphorylate TOP1. Recombinant full-length human TOP1 was incubated with catalytically active recombinant CHK1 and γ^32^P-ATP. TOP1 was robustly phosphorylated by CHK1 in vitro, confirming TOP1 as a direct substrate for CHK1 (Fig. 4G; Appendix Fig. S8D).

### CHK1 phosphorylates TOP1 at a conserved serine residue critical for TOP1 dynamics

**Figure 5:**
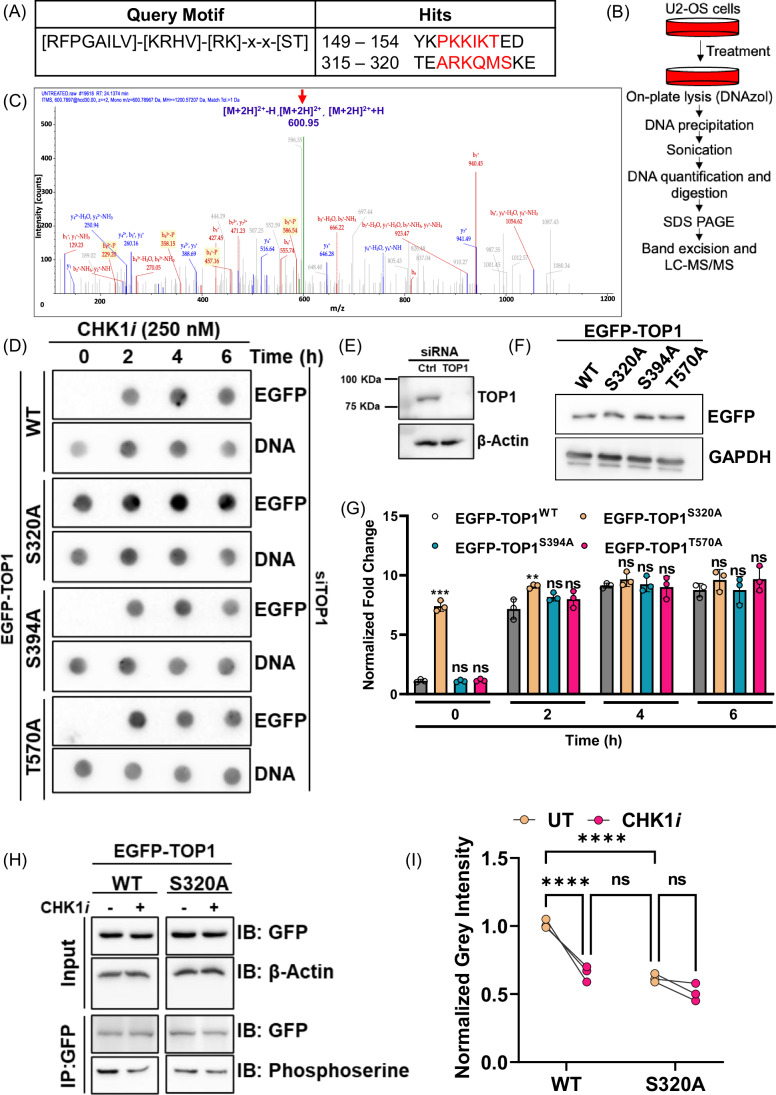
CHK1 regulates TOP1cc kinetics through phosphorylation of TOP1 at Serine 320. (**A**) Predicted CHK1 target motifs on TOP1. (**B**) Experimental workflow for mass spectrometric detection of post-translational modifications (PTMs) on catalytically engaged TOP1. (**C**) Representative spectra of a peptide encompassing the phosphorylated Serine 320 residue on TOP1. (**D**) Representative RADAR assay of U2-OS cells transfected with EGFP-TOP1^WT^, EGFP-TOP1^S320A^, EGFP-TOP1^S394A^, or EGFP-TOP1^T570A^ post siRNA-mediated depletion of endogenous TOP1. Cells were transfected with siRNA targeting TOP1 3’UTR, followed by transfection with plasmids expressing EGFP-TOP1^WT^, EGFP-TOP1^S320A^, EGFP-TOP1^S394A^, or EGFP-TOP1^T570A^ for 24 h. Subsequently, cells were subjected to treatment with CHK1*i* (250 nM) for 2, 4, or 6 h. RADAR assay was carried out to analyze EGFP-TOP1ccs with anti-GFP antibody. (**E**) Representative immunoblot of TOP1 from cells transfected with control siRNA or siRNA targeting TOP1 3’UTR. (**F**) Representative immunoblot with EGFP antibody showing expression levels of EGFP-TOP1^WT^, EGFP-TOP1^S320A^, EGFP-TOP1^S394A^, or EGFP-TOP1^T570A^. (**G**) Densitometric quantification across three replicates of the experiment shown in (**D**). *P* values are: EGFP-TOP1^WT^ *vs.* EGFP-TOP1^S320A^ (0 h) <0.0001, EGFP-TOP1^WT^ *vs.* EGFP-TOP1^S394A^ (0 h) 0.9987, EGFP-TOP1^WT^ *vs.* EGFP-TOP1^T570A^ (0 h) 0.9996, EGFP-TOP1^WT^ *vs.* EGFP-TOP1^S320A^ (2 h) 0.0051, EGFP-TOP1^WT^ *vs.* EGFP-TOP1^S394A^ (2 h) 0.2435, EGFP-TOP1^WT^ *vs.* EGFP-TOP1^T570A^ (2 h) 0.4001, EGFP-TOP1^WT^ *vs.* EGFP-TOP1^S320A^ (4 h) <0.7601, EGFP-TOP1^WT^ *vs.* EGFP-TOP1^S394A^ (4 h) 0.9964, EGFP-TOP1^WT^ *vs.* EGFP-TOP1^T570A^ (4 h) 0.9951, EGFP-TOP1^WT^ *vs.* EGFP-TOP1^S320A^ (6 h) 0.3871, EGFP-TOP1^WT^ *vs.* EGFP-TOP1^S394A^ (6 h) 0.9991, EGFP-TOP1^WT^ *vs.* EGFP-TOP1^T570A^ (6 h) 0.3332 (data from three independent experiments; significance was determined using one-way ANOVA). (**H**, **I**) Representative immunoblot showing immunoprecipitation (and densitometric quantification) of EGFP-TOP1^WT^ and EGFP-TOP1^S320A^ from cells subjected to no treatment or treatment with CHK1*i* (250 nM). Cells were transfected with relevant plasmids and subjected to treatment 48 h post transfection. Immunoprecipitated samples were probed with anti-GFP, and anti-phosphoserine antibodies. *P* values are: EGFP-TOP1^WT^ Untreated *vs.* EGFP-TOP1^WT^ + CHK1*i* < 0.0001, EGFP-TOP1^WT^ Untreated *vs.* EGFP-TOP1^S320A^ Untreated <0.0001, EGFP-TOP1^WT^ + CHK1*i* *vs.* EGFP-TOP1^S320A^ Untreated 0.7946, EGFP-TOP1^S320A^ Untreated *vs.* EGFP-TOP1^S320A^ + CHK1*i* 0.1044 (data from three independent experiments; significance was determined using two-way ANOVA with Tukey test). Error bars represent SD. Source data are available online for this figure.

Guided by the validated CHK1–TOP1 interaction, we next searched for potential CHK1 phosphorylation sites on TOP1. Using the CHK1-specific serine/threonine motifs defined Blasius and coworkers (Blasius et al, 2011) and the Prosite server (https://prosite.expasy.org/), we identified two candidate sites, T154 and S320, neither previously assigned to any kinase (Fig. 5A). A second search using a minimal CHK1 consensus uncovered additional sites: S394 and T570 alongside T154 and S320 (Appendix Table S2).

To map phosphorylation events under physiological conditions, we purified catalytically engaged TOP1ccs from untreated, CHK1*i*- or CPT-treated U2-OS cells *via* the DUST workflow and analyzed them by tandem mass spectrometry (Fig. 5B). MS analysis led to the detection of multiple phosphorylation sites (Y231, S250, S320, S394, T446, Y461, Y480, S534, T570, Y538, Y706) in untreated or treated samples (CHK1*i*, CPT) at different time points (Appendix Table S3; Fig. 5C), reflecting a vibrant spectrum of phosphorylation events associated with catalytically engaged TOP1. Among four predicted CHK1-target sites (T154, S320, S394, and T570; inclusive of both analyses referred to above), T154 was not detected in our MS-analysis.

**Figure EV2:**
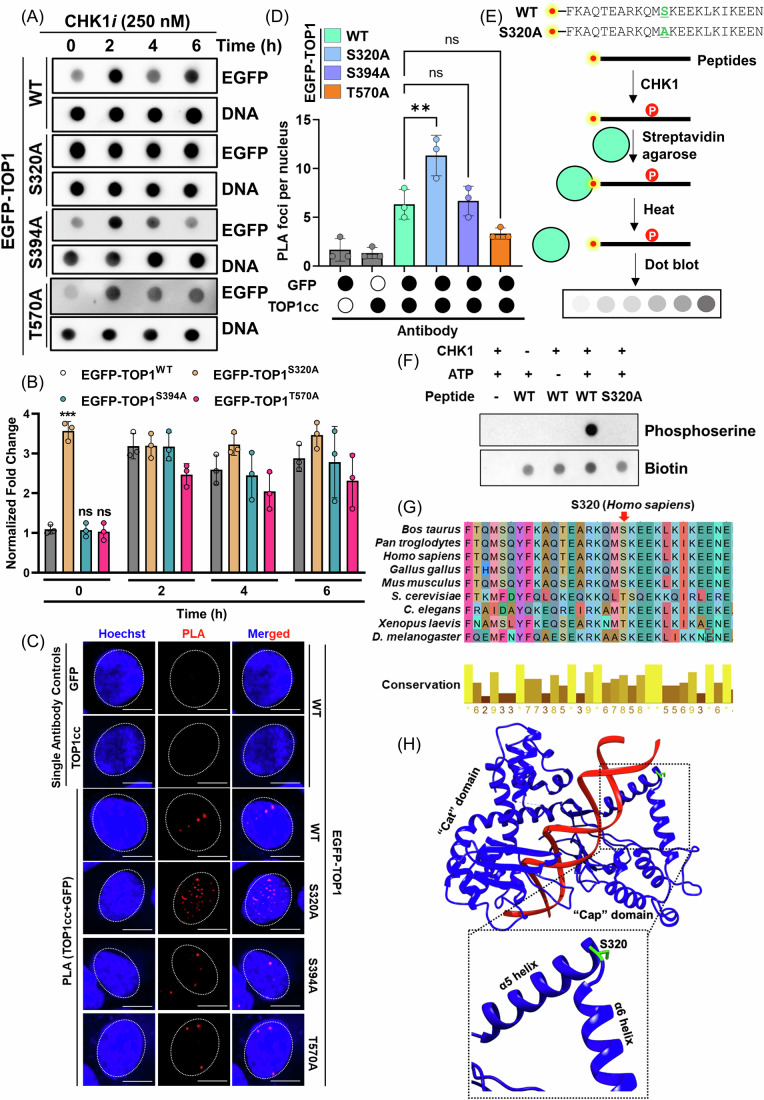
Characterization of S320 phosphorylation as a regulator of TOP1 dynamics. (**A**, **B**) Representative RADAR assay in U2-OS cells transfected with EGFP-TOP1^WT^, EGFP-TOP1^S320A^, EGFP-TOP1^S394A^, or EGFP-TOP1^T570A^ without depletion of endogenous TOP1. Cells were transfected with plasmids expressing EGFP-TOP1^WT^, EGFP-TOP1^S320A^, EGFP-TOP1^S394A^, or EGFP-TOP1^T570A^ followed by treatment with CHK1*i* (250 nM) for 2, 4, or 6 h, 48 h post transfection. Dot blots were probed with anti-GFP antibody. *P* values (0 h) are: 0.007925 (EGFP-TOP1^WT^ *vs.* EGFP-TOP1^S320A^), 0.966818 (EGFP-TOP1^WT^ *vs.* EGFP-TOP1^S394A^), and 0.076058 (EGFP-TOP1^WT^ *vs.* EGFP-TOP1^T570A^) (data from three independent experiments; significance was determined using two-way ANOVA with Tukey test). Error bars represent SD. (**C**, **D**) Representative Proximity Ligation Assay (PLA) images (and quantification across three replicates) of EGFP and TOP1cc in U2-OS cells transfected with EGFP-TOP1^WT^, EGFP-TOP1^S320A^, EGFP-TOP1^S394A^, or EGFP-TOP1^T570A^. Cells were transfected, followed by PLA with anti-GFP and anti-TOP1cc antibody 48 h post transfection. Nuclei are marked with white dotted lines. Scale bars: 10 µm). *P* values are: 0.0011598 (EGFP-TOP1^WT^ *vs.* EGFP-TOP1^S320A^), 0.114290 (EGFP-TOP1^WT^ *vs.* EGFP-TOP1^S394A^), and 0.084425 (EGFP-TOP1^WT^ *vs.* EGFP-TOP1^T570A^) (data from three independent experiments with 200 cells per condition; significance was determined using Kruskal–Wallis test with Dunn’s post hoc analysis). Error bars represent SEM. (**E**, **F**) In vitro kinase assay with recombinant human CHK1 and biotinylated peptides encompassing TOP1 S320 and its phosphoresistant mutant S320A. (**F**) Multiple sequence alignment of TOP1 orthologs from nine eukaryotic species, highlighting conservation of Serine 320. WT and S320A peptides (amino acid highlighted in green) were biotinylated at their N-termini (red). In vitro kinase reactions were performed with recombinant CHK1 and relevant peptides, followed by purification of peptides *via* streptavidin agarose beads (green). Peptides were then dissociated from the beads and subjected to a dot blot. (**G**) Structural context of Serine 320 in the TOP1-DNA complex (PDB ID:1A31). (**H**) Structural context of Serine 320 in TOP1 protein (PDB ID: 1A31). Positioning of Serine 320 relative to α5 and α6 “nose-cone” helices is shown in inset. Source data are available online for this figure.

We then tested the functional relevance of S320, S394, and T570 by generating phospho-resistant EGFP-TOP1 mutants (S320A, S394A, T570A) and tracking their chromatin association using the RADAR assay (with anti-GFP antibody), with or without siRNA-mediated depletion of endogenous TOP1 (Figs. 5D–F and EV2A,B, respectively). EGFP-TOP1^WT^ mirrored the dynamics of endogenous TOP1 in response to CHK1*i* (Figs. 5D and Fig. EV2A vs Fig. 1D). Notably, EGFP-TOP1^S320A^ was found to be constitutively entrapped on the genome (Figs. 5D and EV2A), which was not appreciably enhanced upon CHK1*i* treatment. In contrast, the kinetics of EGFP-TOP1^S394A^ and EGFP-TOP1^T570A^ were essentially identical to that of EGFP-TOP1^WT^ under both untreated and CHK1*i*-treated conditions (Figs. 5D and EV2A). This phenocopy of CHK1 inhibition by TOP1^S320A^ indicates that S320 phosphorylation is pivotal for CHK1-dependent control of TOP1cc levels, while phosphorylation at S394 and T570 is likely to be dispensable under basal conditions. This observation was independently confirmed using PLA with anti-GFP and anti-TOP1cc antibody. PLA confirmed that under unperturbed conditions, EGFP-TOP1^S320A^ (but not EGFP-TOP1^S394A^ or EGFP-TOP1^T570A^) was constitutively trapped on the genome (Fig. EV2C,D). In order to further strengthen the validity of S320 as the target site of CHK1-mediated phosphorylation, we conducted both in vitro and in cellulo experiments. First, we performed in vitro kinase assays with a peptide encompassing TOP1 S320 (and its complementary mutant peptide bearing S320A) (Fig. EV2E). TOP1 S320 peptide was strongly phosphorylated by CHK1, which was completely abolished in the mutant peptide (Fig. EV2F), demonstrating that TOP1 S320 is indeed a CHK1 substrate. Next, we transfected U2-OS cells with EGFP-TOP1^WT^ and EGFP-TOP1^S320A^ and probed their phosphoserine levels under ambient or CHK1*i*-treated conditions. Our results led to a few key observations. First, EGFP-TOP1^S320A^ was found to be significantly less phosphorylated compared to EGFP-TOP1^WT^ under ambient conditions, suggesting that TOP1 is indeed phosphorylated at Serine 320 at the cellular level (Fig. 5H,I). Second, CHK1*i* treatment led to a significant reduction in EGFP-TOP1^WT^ phosphorylation, suggesting that TOP1 is a CHK1 target inside cells (Fig. 5H,I). Finally, and interestingly, while CHK1*i* suppressed phosphorylation of EGFP-TOP1^WT^, it failed to do so in the case of EGFP-TOP1^S320A^ (Fig. 5H,I), which compellingly demonstrated that CHK1 phosphorylates TOP1 at Serine 320 position at the cellular level.

Given the evolutionary conservation of CHK1 and TOP1 across species (Patil et al, 2013; Moreira et al, 2023), a physiologically relevant TOP1–CHK1 interaction is expected to be reflected in pronounced conservation with respect to the CHK1 target site on TOP1. Hence, we performed a multiple sequence alignment of TOP1 from 9 different species, ranging from yeast to humans. Interestingly, a high degree of conservation was observed at the S320 position, with either Serine or Threonine at S320 position (Fig. EV2G). Moreover, this conservation was strongly reflected between −5 and +3 residues with respect to Serine/Threonine 320 (Fig. EV2G), reflecting evolutionary conservation across the entire CHK1 target site. Structurally, S320 is strategically located in a connecting loop between the α5 and α6 helices, which constitute the “nose-cone” resident within the “Cap” domain of TOP1 (Fig. EV2H). The nose-cone helices have been shown to make multiple contacts with the substrate downstream of the scissile site, hence playing an indispensable role in the TOP1 catalytic cycle (Carey et al, 2003; Stewart et al, 1998; Chillemi et al, 2001). Together, these findings identify S320 as a conserved CHK1 phosphorylation site that fine-tunes TOP1 dynamics in cells.

### CHK1-mediated TOP1-S320 phosphorylation acts as a kinetic regulator of TOP1 religation activity

**Figure 6:**
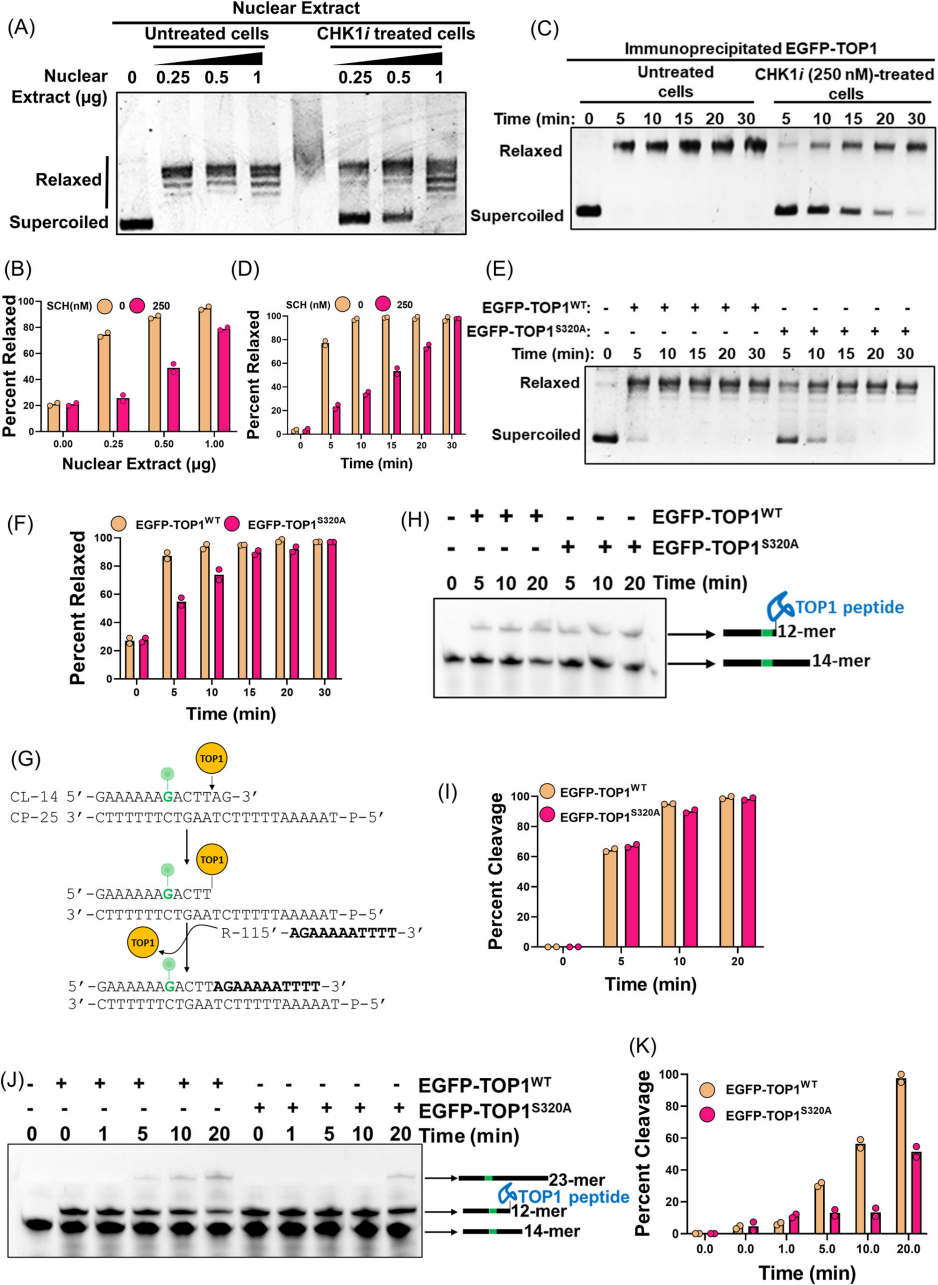
CHK1-mediated TOP1 S320 phosphorylation acts as a kinetic regulator of TOP1 catalytic activity. (**A**, **B**) Plasmid relaxation assay with crude nuclear extracts from U2-OS cells, untreated, or treated with CHK1*i* (250 nM) for 2 h (data from two independent replicates). (**C**, **D**) Plasmid relaxation assay with immunoprecipitated EGFP-TOP1^WT^. U2-OS cells were transiently transfected with EGFP-TOP1^WT^. 48 h post transfection, cells were treated with CHK1*i* (250 nM) for 2 h, followed by immunoprecipitation of EGFP-TOP1 with GFP-trap magnetic agarose beads (from untreated or CHK1*i*-treated cells). Plasmid relaxation assays were performed with the isolated immunocomplexes (data from two independent replicates). (**E**, **F**) Plasmid relaxation assay with EGFP-TOP1^WT^ and EGFP-TOP1^S320A^. U2-OS cells were transiently transfected with EGFP-TOP1^WT^ and EGFP-TOP1^S320A^, followed by immunoprecipitation 48 h post transfection. Plasmid relaxation assay was performed with the isolated immunocomplexes. All plasmid relaxation assays were performed with supercoiled pUC19 substrate (data from two independent replicates). (**G**) Schematic representation of TOP1 cleavage and religation assays. The CL-14 oligonucleotide was FITC labeled at the guanine residue highlighted in green. The CP-25 oligonucleotide was phosphorylated at 5’ end. See “Methods” for more details. (**H**, **I**) Time kinetics of TOP1 cleavage assay with immunoprecipitated EGFP-TOP1^WT^ and EGFP-TOP1^S320A^. EGFP-TOP1^WT^ and EGFP-TOP1^S320A^ were ectopically expressed in U2-OS cells and immunoprecipitated as above. The first lane (from left) shows the unreacted 14-mer (data from two independent replicates). (**J**, **K**) Time kinetics of TOP1 religation assay with immunoprecipitated EGFP-TOP1^WT^ and EGFP-TOP1^S320A^. EGFP-TOP1^WT^ and EGFP-TOP1^S320A^ were ectopically expressed in U2-OS cells and immunoprecipitated as above. The first lane (from left) shows the unreacted substrate (14-mer). The oligonucleotide species represented in each band are schematically shown along the gel images (data from two independent replicates). Source data are available online for this figure.

Given that CHK1 phosphorylates TOP1 and loss of this modification stabilizes TOP1ccs, we asked whether CHK1 controls TOP1 catalysis. To test this, we performed plasmid relaxation assays with crude nuclear extracts prepared from untreated and CHK1*i*-treated U2-OS cells. CHK1*i*-treatment resulted in a marked reduction in cellular TOP1 catalytic activity (Fig. 6A,B). Importantly, CHK1*i* did not directly inhibit purified recombinant human TOP1 (Appendix Fig. S9A,B), ruling out non-specific effects on the enzyme.

To directly assess the enzymatic activity of TOP1, we immunoprecipitated EGFP-TOP1^WT^ from untreated and CHK1*i*-treated cells, followed by a plasmid relaxation assay with these immunocomplexes. Equivalent protein expression and pulldown efficiencies were confirmed by immunoblotting and fluorescence microscopy (Appendix Fig. S9C,D). TOP1 immunocomplexes from CHK1*i*-treated cells displayed drastically reduced relaxation kinetics (Fig. 6C,D), consistent with CHK1-dependent modulation of TOP1 catalysis.

We next investigated whether this regulation of TOP1 by CHK1 is mediated by phosphorylation at S320. Hence, EGFP-TOP1^WT^ and EGFP-TOP1^S320A^ were ectopically expressed in U2-OS cells (Appendix Fig. S9E), immunoprecipitated (Appendix Fig. S9F), and their relative catalytic activities were assessed employing a plasmid relaxation assay. TOP1^S320A^ showed markedly slower catalytic kinetics relative to TOP1^WT^ (Fig. 6E,F). mirroring the effect of CHK1 inhibition and reinforcing S320 as a key regulatory site.

Results of the RADAR assay suggested that CHK1 inhibition is likely to compromise the TOP1 religation reaction rather than cleavage, given the copious generation of TOP1ccs. However, we dissected cleavage as well as religation kinetics of EGFP-TOP1^WT^ and EGFP-TOP1^S320A^, which were ectopically expressed (Appendix Fig. S9G) and immunoprecipitated (Appendix Fig. S9H) from U2-OS cells. Cleavage assay utilizing a fluorescent suicide substrate (Fig. 6G) revealed that EGFP-TOP1^S320A^ shows no significant impairment of cleavage activity compared to EGFP-TOP1^WT^ (Fig. 6H,I), confirming our hypothesis. Next, we tested the religation efficiencies of EGFP-TOP1^WT^ and EGFP-TOP1^S320A^. Measurement of religation kinetics (Fig. 6G) revealed that EGFP-TOP1^S320A^ displays a distinct impairment of religation compared to EGFP-TOP1^WT^ (Fig. 6J,K), thereby establishing S320 phosphorylation as a kinetic “switch” that modulates TOP1cc persistence through direct regulation of the efficiency of the religation reaction. Together, these findings establish CHK1-mediated phosphorylation of TOP1 at S320 as a pivotal regulatory mechanism that safeguards TOP1 catalytic efficiency and prevents TOP1cc accumulation under physiological conditions.

### CHK1-mediated TOP1 regulation limits replication-associated genomic instability

**Figure EV3:**
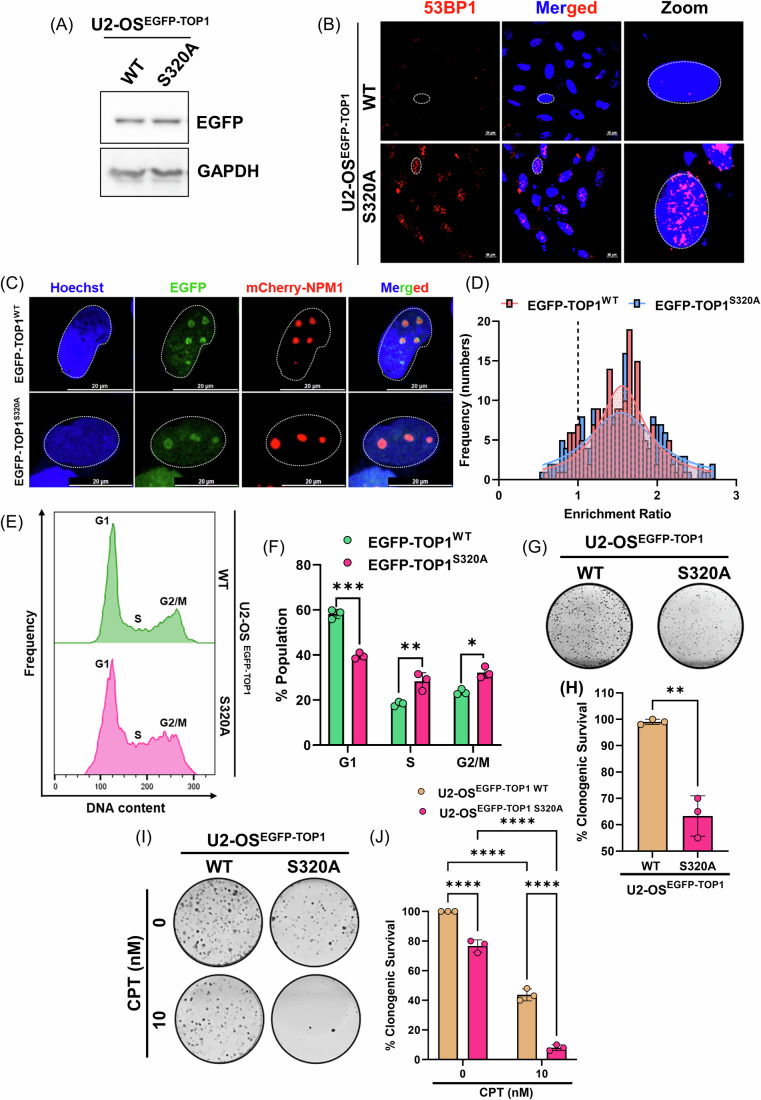
Characterization of U2-OS cells stably expressing EGFP-TOP1^WT^ and EGFP-TOP1^S320A^. (**A**) Immunoblot showing expression of EGFP-TOP1^WT^ and EGFP-TOP1^S320A^ in U2-OS^EGFP-TOP1WT^ and U2-OS^EGFP-TOP1S320A^ stable cells, respectively. (**B**) Representative immunofluorescent microscopic images of 53BP1 levels in U2-OS^EGFP-TOP1WT^ and U2-OS^EGFP-TOP1S320A^ cells. See Fig. 7C for quantification. Nuclei are marked with white dotted lines. Scale bars: 10 µm. (**C**) Nuclear distribution of EGFP-TOP1^WT^ and EGFP-TOP1^S320A^ in U2-OS^EGFP-TOP1WT^ and U2-OS^EGFP-TOP1S320A^ cells, respectively. U2-OS^EGFP-TOP1WT^ and U2-OS^EGFP-TOP1S320A^ cells were transfected with mCherry-NPM1, followed by live cell imaging. Nuclei are marked with white dotted lines. Scale bars: 20 µm. (**D**) Quantification of the experiment shown in (**C**). Refer to the “Methods” section for details on the calculation of the enrichment ratio. Two hundred cells were acquired per condition. (**E**, **F**) Flow cytometric analysis (and quantification thereof) of cell cycle distribution of U2-OS^EGFP-TOP1WT^ and U2-OS^EGFP-TOP1S320A^ cells. *P = 0.012348, **P = 0.005529, ***P = 0.000197 (data from three independent experiments; significance was determined using two-way ANOVA with Tukey test). Error bars represent SD. (**G**, **H**) Clonogenic survival of U2-OS^EGFP-TOP1WT^ and U2-OS^EGFP-TOP1S320A^ cells. U2-OS^EGFP-TOP1WT^ and U2-OS^EGFP-TOP1S320A^ cells were seeded in six-well plates, followed by enumeration of resultant colonies post 7–8 days of incubation. Percentage survival was calculated with respect to the number of colonies in U2-OS^EGFP-TOP1WT^ cells. **P = 0.001311 (data from three independent experiments; significance was determined using unpaired *t* test). (**I**, **J**) Sensitivity of U2-OS^EGFP-TOP1WT^ and U2-OS^EGFP-TOP1S320A^ cells toward CPT. U2-OS^EGFP-TOP1WT^ and U2-OS^EGFP-TOP1S320A^ cells were seeded in six-well plates, followed by treatment with CPT (10 nM) for 16 h post seeding. Resultant colonies were enumerated after 7–8 days of incubation. ****P < 0.0001 (data from three independent experiments; significance was determined using two-way ANOVA with Tukey test). Error bars represent SD. Source data are available online for this figure.

**Figure 7:**
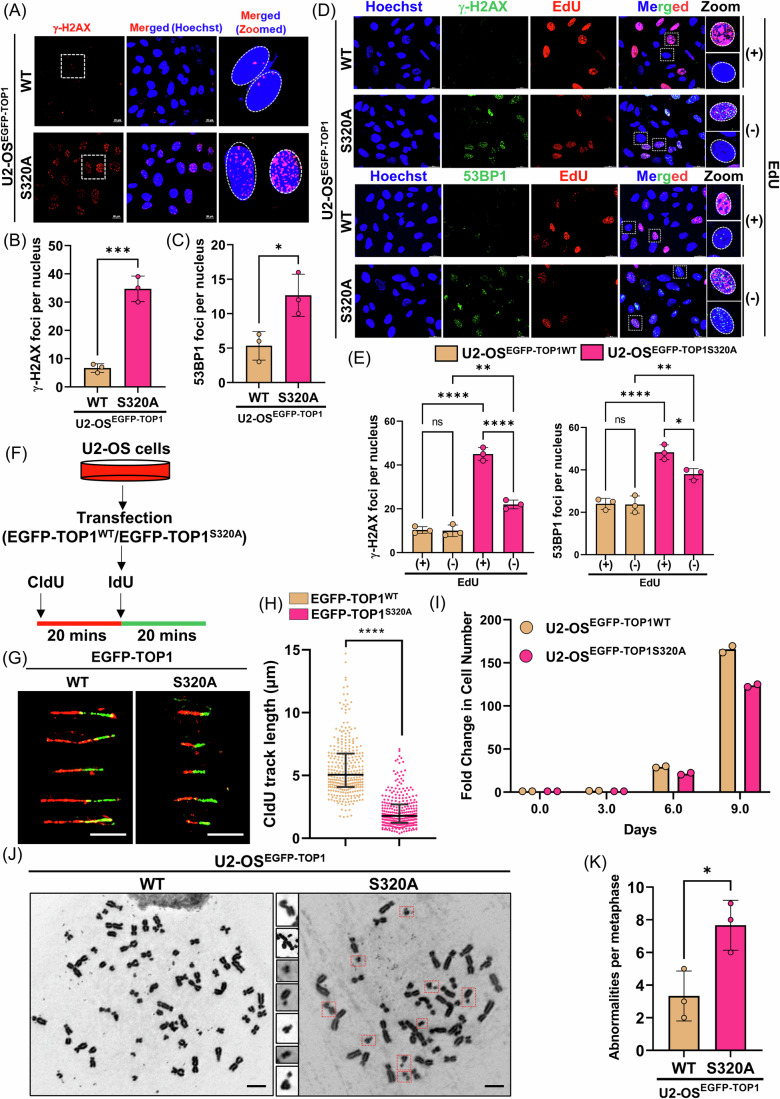
Ablation of CHK1-mediated TOP1 S320 phosphorylation induces replication-associated genomic instability. (**A**) Representative microscopic images of γ-H2AX levels in U2-OS^EGFP-TOP1WT^ and U2-OS^EGFP-TOP1S320A^ stable cells. Cells in white dotted boxes are shown in the zoomed panel. Scale bars: 20 µm. (**B**, **C**) Quantification of γ-H2AX and 53BP1 foci in U2-OS^EGFP-TOP1WT^ and U2-OS^EGFP-TOP1S320A^ stable cells. ***P = 0.0005, *P = 0.0264 (data from three independent experiments with 200 cells per condition; significance was determined using unpaired *t* test). Error bars represent SEM. (**D**, **E**) Representative microscopic images (and quantification across three replicates) showing colocalization of γ-H2AX/53BP1 foci with EdU in U2-OS^EGFP-TOP1WT^ and U2-OS^EGFP-TOP1S320A^ cells. Cells were pulse-labeled with EdU (10 µM; 20 min) prior to fixation. γ-H2AX/53BP1 were visualized by immunofluorescence, while EdU was detected using click chemistry. Scale bars: 20 µm. Cells in white dotted boxes are shown in the zoomed panel. For γ-H2AX foci, *P* values are: 0.9980 (U2-OS^EGFP-TOP1WT^ EdU^+^ *vs.* EdU^-^), < 0.0001 (U2-OS^EGFP-TOP1WT^ EdU^+^ *vs.* U2-OS^EGFP-TOP1S320A^ EdU^+^), 0.0011 (U2-OS^EGFP-TOP1WT^ EdU^−^ *vs.* U2-OS^EGFP-TOP1S320A^ EdU^−^), < 0.0001 (U2-OS^EGFP-TOP1S320A^ EdU^+^ *vs.* EdU^-^). For 53BP1 foci, ns *P* = 0.9992, *P = 0.0197, **P = 0.0030, ****P < 0.0001 (data from three independent experiments with 200 cells per condition; significance was determined using Kruskal–Wallis test with Dunn’s post hoc analysis). Error bars represent SEM. (**F**–**H**) Single-molecule DNA fiber assay in U2-OS cells transiently transfected with EGFP-TOP1^WT^ and EGFP-TOP1^S320A^. Five representative DNA fibers have been shown per sample. Scale bars: 5 µm. In total, 250–300 individual fibers were scored from a total of 15 fields per sample. Quantification pertaining to the experiment shown in (**G**) is presented. Error bars represent median±interquartile range. ****P < 0.0001. See Appendix Fig. S11C for quantification of all replicates. (**I**) Proliferation kinetics of U2-OS^EGFP-TOP1WT^ and U2-OS^EGFP-TOP1S320A^ stable cells. Cells were seeded in 60 mm dishes and cell number was quantified 3-, 6-, or 9-days post seeding (data from two independent replicates). (**J**, **K**) Representative metaphase spreads (and quantification across three replicates, with 100 metaphases per condition) prepared from U2-OS^EGFP-TOP1WT^ and U2-OS^EGFP-TOP1S320A^ cells. Scale bars: 10 µm. Error bars represent SEM. **P = 0.0255 (Kruskal–Wallis test with Dunn’s post hoc analysis). Source data are available online for this figure.

TOP1cc stabilization is a well-established source of genomic instability (Pommier et al, 2022; Duardo et al, 2024). Since TOP1^S320A^ (which ablates CHK1-mediated phosphorylation) is catalytically impaired and constitutively trapped on DNA, we hypothesized that CHK1-dependent TOP1 phosphorylation might protect cells from TOP1cc-induced genomic instability. To test this, we examined basal DNA damage in U2-OS sublines stably expressing EGFP-TOP1^WT^ and EGFP-TOP1^S320A^ (U2-OS^EGFP-TOP1WT^ and U2-OS^EGFP-TOP1S320A^, respectively) (Appendix Fig. S10A,B; Fig. EV3A). U2-OS^EGFP-TOP1S320A^ cells displayed significantly higher levels of γ-H2AX as well as 53BP1 foci, indicative of higher endogenous DNA damage (Figs. 7A–C and EV3B). It is interesting to note that while low-concentration CHK1*i* exposure did not result in appreciable DSB induction at early timepoints (Appendix Fig. S4), constitutive expression of EGFP-TOP1^S320A^ results in copious amounts of DNA damage. This observation highlights the key influence of the length as well as the nature of TOP1cc persistence on DSB induction. While TOP1cc stabilization does not cause significant DNA damage at early timepoints (up to 6 h), constitutive stabilization of TOP1ccs resulting from impaired catalytic activity of TOP1^S320A^ for extended periods results in widespread DNA damage. This further highlights a key distinction between CPT (interfacial inhibition)-induced and CHK1*i*-induced TOP1cc stabilization (which proceeds without formation of a ternary suicide complex). To determine whether this damage was replication-associated, we assessed colocalization of EdU with γ-H2AX or 53BP1 foci in U2-OS^EGFP-TOP1WT^ and U2-OS^EGFP-TOP1S320A^ cells. Replicating (EdU⁺) cells expressing U2-OS^EGFP-TOP1S320A^ showed markedly higher levels of both DNA damage markers (Fig. 7D,E). Consistently, inhibition of DNA replication with aphidicolin or CDC7 inhibition alleviated DNA damage in these cells (Appendix Fig. S10C,D), confirming its replication-associated nature. However, interestingly, both replicating and non-replicating populations of U2-OS^EGFP-TOP1S320A^ cells showed significant elevation in γ-H2AX and 53BP1 levels, suggesting factors both dependent and independent of replication involved in EGFP-TOP1^S320A^-mediated genomic instability (Fig. 7E) (addressed below).

We also observed a conspicuous reduction in EdU incorporation in U2-OS^EGFP-TOP1S320A^ cells compared to U2-OS^EGFP-TOP1WT^ cells (Appendix Fig. S10E–G), consistent with TOP1cc-associated replication impairment (Pommier et al, 2022). Next, we employed a single-molecule DNA fiber assay to directly visualize replication fork dynamics in these cells. DNA fiber analysis revealed severe replication fork slowing in cells expressing EGFP-TOP1^S320A^, evident in drastically shortened fiber tracks (Fig. 7G,H; Appendix Fig. S11A–C). We also characterized the nuclear localization of EGFP-TOP1^WT^ and EGFP-TOP1^S320A^ in U2-OS^EGFP-TOP1WT^ and U2-OS^EGFP-TOP1S320A^ cells, respectively. Both EGFP-TOP1^WT^ and EGFP-TOP1^S320A^ showed similar nuclear distribution with significant enrichment in nucleoli (Fig. EV3C,D). We next evaluated the cell cycle distribution of U2-OS^EGFP-TOP1WT^ and U2-OS^EGFP-TOP1S320A^ cells. U2-OS^EGFP-TOP1S320A^ cells were shown to suffer from relatively higher arrest in S and G2 phases of the cell cycle (Fig. EV3E,F). In concordance with these findings, U2-OS^EGFP-TOP1S320A^ cells displayed reduced colony formation capacity (Fig. EV3G,H), slower proliferation (Fig. 7I), and heightened sensitivity to CPT (Fig. EV3I,J). Finally, metaphase spread analysis revealed increased dicentrics, acentric fragments, and chromatid breaks in U2-OS^EGFP-TOP1S320A^ cells, indicating large-scale genomic instability (Fig. 7J,K).

Together with the preceding results, these findings suggest that although CHK1*i*-induced TOP1cc stabilization is not overtly replication-dependent, the downstream consequences of persistent TOP1ccs are strongly replication-linked. Accordingly, while CHK1 inhibition does not correlate with EdU incorporation (Fig. 2A), TOP1^S320A^ expression clearly links DNA damage to replication status (Fig. 7D). Thus, CHK1-mediated phosphorylation of TOP1 at S320 emerges as a critical protective mechanism that limits replication stress and preserves genome stability.

### CHK1-mediated TOP1 phosphorylation suppresses R-loops and transcription–replication conflicts

**Figure 8:**
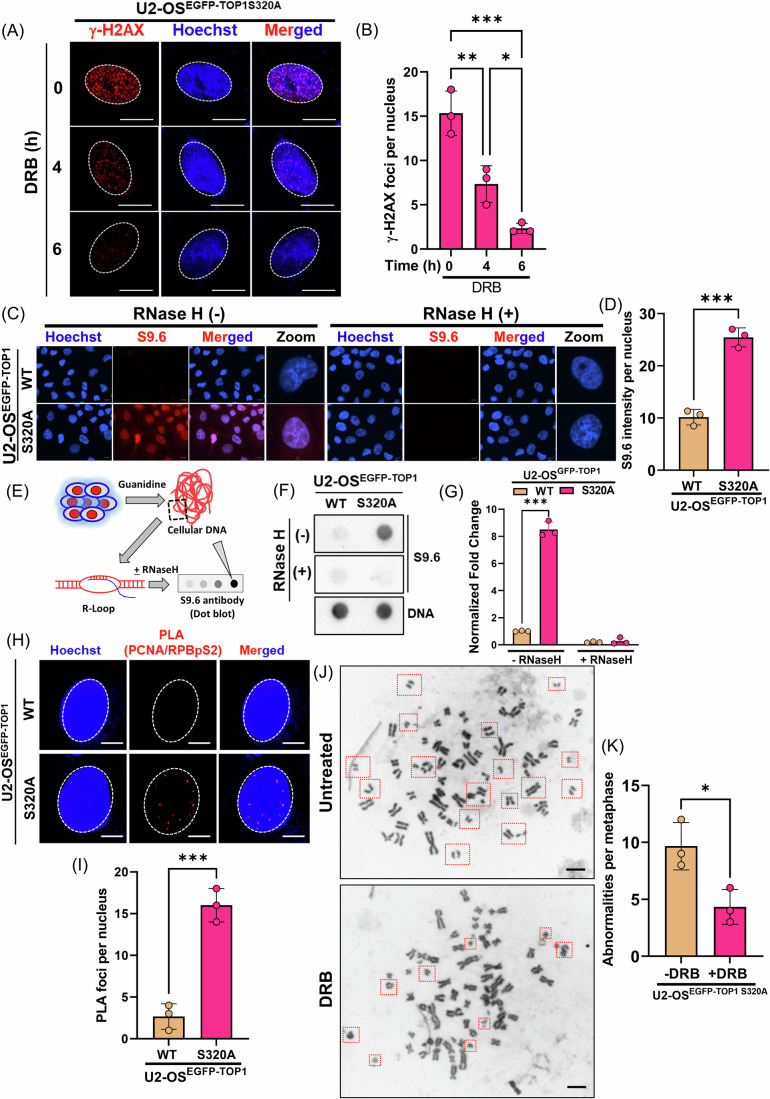
Transcription-associated DNA damage, R-loop accumulation, and transcription replication conflicts upon ablation of CHK1-mediated TOP1 phosphorylation. (**A**, **B**) Representative microscopic images (and quantification across three replicates) of rescue of DNA damage in U2-OS^EGFP-TOP1S320A^ cells in response to the transcription inhibitor DRB. U2-OS^EGFP-TOP1S320A^ cells were treated with DRB (100 µM) for 4 or 6 h, followed by immunofluorescent detection of γ-H2AX foci. Nuclei are marked with white dotted lines. *P = 0.0427, **P = 0.0052, ***P = 0.0003 (data from three independent replicates with 200 cells per condition; significance was determined using Kruskal–Wallis test with Dunn’s post hoc analysis). Scale bars: 10 µm. Error bars represent SEM. (**C**, **D**) Representative microscopic images (and quantification across three replicates) of RNA/DNA hybrid (R-loop) levels in U2-OS^EGFP-TOP1WT^ and U2-OS^EGFP-TOP1S320A^ stable cells. R-loops were immunodetected with the S9.6 monoclonal antibody. Signal specificity was determined using RNase H treatment prior to incubation with the primary antibody. ***P = 0.0004 (data from three independent replicates with 200 cells per condition; significance was determined using Kruskal–Wallis test with Dunn’s post hoc analysis). Scale bars: 10 µm. Error bars represent SEM. (**E**–**G**) Dot blot of R-loop levels in U2-OS^EGFP-TOP1WT^ and U2-OS^EGFP-TOP1S320A^ stable cells. ***P = 0.000172 (data from three independent experiments. Significance was determined using one-way ANOVA). Error bars represent SD. (**H**, **I**) Representative microscopic images (and quantification across three replicates) of Proximity ligation assay (PLA) for detection of transcription–replication collisions (TRCs) in U2-OS^EGFP-TOP1WT^ and U2-OS^EGFP-TOP1S320A^ stable cells. PLA was performed with PCNA and RNA pol II large subunit phospho-serine 2 antibodies. Nuclei are marked with white dotted lines. ***P = 0.0008 (data from three independent experiments with 75 cells per condition; significance was determined using Kruskal–Wallis test with Dunn’s post hoc analysis). Scale bars: 5 µm. Error bars represent SEM. (**J**, **K**) Representative metaphase spreads (and quantification) prepared from U2-OS^EGFP-TOP1S320A^ cells with or without treatment with DRB. U2-OS^EGFP-TOP1S320A^ cells were treated with DRB (20 µM) for 16 h, followed by preparation of metaphase spreads. Aberrant chromosomal structures are highlighted in red dotted boxes. *P = 0.0232 (data from three independent experiments with 100 metaphases per condition). Scale bars: 10 µm. Error bars represent SEM. Source data are available online for this figure.

TOP1ccs stabilized through TOP1 poisoning are known to promote R-loop accumulation and R-loop–associated genomic instability (Cristini et al, 2019; Hidmi et al, 2024; Duardo et al, 2024). Consistent with this, EU incorporation assays revealed mildly (yet significantly) reduced global transcription in U2-OS^EGFP-TOP1S320A^ cells (Appendix Fig. S12A,B). To test whether the catalytic impairment of TOP1^S320A^ translates into transcription-dependent DSBs, we monitored γ-H2AX levels in U2-OS^EGFP-TOP1S320A^ cells in the presence or absence of the transcription inhibitor DRB. Treatment with DRB suppressed DNA damage in U2-OS^EGFP-TOP1S320A^ cells in a time-dependent manner (Fig. 8A,B), indicating that constitutive genomic stabilization of TOP1^S320A^ indeed results in transcription-dependent DNA damage. Further, this DRB-induced rescue of DNA damage was visible in both replicating (EdU^+^) as well as non-replicating (EdU^-^) cells (Appendix Fig. S12C,D), consistent with our findings reported above (Fig. 7E). These two observations, taken together, strongly demonstrate the replication as well as transcription-associated nature of EGFP-TOP1^S320A^-associated DNA damage. Also, notably, while TOP1cc stabilization itself is not transcription-dependent (Fig. 2H), transcription clearly contributes to the conversion of TOP1ccs into DNA damage (Fig. 8A,B).

We next assessed global R-loop levels in U2-OS^EGFP-TOP1S320A^ cells. Immunofluorescent detection with R-loop-specific S9.6 antibody revealed significantly elevated R-loops in U2-OS^EGFP-TOP1S320A^ cells (Fig. 8C,D). This observation was also independently corroborated with dot blot (Fig. 8E–G). Since CPT-induced TOP1cc stabilization has been shown to trigger transcription–replication collisions (TRCs) (Chappidi et al, 2020), and given our earlier observation of replication and transcription-associated DNA damage in U2-OS^EGFP-TOP1S320A^ cells (Figs. 7D and 8A), we asked whether TRCs underlie the transcription-associated damage observed in our experiments. Proximity ligation assays (PLA) between PCNA (replisome marker) and elongating RNA Pol II (pSer2) revealed a striking increase in TRCs in U2-OS^EGFP-TOP1S320A^ cells relative to U2-OS^EGFP-TOP1WT^ cells (Fig. 8H,I). Finally, in order to establish the role played by transcription-associated DNA damage and R-loops in the genomic instability of U2-OS^EGFP-TOP1S320A^ cells, we treated U2-OS^EGFP-TOP1S320A^ cells with the transcription inhibitor DRB, followed by preparation of metaphase spreads from these cells. DRB did not significantly affect replication in these cells (Appendix Fig. S13A,B), while drastically reducing R-loop levels (Appendix Fig. S13C, D). Metaphase spreads prepared from these cells showed a significant reduction in chromosomal abnormalities (Fig. 8J,K), thus providing compelling evidence of the critical role played by transcription-associated DNA damage and R-loop accumulation in TOP1^S320A^-associated DNA damage. Taken together, these findings demonstrate that CHK1-mediated phosphorylation of TOP1 at S320 restrains R-loop accumulation, prevents TRCs, and thereby suppresses transcription-associated genomic instability.

### CHK1-mediated TOP1 regulation safeguards transcriptional integrity of long, highly expressed genes

**Figure 9:**
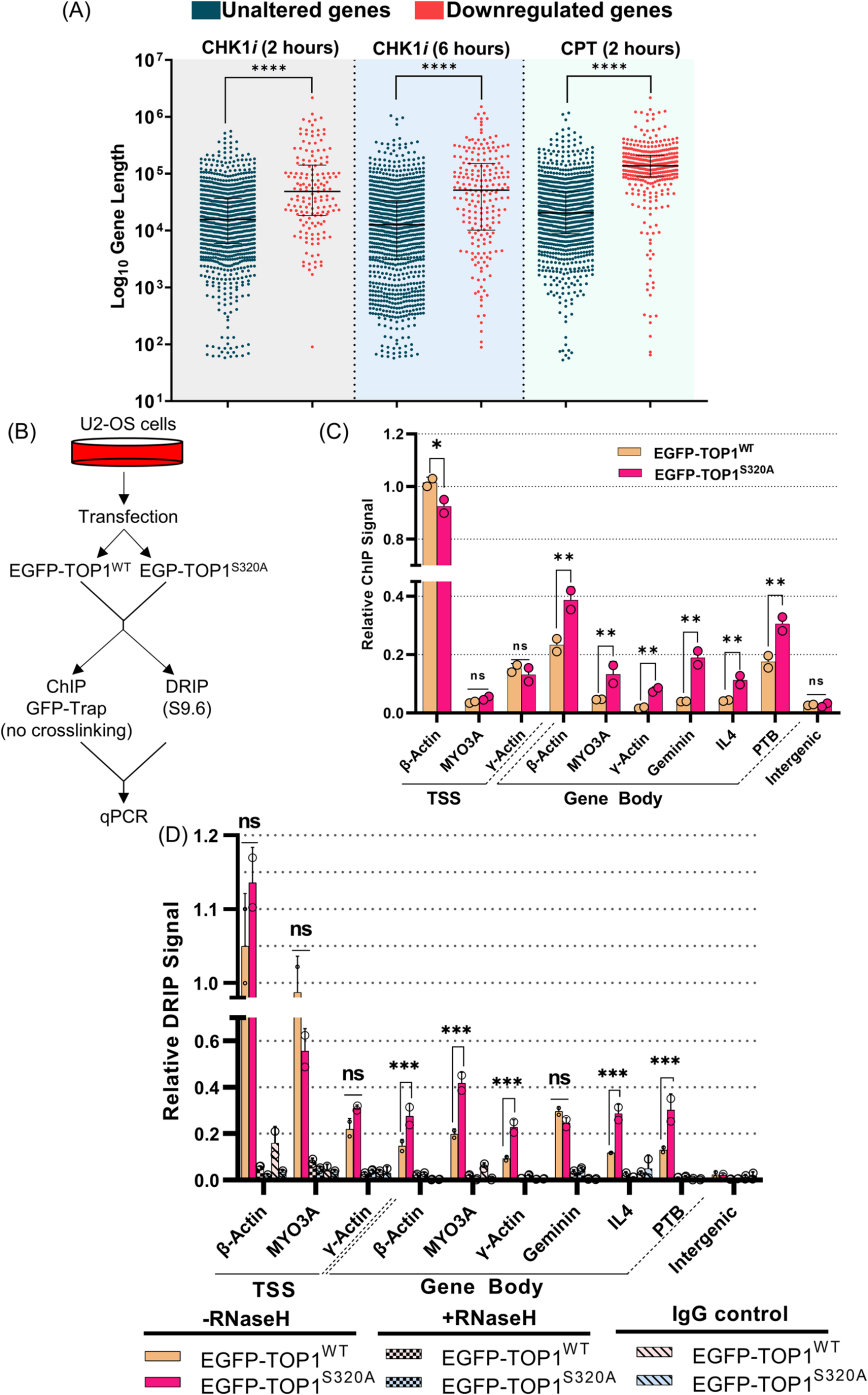
CHK1 suppresses TOP1cc persistence and R-loop accumulation on long genes. (**A**) Analysis of gene lengths from transcriptomic profiling of U2-OS cells treated with CHK1*i* (250 nM) or CPT (1 µM). Quantification shown from one replicate. The experiment was performed in two replicates. ****P < 0.0001. (**B**) Experimental workflow of ChIP and DRIP assays. (**C**) Relative enrichment of TOP1ccs (arising from EGFP-TOP1^WT^ or EGFP-TOP1^S320A^) in transcription start sites (TSS) and gene bodies. U2-OS cells were transiently transfected with EGFP-TOP1^WT^ and EGFP-TOP1^S320A^, followed by chromatin immunoprecipitation with GFP-trap magnetic agarose beads from un-crosslinked nuclei. *P* values (EGFP-TOP1^WT^ *vs.* EGFP-TOP1^S320A^) are: 0.04113 (β-Actin TSS), 0.156727 (MYO3A TSS), 0.511635 (γ-Actin TSS), 0.018755 (β-Actin gene body), 0.010610 (MYO3A gene body), 0.014291 (γ-Actin gene body), 0.014334 (Geminin gene body), 0.014369 (IL4), 0.015524 (PTB), 0.938827 (intergenic) (data from two independent experiments; significance was determined using two-way ANOVA with Tukey test). Error bars represent SEM. (**D**) Relative enrichment of R-loops at TSS and gene bodies. U2-OS cells were transiently transfected with EGFP-TOP1^WT^ and EGFP-TOP1^S320A^, followed by immunoprecipitation with S9.6 monoclonal antibody. Specificity of the signal was determined using RNase H sensitivity. *P* values (EGFP-TOP1^WT^ *vs.* EGFP-TOP1^S320A^) are: 0.291128 (β-Actin TSS), 0.03 (MYO3A TSS), 0.110789 (γ-Actin TSS), 0.009672 (β-Actin gene body), 0.002608 (MYO3A gene body), 0.002977 (γ-Actin gene body), 0.143912 (Geminin gene body), 0.002618 (IL4), 0.007655 (PTB), 0.873055 (intergenic) (data from two independent experiments; significance was determined using two-way ANOVA with Tukey test). Error bars represent SEM. Source data are available online for this figure.

CPT-mediated stabilization of TOP1ccs has been reported to preferentially downregulate long and highly expressed genes (Veloso et al, 2013; Solier et al, 2013). Since loss of CHK1-mediated TOP1 phosphorylation stabilizes TOP1ccs and promotes transcription-associated DNA damage and R-loop accumulation, we next asked whether CHK1 inhibition (CHK1*i*) produces similar transcriptional consequences. To this end, we performed transcriptome-wide analysis (~20,000 genes) in cells treated with CHK1*i* for 2 or 6 h, with CPT (1 μM, 2 h) included as a positive control. Remarkably, CHK1*i* recapitulated CPT-induced preferential downregulation of long and highly expressed genes (Fig. 9A).

To place these findings in a transcriptional context, we examined the genomic occupancy of TOP1^S320A^ using a panel of probes targeting promoter-proximal regions (PPRs) and gene bodies of several well-characterized genes (Cristini et al, 2019) as well as MYO3A (the longest gene identified as CHK1*i*-sensitive, 2.1 Mb). We performed chromatin immunoprecipitation (ChIP) from non-crosslinked nuclei of U2-OS cells expressing EGFP-TOP1^WT^ and EGFP-TOP1^S320A^ in order to probe TOP1cc enrichment on the target genes (Baranello et al, 2016) (Fig. 9B). EGFP-TOP1^S320A^ showed significantly higher occupancy at gene bodies compared with EGFP-TOP1^WT^, whereas no significant difference was observed at promoter-proximal regions (Fig. 9C). Interestingly, this result mirrors the previously reported preferential enrichment of catalytically active TOP1 on gene bodies over promoter-proximal regions (Baranello et al, 2016), which suggests that while EGFP-TOP1^S320A^ retains its catalytic activity, its reduced chromatin dynamics promote entrapment within gene bodies. We further hypothesized that R-loops generated due to elevated TOP1(S320A)ccs should spatially coincide with TOP1^S320A^ entrapment. To test this, we performed DNA/RNA hybrid immunoprecipitation (DRIP) with U2-OS cells ectopically expressing EGFP-TOP1^WT^ and EGFP-TOP1^S320A^, and performed quantitative real-time PCR with the above probes. In agreement with our reasoning, there was significantly higher R-loop enrichment in EGFP-TOP1^S320A^ samples. Further, with the only exception of geminin, R-loops were spatially colocalized with TOP1ccs in gene bodies (Fig. 9D). Collectively, these findings elucidate the genome-wide transcriptional effects of CHK1 inhibition and establish a transcriptional context for TOP1cc stabilization and R-loop accumulation in response to this inhibition. Together, these findings reveal that CHK1 inhibition induces gene-length-dependent transcriptional repression, driven by impaired TOP1 dynamics and accumulation of R-loops at gene bodies.

## Discussion

Maintenance of genomic integrity is of central importance to cellular survival. TOP1, a highly conserved enzyme, plays essential roles in DNA metabolism by relieving topological stress during replication and transcription (Pommier et al, 2016a). The most vulnerable intermediate of its catalytic cycle is the TOP1-DNA covalent complex (TOP1cc), which, while necessary for catalysis, can become a potent source of DNA damage and genomic instability if not efficiently resolved. Consequently, stabilization of TOP1ccs has long served both as a therapeutic strategy in cancer (*via* TOP1 poisons) and as an elegant model system to dissect cellular responses to DNA-protein adducts. Upon exposure to TOP1 poisons, TOP1ccs are targeted by a range of PTMs, including ubiquitylation, SUMOylation, or PARylation (Sun et al, 2020a; Lin et al, 2008; Sun et al, 2021). While TOP1 poisoning has served as an elegant model for studying how cells respond to TOP1ccs, it is nonetheless a valuable endeavor to understand mechanisms that regulate TOP1cc levels under unperturbed cellular metabolism. For example, recent studies have established the TEX264-p97-SPRTN pathway as a vital surveillance mechanism preventing TOP1cc accumulation in unperturbed cells (Fielden et al, 2020). An incisive investigation into cellular mechanisms that regulate steady-state TOP1cc abundance is expected to yield valuable insights into this vital aspect of genomic stability.

Employing a focused panel of inhibitors targeting DNA damage response-associated kinases in an inhibitor screen, we uncovered an unanticipated pathway: CHK1 directly regulates the TOP1 catalytic cycle *via* phosphorylation of serine 320 (S320). This phosphorylation event minimizes genome-wide accumulation of TOP1ccs and thereby safeguards genomic stability. Pharmacological inhibition of CHK1 led to robust accumulation of TOP1ccs. We also established that while ATR kinase is an integral part of CHK1 signaling, its role is limited to maintenance of basal cellular CHK1 activity with respect to the regulation of TOP1cc levels. Strikingly, unlike in the case of TOP1 poisons, these CHK1*i*-induced TOP1ccs were refractory to removal by canonical repair pathways, including proteasomal degradation, TDP1, and autophagy. CHK1*i*-stabilized TOP1ccs were also found to escape recognition by the nuclease machinery as well, with the exception of CtIP, which was found to be capable of limited action on these structures. This observation was in line with the results of the DUST assays, which revealed that CHK1*i*-mediated TOP1cc stabilization fails to induce the canonical post translational modifications which trigger proteasomal degradation of TOP1ccs. Interestingly, SPRTN and p97 were found to be able to target these structures, which is concordant with their ability to target both post-translationally modified and unmodified TOP1ccs (Fielden et al, 2020). Notably, SPRTN has been demonstrated to activate the ATR-CHK1 axis during basal DNA replication *via* cleavage of the CHK1 autoinhibitory domain, leading to its release from replicative chromatin (Halder et al, 2019). This raises the possibility that SPRTN indirectly modulates TOP1cc homeostasis under basal conditions through regulation of constitutive CHK1 activity. However, our results showed that depletion of SPRTN or CtIP or inhibition of p97 does not lead to appreciable accumulation of TOP1cc at steady state (Fig. 3I,J). This suggests that SPRTN protease activity, p97-dependent segregase function, and CtIP-mediated nucleolytic processing are unlikely to act upstream in regulating basal CHK1 activity, but rather function downstream as effector mechanisms facilitating the resolution of TOP1cc lesions under conditions of compromised CHK1 signaling.

Why do CHK1*i*-stabilized TOP1ccs largely escape recognition by the cellular machinery? Biochemical assays revealed a possible basis for this persistence. Our assays revealed that, unlike interfacial inhibitors that lock TOP1 in an irreversible ternary complex, CHK1 inhibition perturbs TOP1 reaction kinetics (discussed below), yielding catalytically engaged but slower-turnover complexes that evade canonical clearance pathways. This unravels a previously uncharacterized mode of TOP1cc stabilization, which largely escapes recognition by the cellular machinery. Eventually, the persistence of these structures inevitably exerts multiple genotoxic effects as evidenced through subsequent experiments. Previously, specific in vivo phosphorylation of TOP1 at serine (S10, S21, S112, S394) and tyrosine (Y268, Y506) residues has been reported (Hackbarth et al, 2008; Yu et al, 2004; Bandyopadhyay et al, 2012). It is noteworthy that these sites were identified from total cellular endogenous/overexpressed TOP1 (soluble mobile fraction), thereby possibly precluding potential identification of phosphorylation(s) on catalytically engaged TOP1, which constitute an acute minority of TOP1 molecules. Hence, we characterized phosphosites exclusively on trapped endogenous TOP1ccs. Mass spectrometry of catalytically engaged TOP1 further revealed a distinct phospho-signature that differs markedly from soluble TOP1. Multiple novel (S320, Y231, and S250) as well as known (S394, T570, and Y480) phosphosites were detected on catalytically engaged TOP1 under unperturbed conditions. In response to lower (200 nM) but not higher (1 μM) concentrations of CPT, four additional phosphosites (Y461, S534, Y538, T706) were detected. Our mass spectrometric data sheds light on a rich and context-specific array of phosphorylation events on TOP1ccs, which warrants deeper investigation. Due to the constraints imposed by the inherent non-quantitative nature of LC-MS/MS workflows, we were not able to determine the responsiveness of these phosphosites to CHK1*i*. Hence, we cross-referenced our MS data with in silico prediction of CHK1 target sites on TOP1, which yielded three overlaps (S320, S394, T570). Among multiple newly identified sites, S320 emerged as the critical CHK1-regulated residue, as confirmed by site-directed mutagenesis and proximity ligation assay. The physiological importance of S320 is highlighted by the evolutionary conservation of this site across species. Kinase-phosphorylation site pairings (KPSPs) show progressive decline in conservation with increasing evolutionary distance (McDonald et al, 2018). However, KPSPs which are associated with dramatic effects of the phosphorylation event on the target protein’s function show striking evolutionary conservation (McDonald et al, 2018). We noticed intriguing conservation of residues between -5 and +3 positions with respect to TOP1 S320, reflecting an evolutionarily conserved KPSP consistent with the high degree of conservation of TOP1 and CHK1 across species. Recent theoretical as well as molecular dynamics (MD) simulation-based calculations have highlighted the critical impact of serine phosphorylation on bending propensities of protein backbones (He et al, 2016; Bickel and Vranken, 2024). Given its strategic positioning at the loop between the α5 and α6 “nose-cone” helices (Carey et al, 2003; Chillemi et al, 2001), phosphorylation of S320 might be implicated in their relative movement, and in turn, their interaction with the DNA substrate. The indispensable role played by the relative dynamics of the “nose-cone” helices has been evidenced by localized non-isomorphism between multiple crystal structures in this region, and theoretically predicted by MD simulations (Sari and Andricioaei, 2005). These studies provide a putative structural basis for our observations. Further, the possibility of other cofactors recognizing phosphorylation on TOP1 Serine 320 to promote its catalytic activity may not be entirely improbable, especially in light of the seminal study from the David Levens group, which demonstrated a critical role played by RNA Pol II in regulating TOP1 catalytic activity (Baranello et al, 2016). However, such a conjecture remains open to future investigation.

Employing a combination of CHK1*i* and a phosphoresistant mutant (TOP1^S320A^) to abrogate CHK1-meditated phosphorylation of TOP1, we gained vital insights into the relevance of this pathway in terms of the biochemistry of TOP1 catalysis. Our results revealed that abrogation of CHK1-mediated TOP1 phosphorylation severely impairs the religation step of the TOP1 catalytic cycle, while preserving the efficiency of the cleavage step. We also observed that while CHK1 critically regulates religation, abrogation of this pathway does not abolish it. Rather, CHK1 inhibition leads to an increase in the lifetime of the religation reaction, thus highlighting a critical difference between interfacial inhibition and CHK1*i*-mediated persistence of TOP1ccs. This also provides a valuable explanation for the escape of these CHK1*i*-stabilized TOP1ccs from the cellular degradation machinery, as alluded to above.

Moreover, the basal level of CHK1 activation is necessary to regulate the TOP1 dynamics for facilitating the removal of topological constraints for smooth progression of replication forks and transcription machinery, and maintaining genomic stability in response to inherently stable TOP1cc for a longer time. Functionally, abrogation of CHK1-mediated TOP1 phosphorylation triggered replication stress, transcriptional impairment, R-loop accumulation, and chromosome instability, underscoring the physiological importance of this regulatory axis. Our observations with DNA damage markers, single-molecule DNA fiber analysis, and metaphase preparations point to widespread implications of this regulatory pathway in the maintenance of genomic integrity. Abrogation of this pathway not only compromises cellular proliferation but also renders cells hypersensitive to TOP1 poisons, suggesting possible exploitation of this vulnerability to reap clinical benefits.

Finally, we were curious to explore if the dysregulation of this TOP1-CHK1 interplay is implicated in cancers. However, our extensive search on The Cancer Genome Atlas (TCGA) database failed to show the prevalence of mutations at the TOP1 S320 position in cancers. In consideration of our data demonstrating the catalytic inefficiency of TOP1^S320A^ and the detrimental consequences associated with this mutation, it can be envisaged that mutations involving TOP1 S320 may not be viable for cellular survival, thus explaining the absence of such mutations in clinical samples. However, CHK1 expression is found to be dysregulated in multiple malignancies compared to corresponding normal tissues. Several studies have shown that high expression of CHK1 is associated with poor prognosis, recurrence, therapy resistance and compromised survival in breast cancer (Al-kaabi et al, 2015), lung cancer (Grabauskiene et al, 2013), leukemia (David et al, 2016) and other cancers (Wang et al, 2013; Zhang et al, 2014; Bao et al, 2006). The relative contribution of dysregulation of the TOP1-CHK1 axis in these phenotypes remains to be explored. Presently, investigational CHK1 and ATR inhibitors are undergoing clinical trials in combination with multiple DNA-damaging agents, including TOP1 inhibitors (Stockton et al, 2024; Slotkin et al, 2022). To date, the rationale behind such approaches has been rooted in checkpoint abrogation by CHK1 inhibitors (Neizer-Ashun and Bhattacharya, 2021). However, our study adds an additional layer of novel mechanistic rationale for combining CHK1 and TOP1 inhibitors. We also revealed that CHK1*i*-mediated sensitization of cancer to various chemotherapeutics cannot be attributed solely to checkpoint abrogation, but also to the accumulation of trapped TOP1ccs on the genome.

**Figure 10:**
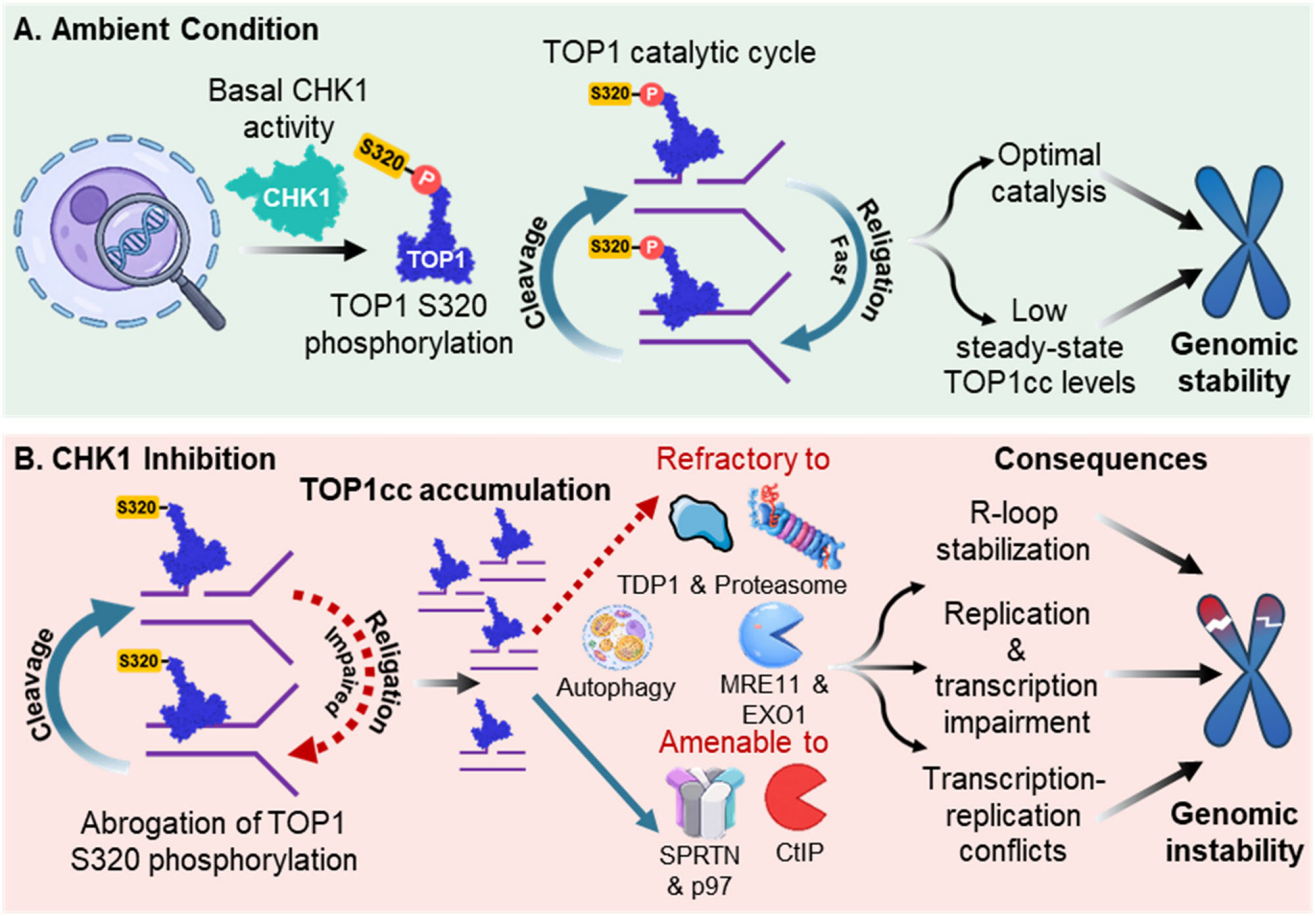
A schematic model of the role played by CHK1 in the regulation of TOP1 catalysis, and its impact on genomic stability. (**A**) Under physiological conditions, CHK1-mediated phosphorylation of TOP1 at Serine 320 ensures efficient catalysis, maintaining minimal TOP1cc levels. (**B**) CHK1 inhibition leads to abrogation of this regulation, resulting in accumulation of TOP1ccs. These TOP1ccs are targeted by SPRTN, p97 and CtIP, while being relatively refractory to processing by the TDP1, proteasomal and autophagic machineries. Abrogation of CHK1-mediated TOP1 phosphorylation at Serine 320 eventually leads to R-loop stabilization, imapired replication and transcription, transcription-replication conflicts and genomic instability.

In summary, our results demonstrate a new pivotal role played by CHK1 in regulating TOP1 dynamics (Fig. 10). This regulatory circuit safeguards genome stability by controlling steady-state TOP1cc levels. Disruption of CHK1-mediated regulation leads to persistent TOP1cc accumulation, replication- and transcription-associated DNA damage, deleterious R-loop formation, and ultimately large-scale genomic instability. Our work unravels a wider jurisdiction of CHK1 as a guardian of genomic stability through direct regulation of TOP1 catalysis. This not only expands the functional repertoire of CHK1 but also refines the mechanistic framework for CHK1 inhibitors in cancer therapy. By revealing that CHK1*i*-driven TOP1cc stabilization contributes to drug sensitization beyond checkpoint abrogation, our findings suggest newer possibilities in combinatorial chemotherapy.

## Methods

**Table:** Reagents and tools table

Reagent/resource	Reference or source	Identifier or catalog number
**Experimental models**		
U2-OS	ECACC	92022711
PANC-1	ECACC	87092802
A549	ECACC	86012804
HCT116	ECACC	91091005
MDA-MB-231	ATCC	HTB-26
WI-38	ATCC	CCL-75
U2-OS^EGFP-TOP1 WT^	This study	N/A
U2-OS^EGFP-TOP1 S320A^	This study	N/A
**Bacterial strains**		
*E. coli* CopyCutter EPI400	Lucigen	LGCC400CH10
DH5α Competent Cells	Thermo Scientific	EC0112
**Recombinant DNA**		
EGFP-TOP1^WT^ plasmid	Prof. Benubrata Das, IACS, Kolkata, India	N/A
mCherry-NPM1 plasmid	Prof. Jing Yi, Shanghai Jiao Tong University School of Medicine, Shanghai, China.	N/A
EGFP-TOP1^S320A^ plasmid	This study	N/A
EGFP-TOP1^S394A^ plasmid	This study	N/A
EGFP-TOP1^T570A^ plasmid	This study	N/A
**Antibodies**		
Anti-TOP1 antibody produced in rabbit	Sigma-Aldrich	HPA019039; RRID: AB_1858187
Anti-DNA-RNA Hybrid Antibody, clone S9.6	Millipore	MABE1095; RRID:AB_2861387
Anti-Topoisomerase I-DNA Covalent Complexes Antibody	Millipore	MABE1084; RRID:AB_2756354
Anti-GFP, N-terminal antibody produced in rabbit	Sigma-Aldrich	G1544; RRID:AB_439690
Mouse Anti-DNA, double-stranded (dsDNA) Monoclonal antibody, Unconjugated	Millipore	MAB030; RRID:AB_93965
Anti-phospho-Histone H2A.X (Ser139) Antibody	Millipore	05-636-I; RRID:AB_2755003
Anti-53BP1, clone BP13	Millipore	MAB3802; RRID:AB_11212586
Anti-Ubiquitin Mouse mAb (FK2)	Millipore	ST1200; RRID:AB_2043482
Anti-Poly ADP-ribose Antibody, clone 10H	Millipore	MABC547; RRID:AB_2861394
Rabbit anti-Phospho KAP-1 (S824) Antibody	Sigma-Aldrich	PLA0140; RRID:AB_3697425
Anti-C1orf124 antibody produced in rabbit	Sigma-Aldrich	HPA025073; RRID:AB_1847695
Anti-TDP1 antibody produced in rabbit	Sigma-Aldrich	SAB1411073
Chk1 Antibody (G-4)	Santa Cruz Biotechnology	sc-8408; RRID:AB_627257
Exo1 Antibody (266)	Santa Cruz Biotechnology	sc-56092; AB_783300
CtIP Antibody (D-4)	Santa Cruz Biotechnology	sc-271339; RRID:AB_10608728
SUMO-1 Antibody	Cell Signaling Technology	4930; RRID:AB_10698887
SUMO-2/3 (18H8) Rabbit mAb	Cell Signaling Technology	4971; RRID:AB_2198425
Mre11 Antibody	Cell Signaling Technology	4895; RRID:AB_2145100
Anti-mouse IgG, HRP-linked Antibody	Cell Signaling Technology	7076; RRID:AB_330924
Anti-rabbit IgG, HRP-linked Antibody	Cell Signaling Technology	7074; RRID:AB_2099233
Rabbit anti-Phospho RPA32 (S33) Antibody	Bethyl Laboratories	A300-246A; RRID:AB_2180847
Rabbit anti-RPA32 Antibody	Bethyl Laboratories	A300-244A; RRID:AB_185548
Phosphoserine antibody	Abcam	ab9332, RRID:AB_307184
Biotin antibody	Abcam	53494; RRID:AB_867860
BrdU (CldU)	Abcam	ab6326; RRID:AB_305426
BrdU (IdU) antibody	BD Biosciences	347580; AB_10015219
Cy3-AffiniPure Donkey Anti-Rat IgG (H + L)	Jackson ImmunoResearch Labs	712-165-153; RRID:AB_2340667
Alexa Fluor 488-AffiniPure F(ab’)2 Fragment Donkey Anti-Mouse IgG (H + L)	Jackson ImmunoResearch Labs	715-546-151; RRID:AB_2340850
Alexa Fluor 594 AffiniPure Goat Anti-Mouse IgG (H + L)	Jackson ImmunoResearch Labs	115-585-003; RRID:AB_2338871
Alexa Fluor 488 AffiniPure Goat Anti-Rabbit IgG (H + L)	Jackson ImmunoResearch Labs	111-545-003; RRID:AB_2338046
Alexa Fluor® 594 AffiniPure® Goat Anti-Rabbit IgG (H + L)	Jackson ImmunoResearch Labs	111-585-003; RRID:AB_2338059
**Oligonucleotides and other sequence-based reagents**		
Control siRNA	Horizon Discovery	D-001810-01-50
siMRE11	Horizon Discovery	L-009271-00-0050
siEXO1	Horizon Discovery	L-013120-00-0050
siCtIP	Horizon Discovery	L-011376-00-0050
siTDP1	Horizon Discovery	L-016112-00-0050
siATG7	Horizon Discovery	L-020112-00-0050
siSPRTN	Horizon Discovery	L-015442-02-0020
siTOP1 (3’UTR) 5’-CUUAGAUUCACUCGCCCUUCA-3’	This study	N/A
TOP S320 peptide FKAQTEARKQMSKEEKLKIKEEN	This study	N/A
TOP S320A mutant peptide FKAQTEARKQMAKEEKLKIKEEN	This study	N/A
S320A Forward Primer 5’-GAAACAGATGGCCAAGGAAGAGA -3’	This study	N/A
S320A Reverse Primer 5’-GAAACAGATGGCCAAGGAAGAGA -3’	This study	N/A
S394A Forward Primer 5’-CAAGGTTCCTGCTCC TCCTCC-3’	This study	N/A
S394A Reverse Primer 5’-GGAGGAGGAGCAGGAACCTTG -3’	This study	N/A
T570A Forward Primer 5’-TAGACTCAATGCTGGTATTCTG -3’	This study	N/A
T570A Reverse Primer 5’-CAGAATACCAGCATTGAGTCTA-3’	This study	N/A
MYO3A TSS Forward Primer 5’-GTCAGATCCGGAGGACC-3’	PMID: 31533039	N/A
MYO3A TSS Reverse Primer 5’--3’	PMID: 31533039	N/A
MYO3A Gene Body Forward Primer 5’-CTACAGCAGCCCACTCAAG-3’	PMID: 31533039	N/A
MYO3A Gene Body Reverse Primer 5’-CATTATCCAGTTTCTTGATTCATG -3’	PMID: 31533039	N/A
β-Actin TSS Forward Primer 5’-CGGGGTCTTTGTCTGAGC-3’	PMID: 31533039	N/A
β-Actin TSS Reverse Primer 5’-CAGTTAGCGCCCAAAGGAC-3’	PMID: 31533039	N/A
β-Actin Gene Body Forward Primer 5’-GGAGCTGTCACATCCAGGGTC -3’	PMID: 31533039	N/A
β-Actin Gene Body Reverse Primer 5’-TGCTGATCCACATCTGCTGG-3’	PMID: 31533039	N/A
g-Actin TSS Forward Primer 5’-CCGCAGTGCAGACTTCCGAG -3’	PMID: 31533039	N/A
g-Actin TSS Reverse Primer 5’-CGGGCGCGTCTGTAACACGG -3’	PMID: 31533039	N/A
g-Actin Gene Body Forward Primer 5’-GTGACACAGCATCACTAAGG-3’	PMID: 31533039	N/A
g-Actin Gene Body Reverse Primer 5’-ACAGCACCGTGTTGGCGT-3’	PMID: 31533039	N/A
PTB Gene Body Forward Primer 5’-GCCGTTGGTACAAAGGTAGG -3’	PMID: 31533039	N/A
PTB Gene Body Reverse Primer 5’-GCCCCTTAGGAATGGAAAAG -3’	PMID: 31533039	N/A
Geminin 7 Gene Body Forward Primer 5’-TCTTCTTCCACCTGGACCAC-3’	PMID: 31533039	N/A
Geminin 7 Gene Body Reverse Primer 5’-GGGACAGAGAGAGTGCCTTG -3’	PMID: 31533039	N/A
IL4 Gene Body Forward Primer 5’-TTCAGGTGACAAGTGCCACAG -3’	PMID: 31533039	N/A
IL4 Gene Body Reverse Primer 5’-CTGGTTGGCTTCCTTCACAG-3’	PMID: 31533039	N/A
Intergenic Forward Primer 5’-ACCCAGCACCCCCTAATACC-3’	PMID: 31533039	N/A
Intergenic Reverse Primer 5’-AGCCGGACATGCTTCCAGAG -3’	PMID: 31533039	N/A
**Chemicals, enzymes, and other reagents**		
Click-iT EdU Cell Proliferation Kit for Imaging, Alexa Fluor 488 dye	ThermoFisher Scientific	C10337
Click-iT EdU Cell Proliferation Kit for Imaging, Alexa Fluor 594 dye	ThermoFisher Scientific	C10339
Click-iT RNA Alexa Fluor 488 Imaging Kit	ThermoFisher Scientific	C10329
Click-iT RNA Alexa Fluor 594 Imaging Kit	ThermoFisher Scientific	C10330
Duolink In Situ PLA Probe Anti-Rabbit PLUS	Sigma-Aldrich	DUO92002
Duolink In Situ PLA Probe Anti-Rabbit Minus	Sigma-Aldrich	DUO92004
Duolink In Situ Detection Reagents Red	Sigma-Aldrich	DUO92008
ChromoTek GFP-Trap Magnetic Agarose	Proteintech Group	gtma; RRID: AB_2631358
SsoAdvanced Universal SYBR Green Supermix	Bio-Rad	1725271
Human Topoisomerase I Enzyme	TopoGEN	TG2005HRC
Recombinant human Chk1 protein	Abcam	ab69918
Phusion High-Fidelity DNA Polymerase	ThermoFisher Scientific	F530S
γ^32^P-ATP	Board of Radiation & Isotope Technology, India	
Adenosine 5′-triphosphate disodium salt trihydrate	Sigma-Aldrich	ATPD-RO;
DharmaFECT1 transfection reagent	Horizon Discovery	T-2001-03
Lipofectamine 2000 Transfection Reagent	ThermoFisher Scientific	11668019
Protein A Magnetic Beads	ThermoFisher Scientific	88845
Protein G Magnetic Beads	ThermoFisher Scientific	88847
PV1019	MedChemExpress	HY-125203
SCH900776	Selleckchem	S2735
AZD6738	Cayman Chemical	21035
Volasertib (BI6727)	Cayman Chemical	18193
U0126	Cayman Chemical	70970
Danusertib	Cayman Chemical	18387
KN-93	Cayman Chemical	13319
LJH685	Cayman Chemical	19913
GSK2606414	Cayman Chemical	17376
MRT68921	Cayman Chemical	19905
ARQ-092	Cayman Chemical	21388
Go6983	Cayman Chemical	13311
CX-4945	Cayman Chemical	16779
Brigatinib (AP26113)	Cayman Chemical	19778
MK1775	Cayman Chemical	MK-1775
KU-55933	Cayman Chemical	16336
Wortmannin	Sigma-Aldrich	W1628
RO-3306	Sigma-Aldrich	SML0569
SP600125	Sigma-Aldrich	S5567
NU7026	Sigma-Aldrich	N1537
PHA-767491	Sigma-Aldrich	PZ0178
SB203580	Sigma-Aldrich	559389
Dorsomorphin (Compound C)	Sigma-Aldrich	171260
Torin1	Sigma-Aldrich	475991
Roscovitine (Seliciclib)	Sigma-Aldrich	557364
PDD00017273	Sigma-Aldrich	SML1781
CB-5083	MedChemExpress	HY-12861
DNAzol Reagent	ThermoFisher Scientific	10503027
DNaseI, RNase free	Roche	10104159001
Ribonuclease H	Roche	10786357001
Benzonase Nuclease	Millipore	E1014
Tris-HCl	Sigma-Aldrich	9310-OP
NaCl	Sigma-Aldrich	S9888
NP-40	Sigma-Aldrich	492018
Triton X-100	Sigma-Aldrich	X100
cOmplete Mini, EDTA-free (protease inhibitor cocktail)	Roche	11836170001
PhosSTOP (phosphatae inhibitor cocktail)	Roche	PHOSS-RO
Glycine	Sigma-Aldrich	G7126
Bovine Serum Albumin	Himedia	MB083
Paraformaldehyde	Sigma-Aldrich	158127
EDTA	Sigma-Aldrich	E4884
DMSO	Sigma-Aldrich	472301
Proteinase K	Sigma-Aldrich	P8044
Hoechst 33258	Sigma-Aldrich	94403
Hoechst 33242	Sigma-Aldrich	AMBH9A260690
Aphidicolin	Sigma-Aldrich	178273
Glycerol	Sigma-Aldrich	G5516
**Software**		
ImageJ	ImageJ	https://imagej.nih.gov/ij/, RRID:SCR_003070
GraphPad Prism	GraphPad Software, Inc	https://www.graphpad.com:443/, RRID: SCR_002798
LAS X	Leica Microsystems	https://www.leica-microsystems.com/products/microscope-software/details/product/leica-las-x-ls/. RRID: SCR_013673
ZEISS ZEN Microscopy Software	Carl Zeiss AG	https://www.zeiss.com/microscopy/en/products/software/zeiss-zen.html. RRID: SCR_013672
Google CoLab	Google LLC	https://colab.research.google.com/notebooks/basic_features_overview.ipynb. RRID: SCR_018009

### Cell culture

U2-OS, PANC-1, A549, and HCT116 cells were procured from ECACC. MDA-MB-231 and WI-38 cells were procured from ATCC. Cells were cultured in Dulbecco’s Modified Eagle Medium supplemented with 10% fetal bovine serum and Penicillin–Streptomycin–Amphotericin B mix. Cells were maintained at 37 °C under 95% relative humidity and 5% CO_2_.

### Development of stable cell lines

U2-OS^EGFP-TOP1WT^ and U2-OS^EGFP-TOP1S320A^ were generated from U2-OS cells. Cells were seeded in six-well plates, followed by lipofection with 500 ng plasmid expressing EGFP-TOP1^WT^ or EGFP-TOP1^S320A^. Cells were sub-cultured 48 h post transfection, followed by selection with 2.4 mg/mL G418. Surviving cells were intermittently expanded in alternate cycles of culturing in the presence or absence of G418 (72 h each) till they reached confluence. Expression was monitored microscopically by visualizing EGFP fluorescence.

### RADAR assay

Rapid Approach to DNA Adduct Recovery (RADAR) assay (Kiianitsa and Maizels, 2013) was performed to quantify cellular levels of TOP1ccs. Briefly, 1.5 × 10^5^ cells were seeded per well in six-well plates (to achieve ~90% confluence), followed by relevant treatments. Cells were lysed with DNAzol reagent. DNA was precipitated with one volume of absolute ethanol, recovered by spooling, and washed with 80% ethanol. Pellets were briefly air-dried, followed by resuspension in 8 mM NaOH. DNA was diluted in Tris-Buffered Saline. DNA (500 ng per sample) was spotted on a nitrocellulose membrane using a slot blot apparatus (Bio-Rad, USA) under vacuum. The membrane was air-dried, sandwiched in Whatman Paper, and baked at 80 °C for 2 h in a hot air oven. Membranes were processed then according to western blotting protocol, and probed using anti-TOP1 and anti-dsDNA antibodies.

### RADAR screening of kinase inhibitors and principal component analysis

A library of 25 small-molecule kinase inhibitors was employed to evaluate their ability to induce TOP1ccs. Briefly, 7.5 × 10^4^ cells were seeded per well in 12-well plates. Cells were treated with relevant concentrations (C1, C2, C3; Appendix Table S1) of inhibitors for 2, 4, or 6 h, followed by sample processing as per the RADAR assay protocol. Densitometric analysis of blots was used for comparative evaluation of TOP1cc stabilization. All signals were normalized to the corresponding DNA control. Further, in order to nullify possible blot-to-blot variations, fold changes in TOP1cc levels were calculated based on an untreated sample of the corresponding blot. All fold change values (9 per inhibitor) were used to perform a principal component analysis on GraphPad Prism to identify significant hits. Values were subjected to standardization, and component selection was performed based on Kaiser’s rule (eigenvalue cut-off of 1). The two top-ranked PCs (based on variance coverage) were used to generate the PCA plot.

### Fluorescence recovery after photobleaching (FRAP)

FRAP-mediated quantification of TOP1 dynamics was performed as described elsewhere (Gupta et al, 2022). U2-OS^EGFP-TOP1WT^ cells were seeded on optically clear thin-bottomed confocal grade 35-mm dishes, followed by relevant treatments 16 h post seeding. FRAP was performed on a Zeiss LSM 780 confocal microscope. Images acquisition was performed using ×40 objective with ×6 optical zoom, resulting in a pixel dwell time of 1 µs. Circular bleach and reference regions of interest (ROIs) were defined with identical diameters. Five pre-bleach images were acquired using 488 nm laser line at 1% power, followed by exposure of the bleach ROI to 20 iterations of 488 nm laser at 100% power, resulting in bleaching of fluorophores. Post bleaching, 250 images were acquired at an interval of 1 ms. The percent mobile and immobile fraction was calculated using Zen 2.3 SP1 FP1 software package. For percentage fluorescence recovery, the following formula was employed:

$$\% {Recovery}\,{at}\,{time}\,t=\frac{\left\{\frac{{MFI}\,{of}\,{bleach}\,{ROI}}{{MFI}\,{of}\,{reference}\,{ROI}}\right\}{at}\,{time}\,t}{\left\{\frac{{MFI}\,{of}\,{bleach}\,{ROI}}{{MFI}\,{of}\,{reference}\,{ROI}}\right\}{at}\,{time}\,0}X100$$

Where MFI represents mean fluorescence intensity. For statistical analysis, nine cells were scored per sample.

### Immunoblotting

Western blotting was performed as reported previously (Guha Majumdar and Subramanian, 2019).

### Assay for TOP1 downregulation (alkaline lysis method)

CPT or SCH-induced TOP1 degradation was assayed using alkaline cell lysates as reported previously (Guha Majumdar et al, 2023) (Appendix Fig. S6F). Briefly, 1.5 × 10^5^ cells were seeded in six-well plates, and subjected to relevant treatments 16 h post seeding. Post treatment, cells were washed once with PBS, followed by alkaline lysis (200 mM NaOH and 2 mM EDTA). Lysates were then collected and neutralized using 1 volume of Tris pH 8.0 (1 M). Appropriate volumes of 10× DNase I digestion buffer and 50× protease inhibitor cocktail (Roche) were added to achieve 1× final concentration of each. DNA was digested on ice for 1 h using a cocktail of DNAse I and Benzonase in order to release trapped TOP1ccs. Samples were then boiled in SDS sample buffer for 20 min and subjected to western blotting using anti-TOP1 antibody.

### DUST assay

Detection of Ubiquitylated and SUMOylated TOP1ccs (DUST) assay was performed as described elsewhere (Sun et al, 2020a). Briefly, 1.6 × 10^6^ cells were seeded in 90 mm dishes. Lysis and DNA recovery were performed as per the RADAR assay protocol. DNA pellets were briefly air-dried, resuspended in Tris-EDTA (TE) buffer (pH 8.0), and heated at 65 °C for 15 min. DNA was then sheared on ice using a Cole-Parmer Ultrasonic Processor (40% amplitude; 4 cycles; 10 s on/10 s off). Samples were centrifuged at 12,500 rpm for 5 min, followed by RNase A treatment of the supernatant for 1 h at 4 °C. Samples were clarified, and DNA was recovered as already reported (Sun et al, 2020a). 25 µg DNA per sample was digested for 2 h at 37 °C using a mix of 10 units of DNase I and 10 units Benzonase. Digested samples were resolved through SDS-PAGE on 6% polyacrylamide gels. SUMOylation, PARylation, and Ubiquitination were detected using specific antibodies. In addition, 2 µg DNA per sample was subjected to dot blotting to ensure equal sample loading.

### EdU and EU incorporation assays

Click-EdU and Click-EU assays were employed for determining levels of cellular replication and transcription, respectively. Cells were seeded on coverslips, followed by appropriate treatments. Cells were incubated with 10 µM EdU or 1 mM EU (for 30 min or 1 h, respectively) prior to termination of the experiment. Cells were then washed with PBS and fixed with 4% paraformaldehyde for 15 min at 4 °C. Fixed cells were processed according to the manufacturer’s instructions (Click-IT kit, Invitrogen, USA). Cells were then observed under a Zeiss LSM 780 confocal microscope.

### Immunofluorescence microscopy and live-cell imaging

Immunofluorescence assays were performed as described previously (Guha Majumdar et al, 2023). Briefly, cells were seeded on coverslips, followed by requisite treatments 16 h post seeding. Cells were washed once with PBS and fixed with 4% paraformaldehyde for 15 min at 4 °C (for RPA32 and phospho-RPA32 immunofluorescence, cells were pre-extracted with 0.5% Triton X-100 in PBS for 7 min on ice prior to fixation). Cells were permeabilized with PBS supplemented with 0.25% v/v Triton X-100. Cells were washed once with PBS and blocked for 1 h at room temperature in 1% BSA in PBS. Coverslips were incubated with relevant primary antibodies (diluted in 2.5% BSA in PBS) overnight at 4 °C, followed by three washes (5 min each) with PBS. Cells were then incubated with Alexa Fluor 488 or Alexa Fluor 594-tagged secondary antibodies at room temperature for 2 h under subdued light and washed thrice with PBS (5 min each). Coverslips were then air-dried and mounted on glass slides using 80% glycerol supplemented with 10 µg/mL Hoechst 33258. In case of experiments involving simultaneous immunofluorescence and EdU incorporation, the Click EdU reaction was performed at the end of secondary antibody incubation.

Live-cell imaging was performed on a Leica MICA High-Throughput Imaging and Analysis system. Nuclei were labeled with 5 µg/mL Hoechst 33242 15 min prior to imaging. For EGFP-TOP and mCherry-NPM1 colocalization experiments, enrichment ratio was calculated as follows:

$${{Enrichment}}\,{{ratio}}=\frac{({{{\rm{EGFP}}}}\,{{{\rm{intensity}}}}\,{{{\rm{in}}}}\,{{{\rm{nucleolus}}}})}{({{{\rm{EGFP}}}}\,{{{\rm{intensity}}}}\,{{{\rm{in}}}}\,{{{\rm{nucleoplasm}}}})}$$

The enrichment ratio is interpreted as follows: 1 = Pan-nuclear localization; >1 = Selective enrichment in nucleolus; <1 = Selective enrichment in nucleoplasm.

In experiments involving image acquisition in both green and red channels in cells transfected with EGFP-TOP1, cells were subjected to prior permeabilization with extraction buffer (0.5% Triton X-100 in PBS) on ice for 7 min, prior to fixation, in order to minimize background signal originating from EGFP.

All microscopic image analyses were performed using ImageJ software.

### TOP1cc immunofluorescence

Immunofluorescence-based detection of TOP1ccs was performed as described previously (Guha Majumdar et al, 2023). Briefly, 1.5 × 10^5^ cells were seeded on coverslips in six-well plates, followed by relevant treatments. Coverslips were washed and fixed with 4% paraformaldehyde for 15 min at 4 °C. Cells were then permeabilized with PBS-0.25% Triton X-100 for 15 min at 4 °C. Permeabilization was followed by denaturation with 2% SDS for 10 min at 20 °C. Cells were washed thrice with PBS (5 min each), followed by blocking with PBS supplemented with 0.05% Tween 20, 0.01% Triton X-100, and 1% BSA (PBSTT) at room temperature for 2 h. Downstream processing was identical to that of immunofluorescence.

### Proximity ligation assay (PLA)

Physical interaction between proteins in cells was probed using Proximity Ligation Assay employing Duolink Proximity Ligation Assay kit (Sigma, St Louis, MO, USA). Briefly, 5 × 10^4^ cells per well were seeded on circular coverslips in 12-well plates, followed by requisite treatments. Samples were fixed with 4% paraformaldehyde and permeabilized with PBS supplemented with 0.25% v/v Triton X-100 at room temperature for 10 min. Samples were blocked with Duolink blocking reagent at 37 °C for 1 h in a humidified chamber. Further processing steps, including incubation with primary and secondary antibodies, ligation, and amplification, were executed as per the manufacturer’s instructions. Cells were imaged under a Zeiss LSM 780 confocal microscope.

### Cell counting experiments for comparative evaluation of cell growth kinetics

In order to compare the growth kinetics of U2-OS^EGFP-TOP1WT^ and U2-OS^EGFP-TOP1S320A^ stable cells, 4000 cells were seeded in 60-mm dishes. Cells were recovered through trypsinization 3, 6-, and 9-days post seeding, and cell number was enumerated using a hemocytometer.

### RNA/DNA hybrid immunofluorescence

RNA-DNA hybrids were visualized as described elsewhere (Marabitti et al, 2019) with minor modifications. Briefly, exponentially growing cells were seeded on coverslips. Cells were fixed with 4% paraformaldehyde (in PBS) at room temperature for 10 min and permeabilized with 0.5% ice-cold Triton X-100. Cells were then subjected to RNase A pre-treatment in RNase A digestion cocktail (6 µg/mL RNase A in 10 mM Tris-HCl pH 7.5 and 500 mM NaCl) for 45 min at 37 °C. For RNase H control, cells were subjected to co-digestion with RNase A and RNase H. Post digestion, coverslips were processed as per the immunofluorescence protocol.

### Detection of RNA/DNA hybrids through dot blot

For dot blot-mediated detection of RNA-DNA hybrids, cells were lysed with DNAzol reagent, followed by precipitation on genomic DNA with 1 volume of 100% ethanol. Precipitated genomic DNA was washed once with 70% ethanol, followed by resuspension in 1× NEB CutSmart buffer. Genomic DNA was randomly fragmented overnight with a cocktail of restriction enzymes consisting of EcoRI, HindIII, XbaI, and NotI. Fragmented DNA was subjected to phenol–chloroform extraction and precipitated overnight with absolute ethanol. The DNA pellet was washed once with 70% ethanol, and resuspended in TE buffer. In total, 1 µg of DNA was loaded per sample. Further processing was similar to RADAR assay. For determining RNase H sensitivity of signal, 1 µg of DNA sample was digested with RNase H for 6 h prior to loading.

### Plasmids, siRNAs, and transfections

EGFP-TOP1^WT^ plasmid was a kind gift from Professor Benu Brata Das, Indian Association for the Cultivation of Science, Kolkata, India. mCherry-NPM1 plasmid was a kind gift from Professor Jing Yi, Shanghai Key Laboratory for Tumor Microenvironment and Inflammation, Key Laboratory of Cell Differentiation and Apoptosis of the Chinese Ministry of Education, Department of Biochemistry and Molecular Cell Biology, Shanghai Jiao Tong University School of Medicine, Shanghai, China. Transfections were carried out using Lipofectamine 2000 reagent (Invitrogen, USA) according to the manufacturer’s protocol. For immunoprecipitation experiments, transfections were performed using the calcium phosphate method. Control siRNA and siRNAs against MRE11, EXO1, CtIP, TDP1, ATG7 and SPRTN (Catalog IDs D-001810-01-50, L-009271-00-0050, L-013120-00-0050, L-011376-00-0050, L-016112-00-0050, L-020112-00-0050 and L-015442-02-0020, respectively) were procured from Horizon Discovery, USA. siRNA targeting 3’UTR of human TOP1 was custom synthesized (5’-CUUAGAUUCACUCGCCCUUCA-3’) with contract research from IDT Technologies (Coralville, IA, USA). siRNA transfections were performed using Dharmafect siRNA transfection reagent.

### Site-directed mutagenesis

EGFP-TOP1 expressing plasmid was subjected to polymerase chain reaction (PCR)-based site-directed mutagenesis. PCR was performed with 20 ng plasmid DNA and corresponding forward and reverse primers, using Phusion DNA polymerase as per the manufacturer’s protocol. The PCR reaction was digested with DpnI enzyme at 37 °C for 2 h, followed by transformation in chemically competent *E. coli* CopyCutter EPI400 cells. Plasmids from resultant colonies were subjected to sequencing. Mutagenesis was performed with the following primers: S320A forward 5’-GAAACAGATGGCCAAGGAAGAGA-3’; S320A reverse 5’-GAAACAGATGGCCAAGGAAGAGA-3’; S394A forward 5’-CAAGGTTCCTGCTCC TCCTCC-3’; S394A reverse 5’-GGAGGAGGAGCAGGAACCTTG-3’; T570A forward 5’-TAGACTCAATGCTGGTATTCTG-3’; T570A reverse 5’-CAGAATACCAGCATTGAGTC TA-3’.

### Mass spectrometric analysis of TOP1 phosphorylation sites

A tandem mass spectrometry-based assay was employed to detect amino acids phosphorylated on catalytically active TOP1. Briefly, exponentially growing cells were seeded in 90 mm dishes, followed by requisite treatments. To specifically enrich catalytically active TOP1 cells were processed as per the DUST assay protocol. Protease and phosphatase inhibitors were included in all steps post lysis. Samples were resolved on SDS-PAGE, followed by gel excision in the 100-125 KDa region. Further sample processing and analysis were performed through contract research (Valerian Chem Private Limited, India). Briefly, samples were reduced with 5 mM tris(2-carboxyethyl)phosphine (TCEP) followed by alkylation with 50 mM iodoacetamide. Samples were digested with Trypsin (1:50, Trypsin/lysate ratio) for 16 h at 37 °C. Digests were cleaned using a C18 silica cartridge and vacuum dried. Dried pellets were resuspended in buffer A (5% acetonitrile, 0.1% formic acid). All analyses were performed on an Ultimate 3000 RSLCnano system coupled with an Orbitrap Eclipse. Approximately 500 ng of each sample was loaded on a C18 column (50 cm, 3.0-μm Easy-spray column, Thermo Fisher Scientific). Peptides were eluted with a 0–40% gradient of buffer B (80% acetonitrile, 0.1% formic acid) at a flow rate of 300 nL/min and injected for MS analysis. LC gradients were run for 100 min. MS1 spectra were acquired in the Orbitrap (R = 240k; AGQ target = 400,000; Max IT = 50 ms; RF Lens = 30%; mass range = 400–2000; centroid data). Dynamic exclusion was employed for 10 s, excluding all charge states for a given precursor. MS2 spectra were collected in a linear ion trap (rate = turbo; AGQ target = 20,000; Max IT = 50 ms; NCE_HCD_ = 35%).

Data was analyzed with Proteome Discoverer (v2.2) against the single reference proteome database. For Sequest search, the precursor and fragment mass tolerances were set at 10 ppm and 0.8 Da, respectively. Enzyme specificity was set for trypsin/P (cleavage at the C terminus of “K/R: unless followed by “P”) along with maximum missed cleavages value of two. Carbamidomethyl on cysteine as a fixed modification, and oxidation of methionine and N-terminal acetylation and Phosphorylation (S,T,Y) were considered as variable modifications for database search. Both peptide spectrum match and protein false discovery rate were set to 0.01 FDR.

### Isolation of mouse splenocytes

Splenic cells were isolated from BALB/c mice. Briefly, 4–6 weeks old male mice (Source: BARC Animal House) were euthanized, and the spleen was harvested under aseptic conditions. The spleen was sliced into small pieces and thoroughly washed thrice with ice-cold PBS. A single cell suspension was created by passing the excised spleen pieces through a cell strainer with the help of the plunger of a sterile 1 mL syringe. The cell suspension was centrifuged at 1600 rpm for 5 min, and the pellet was resuspended in 2 mL of pre-warmed RBC lysis buffer (193 mM NH_4_Cl, 12.5 mM KHCO_3_, 0.01% w/v EDTA). The cell suspension was incubated at 37 °C for 2 min. Post incubation, 30 mL of PBS was added and the cells were centrifuged at 1600 rpm for 5 min. The cell pellet was resuspended in an appropriate volume of PBS. Usage of animals was approved by the BARC animal ethics committee vide order no.: BAEC/01/2023, and animals were treated as per the guidelines of the animal ethics committee.

### RNA sequencing and data analysis

RNA sequencing was performed through contract research. Briefly, cells were seeded in 60-mm dishes and subjected to requisite treatments 16 h post seeding. Cells were then washed twice with ice-cold PBS, and harvested through trypsinization. Total RNA was isolated using Qiagen RNeasy Mini Kit according to the manufacturer’s instructions. Paired-end sequencing (average read length ~150 base pairs) was performed through Illumina Sequencing by Synthesis, and a total of ~50 million reads were analyzed per sample. Differential gene expression analysis (with respect to the untreated sample) was performed through contract research, and downstream data analysis was performed as follows. Downregulated genes were selected with a fold change cutoff of 0.5. Unaltered genes were defined as genes with a fold change lying between 0.9 and 1.1. Gene lengths were retrieved from NCBI through the deployment of a Python code run on the Google Colaboratory environment.

### In vitro kinase assays

In vitro kinase assay was performed with purified, catalytically active human CHK1 and TOP1 proteins in kinase reaction buffer (HEPES pH 7.5, 1 mM DTT, 100 µM Na_3_VO_4_, 10 mM MgCl_2_). Reactions were initiated by adding 50 µM ATP (spiked with 10 µCi ^32^P-labeled ATP) to CHK1 and TOP1 (2 picomoles each). Samples were incubated at 30 °C for 30 min. Reactions were terminated by adding SDS sample buffer. Samples were boiled at 95 °C for 10 min, followed by resolution on SDS-PAGE. The gel was dried and subjected to autoradiography.

For in vitro kinase assays with synthetic peptides (Fig. EV2E), TOP1 S320 (FKAQTEARKQM**S**KEEKLKIKEEN) and TOP1 S320A (FKAQTEARKQM**A**KEEKLKIKEEN) were synthesized with N-terminal biotinylation through contract research from Synbio Technologies (NJ, USA). Reactions were performed with recombinant human CHK1 and the relevant peptides (2 picomoles each), and reactions were performed in kinase reaction buffer with 50 µM ATP. Reactions performed as described above, and terminated by addition of SDS and EDTA (final concentrations 0.5% v/v and 1 mM, respectively). Reactions were diluted to 500 µL with kinase assay buffer (without ATP). Streptavidin agarose magnetic beads (25 µL per condition) were equilibrated twice with kinase assay buffer (without ATP), followed by incubation with the above samples for 4 h at room temperature. Beads were then washed thrice in Tris Buffered Saline (TBS), and resuspended in 100 µL TBS. Samples were boiled at 95 °C for 10 min, followed by dot blotting with anti-phosphoserine and anti-biotin antibodies.

### In vitro plasmid relaxation assay

In vitro plasmid relaxation assay (Guha Majumdar et al, 2023) was performed in TOP1 reaction buffer (10 mM Tris-Cl pH 7.9, 1 mM EDTA, 150 mM NaCl, 0.1% BSA, 0.1 mM Spermidine, 5% glycerol). Supercoiled pUC19 plasmid DNA (200 ng per reaction) was incubated with TOP1 for varying lengths of time at 37 °C, and reactions were terminated using 5× Stopping Buffer (5% sarkosyl, 0.0025% bromophenol blue, 25% glycerol). Samples were resolved on a 1% agarose gel in TAE buffer at 2.5 V/cm for 6 h. Gel was washed once with deionized water, followed by staining with 1 µg/mL ethidium bromide. Since EtBr induces positive supercoiling in covalently closed circular DNA, post-staining was performed in order to avoid EtBr-induced topological perturbations in TOP1-relaxed plasmid intermediates.

### TOP1 cleavage and religation assays

TOP1 cleavage and religation kinetics were measured using immunoprecipitated protein. EGFP-TOP1^WT^ and EGFP-TOP1^S320A^ were ectopically expressed in U2-OS cells and recovered through immunoprecipitation as described above for plasmid relaxation assays. Cleavage and religation reactions were performed as described elsewhere (Ottaviani et al, 2025). DNA Oligonucleotide CL14-FITC (5′-GAAAAAA**G**ACTTAG-3′) and its complementary strand CP25 (5′-TAAAAATTTTTCTAAGTCTTTTTTC-3′) were used to generate the reaction substrate. CL14 was FITC tagged at a guanine (bold and underlined) to visualize various reaction intermediates. CP25 was phosphorylated at 5’ end to avoid spurious religation products. CL14 was annealed with a twofold molar excess of CP25 to obtain the suicide substrate. For cleavage reactions, the suicide substrate was incubated with beads bearing TOP1 immunocomplexes at 37 °C for 5, 10, or 20 min, and reactions were terminated at requisite timepoints using 0.5% SDS. For religation reactions, the suicide substrate was incubated with beads bearing TOP1 immunocomplexes at 25 °C for 60 min followed by 30 min at 37 °C. All reactions were carried out in TOP1 reaction buffer (20 mM Tris HCl pH 7.5, 0.1 mM EDTA, 10 mM MgCl_2_, 50 µg/mL acetylated BSA, 150 mM KCl). An aliquot was removed and saved as the 0 min sample. Religation reaction was then initiated by adding a 200-fold molar excess of R11 oligonucleotide (5′-AGAAAAATTTT-3′), followed by removal of aliquots are requisite timepoints. Reactions were stopped using 0.5% SDS. All samples were heated at 80 degrees for 10 min to release TOP1 immunocomplexes from GFP-trap magnetic agarose beads. Beads were discarded, followed by ethanol precipitation of the samples. Samples were subjected to tryptic digestion (5 µL trypsin from a 1 mg/mL stock) for 60 min at 37 °C. All samples were heated at 60 °C for 10 min with loading dye containing formamide, before resolving on denaturing PAGE (20% acrylamide; 7 M urea) in a Tris-Borate-EDTA buffer system. Due to incomplete action of trypsin on TOP1ccs, the retention of a trypsin-resistant peptide results in slower migration of the 12-nt fragment than the 14-nt CL14 oligonucleotide (this constitutes the reaction product quantified in the cleavage assay; this population is visible as a reaction intermediate in the religation reactions). Samples were visualized in the FITC channel using a G:Box chemi XX9 gel documentation system, followed by densitometry using ImageJ software.

### Preparation of crude nuclear extracts

Total cellular TOP1 activity was assayed as reported elsewhere (Nitiss et al, 2012) with minor modifications. Briefly, exponentially growing cells (5 ×  10^6^ cells per 150-mm dish) were subjected to relevant treatments, washed with ice-cold PBS, and harvested by scraping. The pellet was resuspended in 180 µL of ice-cold low salt extraction buffer (20 mM Tris-HCl pH 7.5, 5 mM KCl, 1 mM MgCl_2_, 10% v/v glycerol, protease inhibitor cocktail) and incubated on ice for 10 min. Cells were subjected to 15–20 strokes of a pre-cooled Dounce homogenizer, followed by 30 min; incubation on ice. Nuclei were recovered by centrifugation at 12,500 rpm for 3 min at 4 °C. Nuclear pellet was resuspended in 180 µL of ice-cold high salt extraction buffer (low salt extraction buffer with 350 mM KCl, supplemented with protease inhibitor cocktail), followed by 80 min incubation on ice. Nuclear extract was clarified by centrifugation at 12,500 rpm for 10 min at 4 °C. Total protein was quantified using the Bradford method, followed by an in vitro plasmid relaxation assay with different amounts of nuclear extract. For SCH-treated samples, low salt and high salt extraction buffers were supplemented with SCH (25 nM) to prevent CHK1 reactivation during lysis.

### Immunoprecipitation and co-immunoprecipitation

For both immunoprecipitation and co-immunoprecipitation experiments, 5 × 10^6^ cells were seeded per dish in 150 mm dishes. Cells were transfected with 15 µg of requisite plasmids 16 h post transfection by the calcium chloride method. The cell medium was replaced with fresh medium 24 h post transfection. Cells were incubated with the fresh medium for 24 h, followed by washing with ice-cold PBS, and harvesting by scraping. Cell pellets were washed once with PBS, and lysed with IP lysis buffer (50 mM Tris-HCl pH 8.0, 300 mM NaCl, 0.4% NP-40, protease inhibitor cocktail), supplemented with DNase I digestion buffer (1× final concentration from a 10X stock), 10 U/mL DNase I and 5 U/mL benzonase. Cells were lysed on ice for 1 h with periodic vortexing. Cell lysates were centrifuged at 12,500 rpm for 20 min to eliminate cellular debris. The supernatants were collected and diluted to 1 mL volume with IP dilution buffer (50 mM Tris-HCl pH 8.0, 0.4% NP-40, protease inhibitor cocktail). Total protein was quantified by the Bradford method, and 5% of each lysate was saved as input. Further, 1 mg protein per sample was incubated with 4 µg anti-GFP antibody overnight at 4 °C on a rotary shaker. Protein A/G magnetic agarose beads were equilibrated with IP dilution buffer, and 25 µl beads were added per sample and incubated for 4 h at 4 °C on a rotary shaker. Beads were then washed, boiled with SDS sample buffer, and samples were analyzed by western blotting using relevant antibodies.

### Immunoprecipitation of active wild-type and mutant TOP1 from human cells

For immunoprecipitation of TOP1, 5 × 10^6^ cells were seeded in 150 mm dishes, and transfected with 15 µg each of EGFP-TOP^WT^ or EGFP-TOP1^S320A^ plasmid by the calcium chloride method. 48 h later, cells were harvested by scraping. Cells were lysed in IP lysis buffer (10 mM Tris-HCl pH 7.5, 150 mM NaCl, 0.5 mM EDTA, 0.5% NP-40), supplemented with protease inhibitor cocktail, 10 U/mL DNase I and 5 U/mL benzonase. Lysis was performed on ice for 1 h. Lysate was clarified by centrifugation at 12500 rpm for 20 min, and diluted to 1 mL volume in IP dilution buffer (10 mM Tris-HCl pH 7.5, 150 mM NaCl, 0.5 mM EDTA) supplemented with protease inhibitor cocktail. Lysates were subjected to total protein quantification (Bradford method), and 1 mg lysate per sample was subjected to immunoprecipitation overnight using GFP-Trap Magnetic Agarose (ChromoTek GmBh) at 4 °C on a rotary shaker. 5% volume of each sample was reserved as input. Beads were washed thrice with IP wash buffer (10 mM Tris-HCl pH 7.5, 150 mM NaCl, 0.5 mM EDTA, 0.05% NP-40) supplemented with protease inhibitor cocktail, and resuspended in 50 µl of TOP1 reaction buffer (100 mM Tris-Cl (pH 7.9), 10 mM EDTA, 1.5 M NaCl, 1% BSA, 1 mM Spermidine, 50% glycerol). For verification of immunoprecipitation efficiency, 10 µl of beads were removed and boiled with SDS sample buffer, followed by western blotting with anti-GFP antibody. The remaining beads were used for plasmid relaxation assays.

### Chromatin immunoprecipitation

For chromatin immunoprecipitation, 5 × 10^6^ cells were seeded in 150 mm dishes. Cells were transfected with the requisite plasmids by the calcium chloride method. 48 h post transfection, cells were harvested by scraping and washed with ice-cold PBS. Cell pellet was resuspended in ice-cold swelling buffer (25 mM HEPES pH 7.8, 1.5 mM MgCl_2_, 10 mM KCl, 0.1% NP-40, and protease inhibitor cocktail), and incubated on ice for 10 min. Cells were disrupted with 20 strokes of a Dounce homogenizer. Nuclei were precipitated at 2000 rpm for 5 min followed by resuspension in sonication buffer (50 mM HEPES pH 7.8, 140 mM NaCl, 1 mM EDTA, 1% Triton X-100, 0.1% sodium deoxycholate, 0.1% SDS, protease inhibitor cocktail), and sonication on ice for 18 cycles (10 s on/30 s off; 50% amplitude) with a Cole-Parmer ultrasonic processor. Samples were centrifuged at 13,000 rpm for 15 min at 4 °C. Supernatant was recovered, and total DNA was spectrophotometrically quantified. 100 µg of genomic DNA per sample was incubated overnight with pre-equilibrated GFP-Trap Magnetic Agarose beads at 4 °C. Further, 5% DNA from each sample was reserved for the preparation of the input sample. Beads were subjected to two washes each with sonication buffer, wash buffer A (50 mM HEPES pH 7.8, 500 mM NaCl, 1 mM EDTA, 1% Triton X-100, 0.1% sodium deoxycholate, 0.1% SDS, protease inhibitor cocktail), and TE buffer (10 mM Tris-HCl pH 8, 1 mM EDTA), respectively. Beads were then treated with elution buffer (50 mM Tris pH 8, 1 mM EDTA, 1% SDS, 50 mM sodium bicarbonate) for 15 min at 65 °C. Elution was performed twice, and the eluates were combined. Samples were digested with RNase A for 1 h at 37 °C. Samples were supplemented with EDTA and digested with Proteinase K for 2 h at 42 °C. Samples were extracted using the phenol–chloroform method, followed by overnight ethanol precipitation in the presence of Glycogen. Samples were recovered with centrifugation, washed once with 70% ethanol, and resuspended in an appropriate volume of 10 mM Tris-HCl pH 8.

Quantitative real-time PCR was performed using SYBR green method. Details of primers employed are provided in Appendix Table S4. All quantifications were made in terms of percentage of input, and the relative ChIP signal was determined based on β-Actin TSS of EGFP-TOP1^WT^ sample, which was set to 1.

### DNA/RNA hybrid immunoprecipitation (DRIP)

For immunoprecipitation of RNA/DNA hybrids, nuclei were isolated as described for immunoprecipitation. Isolated nuclei were lysed on ice with nuclear lysis buffer (50 mM Tris-HCl pH 8.0, 5 mM EDTA, 1% SDS) for 30 min. Nuclear lysates were digested with Proteinase K for 3 h at 55 °C. Genomic DNA was then precipitated with 100% ethanol, washed once with 70% ethanol, and resuspended in IP dilution buffer (16.7 mM Tris-HCl pH 8.0, 1.2 mM EDTA, 167 mM NaCl, 0.01% SDS, 1.1% Triton X-100). Genomic DNA was sonicated as described for chromatin immunoprecipitation. Sonicated DNA was quantified, and 100 µg DNA was incubated overnight with 4 µg of S9.6 antibody and a 1:1 mix of pre-equilibriated Protein A/Protein G magnetic beads at 4°C. In addition, 5% DNA was stored as input. Post incubation steps were identical to those described for chromatin immunoprecipitation. To test the RNase H sensitivity of the signal, samples were treated with 2 U of RNase H before being subjected to immunoprecipitation. An appropriate isotype control was also included. Determination of the relative DRIP signal was identical to chromatin immunoprecipitation. Primers used for qRT-PCR are detailed in Appendix Table S4.

### Metaphase preparations

Metaphase spreads were prepared as described elsewhere (Schmid et al, 2018) with minor modifications. Briefly, cells were grown in 60-mm dishes to sub-confluence. Cells were then incubated in fresh culture medium supplemented with 200 ng/mL nocodazole for 16 h. Cells were then harvested through mitotic shake-off and washed once with pre-warmed PBS. Cells were then resuspended in swelling buffer (75 mM KCl) and incubated at 37 °C for 6 min. Cells were centrifuged at 1800 rpm, for 10 min, followed by resuspension in fixative solution containing methanol and acetic acid in a ratio of 3:1. The fixation step was repeated twice, followed by resuspension in 200 µL of fixative. Cells were dropped on chilled, pre-hydrated glass slides and dried immediately on a hot plate. The dried slides were immersed in a Coplin jar containing 5% solution of Giemsa stain in phosphate buffer. Slides were stained for 20 min, followed by thorough washing with nanopure water. Images were captured with a light microscope at ×100 magnification. A minimum of 100 metaphase spreads were analyzed per sample.

### DNA fiber analysis

DNA fiber analysis was performed as described elsewhere (Biber and Wiesmüller, 2021; Pai Bellare et al, 2025) with minor modifications. Briefly, 2 × 10^5^ cells were plated per well in six-well plates and lipofected with EGFP-TOP1^WT^ and EGFP-TOP1^S320A^. Fort-eight hours post transfection, cells were sequentially labeled with 30 µM CldU and 300 µM IdU (20 min each) with 3 washes with excess volume of pre-warmed PBS in between. Cells were harvested post labeling, counted, and resuspended at a concentration of 1 × 10^6^ cells/mL. Labeled and unlabeled cells were mixed at a ratio of 1:4, and 2.5 µL of the cell suspension was deposited on a glass slide. This was followed by the addition of 6 µL of lysis buffer (0.5% SDS, 200 mM Tris-HCl pH704 and 50 mM EDTA). The slide was incubated for 6 min at room temperature, followed by tilting the slide at ~30°, allowing the droplet to spread down the slide under gravity. The slide was dried, followed by fixation for 15 min with a methanol–acetic acid mixture (3:1). Denaturation and antibody treatments were as per published protocol. Slides were mounted in 80% glycerol. Samples were visualized with a Leica MICA system under ×63 water immersion objective. Image analysis was performed with ImageJ.

### Alphafold multimer (AFM) predictions

Predictions for CHK1–TOP1 interaction were made employing Alphafold Multimer. Data analysis was performed on the Cosmic^2^ server (https://cosmic2.sdsc.edu:8443/gateway/). Predictions were performed using Colabfold, employing alphafold2_multimer_v3 (Mirdita et al, 2022; preprint: Evans et al, 2021). The employed settings were as follows: number of models 5, number of recycles 3, Stop at score (Compute recycle models until plddt (single chain) or ptm score (complex) > threshold is reached) 80. pdb100 was used as a template for published PDB structures. For TOP1, amino acids 201–765 were used for predictions. For CHK1, the following regions were employed: full length (1-476), Kinase domain (1–265), and KA1 domain (391–476). Model visualizations and analyses of pLDDT, pTM, iPTM, and PAE values were performed using the PAE Viewer webserver (http://www.subtiwiki.uni-goettingen.de/v4/paeViewerDemo) (Elfmann and Stülke, 2023). Analyses of pDOCKQ and SPOC scores were performed on the Predictome webserver (https://predictomes.org/) (Schmid and Walter, 2025).

### Statistical analysis

All statistical analyses were performed on Graphpad Prism 8. Tests employed to derive statistical significance are indicated in individual figure legends.

## Supplementary information

Appendix

Peer Review File

Source data Fig. 1

Source data Fig. 2

Source data Fig. 3

Source data Fig. 4

Source data Fig. 5

Source data Fig. 6

Source data Fig. 7

Source data Fig. 8

Source data Fig. 9

Figure EV1 Source Data

Figure EV2 Source Data

Figure EV3 Source Data

Expanded View Figures

## Data Availability

The proteomics data associated with this study have been submitted to the ProteomeXchange Consortium *via* the PRIDE partner repository (Vizcaíno et al, 2013) (https://www.ebi.ac.uk/pride/markdownpage/proteomexchange) with accession number PXD055870. The source data of this paper are collected in the following database record: biostudies:S-SCDT-10_1038-S44318-026-00783-3.

## References

[CR1] Al-kaabi MM, Alshareeda AT, Jerjees DA, Muftah AA, Green AR, Alsubhi NH, Nolan CC, Chan S, Cornford E, Madhusudan S et al (2015) Checkpoint kinase1 (CHK1) is an important biomarker in breast cancer having a role in chemotherapy response. Br J Cancer 112:901–911 25688741 10.1038/bjc.2014.576 PMC4453942

[CR2] Ando K, Shah AK, Sachdev V, Kleinstiver BP, Taylor-Parker J, Welch MM, Hu Y, Salgia R, White FM, Parvin JD et al (2017) Camptothecin resistance is determined by the regulation of topoisomerase I degradation mediated by ubiquitin proteasome pathway. Oncotarget 8:43733–43751 28415827 10.18632/oncotarget.16376 PMC5546437

[CR3] Bandyopadhyay K, Gjerset RA (2011) Protein kinase CK2 is a central regulator of topoisomerase I hyperphosphorylation and camptothecin sensitivity in cancer cell lines. Biochemistry 50:704–714 21182307 10.1021/bi101110e PMC3046806

[CR4] Bandyopadhyay K, Li P, Gjerset RA (2012) CK2-mediated hyperphosphorylation of topoisomerase I targets serine 506, enhances topoisomerase I–DNA binding, and increases cellular camptothecin sensitivity. PLoS ONE 7:e50427 23185622 10.1371/journal.pone.0050427 PMC3503890

[CR5] Bao S, Wu Q, McLendon RE, Hao Y, Shi Q, Hjelmeland AB, Dewhirst MW, Bigner DD, Rich JN (2006) Glioma stem cells promote radioresistance by preferential activation of the DNA damage response. Nature 444:756–760 17051156 10.1038/nature05236

[CR6] Baranello L, Wojtowicz D, Cui K, Devaiah BN, Chung H-J, Chan-Salis KY, Guha R, Wilson K, Zhang X, Zhang H et al (2016) RNA polymerase II regulates topoisomerase 1 activity to favor efficient transcription. Cell 165:357–371 27058666 10.1016/j.cell.2016.02.036 PMC4826470

[CR7] Berti M, Ray Chaudhuri A, Thangavel S, Gomathinayagam S, Kenig S, Vujanovic M, Odreman F, Glatter T, Graziano S, Mendoza-Maldonado R et al (2013) Human RECQ1 promotes restart of replication forks reversed by DNA topoisomerase I inhibition. Nat Struct Mol Biol 20:347–354 23396353 10.1038/nsmb.2501 PMC3897332

[CR8] Biber S, Wiesmüller L (2021) Analysis of replication dynamics using the single-molecule DNA fiber spreading assay. Cell Cycle Checkpoints: Methods Protoc 2267:57–71 10.1007/978-1-0716-1217-0_4 33786784

[CR9] Bickel D, Vranken W (2024) Effects of phosphorylation on protein backbone dynamics and conformational preferences. J Chem Theory Comput 20:4998–5011 38830621 10.1021/acs.jctc.4c00206 PMC11210476

[CR10] Blasius M, Forment JV, Thakkar N, Wagner SA, Choudhary C, Jackson SP (2011) A phospho-proteomic screen identifies substrates of the checkpoint kinase Chk1. Genome Biol 12:R78 21851590 10.1186/gb-2011-12-8-r78 PMC3245618

[CR11] Carey JF, Schultz SJ, Sisson L, Fazzio TG, Champoux JJ (2003) DNA relaxation by human topoisomerase I occurs in the closed clamp conformation of the protein. Proc Natl Acad Sci USA 100:5640–5645 12711735 10.1073/pnas.1031537100 PMC156254

[CR12] Chappidi N, Nascakova Z, Boleslavska B, Zellweger R, Isik E, Andrs M, Menon S, Dobrovolna J, Balbo Pogliano C, Matos J et al (2020) Fork cleavage-religation cycle and active transcription mediate replication restart after fork stalling at co-transcriptional R-Loops. Mol Cell 77:528–541.e8 31759821 10.1016/j.molcel.2019.10.026

[CR13] Chillemi G, Castrignanò T, Desideri A (2001) Structure and hydration of the DNA-human topoisomerase I covalent complex. Biophys J 81:490–500 11423431 10.1016/S0006-3495(01)75716-5 PMC1301528

[CR14] Chowdhuri SP, Das BB (2021) Top1-PARP1 association and beyond: from DNA topology to break repair. NAR Cancer 3:zcab003 33981998 10.1093/narcan/zcab003 PMC8095074

[CR15] Crewe M, Madabhushi R (2021) Topoisomerase-mediated DNA damage in neurological disorders. Front Aging Neurosci 13:751742 34899270 10.3389/fnagi.2021.751742 PMC8656403

[CR16] Cristini A, Ricci G, Britton S, Salimbeni S, Huang S-YN, Marinello J, Calsou P, Pommier Y, Favre G, Capranico G et al (2019) Dual processing of R-loops and topoisomerase I induces transcription-dependent DNA double-strand breaks. Cell Rep 28:3167–3181.e6 31533039 10.1016/j.celrep.2019.08.041 PMC8274950

[CR17] Das SK, Rehman I, Ghosh A, Sengupta S, Majumdar P, Jana B, Das BB (2016) Poly(ADP-ribose) polymers regulate DNA topoisomerase I (Top1) nuclear dynamics and camptothecin sensitivity in living cells. Nucleic Acids Res 44:8363–8375 27466387 10.1093/nar/gkw665 PMC5041477

[CR18] David L, Fernandez-Vidal A, Bertoli S, Grgurevic S, Lepage B, Deshaies D, Prade N, Cartel M, Larrue C, Sarry J-E et al (2016) CHK1 as a therapeutic target to bypass chemoresistance in AML. Sci Signal 9:ra90 27625304 10.1126/scisignal.aac9704

[CR19] Desai SD, Liu LF, Vazquez-Abad D, D’Arpa P (1997) Ubiquitin-dependent destruction of topoisomerase I is stimulated by the antitumor drug camptothecin. J Biol Chem 272:24159–24164 9305865 10.1074/jbc.272.39.24159

[CR20] Duardo RC, Marinello J, Russo M, Morelli S, Pepe S, Guerra F, Gómez-González B, Aguilera A, Capranico G (2024) Human DNA topoisomerase I poisoning causes R loop–mediated genome instability attenuated by transcription factor IIS. Sci Adv 10:eadm8196 38787953 10.1126/sciadv.adm8196 PMC11122683

[CR21] Elfmann C, Stülke J (2023) PAE viewer: a webserver for the interactive visualization of the predicted aligned error for multimer structure predictions and crosslinks. Nucleic Acids Res 51:W404–W410 37140053 10.1093/nar/gkad350 PMC10320053

[CR22] Evans R, O’Neill M, Pritzel A, Antropova N, Senior A, Green T, Žídek A, Bates R, Blackwell S, Yim J et al (2021) Protein complex prediction with AlphaFold-Multimer. Preprint at bioRxiv 10.1101/2021.10.04.463034

[CR23] Fielden J, Wiseman K, Torrecilla I, Li S, Hume S, Chiang S-C, Ruggiano A, Narayan Singh A, Freire R, Hassanieh S et al (2020) TEX264 coordinates p97- and SPRTN-mediated resolution of topoisomerase 1-DNA adducts. Nat Commun 11:1274 32152270 10.1038/s41467-020-15000-w PMC7062751

[CR24] Flatten K, Dai NT, Vroman BT, Loegering D, Erlichman C, Karnitz LM, Kaufmann SH (2005) The role of checkpoint kinase 1 in sensitivity to topoisomerase I poisons. J Biol Chem 280:14349–14355 15699047 10.1074/jbc.M411890200

[CR25] Grabauskiene S, Bergeron EJ, Chen G, Chang AC, Lin J, Thomas DG, Giordano TJ, Beer DG, Morgan MA, Reddy RM (2013) CHK1 levels correlate with sensitization to pemetrexed by CHK1 inhibitors in non-small cell lung cancer cells. Lung Cancer 82:477–484 24113549 10.1016/j.lungcan.2013.09.010 PMC4073640

[CR26] Guha, Majumdar A, Shree S, Das A, Kumar BK, Dey P, Subramanian M, Patro BS (2023) Design, synthesis and development of a dual inhibitor of Topoisomerase 1 and poly (ADP-ribose) polymerase 1 for efficient killing of cancer cells. Eur J Med Chem 258:115598 37406384 10.1016/j.ejmech.2023.115598

[CR27] Guha Majumdar A, Subramanian M (2019) Hydroxychavicol from Piper betle induces apoptosis, cell cycle arrest, and inhibits epithelial-mesenchymal transition in pancreatic cancer cells. Biochem Pharm 166:274–291 31154000 10.1016/j.bcp.2019.05.025

[CR28] Gupta P, Majumdar AG, Patro BS (2022) Non-enzymatic function of WRN RECQL helicase regulates removal of topoisomerase-I-DNA covalent complexes and triggers NF-κB signaling in cancer. Aging Cell 21:e13625 35582959 10.1111/acel.13625 PMC9197415

[CR29] Hackbarth JS, Galvez-Peralta M, Dai NT, Loegering DA, Peterson KL, Meng XW, Karnitz LM, Kaufmann SH (2008) Mitotic phosphorylation stimulates DNA relaxation activity of human topoisomerase I. J Biol Chem 283:16711–16722 18408216 10.1074/jbc.M802246200 PMC2423254

[CR30] Halder S, Torrecilla I, Burkhalter MD, Popović M, Fielden J, Vaz B, Oehler J, Pilger D, Lessel D, Wiseman K, Singh AN, Vendrell I, Fischer R, Philipp M, Ramadan K (2019) SPRTN protease and checkpoint kinase 1 cross-activation loop safeguards DNA replication. Nat Commun 10:3142 31316063 10.1038/s41467-019-11095-y PMC6637133

[CR31] He E, Yan G, Zhang J, Wang J, Li W (2016) Effects of phosphorylation on the intrinsic propensity of backbone conformations of serine/threonine. J Biol Phys 42:247–258 26759163 10.1007/s10867-015-9405-0 PMC4788628

[CR32] Hidmi O, Oster S, Monin J, Aqeilan RI (2024) TOP1 and R-loops facilitate transcriptional DSBs at hypertranscribed cancer driver genes. iScience 27:109082 38375218 10.1016/j.isci.2024.109082 PMC10875566

[CR33] Iyer DR, Rhind N (2017) The intra-S checkpoint responses to DNA damage. Genes 8:74 28218681 10.3390/genes8020074 PMC5333063

[CR34] Jossé R, Martin SE, Guha R, Ormanoglu P, Pfister TD, Reaper PM, Barnes CS, Jones J, Charlton P, Pollard JR et al (2014) ATR inhibitors VE-821 and VX-970 sensitize cancer cells to topoisomerase I inhibitors by disabling DNA replication initiation and fork elongation responses. Cancer Res 74:6968–6979 25269479 10.1158/0008-5472.CAN-13-3369 PMC4252598

[CR35] Kiianitsa K, Maizels N (2013) A rapid and sensitive assay for DNA–protein covalent complexes in living cells. Nucleic Acids Res 41:e104 23519618 10.1093/nar/gkt171 PMC3643584

[CR36] Lascaux P, Hoslett G, Tribble S, Trugenberger C, Antičević I, Otten C, Torrecilla I, Koukouravas S, Zhao Y, Yang H et al (2024) TEX264 drives selective autophagy of DNA lesions to promote DNA repair and cell survival. Cell 187:5698–5718.e26 39265577 10.1016/j.cell.2024.08.020

[CR37] Lin C-P, Ban Y, Lyu YL, Desai SD, Liu LF (2008) A ubiquitin-proteasome pathway for the repair of topoisomerase I-DNA covalent complexes. J Biol Chem 283:21074–21083 18515798 10.1074/jbc.M803493200 PMC2475699

[CR38] Lin C-P, Ban Y, Lyu YL, Liu LF (2009) Proteasome-dependent processing of topoisomerase I-DNA adducts into DNA double strand breaks at arrested replication forks. J Biol Chem 284:28084–28092 19666469 10.1074/jbc.M109.030601 PMC2788859

[CR39] Marabitti V, Lillo G, Malacaria E, Palermo V, Sanchez M, Pichierri P, Franchitto A (2019) ATM pathway activation limits R-loop-associated genomic instability in Werner syndrome cells. Nucleic Acids Res 47:3485–3502 30657978 10.1093/nar/gkz025 PMC6468170

[CR40] McDonald M, Trost B, Napper S (2018) Conservation of kinase-phosphorylation site pairings: evidence for an evolutionarily dynamic phosphoproteome. PLoS ONE 13:e0202036 30106995 10.1371/journal.pone.0202036 PMC6091962

[CR41] Meroni A, Grosser J, Agashe S, Ramakrishnan N, Jackson J, Verma P, Baranello L, Vindigni A (2022) NEDDylated Cullin 3 mediates the adaptive response to topoisomerase 1 inhibitors. Sci Adv 8:eabq0648 36490343 10.1126/sciadv.abq0648 PMC9733930

[CR42] Michelena J, Gatti M, Teloni F, Imhof R, Altmeyer M (2019) Basal CHK1 activity safeguards its stability to maintain intrinsic S-phase checkpoint functions. J Cell Biol 218:2865–2875 31366665 10.1083/jcb.201902085 PMC6719454

[CR43] Mirdita M, Schütze K, Moriwaki Y, Heo L, Ovchinnikov S, Steinegger M (2022) ColabFold: making protein folding accessible to all. Nat Methods 19:679–682 35637307 10.1038/s41592-022-01488-1 PMC9184281

[CR44] Moiseeva TN, Yin Y, Calderon MJ, Qian C, Schamus-Haynes S, Sugitani N, Osmanbeyoglu HU, Rothenberg E, Watkins SC, Bakkenist CJ (2019) An ATR and CHK1 kinase signaling mechanism that limits origin firing during unperturbed DNA replication. Proc Natl Acad Sci USA 116:13374–13383 31209037 10.1073/pnas.1903418116 PMC6613105

[CR45] Montano R, Chung I, Garner KM, Parry D, Eastman A (2012) Preclinical development of the novel Chk1 inhibitor SCH900776 in combination with DNA damaging agents and antimetabolites. Mol Cancer Ther 11:427–438 22203733 10.1158/1535-7163.MCT-11-0406 PMC3277678

[CR46] Moreira F, Arenas M, Videira A, Pereira F (2023) Evolution of TOP1 and TOP1MT topoisomerases in chordata. J Mol Evol 91:192–203 36651963 10.1007/s00239-022-10091-z PMC10081982

[CR47] Neizer-Ashun F, Bhattacharya R (2021) Reality CHEK: understanding the biology and clinical potential of CHK1. Cancer Lett 497:202–211 32991949 10.1016/j.canlet.2020.09.016

[CR48] Nitiss JL, Soans E, Rogojina A, Seth A, Mishina M (2012) Topoisomerase assays. Curr Protoc Pharm Chapter 3:Unit3.3 10.1002/0471141755.ph0303s57 PMC3397423 22684721

[CR49] Ottaviani A, Pietrafesa D, Soren BC, Dasari JB, Olsen SSH, Messina B, Demofonti F, Chicarella G, Agama K, Pommier Y et al (2025) Unveiling the mechanism of action of palmitic acid, a human topoisomerase 1B inhibitor from the Antarctic sponge *Artemisina plumosa*. Int J Mol Sci 26:2018 40076642 10.3390/ijms26052018 PMC11900379

[CR50] Pai Bellare G, Kundu K, Dey P, Philip KT, Chauhan N, Sharma M, Rajput SK, Patro BS (2025) Targeting replication fork processing synergizes with PARP inhibition to potentiate lethality in homologous recombination proficient ovarian cancers. Adv Sci 12:2410718 10.1002/advs.202410718 PMC12079468 40089867

[CR51] Patel AG, Flatten KS, Peterson KL, Beito TG, Schneider PA, Perkins AL, Harki DA, Kaufmann SH (2016) Immunodetection of human topoisomerase I-DNA covalent complexes. Nucleic Acids Res 44:2816–2826 26917015 10.1093/nar/gkw109 PMC4824114

[CR52] Patil M, Pabla N, Dong Z (2013) Checkpoint kinase 1 in DNA damage response and cell cycle regulation. Cell Mol Life Sci 70:4009–4021 23508805 10.1007/s00018-013-1307-3 PMC3731415

[CR53] Patro BS, Frøhlich R, Bohr VA, Stevnsner T (2011) WRN helicase regulates the ATR–CHK1-induced S-phase checkpoint pathway in response to topoisomerase-I–DNA covalent complexes. J Cell Sci 124:3967–3979 22159421 10.1242/jcs.081372 PMC3244981

[CR54] Pommier Y, Barcelo JM, Rao VA, Sordet O, Jobson AG, Thibaut L, Miao Z, Seiler JA, Zhang H, Marchand C et al (2006) Repair of topoisomerase I-mediated DNA damage. Prog Nucleic Acid Res Mol Biol 81:179–229 16891172 10.1016/S0079-6603(06)81005-6 PMC2576451

[CR55] Pommier Y, Nussenzweig A, Takeda S, Austin C (2022) Human topoisomerases and their roles in genome stability and organization. Nat Rev Mol Cell Biol 23:407–427 35228717 10.1038/s41580-022-00452-3 PMC8883456

[CR56] Pommier Y, O’Connor MJ, de Bono J (2016a) Laying a trap to kill cancer cells: PARP inhibitors and their mechanisms of action. Sci Transl Med 8:362ps17 27797957 10.1126/scitranslmed.aaf9246

[CR57] Pommier Y, Sun Y, Huang S-YN, Nitiss JL (2016b) Roles of eukaryotic topoisomerases in transcription, replication and genomic stability. Nat Rev Mol Cell Biol 17:703–721 27649880 10.1038/nrm.2016.111 PMC9248348

[CR58] Saldivar JC, Hamperl S, Bocek MJ, Chung M, Bass TE, Cisneros-Soberanis F, Samejima K, Xie L, Paulson JR, Earnshaw WC et al (2018) An intrinsic S/G2 checkpoint enforced by ATR. Science 361:806–810 30139873 10.1126/science.aap9346 PMC6365305

[CR59] Sampath D, Shi Z, Plunkett W (2002) Inhibition of cyclin-dependent kinase 2 by the Chk1-Cdc25A pathway during the S-phase checkpoint activated by fludarabine: dysregulation by 7-hydroxystaurosporine. Mol Pharm 62:680–688 10.1124/mol.62.3.680 12181445

[CR60] Sari L, Andricioaei I (2005) Rotation of DNA around intact strand in human topoisomerase I implies distinct mechanisms for positive and negative supercoil relaxation. Nucleic Acids Res 33:6621 16314322 10.1093/nar/gki935 PMC1298917

[CR61] Schmid EW, Walter JC (2025) Predictomes: a classifier-curated database of AlphaFold-modeled protein-protein interactions. Mol Cell 85:1216–1232 10.1016/j.molcel.2025.01.034 PMC11931459 40015271

[CR62] Schmid JA, Berti M, Walser F, Raso MC, Schmid F, Krietsch J, Stoy H, Zwicky K, Ursich S, Freire R et al (2018) Histone ubiquitination by the DNA damage response is required for efficient DNA replication in unperturbed S phase. Mol Cell 71:897–910.e8 30122534 10.1016/j.molcel.2018.07.011

[CR63] Slotkin EK, Mauguen A, Ortiz MV, Dela Cruz FS, O’Donohue T, Kinnaman MD, Meyers PA, Wexler LH, Rodriguez S, Avutu V et al (2022) A phase I/II study of prexasertib in combination with irinotecan in patients with relapsed/refractory desmoplastic small round cell tumor and rhabdomyosarcoma. J Clin Oncol 40:11503

[CR64] Solier S, Ryan MC, Martin SE, Varma S, Kohn KW, Liu H, Zeeberg BR, Pommier Y (2013) Transcription poisoning by topoisomerase I is controlled by gene length, splice sites, and miR-142-3p. Cancer Res 73:4830–4839 23786772 10.1158/0008-5472.CAN-12-3504 PMC3874869

[CR65] Stewart L, Redinbo MR, Qiu X, Hol WG, Champoux JJ (1998) A model for the mechanism of human topoisomerase I. Science 279:1534–1541 9488652 10.1126/science.279.5356.1534

[CR66] Stockton S, Shyr C, Cecchini M, Aljumaily R, Halfdanarson TR, Sonbol MB, Whisenant J, Ivy SP, LoRusso P, Das S et al (2024) A phase I study of ATR inhibitor BAY1895344 (elimusertib) plus topotecan (ETCTN 10402): results of dose escalation. J Clin Oncol 42:3076

[CR67] Sun Y, Chen J, Huang SN, Su YP, Wang W, Agama K, Saha S, Jenkins LM, Pascal JM, Pommier Y (2021) PARylation prevents the proteasomal degradation of topoisomerase I DNA-protein crosslinks and induces their deubiquitylation. Nat Commun 12:5010 34408146 10.1038/s41467-021-25252-9 PMC8373905

[CR68] Sun Y, Miller Jenkins LM, Su YP, Nitiss KC, Nitiss JL, Pommier Y (2020a) A conserved SUMO pathway repairs topoisomerase DNA-protein cross-links by engaging ubiquitin-mediated proteasomal degradation. Sci Adv 6:eaba6290 33188014 10.1126/sciadv.aba6290 PMC7673754

[CR69] Sun Y, Saha LK, Saha S, Jo U, Pommier Y (2020b) Debulking of topoisomerase DNA-protein crosslinks (TOP-DPC) by the proteasome, non-proteasomal and non-proteolytic pathways. DNA Repair 94:102926 32674013 10.1016/j.dnarep.2020.102926 PMC9210512

[CR70] Veloso A, Biewen B, Paulsen MT, Berg N, Lima LC de A, Prasad J, Bedi K, Magnuson B, Wilson TE, Ljungman M (2013) Genome-wide transcriptional effects of the anti-cancer agent camptothecin. PLoS ONE 8:e78190 24194914 10.1371/journal.pone.0078190 PMC3806802

[CR71] Vizcaíno JA, Côté RG, Csordas A, Dianes JA, Fabregat A, Foster JM, Griss J, Alpi E, Birim M, Contell J, O’Kelly G, Schoenegger A, Ovelleiro D, Pérez-Riverol Y, Reisinger F, Ríos D, Wang R, Hermjakob H (2013) The PRoteomics IDEntifications (PRIDE) database and associated tools: status in 2013. Nucleic Acids Res 41:D1063–D1069 23203882 10.1093/nar/gks1262 PMC3531176

[CR72] Wang J-L, Wang X, Wang H, Iliakis G, Wang Y (2002) CHK1-regulated S-phase checkpoint response reduces camptothecin cytotoxicity. Cell Cycle 1:267–272 12429946

[CR73] Wang W-J, Wu S-P, Liu J-B, Shi Y-S, Huang X, Zhang Q-B, Yao K-T (2013) MYC regulation of CHK1 and CHK2 promotes radioresistance in a stem cell-like population of nasopharyngeal carcinoma cells. Cancer Res 73:1219–1231 23269272 10.1158/0008-5472.CAN-12-1408

[CR74] Yaneva D, Sparks JL, Donsbach M, Zhao S, Weickert P, Bezalel-Buch R, Stingele J, Walter JC (2023) The FANCJ helicase unfolds DNA-protein crosslinks to promote their repair. Mol Cell 83:43–56.e10 36608669 10.1016/j.molcel.2022.12.005 PMC9881729

[CR75] Yu D, Khan E, Khaleque MA, Lee J, Laco G, Kohlhagen G, Kharbanda S, Cheng Y-C, Pommier Y, Bharti A (2004) Phosphorylation of DNA topoisomerase I by the c-Abl tyrosine kinase confers camptothecin sensitivity. J Biol Chem 279:51851–51861 15448168 10.1074/jbc.M404396200

[CR76] Zeman MK, Cimprich KA (2014) Causes and consequences of replication stress. Nat Cell Biol 16:2–9 24366029 10.1038/ncb2897 PMC4354890

[CR77] Zhang P, Wei Y, Wang L, Debeb BG, Yuan Y, Zhang J, Yuan J, Wang M, Chen D, Sun Y et al (2014) ATM-mediated stabilization of ZEB1 promotes DNA damage response and radioresistance through CHK1. Nat Cell Biol 16:864–875 25086746 10.1038/ncb3013 PMC4150825

[CR78] Zhang Y, Hunter T (2014) Roles of Chk1 in cell biology and cancer therapy. Int J Cancer 134:1013–1023 23613359 10.1002/ijc.28226 PMC3852170

