# Supplementary material for: A CHK1-mediated phosphorylation switch suppresses human Topoisomerase 1-associated genomic instability
Source: EMBO J. 2026 May 13;45(12):4220–56. doi: 10.1038/s44318-026-00783-3 (PMC13270093; doi:10.1038/s44318-026-00783-3)
Supplement: Supplementary file 1 — Appendix [file 44318_2026_783_MOESM1_ESM.pdf]

Appendix

A CHK1-mediated phosphorylation switch suppresses human Topoisomerase 1-associated genomic instability

Ananda Guha Majumdar[1,2], Nitish Chauhan[1,2], Pooja Gupta[1,2], Mahesh Subramanian[1,2*] and Birija Sankar Patro[1,2*]

*Author for correspondence: (BSP) bisank@barc.gov.in; (MS) maheshs@barc.gov.in

Contents

Page No.

Appendix Figure S1. RADAR screening of kinase inhibitors. 3

Appendix Figure S2: RADAR screening of kinase inhibitors. 5

Appendix Figure S3. Regulation of TOP1 dynamics by CHK1. 7

Appendix Figure S4. Analysis of DNA damage status of cells exposed to CHK1 inhibition 9

Appendix Figure S5. Single stranded DNA accumulation in cells exposed to CHK1 inhibition. 11

Appendix Figure S6. Characterization of CHK1i-induced stabilization of TOP1ccs (contribution of replication and transcription, and targeting by cellular machinery). 13

Appendix Figure S7. *In silico* analysis of CHK1-TOP1 interaction. 15

Appendix Figure S8. Characterization of TOP1-CHK1 interaction. 16

Appendix Figure S9. Characterization of expression levels and immunoprecipitation efficiencies of EGFP-TOP1^{WT} and EGFP-TOP1^{S320A}. 18

Appendix Figure S10. Replication-dependent DNA damage and replication perturbations associated with EGFP-TOP1^{S320A} expression 19

Appendix Figure S11. Transfection efficiency of EGFP-TOP1^{WT} and EGFP-TOP1^{S320A}. 21

Appendix Figure S12. Characterization of transcription-associated DNA damage in cells expressing EGFP-TOP1^{S320A}. 22

Appendix Figure S13. Transcription-associated genomic instability in cells expressing EGFP-TOP1S320A. 23

Appendix Table S1: Kinase inhibitors, their targets, and concentrations employed in the RADAR screen 24

Appendix Table S2: Prediction of CHK1 target motifs on TOP1. While the entire motif is highlighted in green, the target residue is depicted in red. 25

Appendix Table S3: Details of sites found to be phosphorylated on catalytically active TOP1. Whether they have previously been reported elsewhere is also indicated. References pertaining to these studies are provided in the main text. 26

Appendix Table S4: Details of primers used in qPCR 27

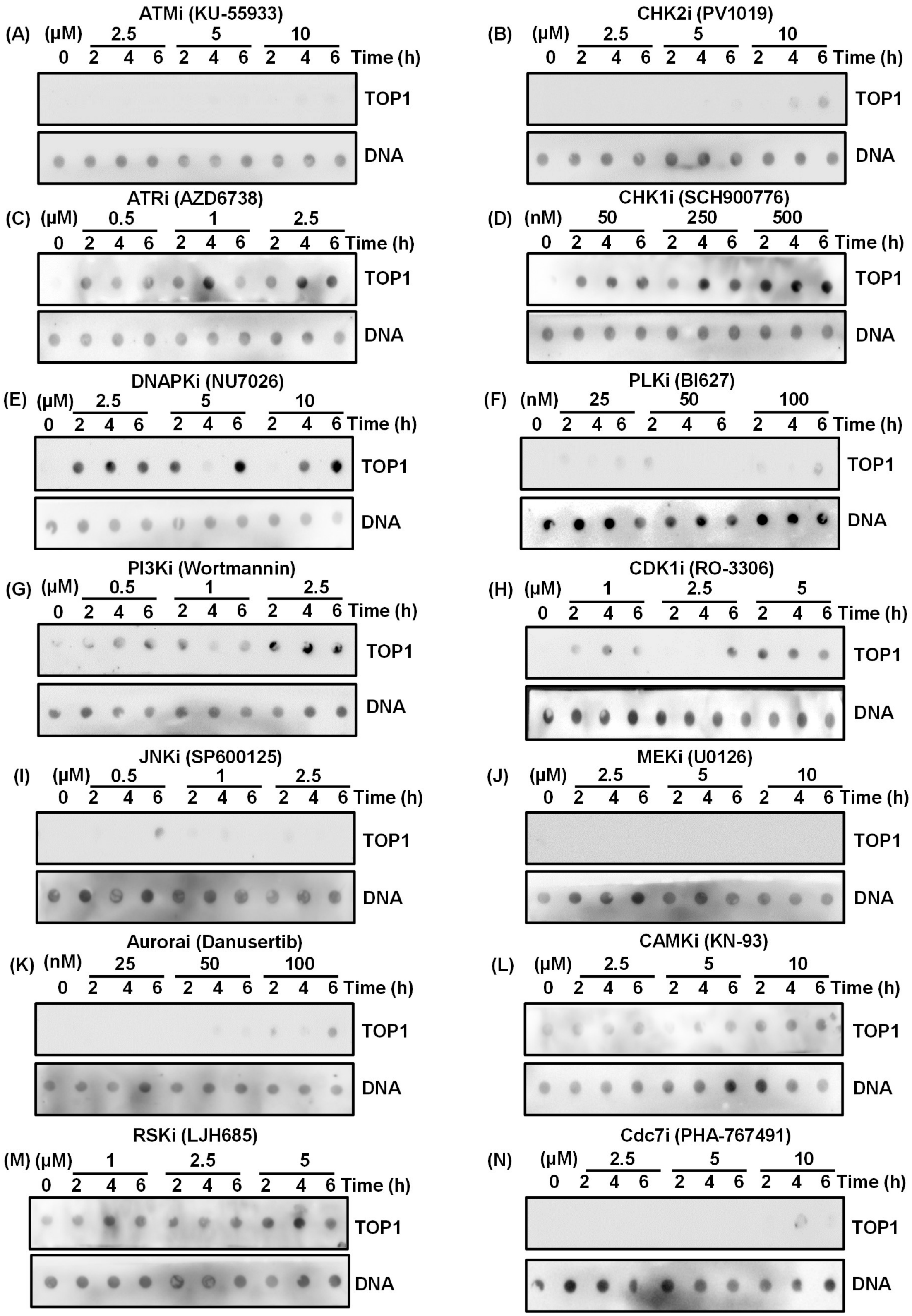
ATMi (KU-55933)
(A) (μM) 2.5 5 10
0 2 4 6 2 4 6 2 4 6 Time (h)
TOP1
DNA
CHK2i (PV1019)
(B) (μM) 2.5 5 10
0 2 4 6 2 4 6 2 4 6 Time (h)
TOP1
DNA
ATRi (AZD6738)
(C) (μM) 0.5 1 2.5
0 2 4 6 2 4 6 2 4 6 Time (h)
TOP1
DNA
CHK1i (SCH900776)
(D) (nM) 50 250 500
0 2 4 6 2 4 6 2 4 6 Time (h)
TOP1
DNA
DNAPKi (NU7026)
(E) (μM) 2.5 5 10
0 2 4 6 2 4 6 2 4 6 Time (h)
TOP1
DNA
PLKi (BI627)
(F) (nM) 25 50 100
0 2 4 6 2 4 6 2 4 6 Time (h)
TOP1
DNA
PI3Ki (Wortmannin)
(G) (μM) 0.5 1 2.5
0 2 4 6 2 4 6 2 4 6 Time (h)
TOP1
DNA
CDK1i (RO-3306)
(H) (μM) 1 2.5 5
0 2 4 6 2 4 6 2 4 6 Time (h)
TOP1
DNA
JNKi (SP600125)
(I) (μM) 0.5 1 2.5
0 2 4 6 2 4 6 2 4 6 Time (h)
TOP1
DNA
MEKi (U0126)
(J) (μM) 2.5 5 10
0 2 4 6 2 4 6 2 4 6 Time (h)
TOP1
DNA
Aurorai (Danusertib)
(K) (nM) 25 50 100
0 2 4 6 2 4 6 2 4 6 Time (h)
TOP1
DNA
CAMKi (KN-93)
(L) (μM) 2.5 5 10
0 2 4 6 2 4 6 2 4 6 Time (h)
TOP1
DNA
RSKi (LJH685)
(M) (μM) 1 2.5 5
0 2 4 6 2 4 6 2 4 6 Time (h)
TOP1
DNA
Cdc7i (PHA-767491)
(N) (μM) 2.5 5 10
0 2 4 6 2 4 6 2 4 6 Time (h)
TOP1
DNA

Appendix Figure S1. RADAR screening of kinase inhibitors.

(A-N) RADAR assay screen for TOP1cc trapping by small molecule kinase inhibitors. U2-OS cells were treated with indicated concentrations of inhibitors for 2, 4 or 6 h, followed by evaluation of TOP1cc stabilization through RADAR assay.

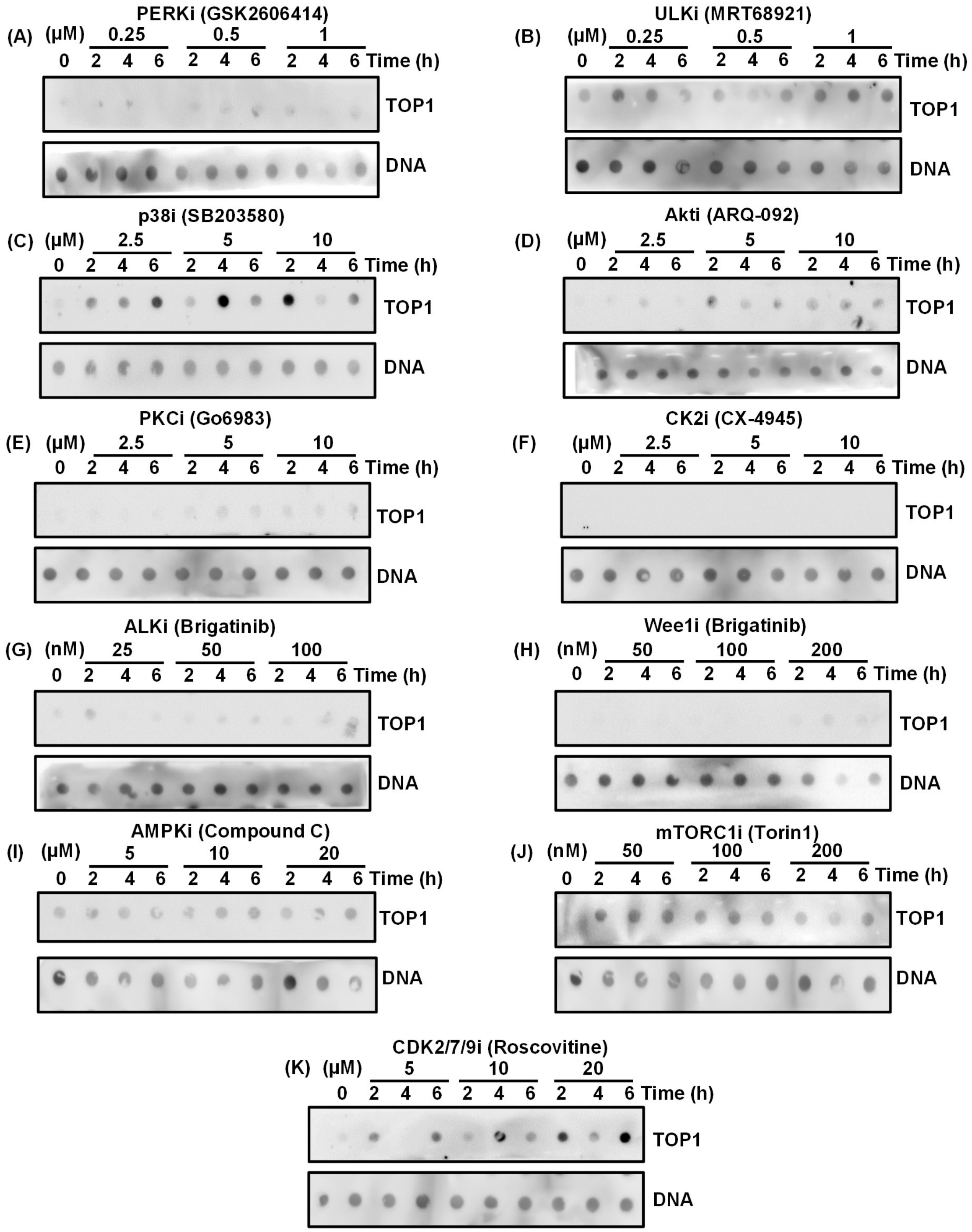
(A) PERKi (GSK2606414)
(μM) 0.25 0.5 1
0 2 4 6 2 4 6 2 4 6 Time (h)
TOP1
DNA
(B) ULKi (MRT68921)
(μM) 0.25 0.5 1
0 2 4 6 2 4 6 2 4 6 Time (h)
TOP1
DNA
(C) p38i (SB203580)
(μM) 2.5 5 10
0 2 4 6 2 4 6 2 4 6 Time (h)
TOP1
DNA
(D) Akti (ARQ-092)
(μM) 2.5 5 10
0 2 4 6 2 4 6 2 4 6 Time (h)
TOP1
DNA
(E) PKCi (Go6983)
(μM) 2.5 5 10
0 2 4 6 2 4 6 2 4 6 Time (h)
TOP1
DNA
(F) CK2i (CX-4945)
(μM) 2.5 5 10
0 2 4 6 2 4 6 2 4 6 Time (h)
TOP1
DNA
(G) ALKi (Brigatinib)
(nM) 25 50 100
0 2 4 6 2 4 6 2 4 6 Time (h)
TOP1
DNA
(H) Wee1i (Brigatinib)
(nM) 50 100 200
0 2 4 6 2 4 6 2 4 6 Time (h)
TOP1
DNA
(I) AMPKi (Compound C)
(μM) 5 10 20
0 2 4 6 2 4 6 2 4 6 Time (h)
TOP1
DNA
(J) mTORC1i (Torin1)
(nM) 50 100 200
0 2 4 6 2 4 6 2 4 6 Time (h)
TOP1
DNA
(K) CDK2/7/9i (Roscovitine)
(μM) 5 10 20
0 2 4 6 2 4 6 2 4 6 Time (h)
TOP1
DNA

Appendix Figure S2: RADAR screening of kinase inhibitors.

(A-K) RADAR assay screen for TOP1cc trapping by small molecule kinase inhibitors. U2-OS cells were treated with indicated concentrations of inhibitors for 2, 4 or 6 h, followed by evaluation of TOP1cc stabilization through RADAR assay.

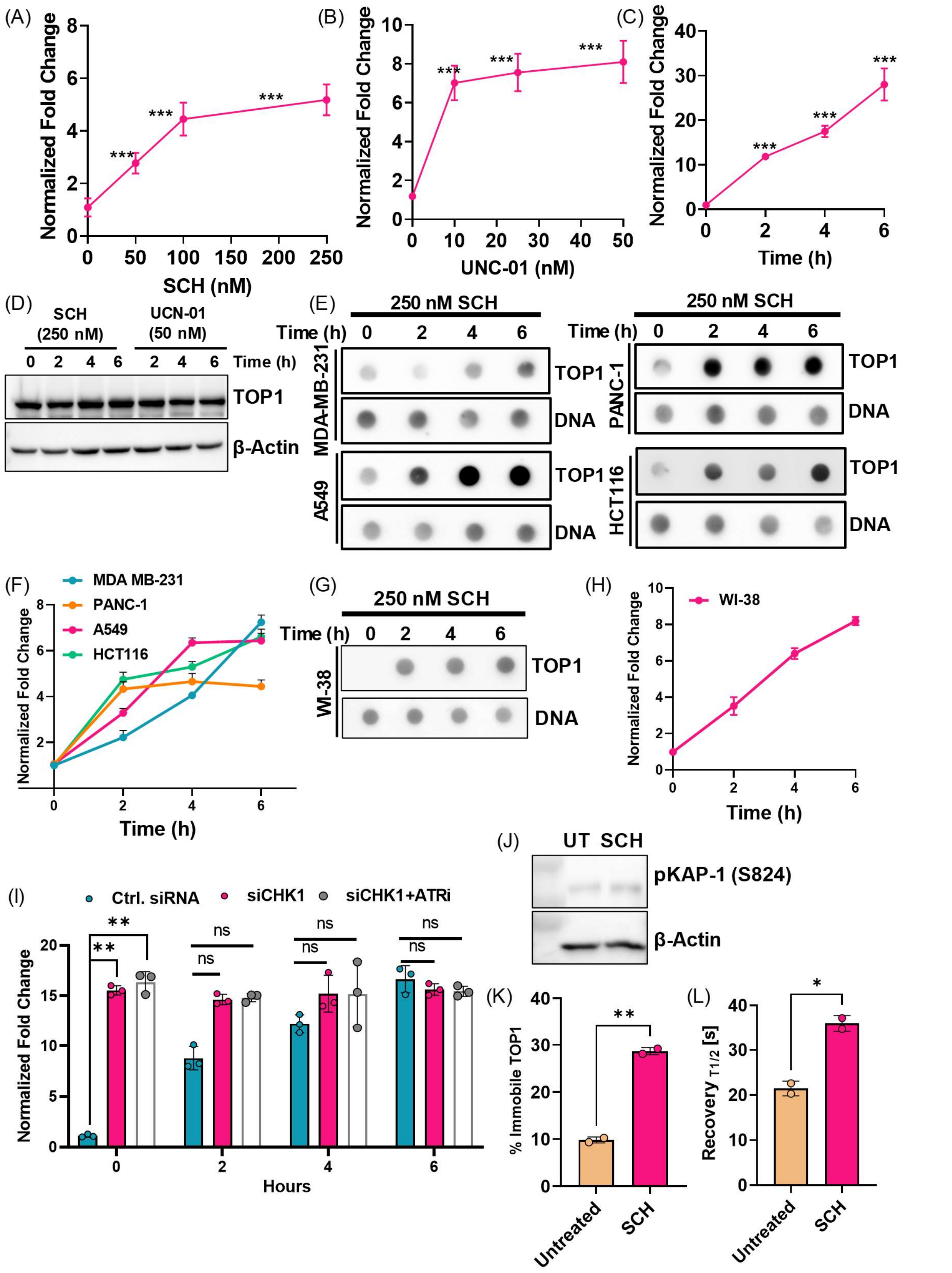

(A)
Normalized Fold Change
SCH (nM)
(B)
UNC-01 (nM)
(C)
Time (h)
(D)
SCH (250 nM)
UCN-01 (50 nM)
Time (h)
TOP1
β-Actin
(E)
250 nM SCH
MDA-MB-231
A549
PANC-1
HCT116
DNA
(F)
MDA MB-231
PANC-1
A549
HCT116
(G)
WI-38
(H)
(I)
Ctrl. siRNA
siCHK1
siCHK1+ATRi
Hours
(J)
UT SCH
pKAP-1 (S824)
(K)
% Immobile TOP1
Untreated
SCH
(L)
Recovery T1/2 [s]

Appendix Figure S3. Regulation of TOP1 dynamics by CHK1.

(A-C) Quantitative densitometric analyses of dot blots shown in Fig. 1C-E. (D) Global TOP1 levels in U2-OS cells treated with SCH (250 nM) or UCN-01(50 nM) for 2-,4- or 6 h using TOP1 downregulation assay (alkaline lysis method). (E, F) SCH-mediated TOP1cc stabilization in different cancer cells. MDA-MB-231, PANC-1, A549 and HCT116 cells were treated with SCH (250 nM) for 2, 4 or 6 h, followed by RADAR assay. (G, H) SCH-mediated TOP1cc stabilization in non-transformed WI-38 (human fetal lung) cells. (I) Densitometric quantification of Fig. 1E (mean±SEM) (J) Immunoblot for phospho-KAP-1 (S824) in U2-OS cells treated with SCH (250 nM) for 6 h. (K, L) Quantification across three replicates of data presented in Fig. 1K, L. Error bars represent SEM. ns: not significant. ** $P<0.01$; *** $P<0.001$ (Data from three independent experiments; significance was determined using one-way ANOVA). Error bars represent SD.

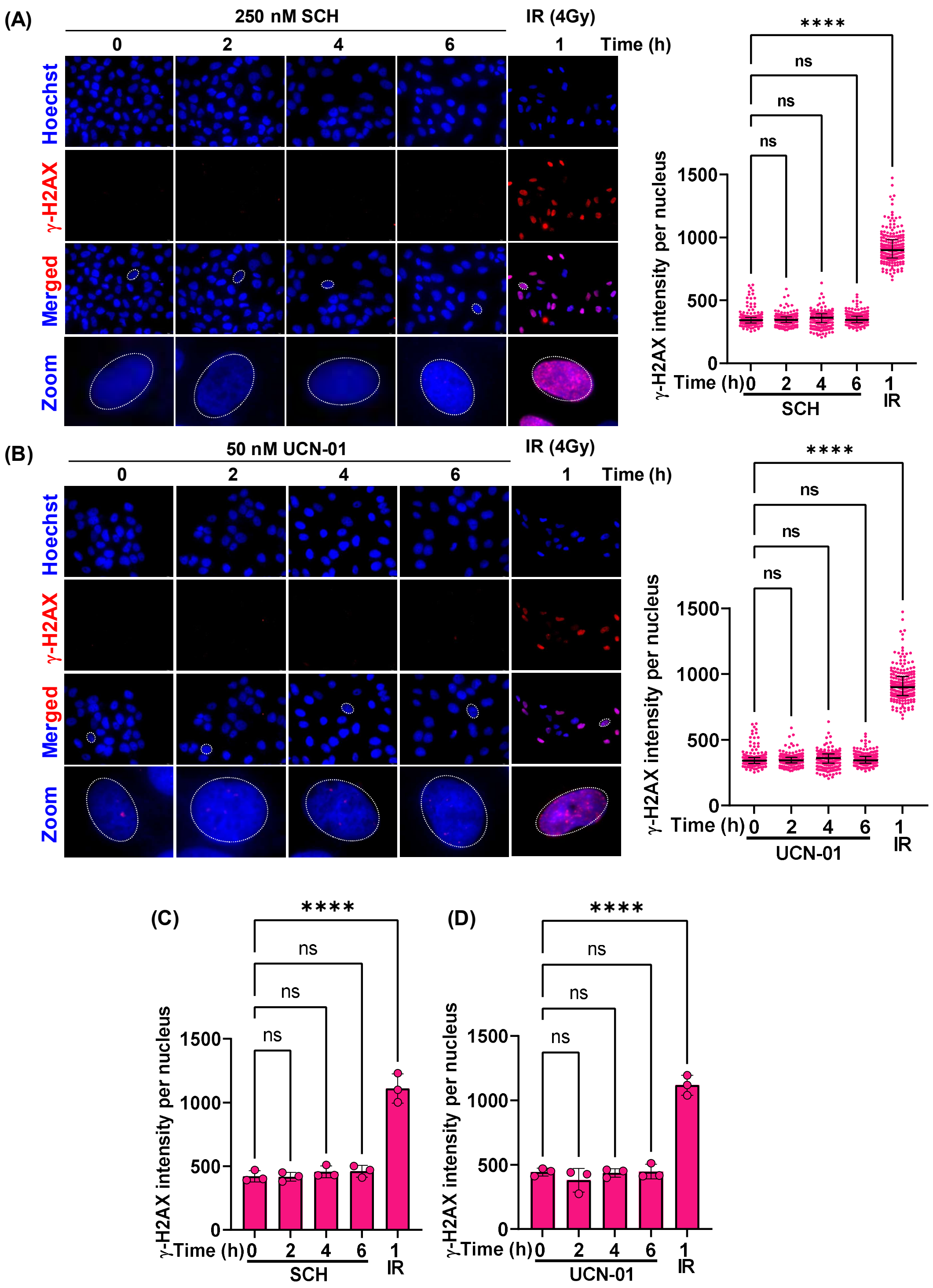
(A)
250 nM SCH
IR (4Gy)
0
2
4
6
1
Time (h)
Hoechst
γ-H2AX
Merged
Zoom
****
ns
ns
ns
1500
1000
500
0
γ-H2AX intensity per nucleus
Time (h)
SCH
IR
(B)
50 nM UCN-01
IR (4Gy)
Time (h)
UCN-01
(C)
(D)

Appendix Figure S4. Analysis of DNA damage status of cells exposed to CHK1 inhibition.

(A) Representative microscopic images (and quantification of the shown replicate) showing γ-H2AX levels in U2-OS cells treated with SCH (250 nM) for 2-,4- or 6 h. (B) Representative microscopic images (and quantification of the shown replicate) showing γ-H2AX levels in U2-OS cells treated with UCN-01 (50 nM) for 2-,4- or 6 h. In both cases, IR (4 Gy) was used as positive control. Cells were subjected to 4 Gy IR and samples were processed 1 h post irradiation. (C,D) Quantification of all replicates of (A) and (B) respectively. Data from three independent experiments with 200 cells per condition. Error bars represent interquartile range (A, B) or SEM (C, D). ns: not significant, **** $P<0.0001$ (Kruskal Wallis test with Dunn's post-hoc analysis). Scale bars = 10 μm.

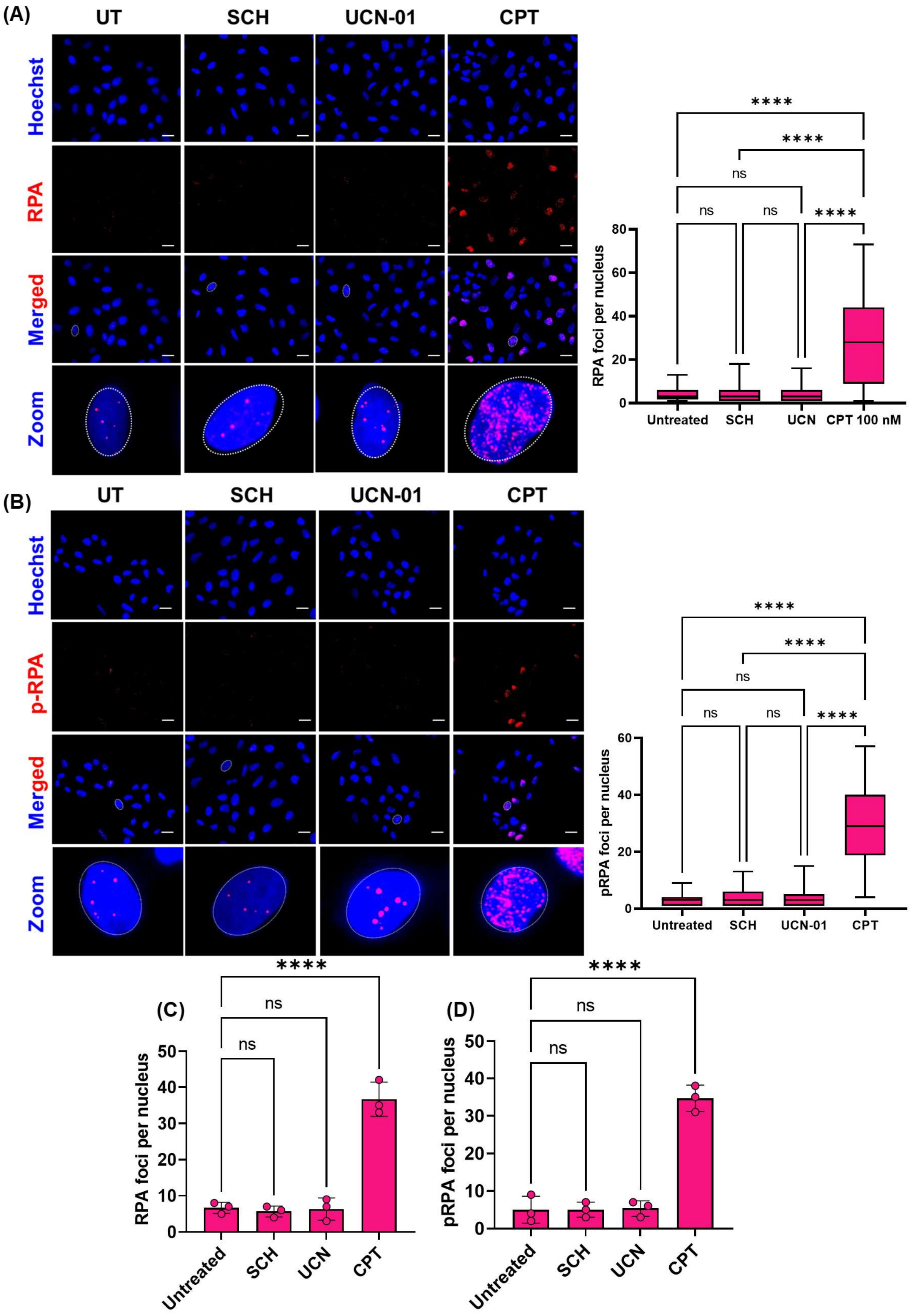
(A)
UT
SCH
UCN-01
CPT
Hoechst
RPA
Merged
Zoom
RPA foci per nucleus
Untreated
SCH
UCN
CPT 100 nM
ns
****
(B)
p-RPA
pRPA foci per nucleus
UCN-01
CPT
(C)
(D)

Appendix Figure S5. Single stranded DNA accumulation in cells exposed to CHK1 inhibition.

(A) Representative microscopic images (and quantification of the same experiment) showing RPA-32 foci in U2-OS cells treated with SCH (250 nM) or UCN-01 (50 nM) for 2 h or CPT (100 nM) for 1 h (B) Representative microscopic images (and quantification of the same experiment) showing RPA-32 foci in U2-OS cells treated with SCH (250 nM) or UCN-01 (50 nM) for 2 h or CPT (100 nM) for 1 hour. (C, D) Quantification of all replicates of (A) and (B), respectively. Data from three independent experiments with 200 cells per condition. ns: not significant. Error bars represent maximum and minimum values (A, B) or SEM (C, D). **** $P<0.0001$ (Kruskal Wallis test with Dunn's post-hoc analysis for DNA). Scale bars = 10 μm.

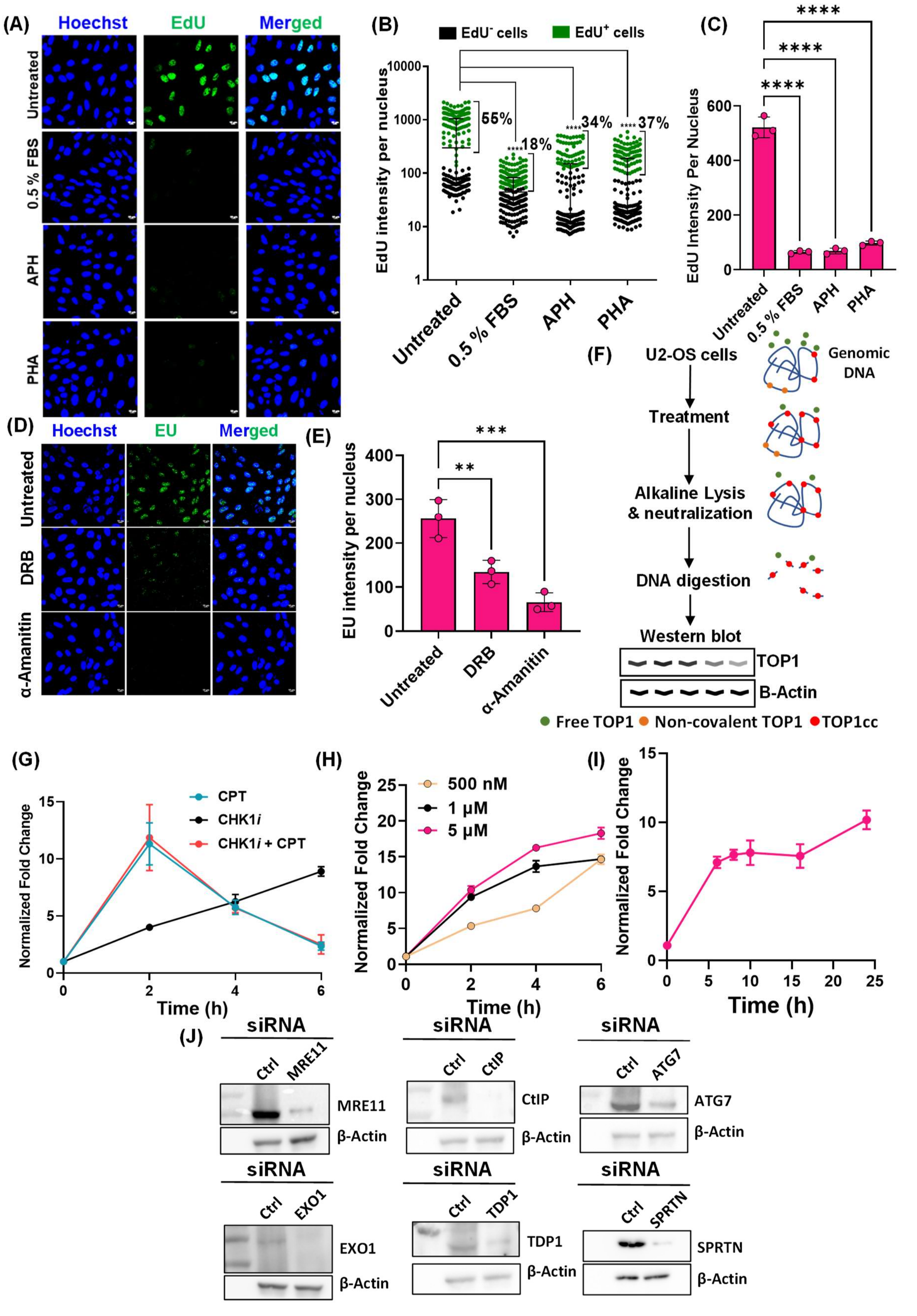

(A)
Hoechst
EdU
Merged
Untreated
0.5 % FBS
APH
PHA
(B)
EdU⁻ cells
EdU⁺ cells
EdU intensity per nucleus
10000
1000
100
10
1
55%
18%
34%
37%
****
Untreated
0.5 % FBS
APH
PHA
(C)
EdU Intensity Per Nucleus
600
400
200
0
****
Untreated
0.5 % FBS
APH
PHA
(D)
Hoechst
EU
Merged
Untreated
DRB
α-Amanitin
(E)
EU intensity per nucleus
400
300
200
100
0
**
***
Untreated
DRB
α-Amanitin
(F)
U2-OS cells
Genomic DNA
Treatment
Alkaline Lysis & neutralization
DNA digestion
Western blot
TOP1
B-Actin
Free TOP1
Non-covalent TOP1
TOP1cc
(G)
Normalized Fold Change
CPT
CHK1i
CHK1i + CPT
Time (h)
(H)
Normalized Fold Change
500 nM
1 µM
5 µM
Time (h)
(I)
Normalized Fold Change
Time (h)
(J)
siRNA
Ctrl
MRE11
β-Actin
CtIP
ATG7
EXO1
TDP1
SPRTN

Appendix Figure S6. Characterization of CHK1i-induced stabilization of TOP1ccs (contribution of replication and transcription, and targeting by cellular machinery).

(A, B) Representative microscopic images (and quantification of the shown replicate) of EdU incorporation in U2-OS cells under untreated, serum starvation (0.5 % serum *i.e.,* FBS for 48 h), aphidicolin (APH; 250 nM, 16 h) or CDC7 inhibitor (PHA; 5 µM, 4 h) conditions. U2-OS cells were subjected to requisite treatments, followed by incubation with EdU (10 µM) for 20 min prior to termination of experiment. EdU incorporation was measured using Click chemistry. Percentage of EdU positive cells is indicated against each sample. (C) Quantification of data from three replicates of the experiment shown in (A). (D, E) Representative microscopic images (and quantification across three replicates) of EU incorporation in cells treated with the transcription inhibitors DRB and α-Amanitin. U2-OS cells were treated with DRB (100 µM) for 4 h or α-Amanitin (40 µg/mL) for 16 h, prior to incubation with EU (1 mM) for 1 h, followed by termination of experiment. EU incorporation was visualized employing click chemistry. (F) Schematic representation of TOP1 downregulation assay (alkaline lysis method) of assessing global TOP1 downregulation. (G) Densitometric quantification of Fig 3B. (H, I) Densitometric quantification of RADAR assay blots shown in Fig 3E, F. (J) Immunoblots showing siRNA mediated depletion of the indicated proteins. All experiments were performed in three independent replicates. For microscopy, 200 cells were scored per condition. Scale bars: 10 µm. Error bars represent SEM. **** $P<0.0001$ (Kruskal Wallis test with Dunn's post-hoc analysis).

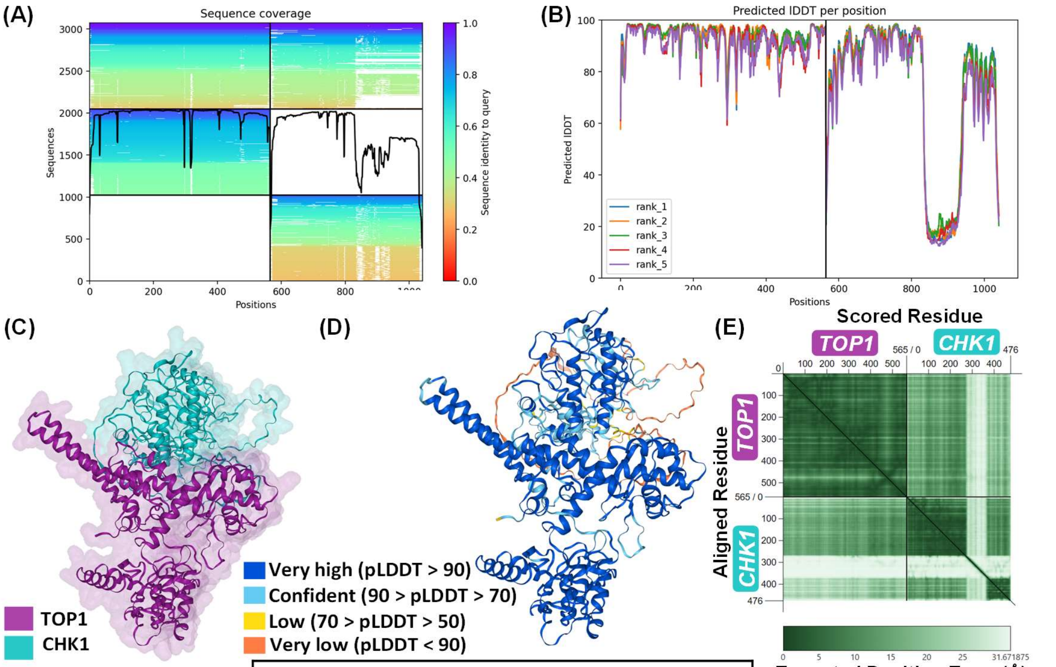

(F)

Confidence Metrics		
Mean pLDDT	pTM	ipTM
84.0622	0.7200	0.6100

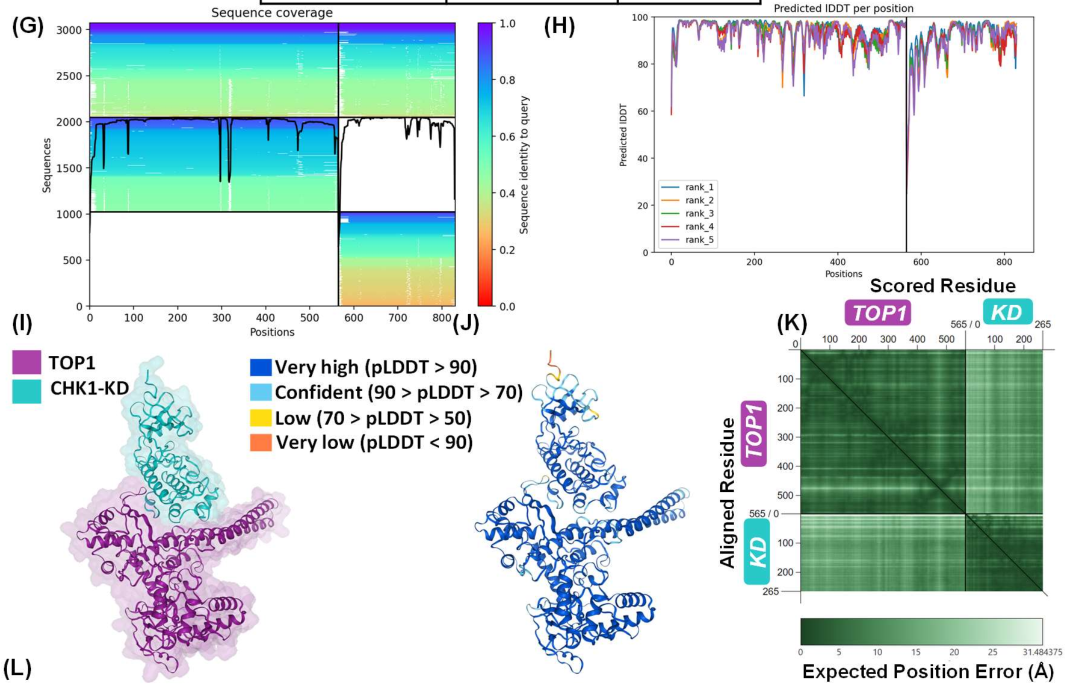

(L)

Confidence Metrics					
Mean pLDDT	pTM	ipTM	SPOC Score	pDOCKQ	Mean PAE
94.2633	0.8200	0.6400	0.414	0.349	7.5

Appendix Figure S7. *In silico* analysis of CHK1-TOP1 interaction.

Sequence coverage (A) and pLDDT (B) plots of 5 models for TOP1-CHK1 interaction produced by Alphafold Multimer V3. The top ranked model has been reproduced in Figure 2. The second ranked model has been shown in (C) and (D). (E, F) PAE plot and confidence metrics of the second ranked model. Sequence coverage (G) and pLDDT (H) plots of 5 models for interaction between TOP1 and CHK1 kinase domain (KD) produced by Alphafold Multimer V3. (I, J) Visualization of the top-ranked model. (K, L) PAE plot and confidence metrics of the top ranked model.

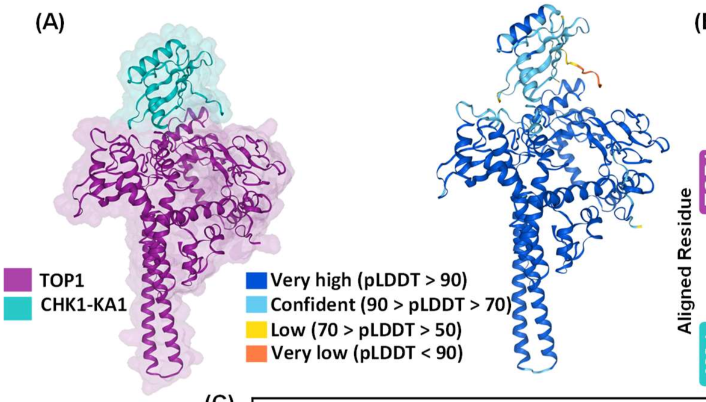

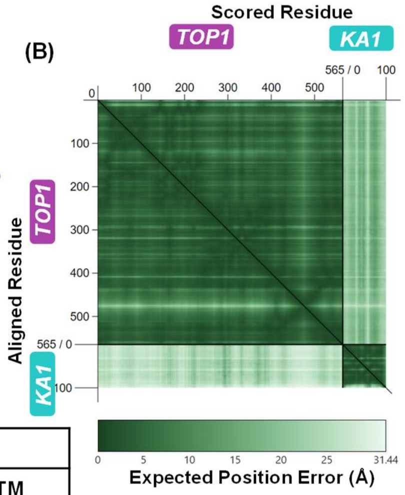

(C)

Confidence Metrics		
Mean pLDDT	pTM	ipTM
93.2553	0.8300	0.3400

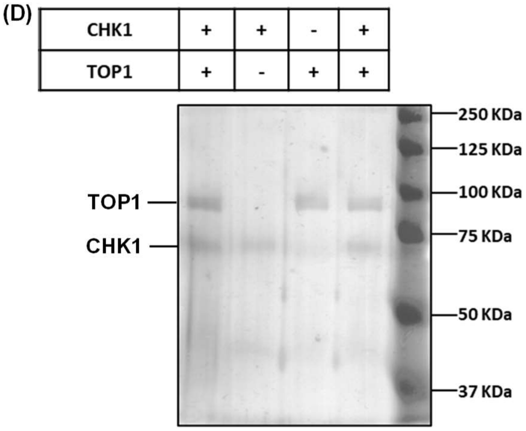

Appendix Figure S8. Characterization of TOP1-CHK1 interaction.

Visualization (A) and PAE plot (B) of the best ranked model for interaction between TOP1 and CHK1 KA1 domain produced by Alphafold Multimer V3. (C) Confidence metrics of the prediction. (D) Silver-stained gel corresponding to the *in vitro* kinase assay reaction. Parallel gels were run for autoradiography and silver stain.

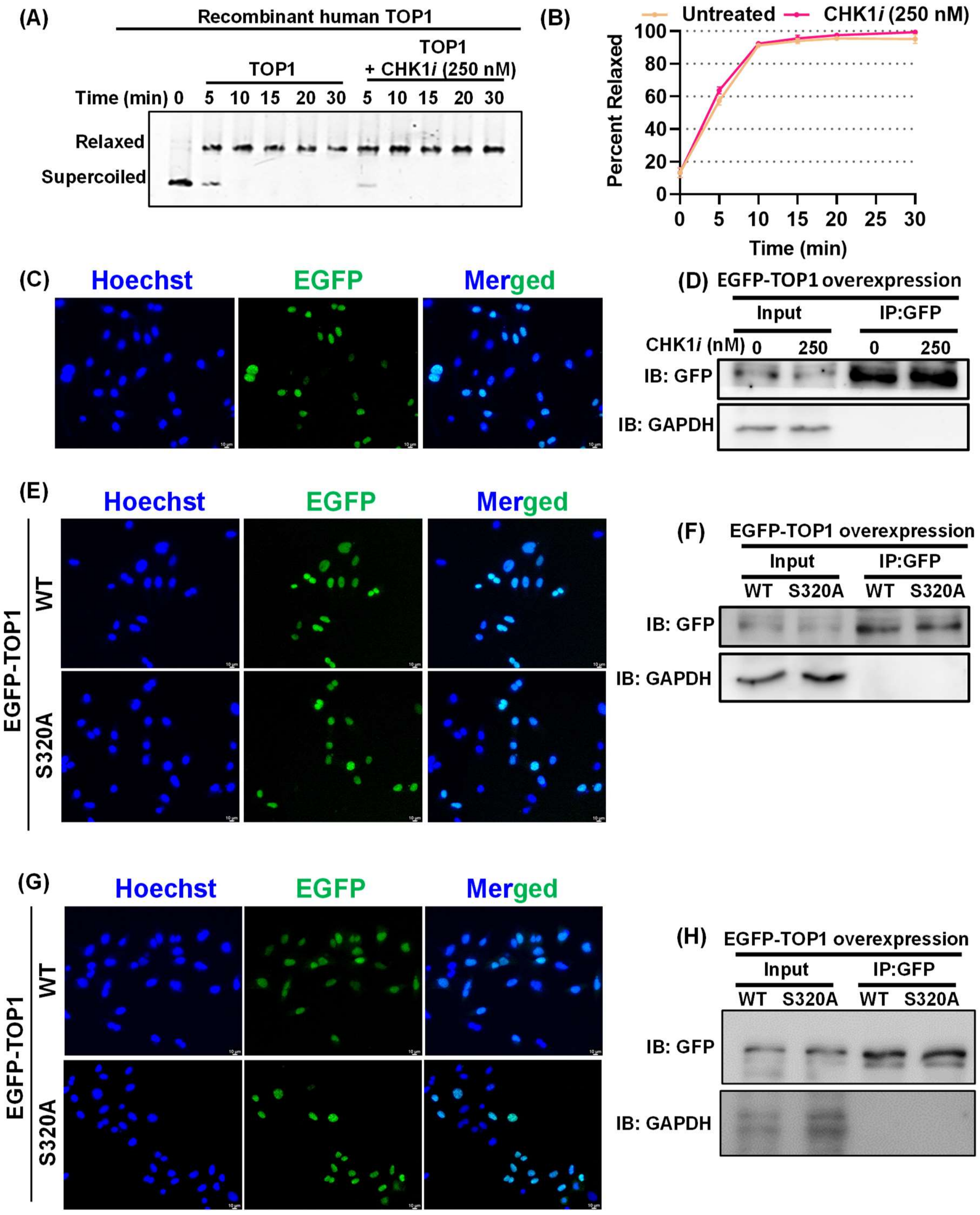
(A)
Recombinant human TOP1
TOP1
TOP1 + CHK1*i* (250 nM)
Time (min) 0 5 10 15 20 30 5 10 15 20 30
Relaxed
Supercoiled
(B)
Untreated
CHK1*i* (250 nM)
Percent Relaxed
Time (min)
0 20 40 60 80 100
0 5 10 15 20 25 30
(C)
Hoechst
EGFP
Merged
(D) EGFP-TOP1 overexpression
Input
IP:GFP
CHK1*i* (nM) 0 250 0 250
IB: GFP
IB: GAPDH
(E)
Hoechst
EGFP
Merged
EGFP-TOP1
WT
S320A
(F) EGFP-TOP1 overexpression
Input
IP:GFP
WT S320A WT S320A
IB: GFP
IB: GAPDH
(G)
Hoechst
EGFP
Merged
EGFP-TOP1
WT
S320A
(H) EGFP-TOP1 overexpression
Input
IP:GFP
WT S320A WT S320A
IB: GFP
IB: GAPDH

Appendix Figure S9. Characterization of expression levels and immunoprecipitation efficiencies of EGFP-TOP1^{WT} and EGFP-TOP1^{S320A}.

(A, B) Plasmid relaxation assay with recombinant human TOP1 in the absence or presence of CHK1*i* (250 nM). Data from two independent replicates. (C) Representative live-cell microscopy images showing transfection efficiency pertaining to the experiment shown in Fig. 6C. (D) Immunoprecipitation efficiency of EGFP-TOP1^{WT} from U2-OS cells under untreated or CHK1*i*-treated conditions. (E) Representative live-cell microscopy images showing transfection efficiency pertaining to the experiment shown in Fig. 6E. (F) Immunoprecipitation efficiency of EGFP-TOP1^{WT} and EGFP-TOP1^{S320A} from U2-OS cells. (G) Representative live-cell microscopy images showing transfection efficiency pertaining to the experiment shown in Fig. 6H, J. (H) Immunoprecipitation efficiency of EGFP-TOP1^{WT} and EGFP-TOP1^{S320A} from U2-OS cells. These immunocomplexes were employed for TOP1 cleavage and religation assays. N=3. Error bars represent SEM.

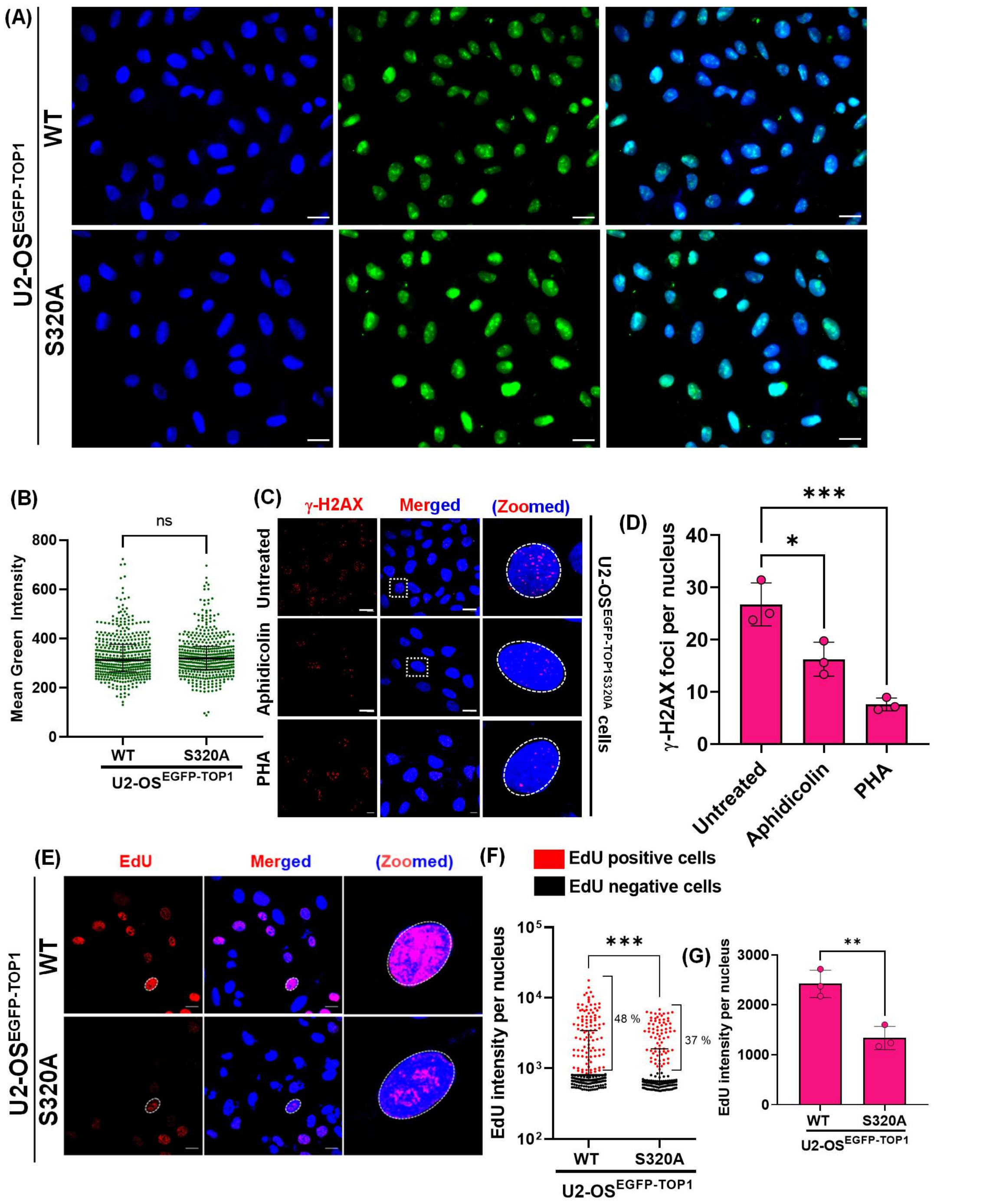

Appendix Figure S10. Replication-dependent DNA damage and replication perturbations associated with EGFP-TOP1^{S320A} expression.

(A, B) Representative live cell fluorescence microscopy images (and quantification) showing expression of EGFP-TOP1^{WT} and EGFP-TOP1^{S320A} in U2-OS$^{EGFP-TOP1WT}$ and U2-OS^{EGFP-}

^{TOP1S320A} cells, respectively. Data from the shown replicate with 500 cells per condition. (C, D) Representative microscopic images (and quantification across tree replicates) of γ-H2AX levels in U2-OS$^{EGFP\text{-}TOP1S320A}$ cells treated with replication inhibitors Aphidicolin or CDC7*i* (PHA). Cells were treated with aphidicolin (200 nM) for 16 h or PHA (5 μM) for 6 h before immunofluorescent detection of γ-H2AX foci. Data from three independent experiments with 200 cells per condition. (E-G) Representative microscopic images (and quantification of the shown replicate, as well as data from three replicates) of EdU incorporation assay in U2-OS$^{EGFP\text{-}TOP1WT}$ and U2-OS$^{EGFP\text{-}TOP1S320A}$ cells. Cells were labelled with EdU (10 μM) for 30 min, followed by detection through click chemistry. Scale bars 10 μm. Data from three independent experiments with 200 cells per condition. Error bars represent interquartile range (B, F) or SEM (D, G). * $P<0.05$, ** $P<0.01$; **** $P<0.0001$ (Kruskal Wallis test with Dunn's post-hoc analysis).

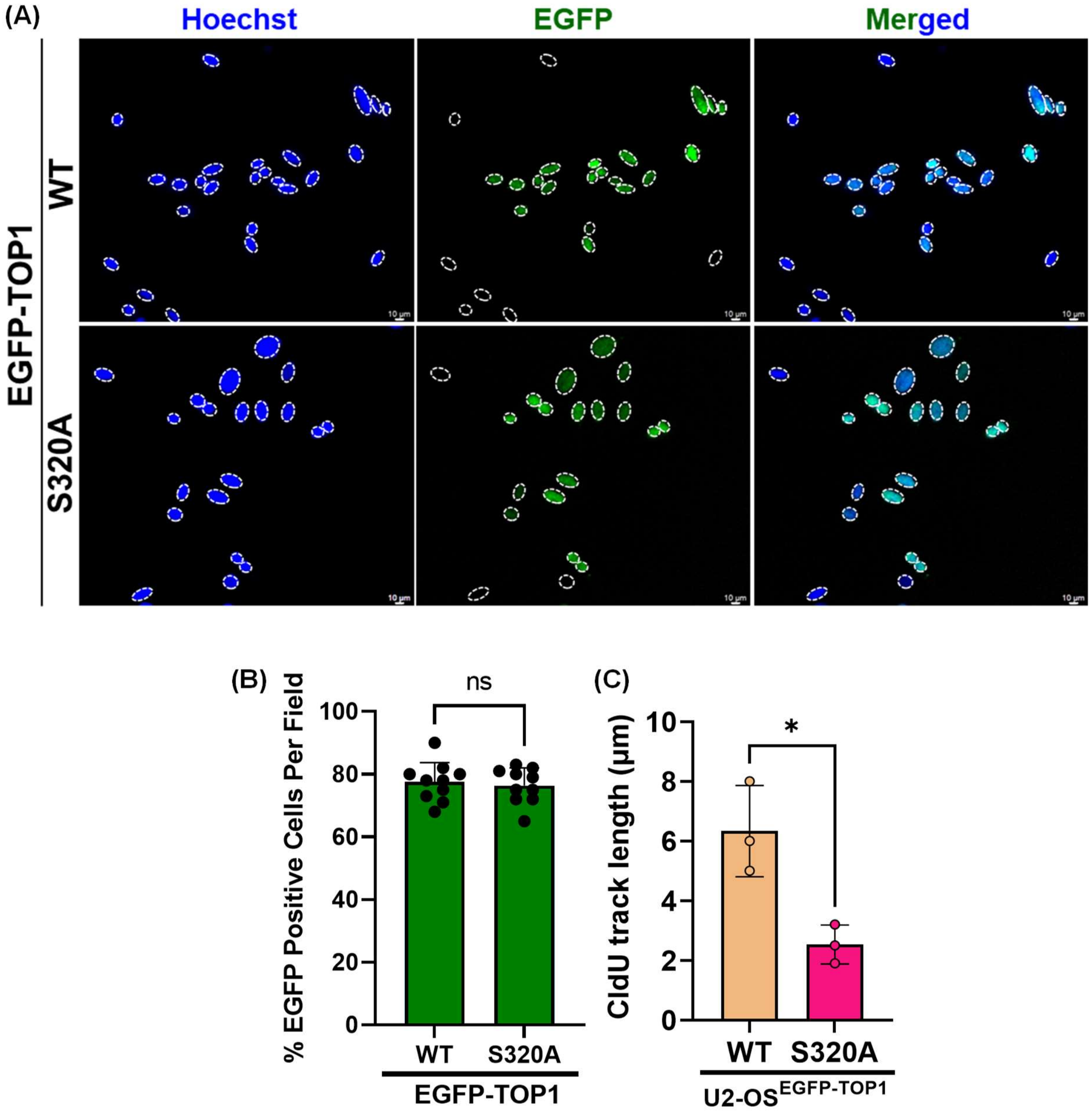

Appendix Figure S11. Transfection efficiency of EGFP-TOP1^{WT} and EGFP-TOP1^{S320A}.

(A) Representative live-cell microscopy images showing transfection efficiency pertaining to the experiment shown in Figure 7G. (B) Quantification of experiment shown in (A) across 10 fields. (C) Quantification across all replicates pertaining to experiment shown in Fig.7G. Error bars represent SEM. ns: not significant, $P<0.05$ (Kruskal Wallis test with Dunn's post-hoc analysis).

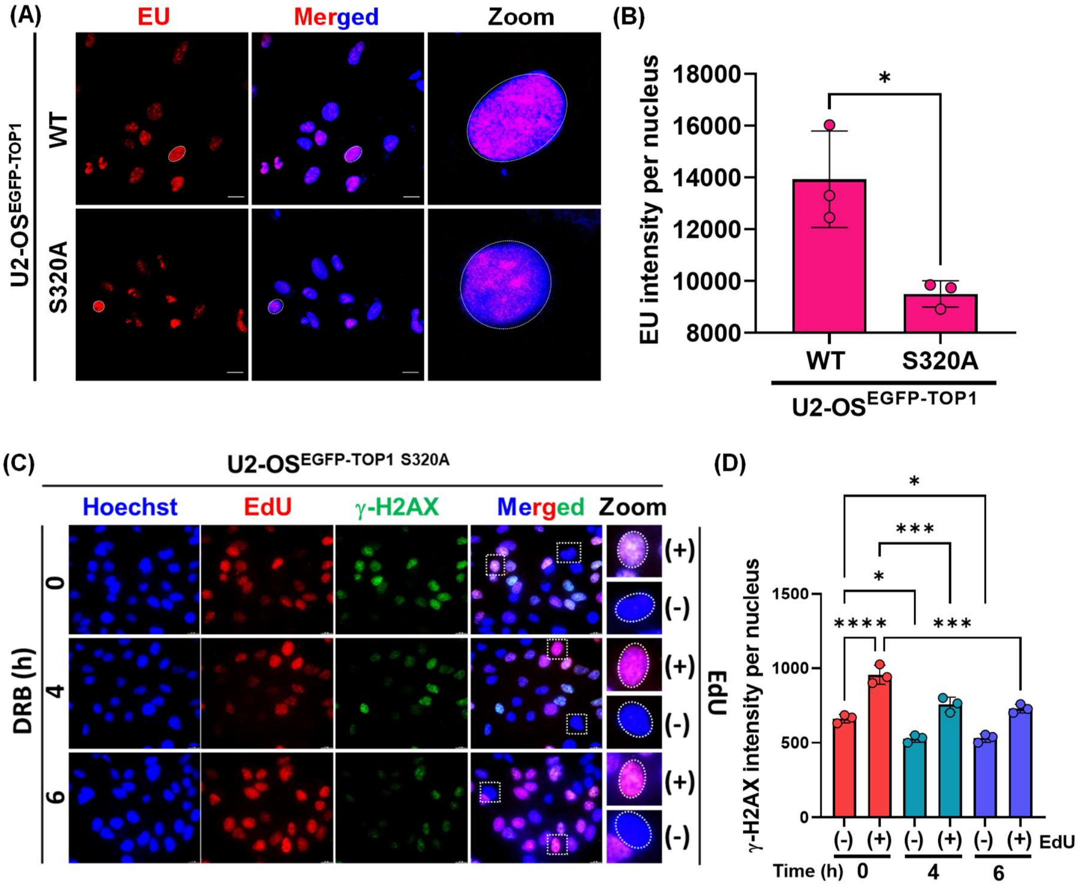

Appendix Figure S12. Characterization of transcription-associated DNA damage in cells expressing EGFP-TOP1^{S320A}.

(A, B) Representative microscopic images (and quantification across three replicates) of EU incorporation assay in U2-OS$^{EGFP\text{-}TOP1WT}$ and U2-OS$^{EGFP\text{-}TOP1S320A}$ cells. Cells were labelled with EU (1 mM) for 1 h, followed by detection through click chemistry. Data from three independent experiments with 200 cells per condition. (C, D) Representative microscopic images (and quantification across three replicates) showing EdU/ γ-H2AX dual staining in U2-OS$^{EGFP\text{-}TOP1S320A}$ cells treated with DRB (100 μM) for 4 or 6 h. Data from three independent experiments with 200 cells per condition. Scale bars 10 μm. Error bars represent SEM. *P<0.05; *** P<0.0001; **** P<0.0001 (Kruskal Wallis test with Dunn's post-hoc analysis).

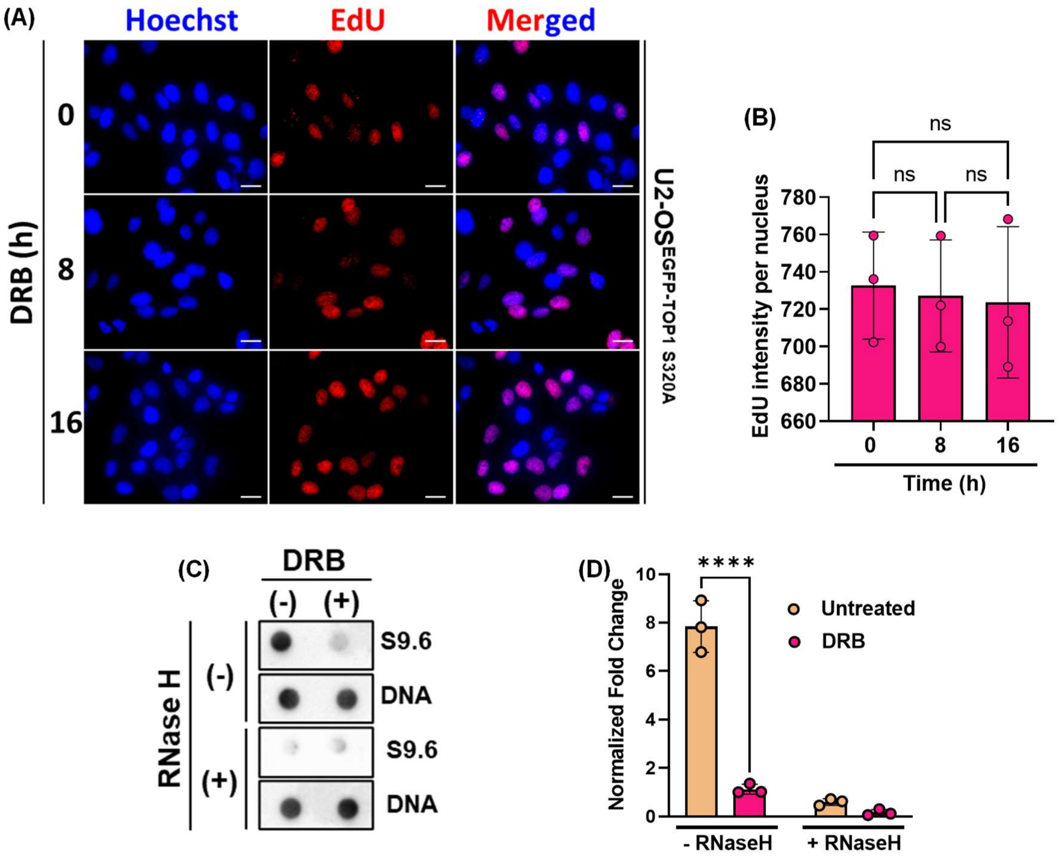

Appendix Figure S13. Transcription-associated genomic instability in cells expressing EGFP-TOP1S320A.

(A, B) Representative microscopic images (and quantification across three replicates) of EdU incorporation levels in U2-OS$^{EGFP\text{-}TOP1S320A}$ cells upon treatment with DRB (20 µM) with 8 or 16 h. U2-OS$^{EGFP\text{-}TOP1S320A}$ cells were treated with DRB (20 µM) for 8 or 16 h, followed by labelling with EdU (10 µM) for 20 min prior to termination of experiment. Data from three independent experiments with 200 cells per condition. (C, D) Dot blot showing R-loop levels in U2-OS$^{EGFP\text{-}TOP1S320A}$ cells treated with DRB (20 µM) for 16 h. Data from three independent experiments with 200 cells per condition. Scale bars 10 µm. Error bars represent SEM. ns: not significant, P<0.05; **** P<0.0001 (Kruskal Wallis test with Dunn's post-hoc analysis).

INHIBITOR	TARGET KINASE (S)	C1 (µM)	C2 (µM)	C3 (µM)
KU-55933	ATM	2.5	5	10
PV1019	CHK2	2.5	5	10
AZD6738	ATR	0.5	1	2.5
SCH900776	CHK1	0.1	0.25	0.5
NU7026	DNAPK	2.5	5	10
Volasertib (BI627)	PLK1, PLK2, PLK3	0.025	0.05	0.1
Wortmannin	PI3K	0.5	1	2.5
RO-3306	CDK1	1 1	2.5	5
SP600125	JNK	5	10	20
U0126	MEK1, MEK2	2.5	5	10
Danusertib	Aurora A, Aurora B, Aurora C	0.025	0.05	0.1
KN-93	CAMK	5	10	20
LJH685	RSK1, RSK2, RSK3	1	2.5	5
PHA-767491	Cdc7	2.5	5	10
GSK2606414	PERK	0.25	0.5	1
MRT68921	ULK1, ULK2	0.25	0.5	1
SB203580	p38	2.5	5	10
ARQ-092	Akt1, Akt2, Akt3	2.5	5	10
Go6983	PKCα, PKCβ, PKCγ, PKCδ, PKCζ, PKCµ	2.5	5	10
CX-4945	CK2	2.5	5	10
Brigatinib (AP26113)	ALK	0.025	0.05	0.1
MK1775	Wee1	0.05	0.1	0.2
Compound C	AMPK	5	10	20
Torin1	mTORC1	0.1	0.25	0.5
Roscovitine	CDK2, CDK7, CDK9	5	10	20

Appendix Table S1: Kinase inhibitors, their targets, and concentrations employed in the RADAR screen.

Minimal Sequence

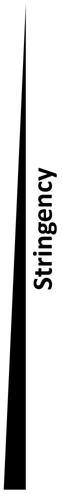

Full Sequence

Query Motif	Hits	
	Position	Sequence
[RK]-x-x-[ST]	44 – 47	KDREKSKH
	64 - 67	KEKEKTKH
	70 – 73	KHKDGSSE
	151 – 154	PKKIKTED
	284 – 287	WRKEMTNE
	310 – 313	YFKAQTEA
	317 – 320	ARKQMSKE
	391 – 394	DAKVPSPP
	443 – 446	WQKYETAR
	567 – 570	FDRLNTGI
	603 – 606	QLKELTAP
	615 – 618	PAKILSYN
[KRHV]-[RK]-x-x-[ST]	69 – 73	TKHKDGSSE
	150 – 154	KPKKIKTED
	283 – 287	DWRKEMTNE
	316 – 320	EARKQMSKE
[RFPGAILV]-[KRHV]-[RK]-x-x-[ST]	149 – 154	YKPKKIKTED
	315 – 320	TEARKQMSKE

Appendix Table S2: Prediction of CHK1 target motifs on TOP1. While the entire motif is highlighted in green, the target residue is depicted in red.

Site	Untreated	250 nM SCH 2h	250 nM SCH 6h	200 nM CPT 2h	1 µM CPT 2h	Previously Detected?
S320	+	+	+	+	+	No
S394	+	+	-	-	+	Yes
T570	+	-	-	+	-	Yes
Y231	+	-	-	-	-	No
S250	+	-	-	+	-	No
Y480	+	-	-	+	-	Yes
T446	-	+	-	-	-	No
Y461	-	-	+	+	-	No
S534	-	-	-	+	-	No
Y538	-	-	-	+	-	No
T706	-	-	-	+	-	Yes

Appendix Table S3: Details of sites found to be phosphorylated on catalytically active TOP1. Whether they have previously been reported elsewhere is also indicated. References pertaining to these studies are provided in the main text**.**

	Forward Primer	Reverse Primer
**MYO3A TSS**	GTCAGATCCGGAGGACC	GTTCATCCCTCTCCTCCC
**MYO3A Gene Body**	CTACAGCAGCCCACTCAAG	CATTATCCAGTTTCTTGATTCATG
**β-Actin TSS**	CGGGGTCTTTGTCTGAGC	CAGTTAGCGCCCAAAGGAC
**β-Actin Gene Body**	GGAGCTGTCACATCCAGGGTC	TGCTGATCCACATCTGCTGG
**g-Actin TSS**	CCGCAGTGCAGACTTCCGAG	CGGGCGCGTCTGTAACACGG
**g-Actin Gene Body**	GTGACACAGCATCACTAAGG	ACAGCACCGTGTTGGCGT
**PTB Gene Body**	GCCGTTGGTACAAAGGTAGG	GCCCCTTAGGAATGGAAAAG
**Geminin 7 Gene Body**	TCTTCTTCCACCTGGACCAC	GGGACAGAGAGAGTGCCTTG
**IL4 Gene Body**	TTCAGGTGACAAGTGCCACAG	CTGGTTGGCTTCCTTCACAG
**Intergenic**	ACCCAGCACCCCCTAATACC	AGCCGGACATGCTTCCAGAG

Appendix Table S4: Details of primers used in qPCR.
