# Supplementary figures and images for: A CHK1-mediated phosphorylation switch suppresses human Topoisomerase 1-associated genomic instability (part 1 of 3)
Source: EMBO J. 2026 May 13;45(12):4220–56. doi: 10.1038/s44318-026-00783-3 (PMC13270093; doi:10.1038/s44318-026-00783-3)

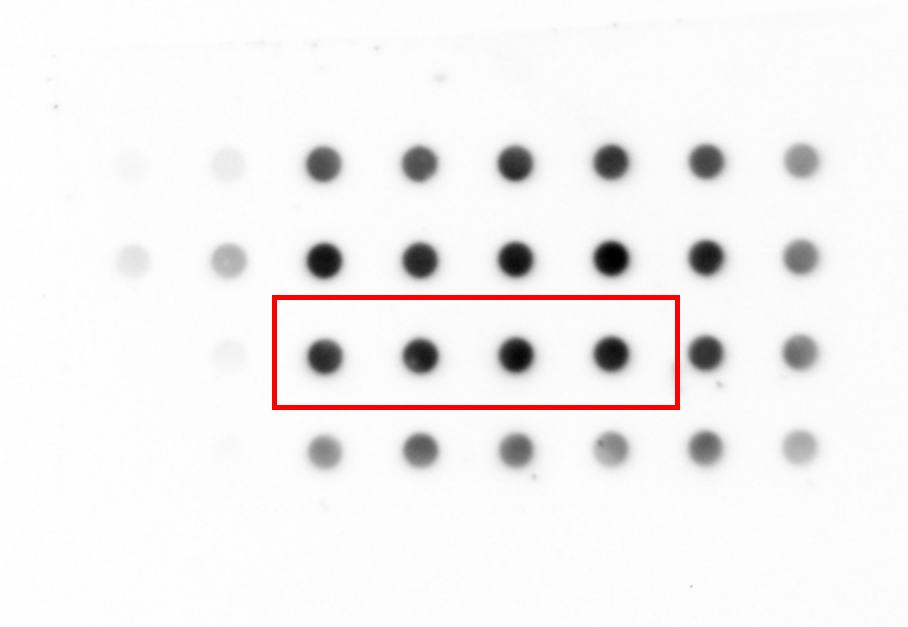

Supplement: Supplementary file 3 — Source data Fig. 1 [file 44318_2026_783_MOESM3_ESM.zip › Figure 1/Figure 1C/Fig 1C_SCH_DNA.tif]

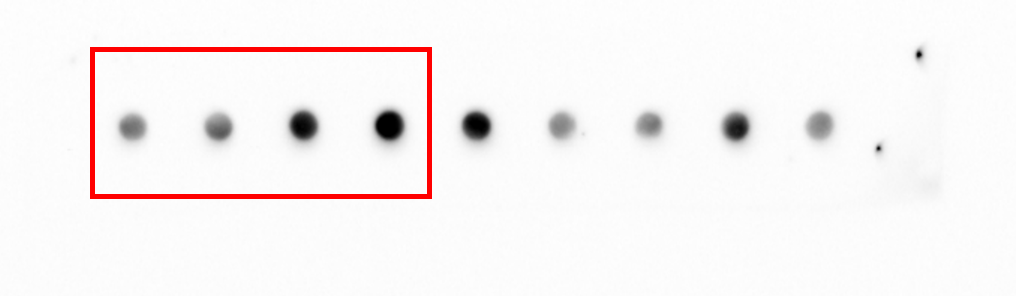

Supplement: Supplementary file 3 — Source data Fig. 1 [file 44318_2026_783_MOESM3_ESM.zip › Figure 1/Figure 1C/Fig 1C_SCH_TOP1.tif]

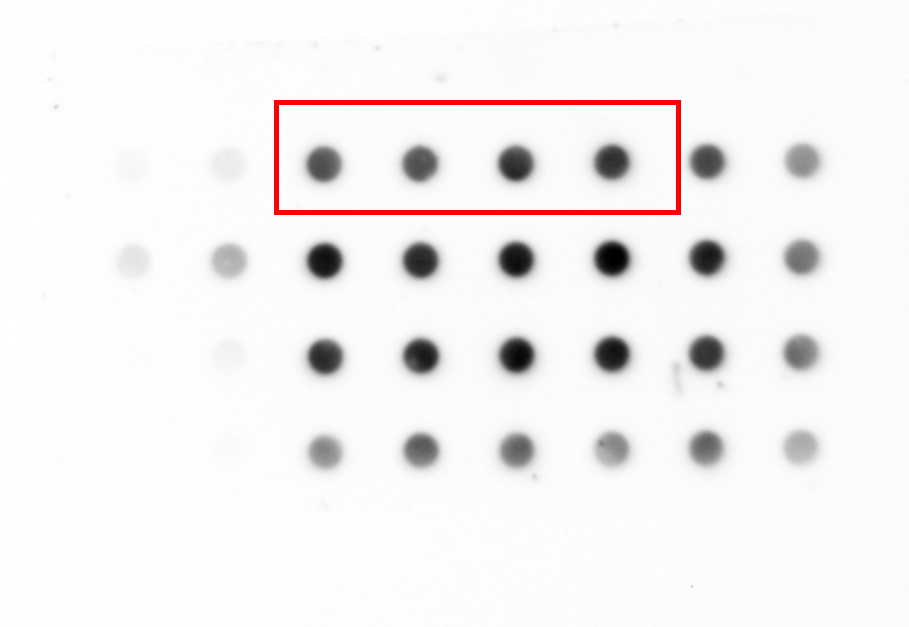

Supplement: Supplementary file 3 — Source data Fig. 1 [file 44318_2026_783_MOESM3_ESM.zip › Figure 1/Figure 1C/Fig 1C_UCN01_DNA.tif]

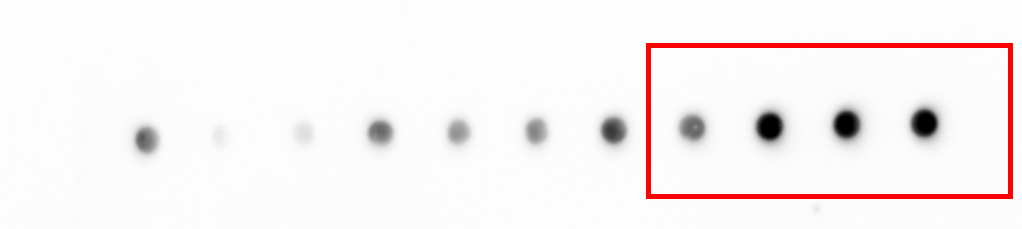

Supplement: Supplementary file 3 — Source data Fig. 1 [file 44318_2026_783_MOESM3_ESM.zip › Figure 1/Figure 1C/Fig 1C_UCN01_TOP1.tif]

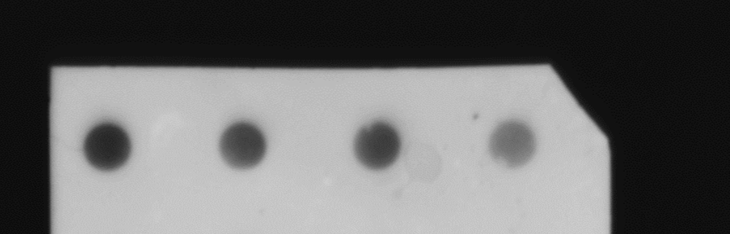

Supplement: Supplementary file 3 — Source data Fig. 1 [file 44318_2026_783_MOESM3_ESM.zip › Figure 1/Figure 1D/Fig 1D_DNA.tif]

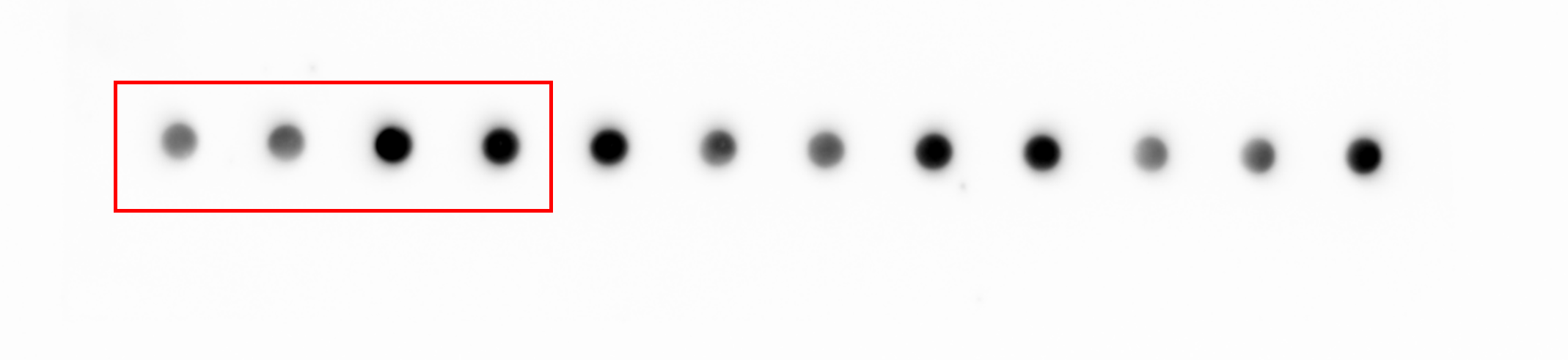

Supplement: Supplementary file 3 — Source data Fig. 1 [file 44318_2026_783_MOESM3_ESM.zip › Figure 1/Figure 1D/Fig 1D_TOP1.tif]

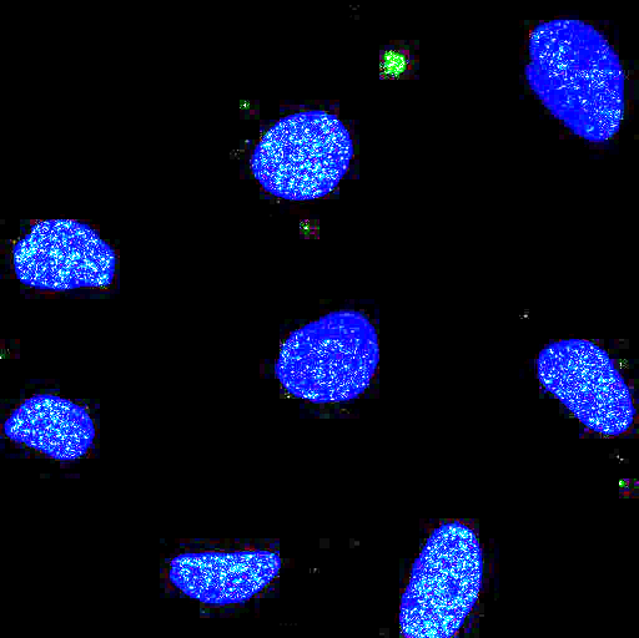

Supplement: Supplementary file 3 — Source data Fig. 1 [file 44318_2026_783_MOESM3_ESM.zip › Figure 1/Figure 1E/CPT_Merged.tif]

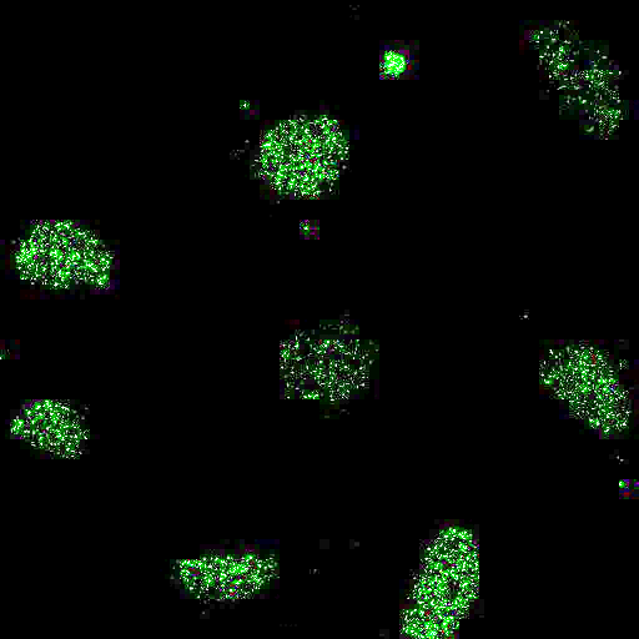

Supplement: Supplementary file 3 — Source data Fig. 1 [file 44318_2026_783_MOESM3_ESM.zip › Figure 1/Figure 1E/CPT_TOP1cc.tif]

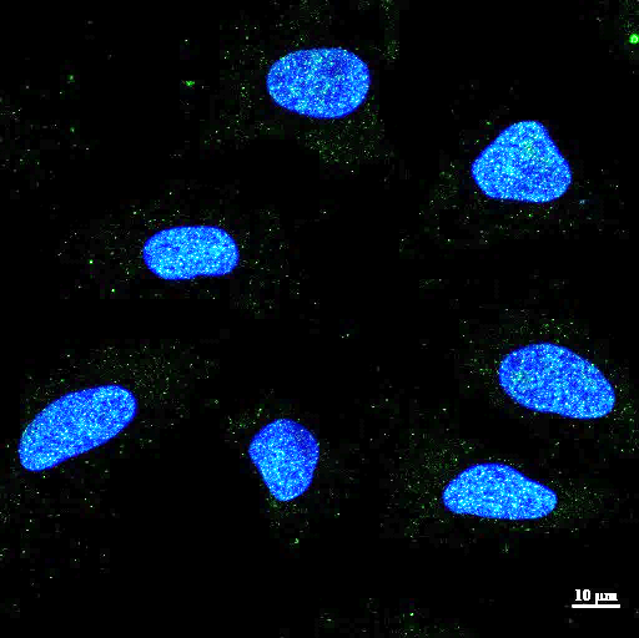

Supplement: Supplementary file 3 — Source data Fig. 1 [file 44318_2026_783_MOESM3_ESM.zip › Figure 1/Figure 1E/SCH_Merged.tif]

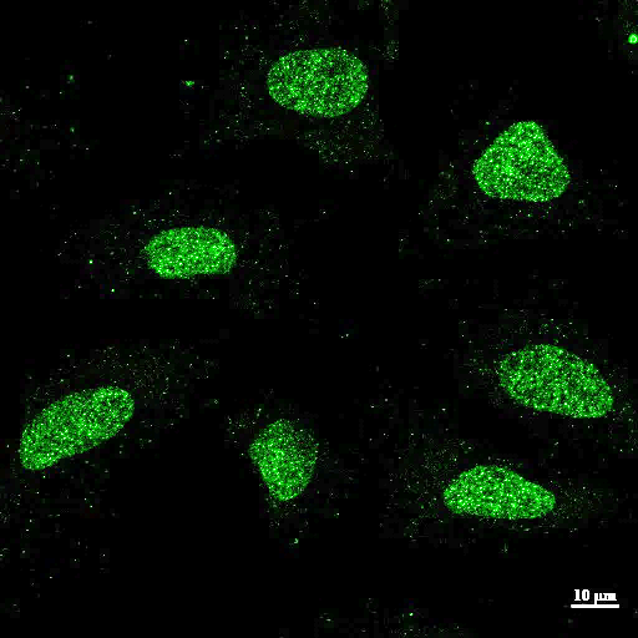

Supplement: Supplementary file 3 — Source data Fig. 1 [file 44318_2026_783_MOESM3_ESM.zip › Figure 1/Figure 1E/SCH_TOP1cc.tif]

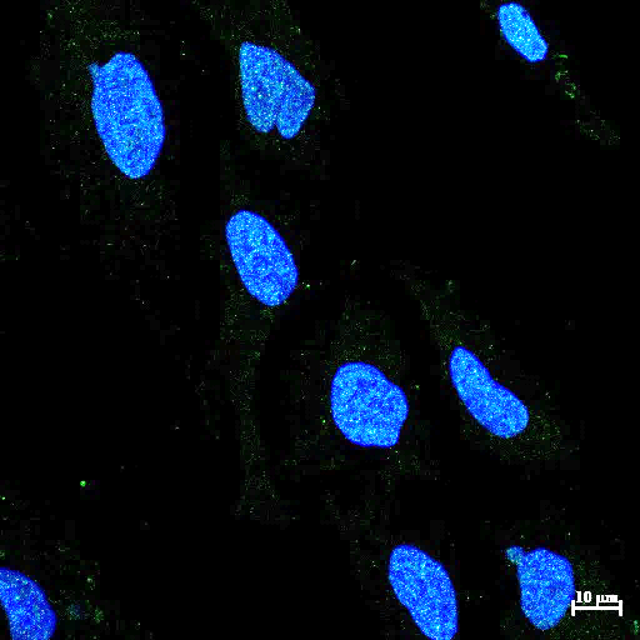

Supplement: Supplementary file 3 — Source data Fig. 1 [file 44318_2026_783_MOESM3_ESM.zip › Figure 1/Figure 1E/UCN01_Merged.tif]

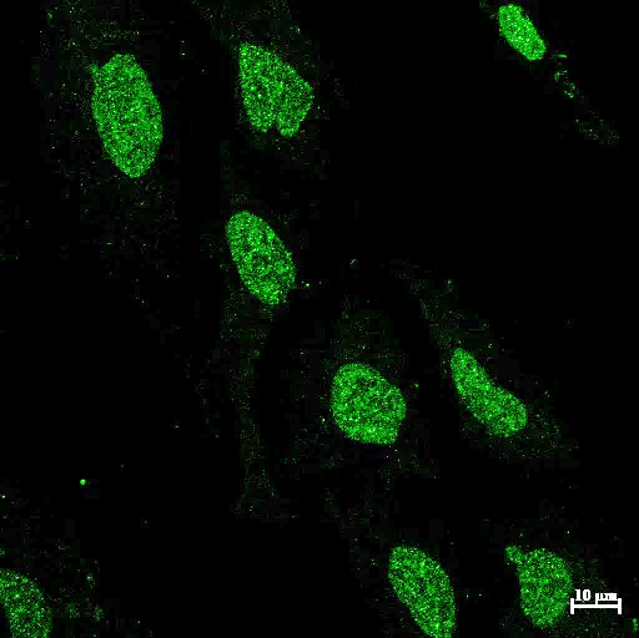

Supplement: Supplementary file 3 — Source data Fig. 1 [file 44318_2026_783_MOESM3_ESM.zip › Figure 1/Figure 1E/UCN01_TOP1cc.tif]

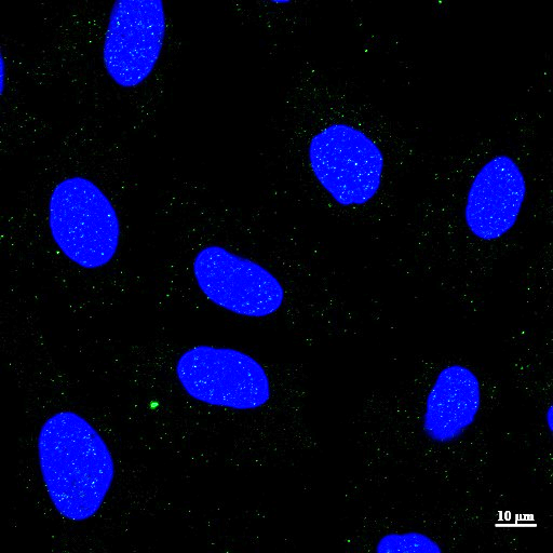

Supplement: Supplementary file 3 — Source data Fig. 1 [file 44318_2026_783_MOESM3_ESM.zip › Figure 1/Figure 1E/Untreated_Merged.tif]

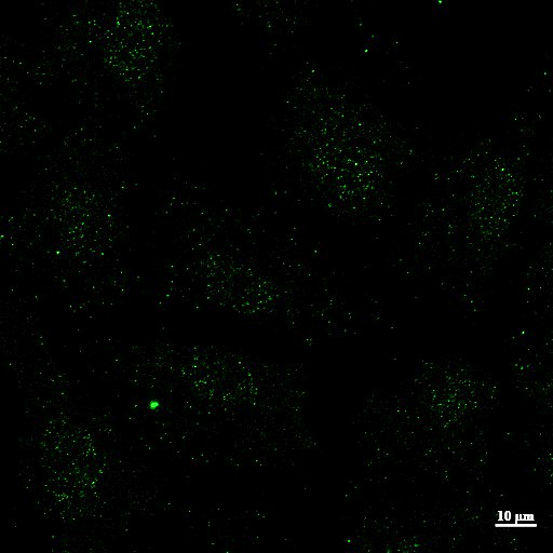

Supplement: Supplementary file 3 — Source data Fig. 1 [file 44318_2026_783_MOESM3_ESM.zip › Figure 1/Figure 1E/Untreated_TOP1cc.tif]

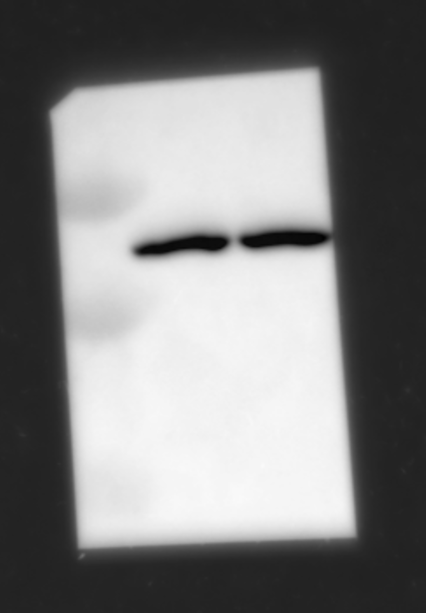

Supplement: Supplementary file 3 — Source data Fig. 1 [file 44318_2026_783_MOESM3_ESM.zip › Figure 1/Figure 1G/Beta Actin.tif]

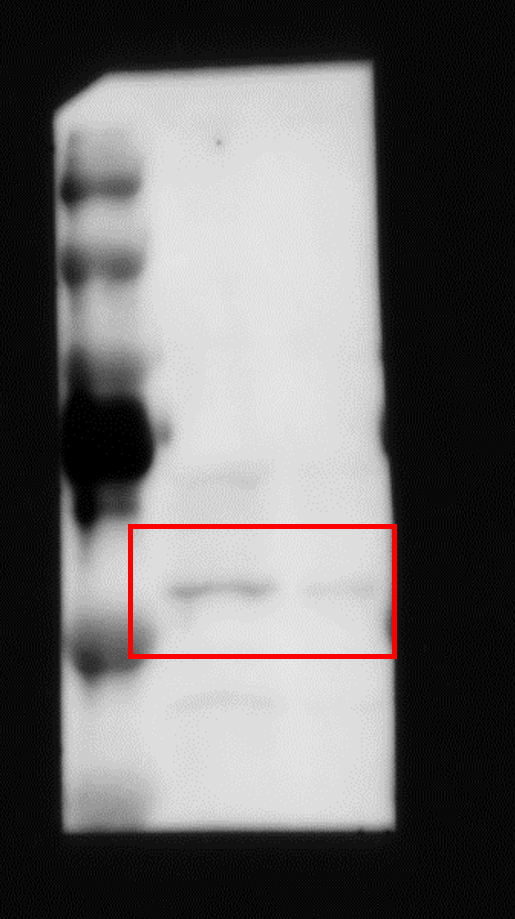

Supplement: Supplementary file 3 — Source data Fig. 1 [file 44318_2026_783_MOESM3_ESM.zip › Figure 1/Figure 1G/CHK1.tif]

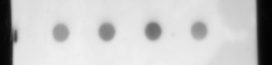

Supplement: Supplementary file 3 — Source data Fig. 1 [file 44318_2026_783_MOESM3_ESM.zip › Figure 1/Figure 1H/ATRi_DNA.tif]

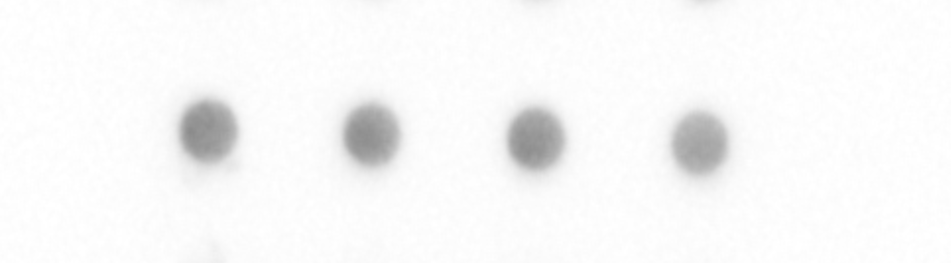

Supplement: Supplementary file 3 — Source data Fig. 1 [file 44318_2026_783_MOESM3_ESM.zip › Figure 1/Figure 1H/ATRi_TOP1.tif]

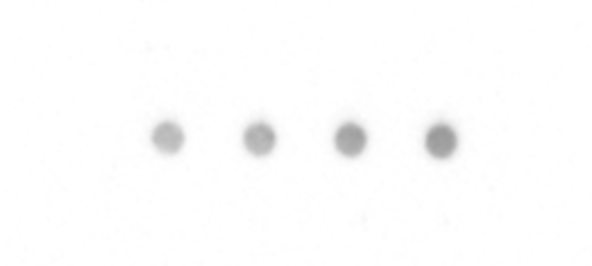

Supplement: Supplementary file 3 — Source data Fig. 1 [file 44318_2026_783_MOESM3_ESM.zip › Figure 1/Figure 1H/Figure 1H siCtrl_DNA.tif]

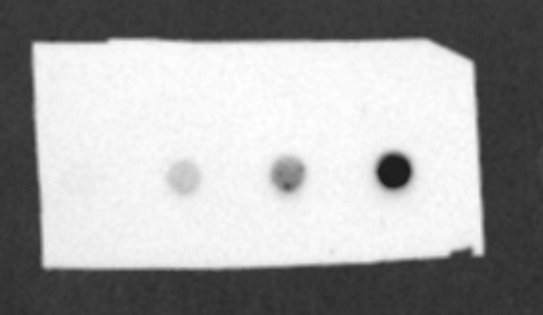

Supplement: Supplementary file 3 — Source data Fig. 1 [file 44318_2026_783_MOESM3_ESM.zip › Figure 1/Figure 1H/Figure 1H siCtrl_TOP1.tif]

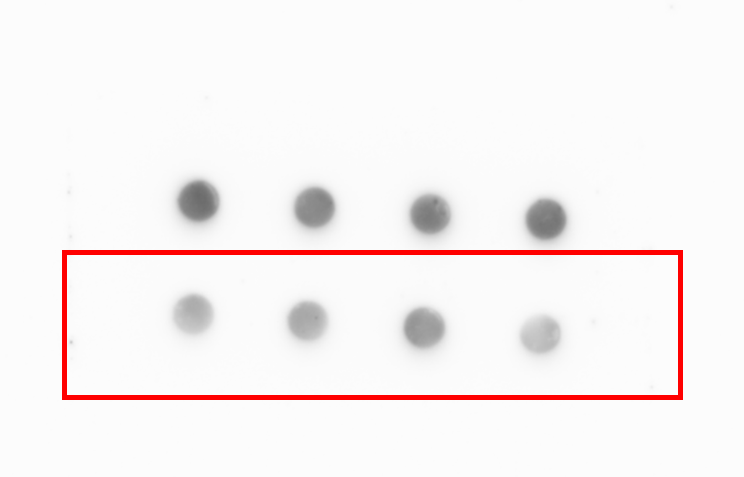

Supplement: Supplementary file 3 — Source data Fig. 1 [file 44318_2026_783_MOESM3_ESM.zip › Figure 1/Figure 1H/siCHK1_DNA.tif]

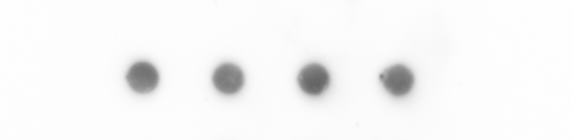

Supplement: Supplementary file 3 — Source data Fig. 1 [file 44318_2026_783_MOESM3_ESM.zip › Figure 1/Figure 1H/siCHK1_TOP1.tif]

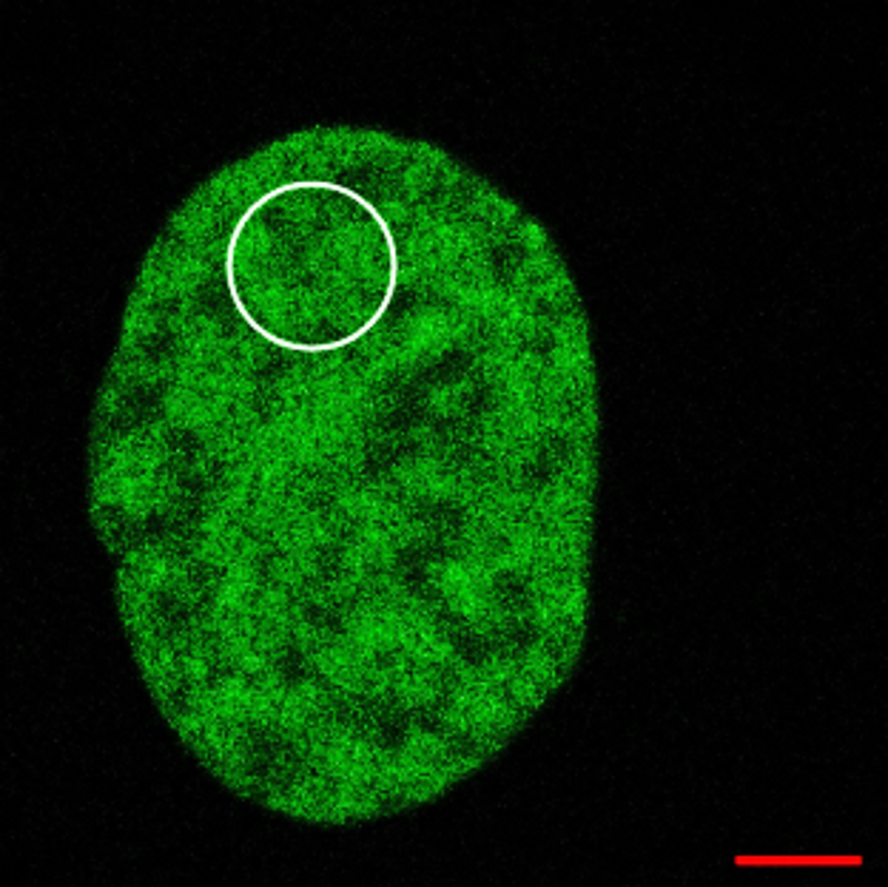

Supplement: Supplementary file 3 — Source data Fig. 1 [file 44318_2026_783_MOESM3_ESM.zip › Figure 1/Figure 1I/SCH_0S.tif]

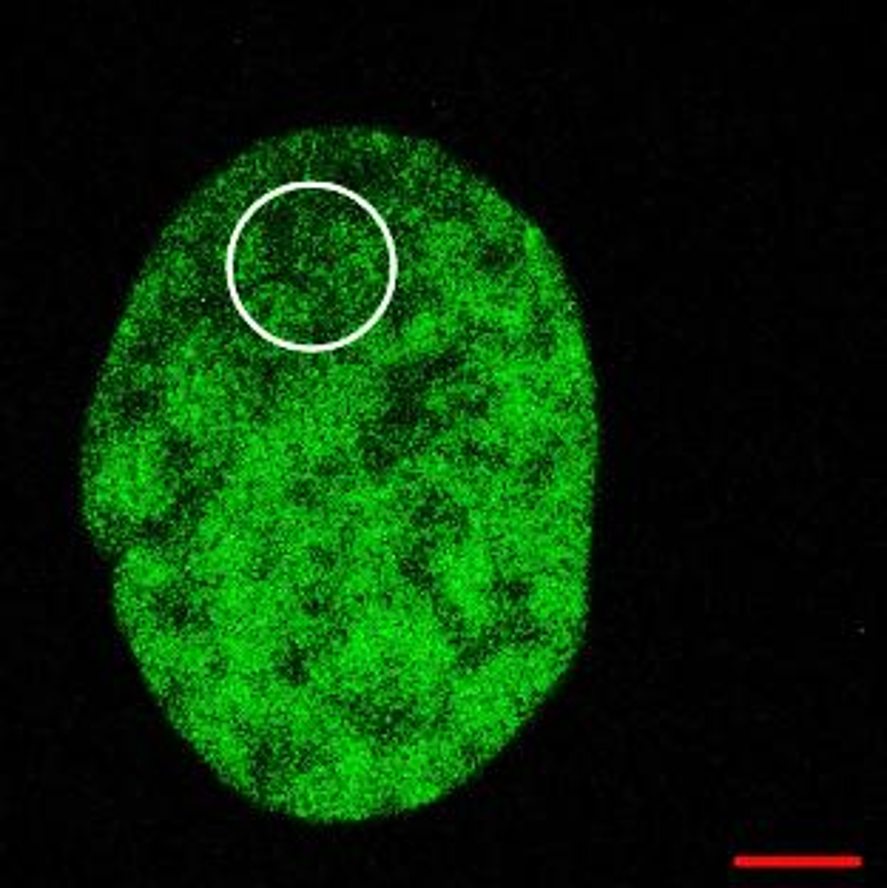

Supplement: Supplementary file 3 — Source data Fig. 1 [file 44318_2026_783_MOESM3_ESM.zip › Figure 1/Figure 1I/SCH_150S.tif]

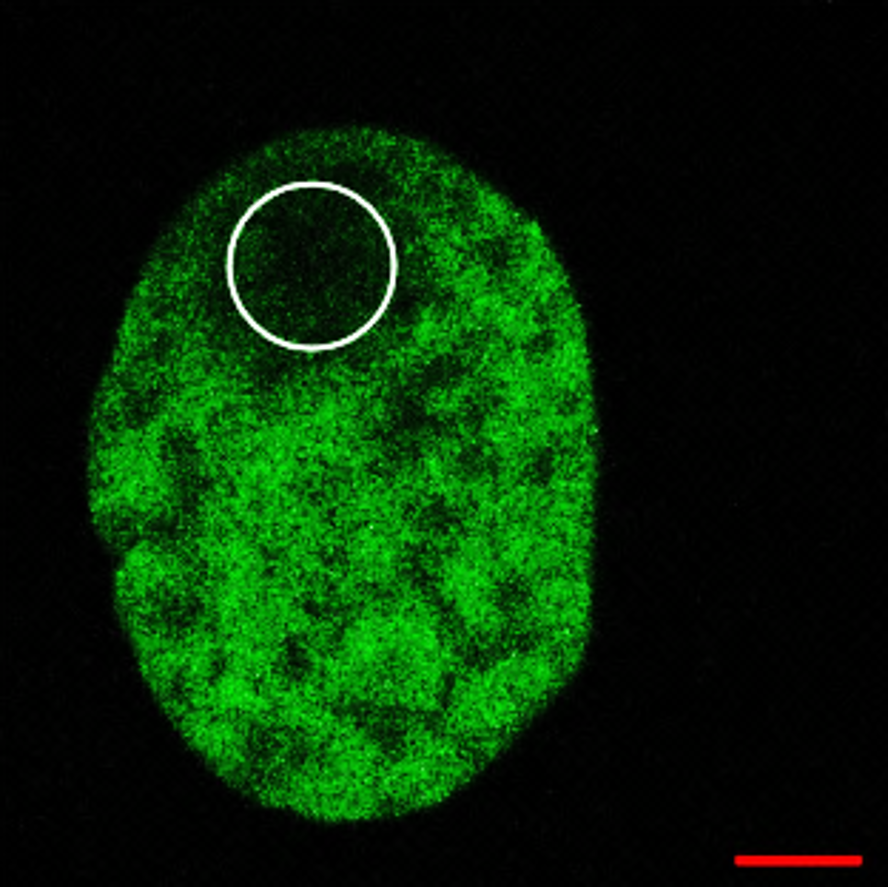

Supplement: Supplementary file 3 — Source data Fig. 1 [file 44318_2026_783_MOESM3_ESM.zip › Figure 1/Figure 1I/SCH_30S.tif]

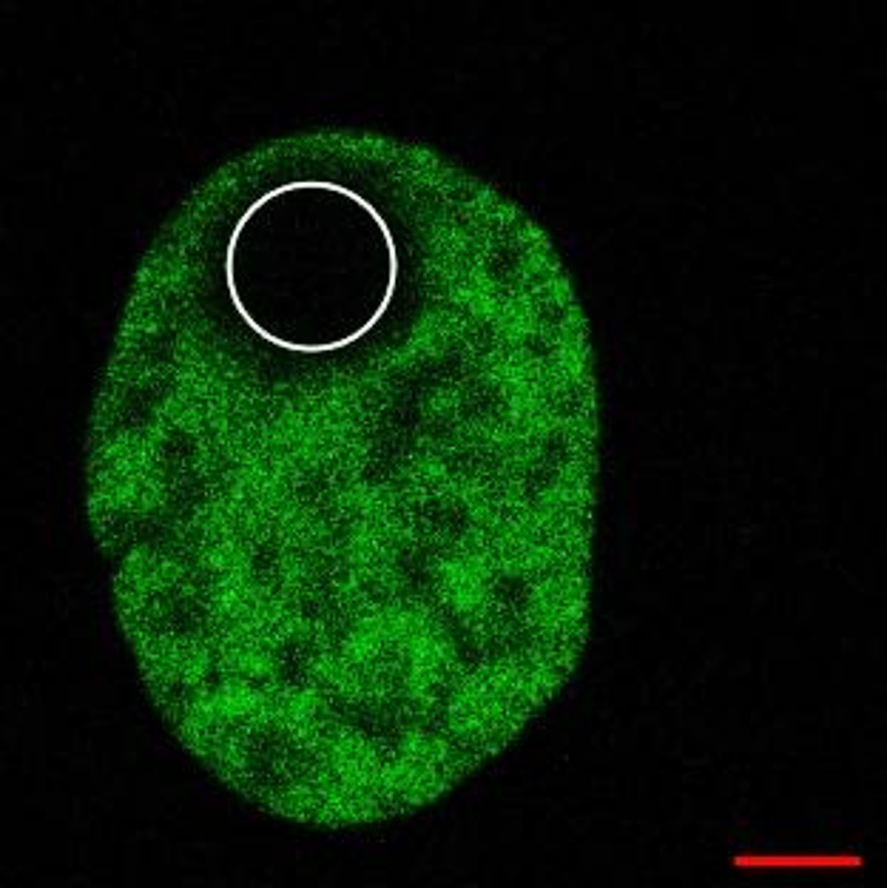

Supplement: Supplementary file 3 — Source data Fig. 1 [file 44318_2026_783_MOESM3_ESM.zip › Figure 1/Figure 1I/SCH_8S.tif]

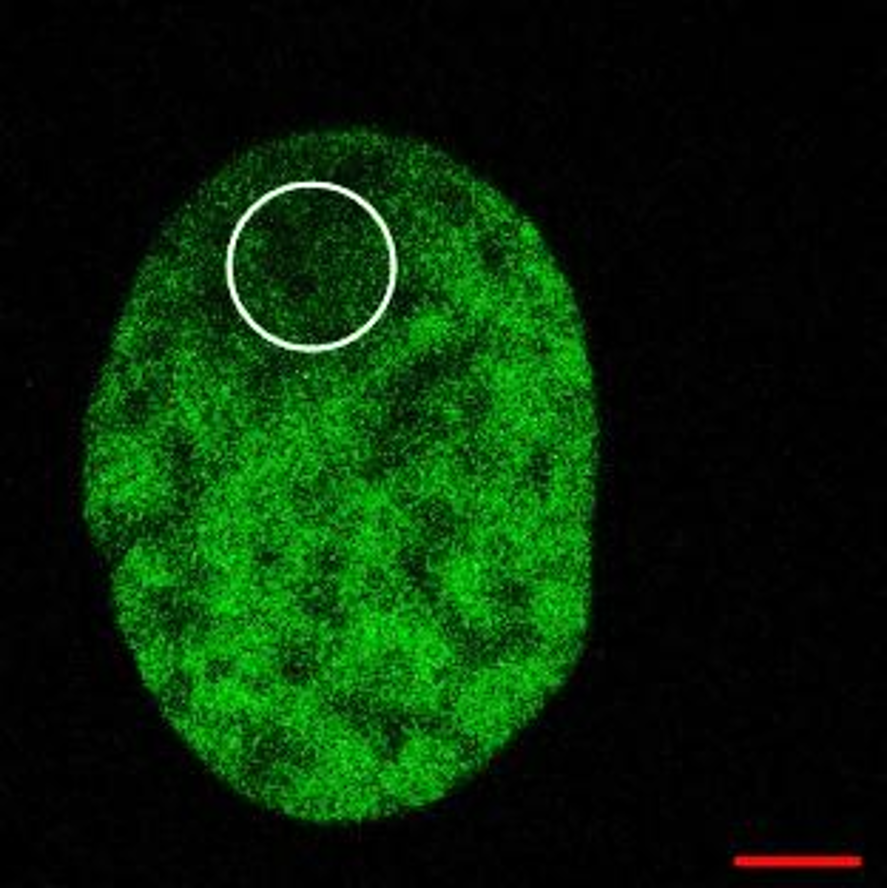

Supplement: Supplementary file 3 — Source data Fig. 1 [file 44318_2026_783_MOESM3_ESM.zip › Figure 1/Figure 1I/SCH_90S.tif]

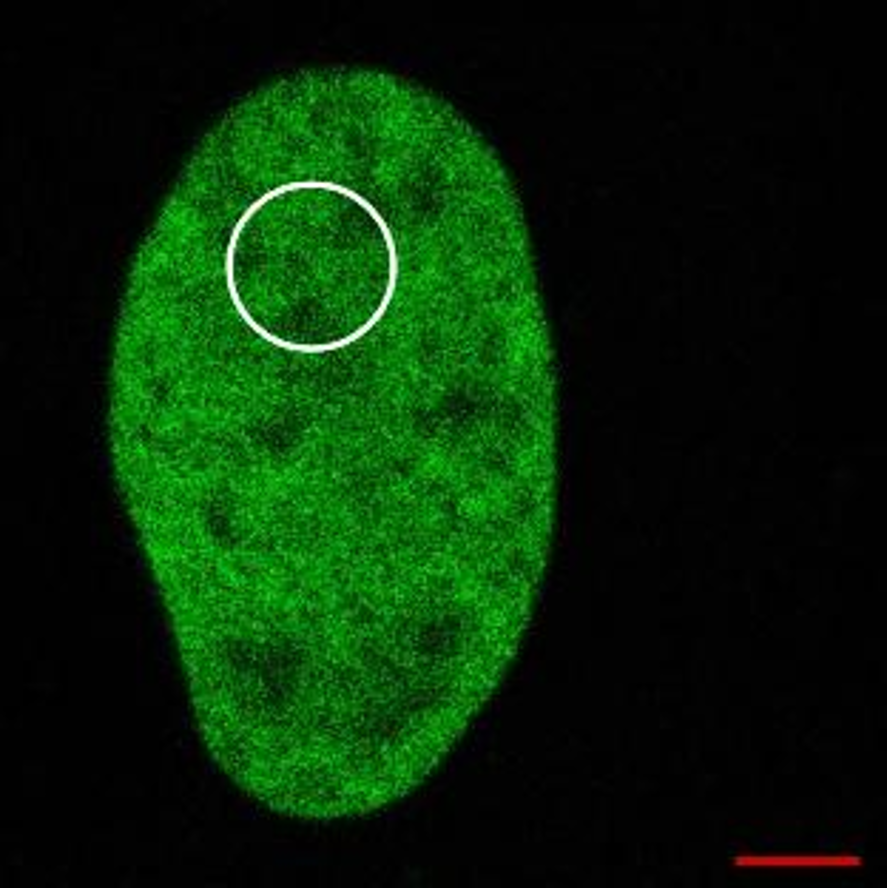

Supplement: Supplementary file 3 — Source data Fig. 1 [file 44318_2026_783_MOESM3_ESM.zip › Figure 1/Figure 1I/Untreated_0S.tif]

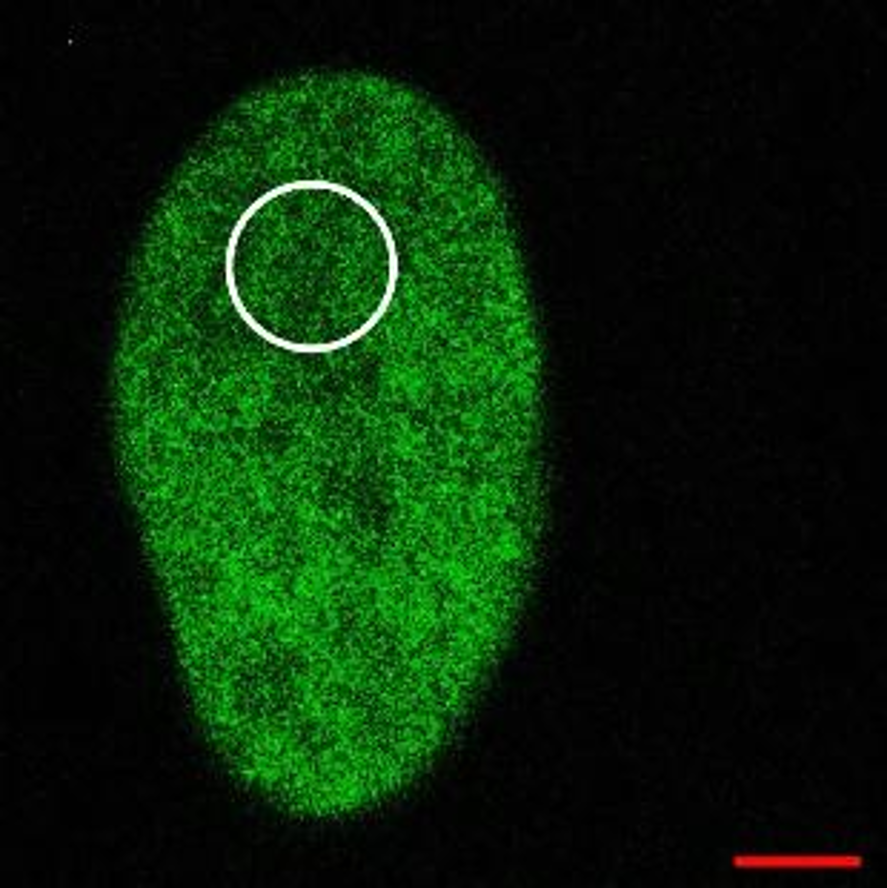

Supplement: Supplementary file 3 — Source data Fig. 1 [file 44318_2026_783_MOESM3_ESM.zip › Figure 1/Figure 1I/Untreated_150S.tif]

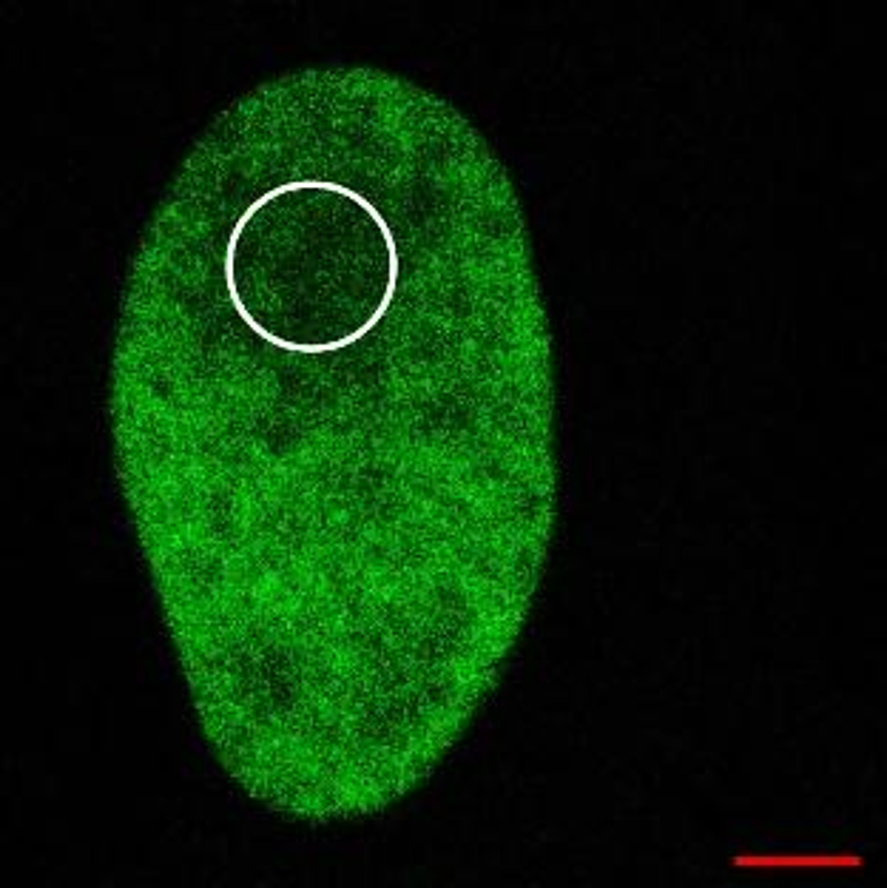

Supplement: Supplementary file 3 — Source data Fig. 1 [file 44318_2026_783_MOESM3_ESM.zip › Figure 1/Figure 1I/Untreated_30S.tif]

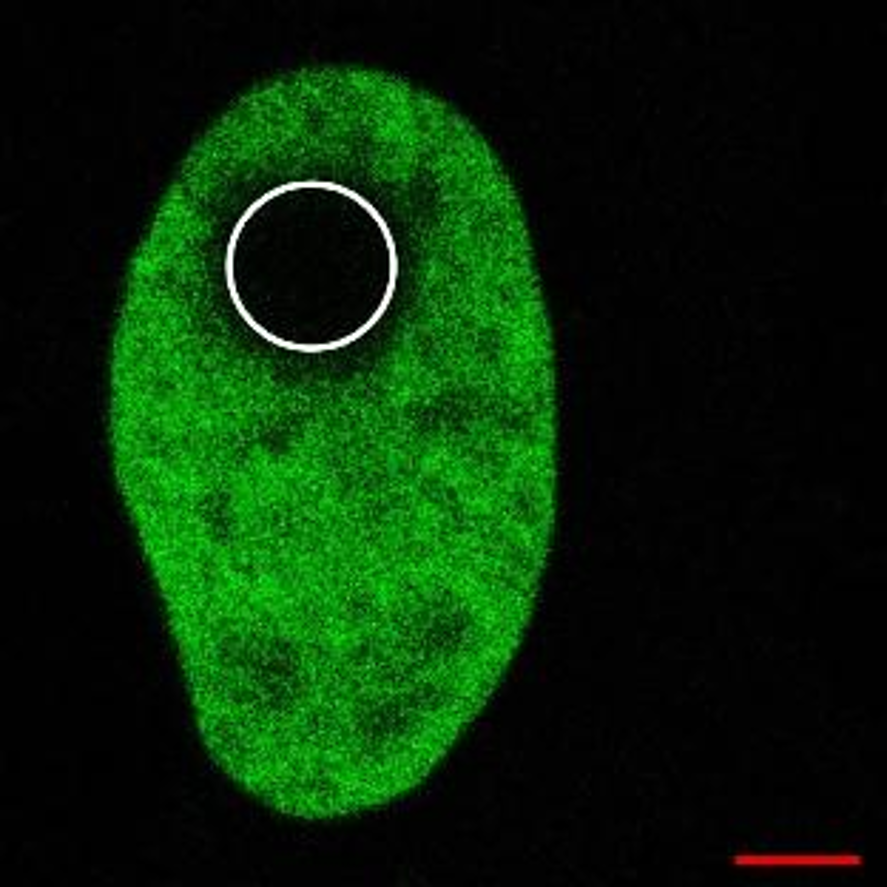

Supplement: Supplementary file 3 — Source data Fig. 1 [file 44318_2026_783_MOESM3_ESM.zip › Figure 1/Figure 1I/Untreated_8S.tif]

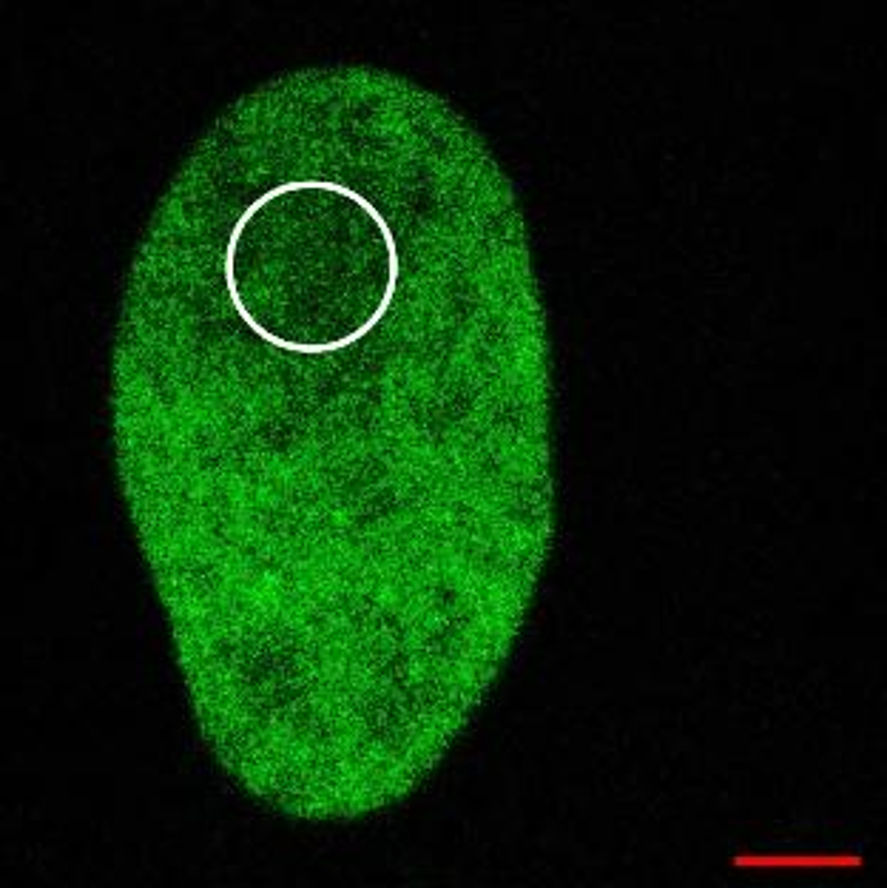

Supplement: Supplementary file 3 — Source data Fig. 1 [file 44318_2026_783_MOESM3_ESM.zip › Figure 1/Figure 1I/Untreated_90S.tif]

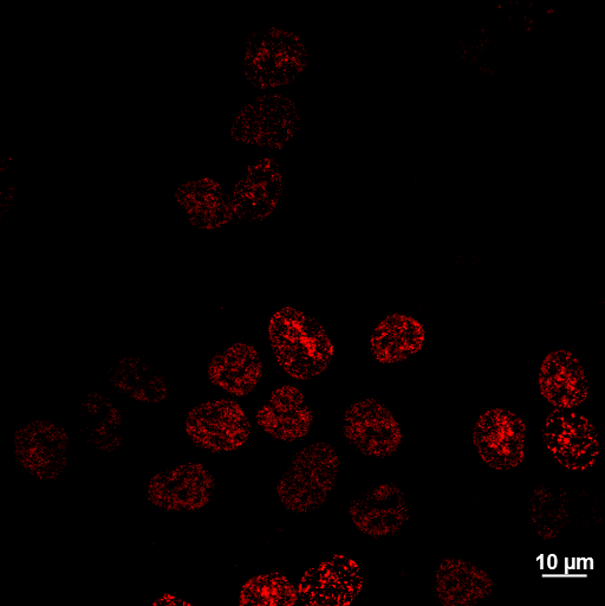

Supplement: Supplementary file 4 — Source data Fig. 2 [file 44318_2026_783_MOESM4_ESM.zip › Figure 2/Figure 2A/CHK1i_0h_EdU.tif]

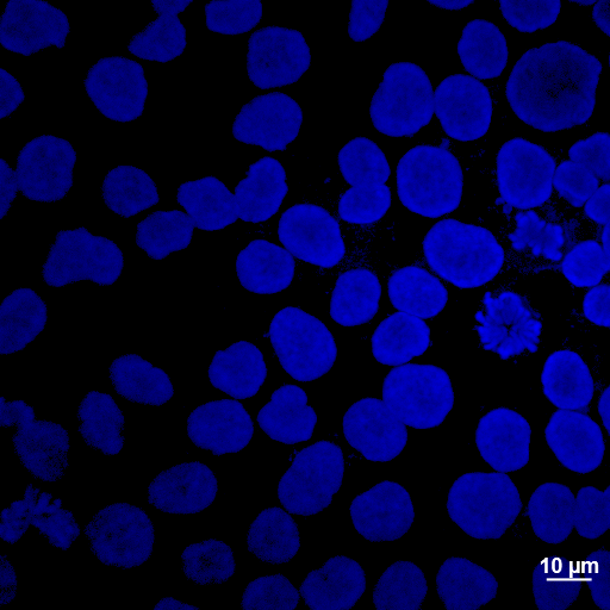

Supplement: Supplementary file 4 — Source data Fig. 2 [file 44318_2026_783_MOESM4_ESM.zip › Figure 2/Figure 2A/CHK1i_0h_Hoechst.tif]

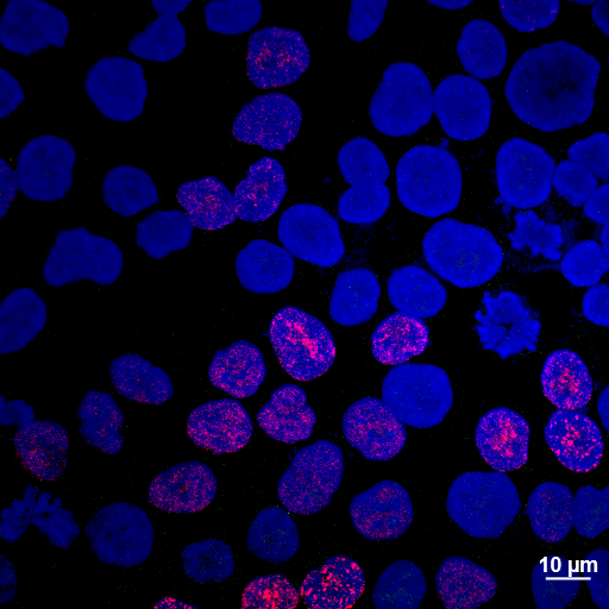

Supplement: Supplementary file 4 — Source data Fig. 2 [file 44318_2026_783_MOESM4_ESM.zip › Figure 2/Figure 2A/CHK1i_0h_Merged.tif]

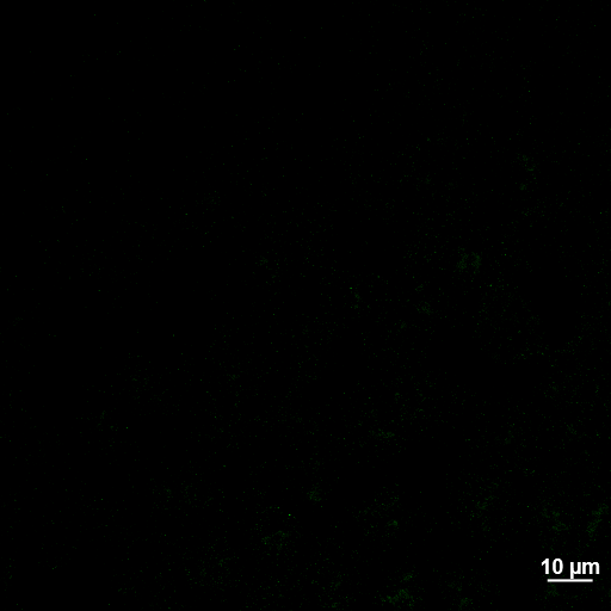

Supplement: Supplementary file 4 — Source data Fig. 2 [file 44318_2026_783_MOESM4_ESM.zip › Figure 2/Figure 2A/CHK1i_0h_TOP1cc.tif]

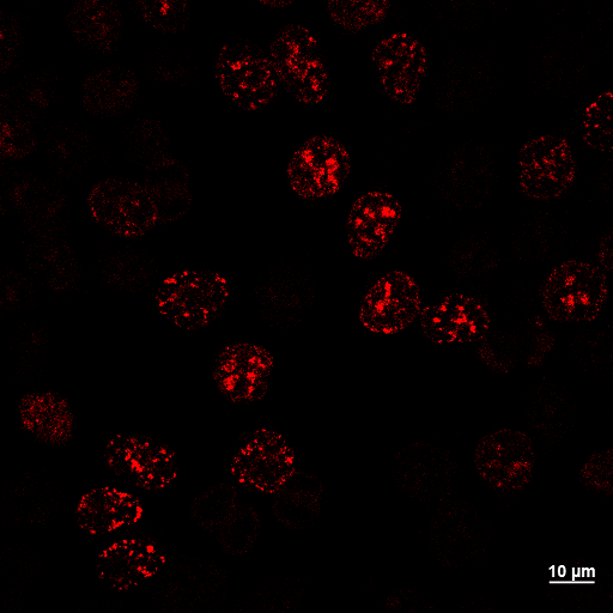

Supplement: Supplementary file 4 — Source data Fig. 2 [file 44318_2026_783_MOESM4_ESM.zip › Figure 2/Figure 2A/CHK1i_4h_EdU.tif]

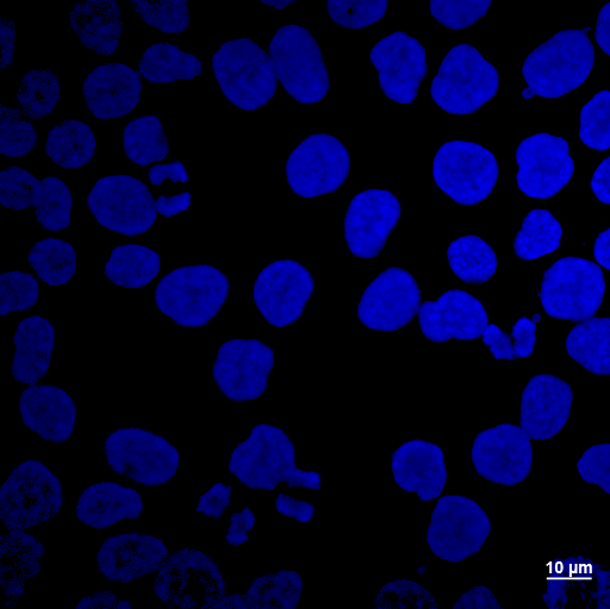

Supplement: Supplementary file 4 — Source data Fig. 2 [file 44318_2026_783_MOESM4_ESM.zip › Figure 2/Figure 2A/CHK1i_4h_Hoechst.tif]

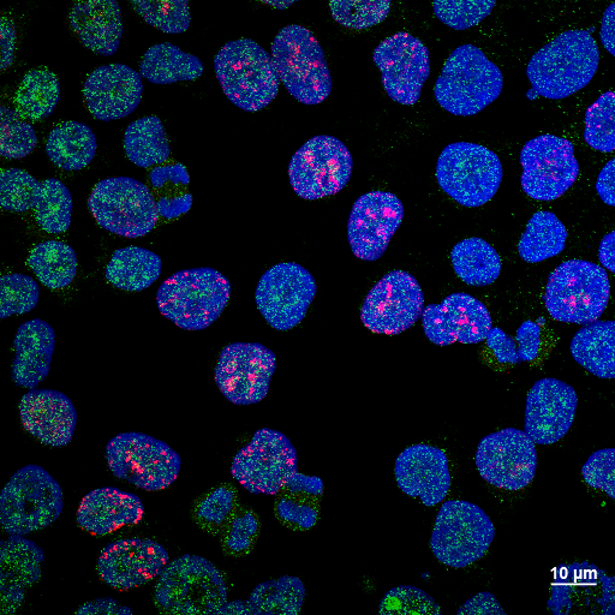

Supplement: Supplementary file 4 — Source data Fig. 2 [file 44318_2026_783_MOESM4_ESM.zip › Figure 2/Figure 2A/CHK1i_4h_Merged.tif]

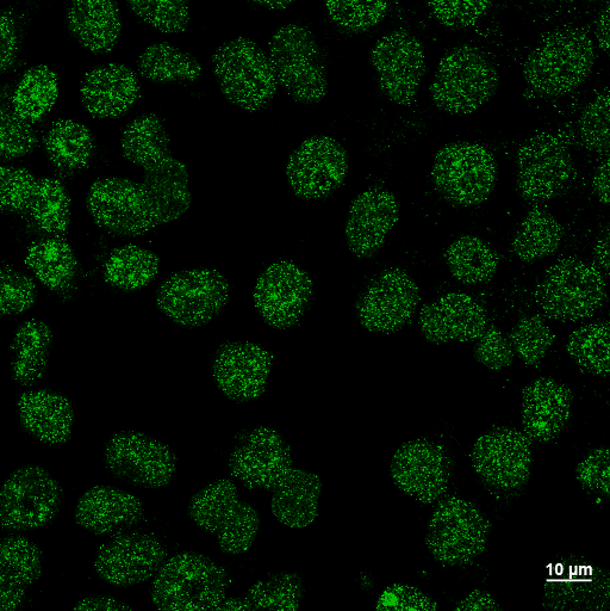

Supplement: Supplementary file 4 — Source data Fig. 2 [file 44318_2026_783_MOESM4_ESM.zip › Figure 2/Figure 2A/CHK1i_4h_TOP1cc.tif]

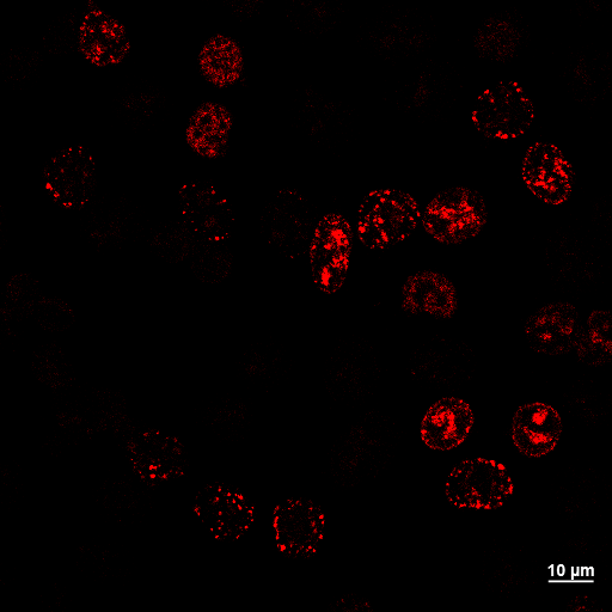

Supplement: Supplementary file 4 — Source data Fig. 2 [file 44318_2026_783_MOESM4_ESM.zip › Figure 2/Figure 2A/CHK1i_6h_EdU.tif]

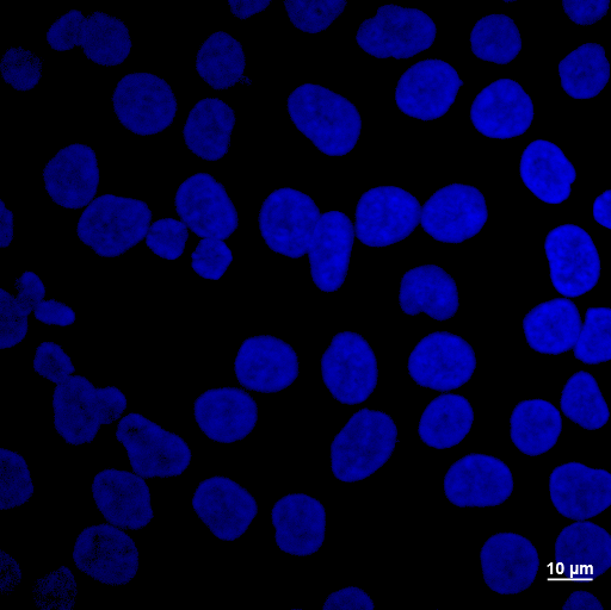

Supplement: Supplementary file 4 — Source data Fig. 2 [file 44318_2026_783_MOESM4_ESM.zip › Figure 2/Figure 2A/CHK1i_6h_Hoechst.tif]

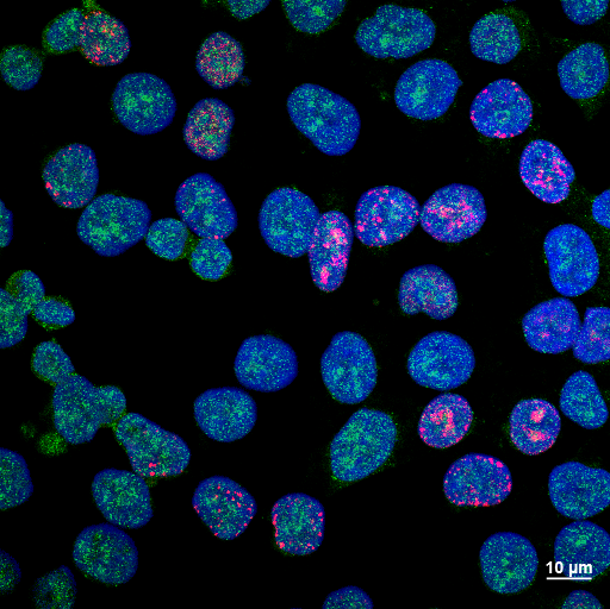

Supplement: Supplementary file 4 — Source data Fig. 2 [file 44318_2026_783_MOESM4_ESM.zip › Figure 2/Figure 2A/CHK1i_6h_Merged.tif]

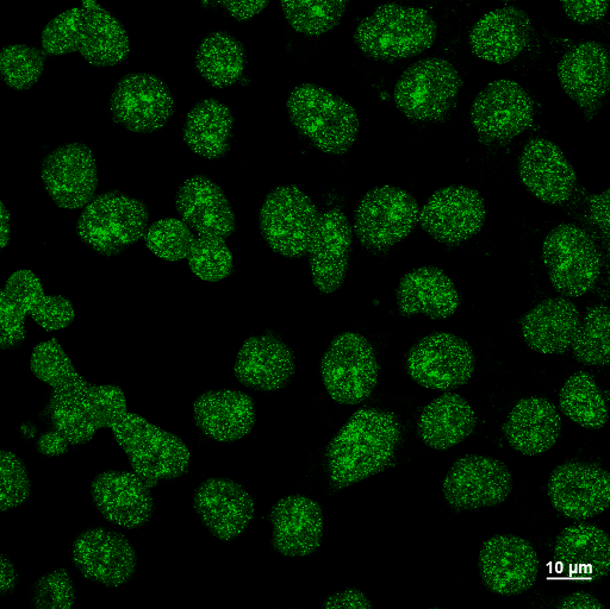

Supplement: Supplementary file 4 — Source data Fig. 2 [file 44318_2026_783_MOESM4_ESM.zip › Figure 2/Figure 2A/CHK1i_6h_TOP1cc.tif]

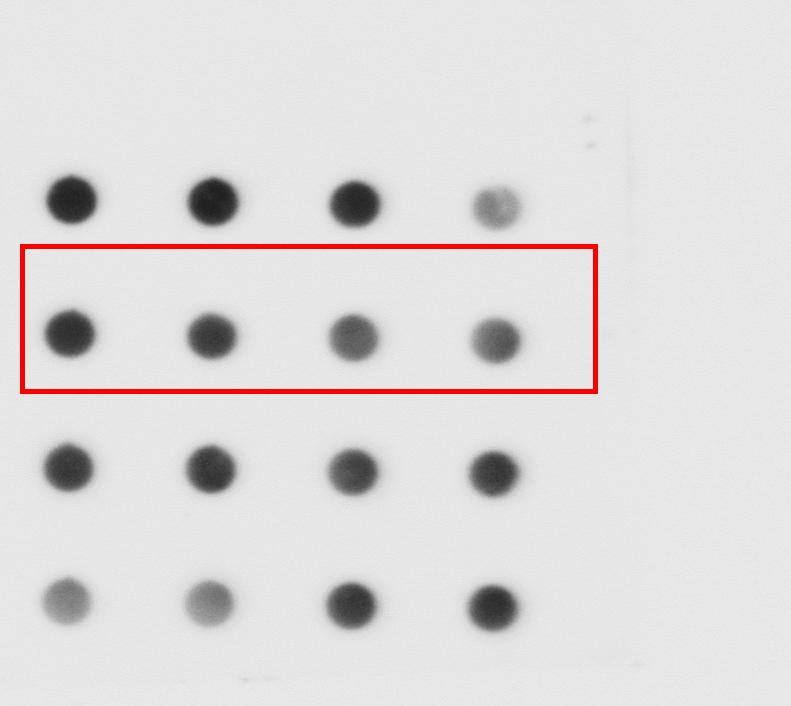

Supplement: Supplementary file 4 — Source data Fig. 2 [file 44318_2026_783_MOESM4_ESM.zip › Figure 2/Figure 2C/APH_DNA.tif]

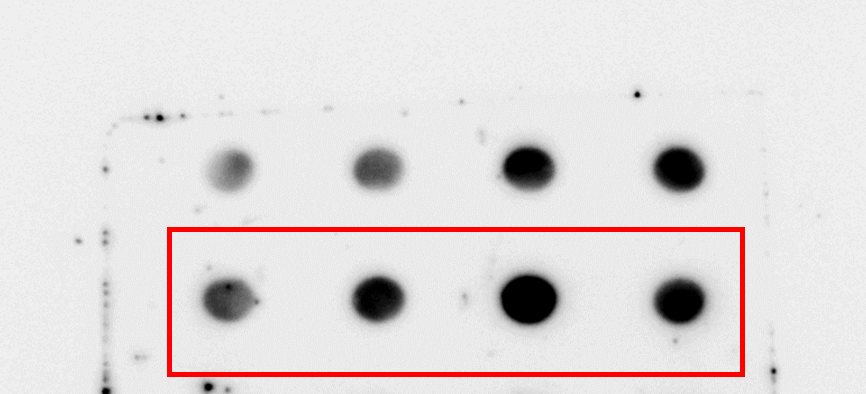

Supplement: Supplementary file 4 — Source data Fig. 2 [file 44318_2026_783_MOESM4_ESM.zip › Figure 2/Figure 2C/APH_TOP1.tif]

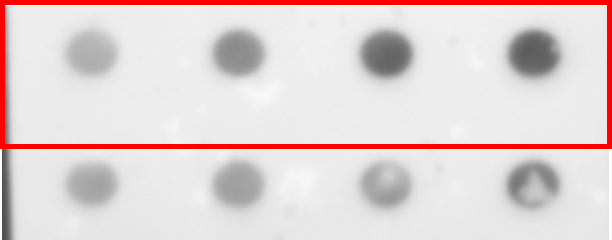

Supplement: Supplementary file 4 — Source data Fig. 2 [file 44318_2026_783_MOESM4_ESM.zip › Figure 2/Figure 2C/PHA_DNA.tif]

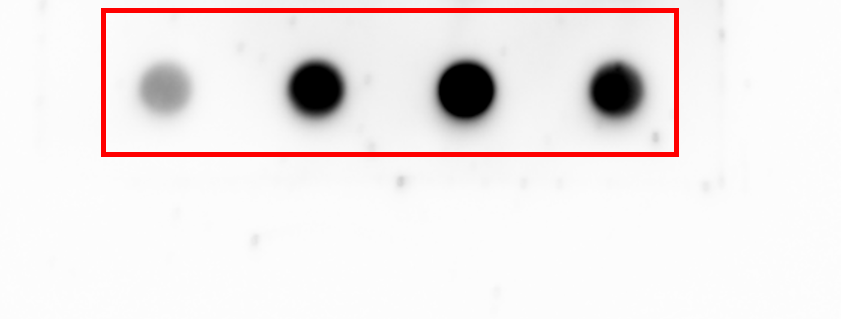

Supplement: Supplementary file 4 — Source data Fig. 2 [file 44318_2026_783_MOESM4_ESM.zip › Figure 2/Figure 2C/PHA_TOP1.tif]

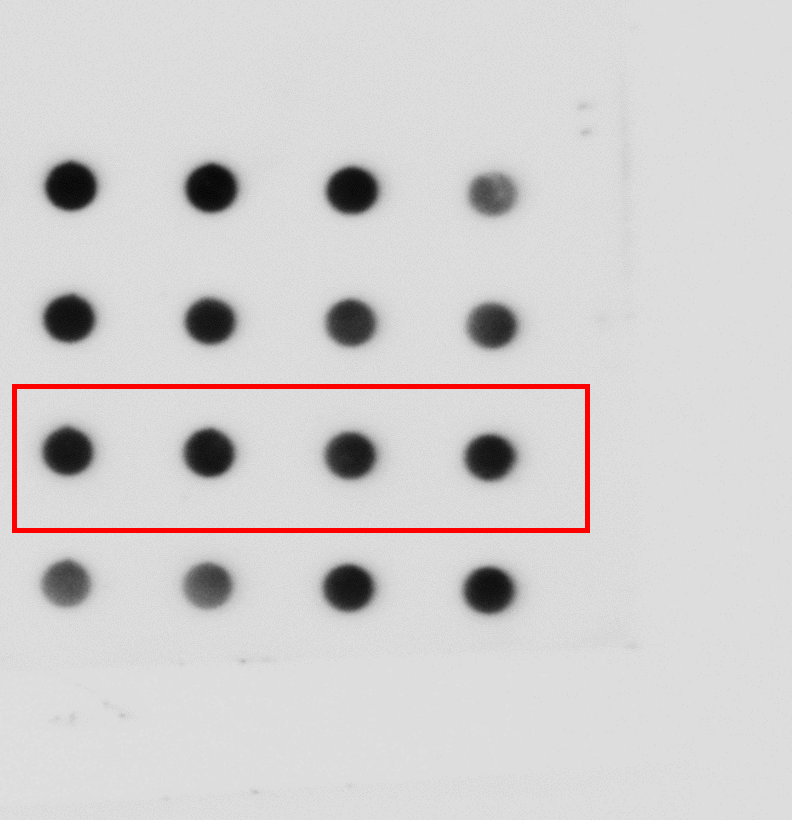

Supplement: Supplementary file 4 — Source data Fig. 2 [file 44318_2026_783_MOESM4_ESM.zip › Figure 2/Figure 2C/Serum Starvation_DNA.tif]

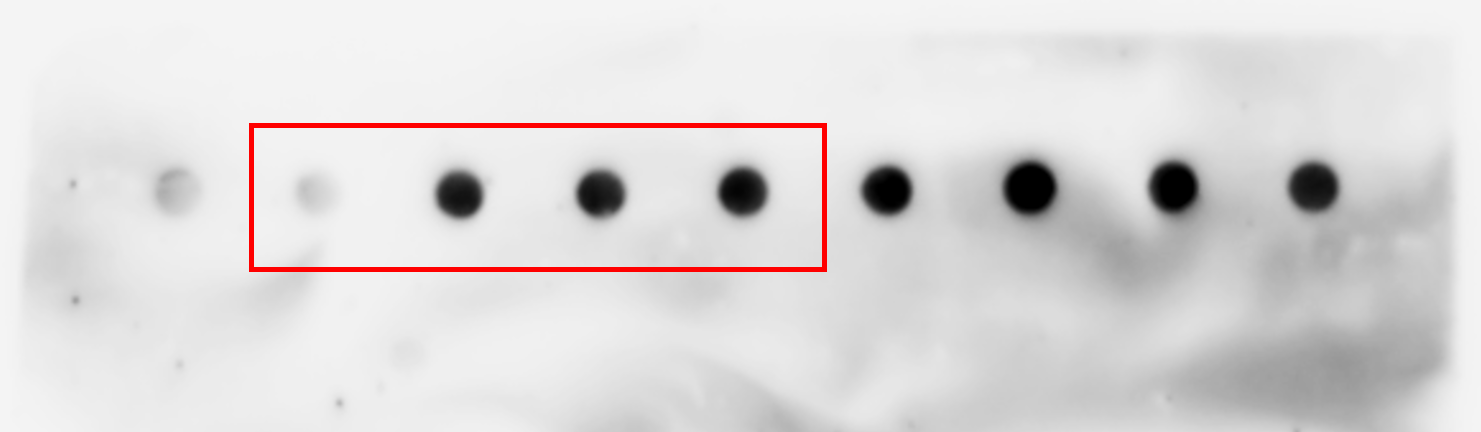

Supplement: Supplementary file 4 — Source data Fig. 2 [file 44318_2026_783_MOESM4_ESM.zip › Figure 2/Figure 2C/Serum Starvation_TOP1.tif]

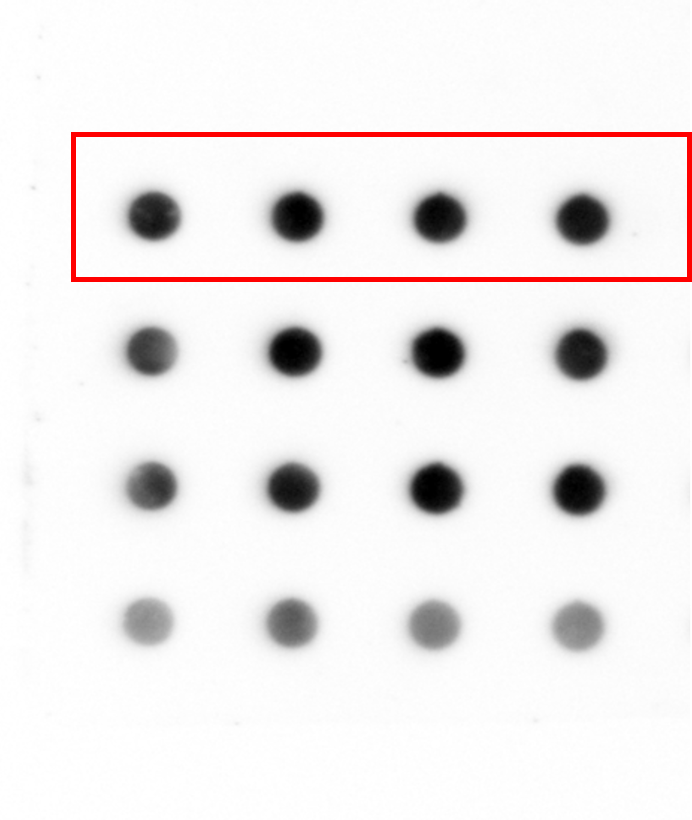

Supplement: Supplementary file 4 — Source data Fig. 2 [file 44318_2026_783_MOESM4_ESM.zip › Figure 2/Figure 2C/UT_DNA.tif]

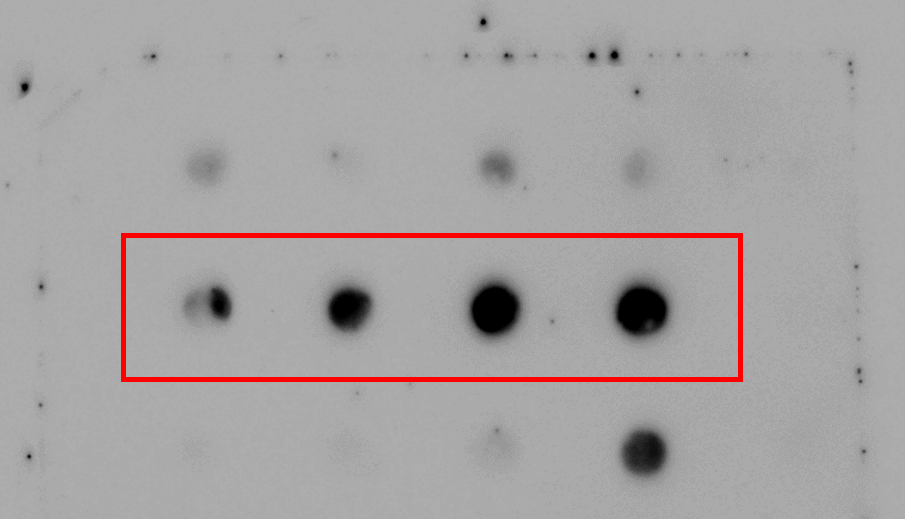

Supplement: Supplementary file 4 — Source data Fig. 2 [file 44318_2026_783_MOESM4_ESM.zip › Figure 2/Figure 2C/UT_TOP1.tif]

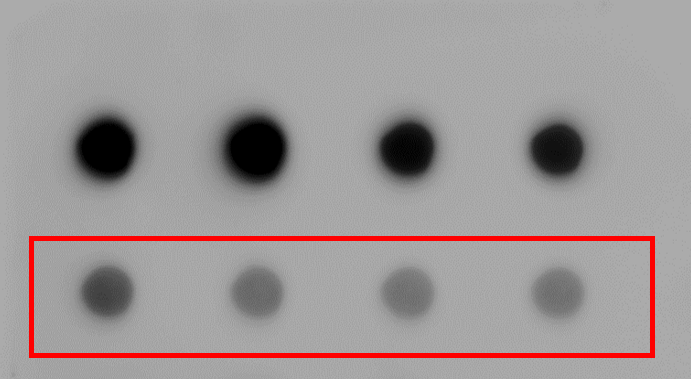

Supplement: Supplementary file 4 — Source data Fig. 2 [file 44318_2026_783_MOESM4_ESM.zip › Figure 2/Figure 2E/Amanitin_DNA.tif]

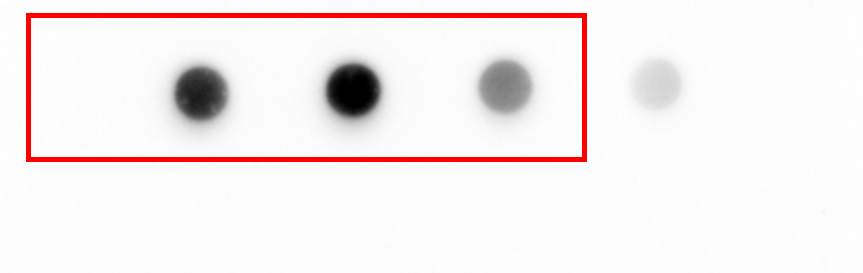

Supplement: Supplementary file 4 — Source data Fig. 2 [file 44318_2026_783_MOESM4_ESM.zip › Figure 2/Figure 2E/Amanitin_TOP1.tif]

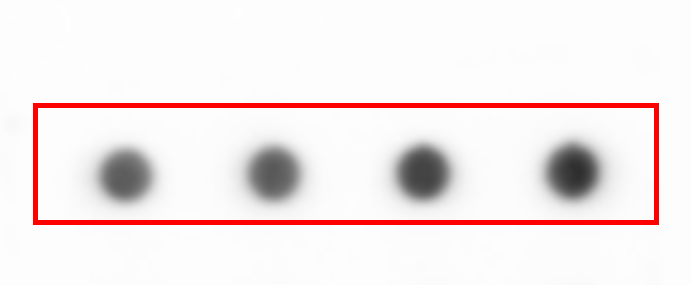

Supplement: Supplementary file 4 — Source data Fig. 2 [file 44318_2026_783_MOESM4_ESM.zip › Figure 2/Figure 2E/DRB_DNA.tif]

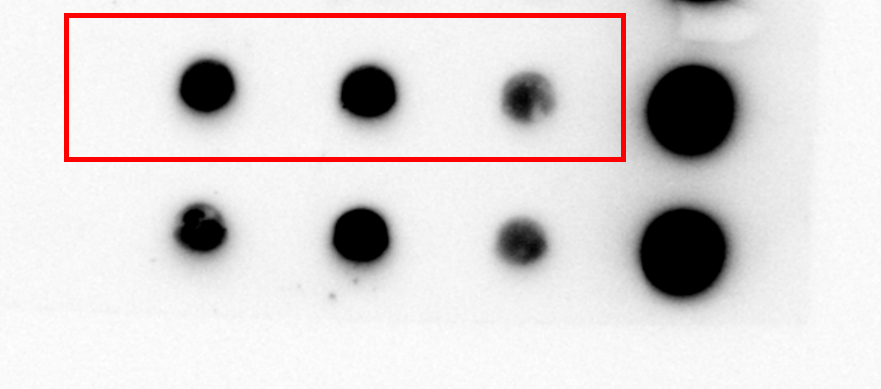

Supplement: Supplementary file 4 — Source data Fig. 2 [file 44318_2026_783_MOESM4_ESM.zip › Figure 2/Figure 2E/DRB_TOP1.tif]

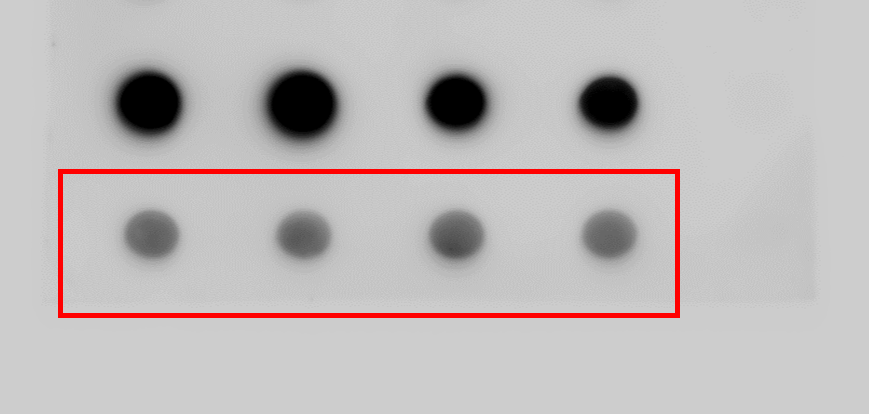

Supplement: Supplementary file 4 — Source data Fig. 2 [file 44318_2026_783_MOESM4_ESM.zip › Figure 2/Figure 2E/UT_DNA.tif]

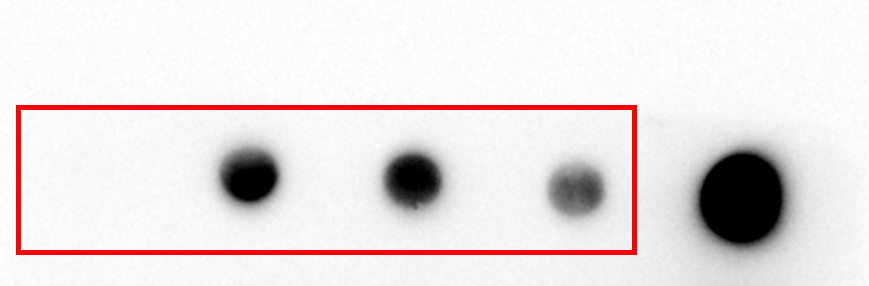

Supplement: Supplementary file 4 — Source data Fig. 2 [file 44318_2026_783_MOESM4_ESM.zip › Figure 2/Figure 2E/UT_TOP1.tif]

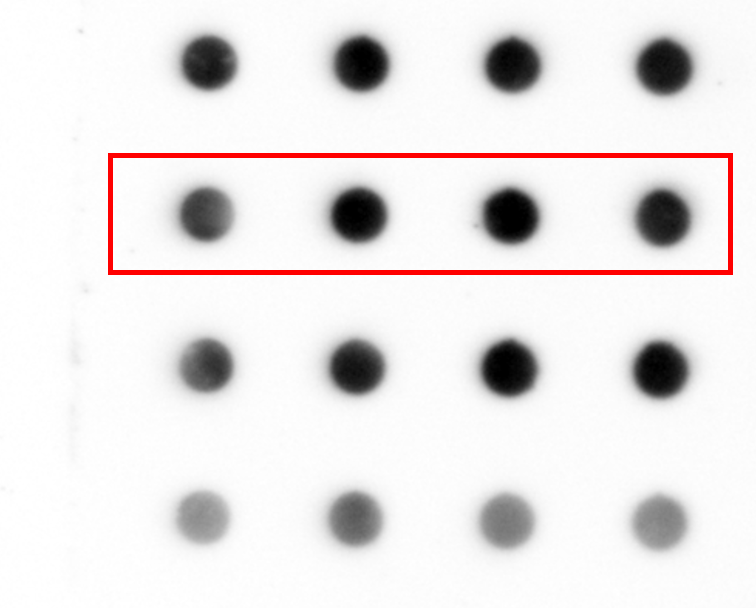

Supplement: Supplementary file 4 — Source data Fig. 2 [file 44318_2026_783_MOESM4_ESM.zip › Figure 2/Figure 2G/DNA.tif]

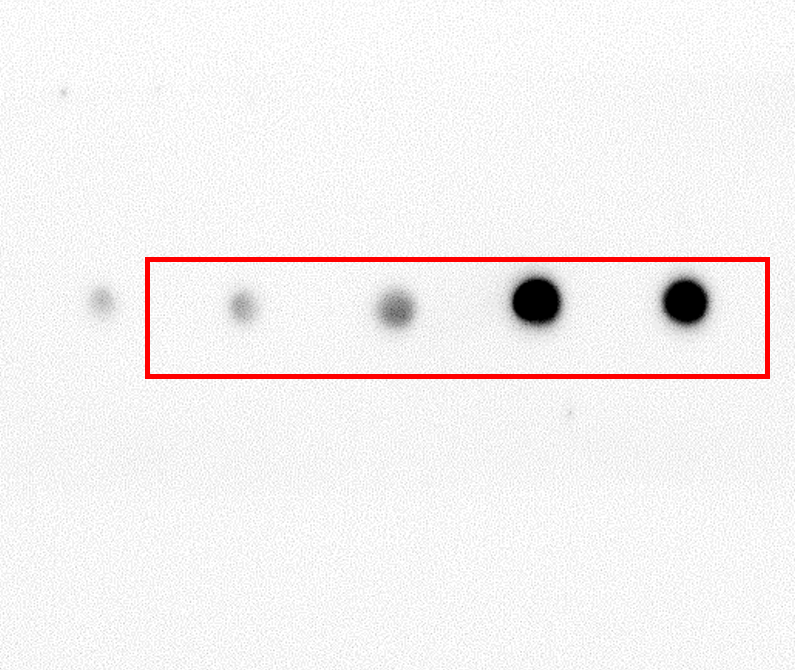

Supplement: Supplementary file 4 — Source data Fig. 2 [file 44318_2026_783_MOESM4_ESM.zip › Figure 2/Figure 2G/TOP1.tif]

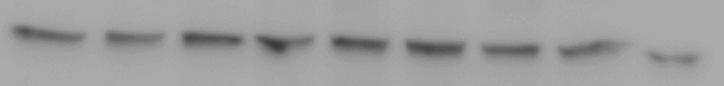

Supplement: Supplementary file 5 — Source data Fig. 3 [file 44318_2026_783_MOESM5_ESM.zip › Figure 3/Figure 3A/Beta Actin.tif]

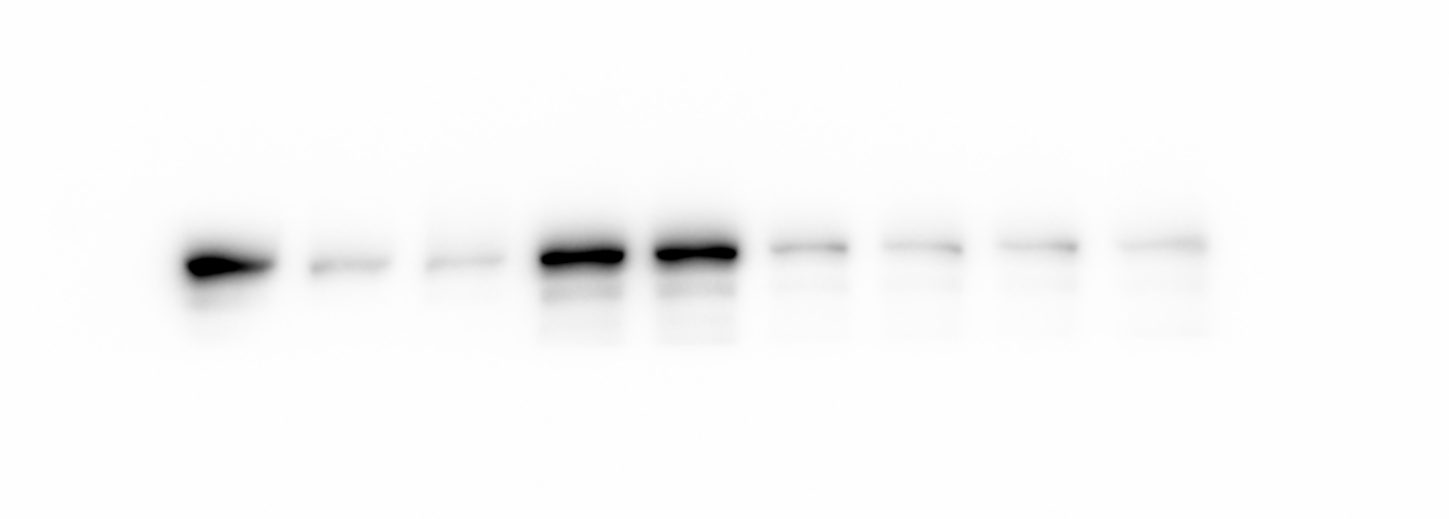

Supplement: Supplementary file 5 — Source data Fig. 3 [file 44318_2026_783_MOESM5_ESM.zip › Figure 3/Figure 3A/TOP1.tif]

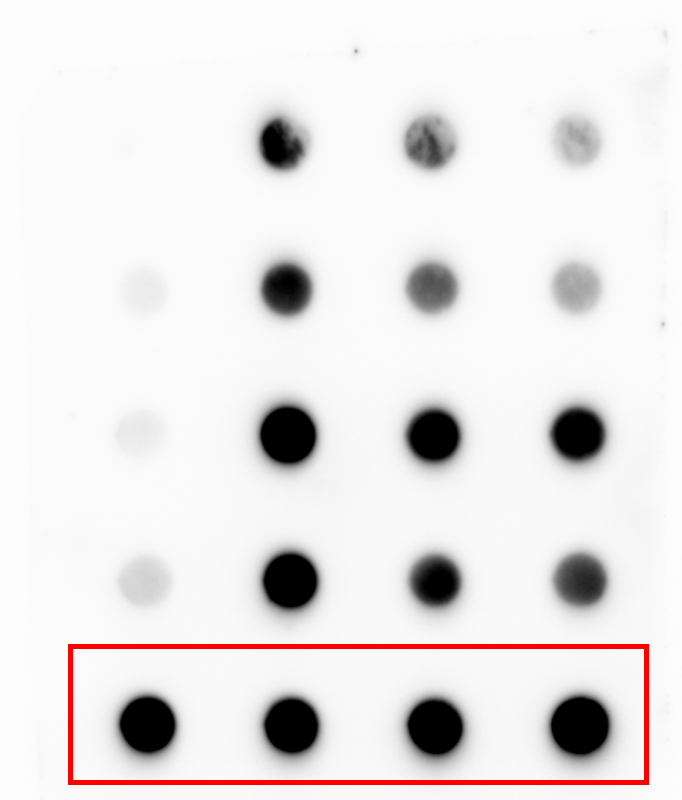

Supplement: Supplementary file 5 — Source data Fig. 3 [file 44318_2026_783_MOESM5_ESM.zip › Figure 3/Figure 3B/CPT_DNA.tif]

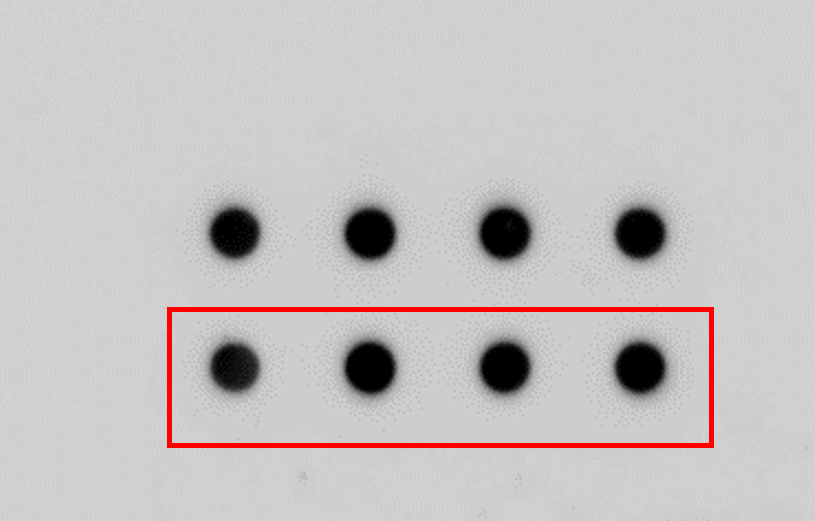

Supplement: Supplementary file 5 — Source data Fig. 3 [file 44318_2026_783_MOESM5_ESM.zip › Figure 3/Figure 3B/CPT_SCH_DNA.tif]

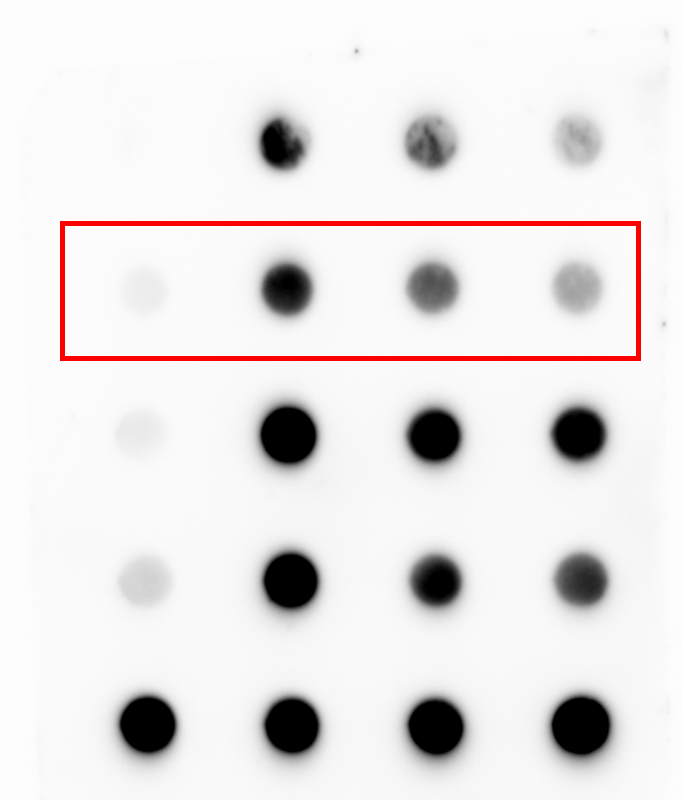

Supplement: Supplementary file 5 — Source data Fig. 3 [file 44318_2026_783_MOESM5_ESM.zip › Figure 3/Figure 3B/CPT_SCH_TOP1.tif]

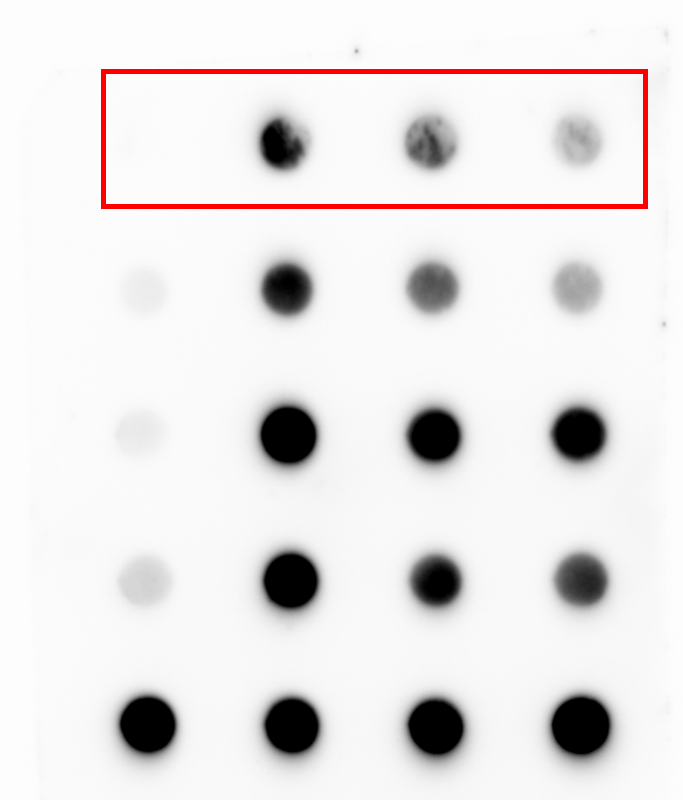

Supplement: Supplementary file 5 — Source data Fig. 3 [file 44318_2026_783_MOESM5_ESM.zip › Figure 3/Figure 3B/CPT_TOP1.tif]

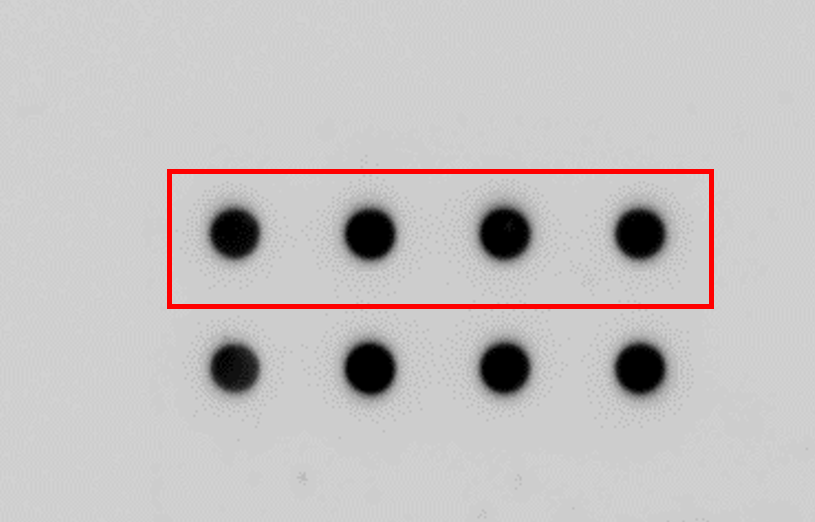

Supplement: Supplementary file 5 — Source data Fig. 3 [file 44318_2026_783_MOESM5_ESM.zip › Figure 3/Figure 3B/SCH_DNA.tif]

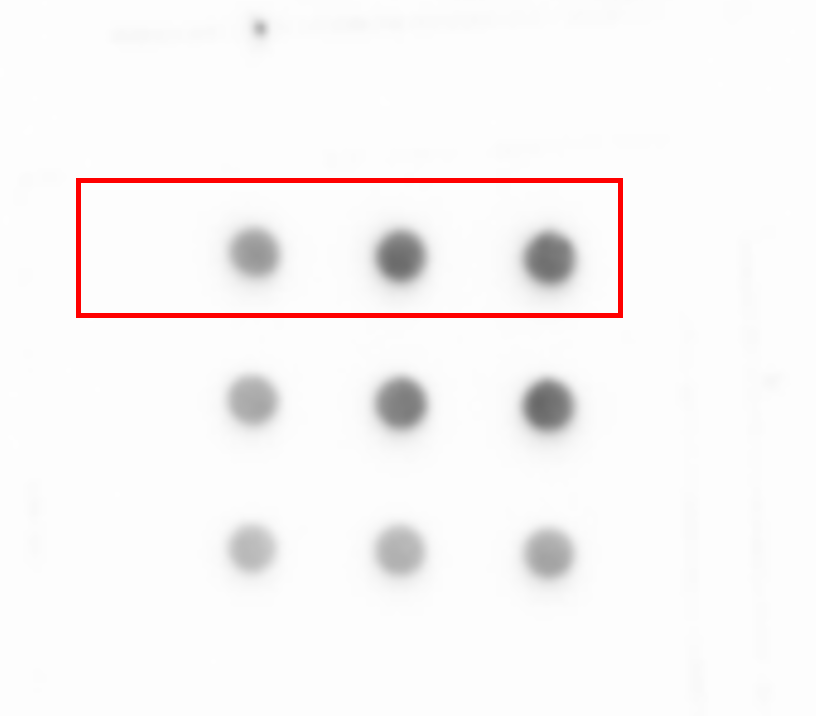

Supplement: Supplementary file 5 — Source data Fig. 3 [file 44318_2026_783_MOESM5_ESM.zip › Figure 3/Figure 3B/SCH_TOP1.tif]

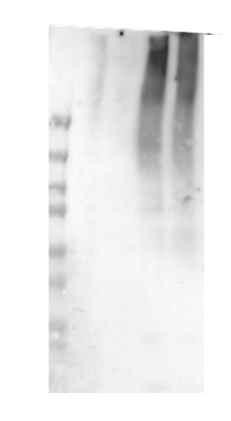

Supplement: Supplementary file 5 — Source data Fig. 3 [file 44318_2026_783_MOESM5_ESM.zip › Figure 3/Figure 3C/PAR.tif]

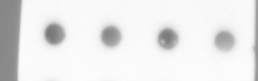

Supplement: Supplementary file 5 — Source data Fig. 3 [file 44318_2026_783_MOESM5_ESM.zip › Figure 3/Figure 3C/PAR_DNA.tif]

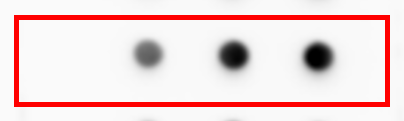

Supplement: Supplementary file 5 — Source data Fig. 3 [file 44318_2026_783_MOESM5_ESM.zip › Figure 3/Figure 3C/PAR_TOP1.tif]

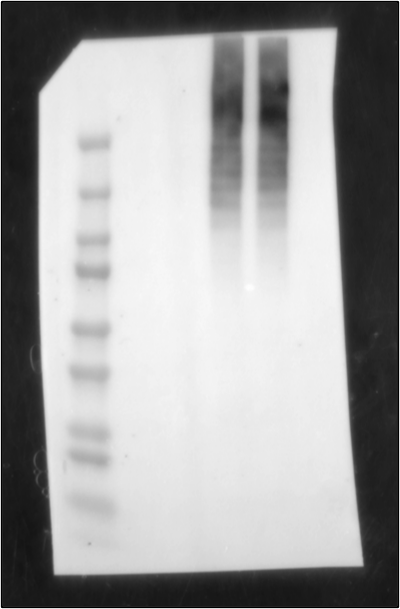

Supplement: Supplementary file 5 — Source data Fig. 3 [file 44318_2026_783_MOESM5_ESM.zip › Figure 3/Figure 3C/SUMO1.tif]

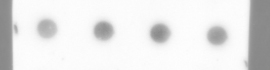

Supplement: Supplementary file 5 — Source data Fig. 3 [file 44318_2026_783_MOESM5_ESM.zip › Figure 3/Figure 3C/SUMO1_2_3_DNA.tif]

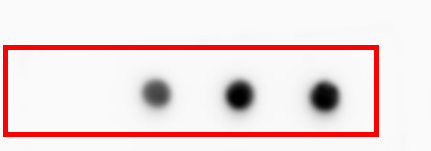

Supplement: Supplementary file 5 — Source data Fig. 3 [file 44318_2026_783_MOESM5_ESM.zip › Figure 3/Figure 3C/SUMO1_2_3_TOP1.tif]

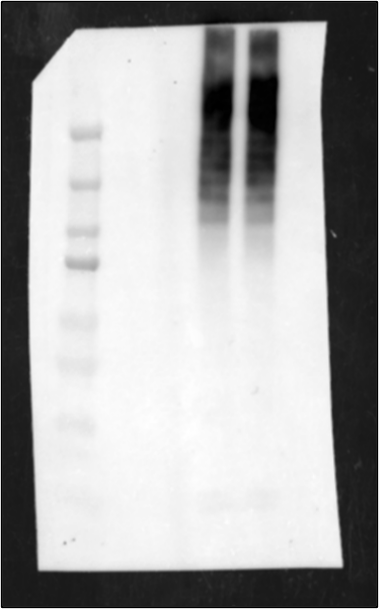

Supplement: Supplementary file 5 — Source data Fig. 3 [file 44318_2026_783_MOESM5_ESM.zip › Figure 3/Figure 3C/SUMO2_3.tif]

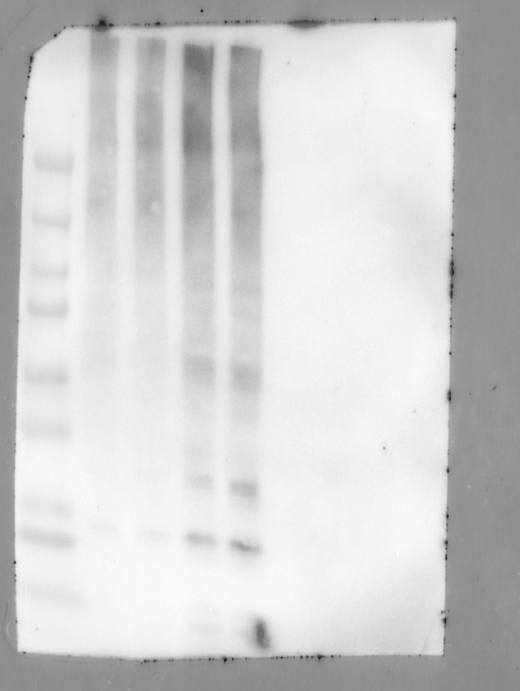

Supplement: Supplementary file 5 — Source data Fig. 3 [file 44318_2026_783_MOESM5_ESM.zip › Figure 3/Figure 3C/Ub.tif]

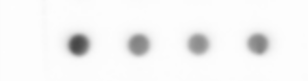

Supplement: Supplementary file 5 — Source data Fig. 3 [file 44318_2026_783_MOESM5_ESM.zip › Figure 3/Figure 3C/Ub_DNA.tif]

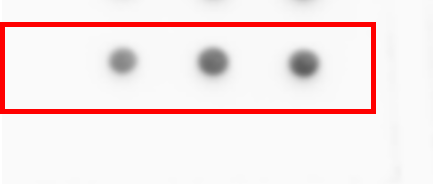

Supplement: Supplementary file 5 — Source data Fig. 3 [file 44318_2026_783_MOESM5_ESM.zip › Figure 3/Figure 3C/Ub_TOP1.tif]

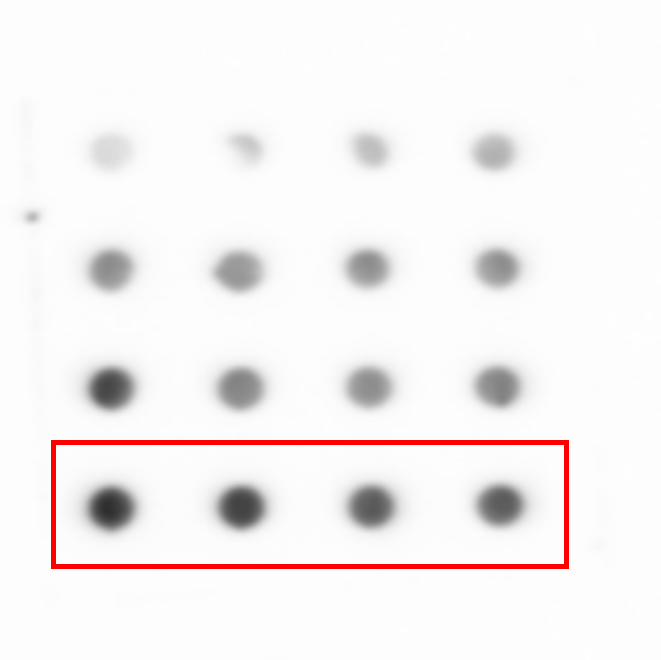

Supplement: Supplementary file 5 — Source data Fig. 3 [file 44318_2026_783_MOESM5_ESM.zip › Figure 3/Figure 3E/1uM_DNA.tif]

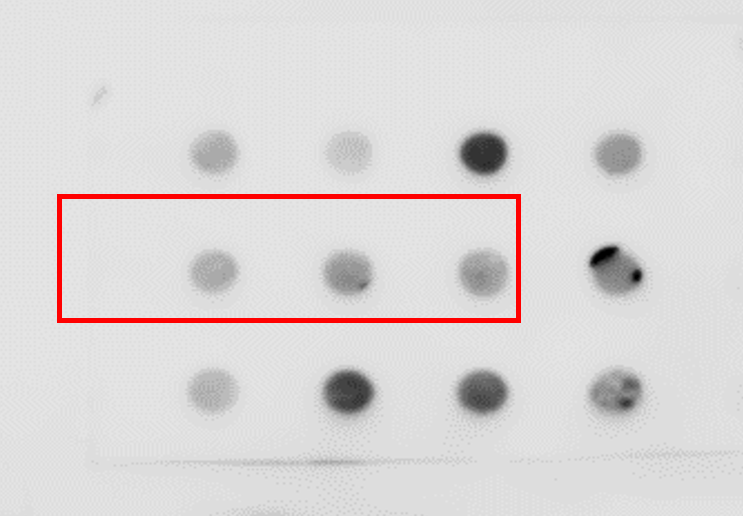

Supplement: Supplementary file 5 — Source data Fig. 3 [file 44318_2026_783_MOESM5_ESM.zip › Figure 3/Figure 3E/1uM_TOP1.tif]

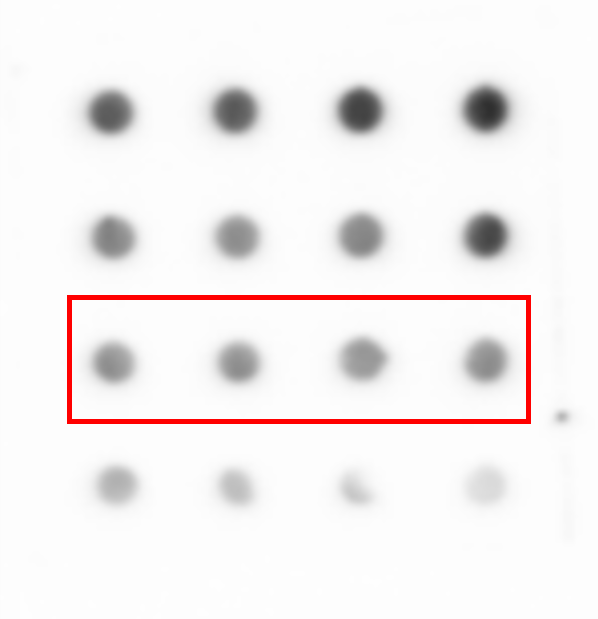

Supplement: Supplementary file 5 — Source data Fig. 3 [file 44318_2026_783_MOESM5_ESM.zip › Figure 3/Figure 3E/500nM_DNA.tif]

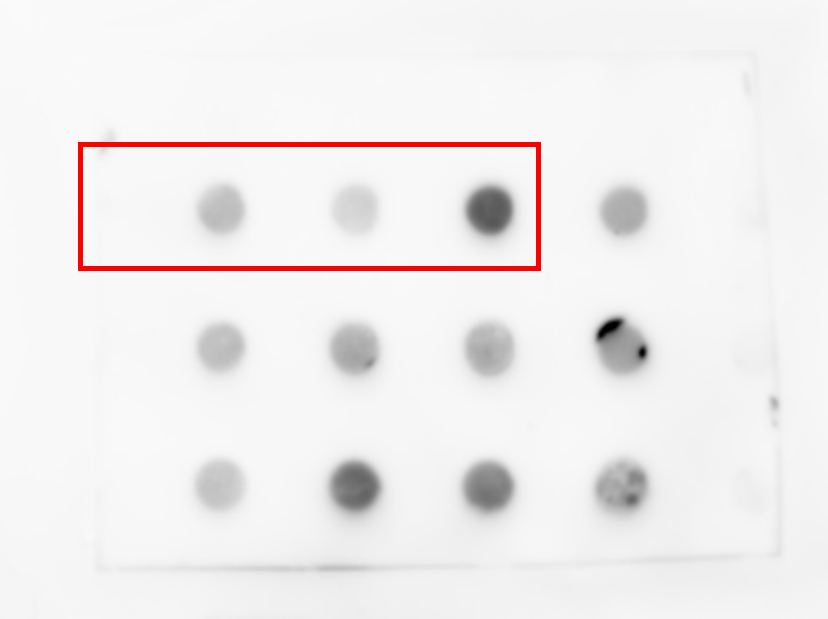

Supplement: Supplementary file 5 — Source data Fig. 3 [file 44318_2026_783_MOESM5_ESM.zip › Figure 3/Figure 3E/500nM_TOP1.tif]

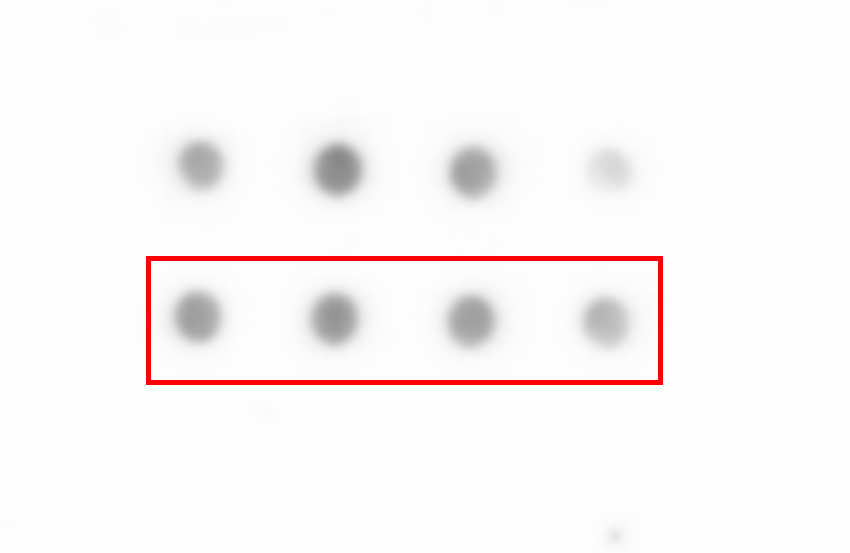

Supplement: Supplementary file 5 — Source data Fig. 3 [file 44318_2026_783_MOESM5_ESM.zip › Figure 3/Figure 3E/5uM_DNA.tif]

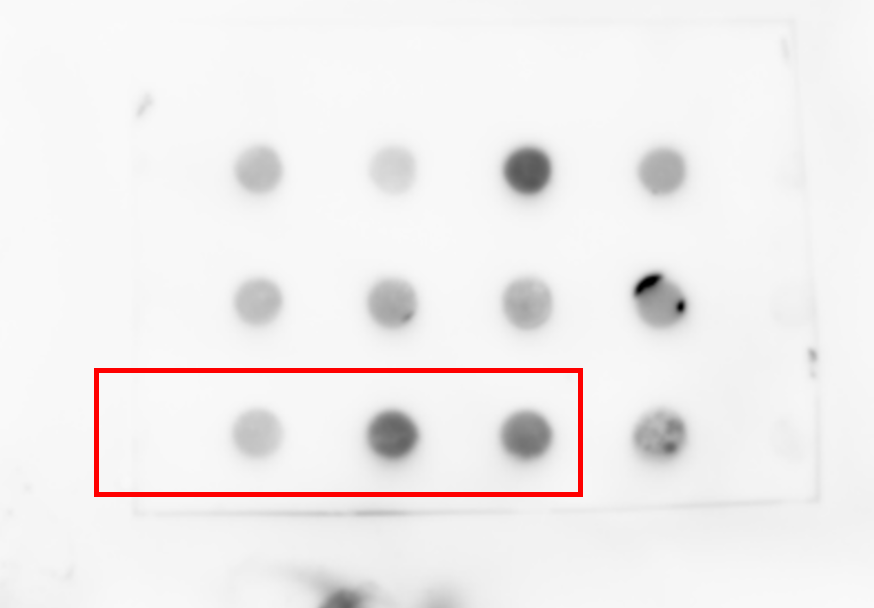

Supplement: Supplementary file 5 — Source data Fig. 3 [file 44318_2026_783_MOESM5_ESM.zip › Figure 3/Figure 3E/5uM_TOP1.tif]

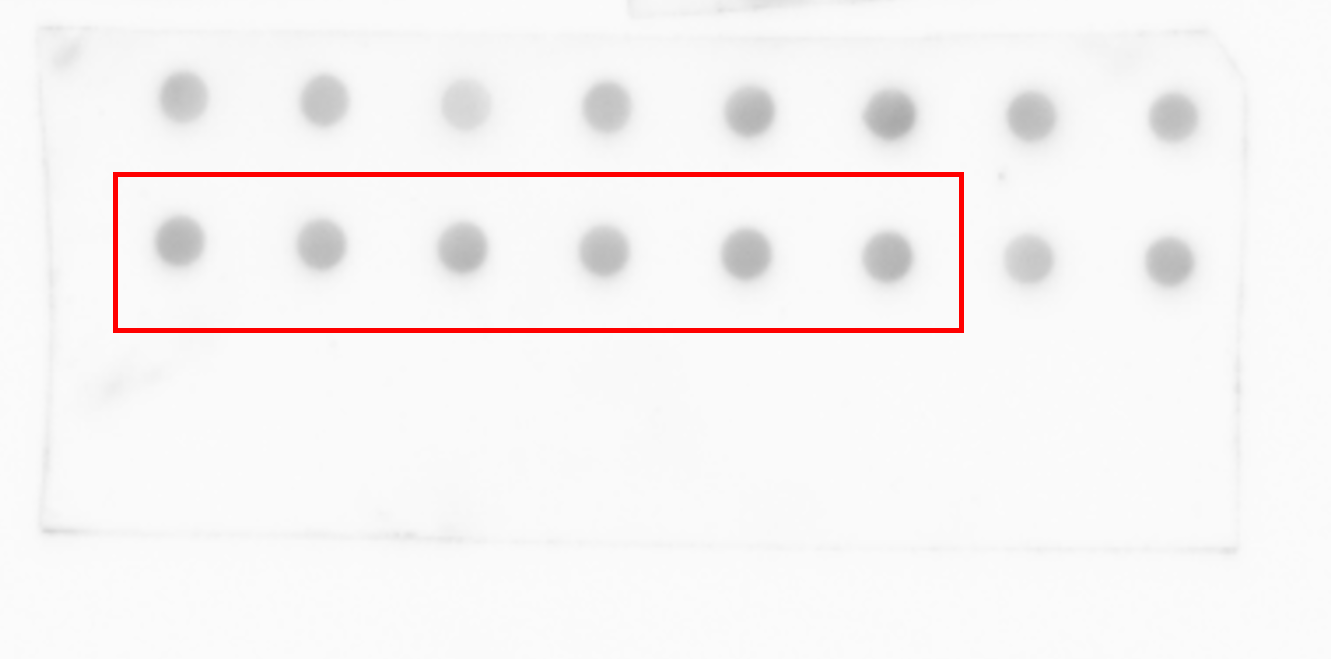

Supplement: Supplementary file 5 — Source data Fig. 3 [file 44318_2026_783_MOESM5_ESM.zip › Figure 3/Figure 3F/DNA.tif]

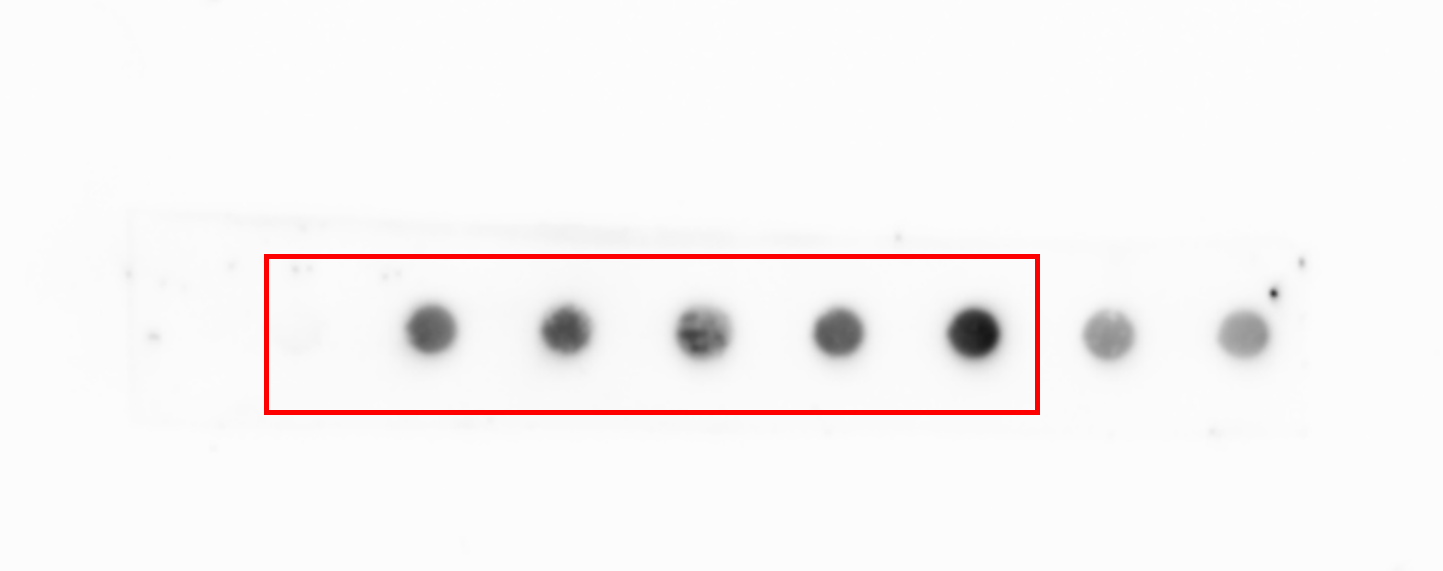

Supplement: Supplementary file 5 — Source data Fig. 3 [file 44318_2026_783_MOESM5_ESM.zip › Figure 3/Figure 3F/TOP1.tif]

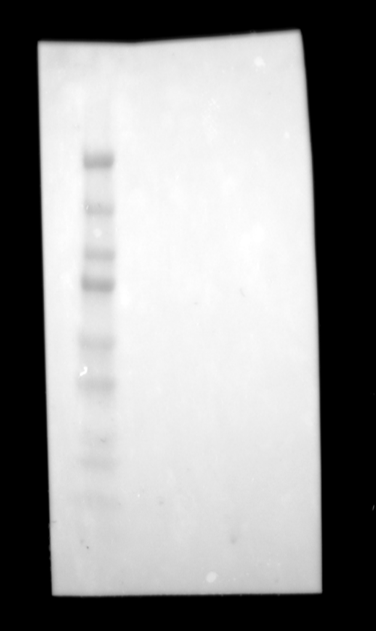

Supplement: Supplementary file 5 — Source data Fig. 3 [file 44318_2026_783_MOESM5_ESM.zip › Figure 3/Figure 3G/PAR.tif]

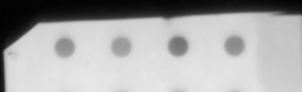

Supplement: Supplementary file 5 — Source data Fig. 3 [file 44318_2026_783_MOESM5_ESM.zip › Figure 3/Figure 3G/PAR_DNA.tif]

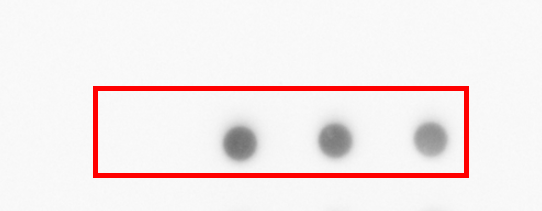

Supplement: Supplementary file 5 — Source data Fig. 3 [file 44318_2026_783_MOESM5_ESM.zip › Figure 3/Figure 3G/PAR_TOP1.tif]

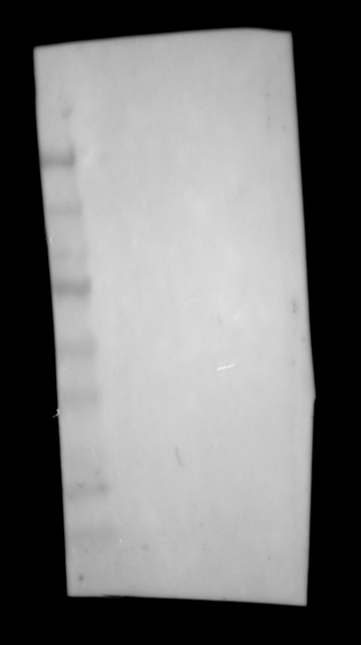

Supplement: Supplementary file 5 — Source data Fig. 3 [file 44318_2026_783_MOESM5_ESM.zip › Figure 3/Figure 3G/SUMO1.tif]

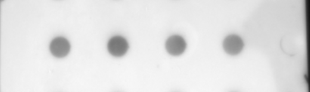

Supplement: Supplementary file 5 — Source data Fig. 3 [file 44318_2026_783_MOESM5_ESM.zip › Figure 3/Figure 3G/SUMO1_2_3_DNA.tif]

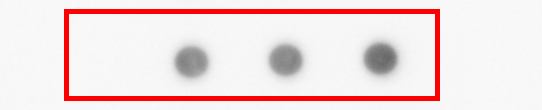

Supplement: Supplementary file 5 — Source data Fig. 3 [file 44318_2026_783_MOESM5_ESM.zip › Figure 3/Figure 3G/SUMO1_2_3_TOP1.tif]

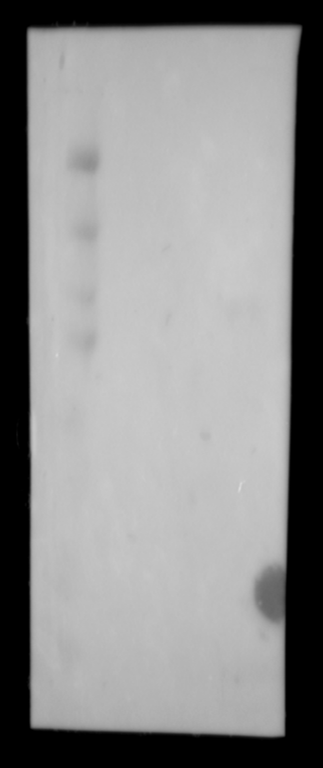

Supplement: Supplementary file 5 — Source data Fig. 3 [file 44318_2026_783_MOESM5_ESM.zip › Figure 3/Figure 3G/SUMO2_3.tif]

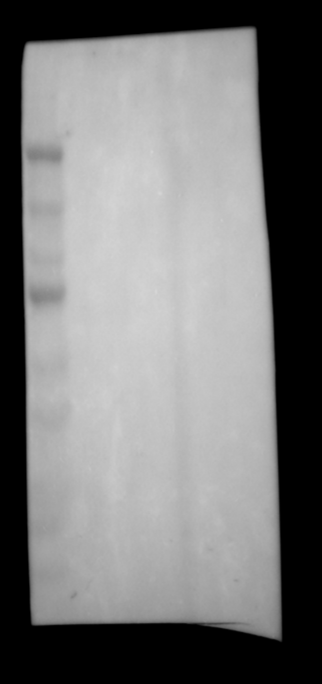

Supplement: Supplementary file 5 — Source data Fig. 3 [file 44318_2026_783_MOESM5_ESM.zip › Figure 3/Figure 3G/Ub.tif]

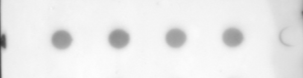

Supplement: Supplementary file 5 — Source data Fig. 3 [file 44318_2026_783_MOESM5_ESM.zip › Figure 3/Figure 3G/Ub_DNA.tif]

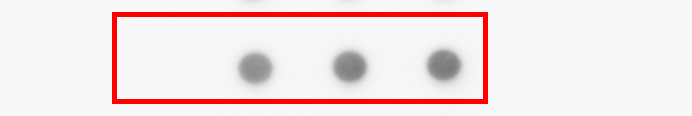

Supplement: Supplementary file 5 — Source data Fig. 3 [file 44318_2026_783_MOESM5_ESM.zip › Figure 3/Figure 3G/Ub_TOP1.tif]

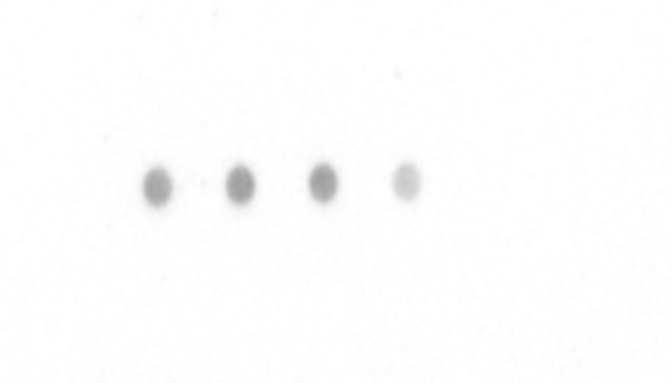

Supplement: Supplementary file 5 — Source data Fig. 3 [file 44318_2026_783_MOESM5_ESM.zip › Figure 3/Figure 3I/Figure 3I siSPRTN_DNA.tif]

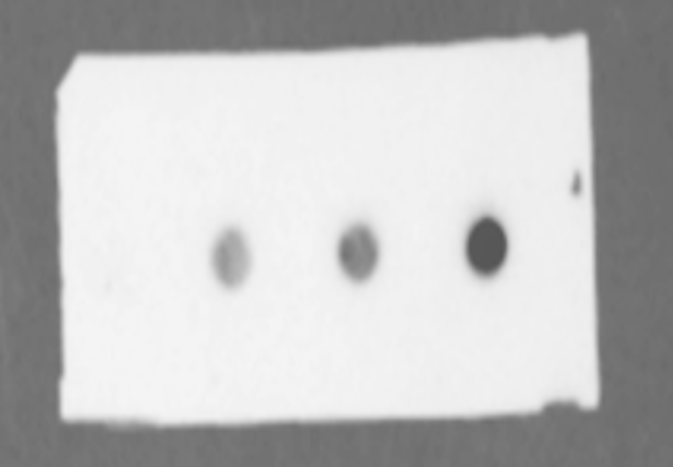

Supplement: Supplementary file 5 — Source data Fig. 3 [file 44318_2026_783_MOESM5_ESM.zip › Figure 3/Figure 3I/Figure 3I siSPRTN_TOP1.tif]

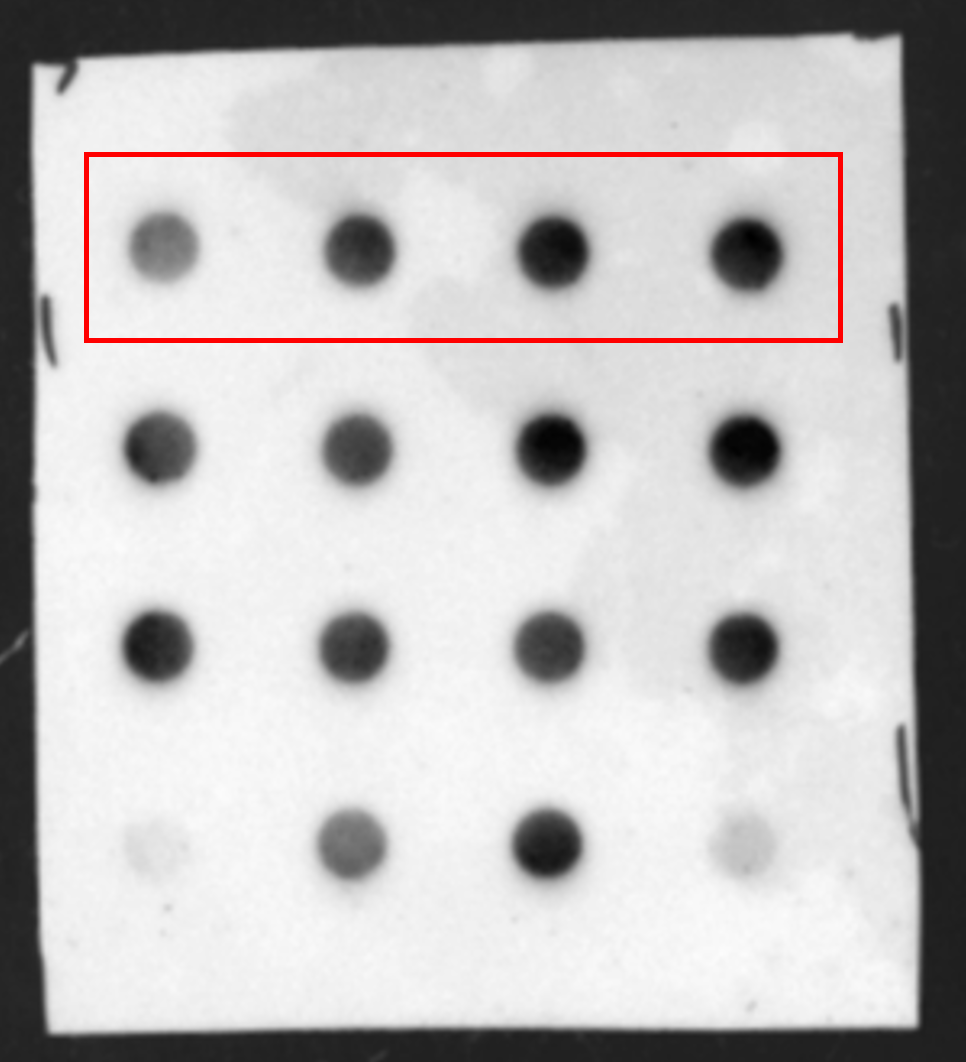

Supplement: Supplementary file 5 — Source data Fig. 3 [file 44318_2026_783_MOESM5_ESM.zip › Figure 3/Figure 3I/p97i_DNA.tif]

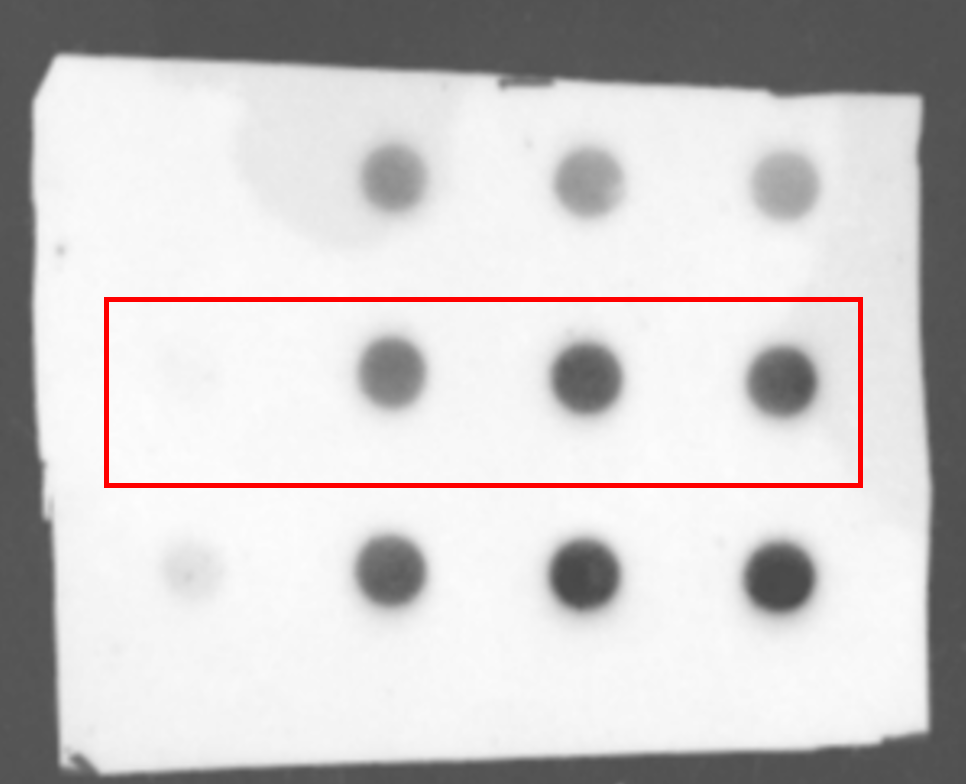

Supplement: Supplementary file 5 — Source data Fig. 3 [file 44318_2026_783_MOESM5_ESM.zip › Figure 3/Figure 3I/p97i_TOP1.tif]
