# Supplementary figures and images for: A CHK1-mediated phosphorylation switch suppresses human Topoisomerase 1-associated genomic instability (part 2 of 3)
Source: EMBO J. 2026 May 13;45(12):4220–56. doi: 10.1038/s44318-026-00783-3 (PMC13270093; doi:10.1038/s44318-026-00783-3)

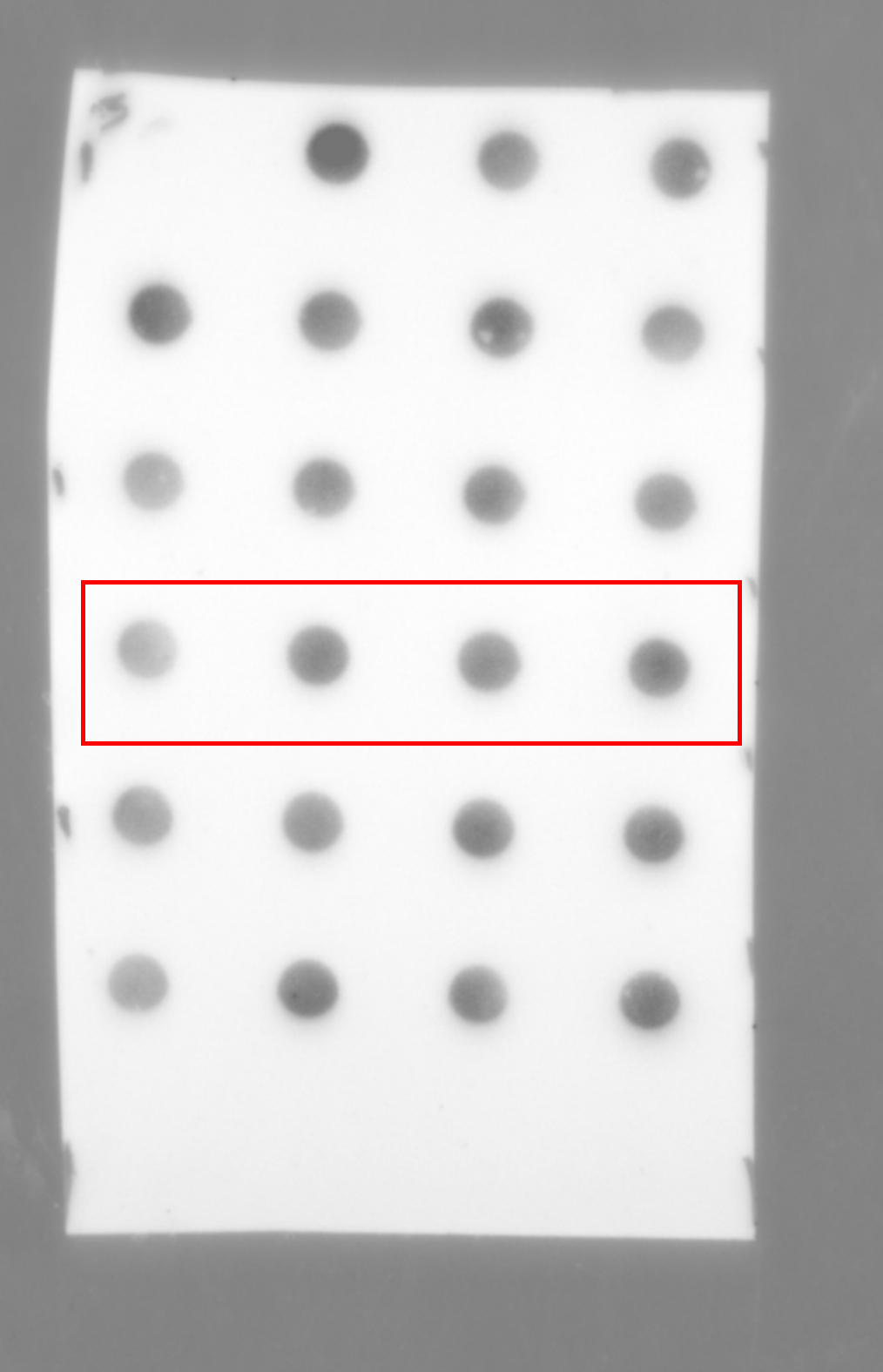

Supplement: Supplementary file 5 — Source data Fig. 3 [file 44318_2026_783_MOESM5_ESM.zip › Figure 3/Figure 3I/siATG7_DNA.tif]

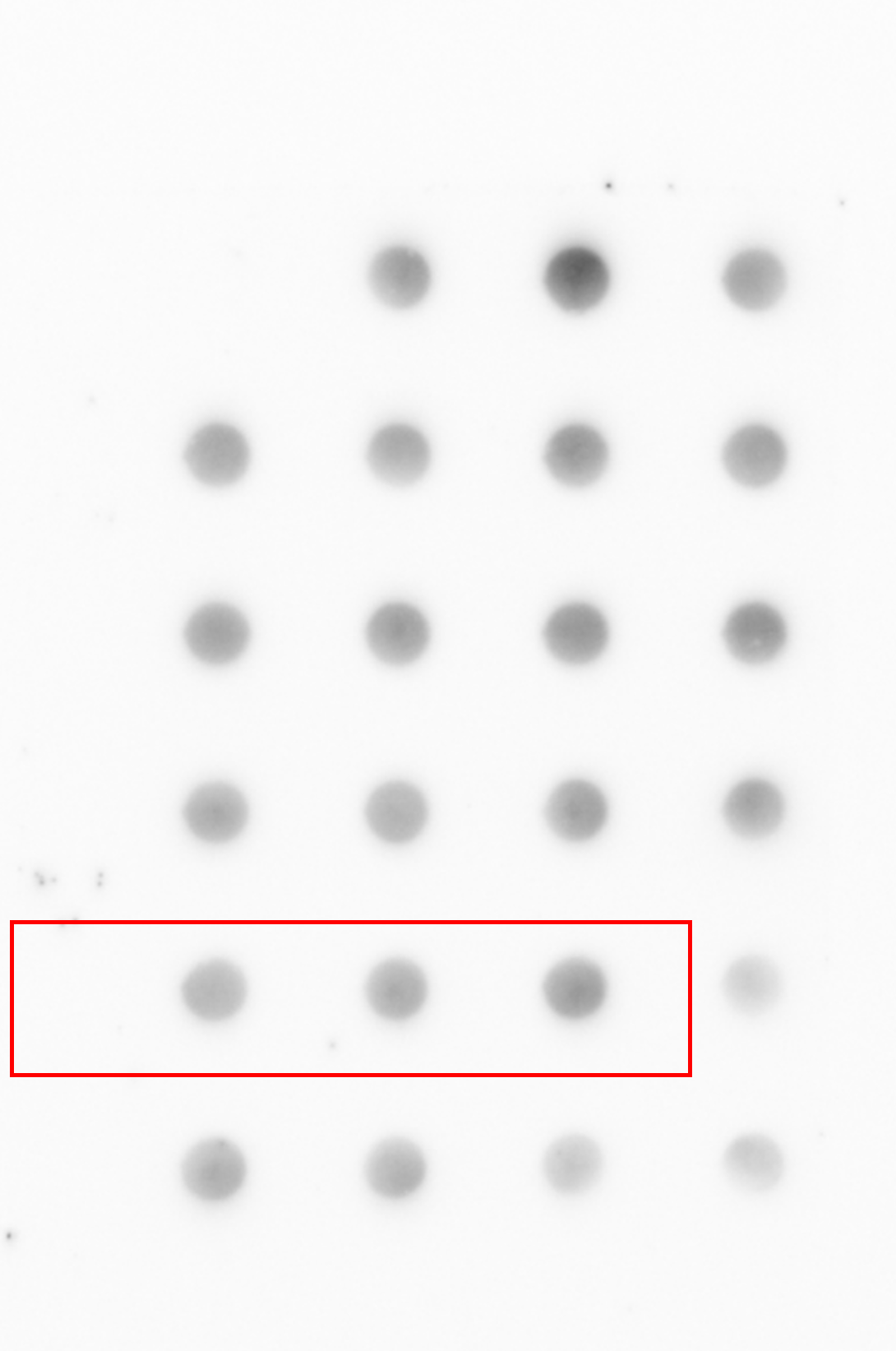

Supplement: Supplementary file 5 — Source data Fig. 3 [file 44318_2026_783_MOESM5_ESM.zip › Figure 3/Figure 3I/siATG7_TOP1.tif]

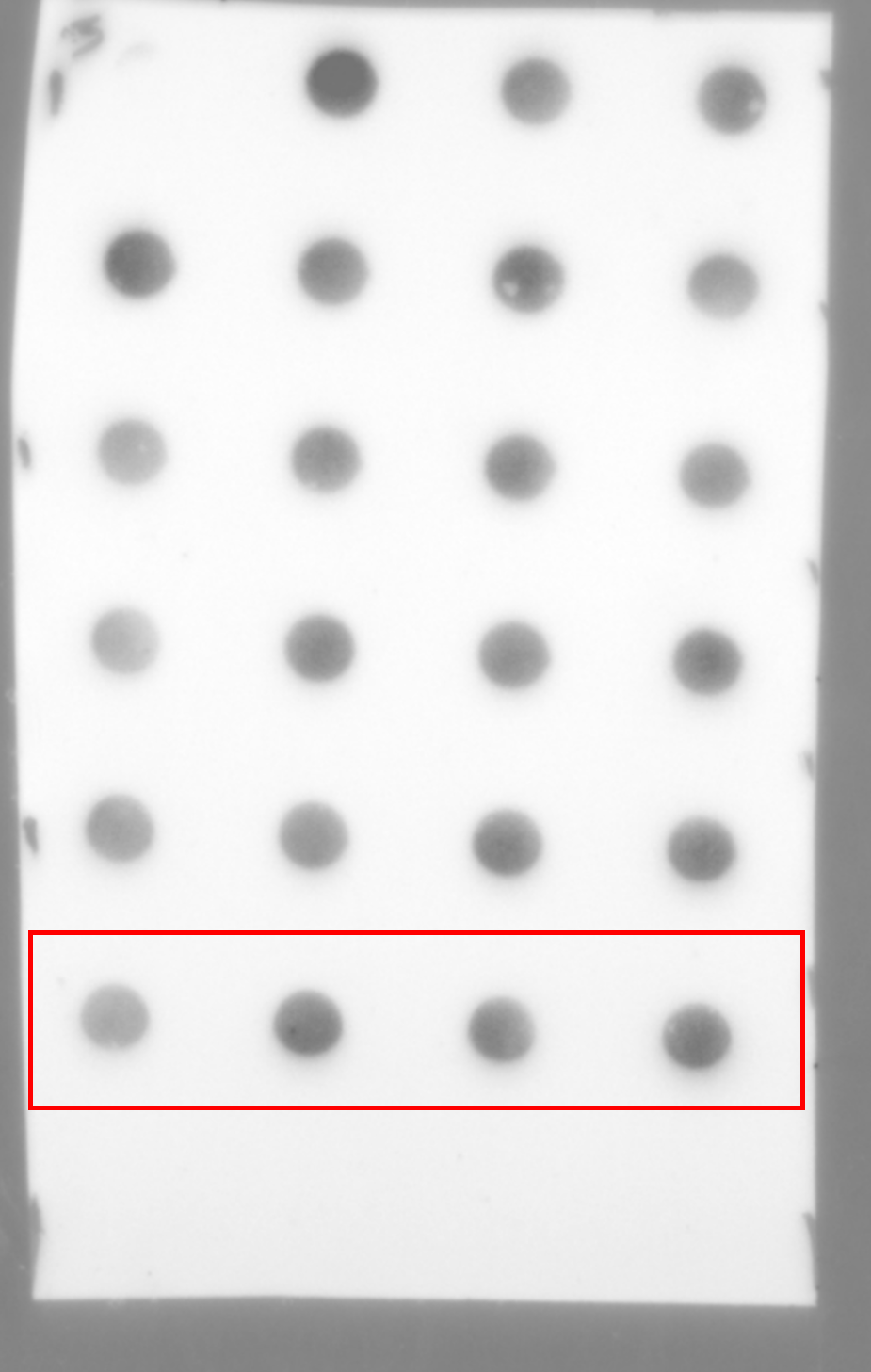

Supplement: Supplementary file 5 — Source data Fig. 3 [file 44318_2026_783_MOESM5_ESM.zip › Figure 3/Figure 3I/siCtIP_DNA.tif]

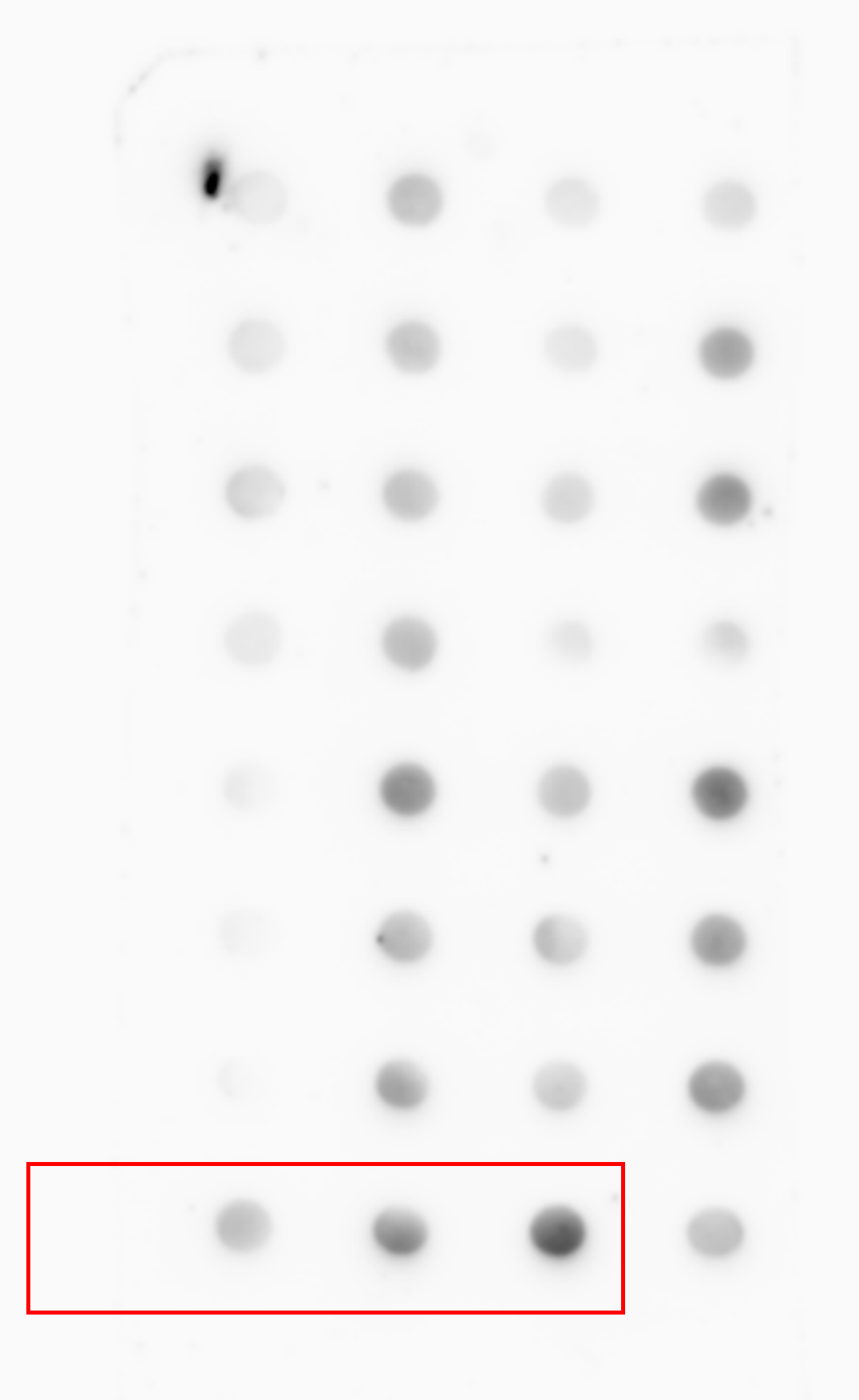

Supplement: Supplementary file 5 — Source data Fig. 3 [file 44318_2026_783_MOESM5_ESM.zip › Figure 3/Figure 3I/siCtIP_TOP1.tif]

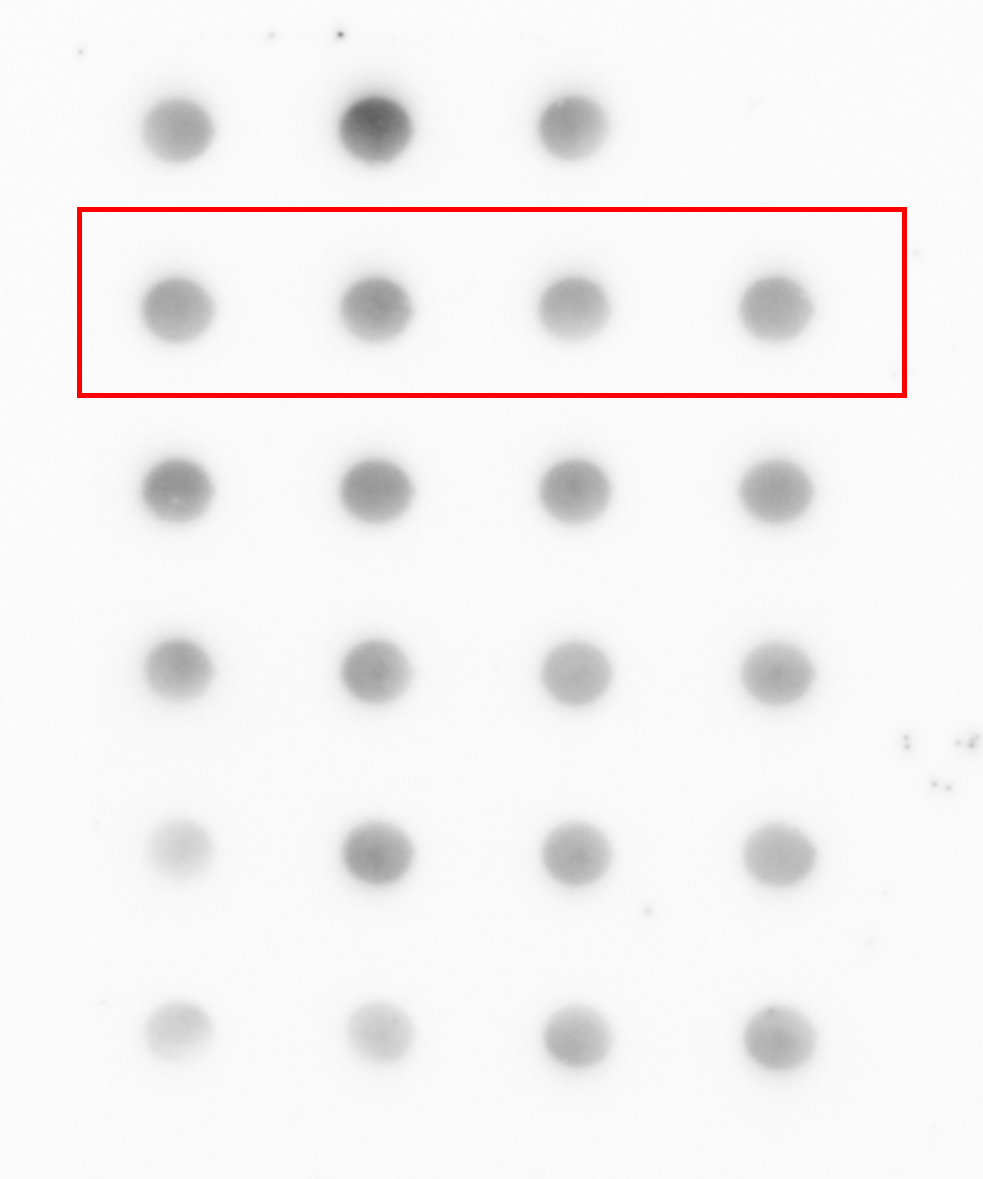

Supplement: Supplementary file 5 — Source data Fig. 3 [file 44318_2026_783_MOESM5_ESM.zip › Figure 3/Figure 3I/siCtrl_DNA.tif]

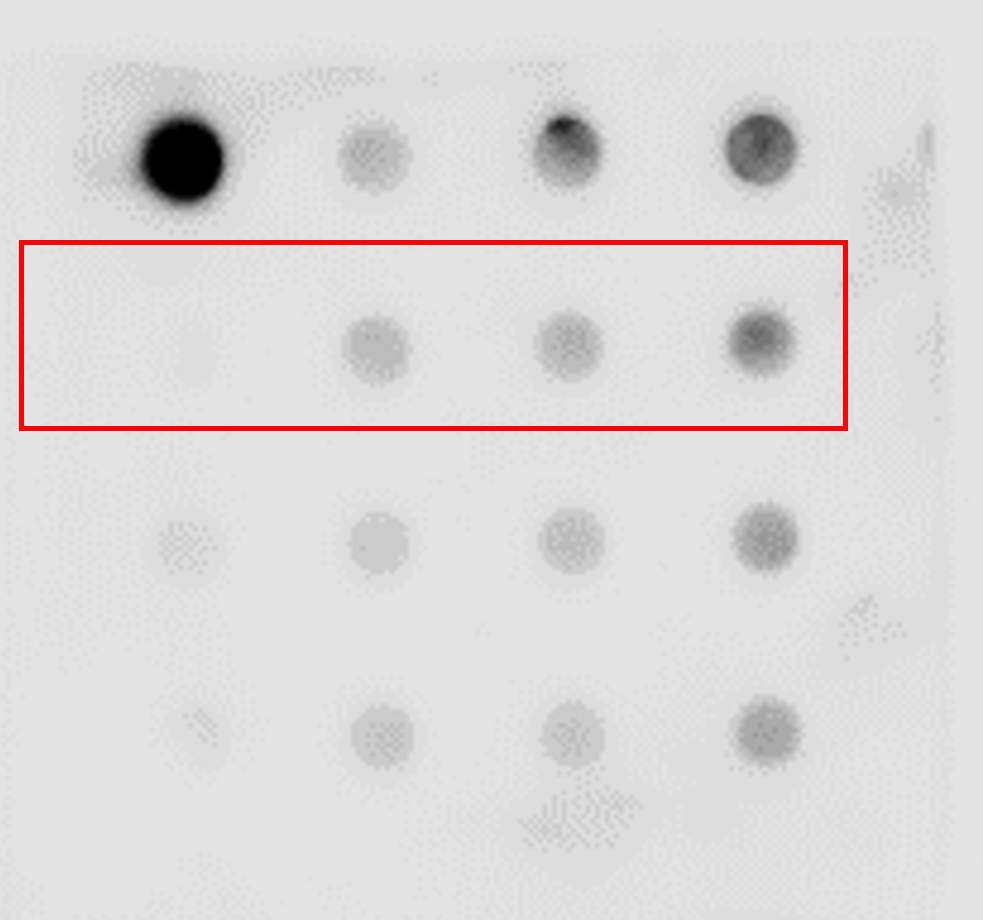

Supplement: Supplementary file 5 — Source data Fig. 3 [file 44318_2026_783_MOESM5_ESM.zip › Figure 3/Figure 3I/siCtrl_TOP1.tif]

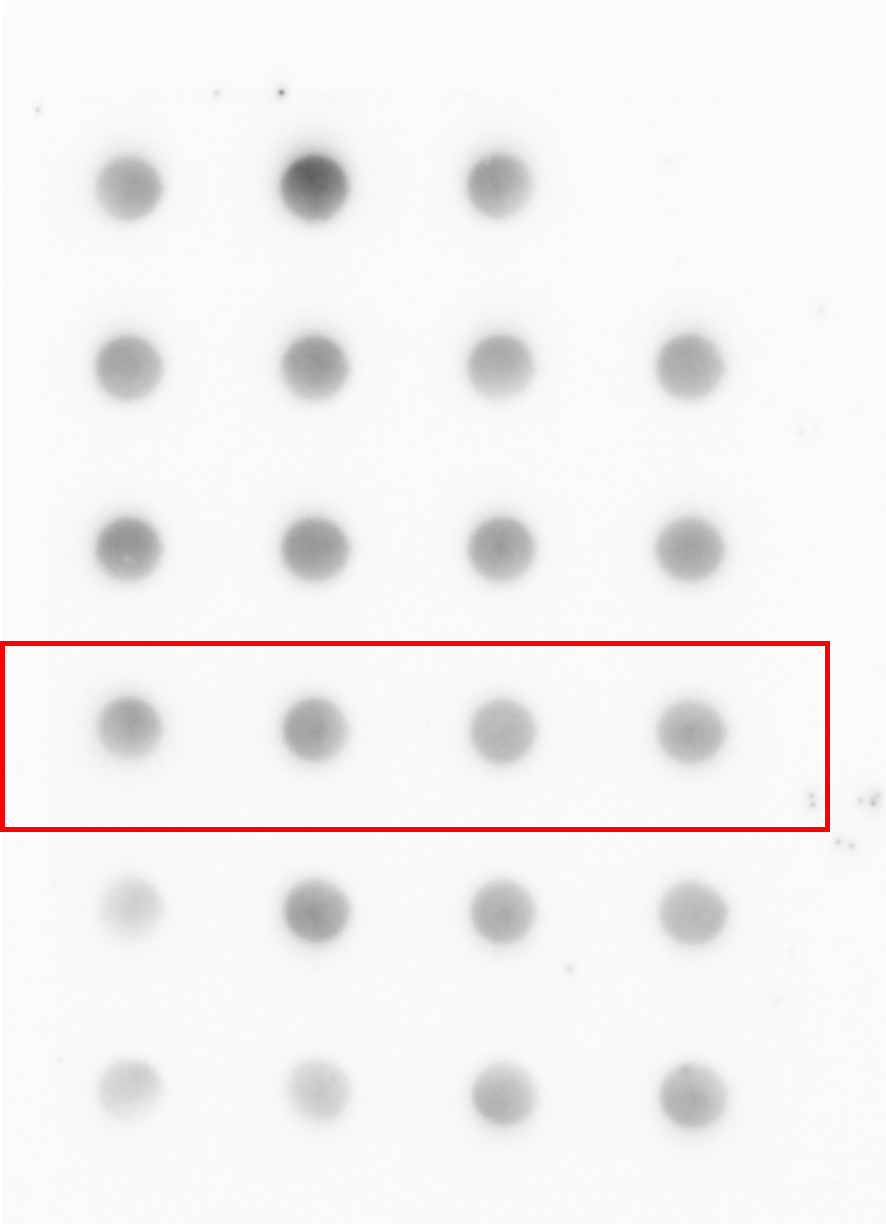

Supplement: Supplementary file 5 — Source data Fig. 3 [file 44318_2026_783_MOESM5_ESM.zip › Figure 3/Figure 3I/siEXO1_DNA.tif]

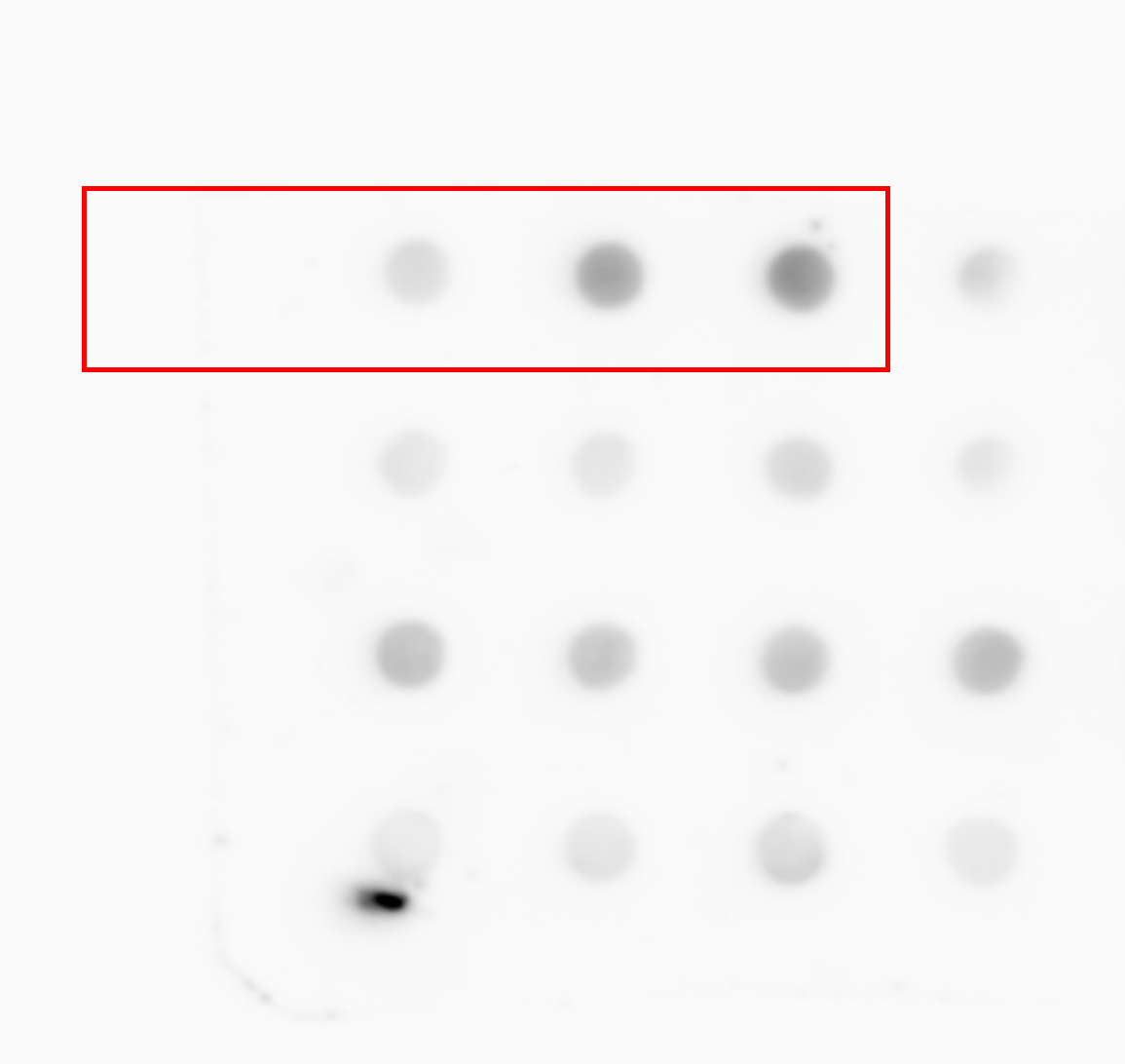

Supplement: Supplementary file 5 — Source data Fig. 3 [file 44318_2026_783_MOESM5_ESM.zip › Figure 3/Figure 3I/siEXO1_TOP1.tif]

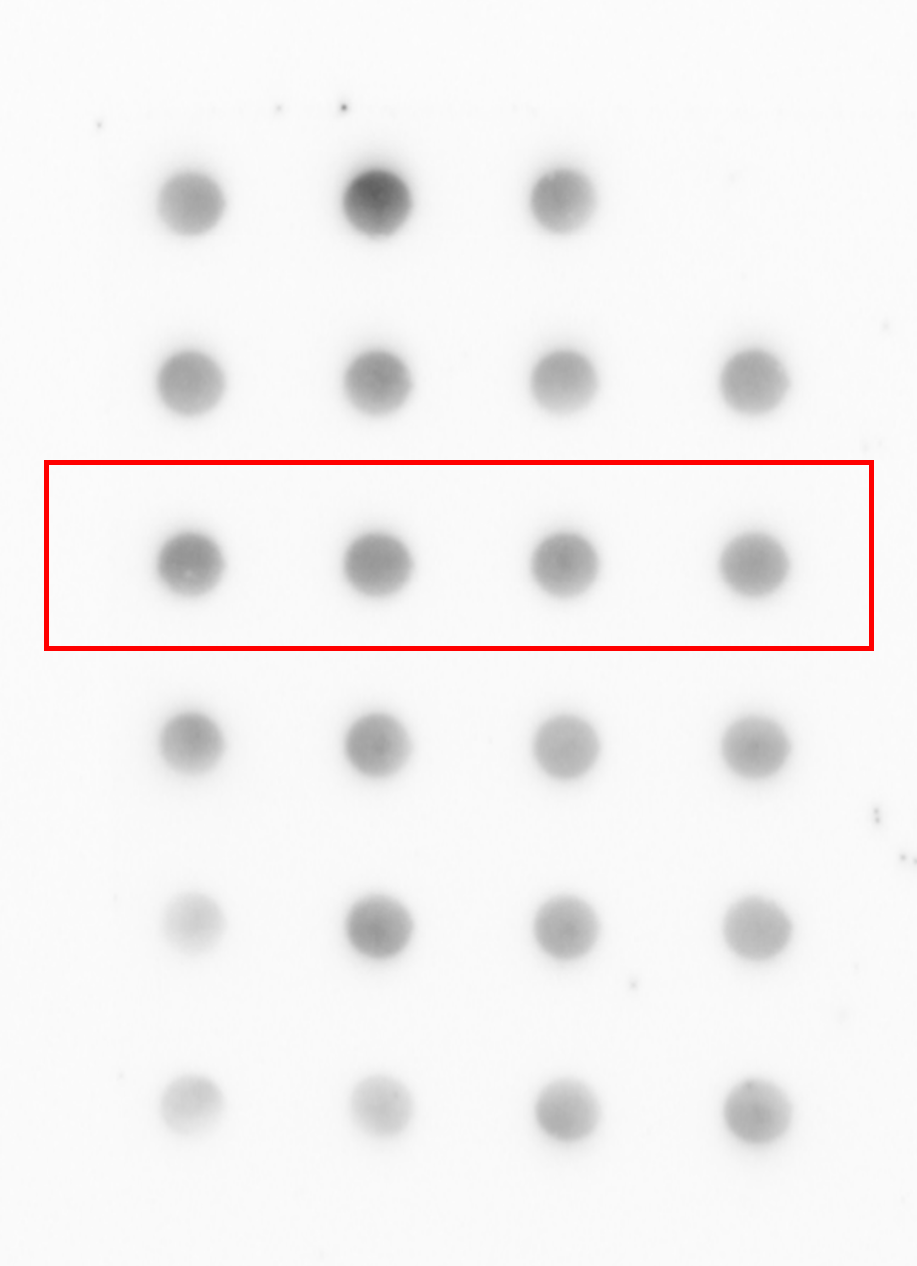

Supplement: Supplementary file 5 — Source data Fig. 3 [file 44318_2026_783_MOESM5_ESM.zip › Figure 3/Figure 3I/siMRE11_DNA.tif]

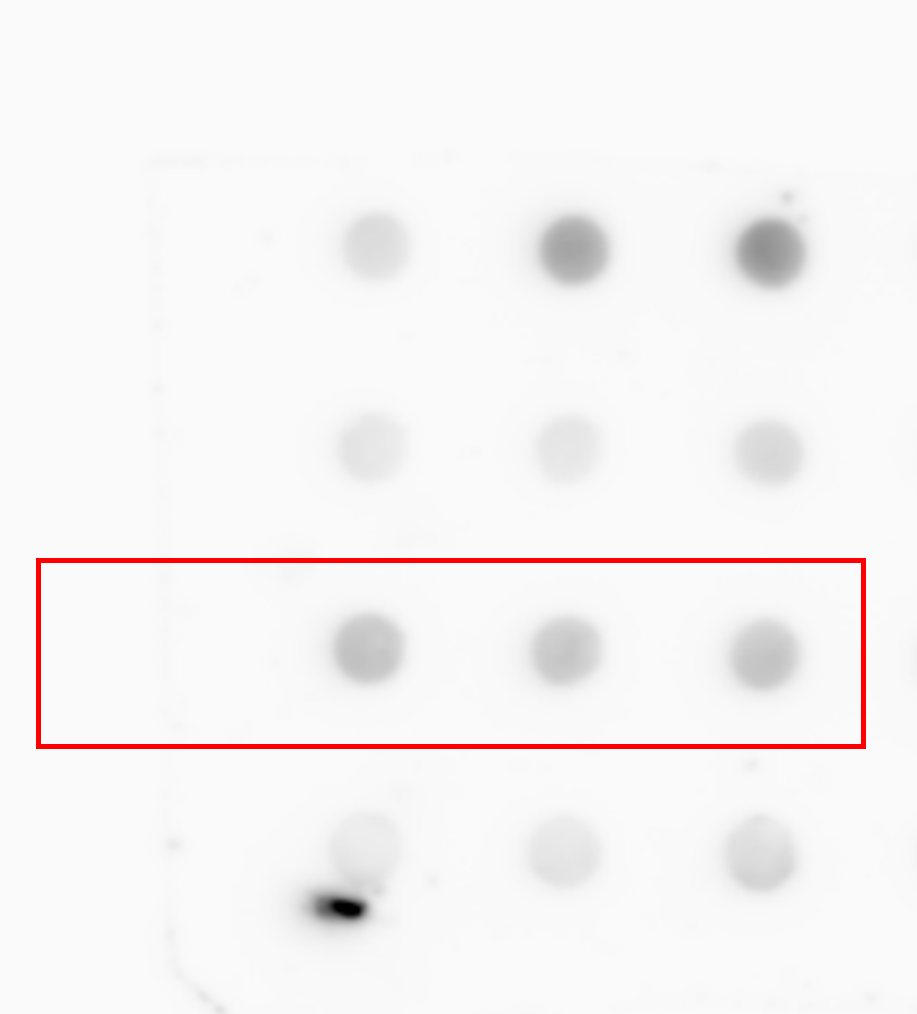

Supplement: Supplementary file 5 — Source data Fig. 3 [file 44318_2026_783_MOESM5_ESM.zip › Figure 3/Figure 3I/siMRE11_TOP1.tif]

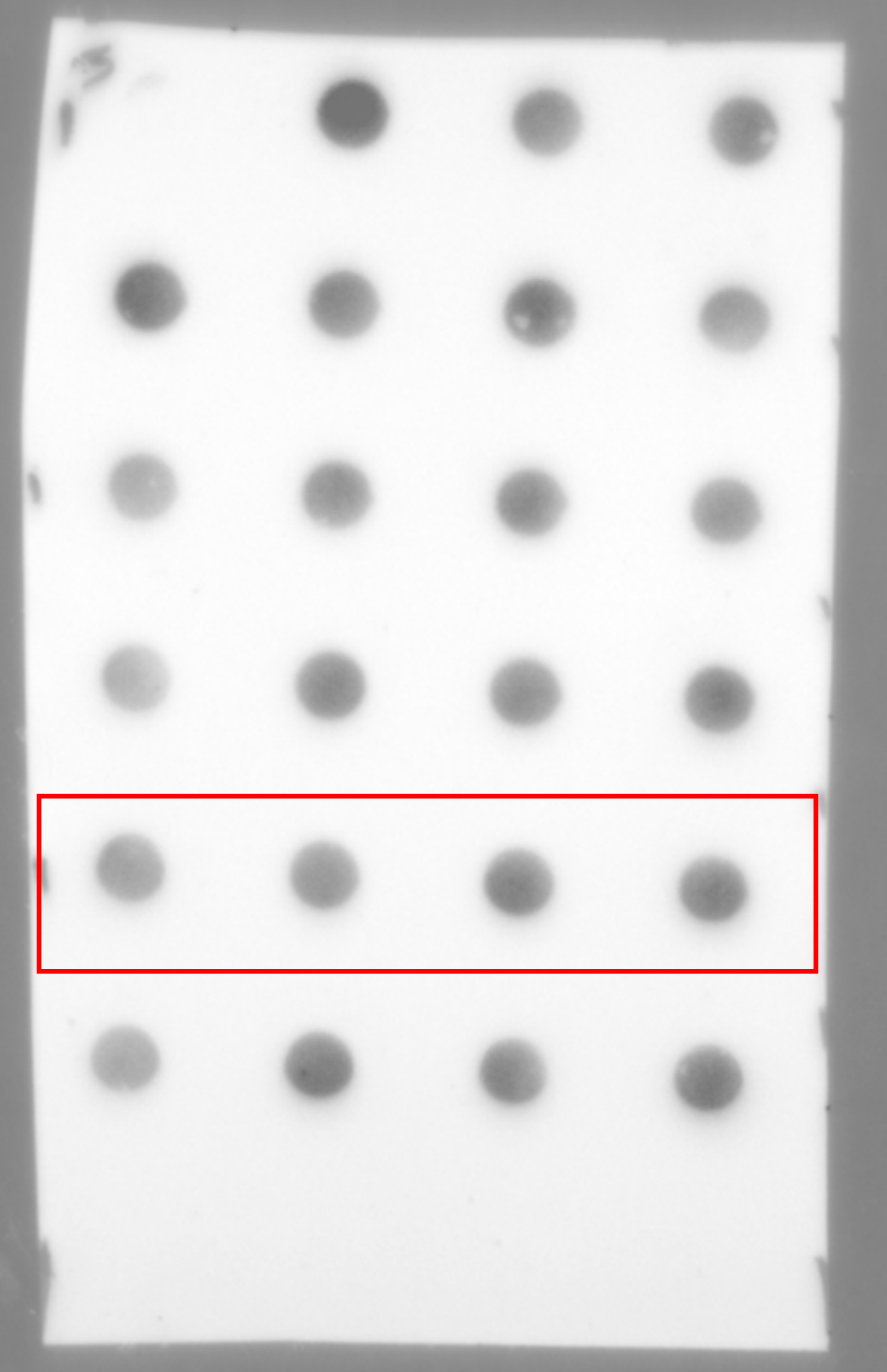

Supplement: Supplementary file 5 — Source data Fig. 3 [file 44318_2026_783_MOESM5_ESM.zip › Figure 3/Figure 3I/siTDP1_DNA.tif]

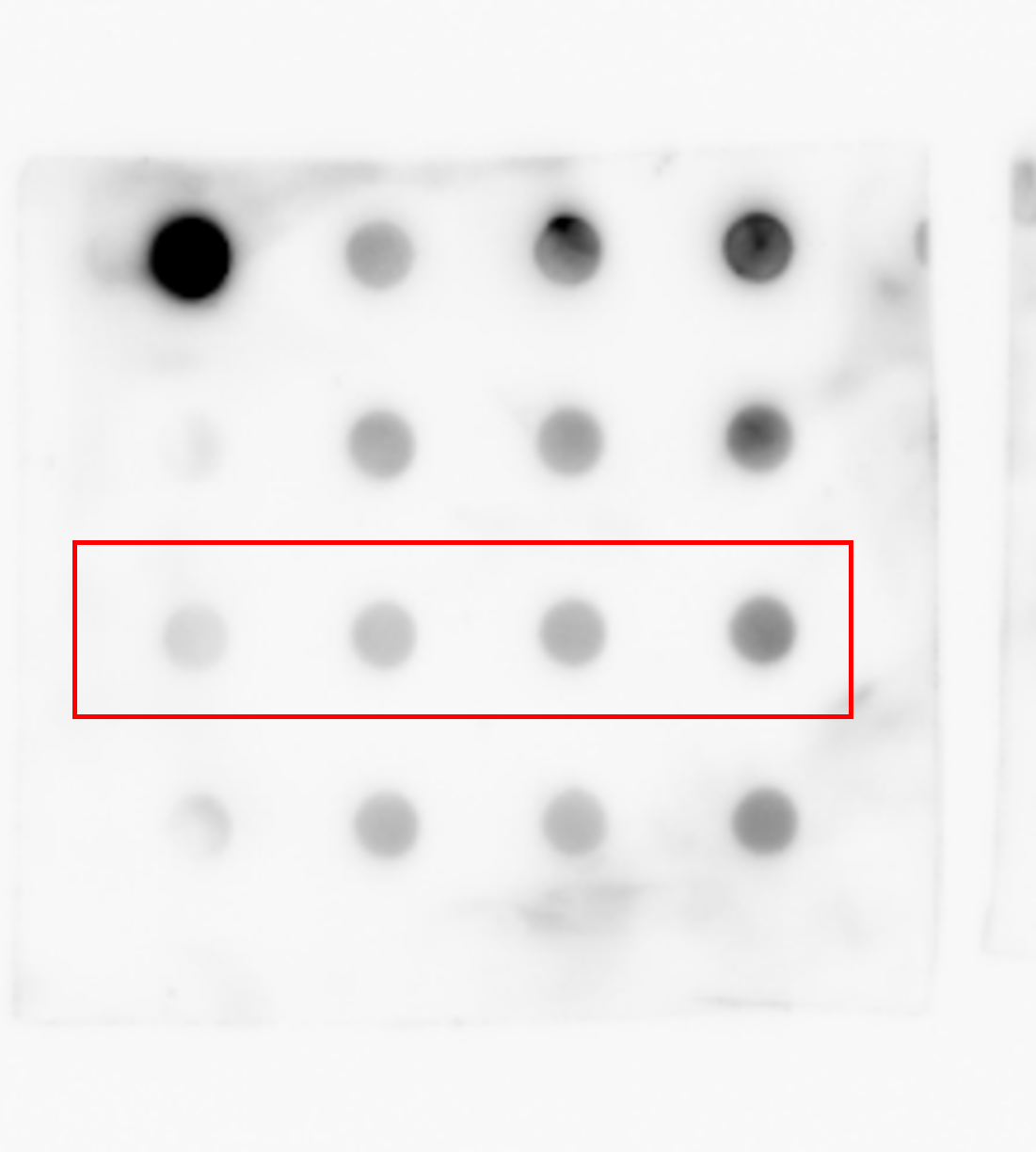

Supplement: Supplementary file 5 — Source data Fig. 3 [file 44318_2026_783_MOESM5_ESM.zip › Figure 3/Figure 3I/siTDP1_TOP1.tif]

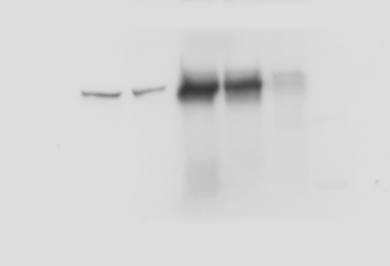

Supplement: Supplementary file 6 — Source data Fig. 4 [file 44318_2026_783_MOESM6_ESM.zip › Figure 4/Figure 4D/CHK1.tif]

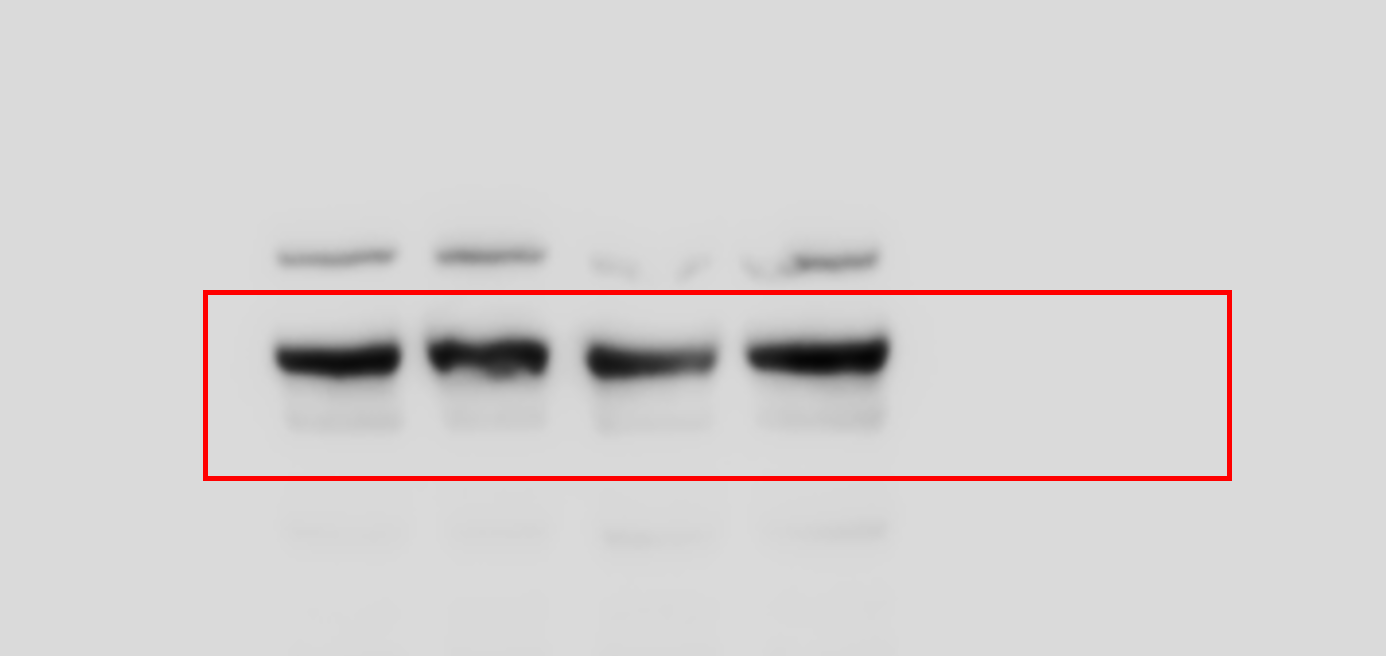

Supplement: Supplementary file 6 — Source data Fig. 4 [file 44318_2026_783_MOESM6_ESM.zip › Figure 4/Figure 4D/GFP.tif]

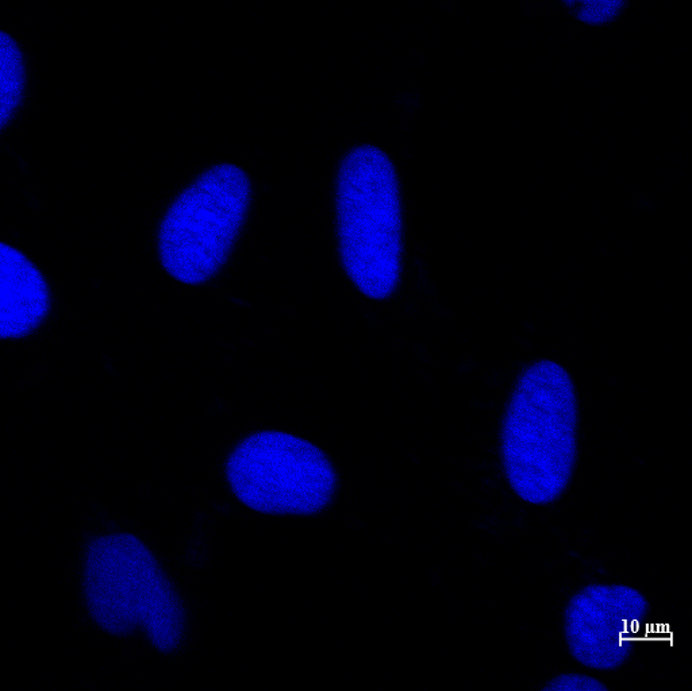

Supplement: Supplementary file 6 — Source data Fig. 4 [file 44318_2026_783_MOESM6_ESM.zip › Figure 4/Figure 4E/Antibody Control_CHK1_Hoechst.tif]

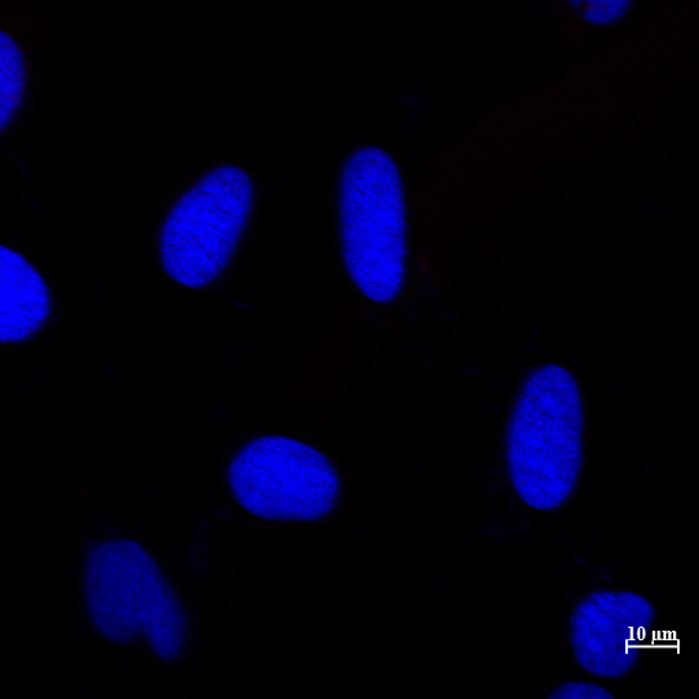

Supplement: Supplementary file 6 — Source data Fig. 4 [file 44318_2026_783_MOESM6_ESM.zip › Figure 4/Figure 4E/Antibody Control_CHK1_Merged.tif]

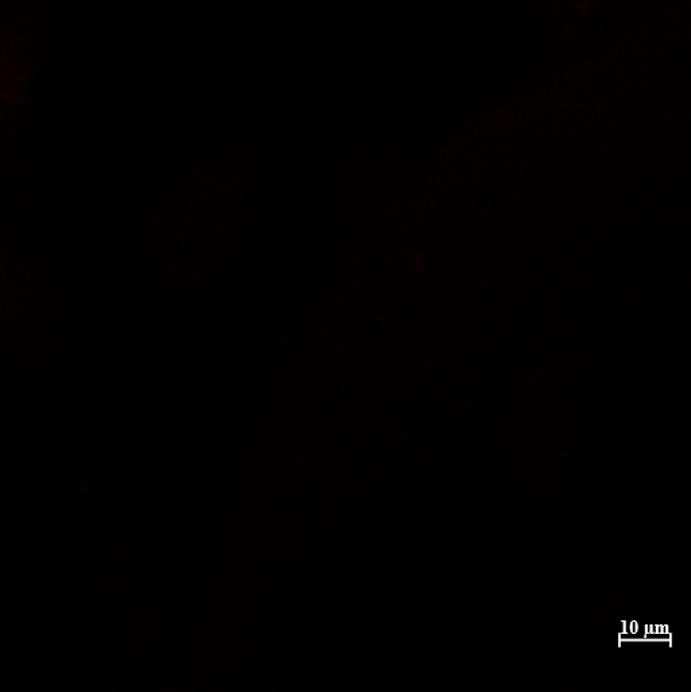

Supplement: Supplementary file 6 — Source data Fig. 4 [file 44318_2026_783_MOESM6_ESM.zip › Figure 4/Figure 4E/Antibody Control_CHK1_PLA.tif]

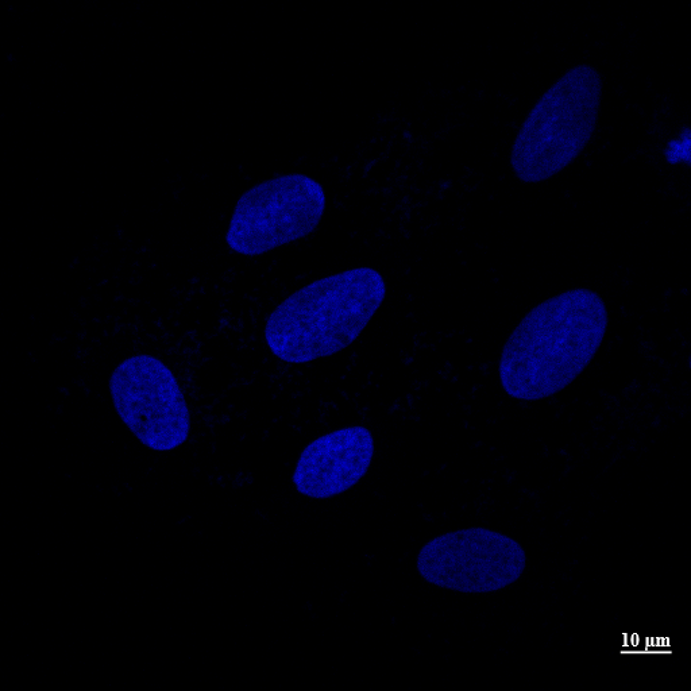

Supplement: Supplementary file 6 — Source data Fig. 4 [file 44318_2026_783_MOESM6_ESM.zip › Figure 4/Figure 4E/Antibody Control_TOP1_Hoechst.tif]

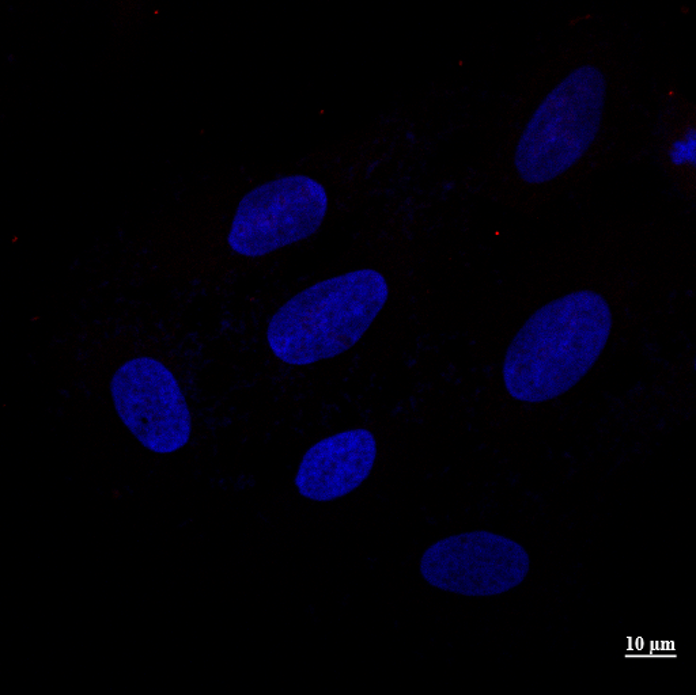

Supplement: Supplementary file 6 — Source data Fig. 4 [file 44318_2026_783_MOESM6_ESM.zip › Figure 4/Figure 4E/Antibody Control_TOP1_Merged.tif]

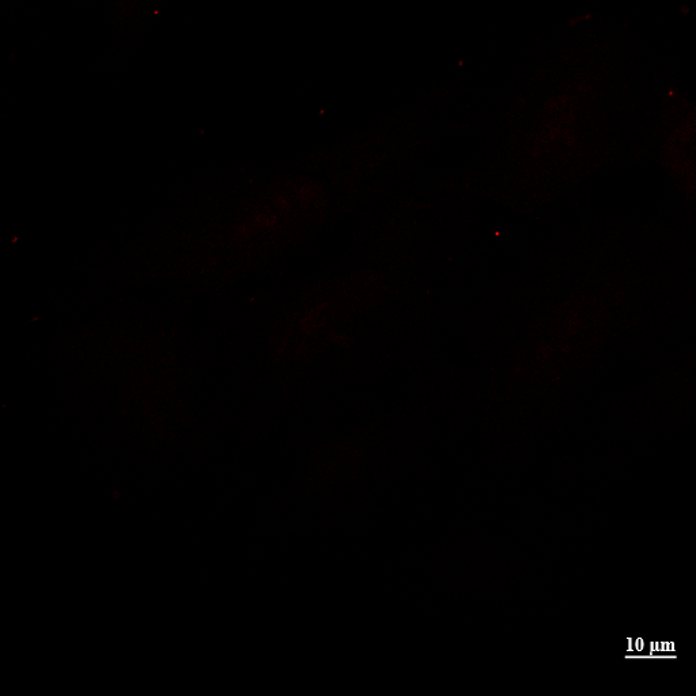

Supplement: Supplementary file 6 — Source data Fig. 4 [file 44318_2026_783_MOESM6_ESM.zip › Figure 4/Figure 4E/Antibody Control_TOP1_PLA.tif]

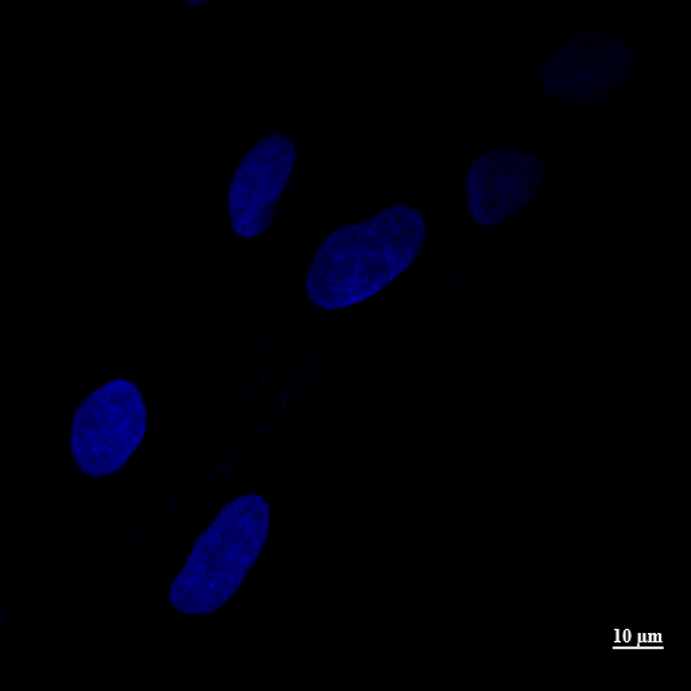

Supplement: Supplementary file 6 — Source data Fig. 4 [file 44318_2026_783_MOESM6_ESM.zip › Figure 4/Figure 4E/TOP1_CHK1_CHK1i_Hoechst.tif]

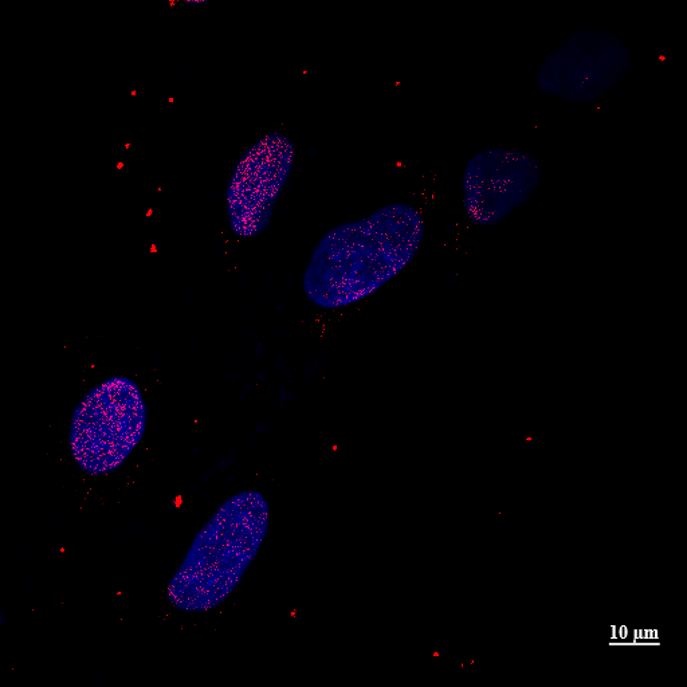

Supplement: Supplementary file 6 — Source data Fig. 4 [file 44318_2026_783_MOESM6_ESM.zip › Figure 4/Figure 4E/TOP1_CHK1_CHK1i_Merged.tif]

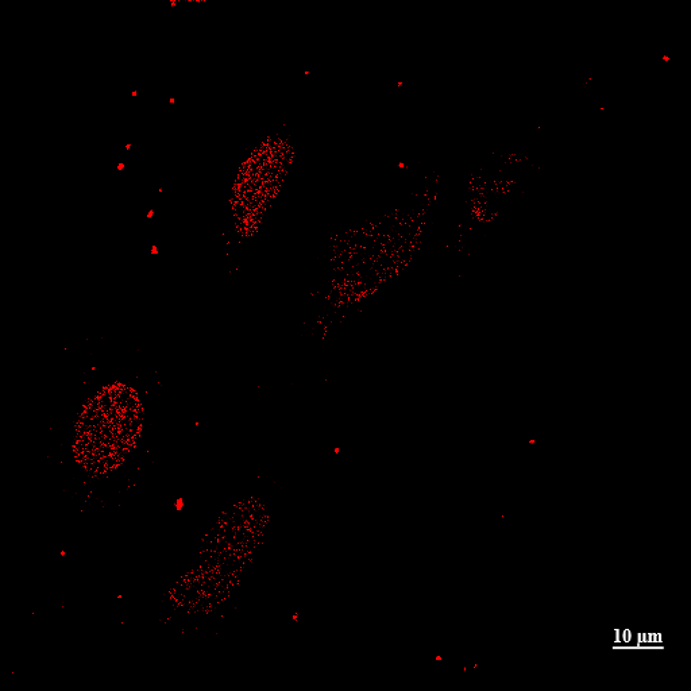

Supplement: Supplementary file 6 — Source data Fig. 4 [file 44318_2026_783_MOESM6_ESM.zip › Figure 4/Figure 4E/TOP1_CHK1_CHK1i_PLA.tif]

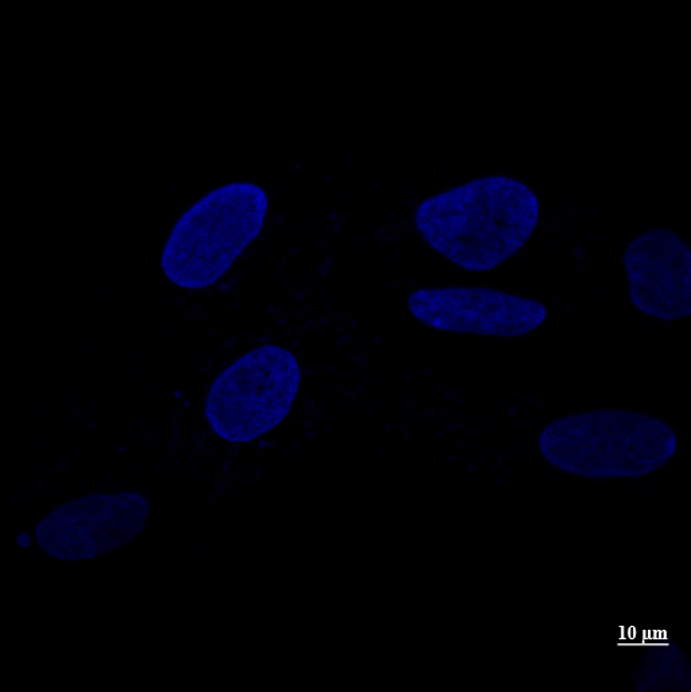

Supplement: Supplementary file 6 — Source data Fig. 4 [file 44318_2026_783_MOESM6_ESM.zip › Figure 4/Figure 4E/TOP1_CHK1_Untreated_Hoechst.tif]

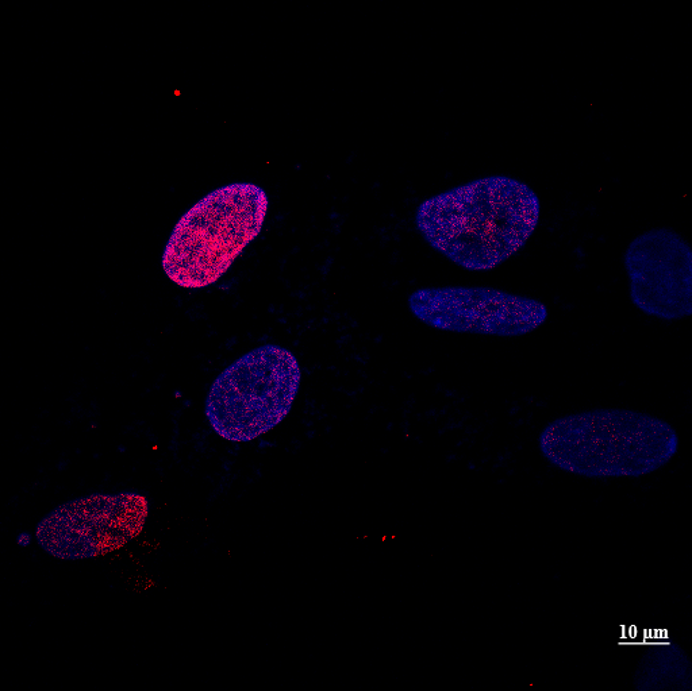

Supplement: Supplementary file 6 — Source data Fig. 4 [file 44318_2026_783_MOESM6_ESM.zip › Figure 4/Figure 4E/TOP1_CHK1_Untreated_Merged.tif]

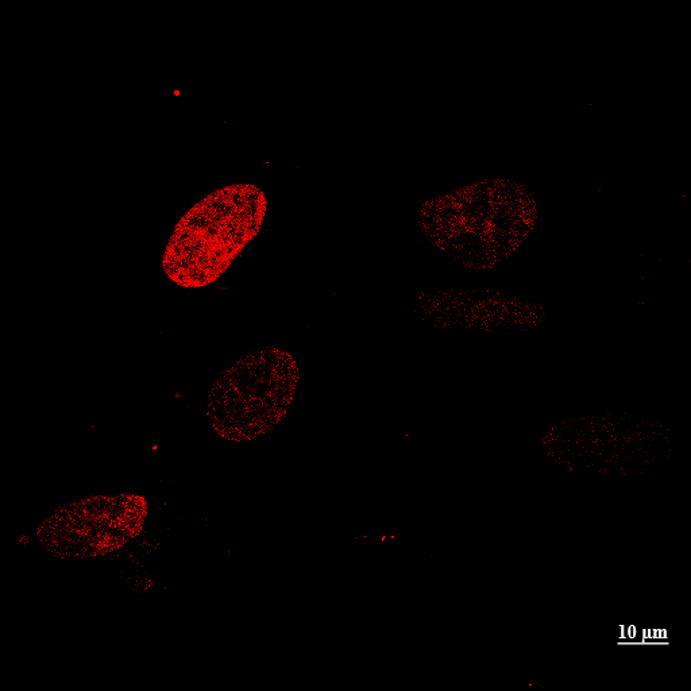

Supplement: Supplementary file 6 — Source data Fig. 4 [file 44318_2026_783_MOESM6_ESM.zip › Figure 4/Figure 4E/TOP1_CHK1_Untreated_PLA.tif]

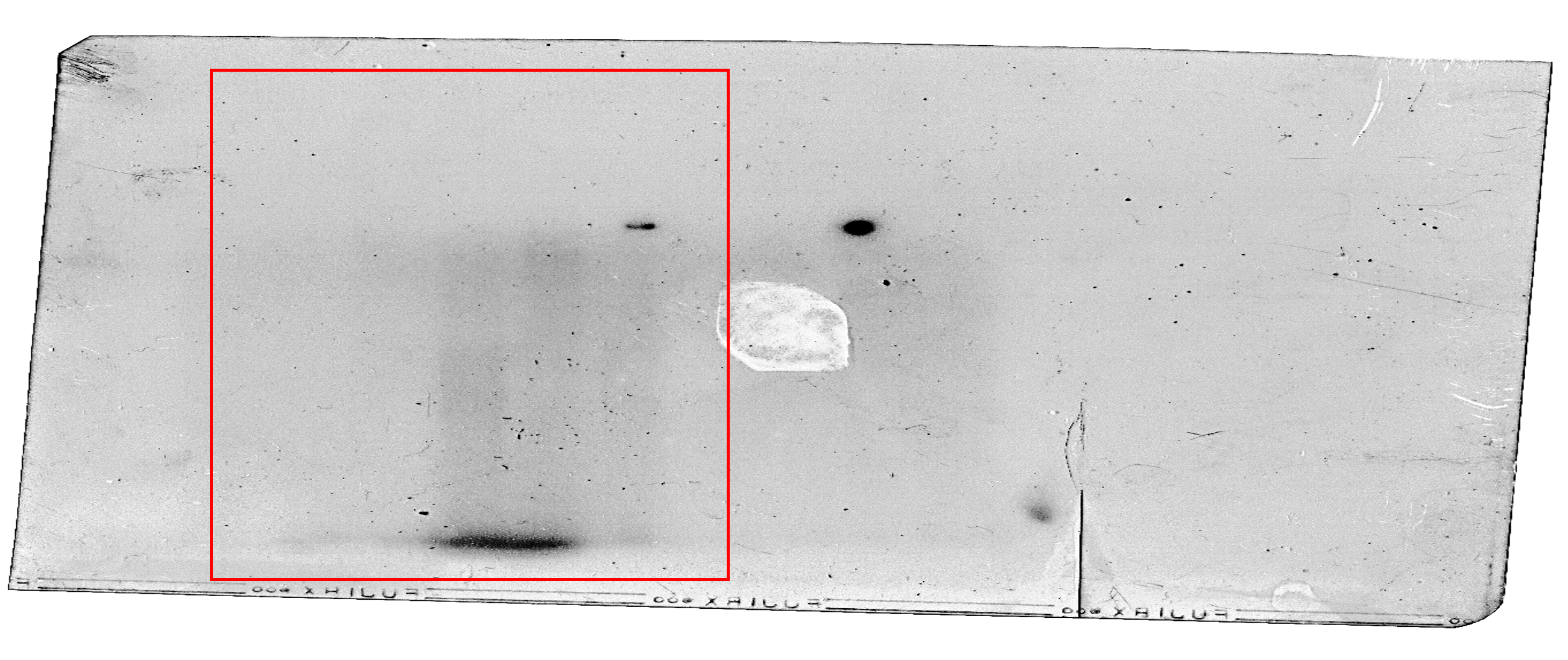

Supplement: Supplementary file 6 — Source data Fig. 4 [file 44318_2026_783_MOESM6_ESM.zip › Figure 4/Figure 4G/Autoradiograph.tif]

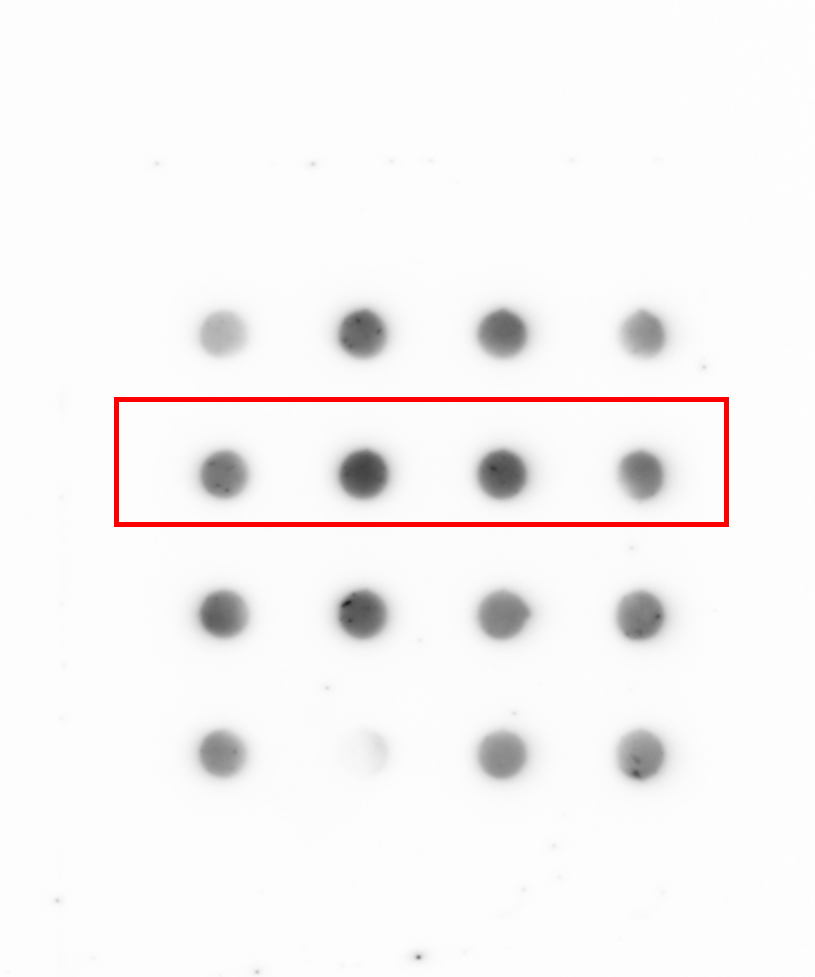

Supplement: Supplementary file 7 — Source data Fig. 5 [file 44318_2026_783_MOESM7_ESM.zip › Figure 5/Figure 5D/S320A_DNA.tif]

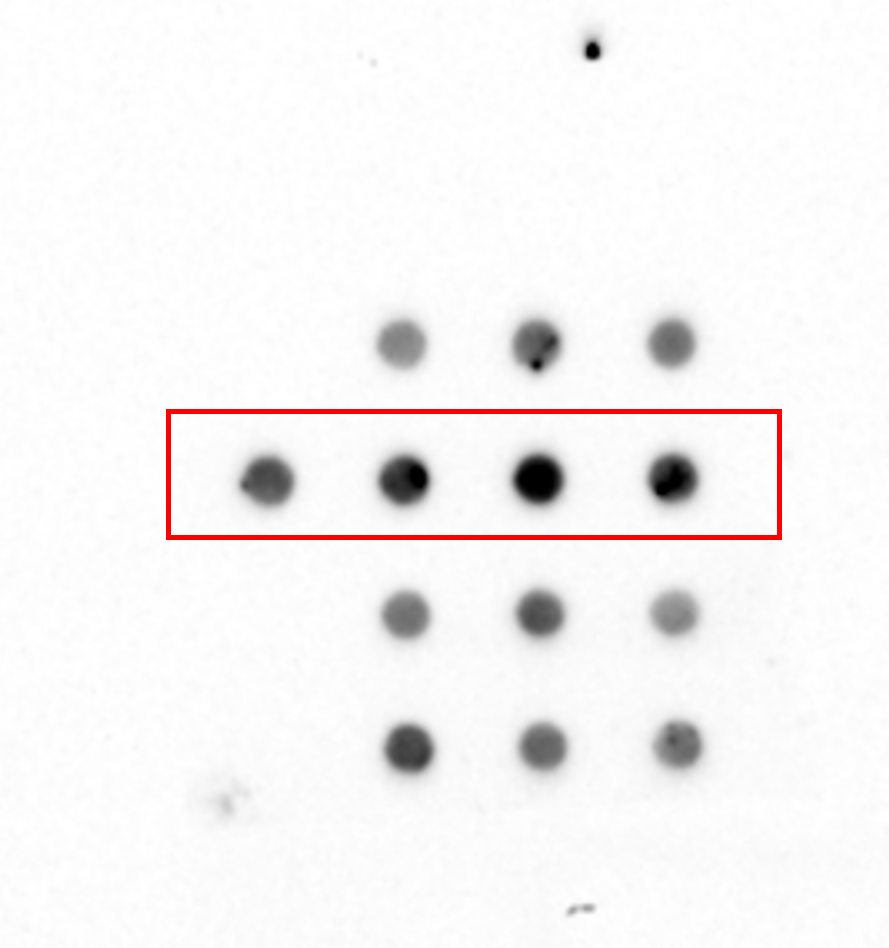

Supplement: Supplementary file 7 — Source data Fig. 5 [file 44318_2026_783_MOESM7_ESM.zip › Figure 5/Figure 5D/S320A_GFP.tif]

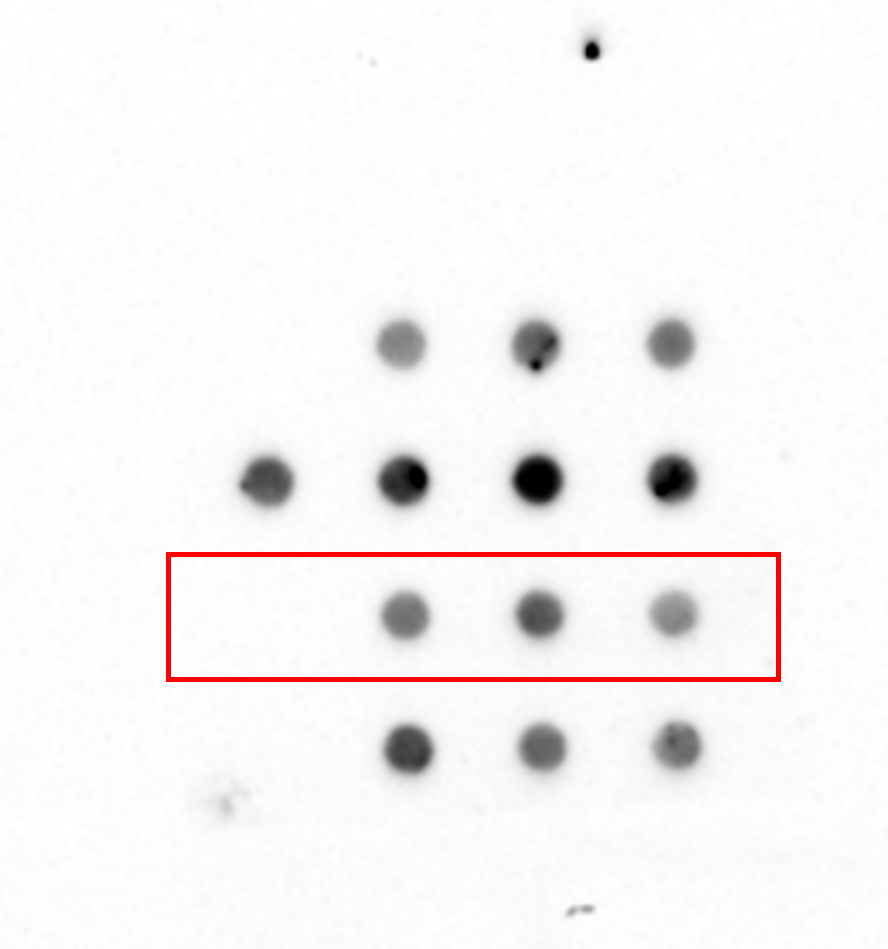

Supplement: Supplementary file 7 — Source data Fig. 5 [file 44318_2026_783_MOESM7_ESM.zip › Figure 5/Figure 5D/S394A_GFP.tif]

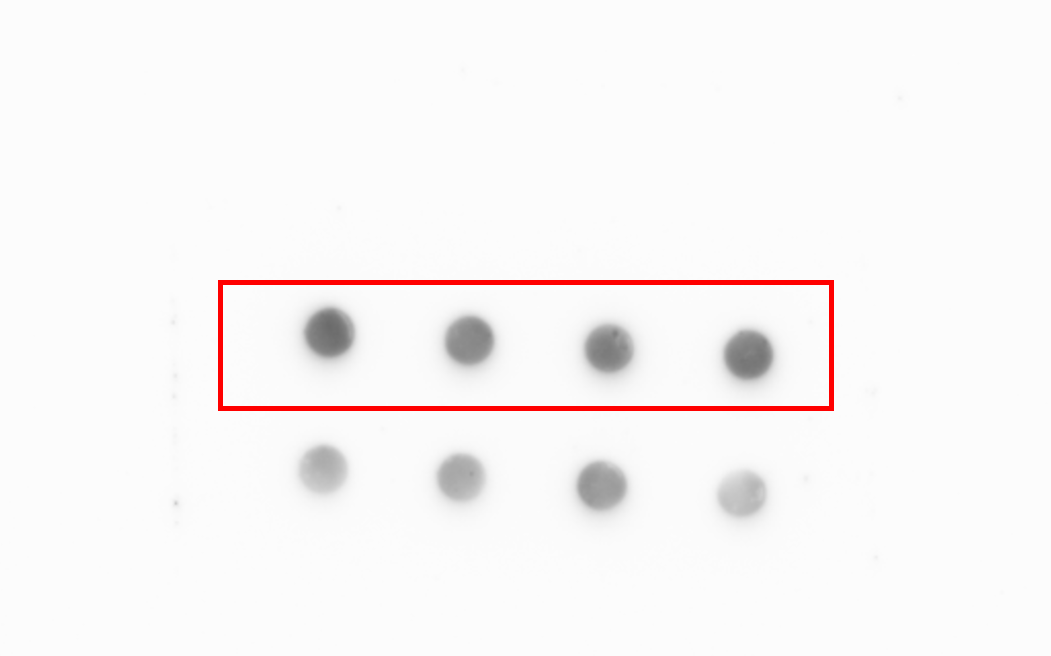

Supplement: Supplementary file 7 — Source data Fig. 5 [file 44318_2026_783_MOESM7_ESM.zip › Figure 5/Figure 5D/T570A_DNA.tif]

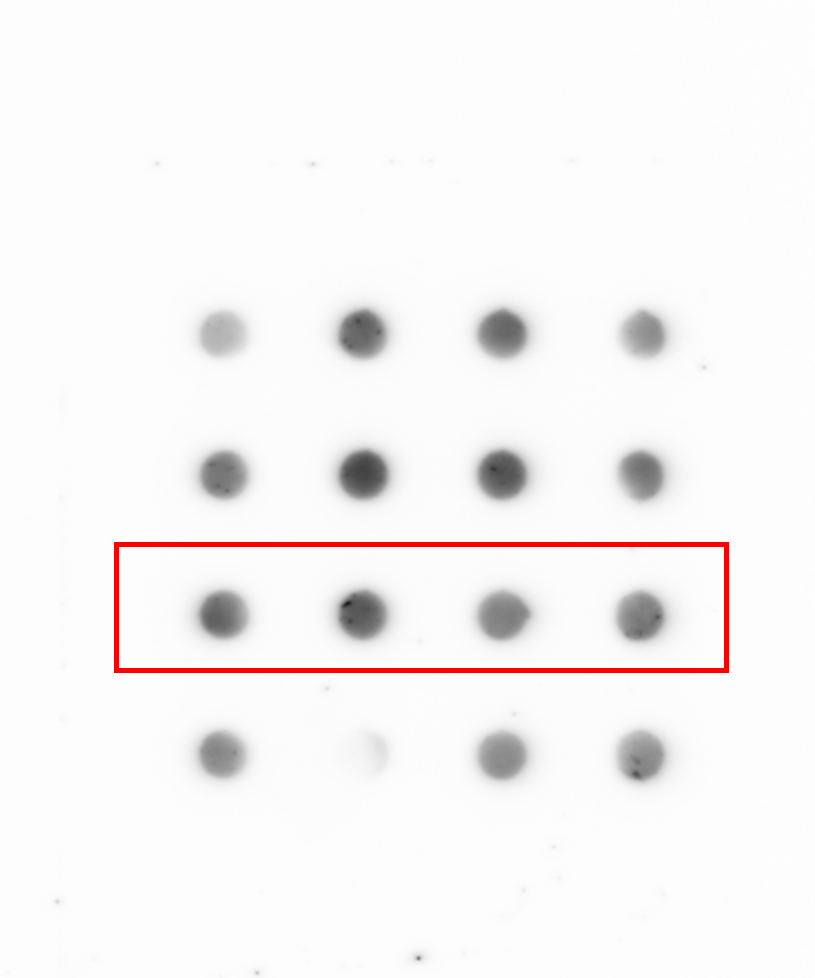

Supplement: Supplementary file 7 — Source data Fig. 5 [file 44318_2026_783_MOESM7_ESM.zip › Figure 5/Figure 5D/T570A_GFP.tif]

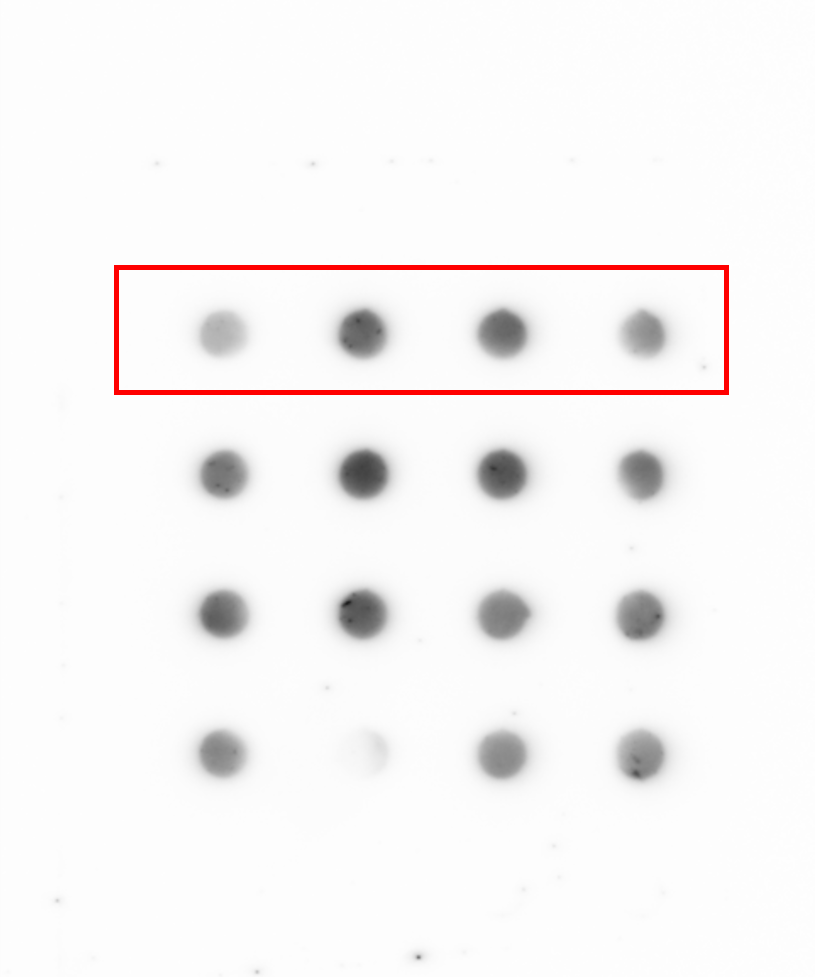

Supplement: Supplementary file 7 — Source data Fig. 5 [file 44318_2026_783_MOESM7_ESM.zip › Figure 5/Figure 5D/WT_DNA.tif]

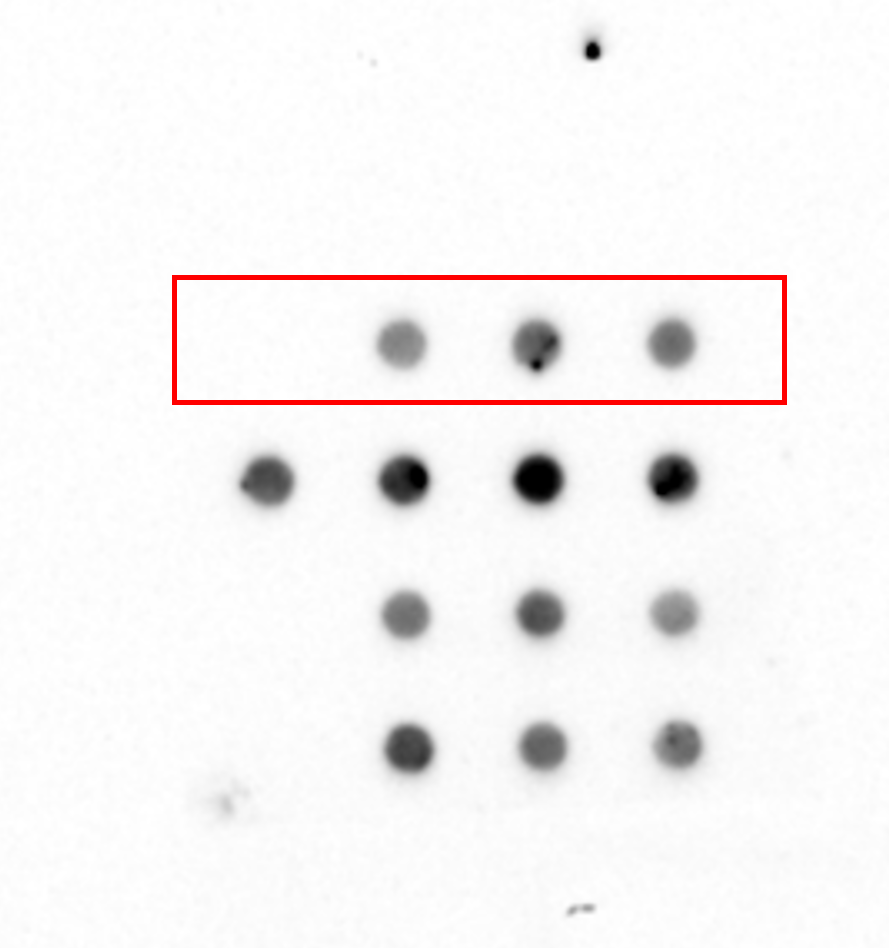

Supplement: Supplementary file 7 — Source data Fig. 5 [file 44318_2026_783_MOESM7_ESM.zip › Figure 5/Figure 5D/WT_GFP.tif]

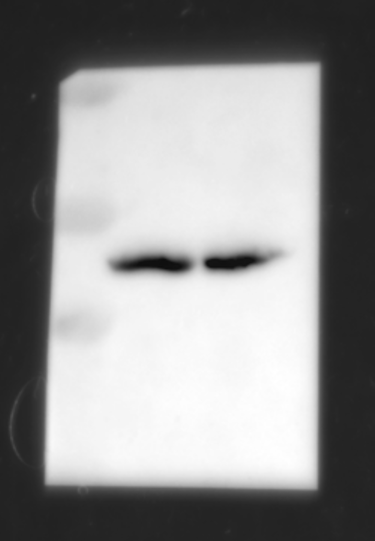

Supplement: Supplementary file 7 — Source data Fig. 5 [file 44318_2026_783_MOESM7_ESM.zip › Figure 5/Figure 5E/Beta Actin.tif]

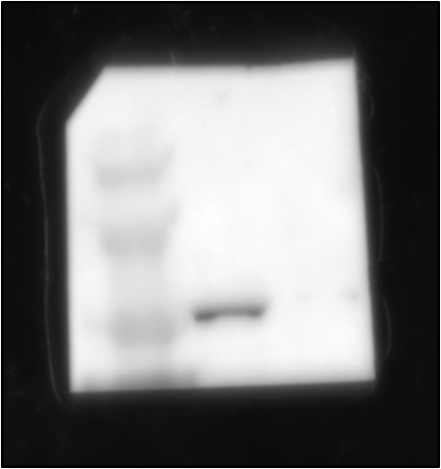

Supplement: Supplementary file 7 — Source data Fig. 5 [file 44318_2026_783_MOESM7_ESM.zip › Figure 5/Figure 5E/TOP1.tif]

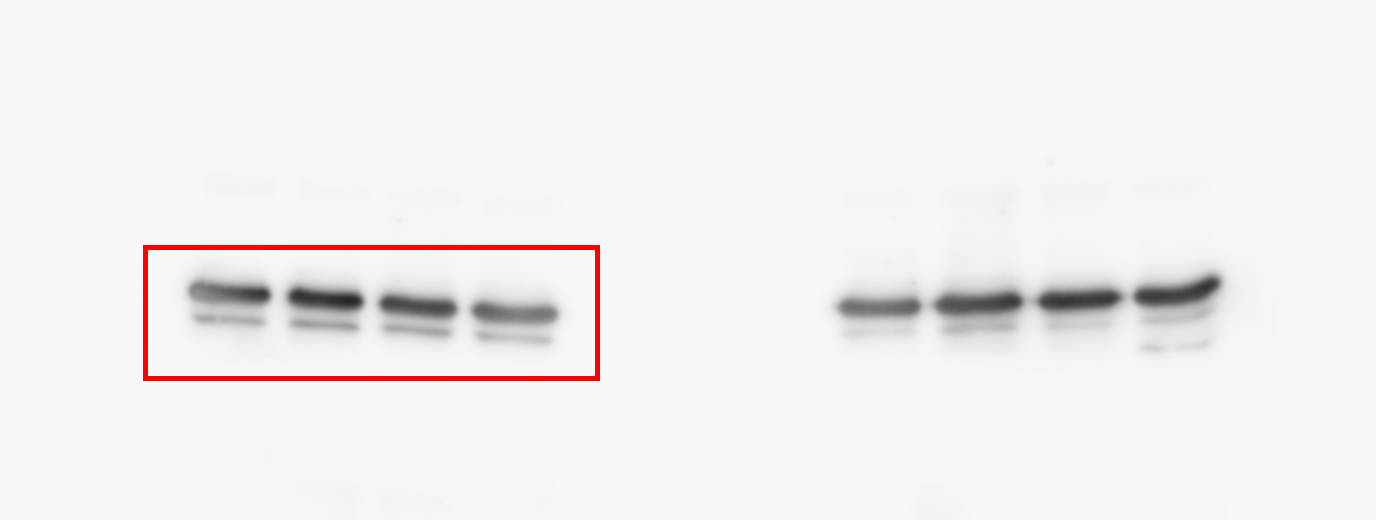

Supplement: Supplementary file 7 — Source data Fig. 5 [file 44318_2026_783_MOESM7_ESM.zip › Figure 5/Figure 5F/GAPDH.tif]

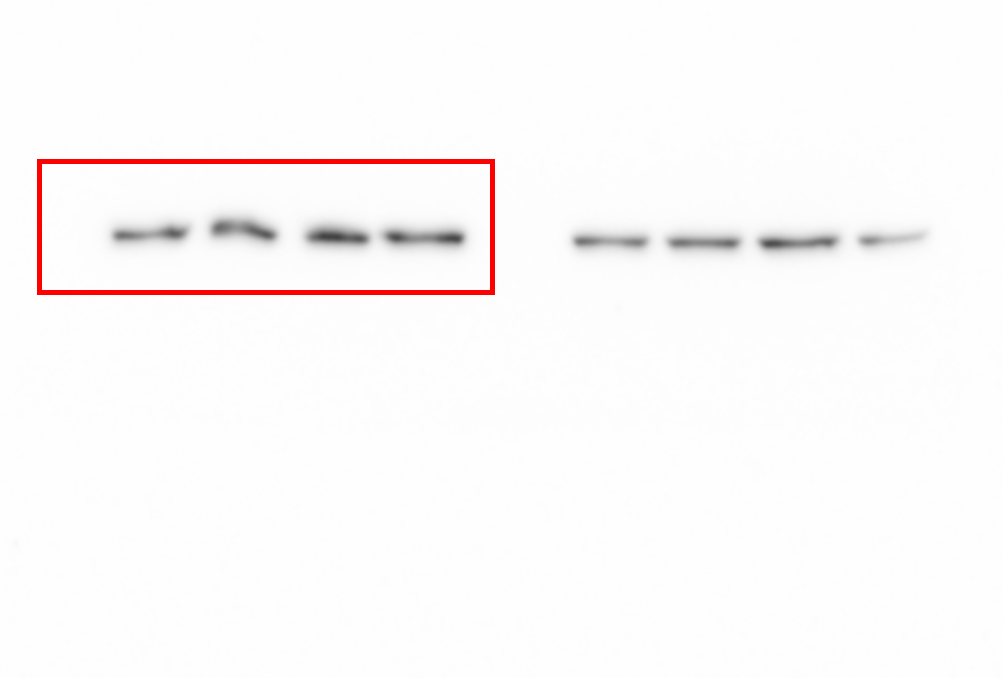

Supplement: Supplementary file 7 — Source data Fig. 5 [file 44318_2026_783_MOESM7_ESM.zip › Figure 5/Figure 5F/GFP.tif]

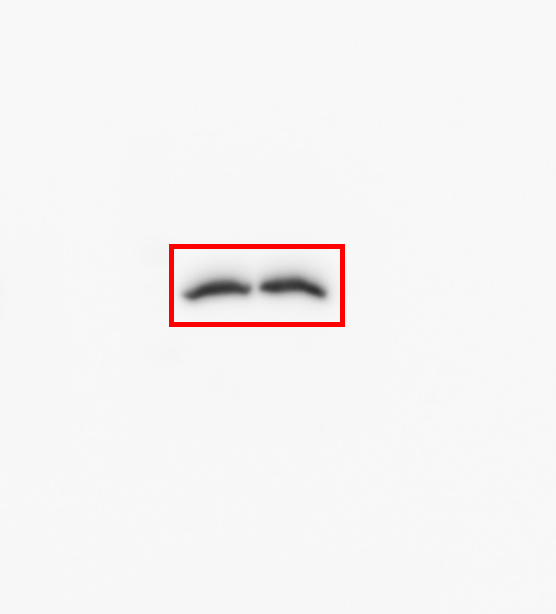

Supplement: Supplementary file 7 — Source data Fig. 5 [file 44318_2026_783_MOESM7_ESM.zip › Figure 5/Figure 5H/Input_S320A_(Beta Actin Blot).tif]

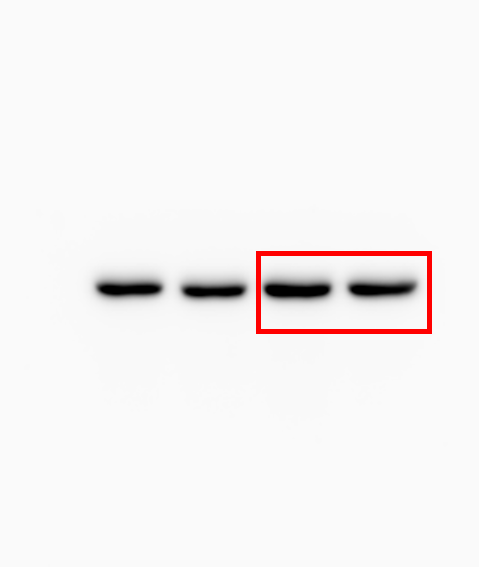

Supplement: Supplementary file 7 — Source data Fig. 5 [file 44318_2026_783_MOESM7_ESM.zip › Figure 5/Figure 5H/Input_S320A_(GFP Blot).tif]

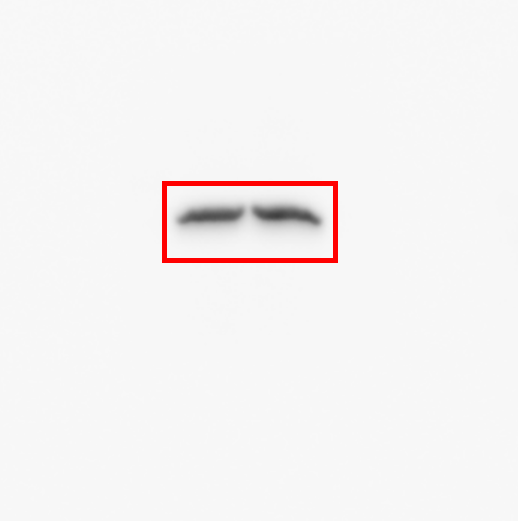

Supplement: Supplementary file 7 — Source data Fig. 5 [file 44318_2026_783_MOESM7_ESM.zip › Figure 5/Figure 5H/Input_WT_(Beta Actin Blot).tif]

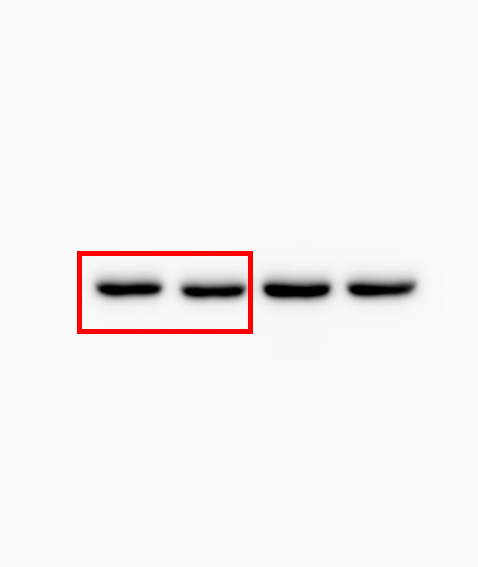

Supplement: Supplementary file 7 — Source data Fig. 5 [file 44318_2026_783_MOESM7_ESM.zip › Figure 5/Figure 5H/Input_WT_(GFP Blot).tif]

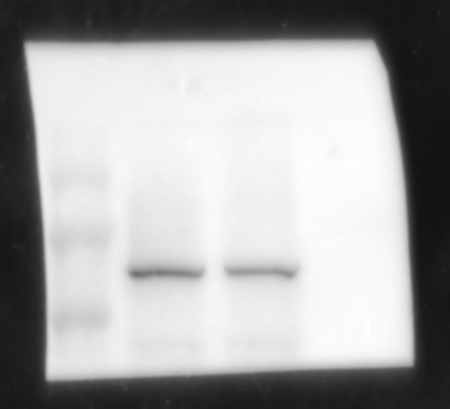

Supplement: Supplementary file 7 — Source data Fig. 5 [file 44318_2026_783_MOESM7_ESM.zip › Figure 5/Figure 5H/IP_S320A_(GFP Blot).tif]

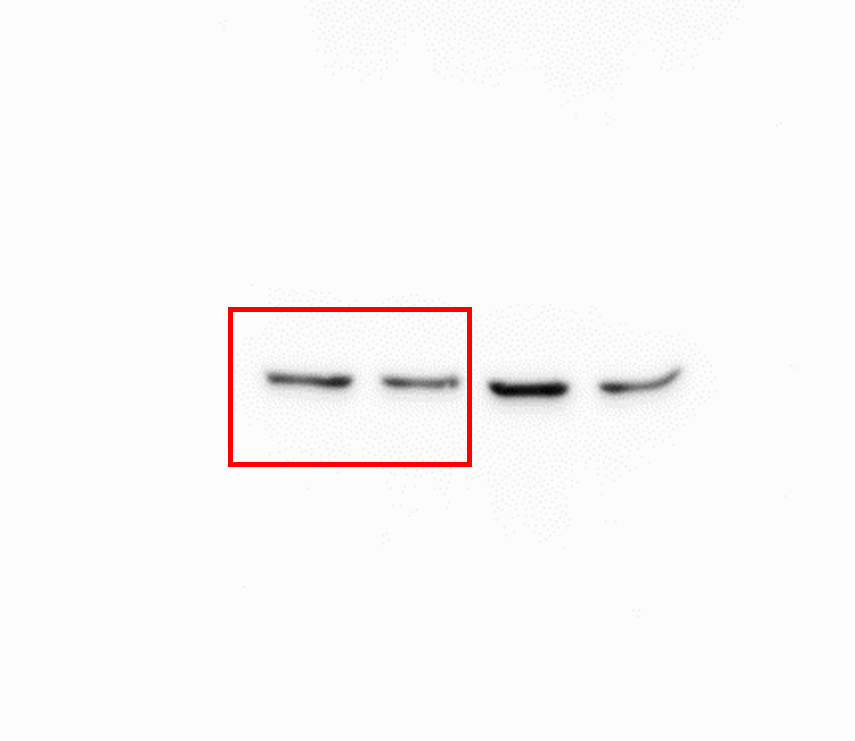

Supplement: Supplementary file 7 — Source data Fig. 5 [file 44318_2026_783_MOESM7_ESM.zip › Figure 5/Figure 5H/IP_S320A_(Phosphoserine Blot).tif]

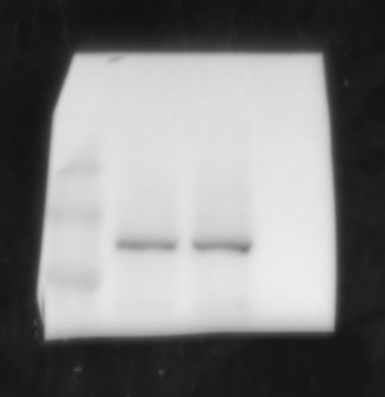

Supplement: Supplementary file 7 — Source data Fig. 5 [file 44318_2026_783_MOESM7_ESM.zip › Figure 5/Figure 5H/IP_WT_(GFP Blot).tif]

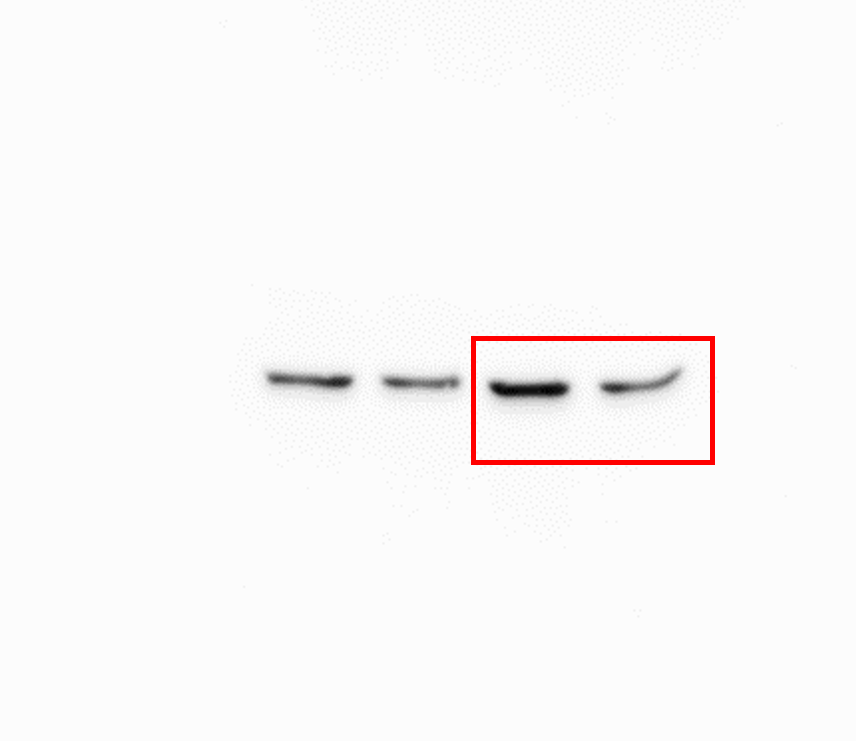

Supplement: Supplementary file 7 — Source data Fig. 5 [file 44318_2026_783_MOESM7_ESM.zip › Figure 5/Figure 5H/IP_WT_(Phosphoserine Blot).tif]

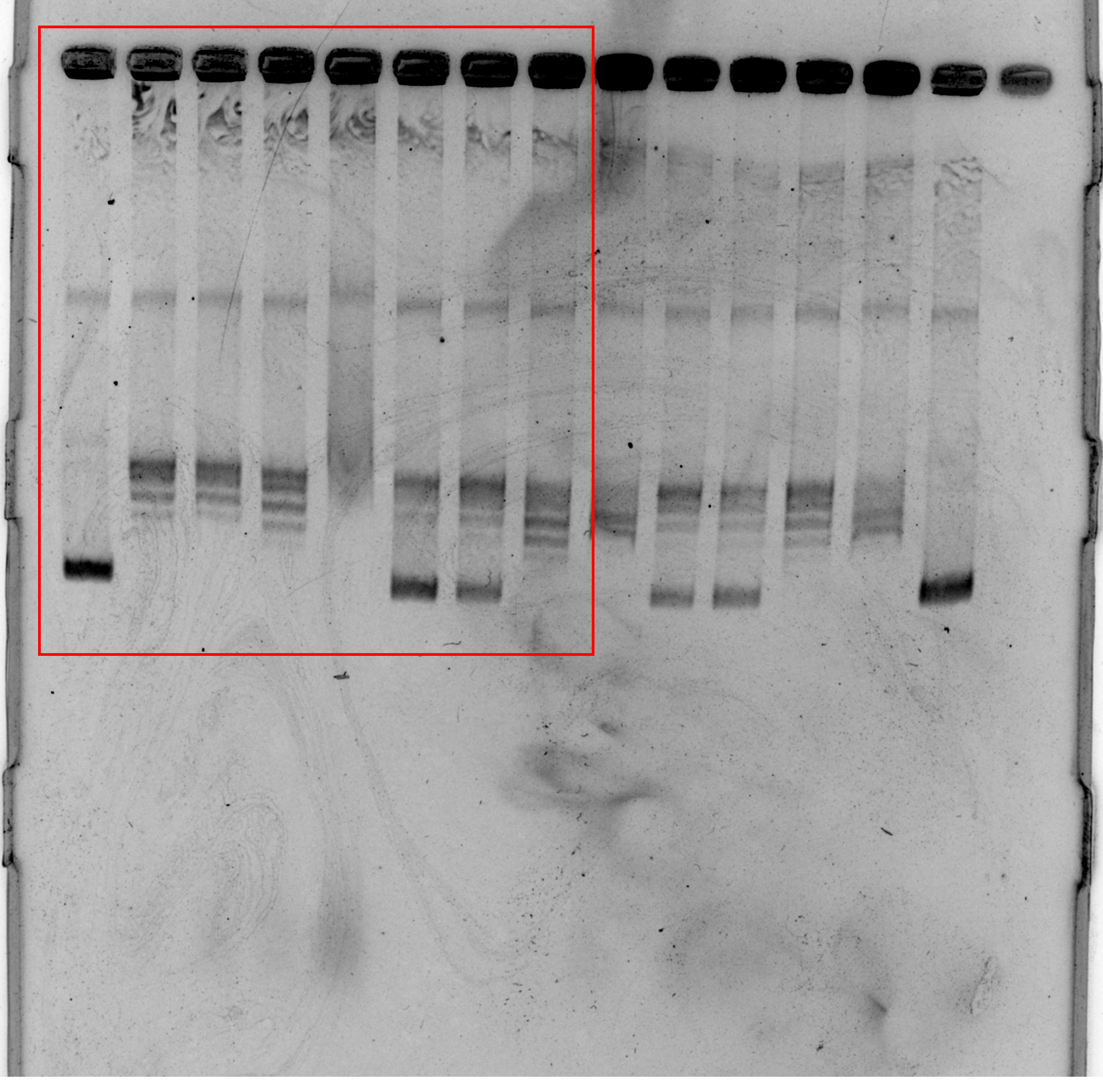

Supplement: Supplementary file 8 — Source data Fig. 6 [file 44318_2026_783_MOESM8_ESM.zip › Figure 6/Figure 6A/Plasmid Relaxation Assay_Nuclear Extracts.tif]

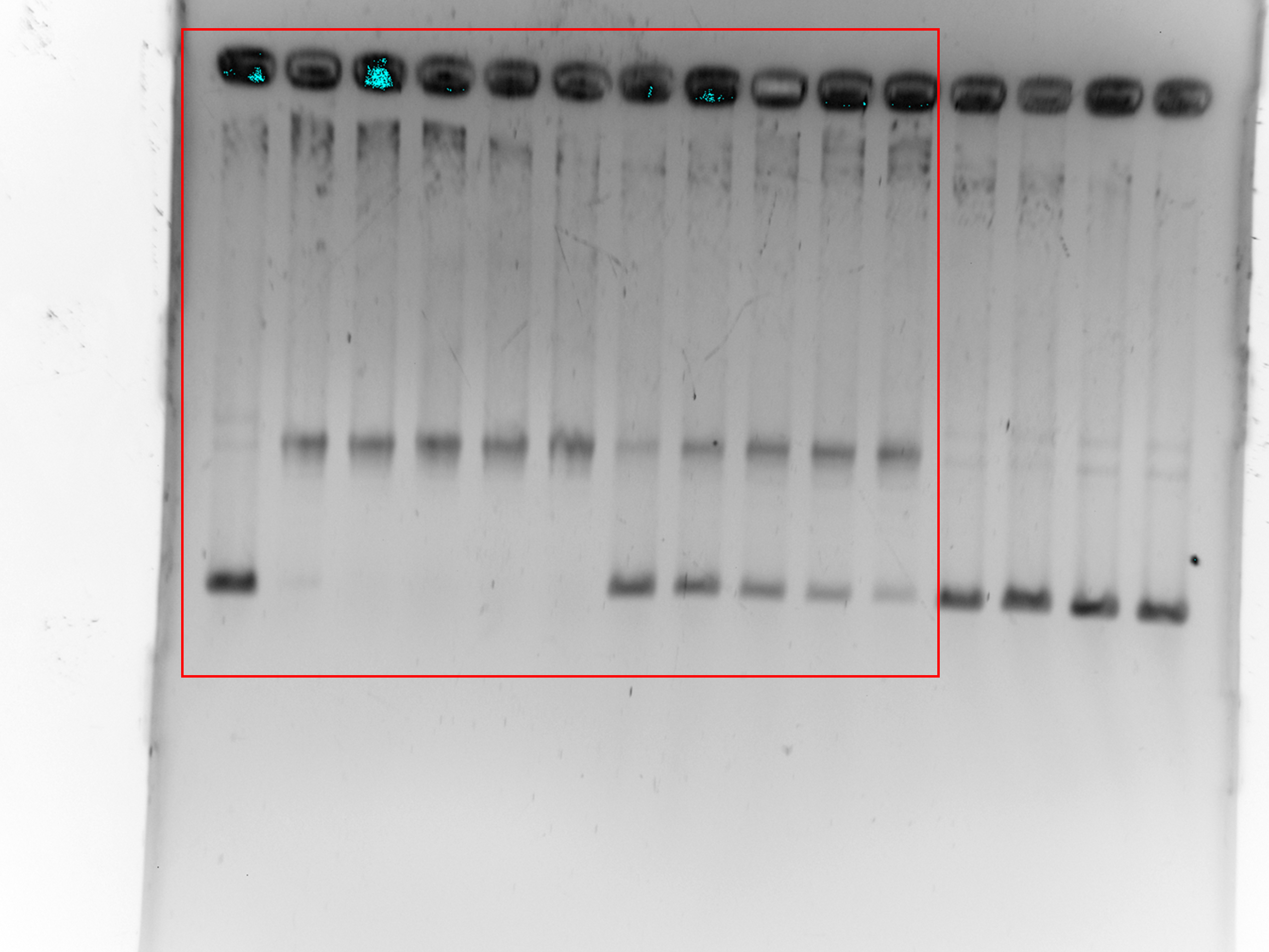

Supplement: Supplementary file 8 — Source data Fig. 6 [file 44318_2026_783_MOESM8_ESM.zip › Figure 6/Figure 6C/Plasmid Relaxation.tif]

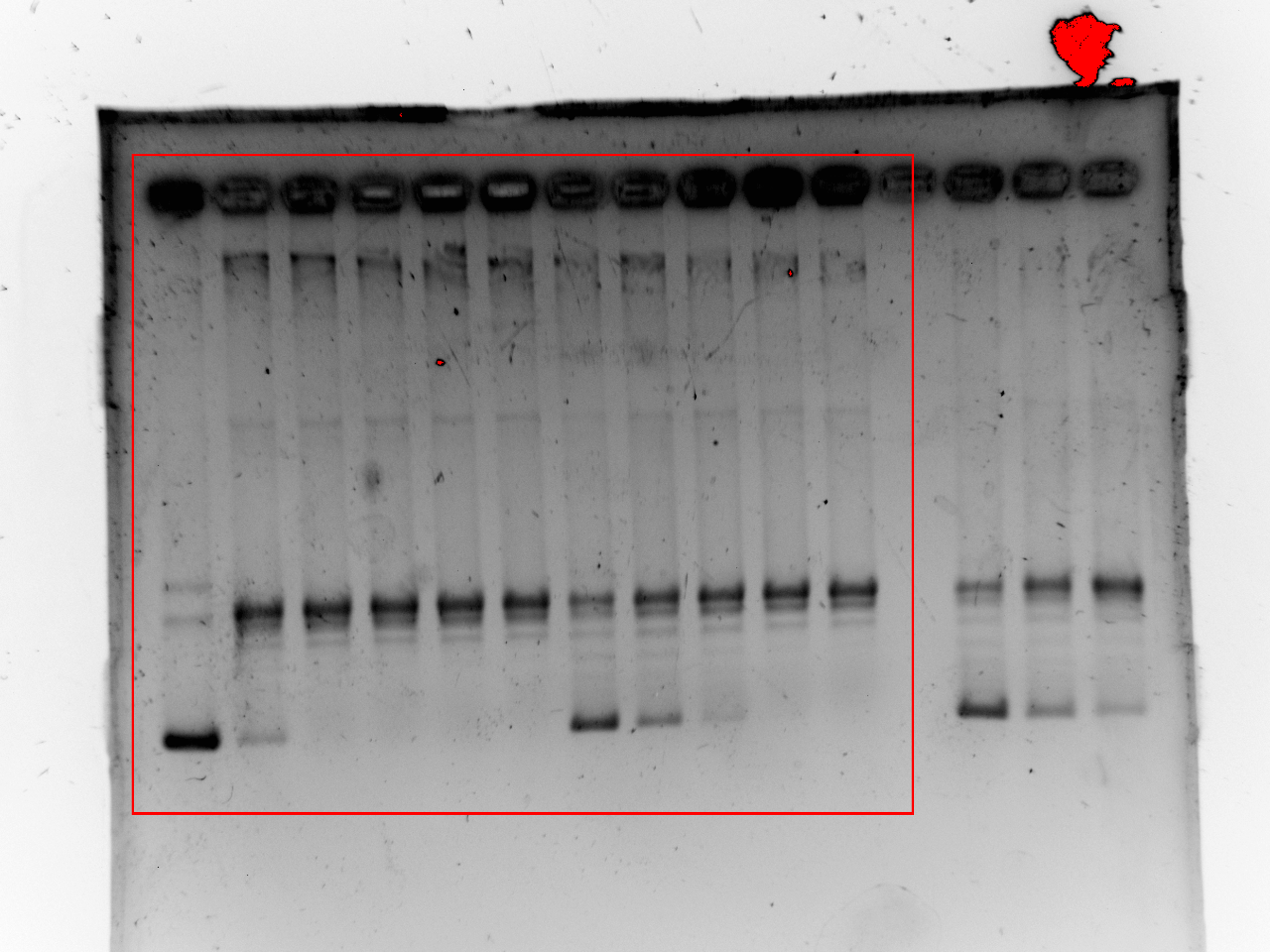

Supplement: Supplementary file 8 — Source data Fig. 6 [file 44318_2026_783_MOESM8_ESM.zip › Figure 6/Figure 6E/Plasmid Relaxation.tif]

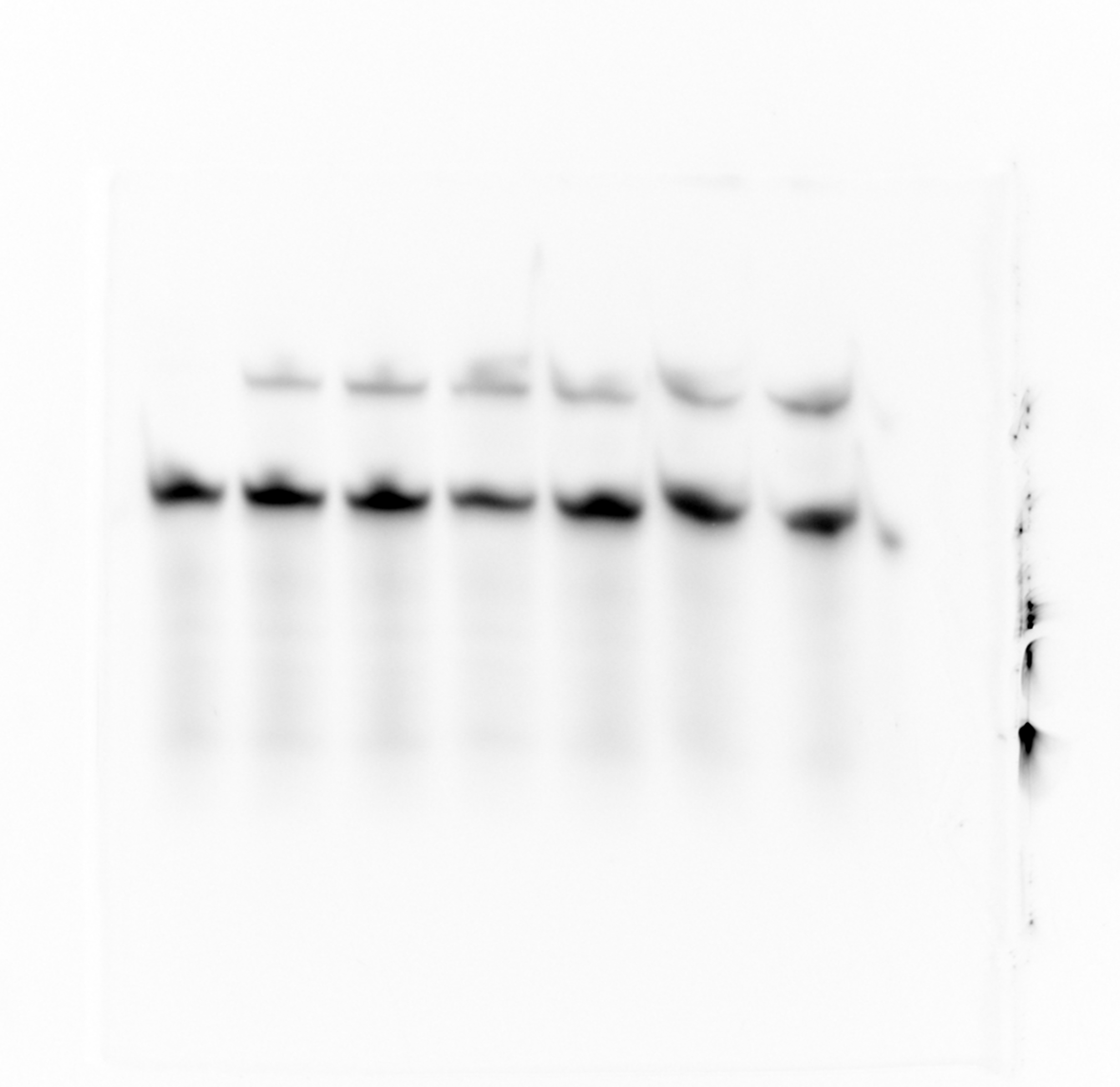

Supplement: Supplementary file 8 — Source data Fig. 6 [file 44318_2026_783_MOESM8_ESM.zip › Figure 6/Figure 6H/Cleavage Assay.tif]

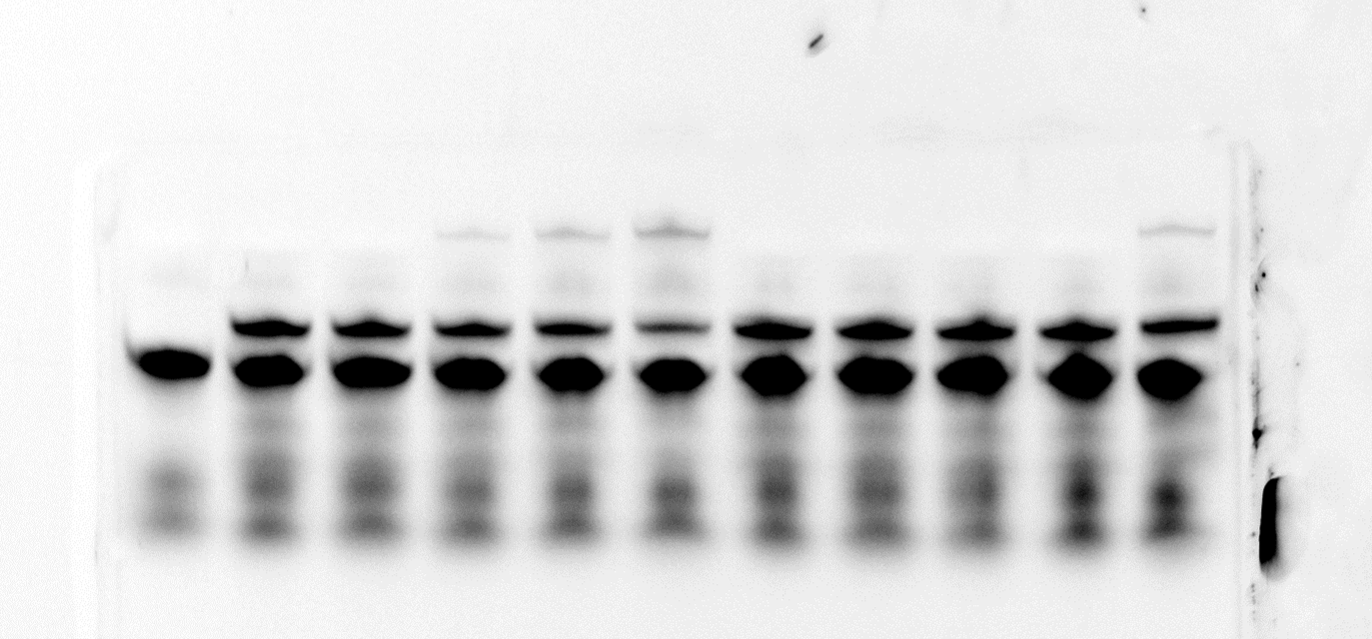

Supplement: Supplementary file 8 — Source data Fig. 6 [file 44318_2026_783_MOESM8_ESM.zip › Figure 6/Figure 6J/Religation Assay.tif]

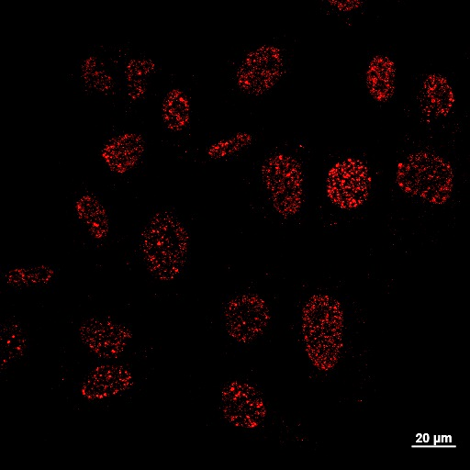

Supplement: Supplementary file 9 — Source data Fig. 7 [file 44318_2026_783_MOESM9_ESM.zip › Figure 7/Figure 7A/S320A_g2HAX.tif]

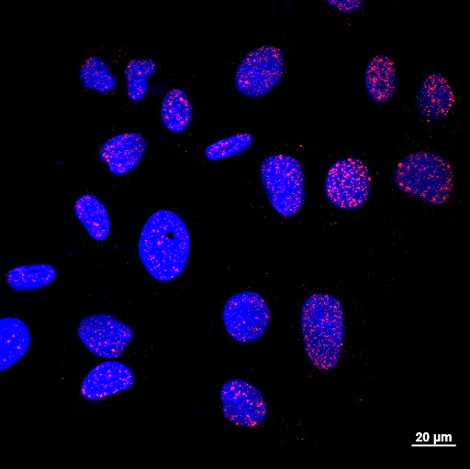

Supplement: Supplementary file 9 — Source data Fig. 7 [file 44318_2026_783_MOESM9_ESM.zip › Figure 7/Figure 7A/S320A_Merged.tif]

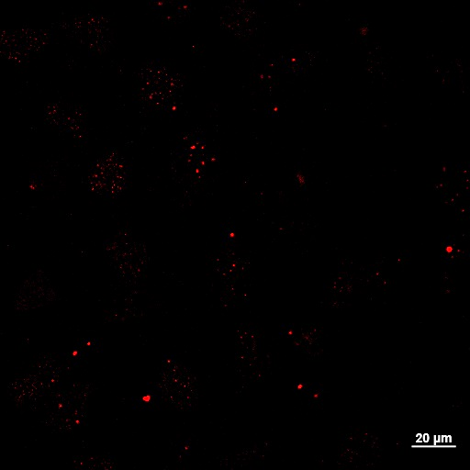

Supplement: Supplementary file 9 — Source data Fig. 7 [file 44318_2026_783_MOESM9_ESM.zip › Figure 7/Figure 7A/WT_gH2AX.tif]

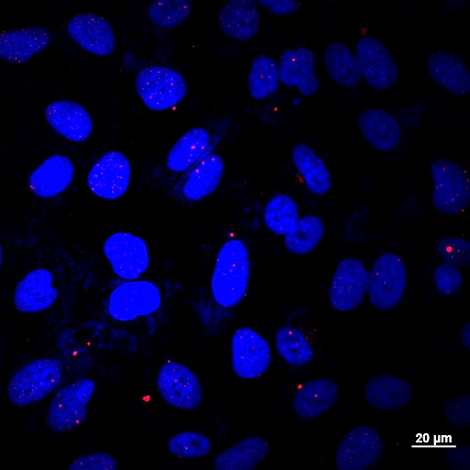

Supplement: Supplementary file 9 — Source data Fig. 7 [file 44318_2026_783_MOESM9_ESM.zip › Figure 7/Figure 7A/WT_Merged.tif]

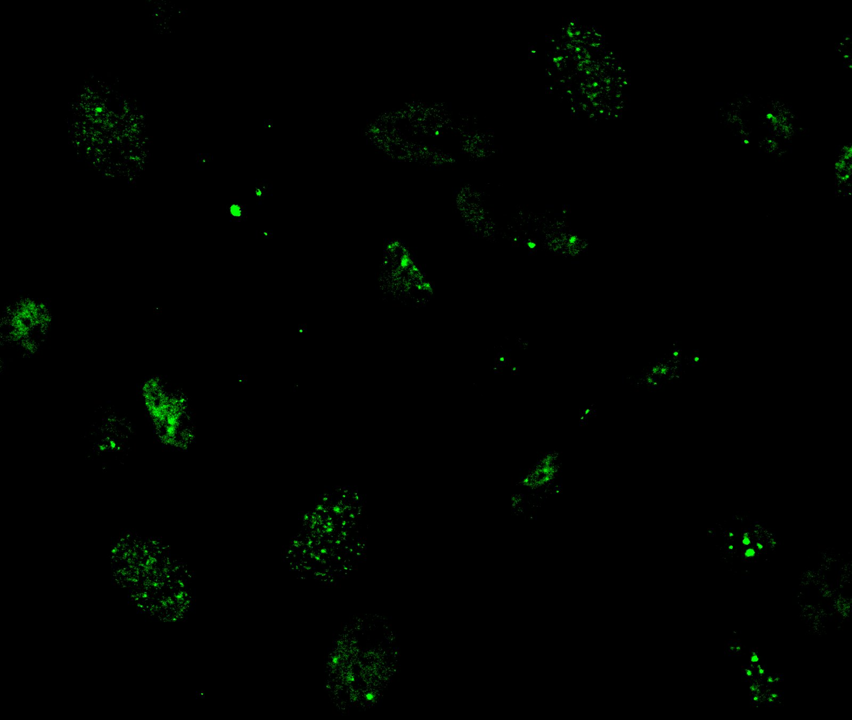

Supplement: Supplementary file 9 — Source data Fig. 7 [file 44318_2026_783_MOESM9_ESM.zip › Figure 7/Figure 7D/S320A_53BP1_EdU_53BP1.tif]

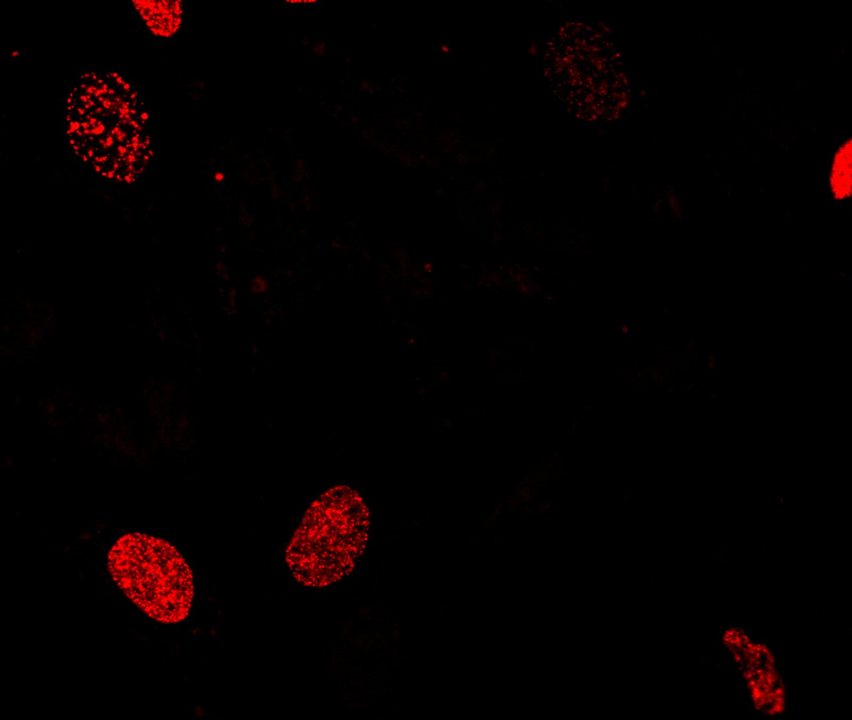

Supplement: Supplementary file 9 — Source data Fig. 7 [file 44318_2026_783_MOESM9_ESM.zip › Figure 7/Figure 7D/S320A_53BP1_EdU_EdU.tif]

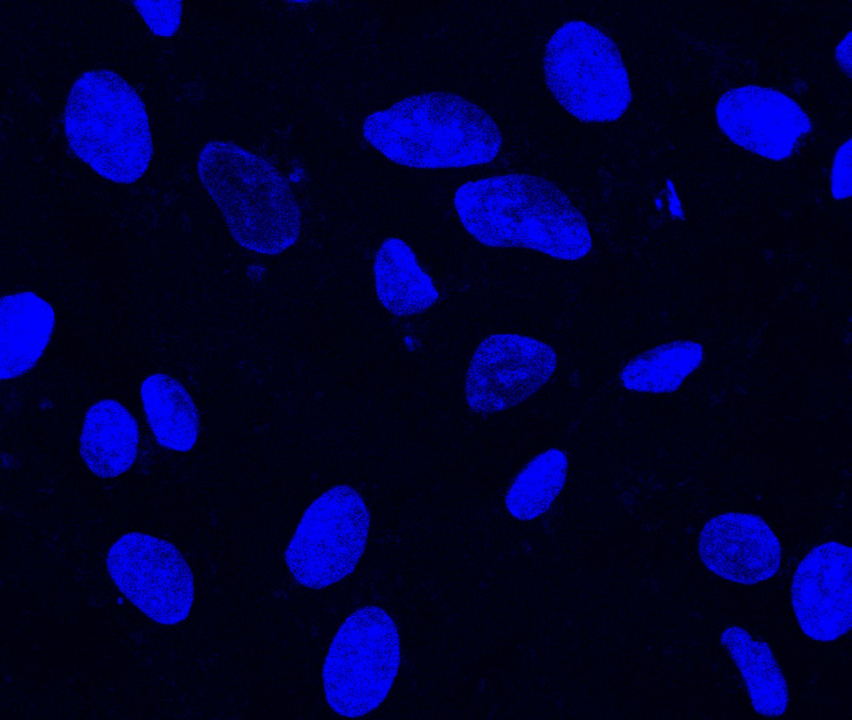

Supplement: Supplementary file 9 — Source data Fig. 7 [file 44318_2026_783_MOESM9_ESM.zip › Figure 7/Figure 7D/S320A_53BP1_EdU_Hoechst.tif]

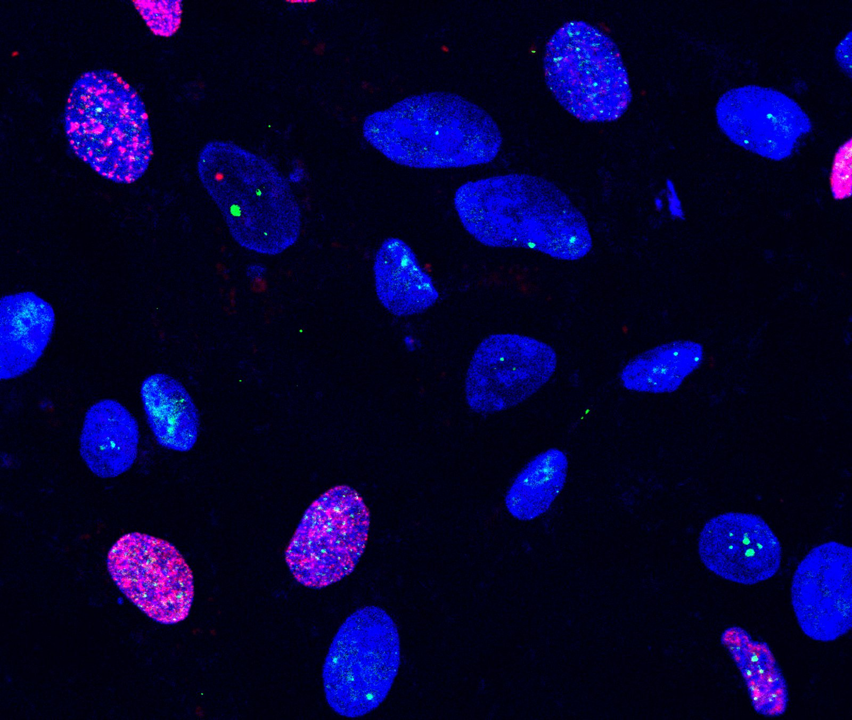

Supplement: Supplementary file 9 — Source data Fig. 7 [file 44318_2026_783_MOESM9_ESM.zip › Figure 7/Figure 7D/S320A_53BP1_EdU_Merged.tif]

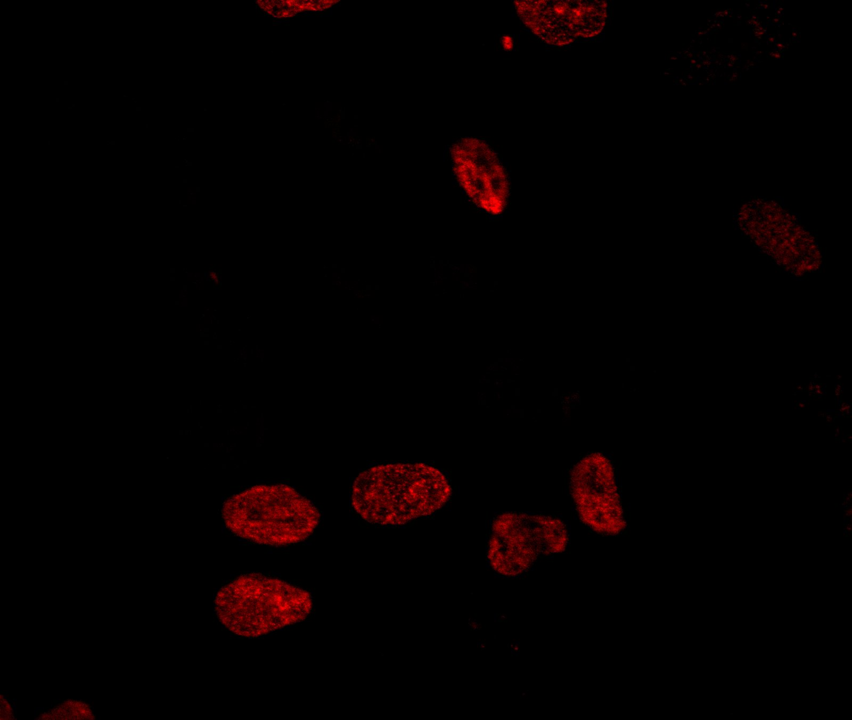

Supplement: Supplementary file 9 — Source data Fig. 7 [file 44318_2026_783_MOESM9_ESM.zip › Figure 7/Figure 7D/S320A_gH2AX_EdU_EdU.tif]

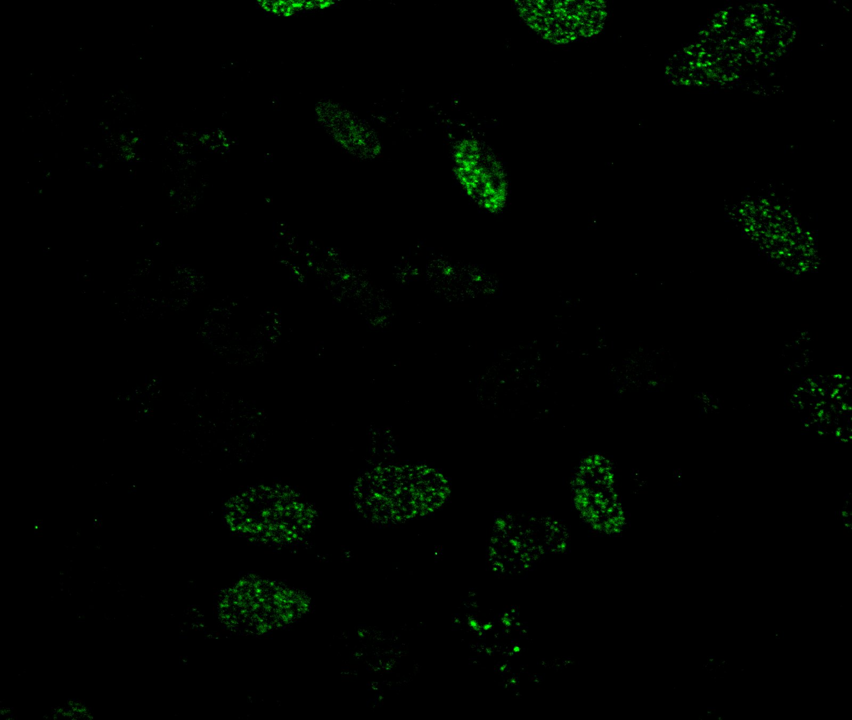

Supplement: Supplementary file 9 — Source data Fig. 7 [file 44318_2026_783_MOESM9_ESM.zip › Figure 7/Figure 7D/S320A_gH2AX_EdU_gH2AX.tif]

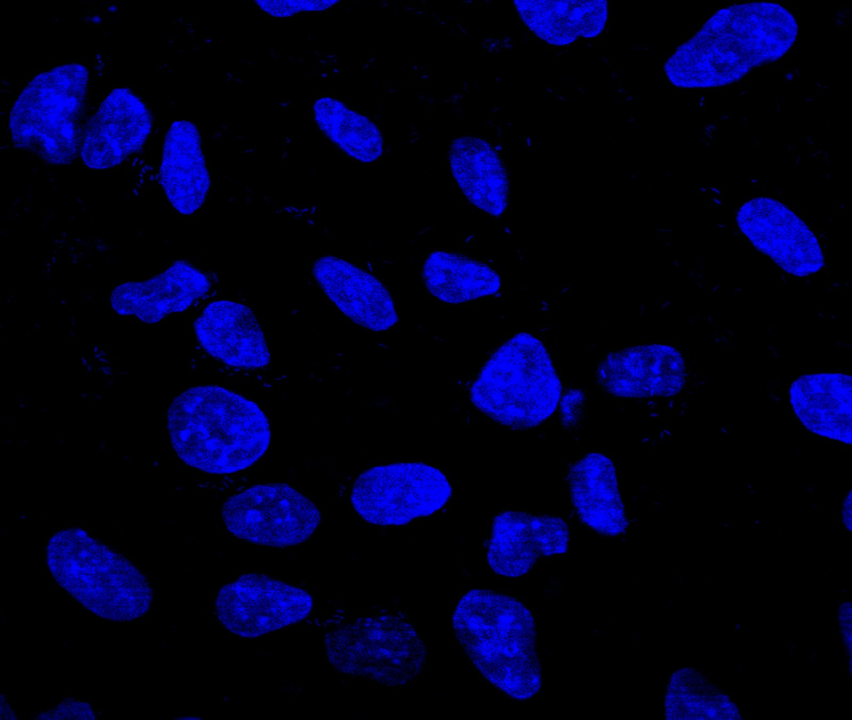

Supplement: Supplementary file 9 — Source data Fig. 7 [file 44318_2026_783_MOESM9_ESM.zip › Figure 7/Figure 7D/S320A_gH2AX_EdU_Hoechst.tif]

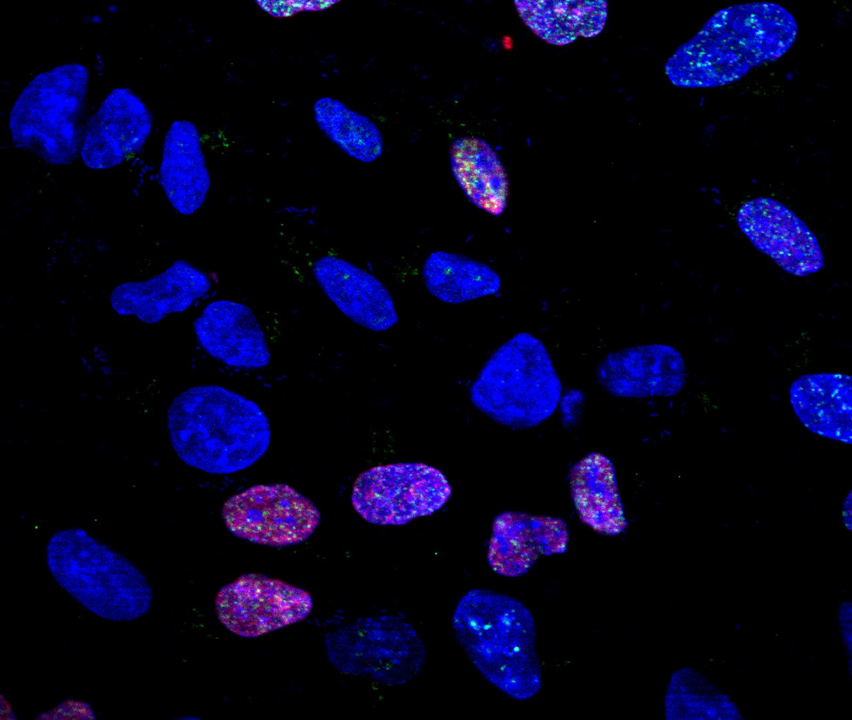

Supplement: Supplementary file 9 — Source data Fig. 7 [file 44318_2026_783_MOESM9_ESM.zip › Figure 7/Figure 7D/S320A_gH2AX_EdU_Merged.tif]

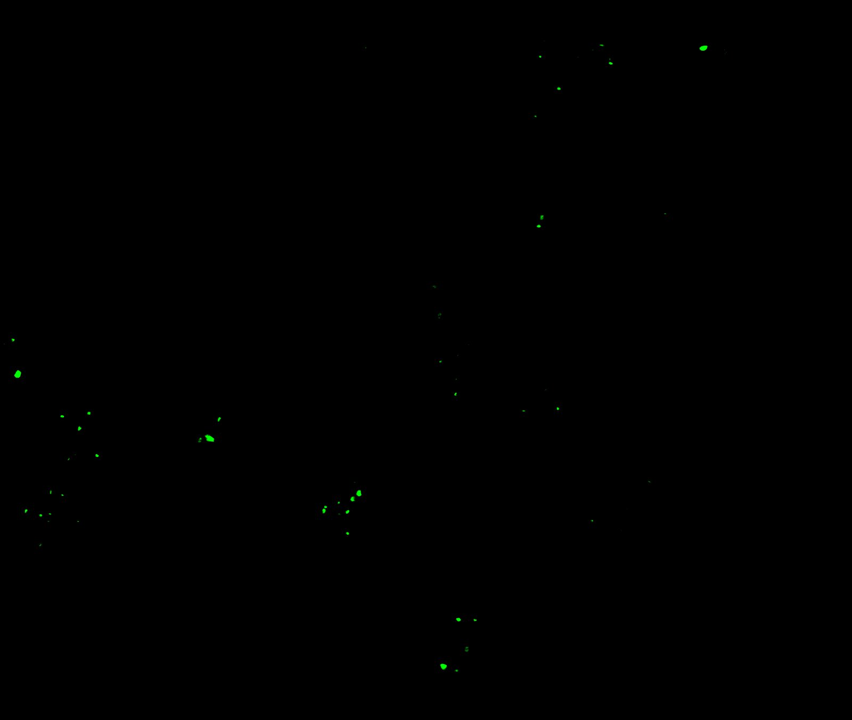

Supplement: Supplementary file 9 — Source data Fig. 7 [file 44318_2026_783_MOESM9_ESM.zip › Figure 7/Figure 7D/WT_53BP1_EdU_53BP1.tif]

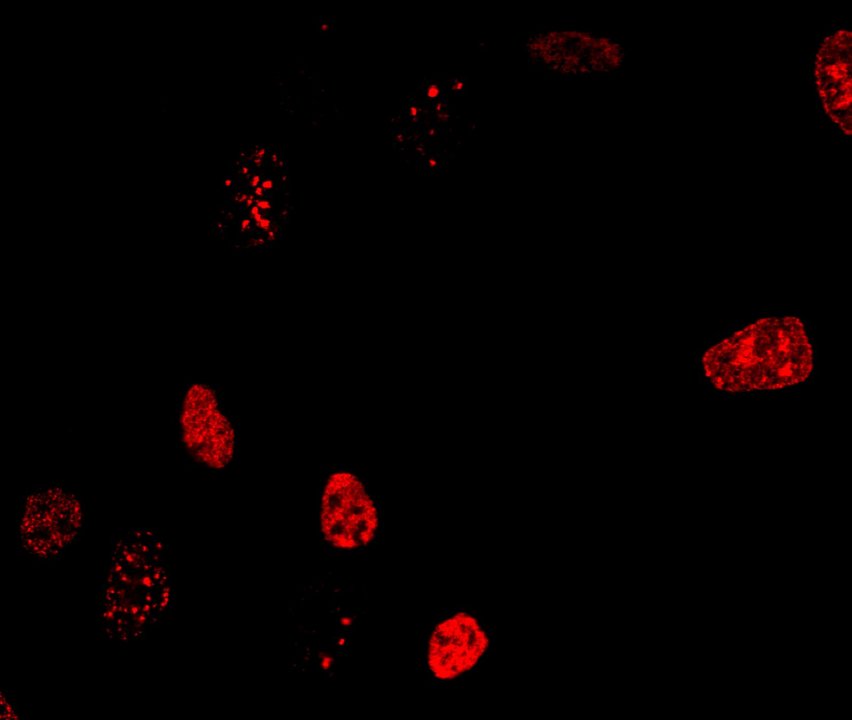

Supplement: Supplementary file 9 — Source data Fig. 7 [file 44318_2026_783_MOESM9_ESM.zip › Figure 7/Figure 7D/WT_53BP1_EdU_EdU.tif]

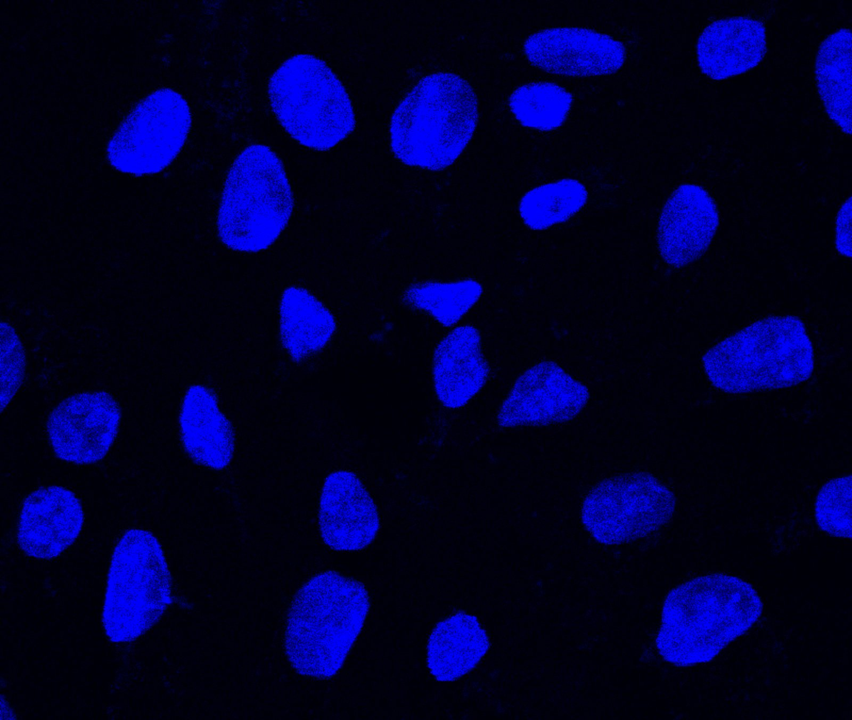

Supplement: Supplementary file 9 — Source data Fig. 7 [file 44318_2026_783_MOESM9_ESM.zip › Figure 7/Figure 7D/WT_53BP1_EdU_Hoechst.tif]

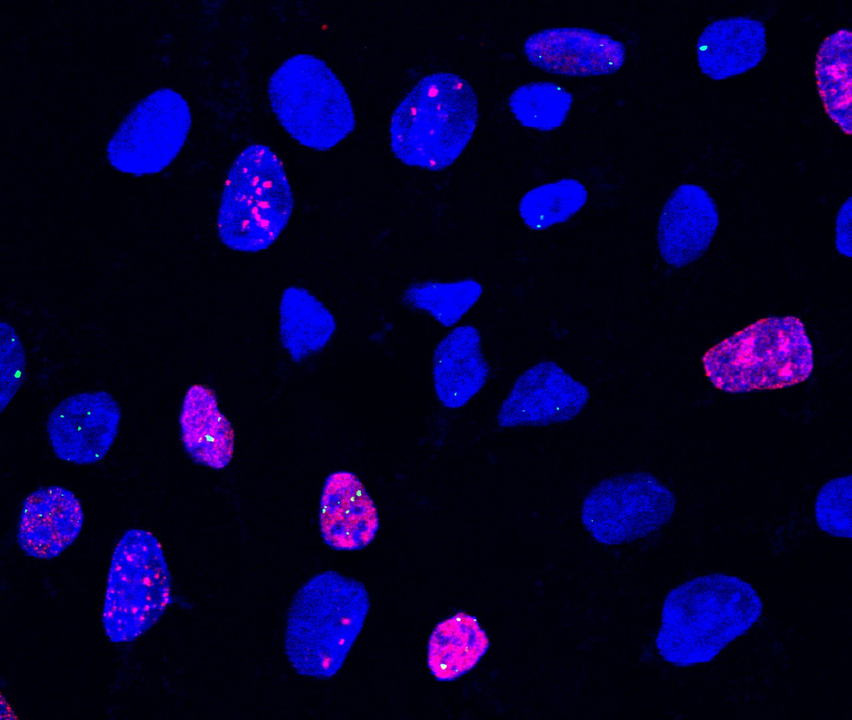

Supplement: Supplementary file 9 — Source data Fig. 7 [file 44318_2026_783_MOESM9_ESM.zip › Figure 7/Figure 7D/WT_53BP1_EdU_Merged.tif]

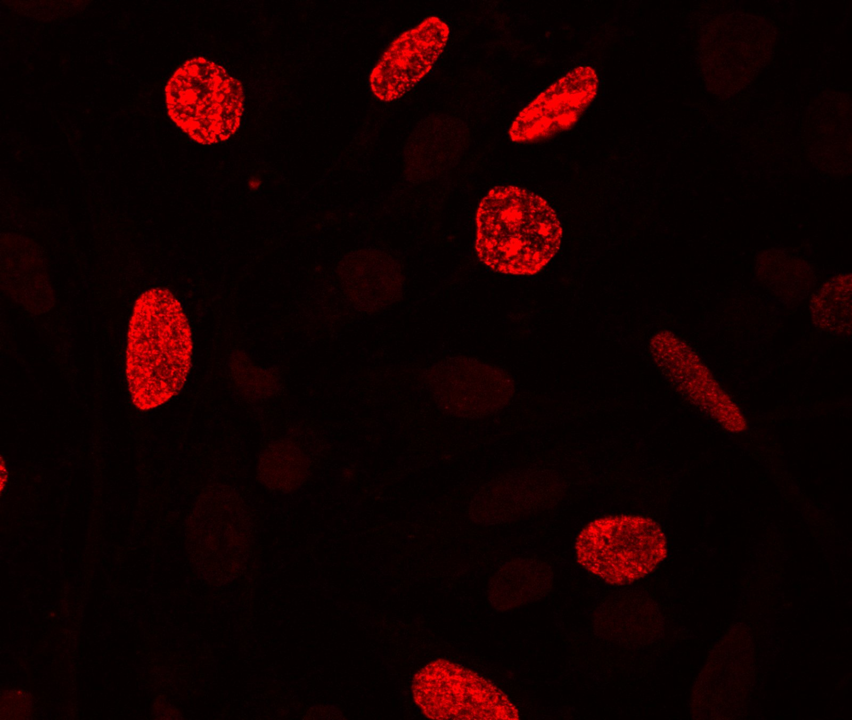

Supplement: Supplementary file 9 — Source data Fig. 7 [file 44318_2026_783_MOESM9_ESM.zip › Figure 7/Figure 7D/WT_gH2AX_EdU_EdU.tif]

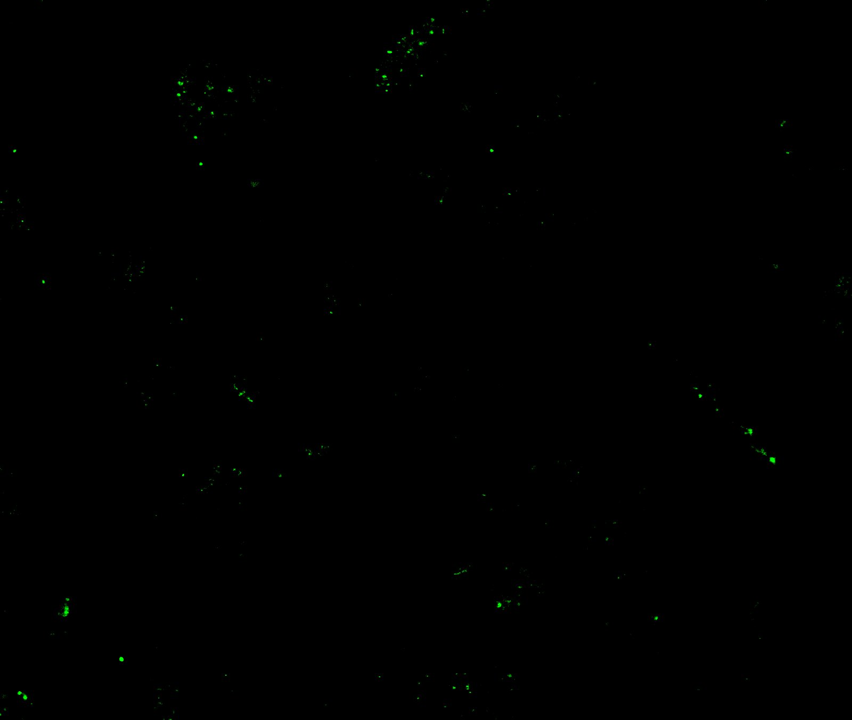

Supplement: Supplementary file 9 — Source data Fig. 7 [file 44318_2026_783_MOESM9_ESM.zip › Figure 7/Figure 7D/WT_gH2AX_EdU_gH2AX.tif]

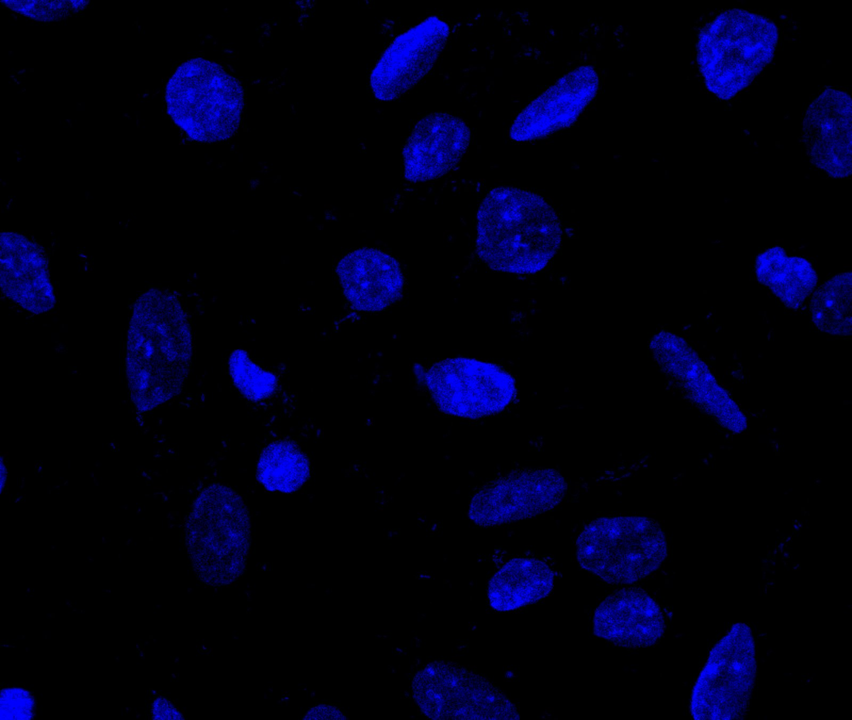

Supplement: Supplementary file 9 — Source data Fig. 7 [file 44318_2026_783_MOESM9_ESM.zip › Figure 7/Figure 7D/WT_gH2AX_EdU_Hoechst.tif]

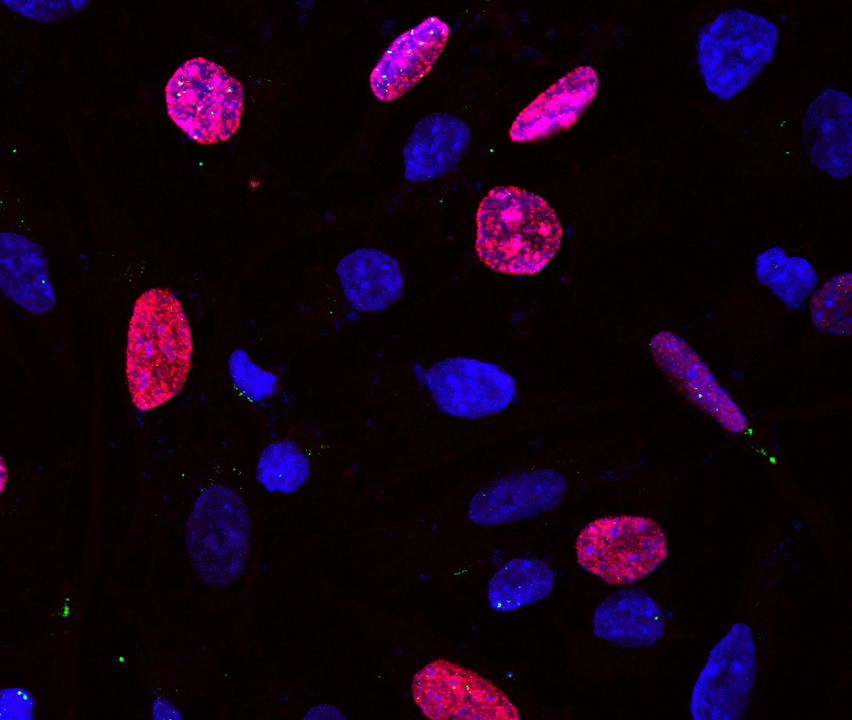

Supplement: Supplementary file 9 — Source data Fig. 7 [file 44318_2026_783_MOESM9_ESM.zip › Figure 7/Figure 7D/WT_gH2AX_EdU_Merged.tif]

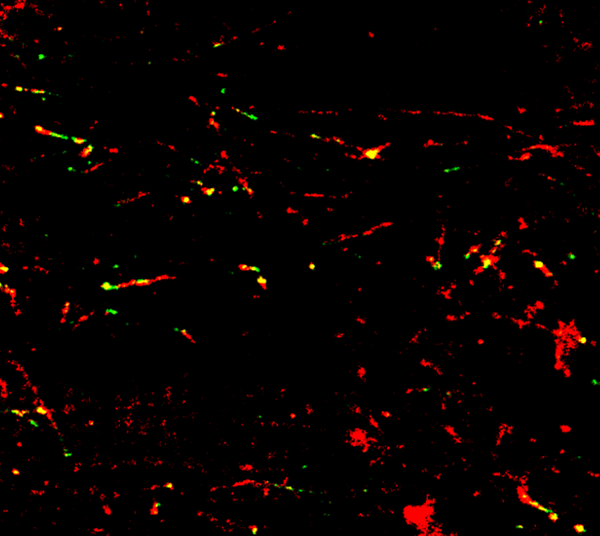

Supplement: Supplementary file 9 — Source data Fig. 7 [file 44318_2026_783_MOESM9_ESM.zip › Figure 7/Figure 7G/S320A_Fiber_1.tif]

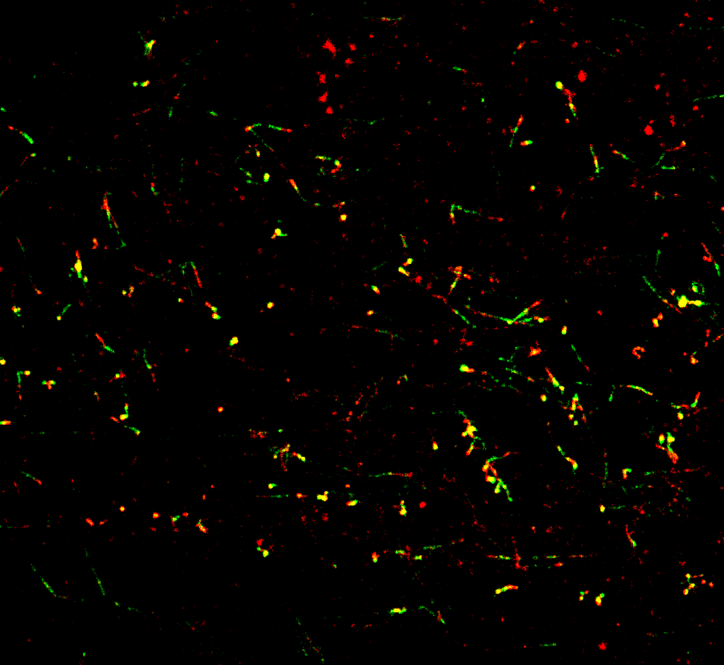

Supplement: Supplementary file 9 — Source data Fig. 7 [file 44318_2026_783_MOESM9_ESM.zip › Figure 7/Figure 7G/S320A_Fiber_2.tif]

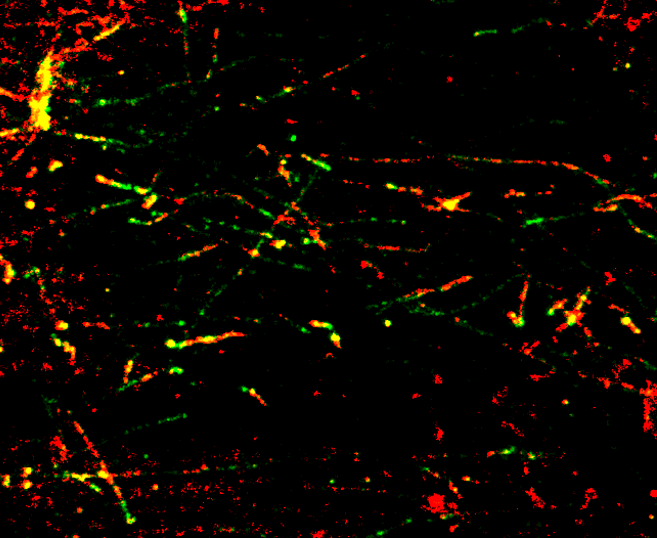

Supplement: Supplementary file 9 — Source data Fig. 7 [file 44318_2026_783_MOESM9_ESM.zip › Figure 7/Figure 7G/S320A_Fiber_3.tif]

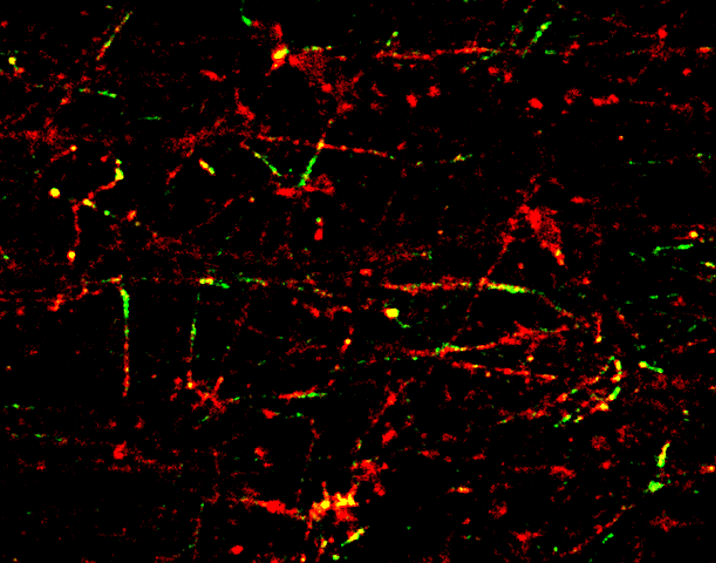

Supplement: Supplementary file 9 — Source data Fig. 7 [file 44318_2026_783_MOESM9_ESM.zip › Figure 7/Figure 7G/S320A_Fiber_4.tif]

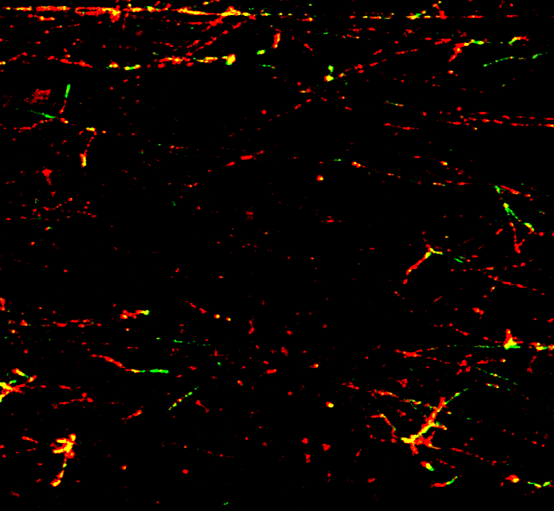

Supplement: Supplementary file 9 — Source data Fig. 7 [file 44318_2026_783_MOESM9_ESM.zip › Figure 7/Figure 7G/S320A_Fiber_5.tif]

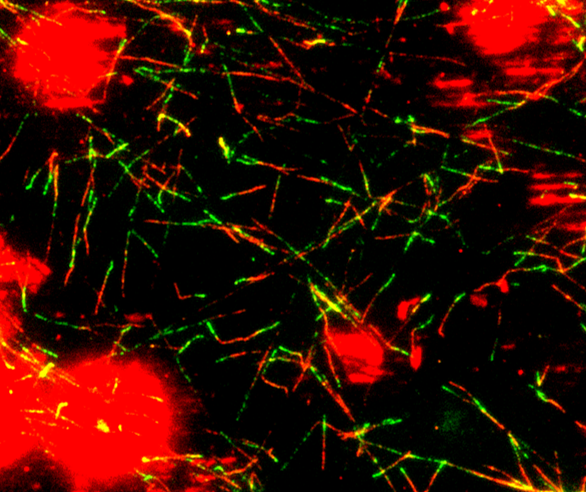

Supplement: Supplementary file 9 — Source data Fig. 7 [file 44318_2026_783_MOESM9_ESM.zip › Figure 7/Figure 7G/WT_Fiber_1.tif]

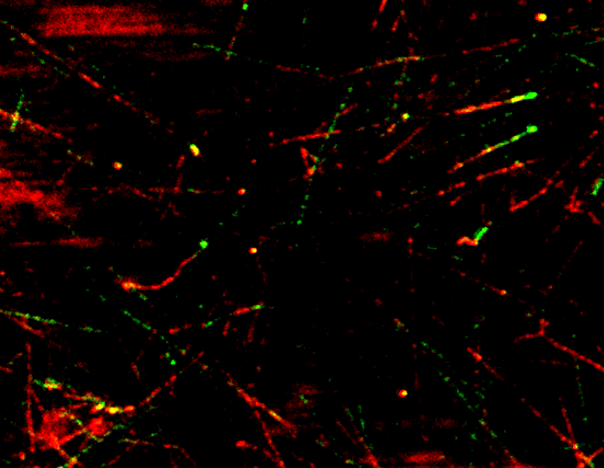

Supplement: Supplementary file 9 — Source data Fig. 7 [file 44318_2026_783_MOESM9_ESM.zip › Figure 7/Figure 7G/WT_Fiber_2.tif]

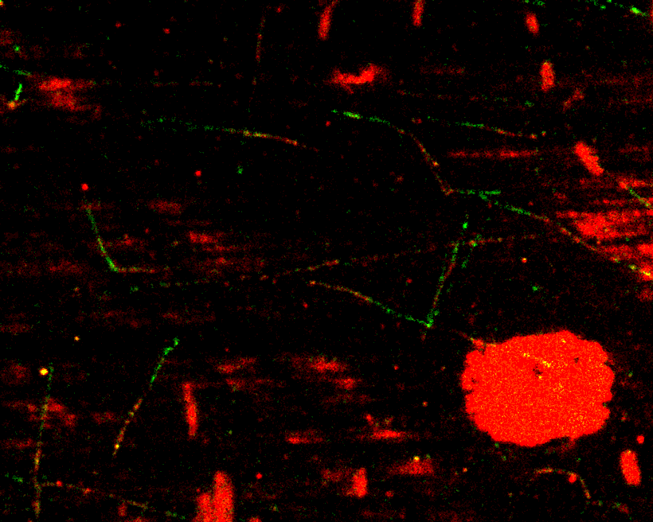

Supplement: Supplementary file 9 — Source data Fig. 7 [file 44318_2026_783_MOESM9_ESM.zip › Figure 7/Figure 7G/WT_Fiber_3.tif]

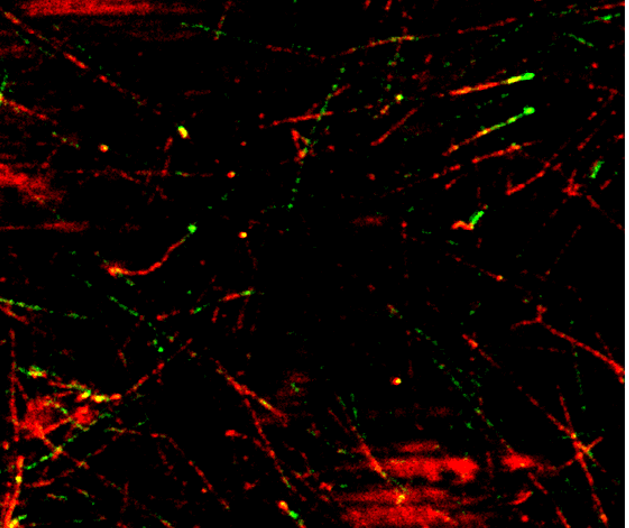

Supplement: Supplementary file 9 — Source data Fig. 7 [file 44318_2026_783_MOESM9_ESM.zip › Figure 7/Figure 7G/WT_Fiber_4.tif]

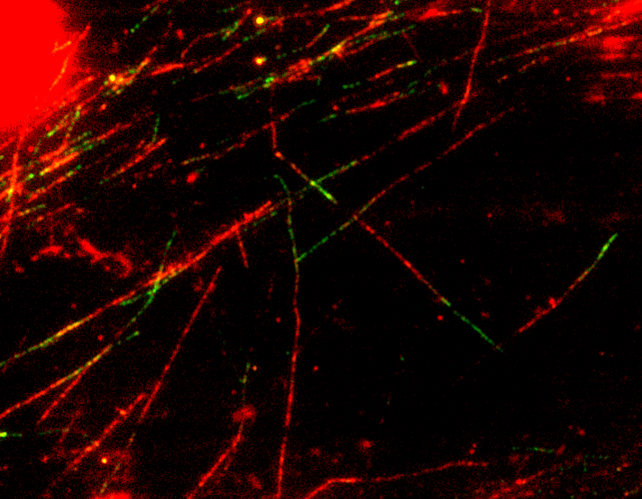

Supplement: Supplementary file 9 — Source data Fig. 7 [file 44318_2026_783_MOESM9_ESM.zip › Figure 7/Figure 7G/WT_Fiber_5.tif]

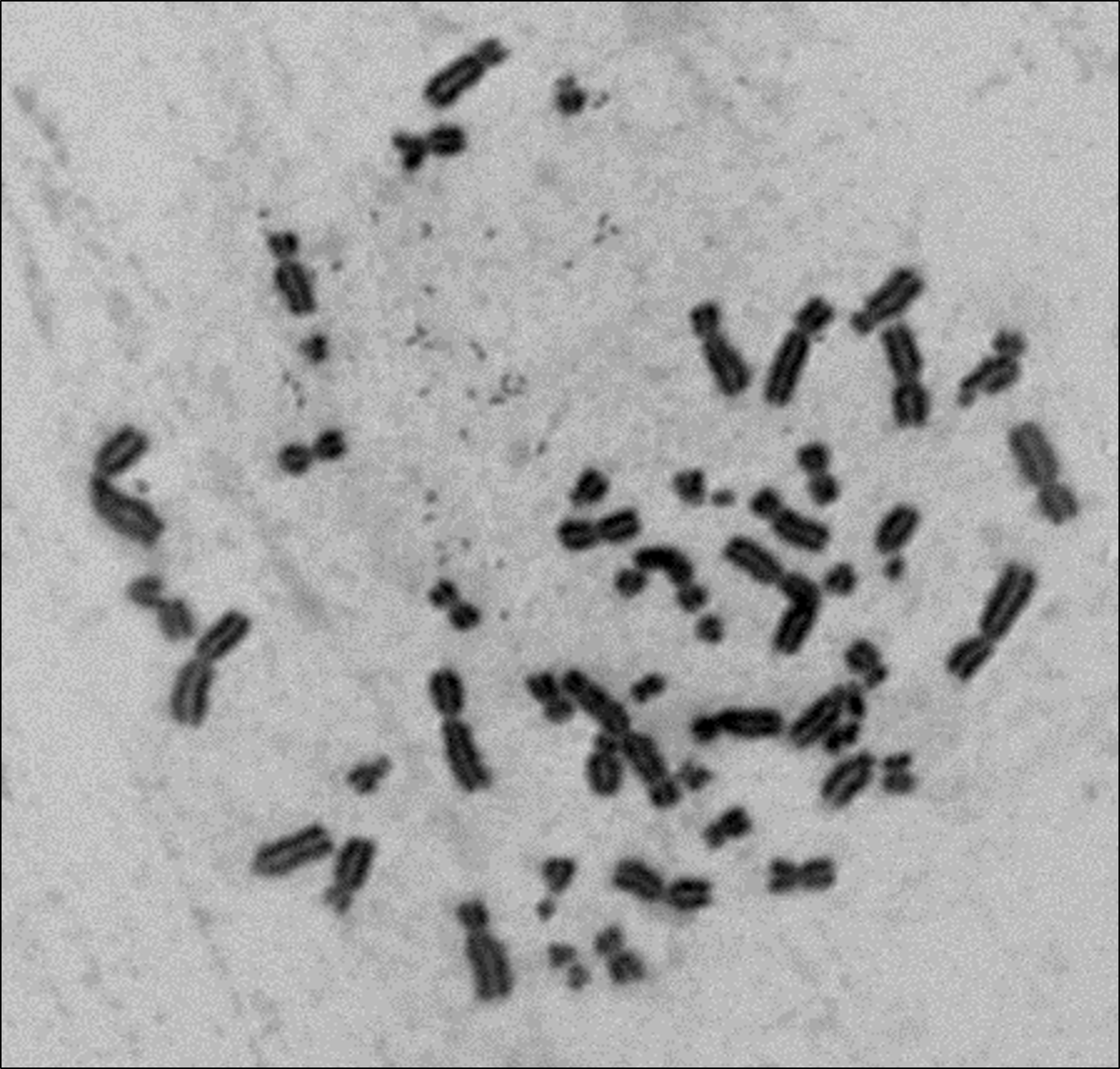

Supplement: Supplementary file 9 — Source data Fig. 7 [file 44318_2026_783_MOESM9_ESM.zip › Figure 7/Figure 7J/Metaphase_S320A.tif]

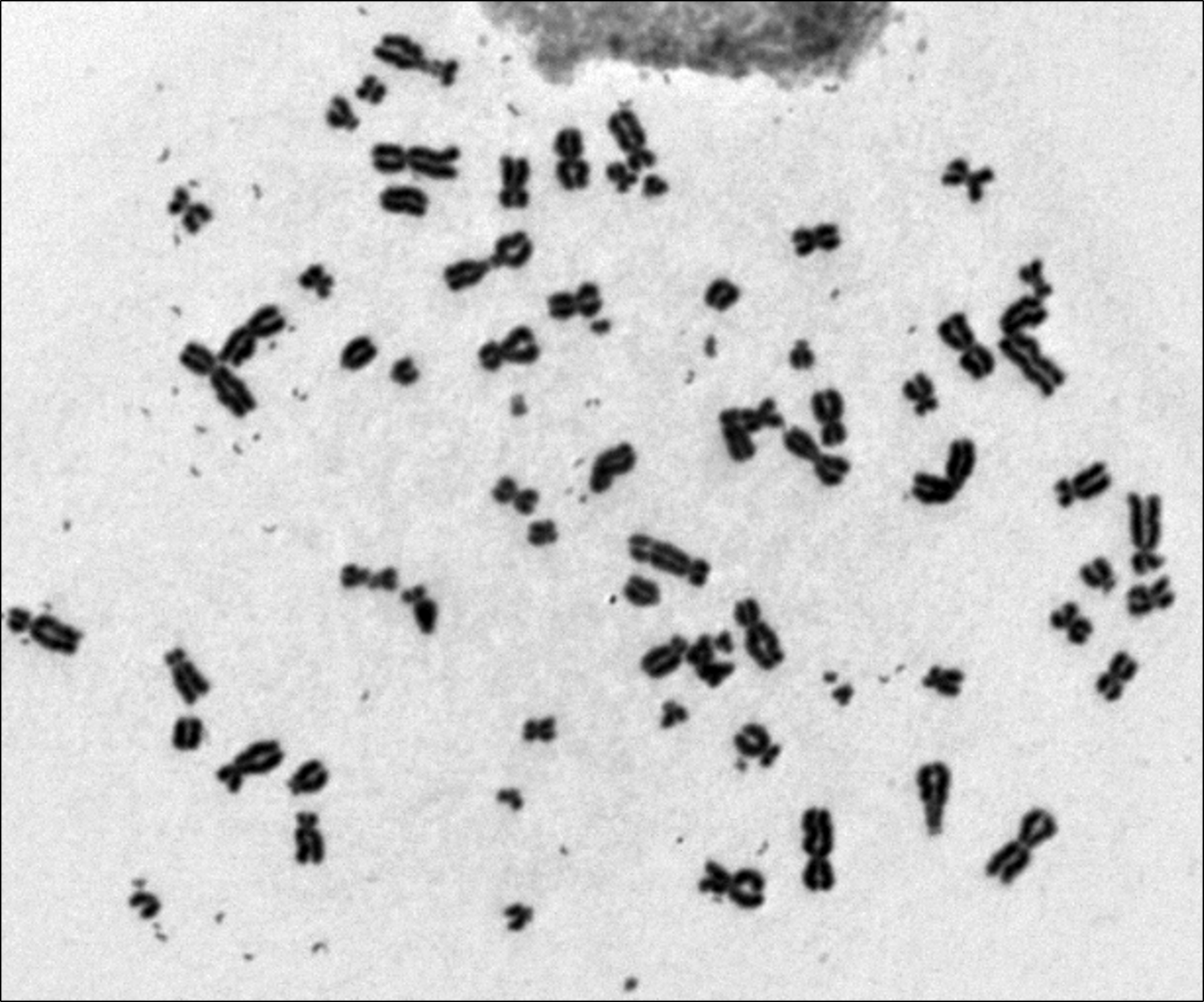

Supplement: Supplementary file 9 — Source data Fig. 7 [file 44318_2026_783_MOESM9_ESM.zip › Figure 7/Figure 7J/Metaphase_WT.tif]

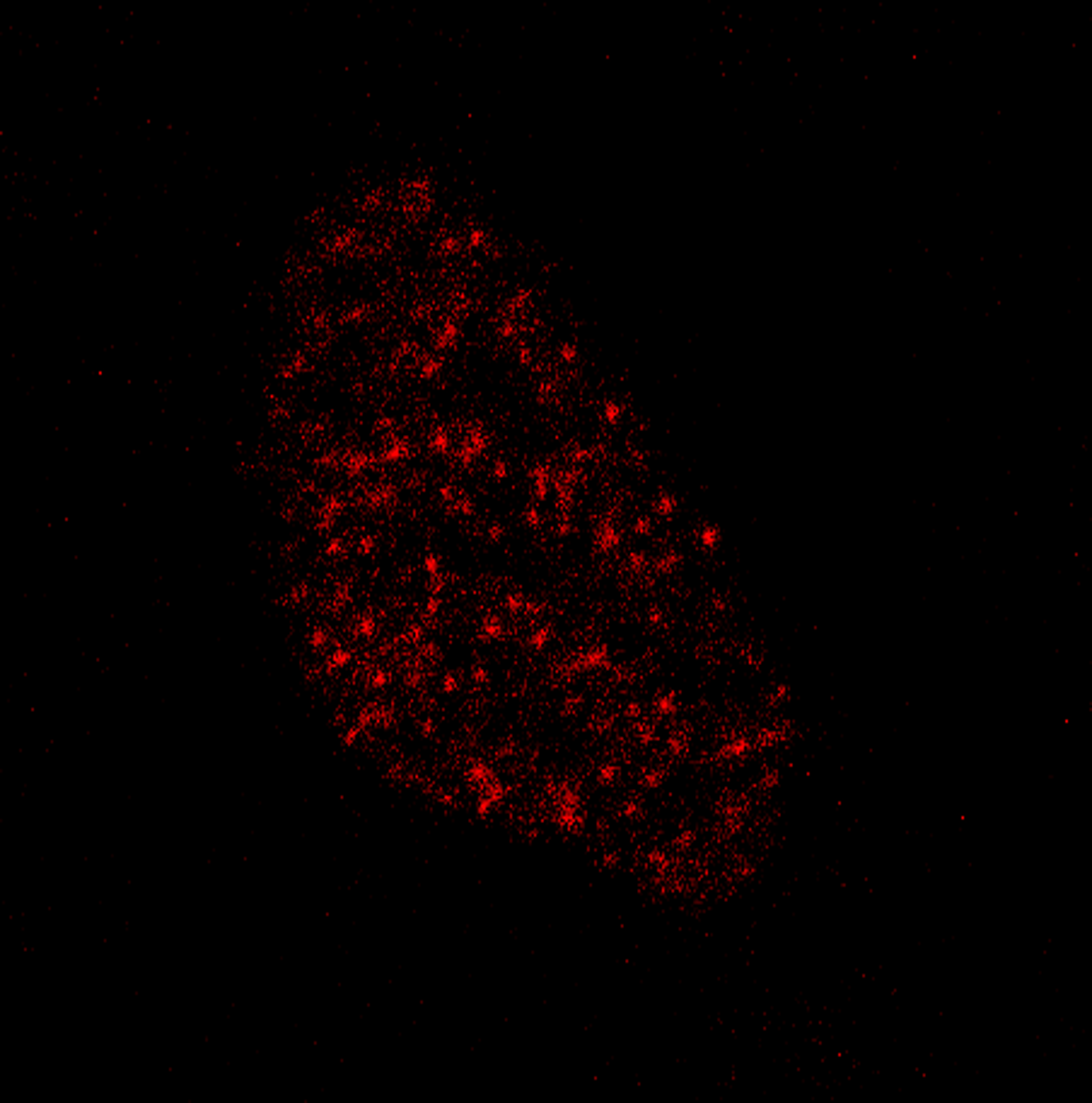

Supplement: Supplementary file 10 — Source data Fig. 8 [file 44318_2026_783_MOESM10_ESM.zip › Figure 8/Figure 8A/DRB_4h_gH2AX.tif]

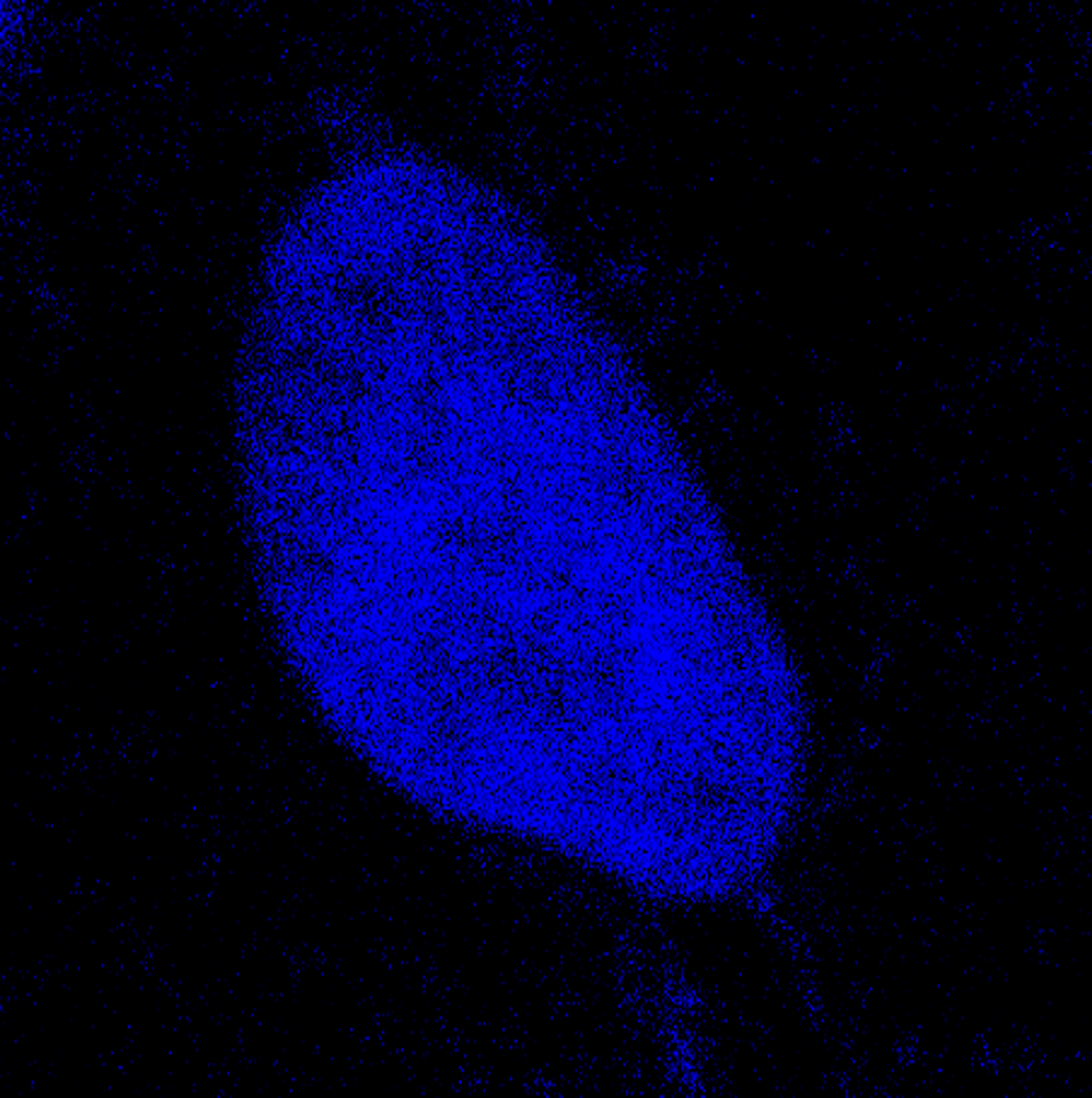

Supplement: Supplementary file 10 — Source data Fig. 8 [file 44318_2026_783_MOESM10_ESM.zip › Figure 8/Figure 8A/DRB_4h_Hoechst.tif]

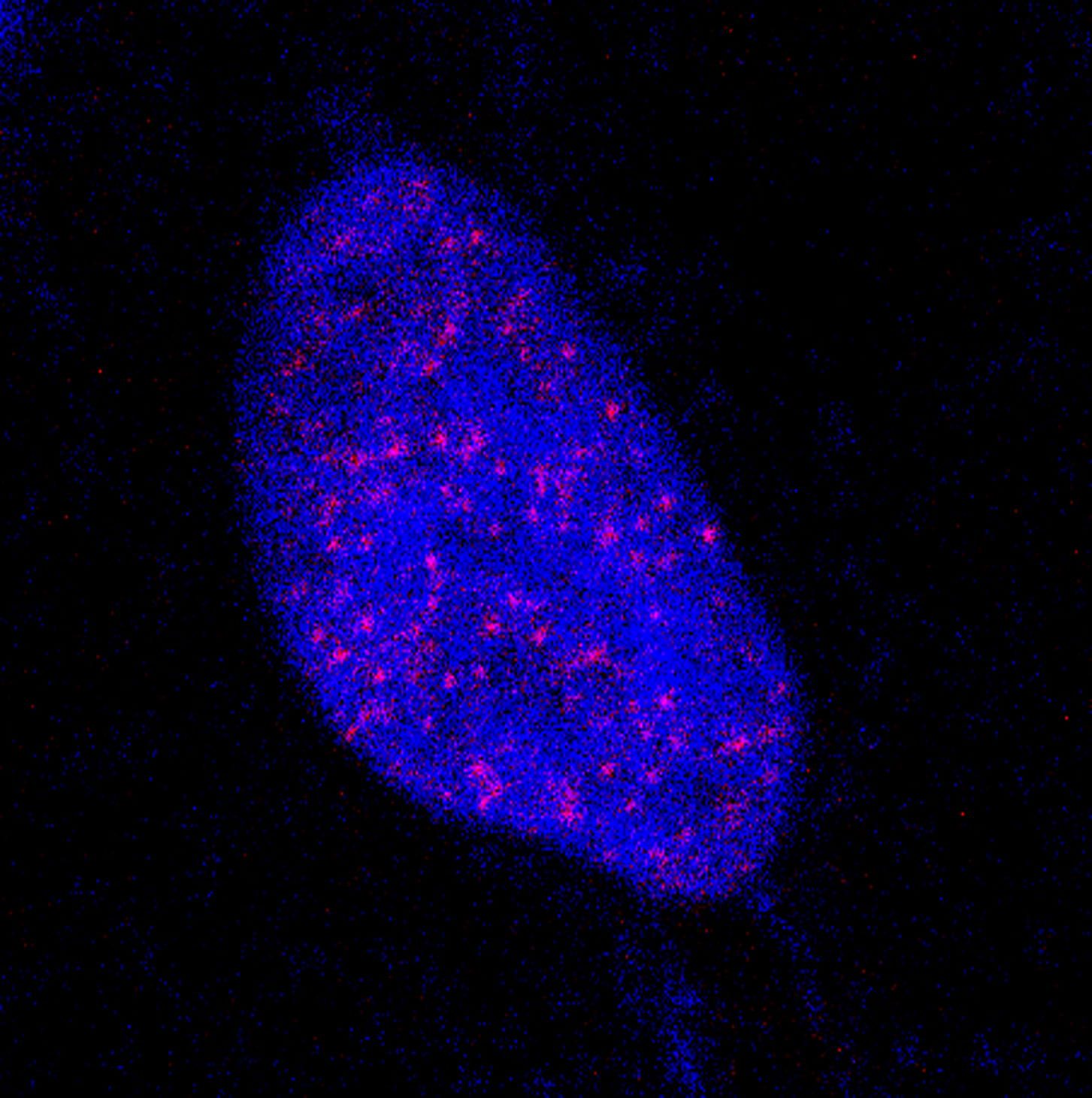

Supplement: Supplementary file 10 — Source data Fig. 8 [file 44318_2026_783_MOESM10_ESM.zip › Figure 8/Figure 8A/DRB_4h_Merged.tif]

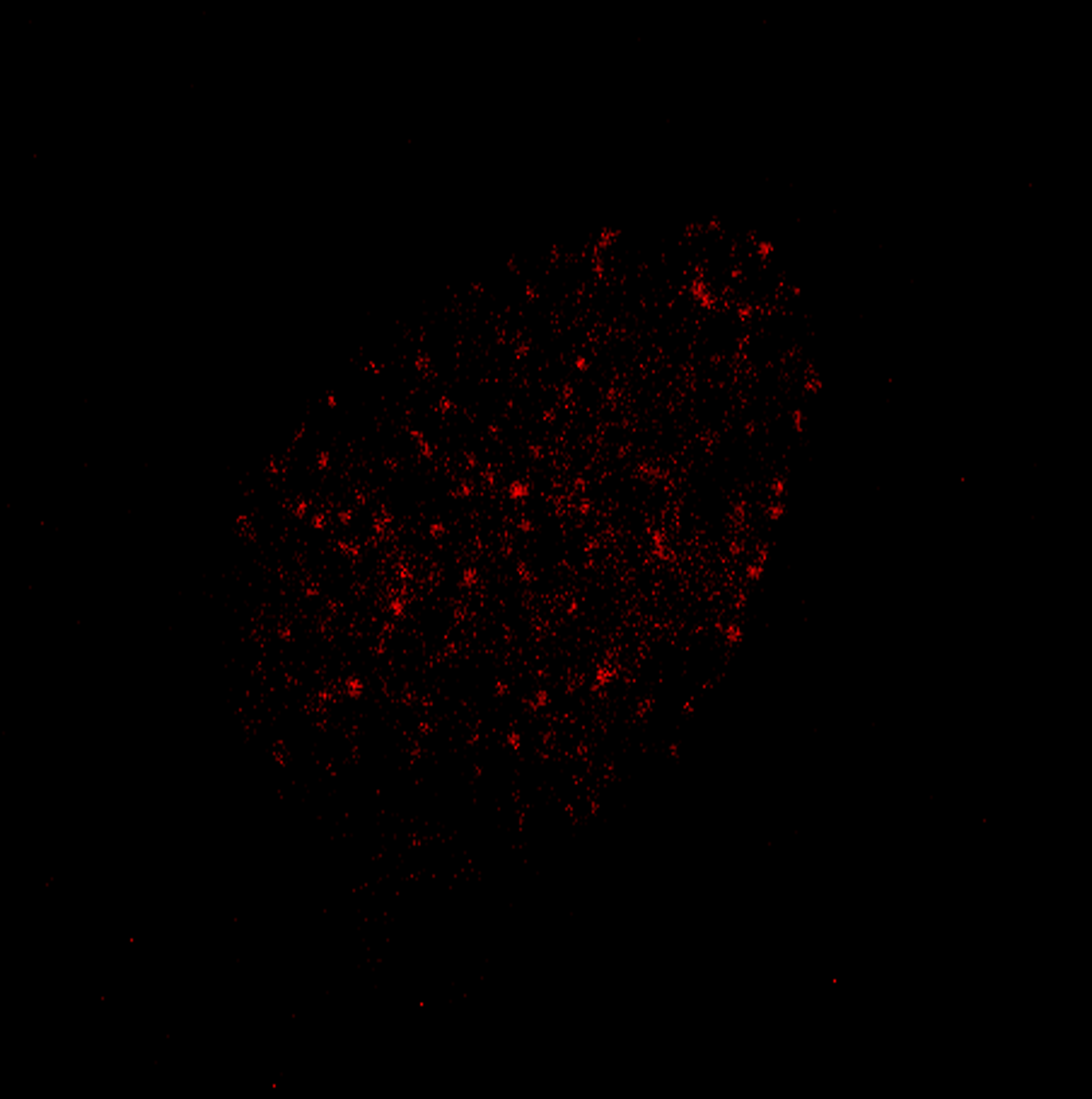

Supplement: Supplementary file 10 — Source data Fig. 8 [file 44318_2026_783_MOESM10_ESM.zip › Figure 8/Figure 8A/DRB_6h_gH2AX.tif]

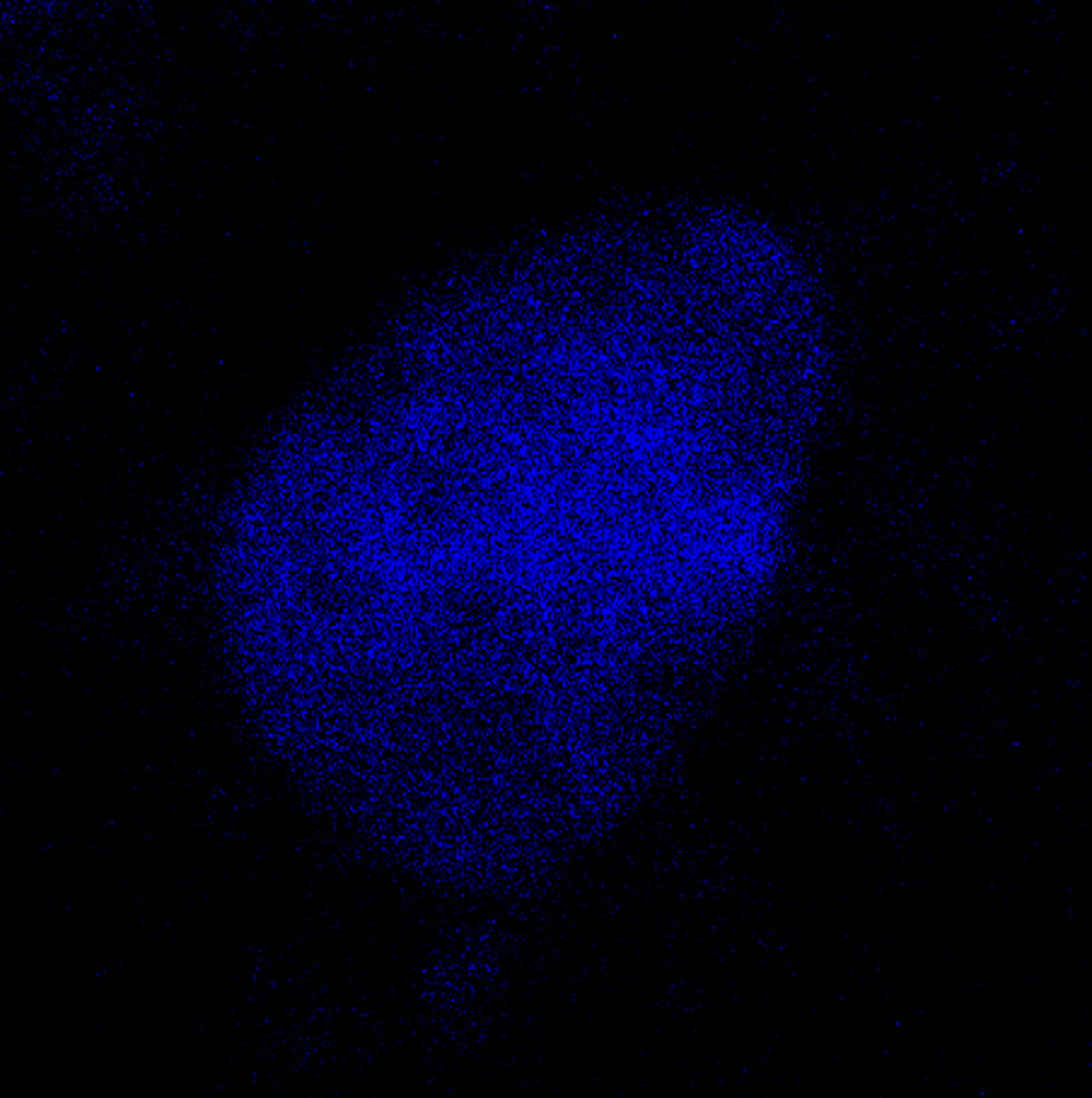

Supplement: Supplementary file 10 — Source data Fig. 8 [file 44318_2026_783_MOESM10_ESM.zip › Figure 8/Figure 8A/DRB_6h_Hoechst.tif]

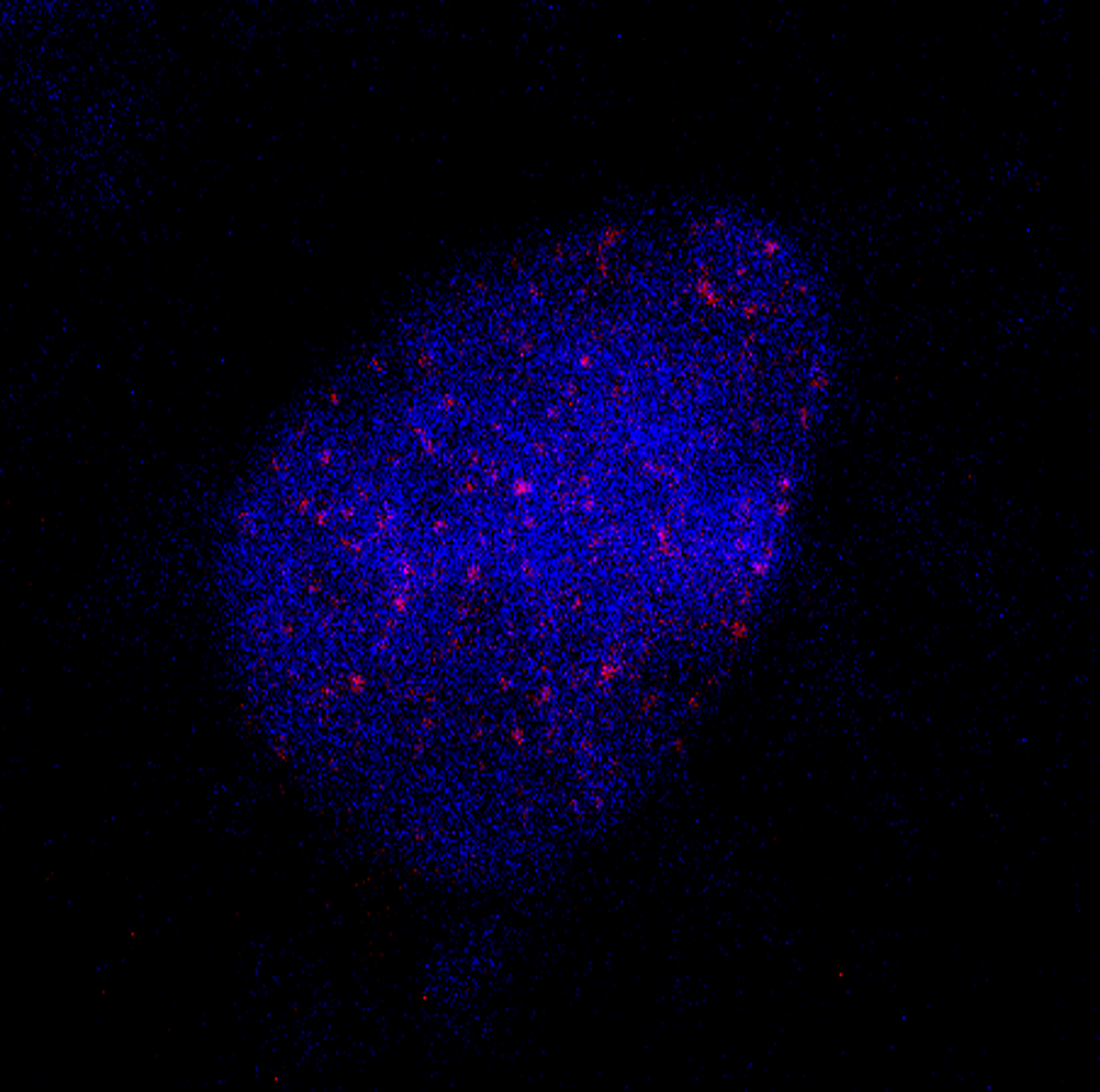

Supplement: Supplementary file 10 — Source data Fig. 8 [file 44318_2026_783_MOESM10_ESM.zip › Figure 8/Figure 8A/DRB_6h_Merged.tif]

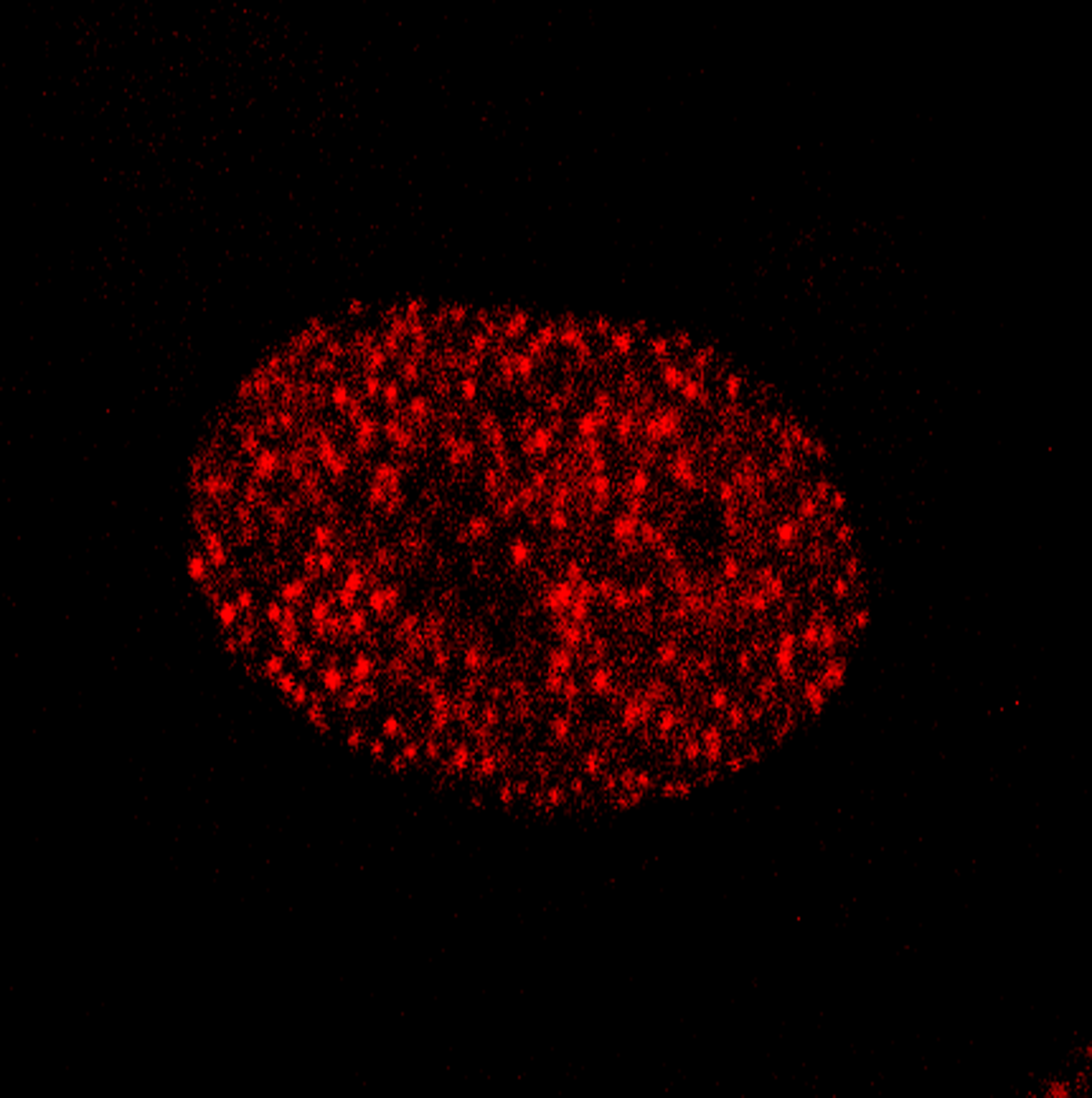

Supplement: Supplementary file 10 — Source data Fig. 8 [file 44318_2026_783_MOESM10_ESM.zip › Figure 8/Figure 8A/UT_gH2AX.tif]

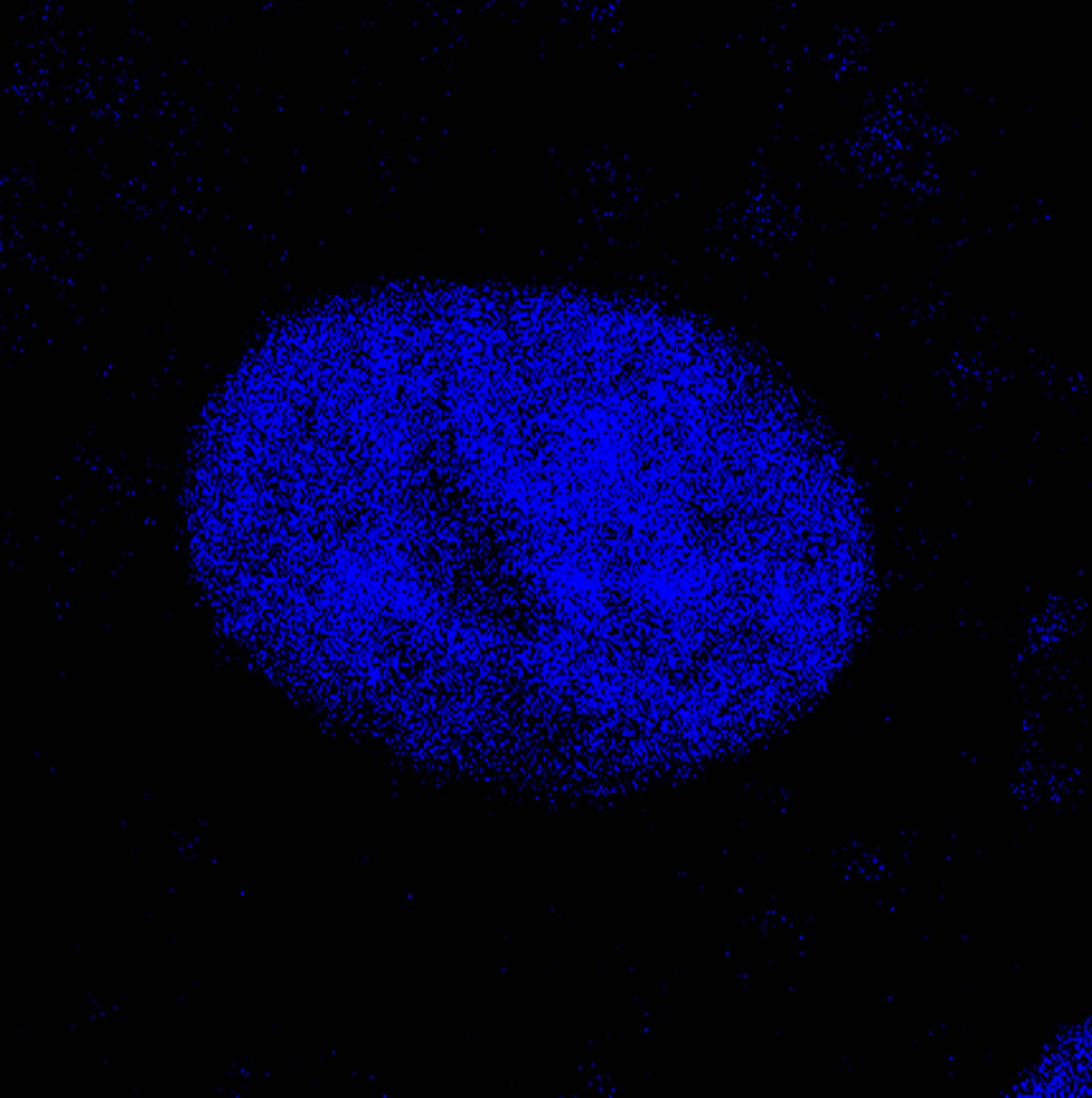

Supplement: Supplementary file 10 — Source data Fig. 8 [file 44318_2026_783_MOESM10_ESM.zip › Figure 8/Figure 8A/UT_Hoechst.tif]

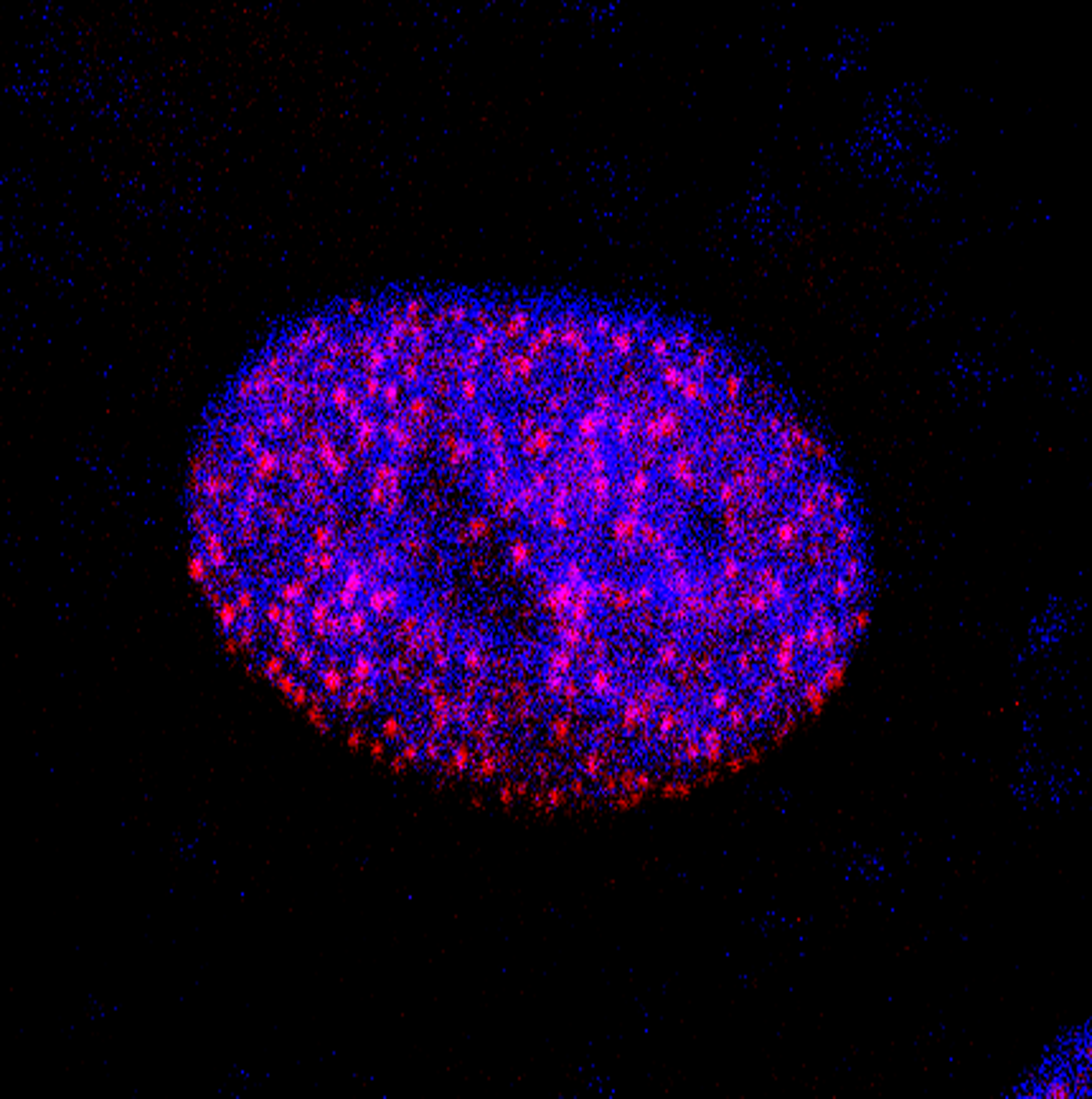

Supplement: Supplementary file 10 — Source data Fig. 8 [file 44318_2026_783_MOESM10_ESM.zip › Figure 8/Figure 8A/UT_Merged.tif]

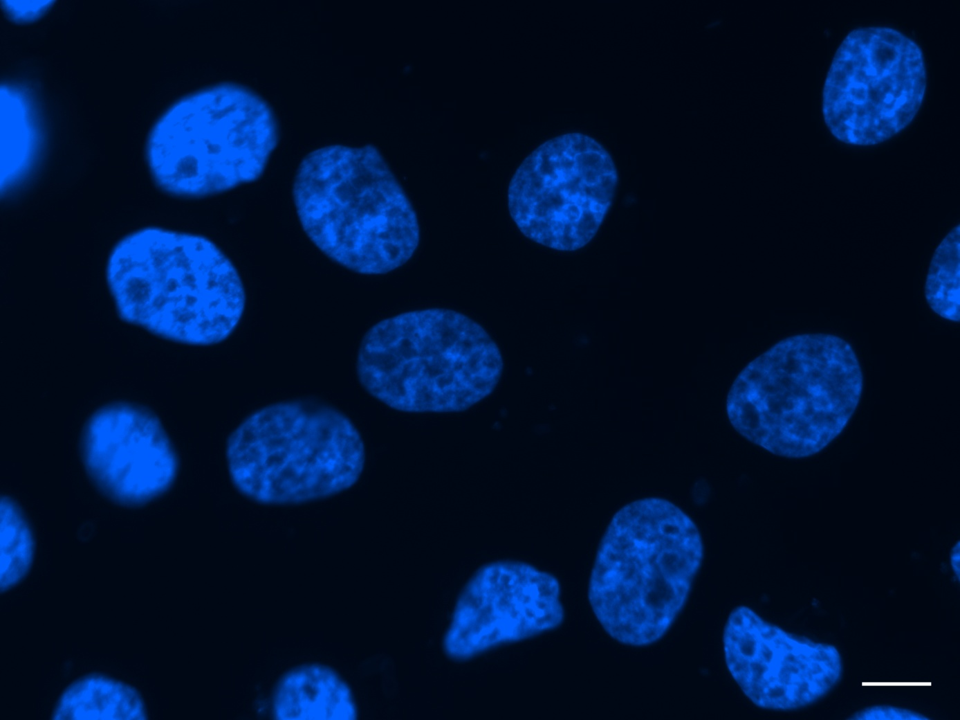

Supplement: Supplementary file 10 — Source data Fig. 8 [file 44318_2026_783_MOESM10_ESM.zip › Figure 8/Figure 8C/RNaseH Control/S320A_Hoechst.tif]

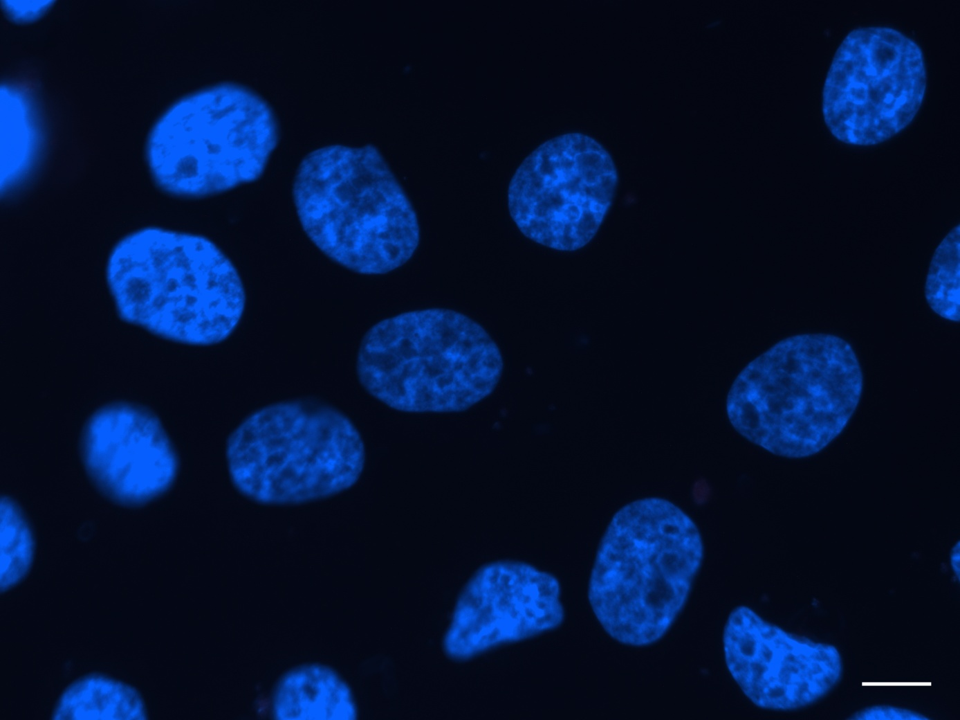

Supplement: Supplementary file 10 — Source data Fig. 8 [file 44318_2026_783_MOESM10_ESM.zip › Figure 8/Figure 8C/RNaseH Control/S320A_Merged.tif]

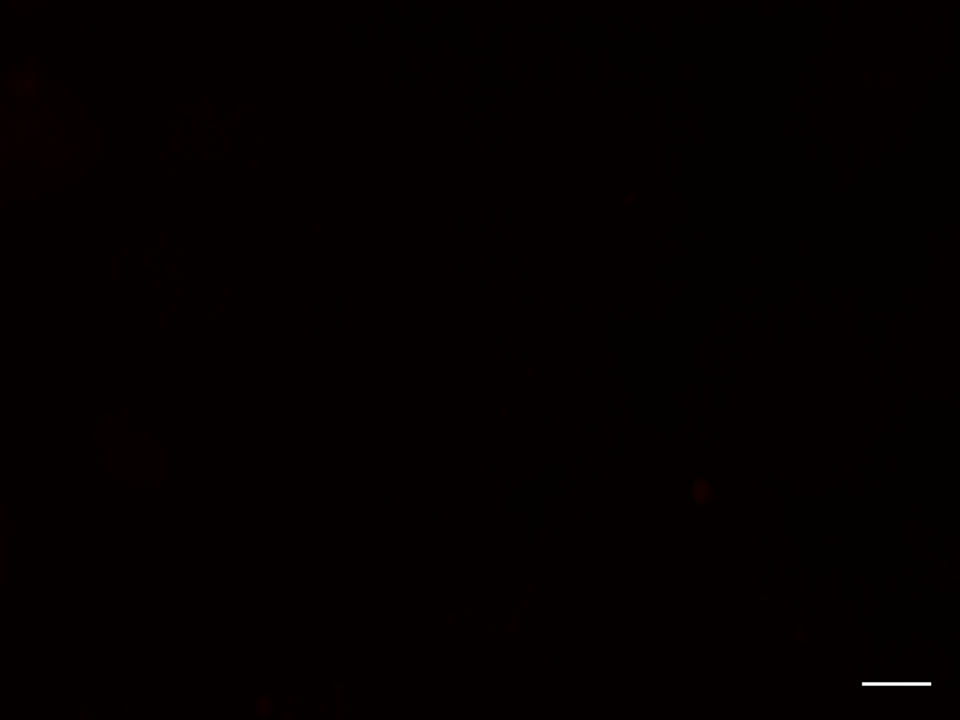

Supplement: Supplementary file 10 — Source data Fig. 8 [file 44318_2026_783_MOESM10_ESM.zip › Figure 8/Figure 8C/RNaseH Control/S320A_S9.6.tif]

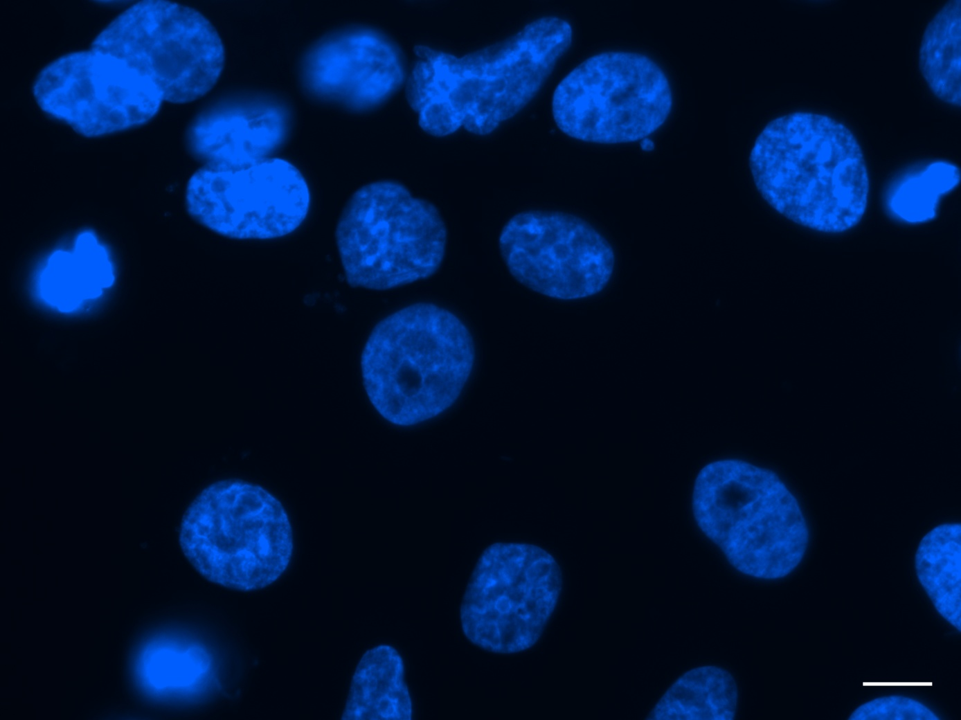

Supplement: Supplementary file 10 — Source data Fig. 8 [file 44318_2026_783_MOESM10_ESM.zip › Figure 8/Figure 8C/RNaseH Control/WT_Hoechst.tif]

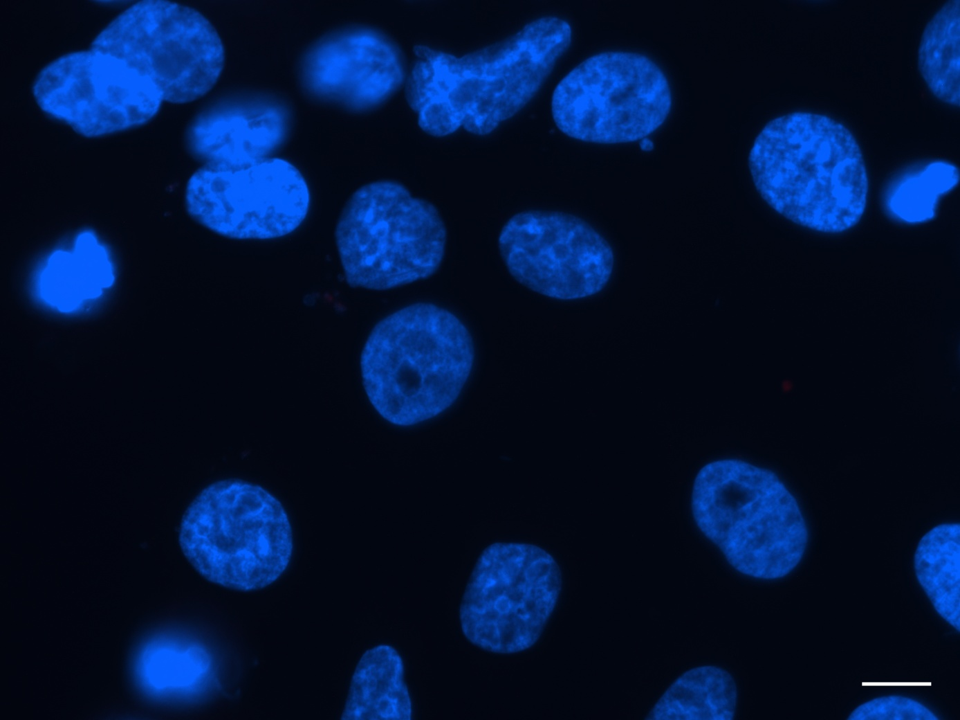

Supplement: Supplementary file 10 — Source data Fig. 8 [file 44318_2026_783_MOESM10_ESM.zip › Figure 8/Figure 8C/RNaseH Control/WT_Merged.tif]

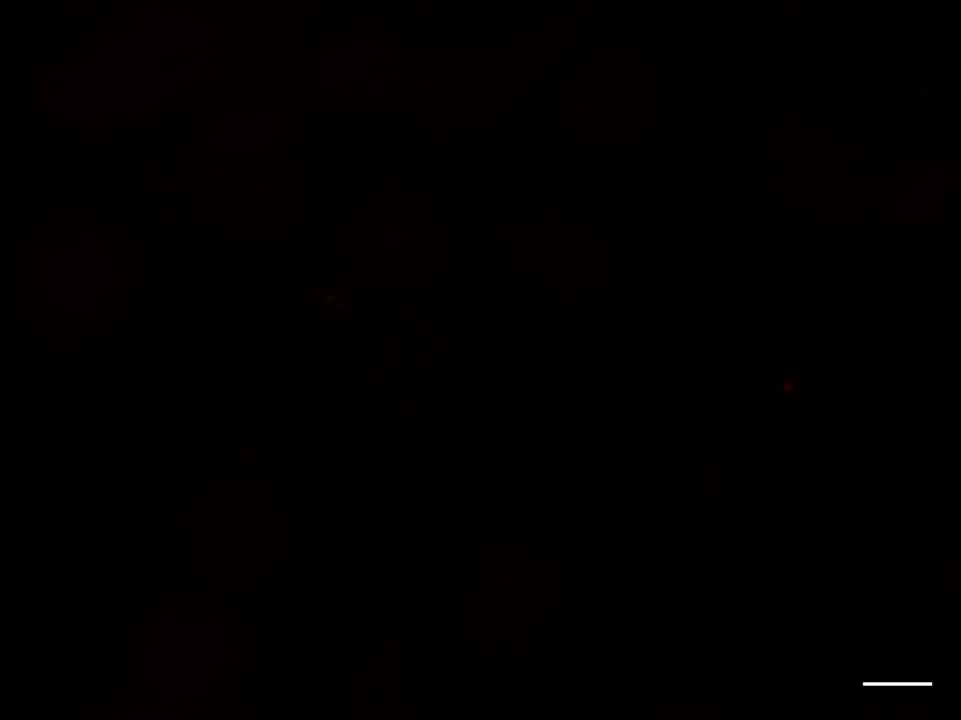

Supplement: Supplementary file 10 — Source data Fig. 8 [file 44318_2026_783_MOESM10_ESM.zip › Figure 8/Figure 8C/RNaseH Control/WT_S9.6.tif]

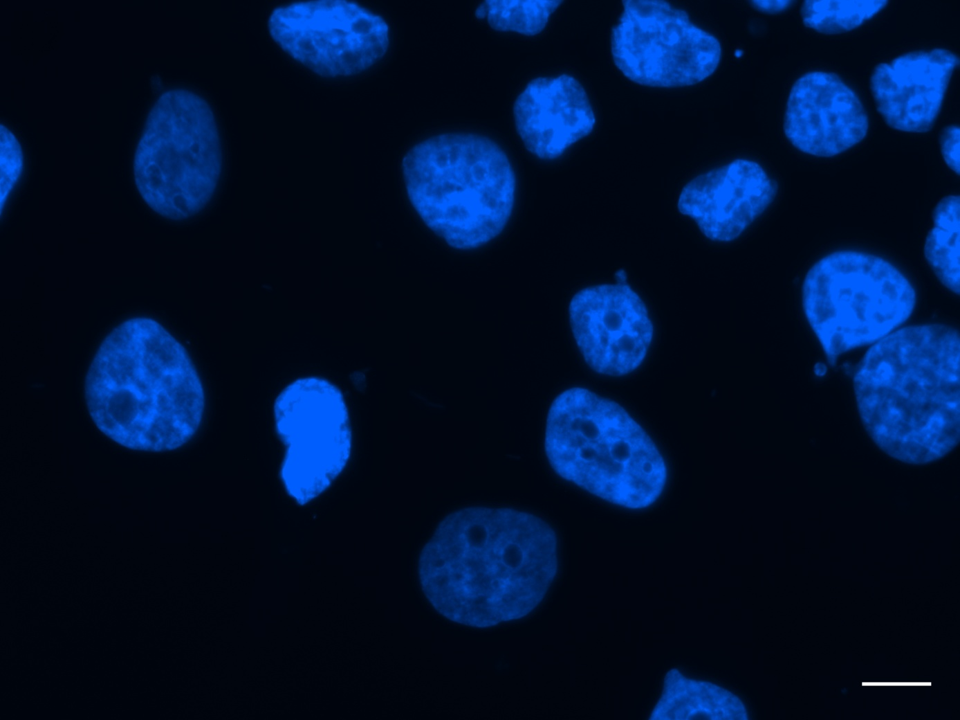

Supplement: Supplementary file 10 — Source data Fig. 8 [file 44318_2026_783_MOESM10_ESM.zip › Figure 8/Figure 8C/Without RNaseH/S320A_Hoechst.tif]

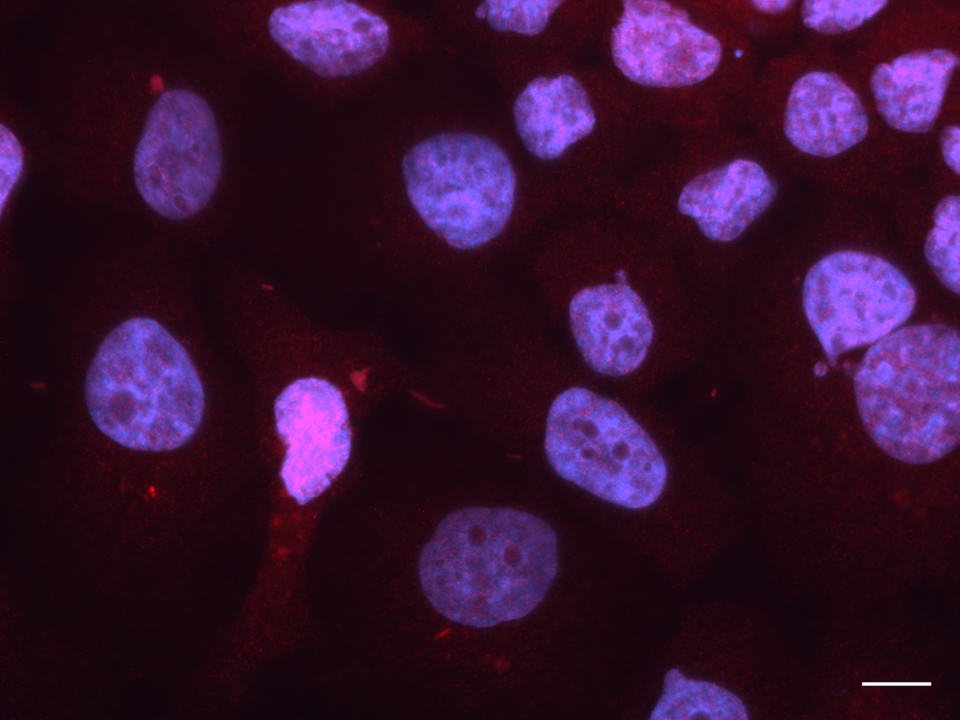

Supplement: Supplementary file 10 — Source data Fig. 8 [file 44318_2026_783_MOESM10_ESM.zip › Figure 8/Figure 8C/Without RNaseH/S320A_Merged.tif]
