# Supplementary figures and images for: A CHK1-mediated phosphorylation switch suppresses human Topoisomerase 1-associated genomic instability (part 3 of 3)
Source: EMBO J. 2026 May 13;45(12):4220–56. doi: 10.1038/s44318-026-00783-3 (PMC13270093; doi:10.1038/s44318-026-00783-3)

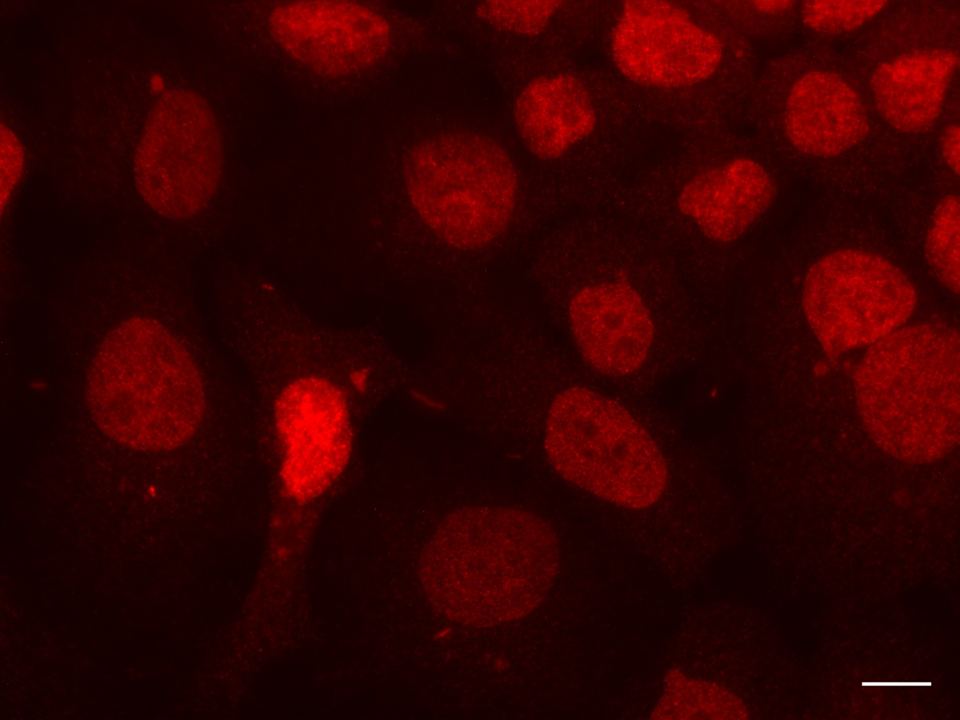

Supplement: Supplementary file 10 — Source data Fig. 8 [file 44318_2026_783_MOESM10_ESM.zip › Figure 8/Figure 8C/Without RNaseH/S320A_S9.6.tif]

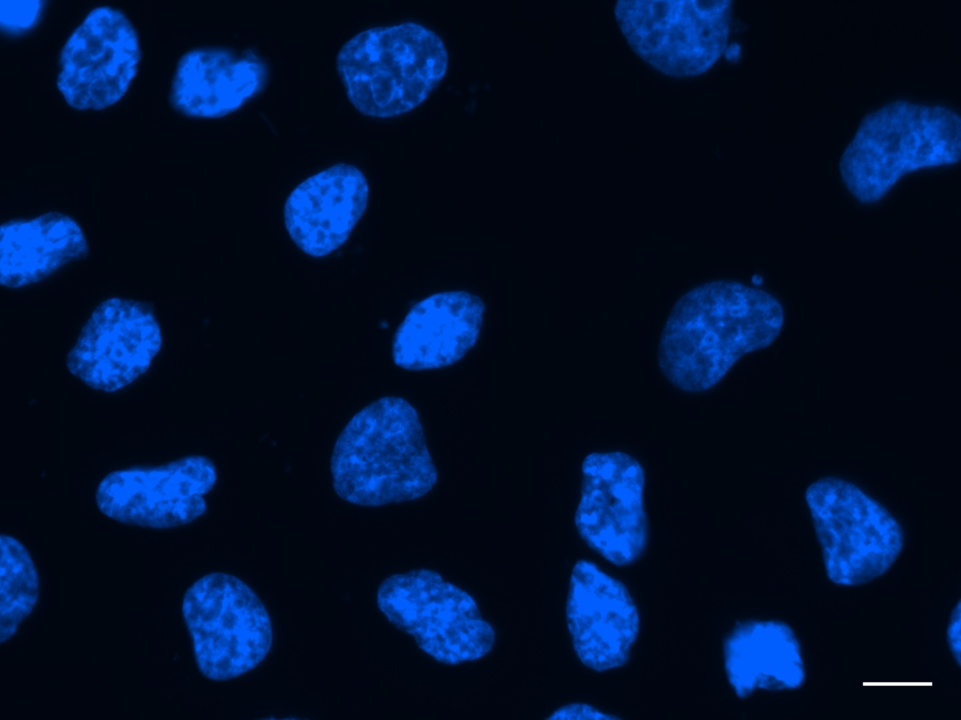

Supplement: Supplementary file 10 — Source data Fig. 8 [file 44318_2026_783_MOESM10_ESM.zip › Figure 8/Figure 8C/Without RNaseH/WT_Hoechst.tif]

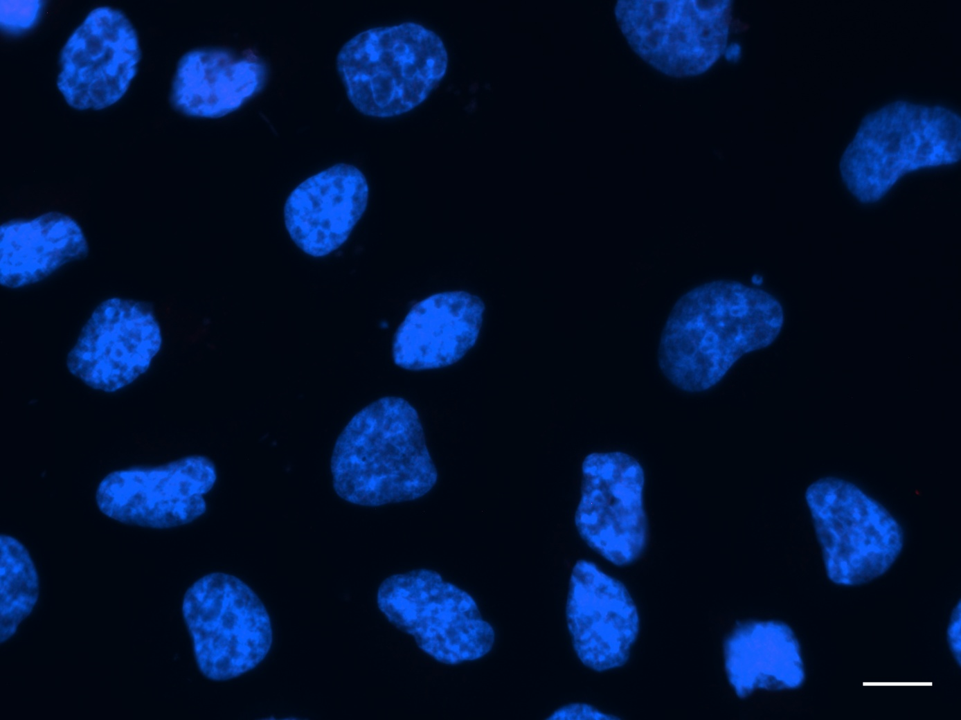

Supplement: Supplementary file 10 — Source data Fig. 8 [file 44318_2026_783_MOESM10_ESM.zip › Figure 8/Figure 8C/Without RNaseH/WT_Merged.tif]

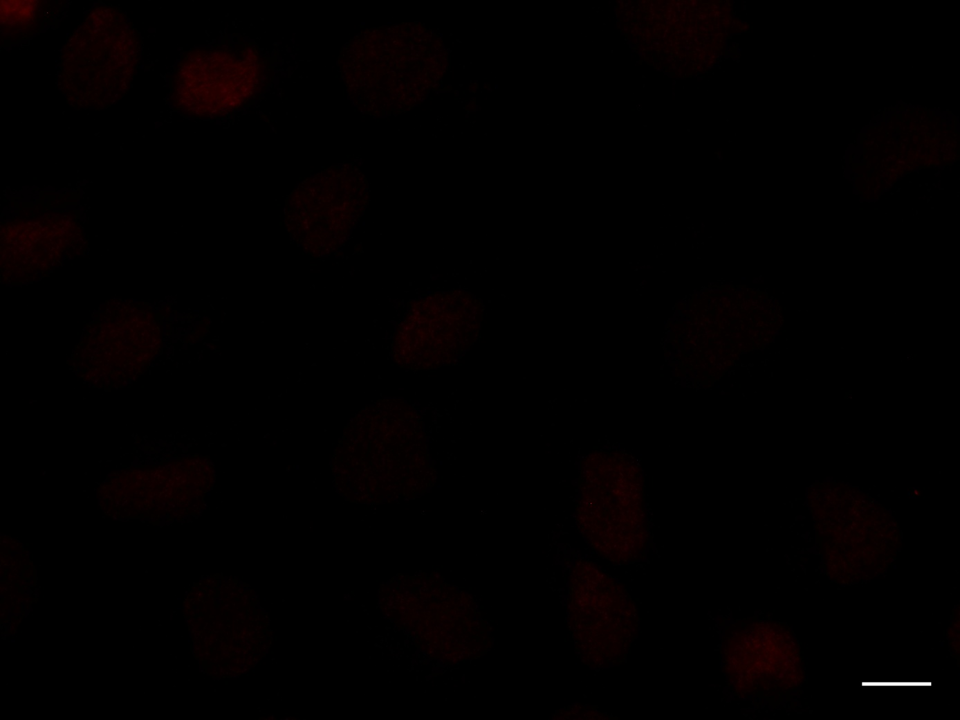

Supplement: Supplementary file 10 — Source data Fig. 8 [file 44318_2026_783_MOESM10_ESM.zip › Figure 8/Figure 8C/Without RNaseH/WT_S9.6.tif]

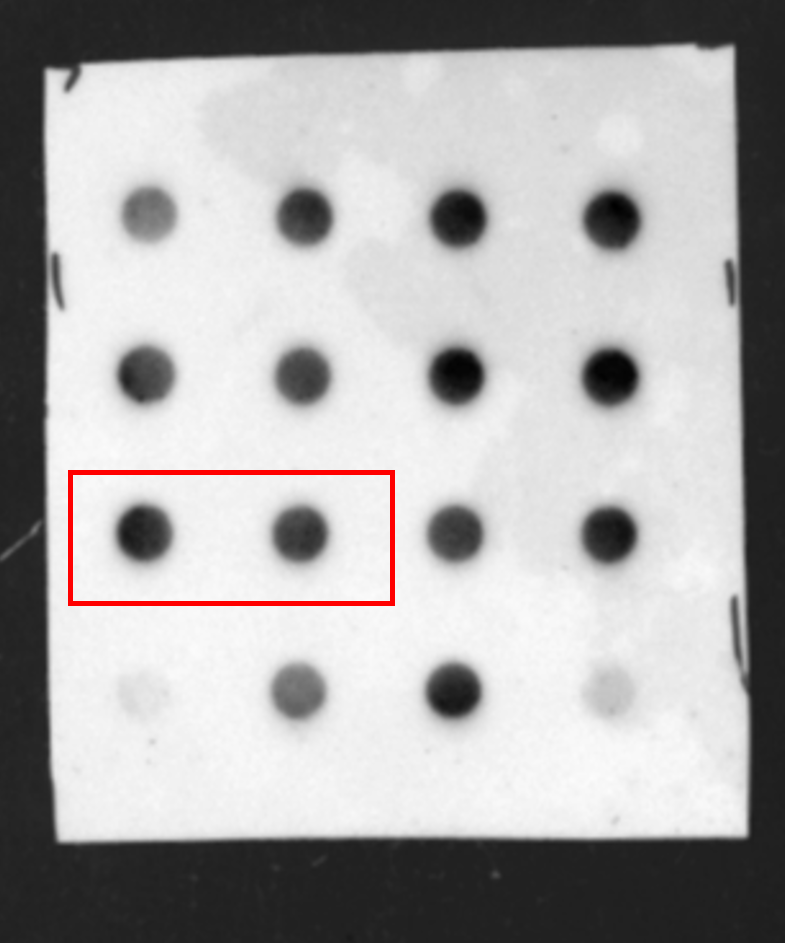

Supplement: Supplementary file 10 — Source data Fig. 8 [file 44318_2026_783_MOESM10_ESM.zip › Figure 8/Figure 8F/DNA.tif]

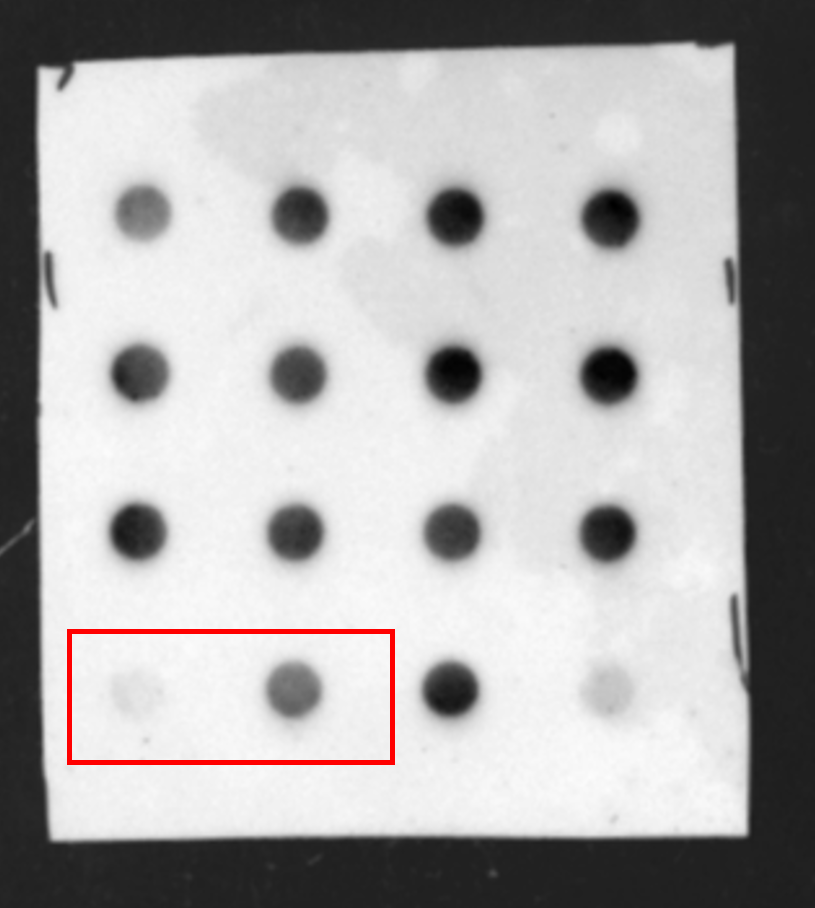

Supplement: Supplementary file 10 — Source data Fig. 8 [file 44318_2026_783_MOESM10_ESM.zip › Figure 8/Figure 8F/S9.6_NoRNaseH.tif]

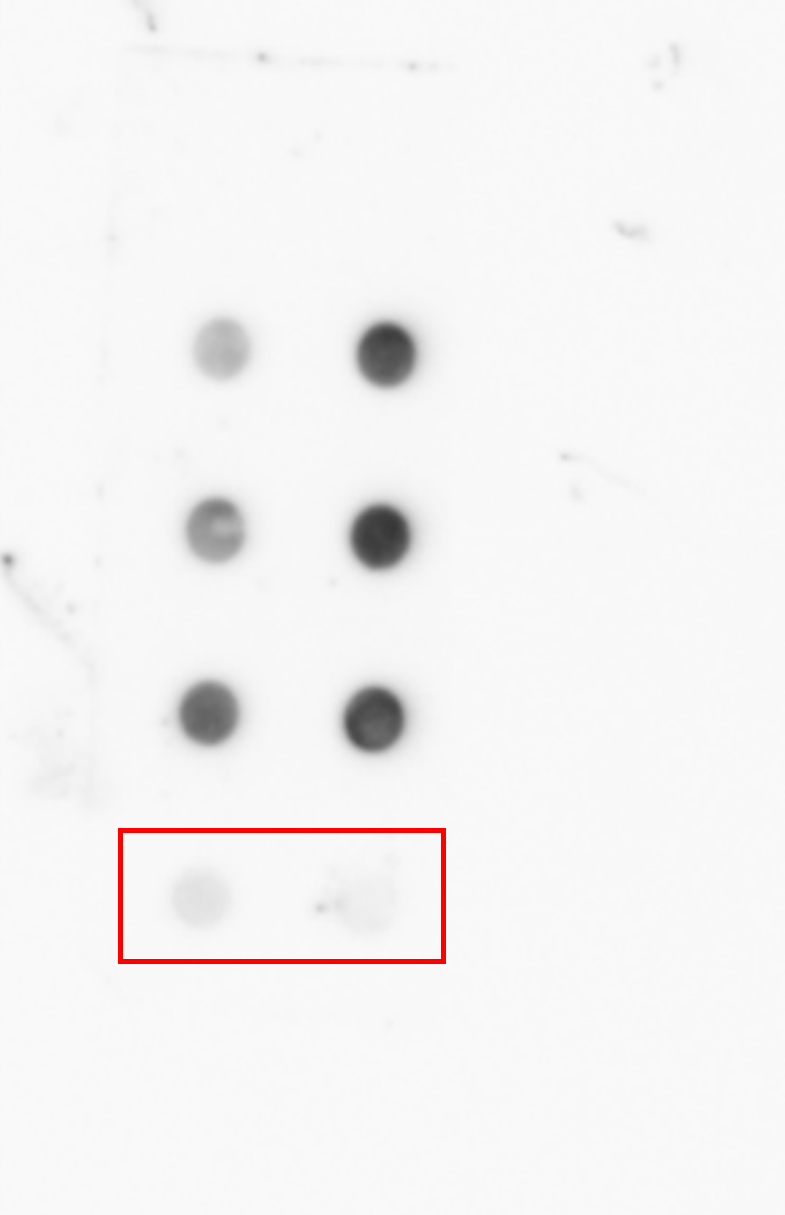

Supplement: Supplementary file 10 — Source data Fig. 8 [file 44318_2026_783_MOESM10_ESM.zip › Figure 8/Figure 8F/S9.6_RNaseH.tif]

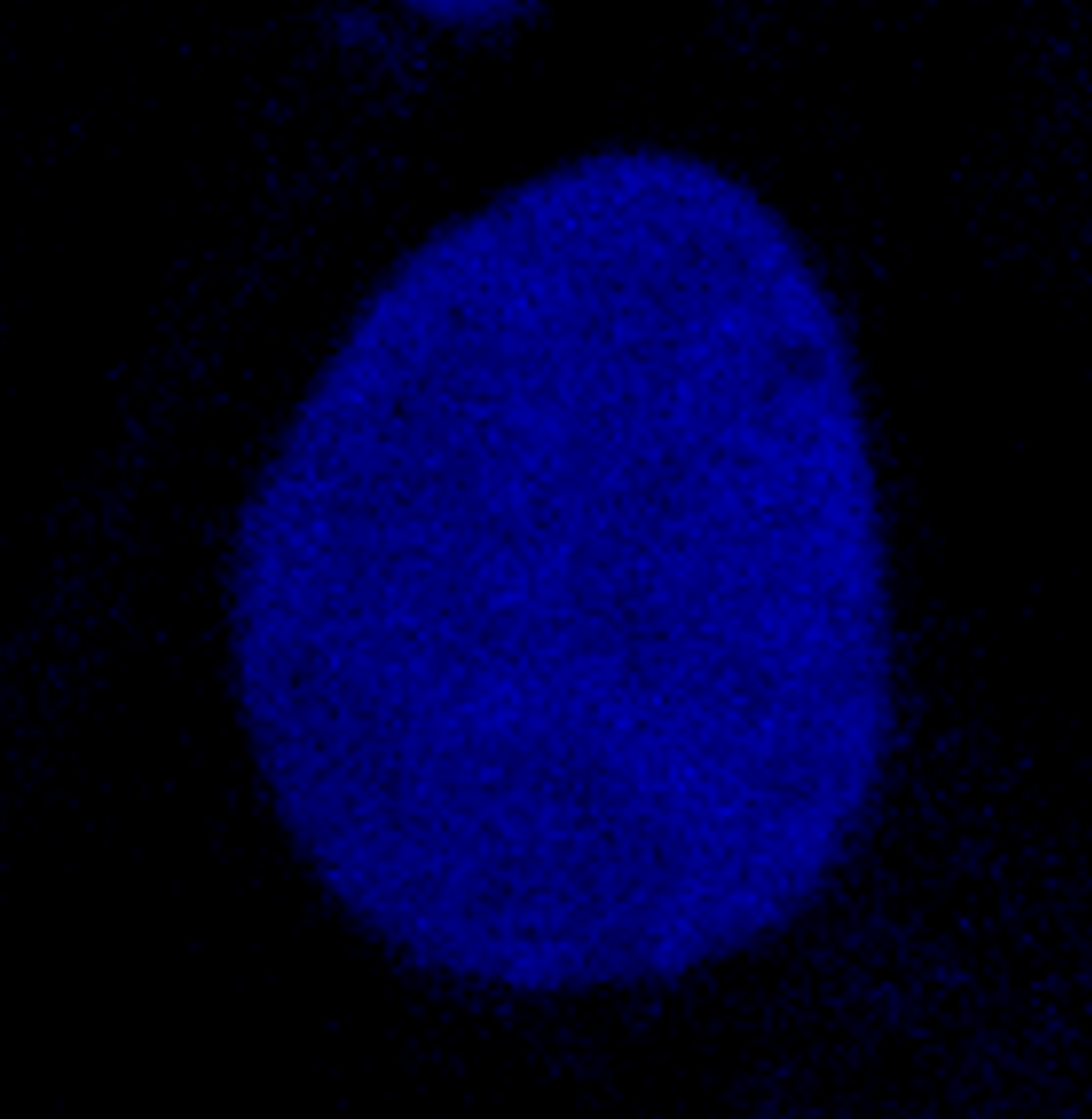

Supplement: Supplementary file 10 — Source data Fig. 8 [file 44318_2026_783_MOESM10_ESM.zip › Figure 8/Figure 8H/S320A_Hoechst.tif]

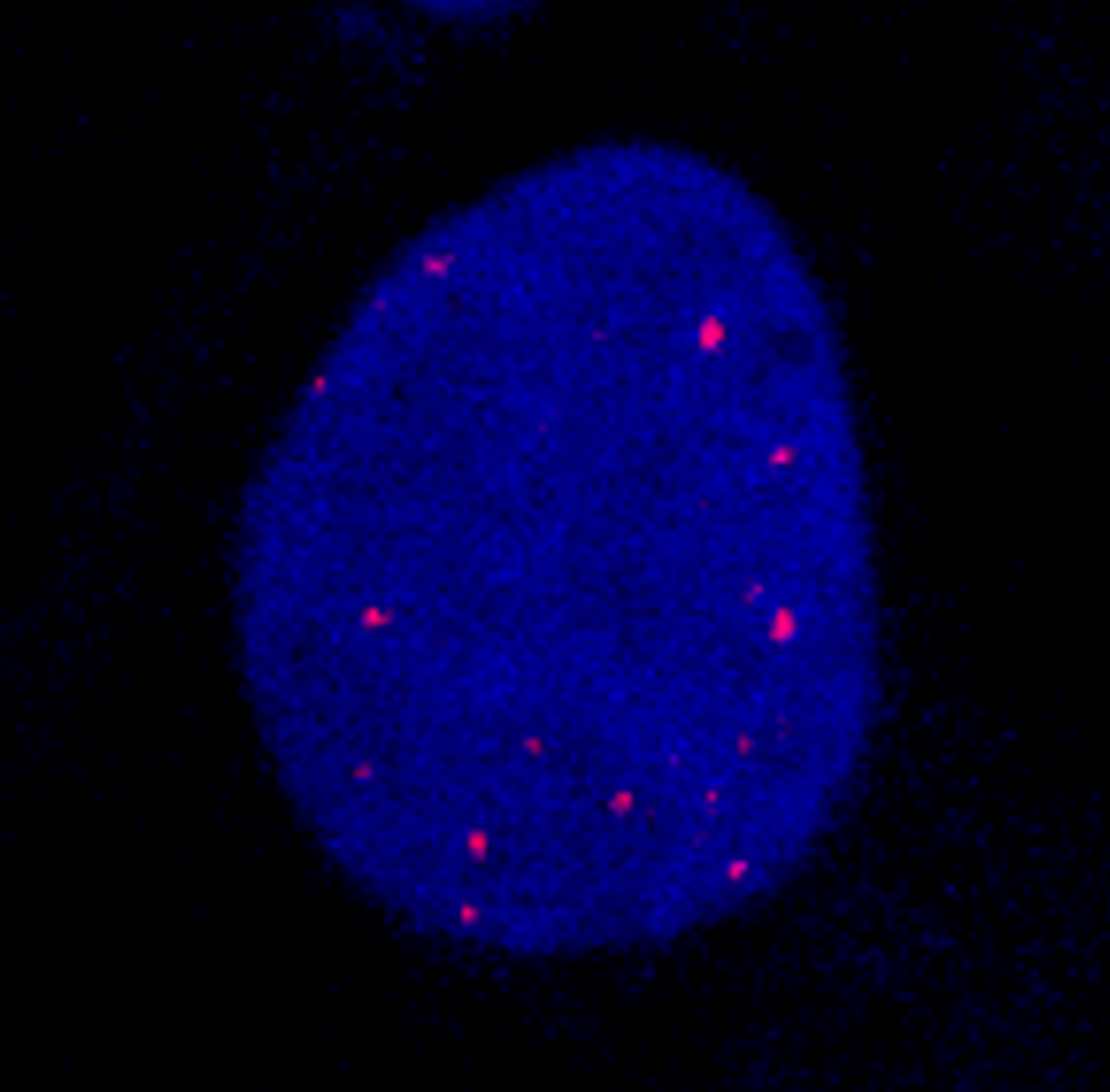

Supplement: Supplementary file 10 — Source data Fig. 8 [file 44318_2026_783_MOESM10_ESM.zip › Figure 8/Figure 8H/S320A_Merged.tif]

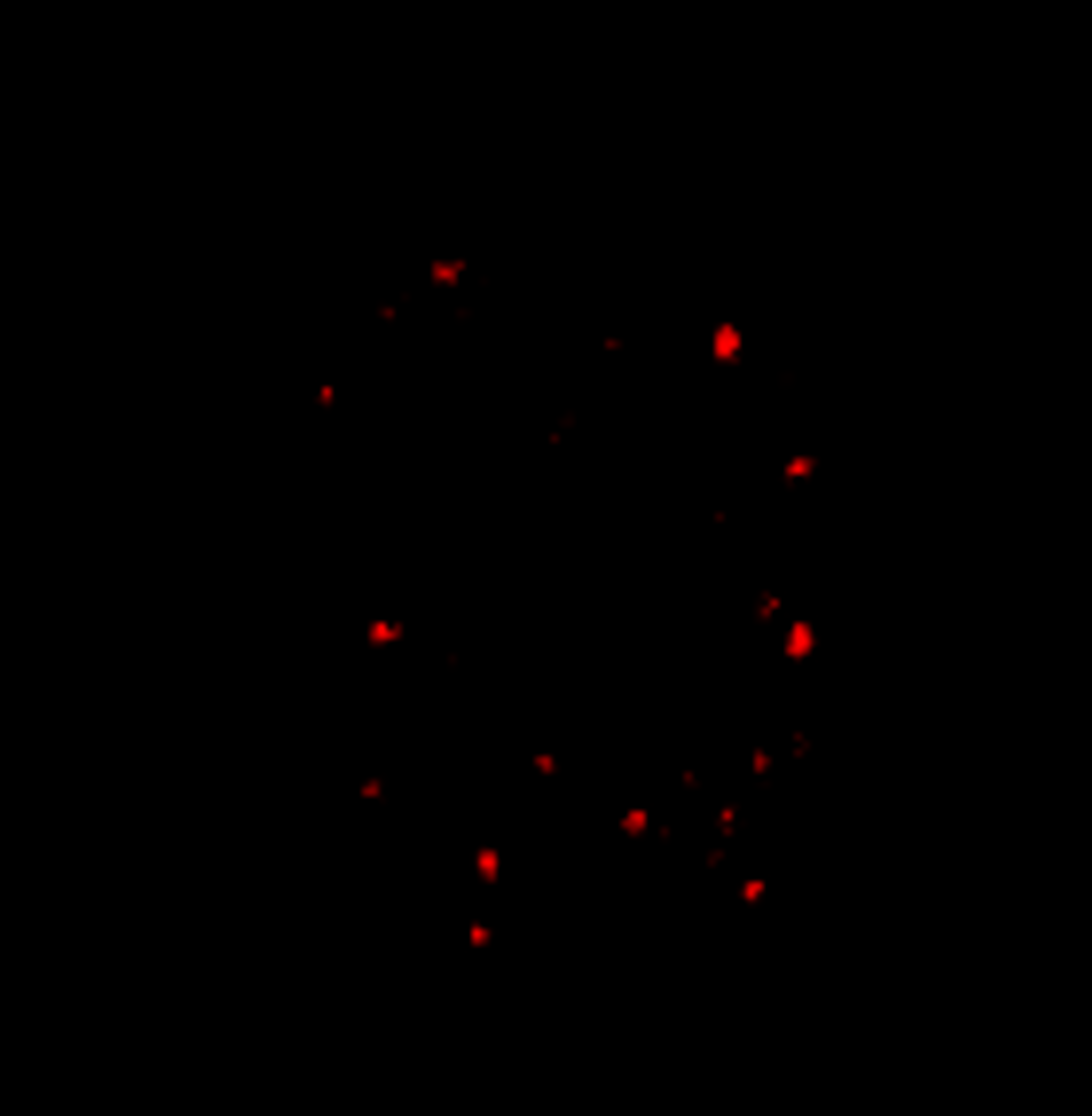

Supplement: Supplementary file 10 — Source data Fig. 8 [file 44318_2026_783_MOESM10_ESM.zip › Figure 8/Figure 8H/S320A_PLA.tif]

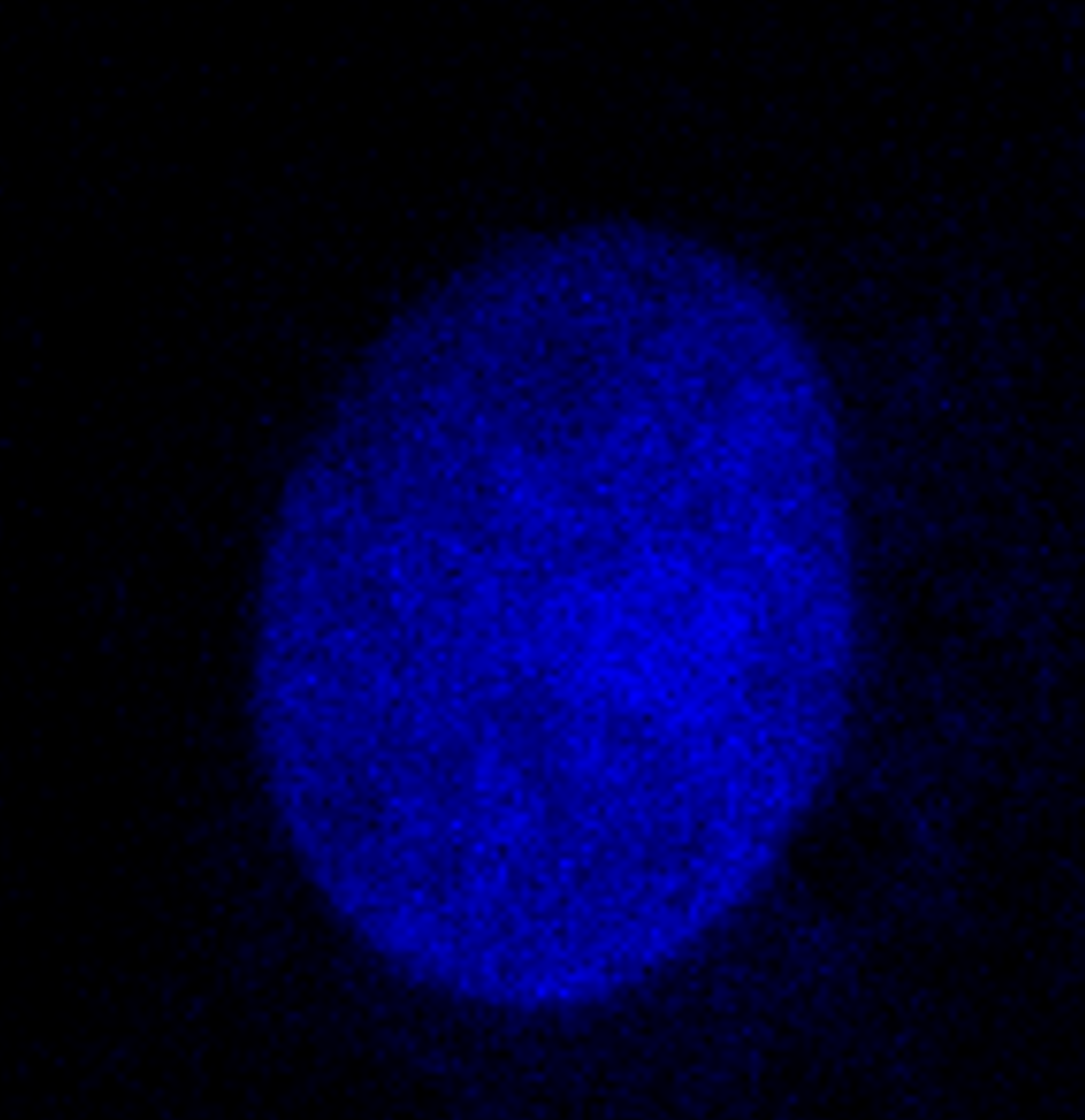

Supplement: Supplementary file 10 — Source data Fig. 8 [file 44318_2026_783_MOESM10_ESM.zip › Figure 8/Figure 8H/WT_Hoechst.tif]

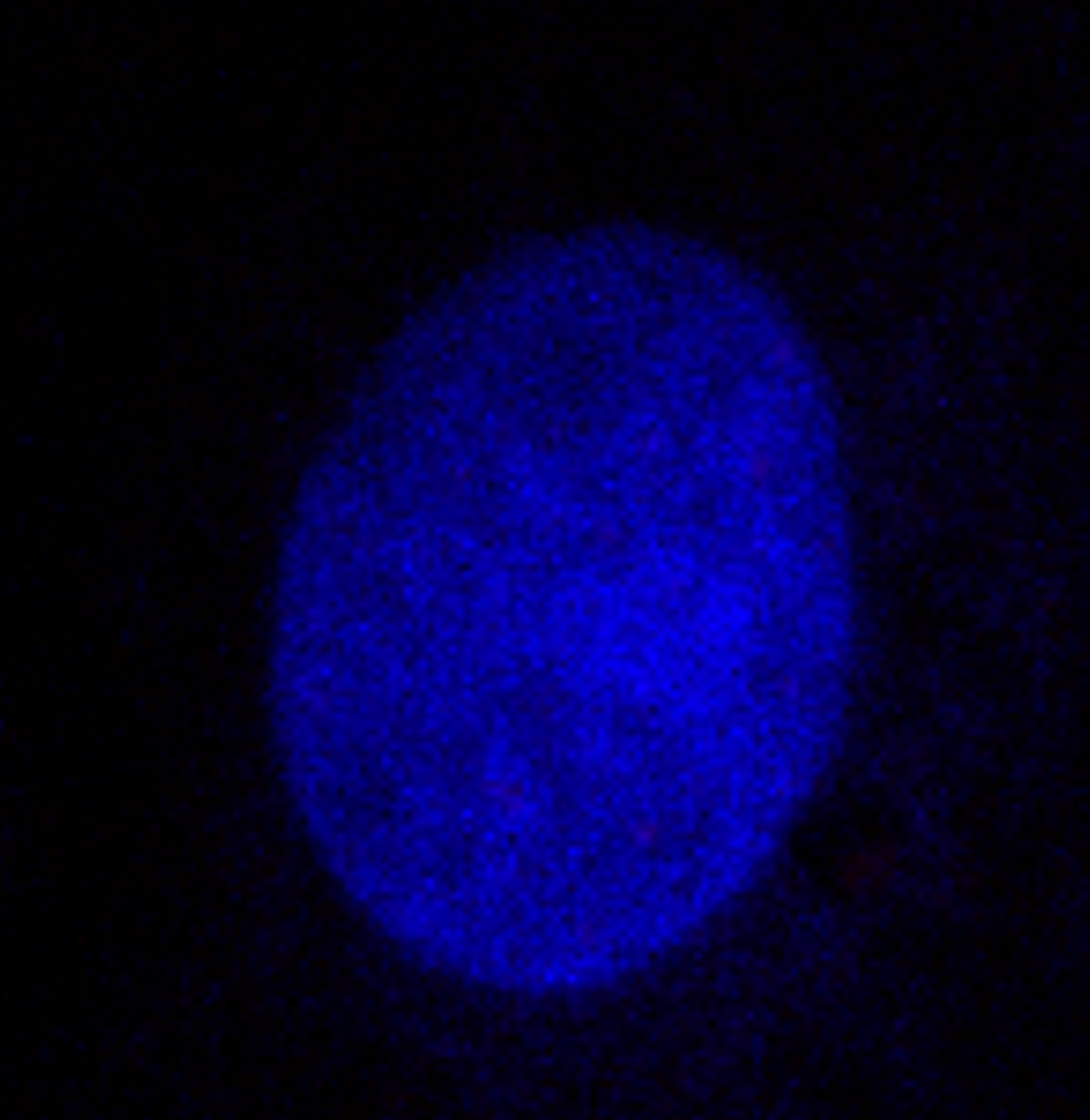

Supplement: Supplementary file 10 — Source data Fig. 8 [file 44318_2026_783_MOESM10_ESM.zip › Figure 8/Figure 8H/WT_Merged.tif]

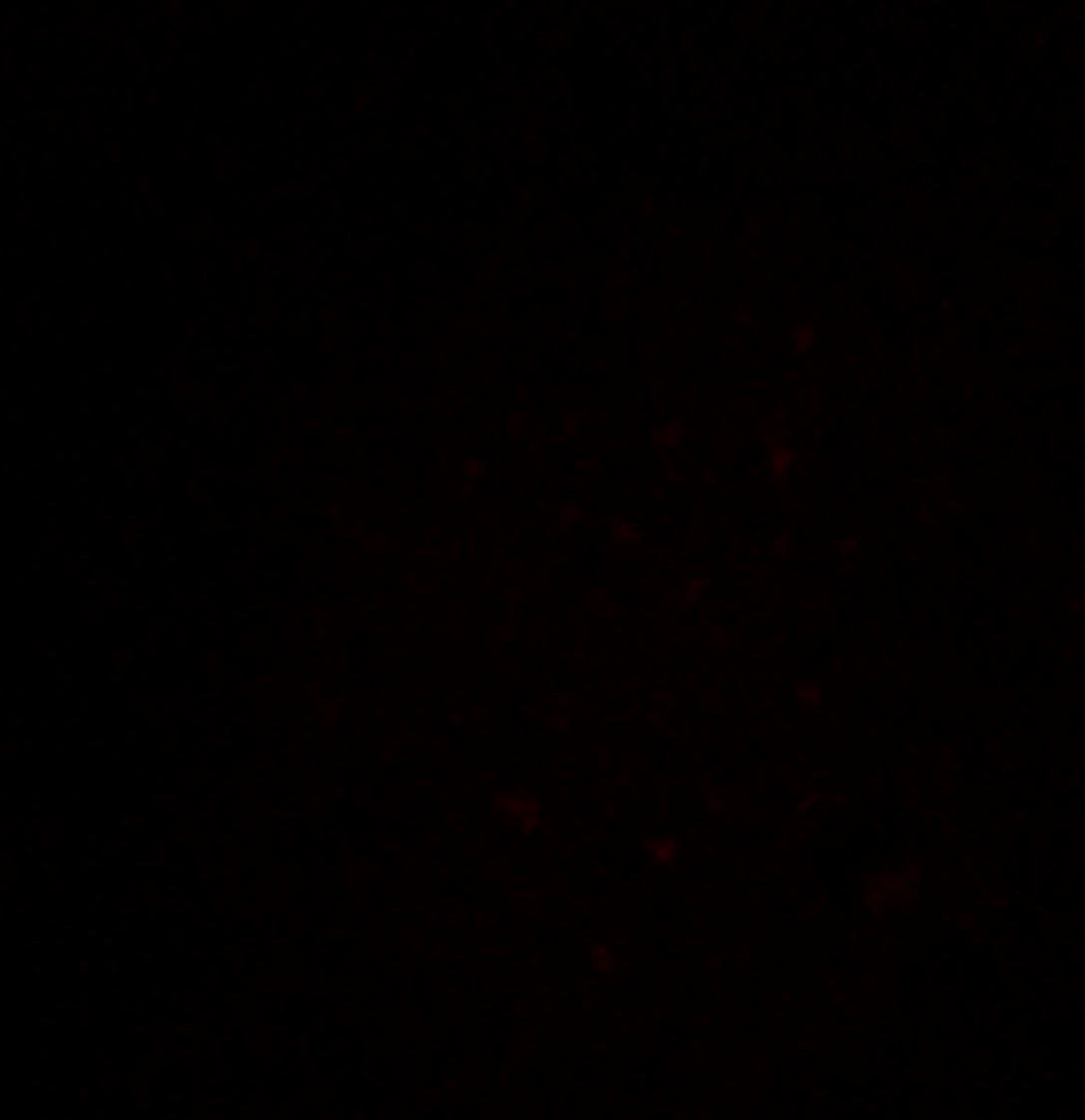

Supplement: Supplementary file 10 — Source data Fig. 8 [file 44318_2026_783_MOESM10_ESM.zip › Figure 8/Figure 8H/WT_PLA.tif]

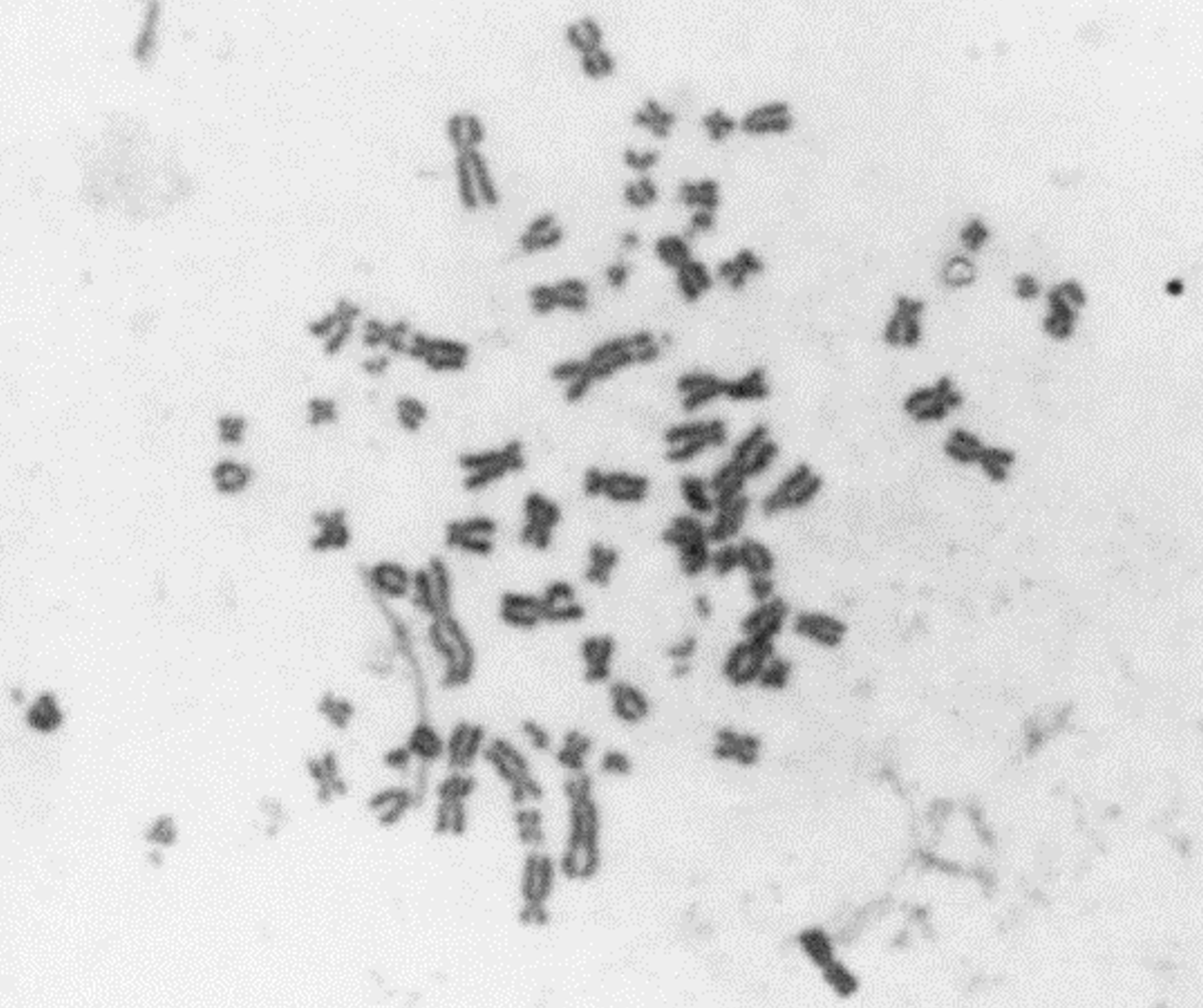

Supplement: Supplementary file 10 — Source data Fig. 8 [file 44318_2026_783_MOESM10_ESM.zip › Figure 8/Figure 8J/DRB.tif]

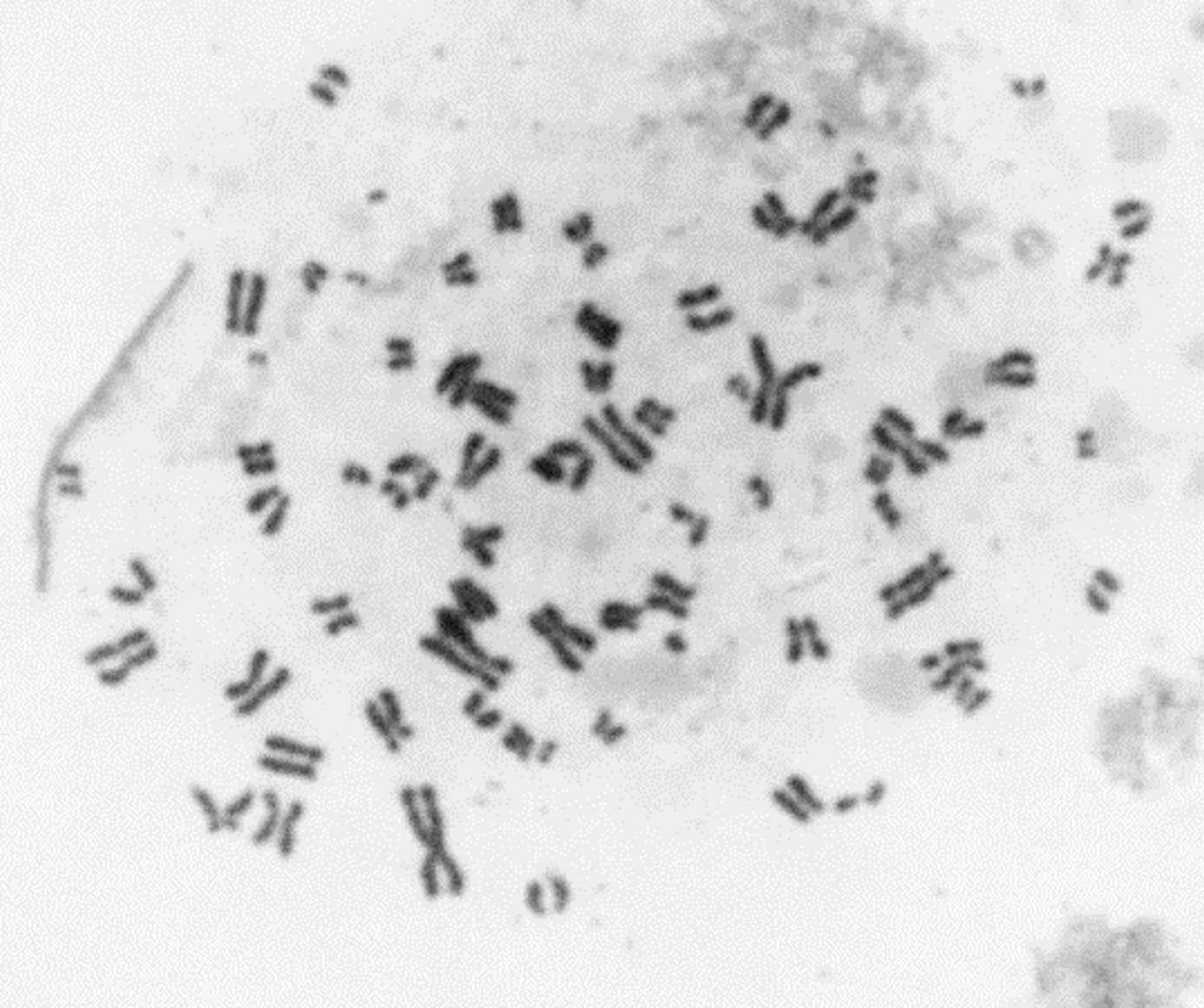

Supplement: Supplementary file 10 — Source data Fig. 8 [file 44318_2026_783_MOESM10_ESM.zip › Figure 8/Figure 8J/Untreated.tif]

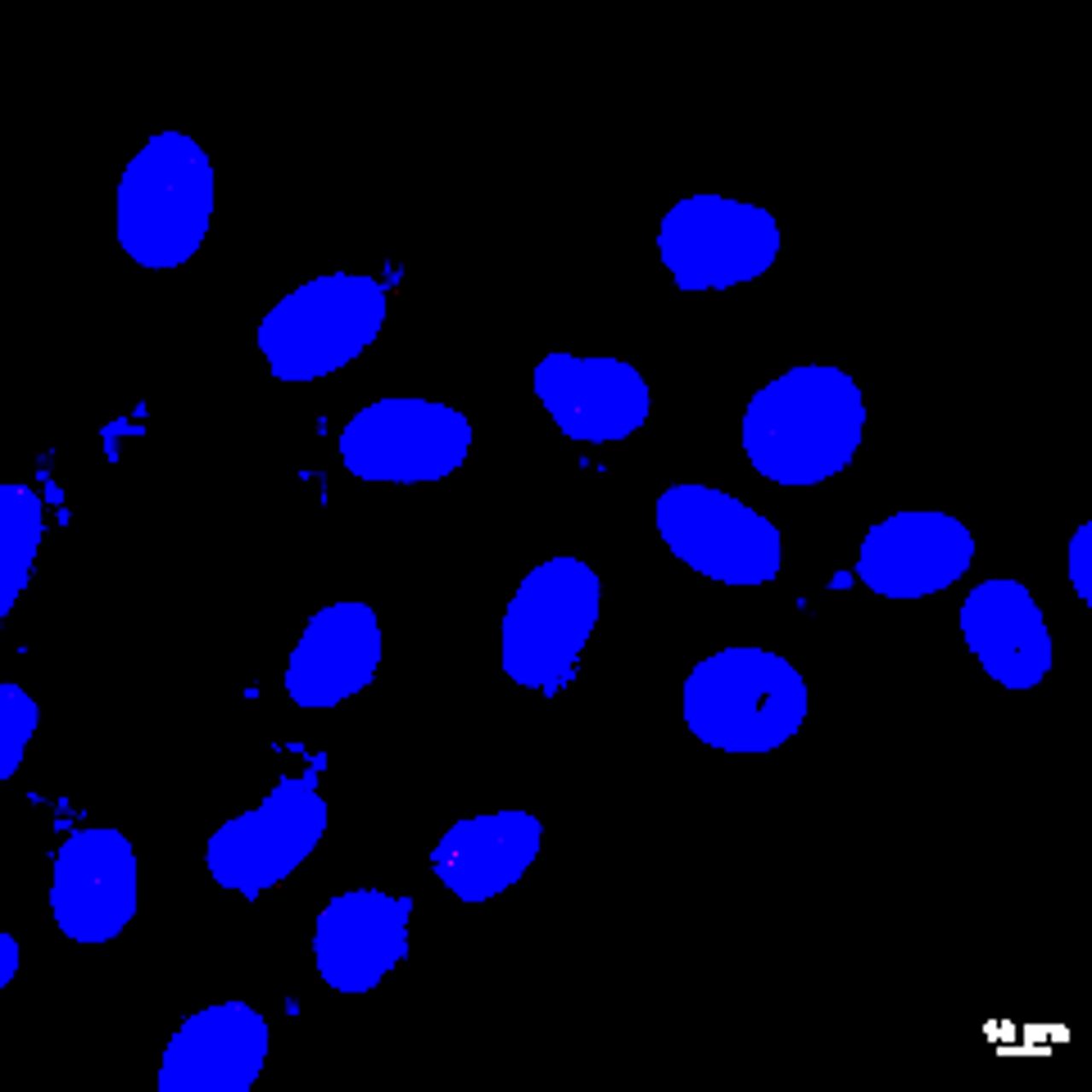

Supplement: Supplementary file 12 — Figure EV1 Source Data [file 44318_2026_783_MOESM12_ESM.zip › Figure EV1/Figure EV1A/0.5�FBS_CHK1i_Hoechst.tif]

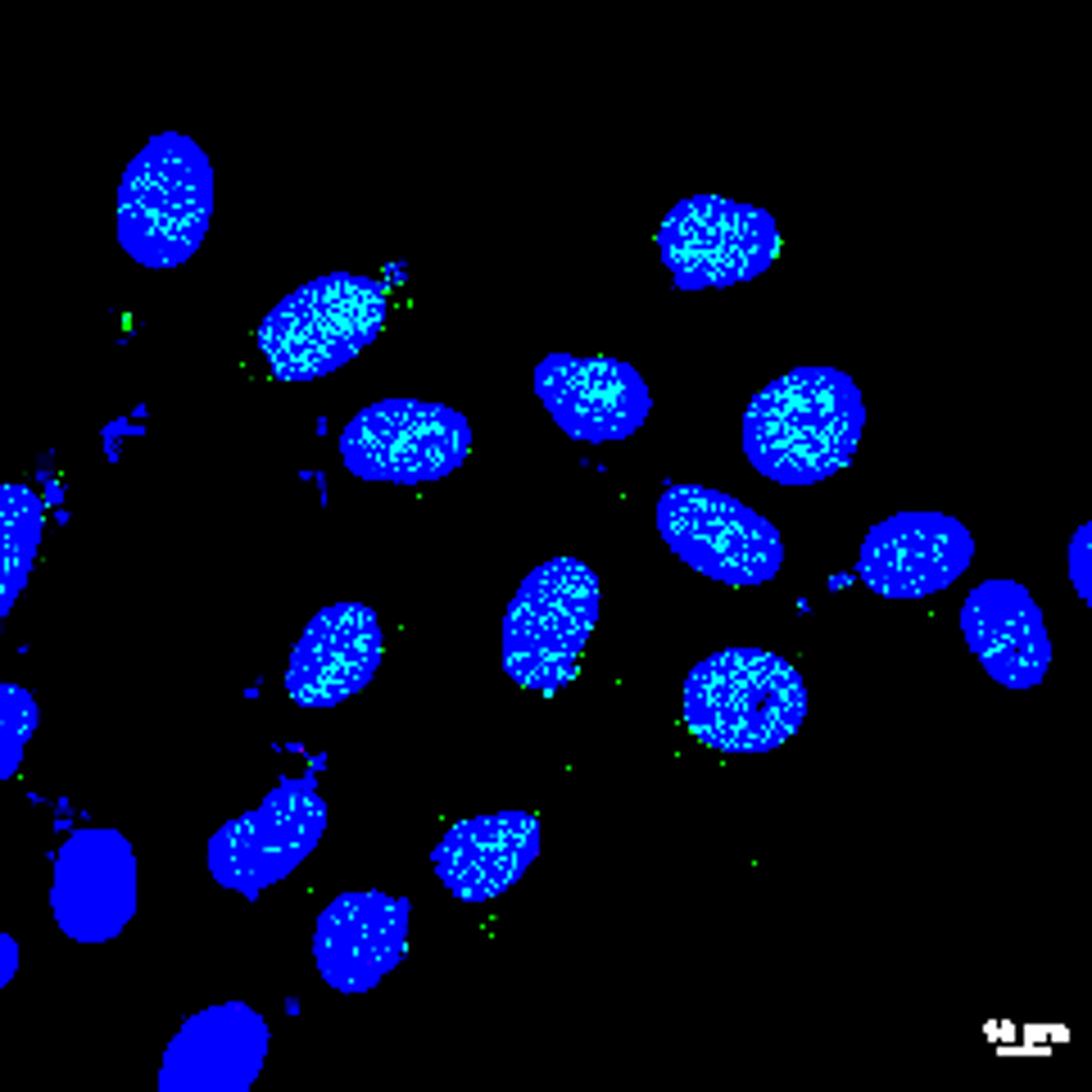

Supplement: Supplementary file 12 — Figure EV1 Source Data [file 44318_2026_783_MOESM12_ESM.zip › Figure EV1/Figure EV1A/0.5�FBS_CHK1i_Merged.tif]

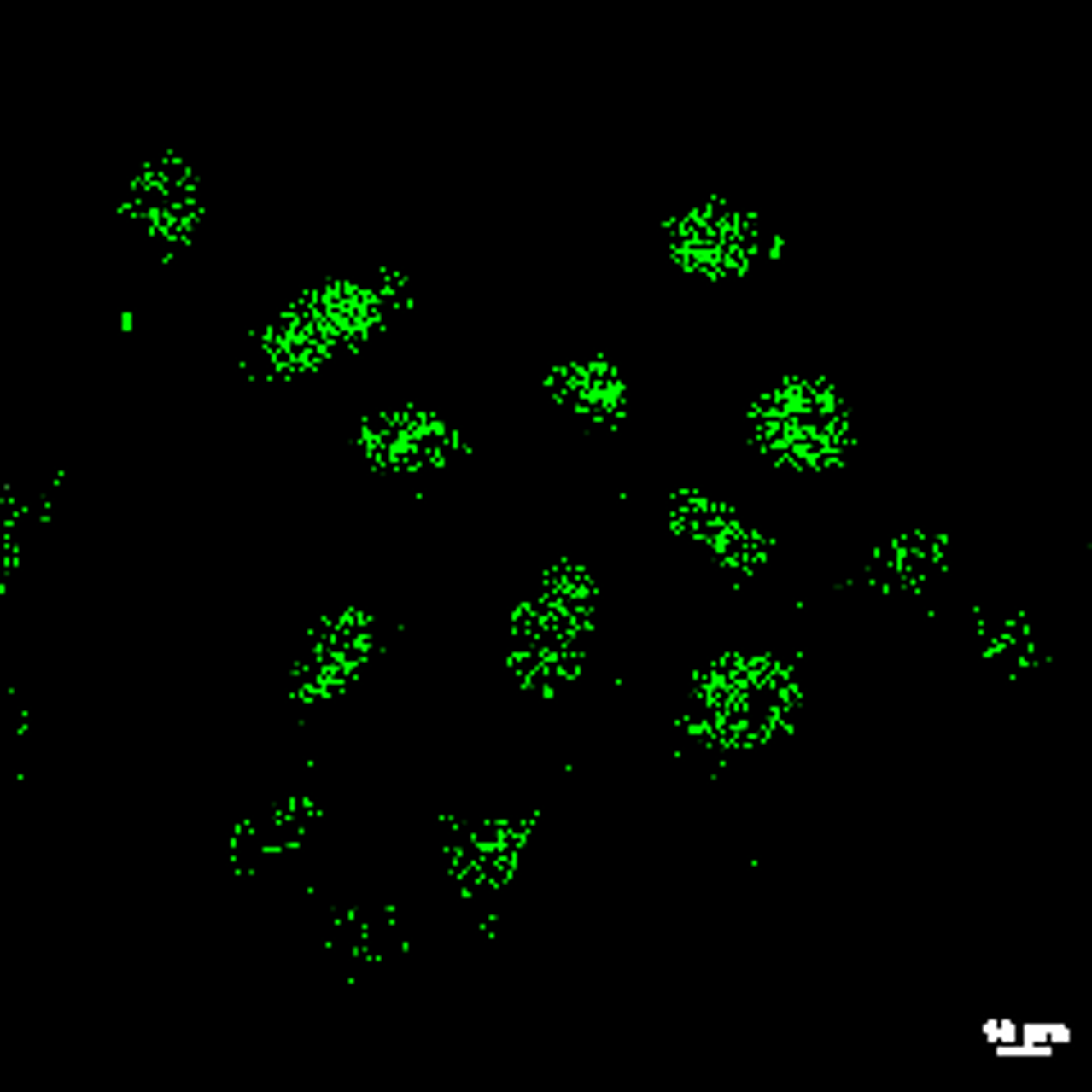

Supplement: Supplementary file 12 — Figure EV1 Source Data [file 44318_2026_783_MOESM12_ESM.zip › Figure EV1/Figure EV1A/0.5�FBS_CHK1i_TOP1cc.tif]

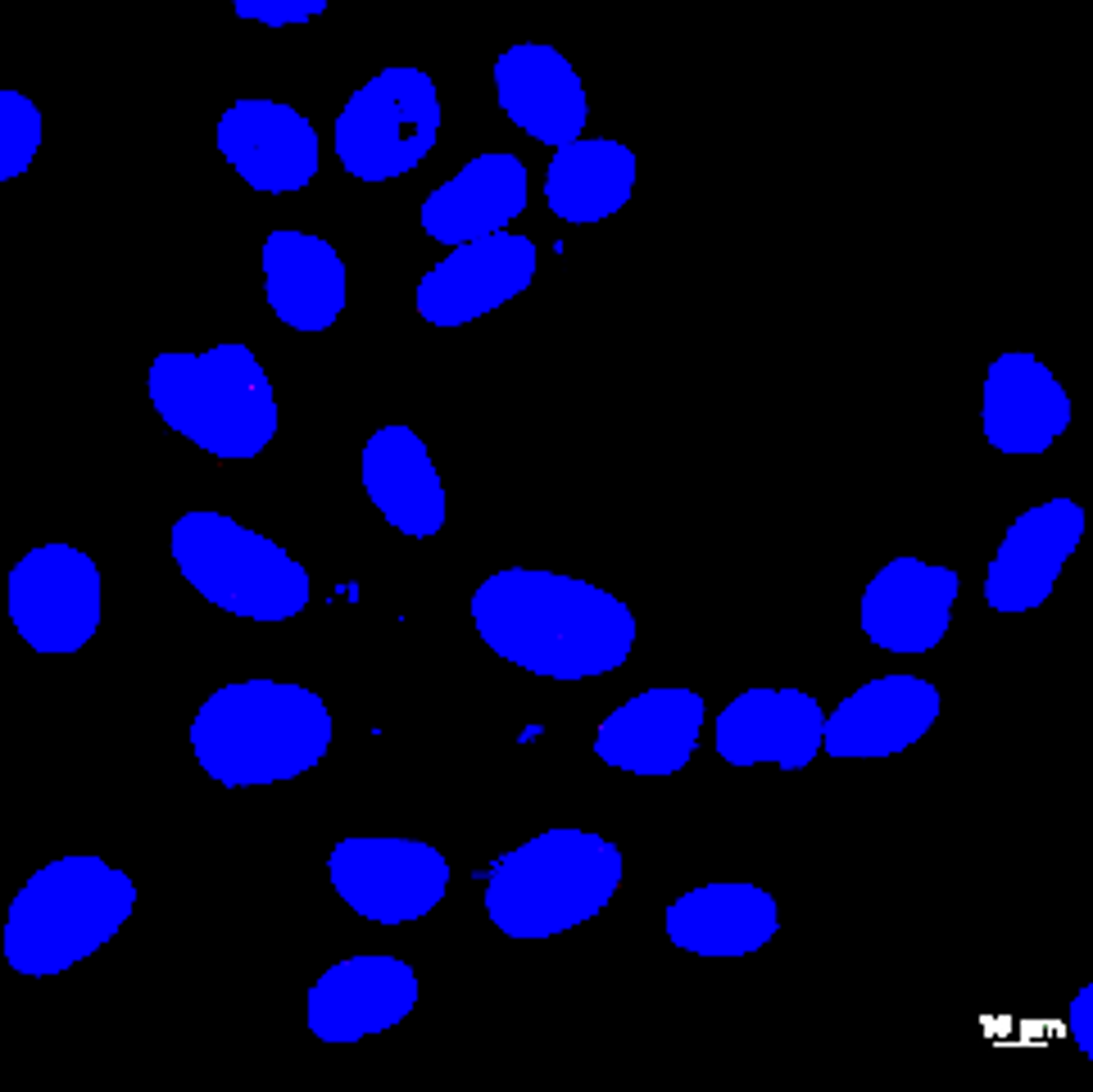

Supplement: Supplementary file 12 — Figure EV1 Source Data [file 44318_2026_783_MOESM12_ESM.zip › Figure EV1/Figure EV1A/0.5�FBS_UT_Hoechst.tif]

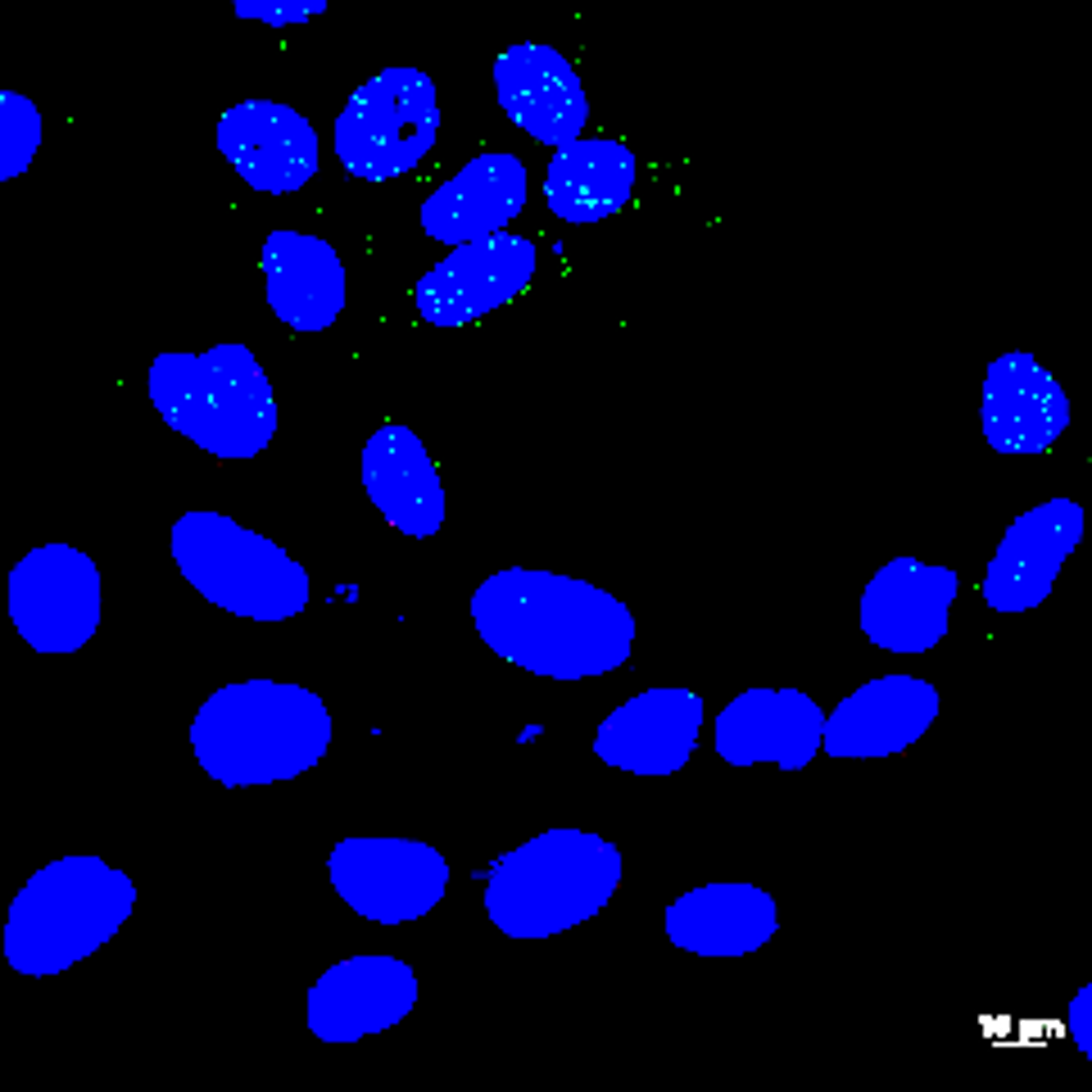

Supplement: Supplementary file 12 — Figure EV1 Source Data [file 44318_2026_783_MOESM12_ESM.zip › Figure EV1/Figure EV1A/0.5�FBS_UT_Merged.tif]

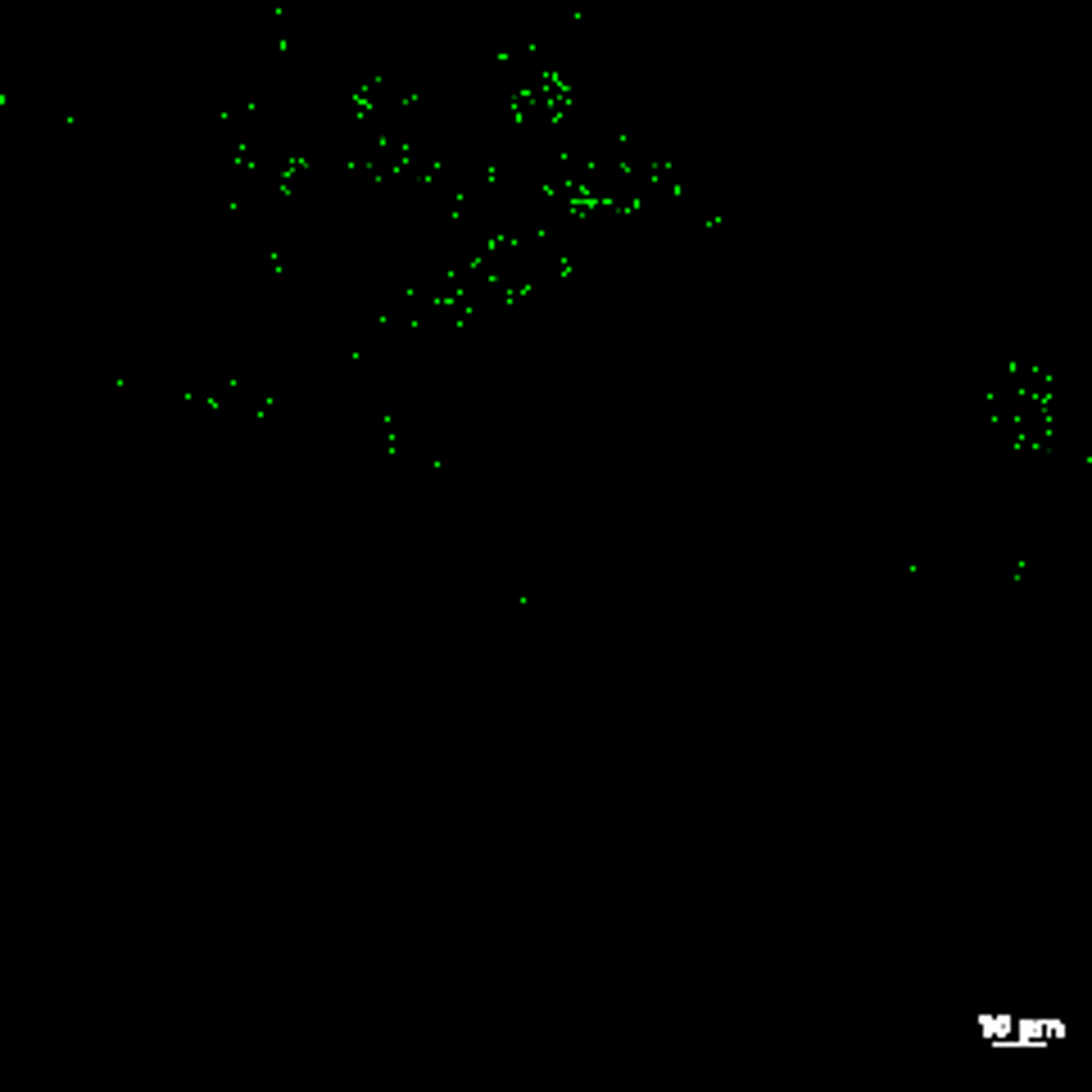

Supplement: Supplementary file 12 — Figure EV1 Source Data [file 44318_2026_783_MOESM12_ESM.zip › Figure EV1/Figure EV1A/0.5�FBS_UT_TOP1cc.tif]

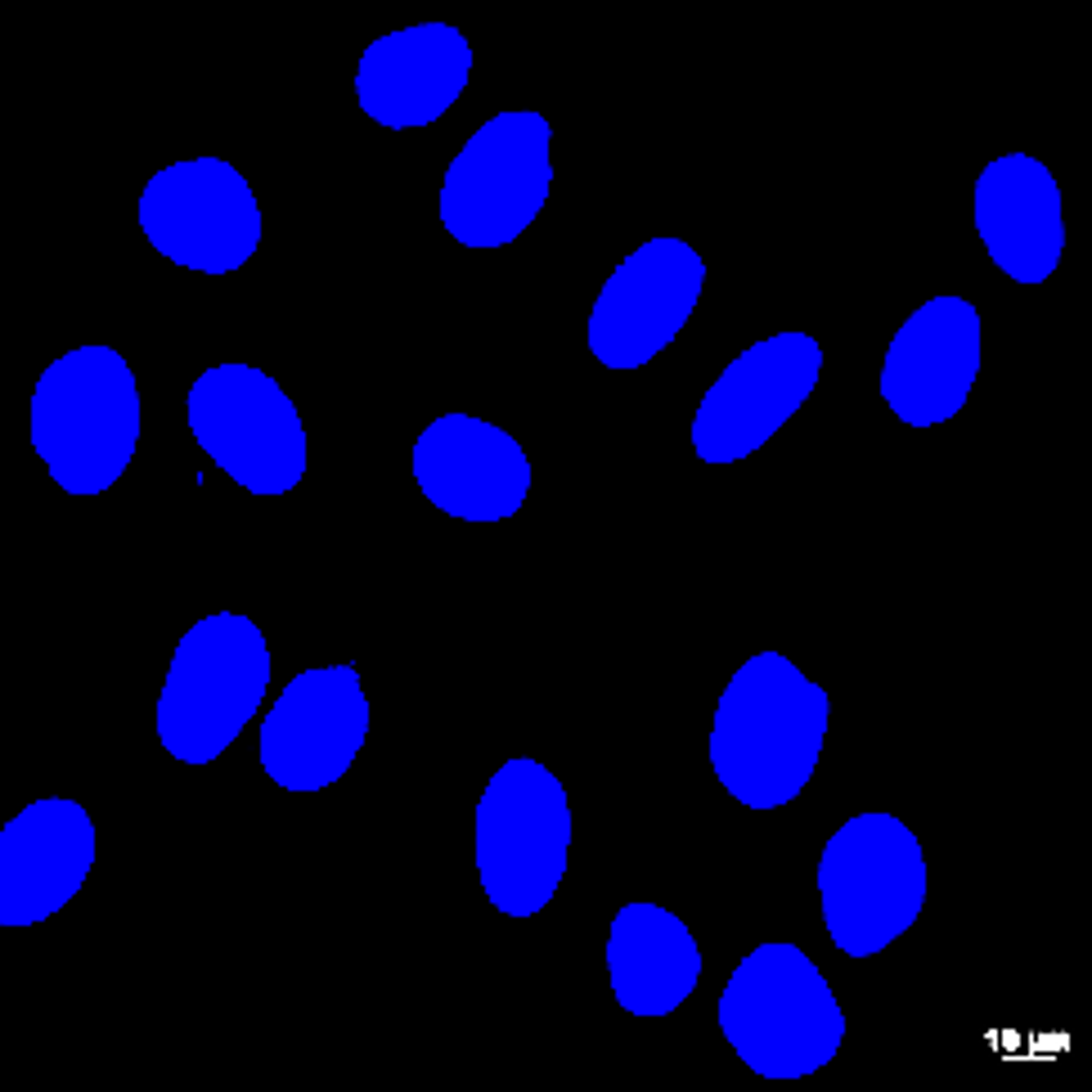

Supplement: Supplementary file 12 — Figure EV1 Source Data [file 44318_2026_783_MOESM12_ESM.zip › Figure EV1/Figure EV1A/APH_CHK1i_Hoechst.tif]

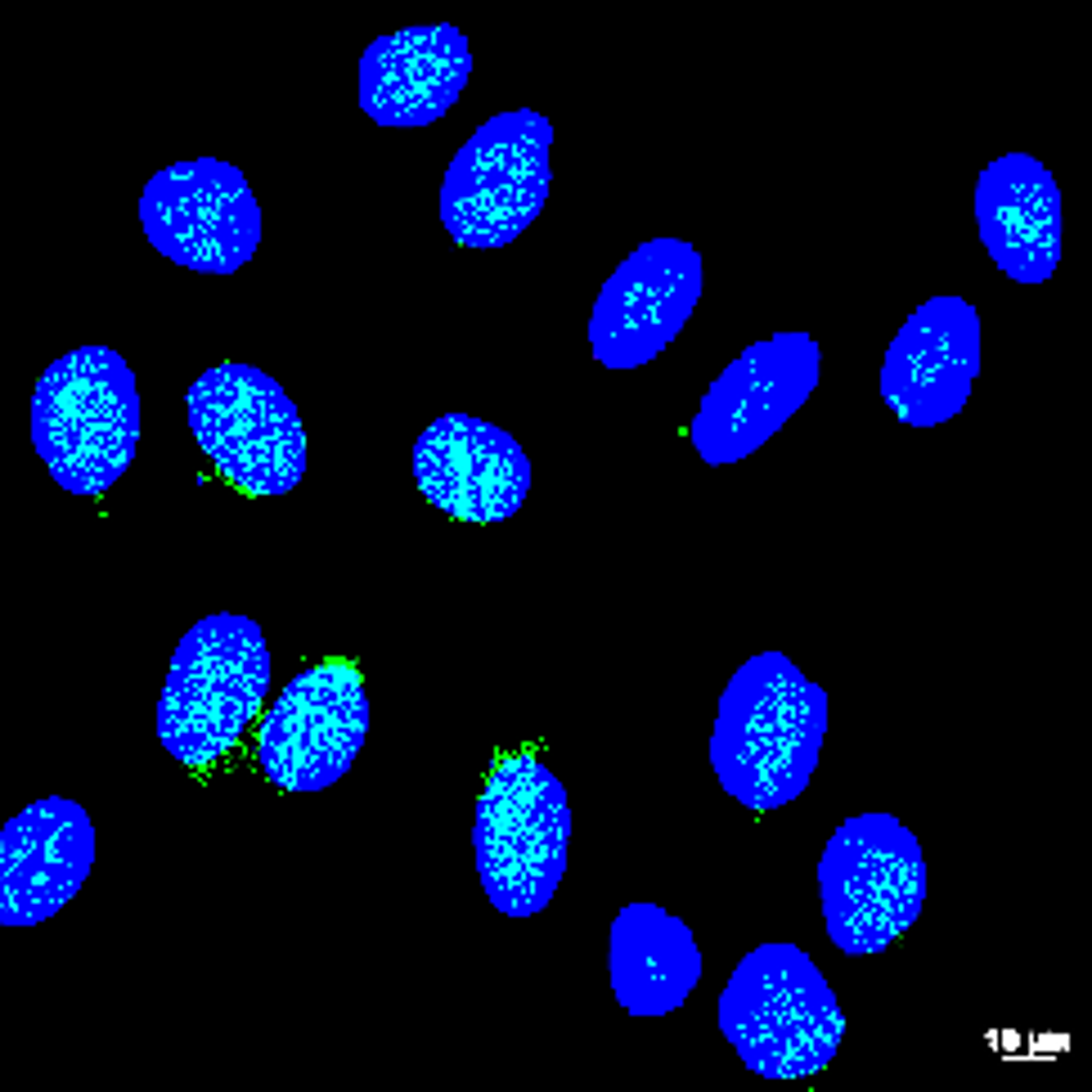

Supplement: Supplementary file 12 — Figure EV1 Source Data [file 44318_2026_783_MOESM12_ESM.zip › Figure EV1/Figure EV1A/APH_CHK1i_Merged.tif]

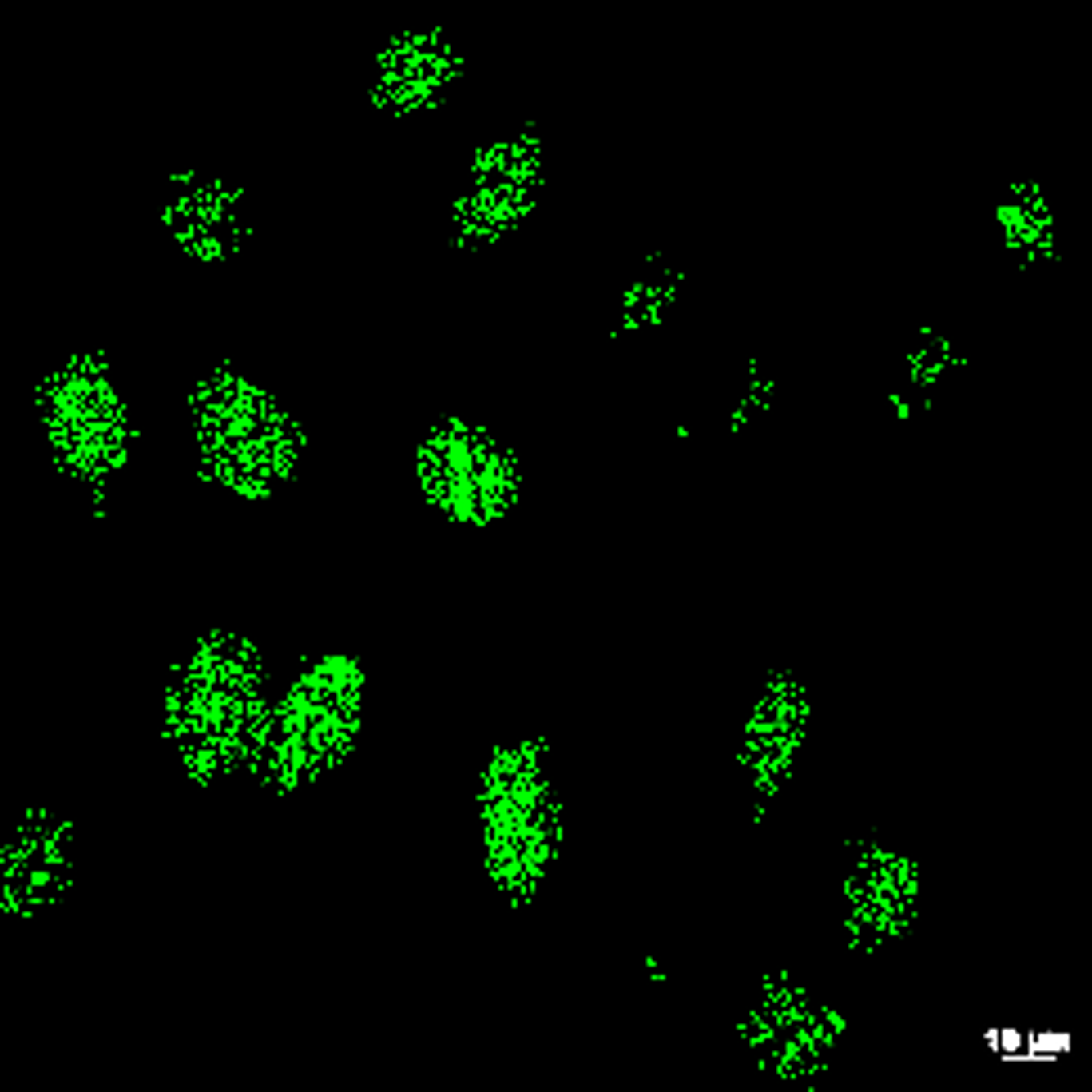

Supplement: Supplementary file 12 — Figure EV1 Source Data [file 44318_2026_783_MOESM12_ESM.zip › Figure EV1/Figure EV1A/APH_CHK1i_TOP1cc.tif]

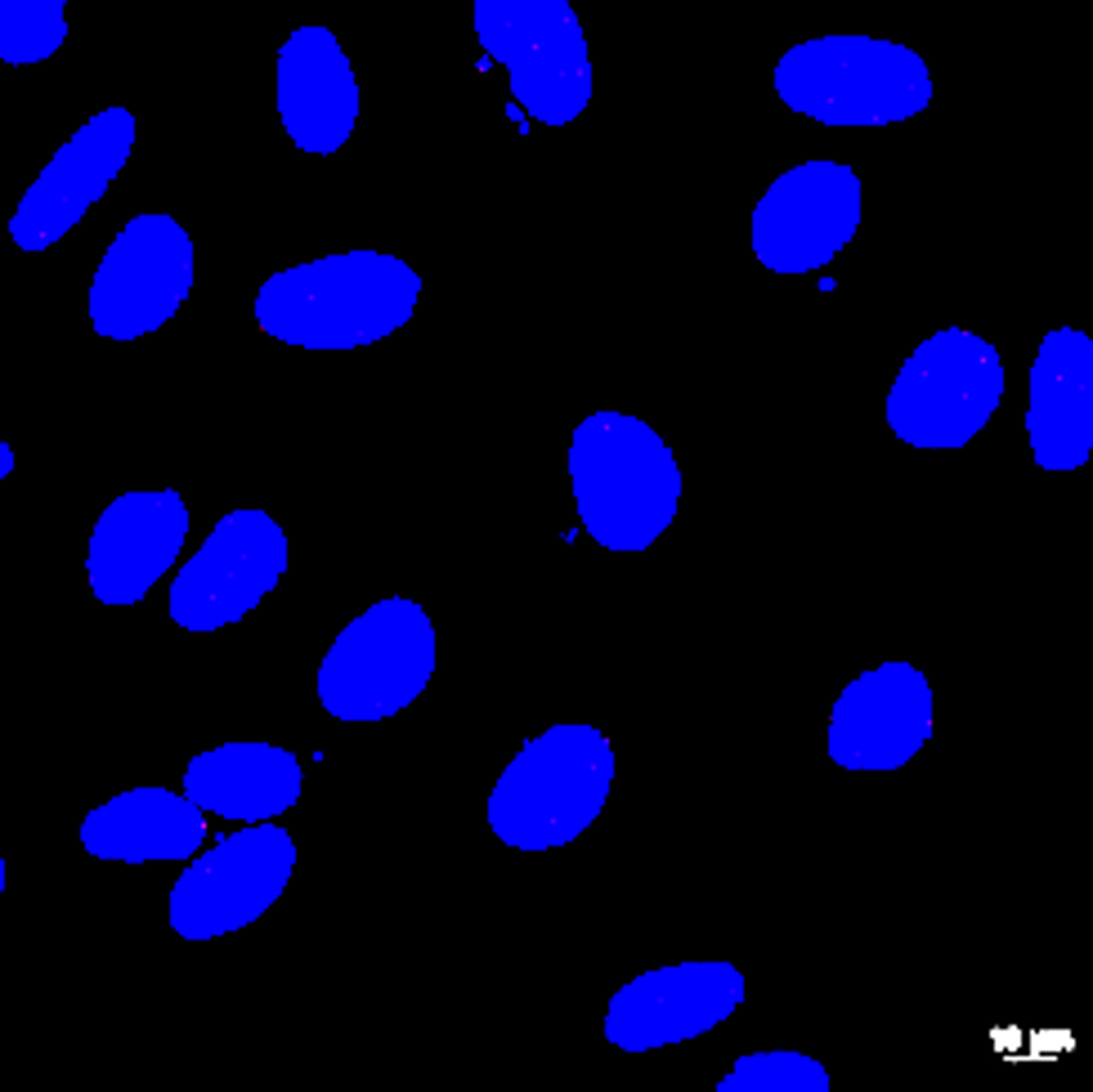

Supplement: Supplementary file 12 — Figure EV1 Source Data [file 44318_2026_783_MOESM12_ESM.zip › Figure EV1/Figure EV1A/APH_UT_Hoechst.tif]

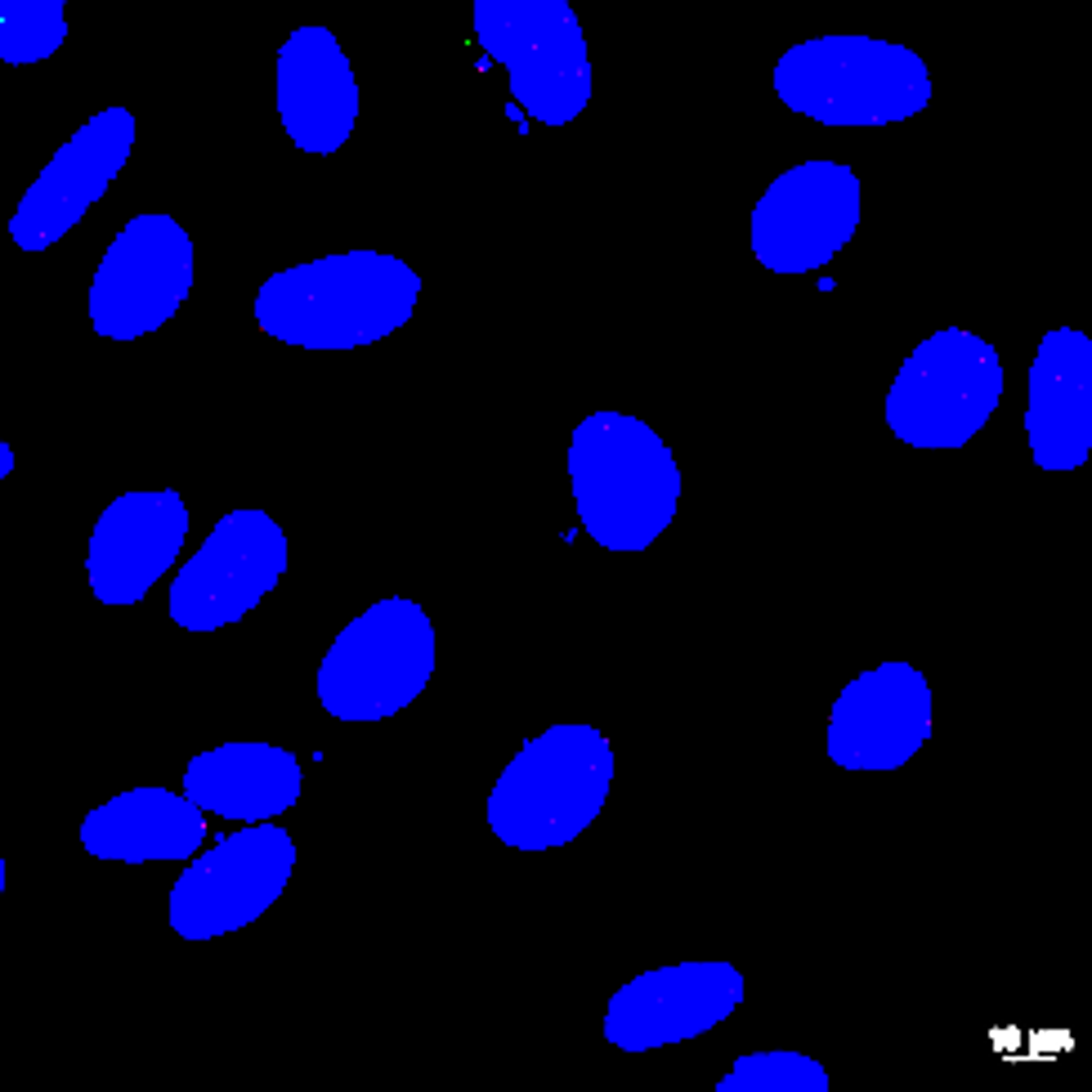

Supplement: Supplementary file 12 — Figure EV1 Source Data [file 44318_2026_783_MOESM12_ESM.zip › Figure EV1/Figure EV1A/APH_UT_Merged.tif]

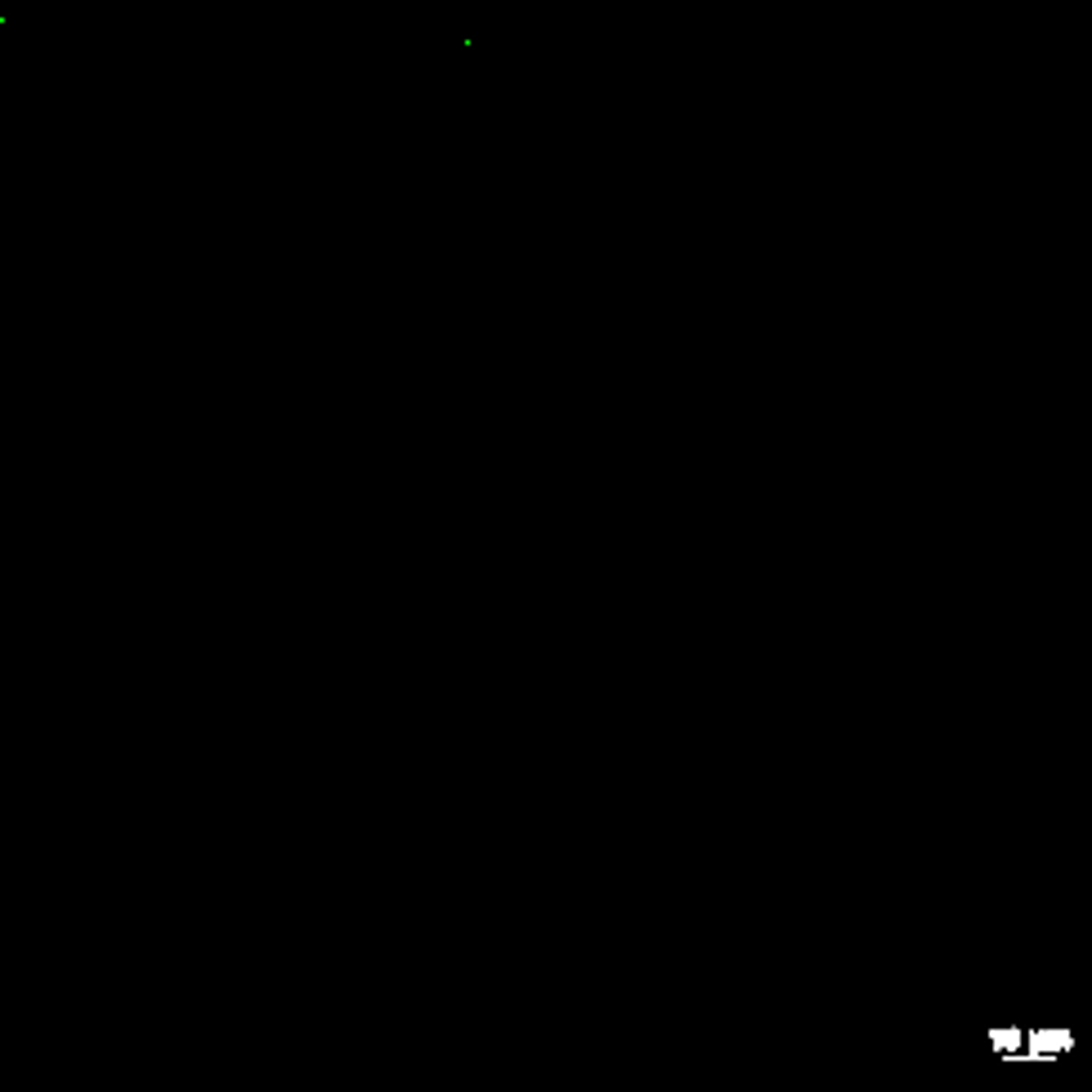

Supplement: Supplementary file 12 — Figure EV1 Source Data [file 44318_2026_783_MOESM12_ESM.zip › Figure EV1/Figure EV1A/APH_UT_TOP1cc.tif]

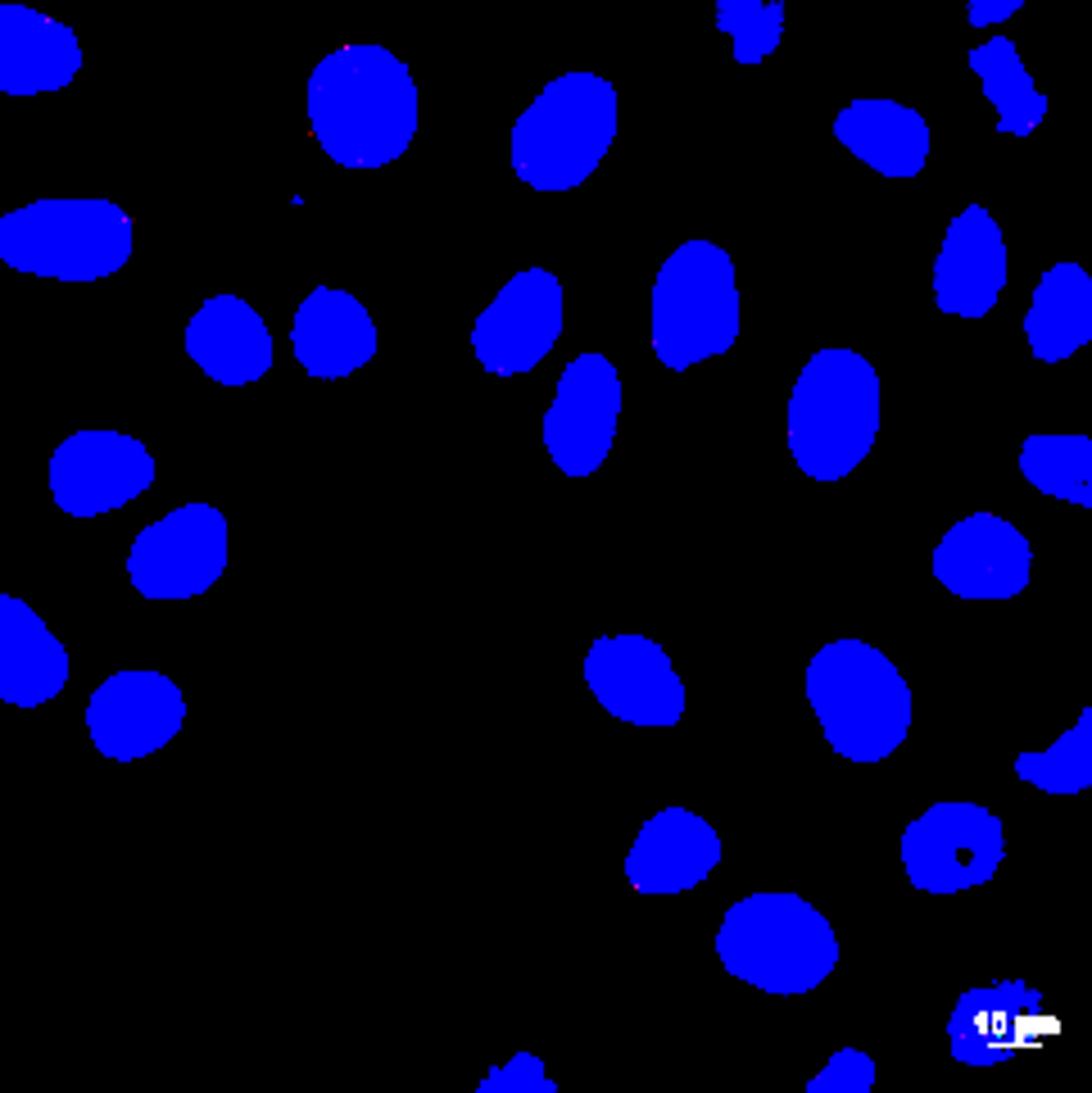

Supplement: Supplementary file 12 — Figure EV1 Source Data [file 44318_2026_783_MOESM12_ESM.zip › Figure EV1/Figure EV1A/CHK1i_Hoechst.tif]

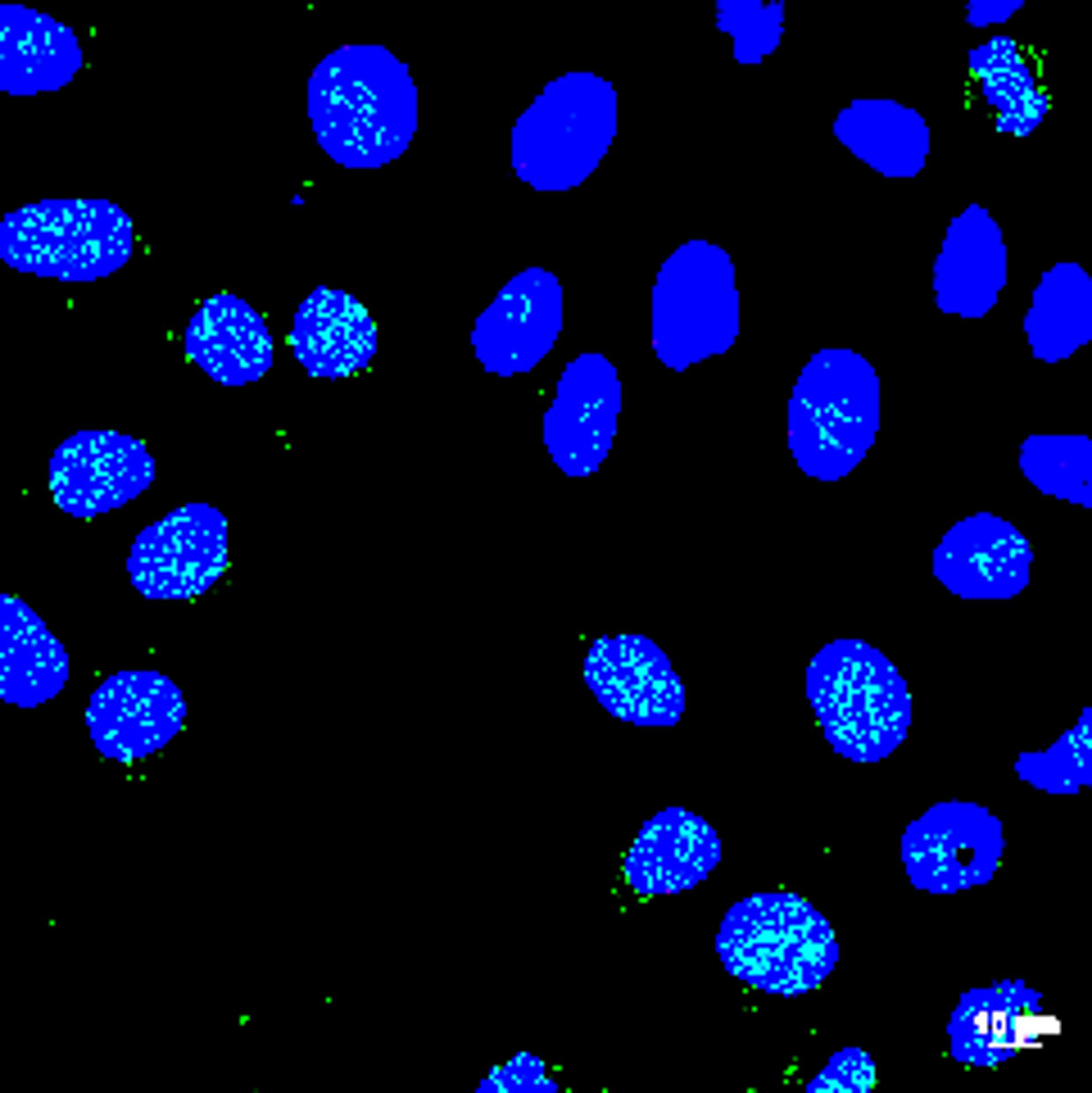

Supplement: Supplementary file 12 — Figure EV1 Source Data [file 44318_2026_783_MOESM12_ESM.zip › Figure EV1/Figure EV1A/CHK1i_Merged.tif]

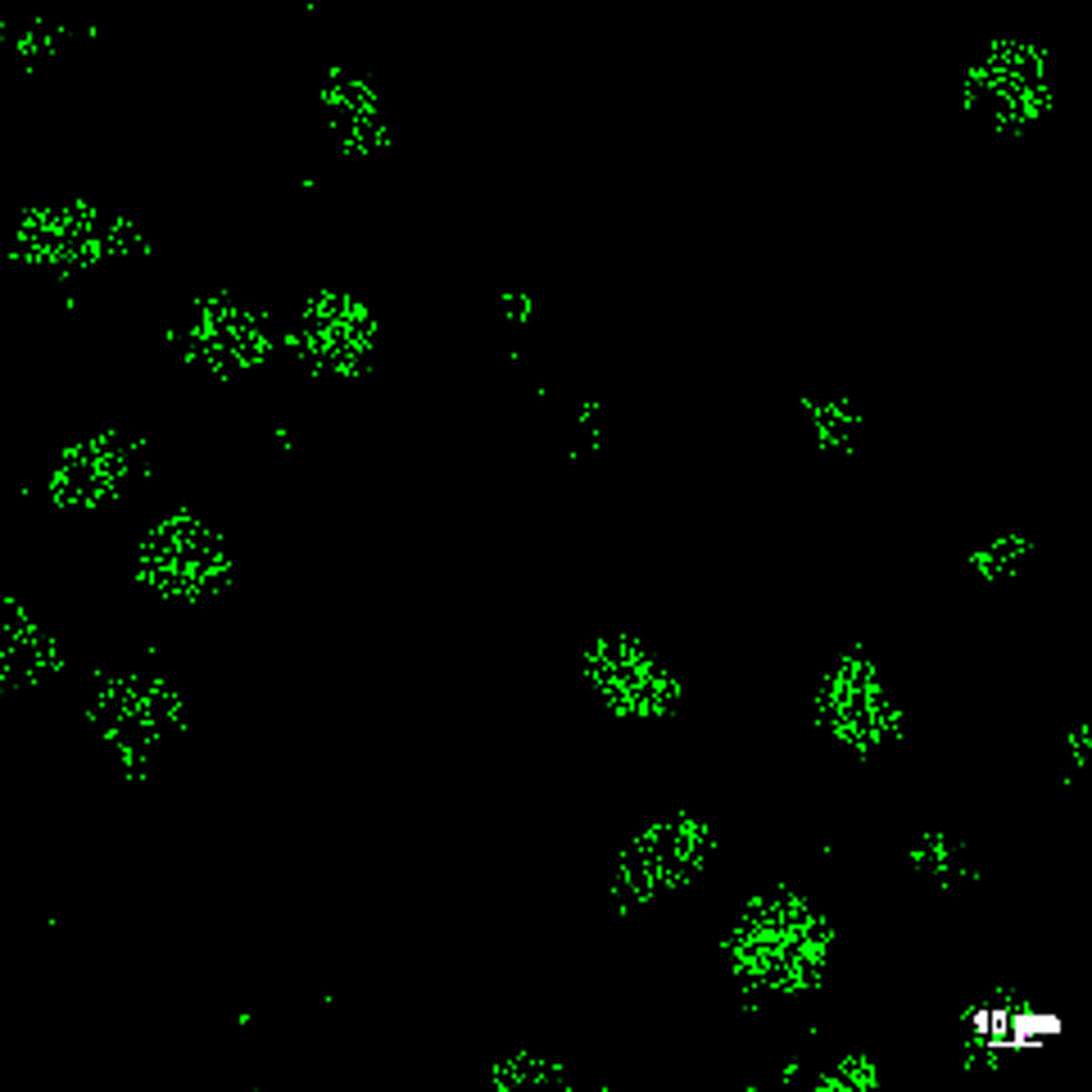

Supplement: Supplementary file 12 — Figure EV1 Source Data [file 44318_2026_783_MOESM12_ESM.zip › Figure EV1/Figure EV1A/CHK1i_TOP1cc.tif]

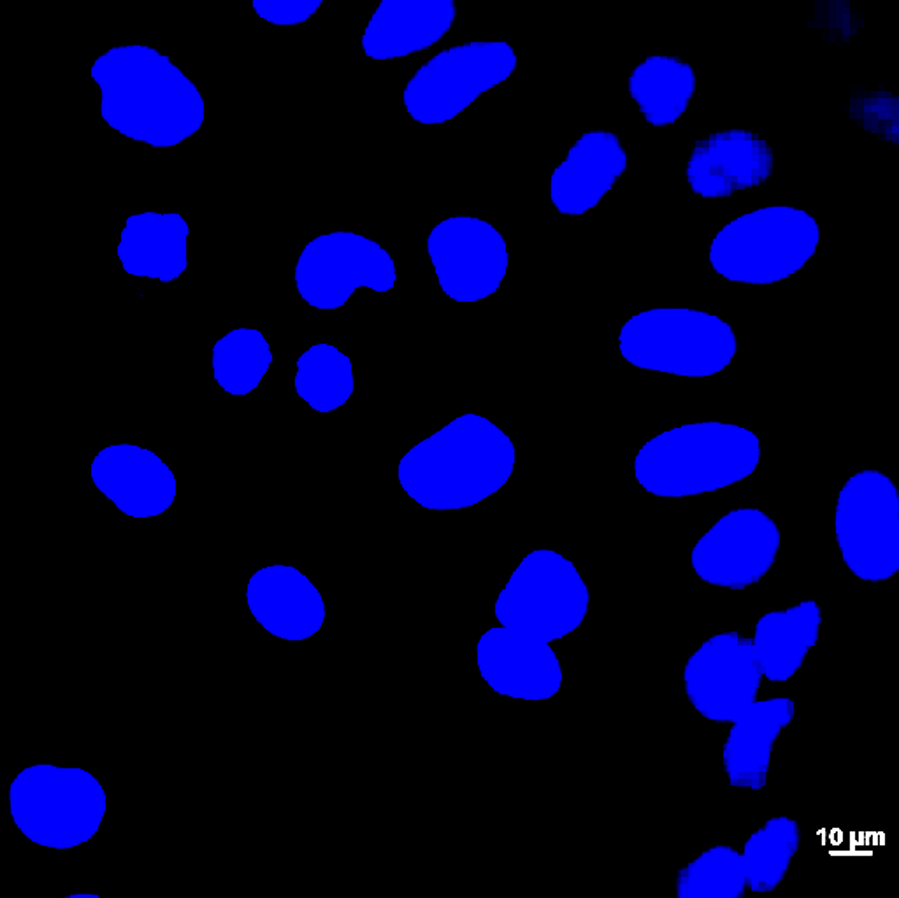

Supplement: Supplementary file 12 — Figure EV1 Source Data [file 44318_2026_783_MOESM12_ESM.zip › Figure EV1/Figure EV1A/PHA_CHK1i_Hoechst.tif]

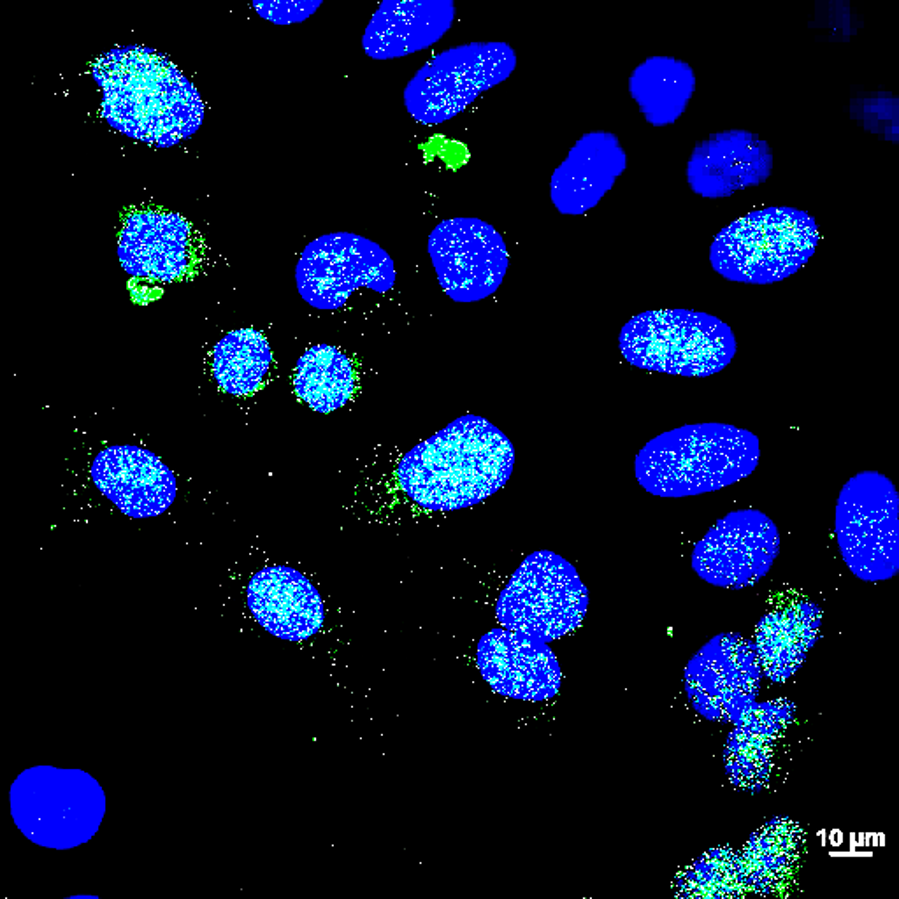

Supplement: Supplementary file 12 — Figure EV1 Source Data [file 44318_2026_783_MOESM12_ESM.zip › Figure EV1/Figure EV1A/PHA_CHK1i_Merged.tif]

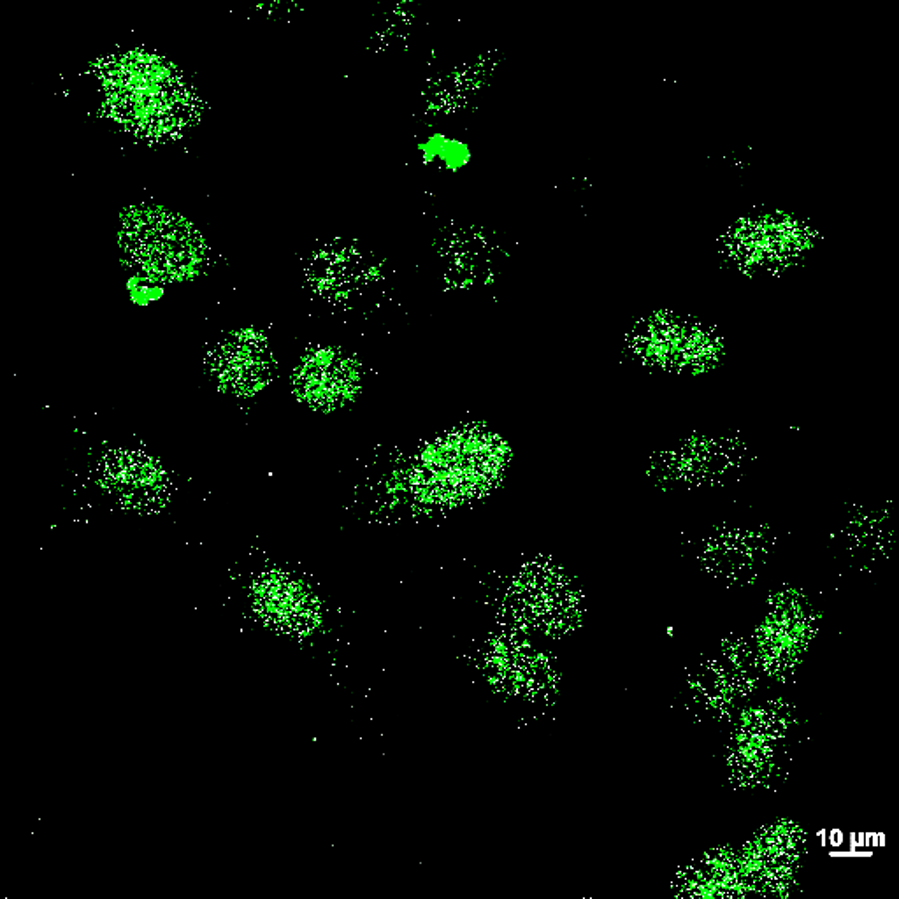

Supplement: Supplementary file 12 — Figure EV1 Source Data [file 44318_2026_783_MOESM12_ESM.zip › Figure EV1/Figure EV1A/PHA_CHK1i_TOP1cc.tif]

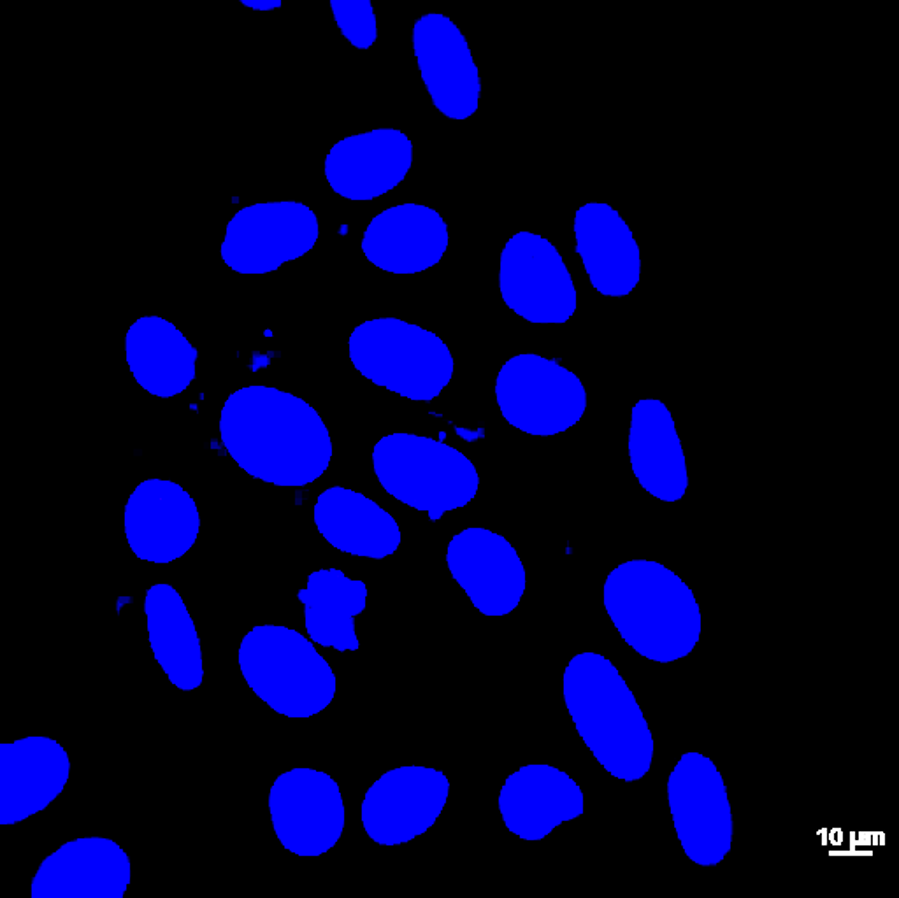

Supplement: Supplementary file 12 — Figure EV1 Source Data [file 44318_2026_783_MOESM12_ESM.zip › Figure EV1/Figure EV1A/PHA_UT_Hoechst.tif]

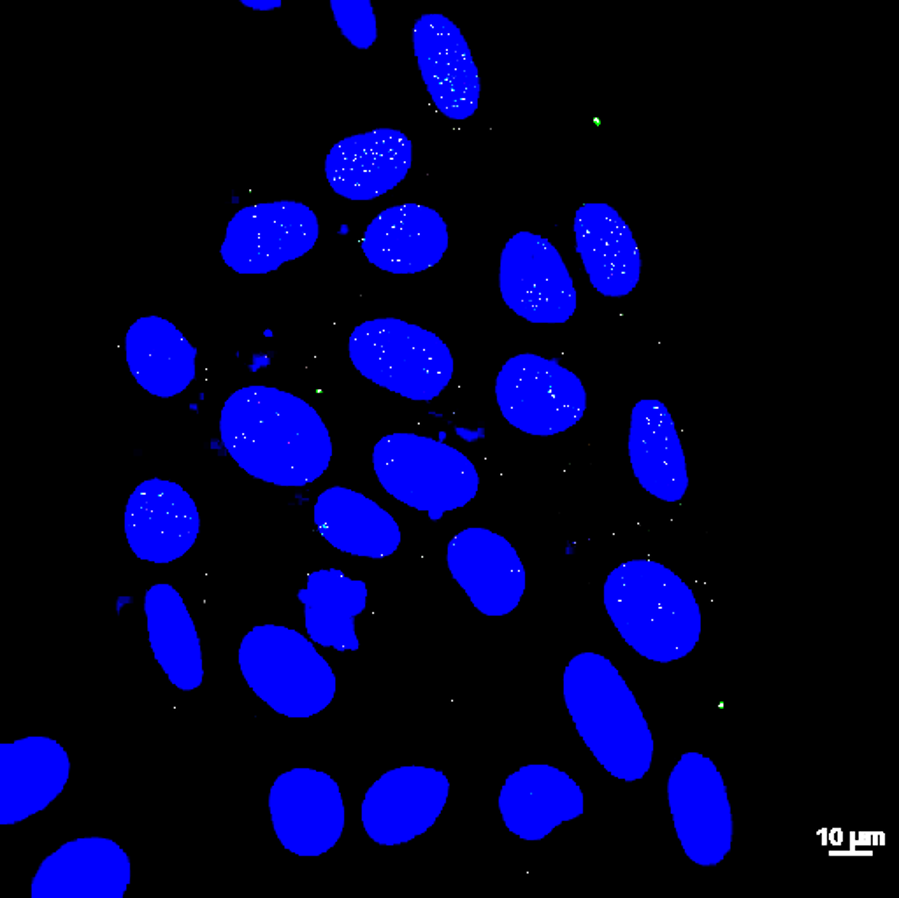

Supplement: Supplementary file 12 — Figure EV1 Source Data [file 44318_2026_783_MOESM12_ESM.zip › Figure EV1/Figure EV1A/PHA_UT_Merged.tif]

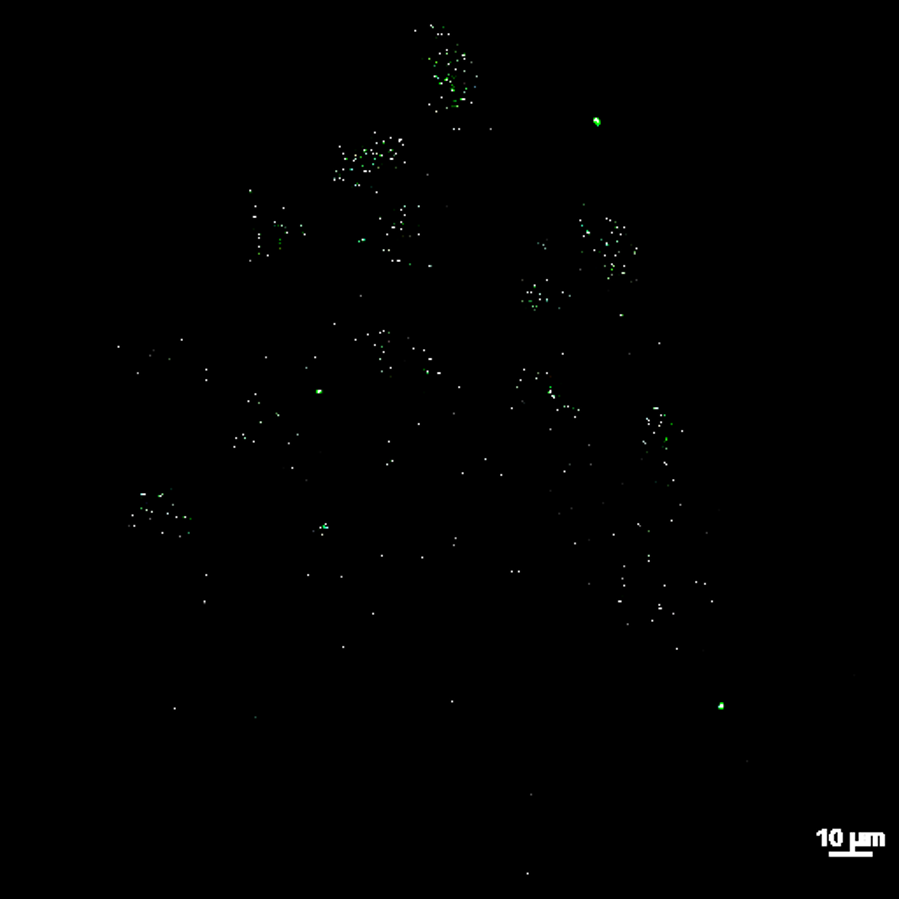

Supplement: Supplementary file 12 — Figure EV1 Source Data [file 44318_2026_783_MOESM12_ESM.zip › Figure EV1/Figure EV1A/PHA_UT_TOP1cc.tif]

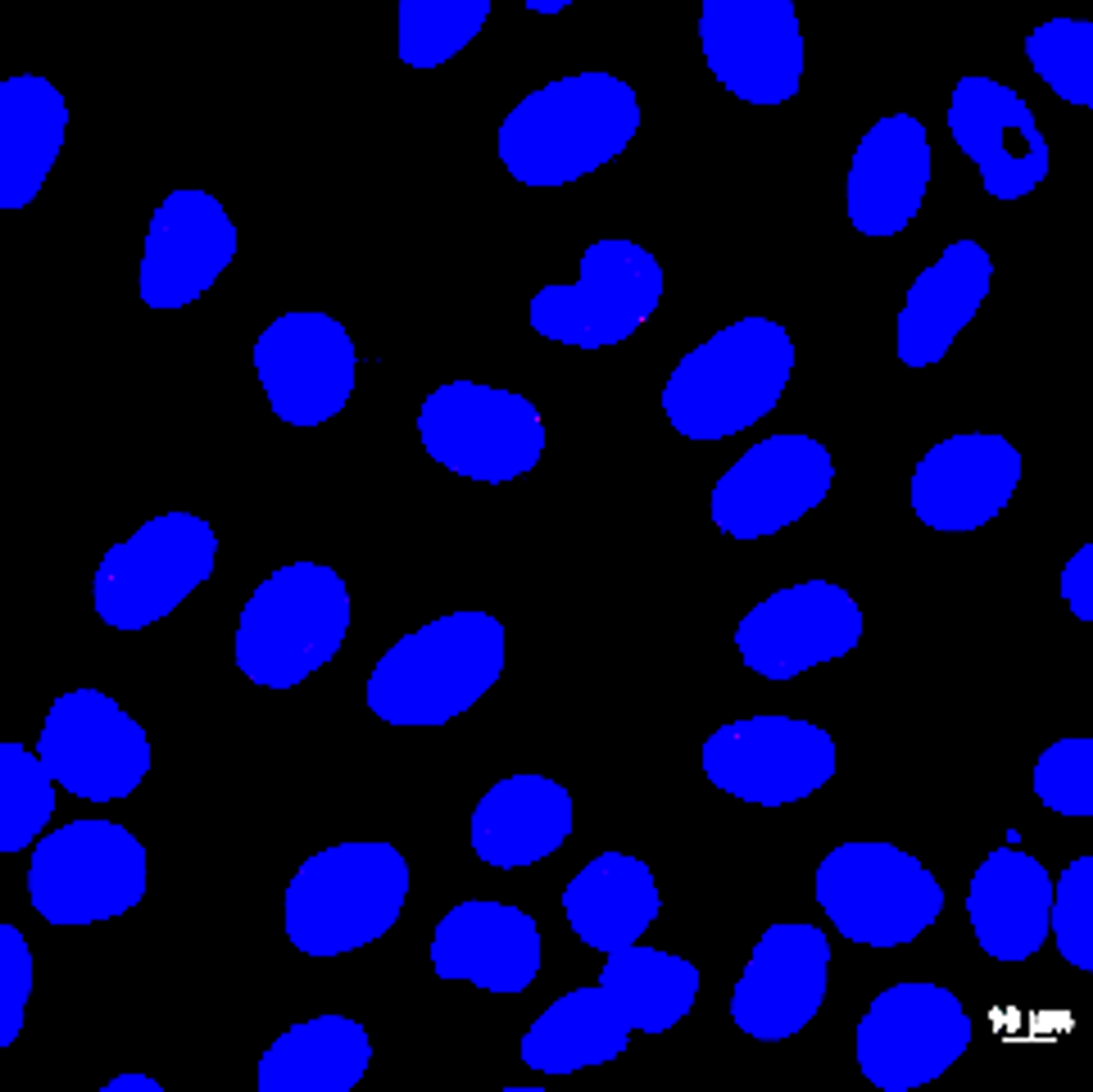

Supplement: Supplementary file 12 — Figure EV1 Source Data [file 44318_2026_783_MOESM12_ESM.zip › Figure EV1/Figure EV1A/UT_Hoechst.tif]

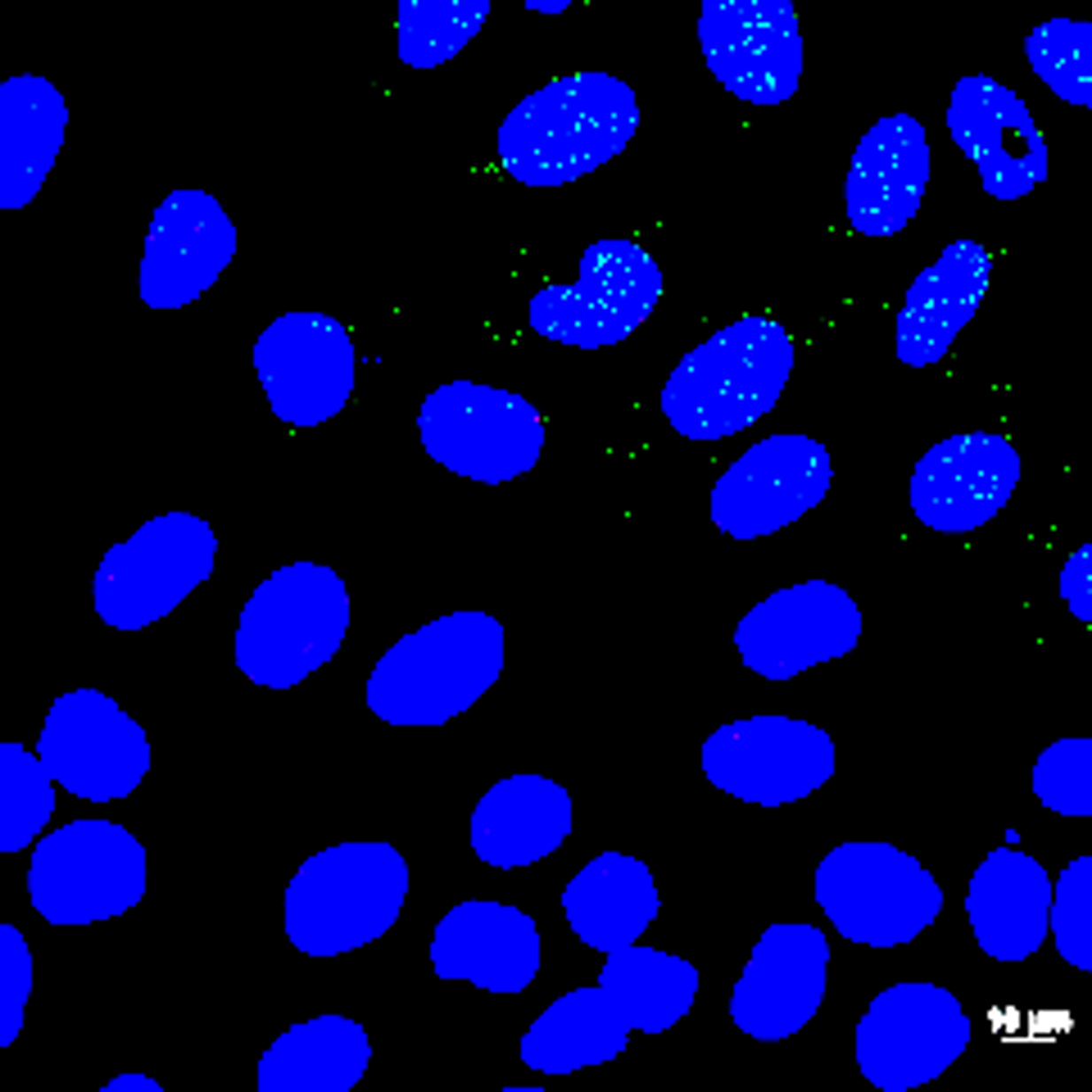

Supplement: Supplementary file 12 — Figure EV1 Source Data [file 44318_2026_783_MOESM12_ESM.zip › Figure EV1/Figure EV1A/UT_Merged.tif]

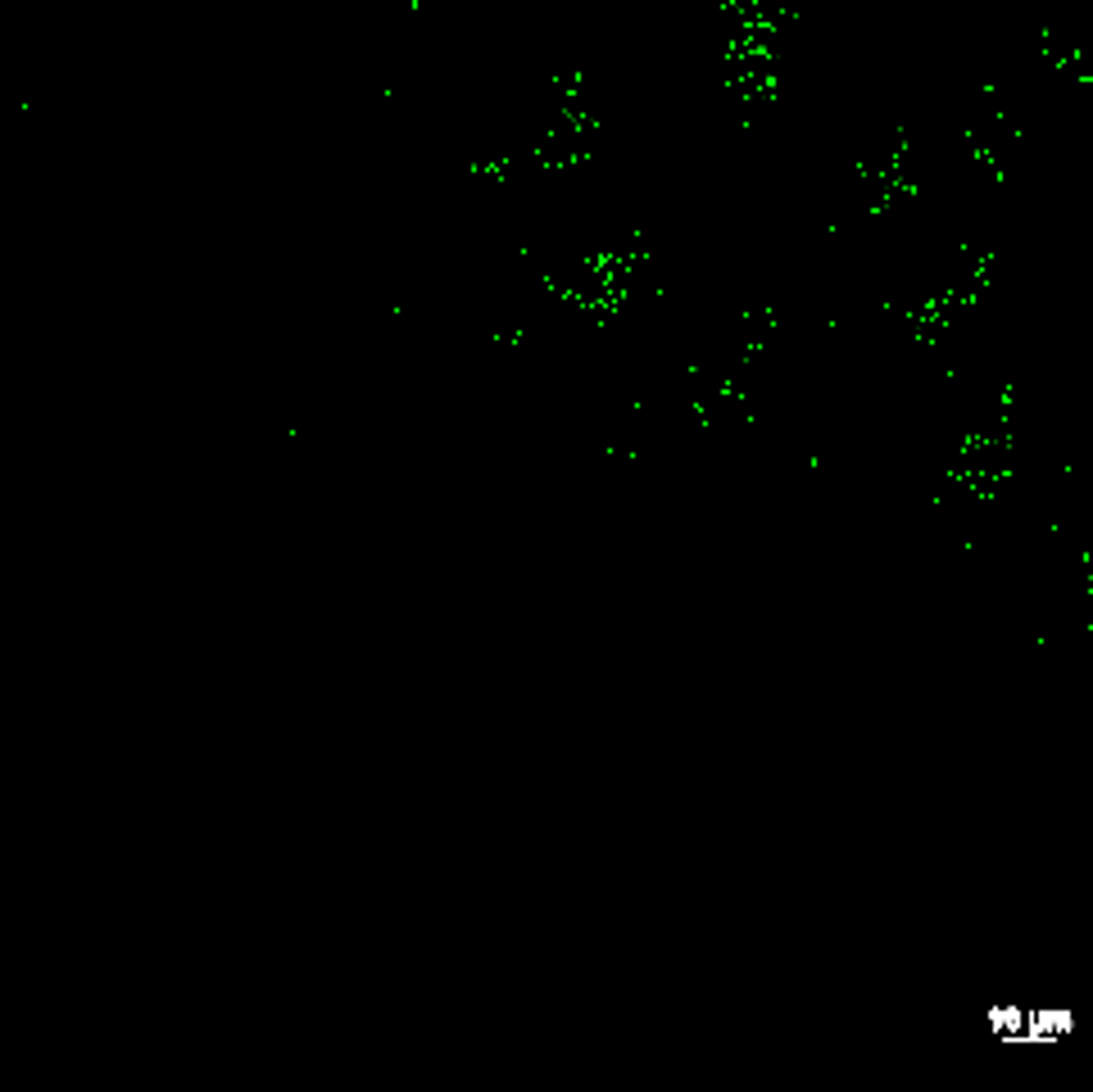

Supplement: Supplementary file 12 — Figure EV1 Source Data [file 44318_2026_783_MOESM12_ESM.zip › Figure EV1/Figure EV1A/UT_TOP1cc.tif]

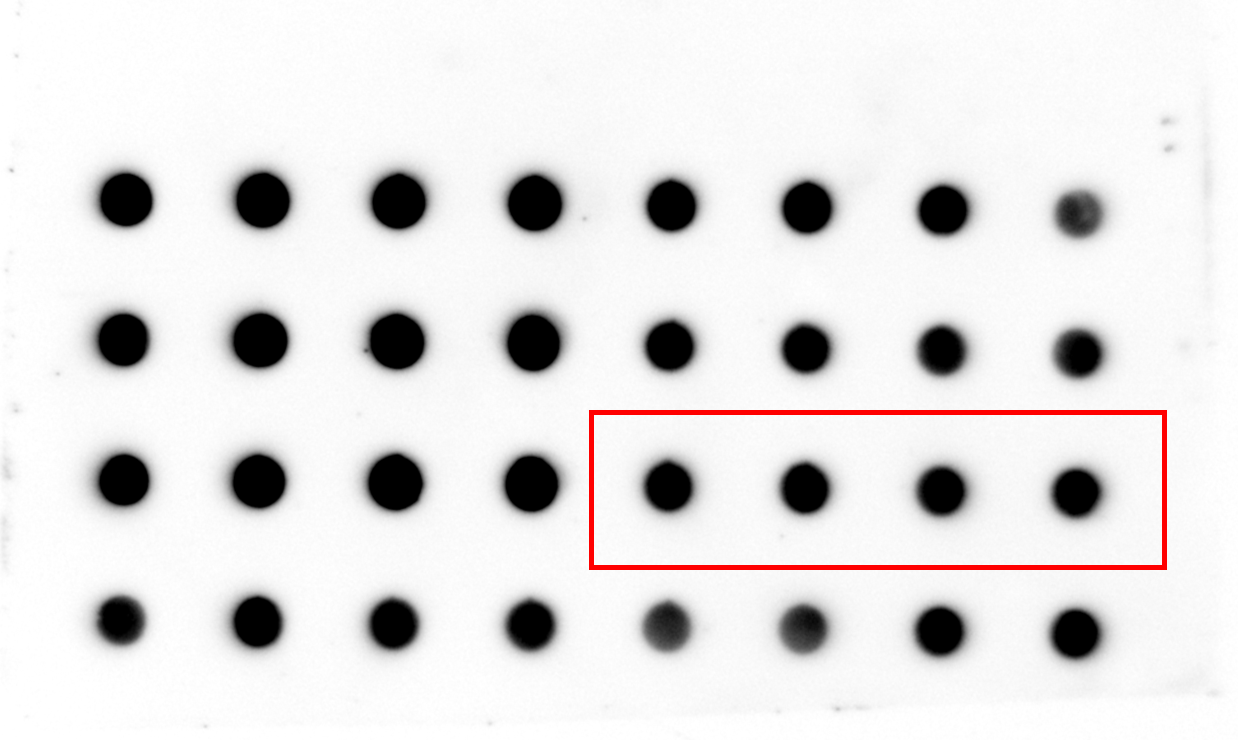

Supplement: Supplementary file 13 — Figure EV2 Source Data [file 44318_2026_783_MOESM13_ESM.zip › Figure EV2/Figure EV2A/S320A_DNA.tif]

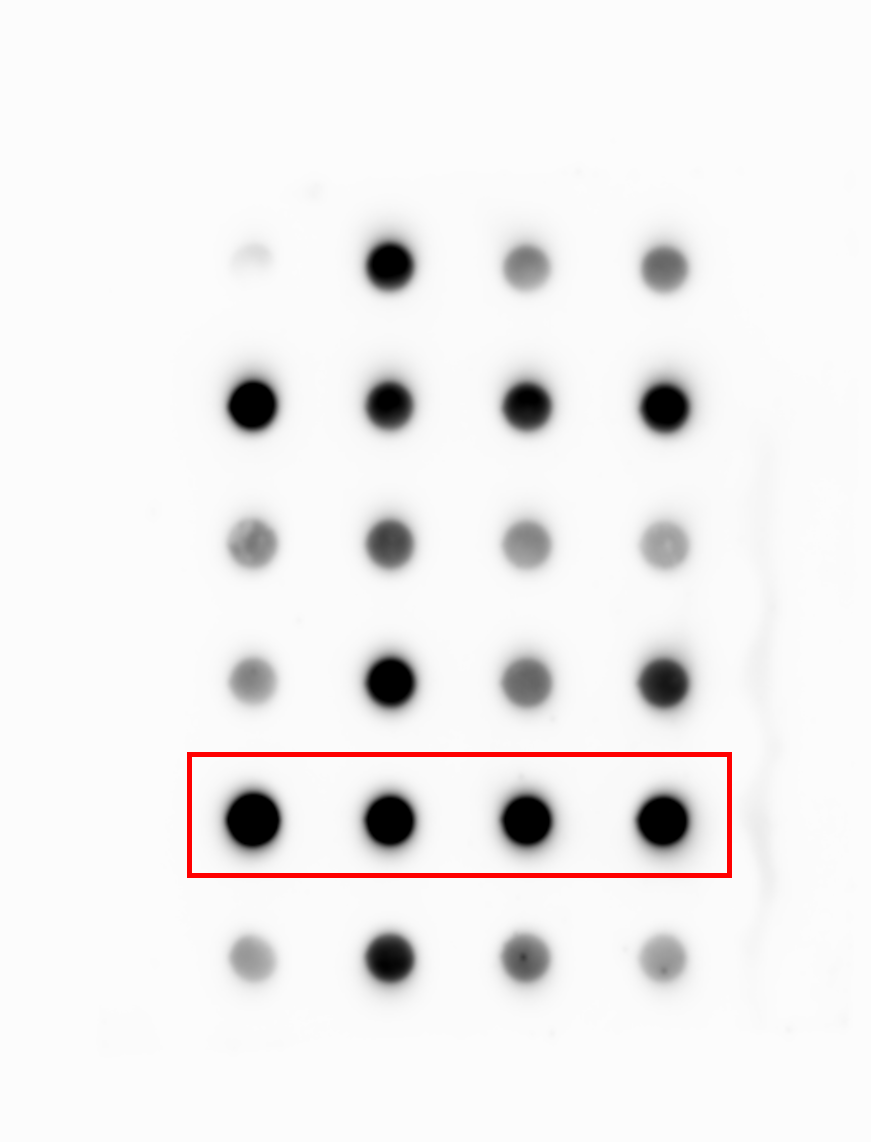

Supplement: Supplementary file 13 — Figure EV2 Source Data [file 44318_2026_783_MOESM13_ESM.zip › Figure EV2/Figure EV2A/S320A_GFP.tif]

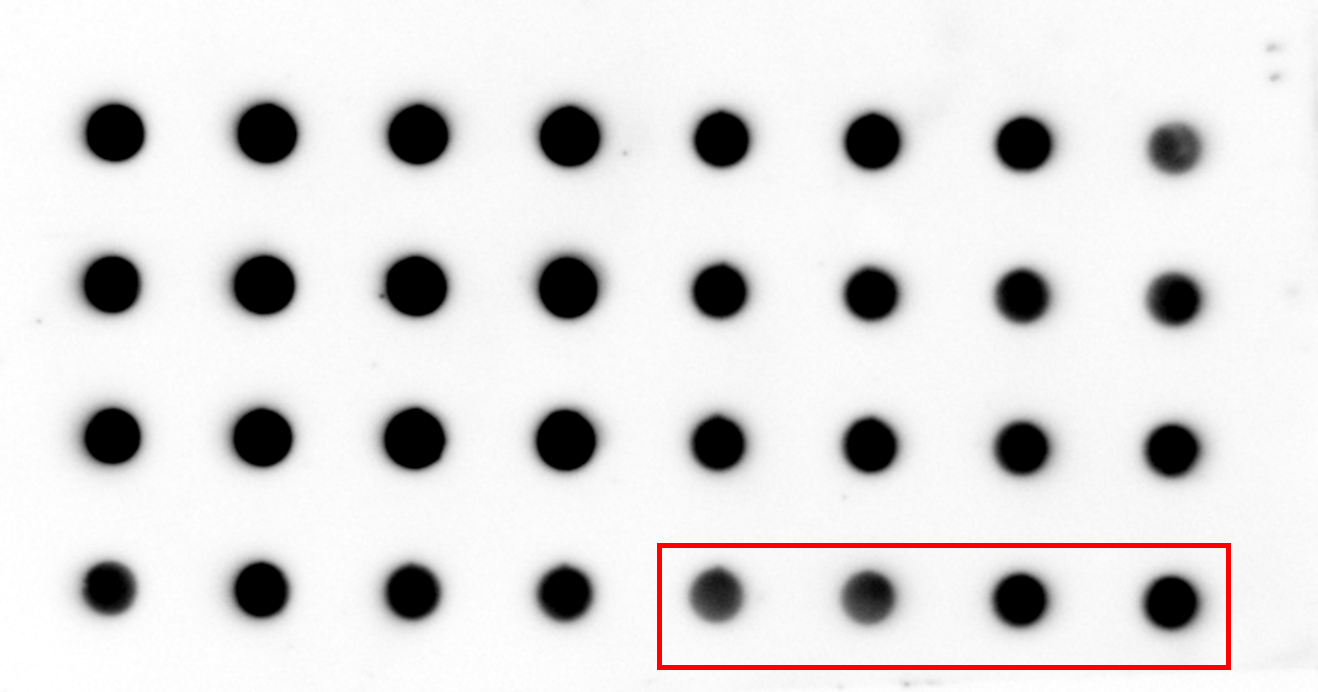

Supplement: Supplementary file 13 — Figure EV2 Source Data [file 44318_2026_783_MOESM13_ESM.zip › Figure EV2/Figure EV2A/S394A_DNA.tif]

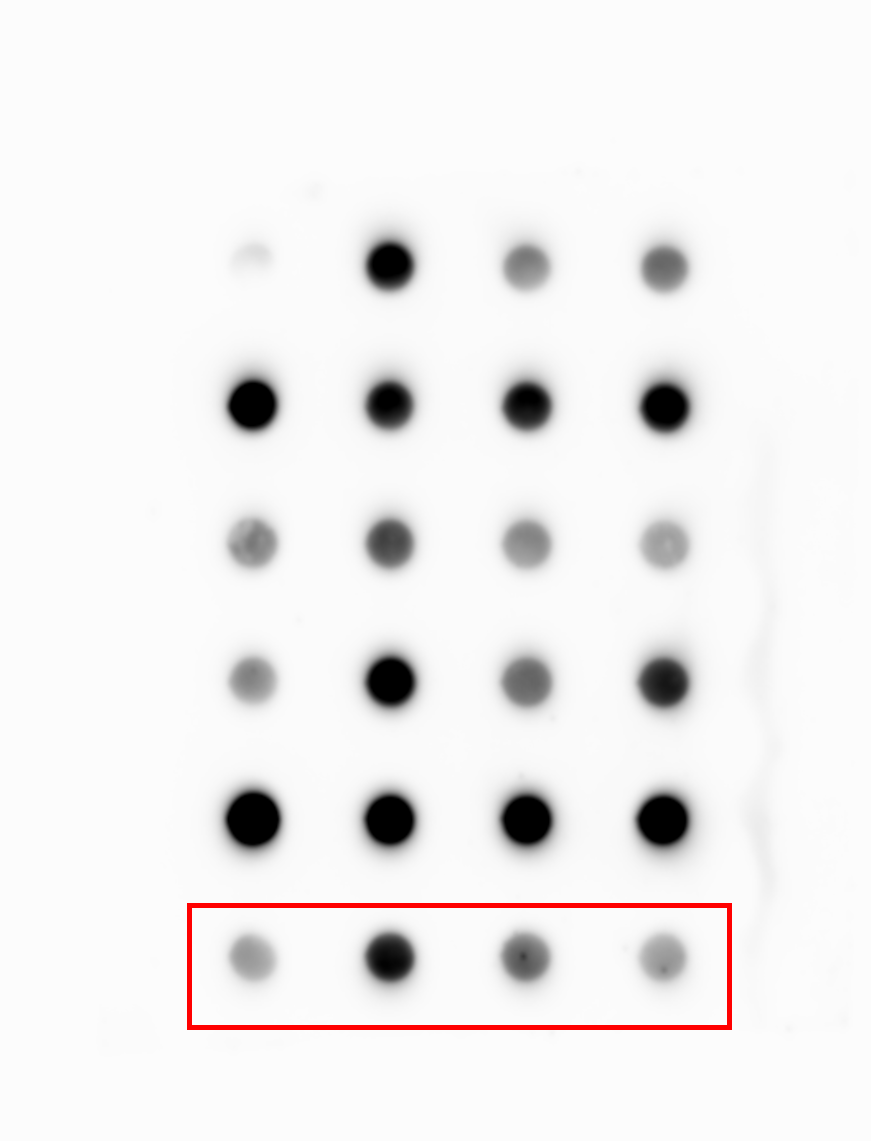

Supplement: Supplementary file 13 — Figure EV2 Source Data [file 44318_2026_783_MOESM13_ESM.zip › Figure EV2/Figure EV2A/S394A_GFP.tif]

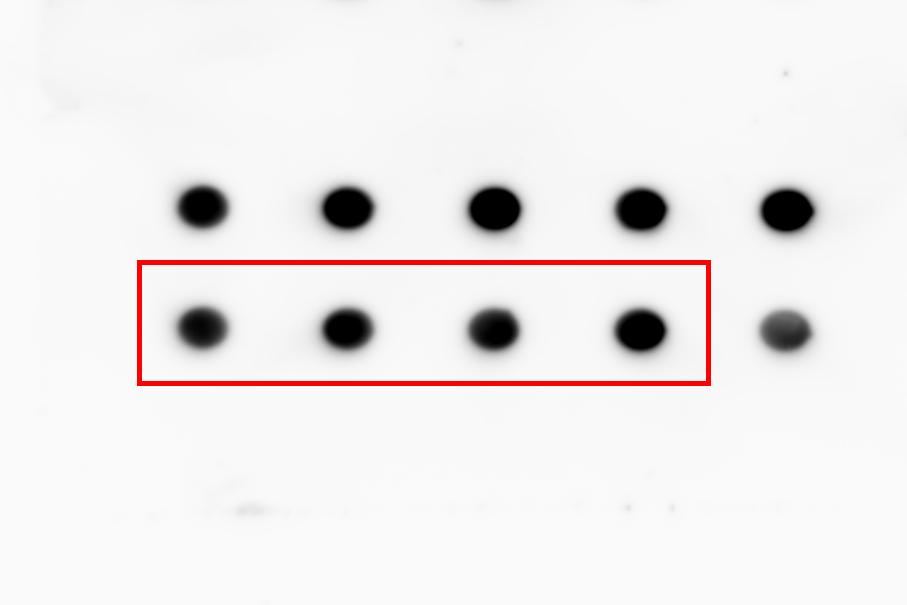

Supplement: Supplementary file 13 — Figure EV2 Source Data [file 44318_2026_783_MOESM13_ESM.zip › Figure EV2/Figure EV2A/T570A_DNA.tif]

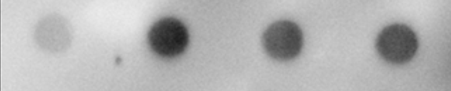

Supplement: Supplementary file 13 — Figure EV2 Source Data [file 44318_2026_783_MOESM13_ESM.zip › Figure EV2/Figure EV2A/T570A_GFP.tif]

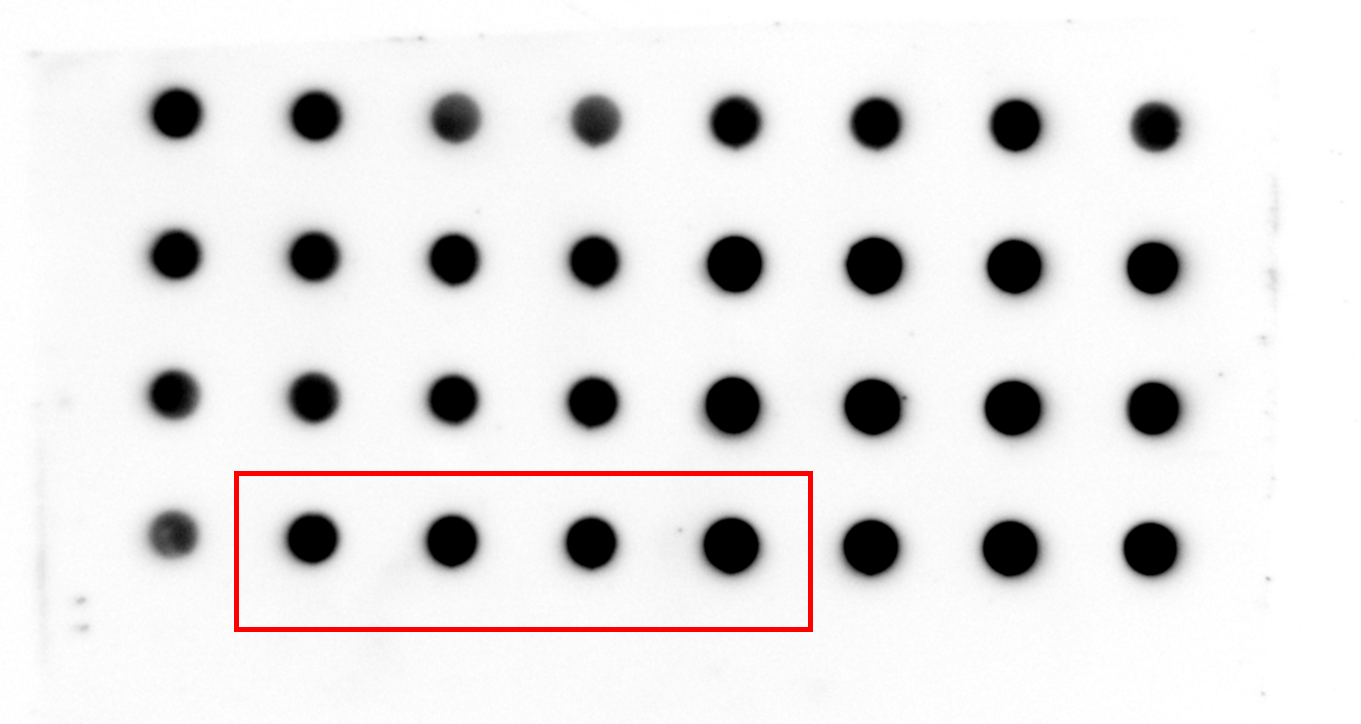

Supplement: Supplementary file 13 — Figure EV2 Source Data [file 44318_2026_783_MOESM13_ESM.zip › Figure EV2/Figure EV2A/WT_DNA.tif]

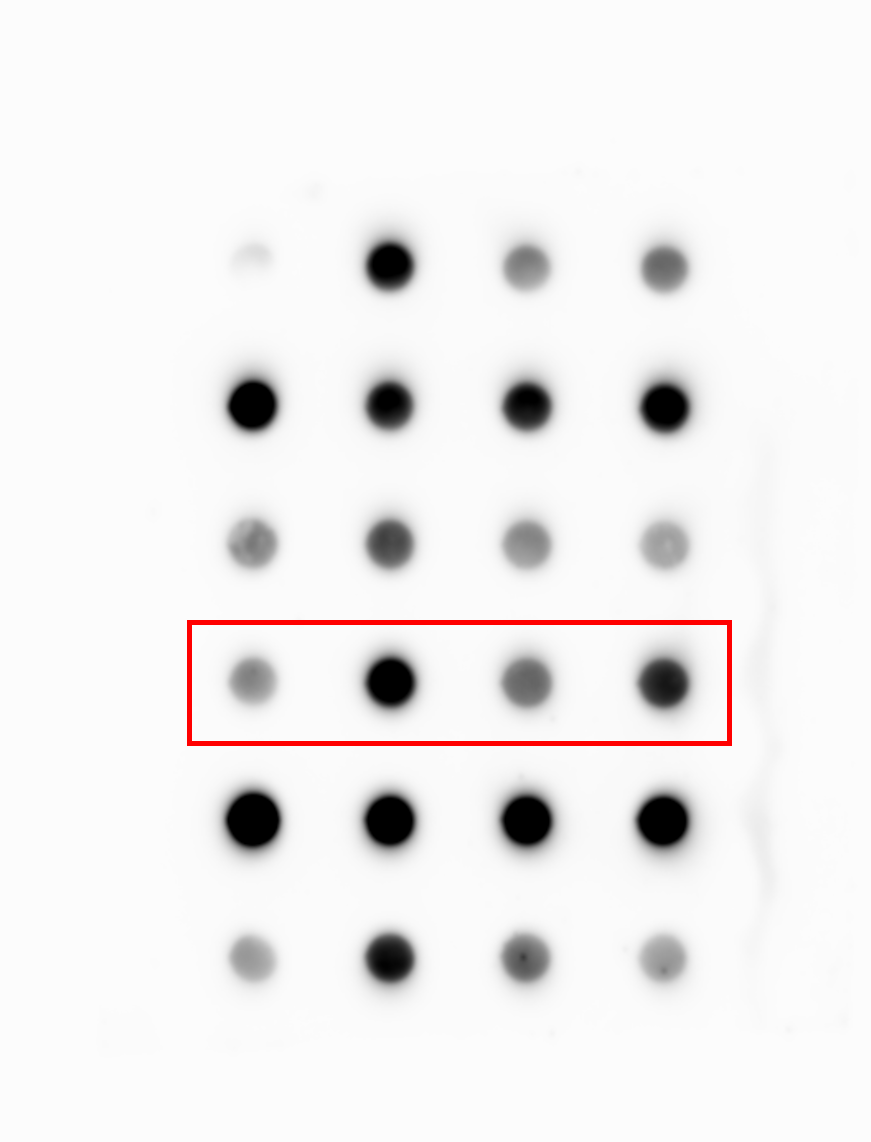

Supplement: Supplementary file 13 — Figure EV2 Source Data [file 44318_2026_783_MOESM13_ESM.zip › Figure EV2/Figure EV2A/WT_GFP.tif]

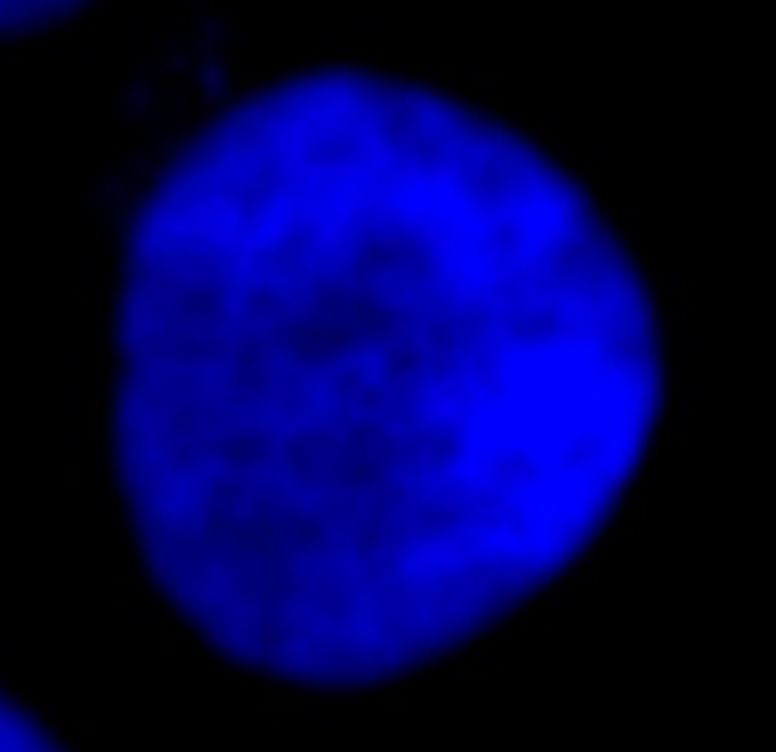

Supplement: Supplementary file 13 — Figure EV2 Source Data [file 44318_2026_783_MOESM13_ESM.zip › Figure EV2/Figure EV2C/Antibody Control_GFP_Hoechst.tif]

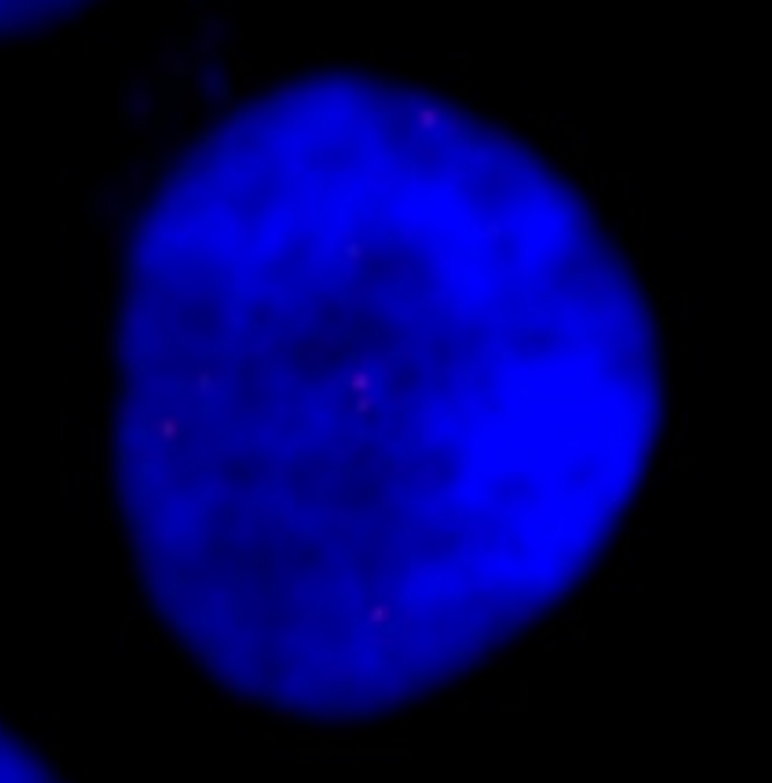

Supplement: Supplementary file 13 — Figure EV2 Source Data [file 44318_2026_783_MOESM13_ESM.zip › Figure EV2/Figure EV2C/Antibody Control_GFP_Merged.tif]

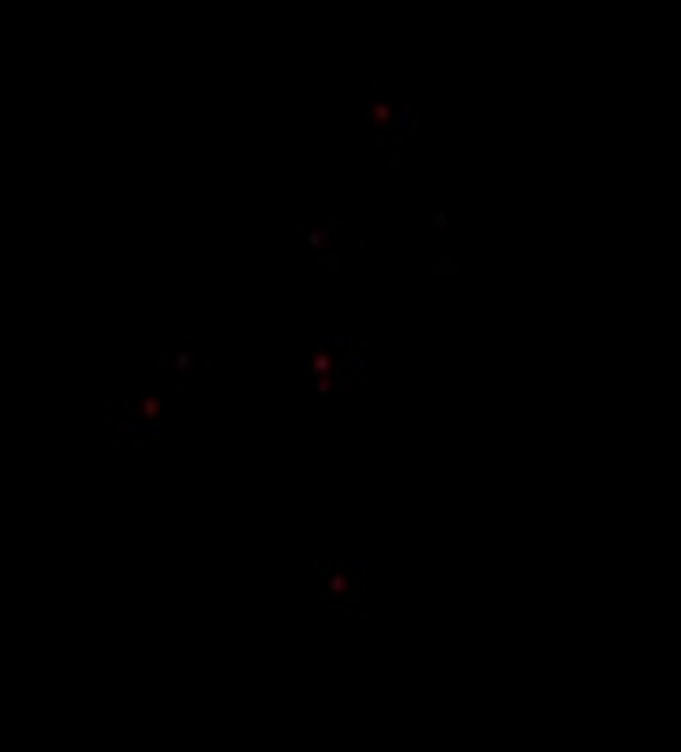

Supplement: Supplementary file 13 — Figure EV2 Source Data [file 44318_2026_783_MOESM13_ESM.zip › Figure EV2/Figure EV2C/Antibody Control_GFP_PLA.tif]

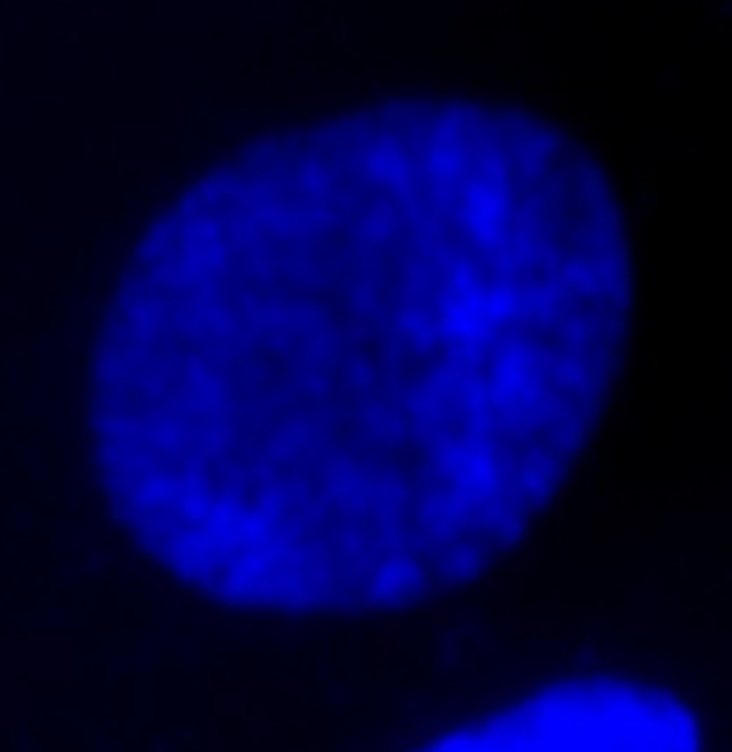

Supplement: Supplementary file 13 — Figure EV2 Source Data [file 44318_2026_783_MOESM13_ESM.zip › Figure EV2/Figure EV2C/Antibody Control_TOP1cc_Hoechst.tif]

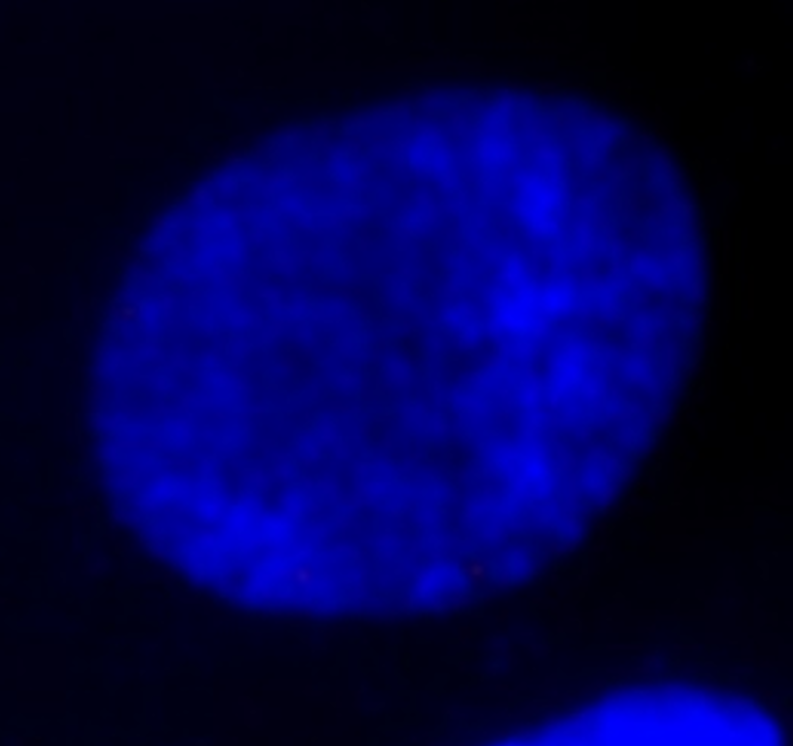

Supplement: Supplementary file 13 — Figure EV2 Source Data [file 44318_2026_783_MOESM13_ESM.zip › Figure EV2/Figure EV2C/Antibody Control_TOP1cc_Merged.tif]

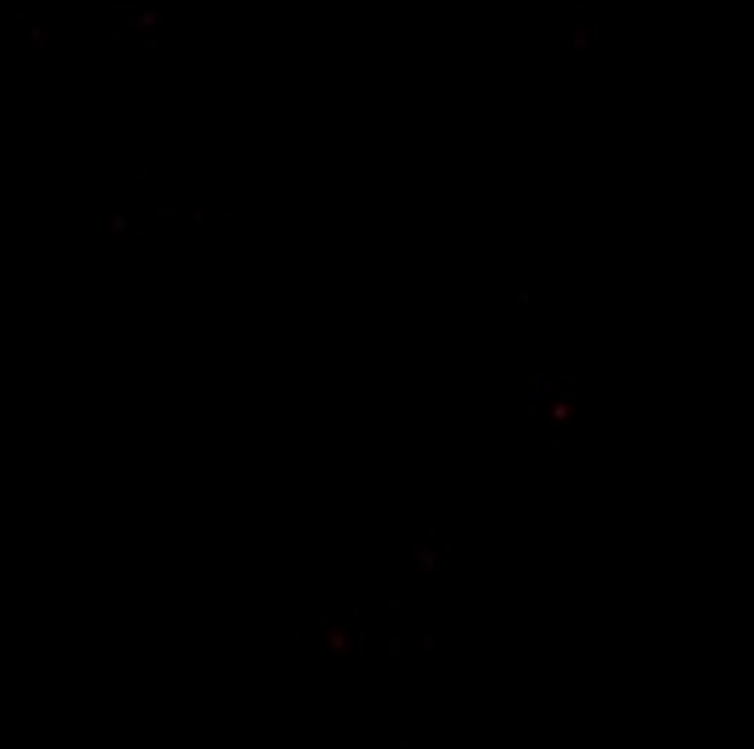

Supplement: Supplementary file 13 — Figure EV2 Source Data [file 44318_2026_783_MOESM13_ESM.zip › Figure EV2/Figure EV2C/Antibody Control_TOP1cc_PLA.tif]

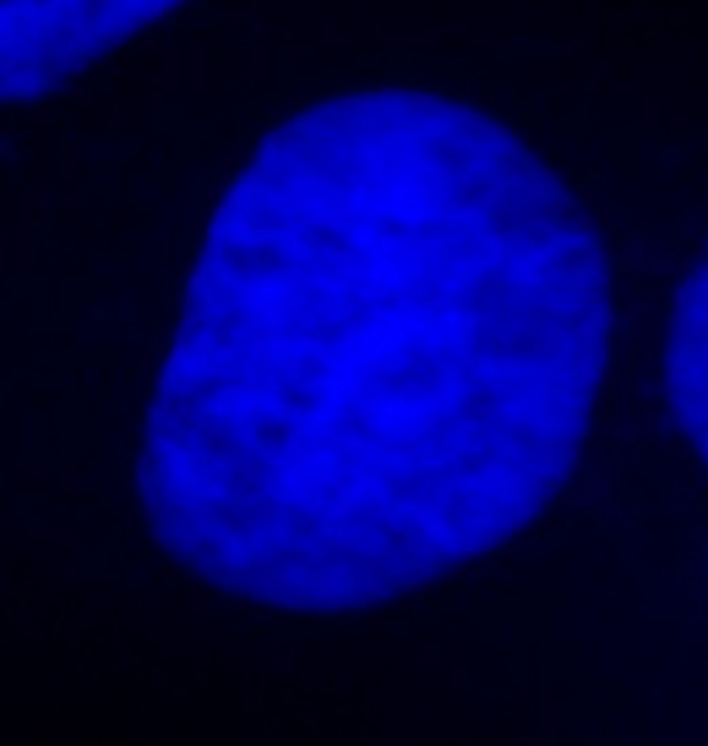

Supplement: Supplementary file 13 — Figure EV2 Source Data [file 44318_2026_783_MOESM13_ESM.zip › Figure EV2/Figure EV2C/S320A_Hoehcst.tif]

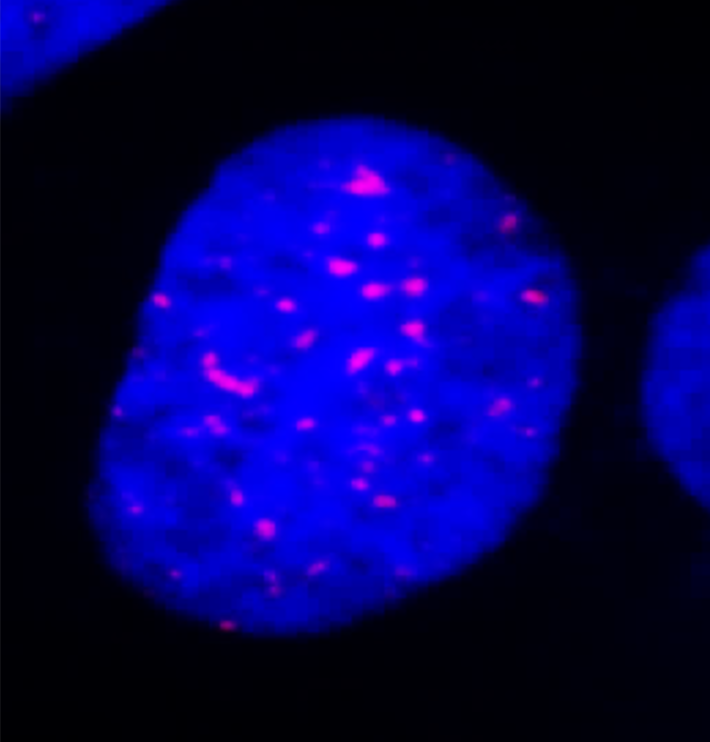

Supplement: Supplementary file 13 — Figure EV2 Source Data [file 44318_2026_783_MOESM13_ESM.zip › Figure EV2/Figure EV2C/S320A_Merged.tif]

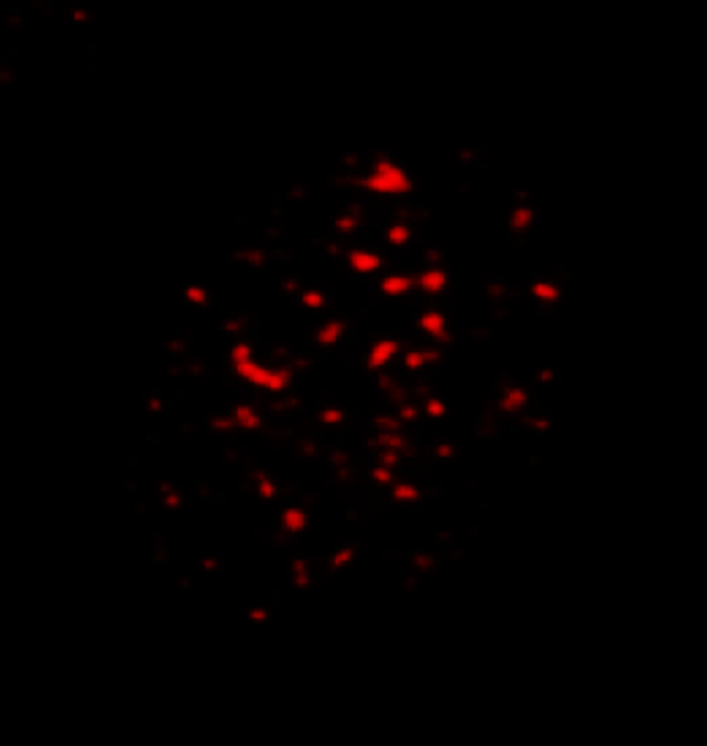

Supplement: Supplementary file 13 — Figure EV2 Source Data [file 44318_2026_783_MOESM13_ESM.zip › Figure EV2/Figure EV2C/S320A_PLA.tif]

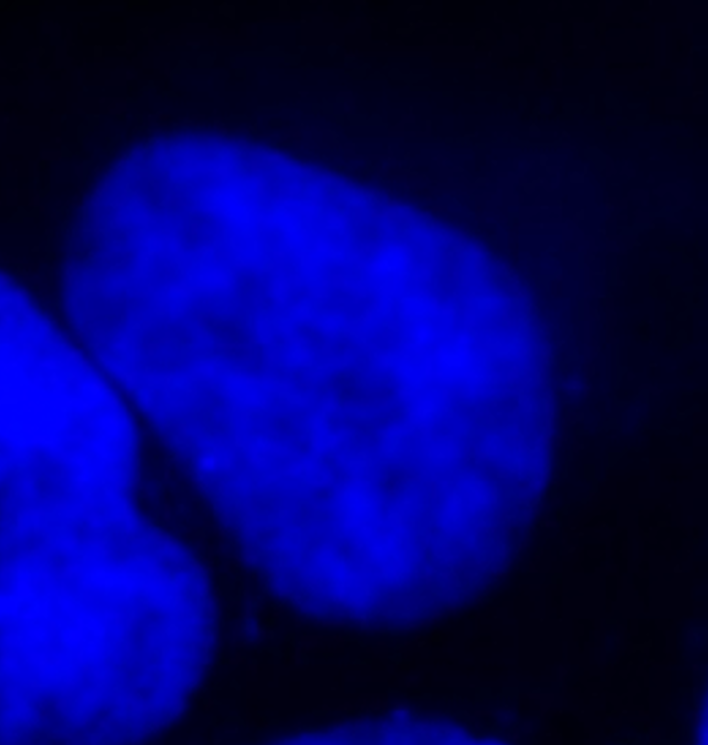

Supplement: Supplementary file 13 — Figure EV2 Source Data [file 44318_2026_783_MOESM13_ESM.zip › Figure EV2/Figure EV2C/S394A_Hoechst.tif]

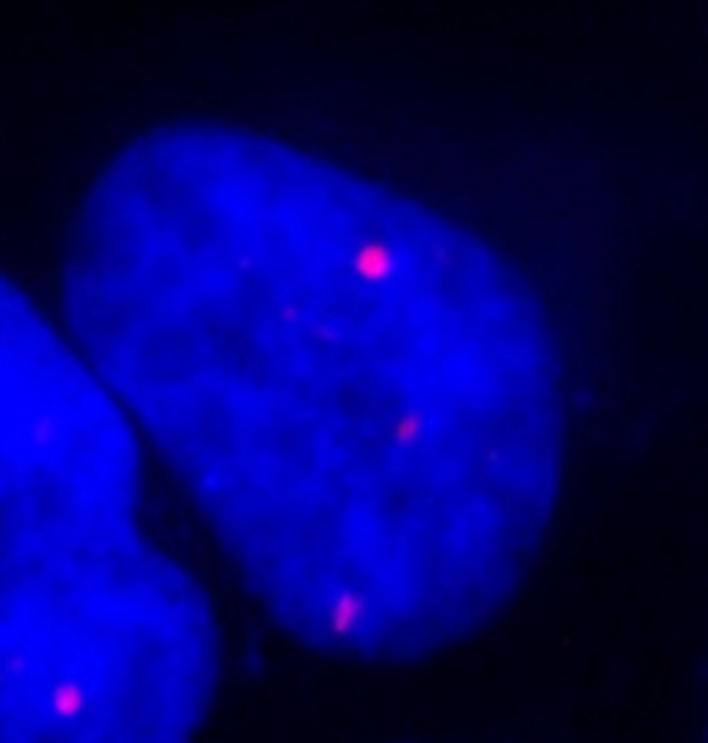

Supplement: Supplementary file 13 — Figure EV2 Source Data [file 44318_2026_783_MOESM13_ESM.zip › Figure EV2/Figure EV2C/S394A_Merged.tif]

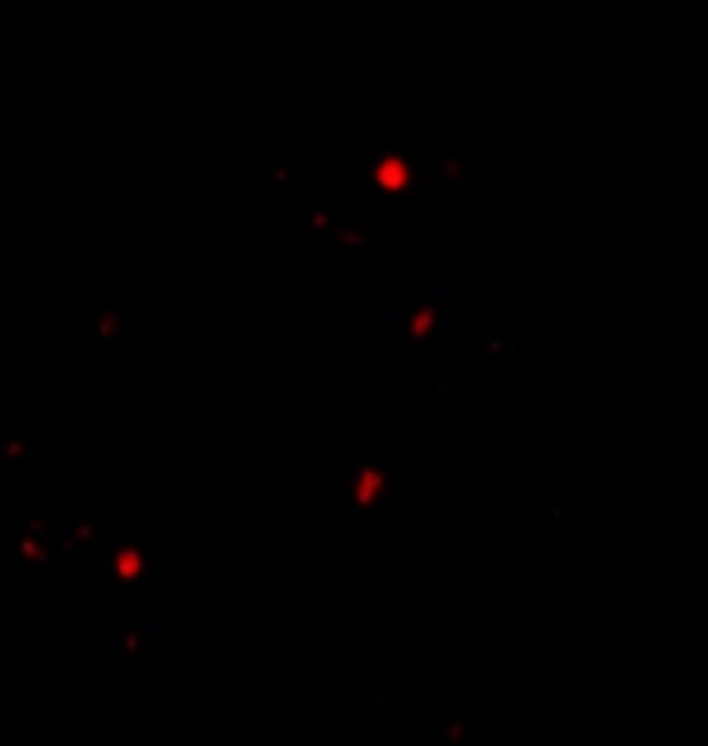

Supplement: Supplementary file 13 — Figure EV2 Source Data [file 44318_2026_783_MOESM13_ESM.zip › Figure EV2/Figure EV2C/S394A_PLA.tif]

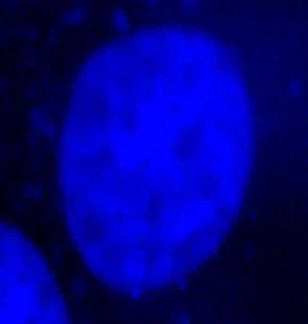

Supplement: Supplementary file 13 — Figure EV2 Source Data [file 44318_2026_783_MOESM13_ESM.zip › Figure EV2/Figure EV2C/T570A_Hoechst.tif]

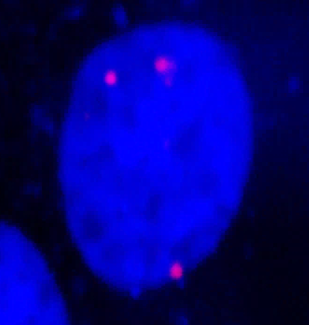

Supplement: Supplementary file 13 — Figure EV2 Source Data [file 44318_2026_783_MOESM13_ESM.zip › Figure EV2/Figure EV2C/T570A_Merged.tif.png]

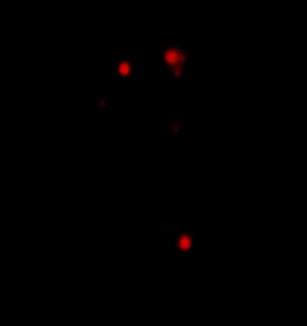

Supplement: Supplementary file 13 — Figure EV2 Source Data [file 44318_2026_783_MOESM13_ESM.zip › Figure EV2/Figure EV2C/T570A_PLA.tif]

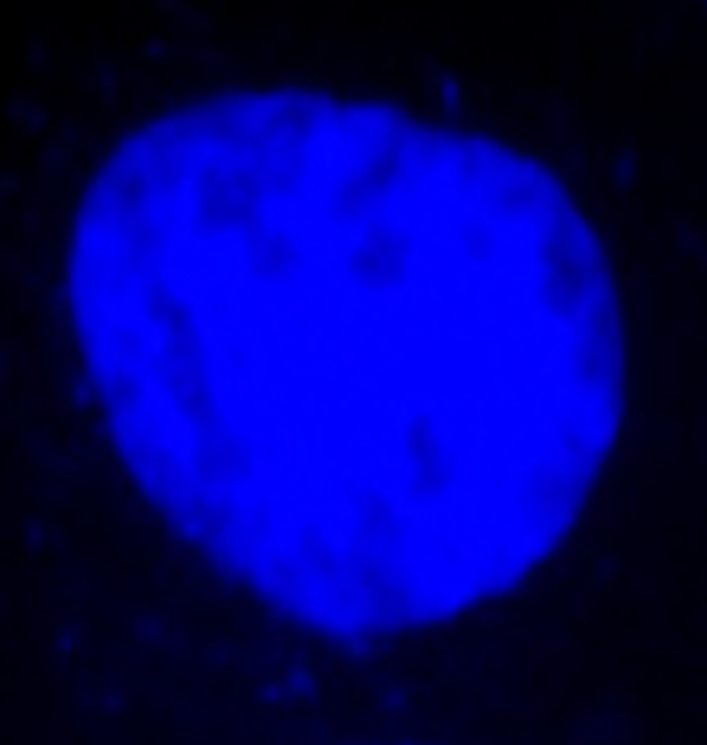

Supplement: Supplementary file 13 — Figure EV2 Source Data [file 44318_2026_783_MOESM13_ESM.zip › Figure EV2/Figure EV2C/WT_Hoechst.tif]

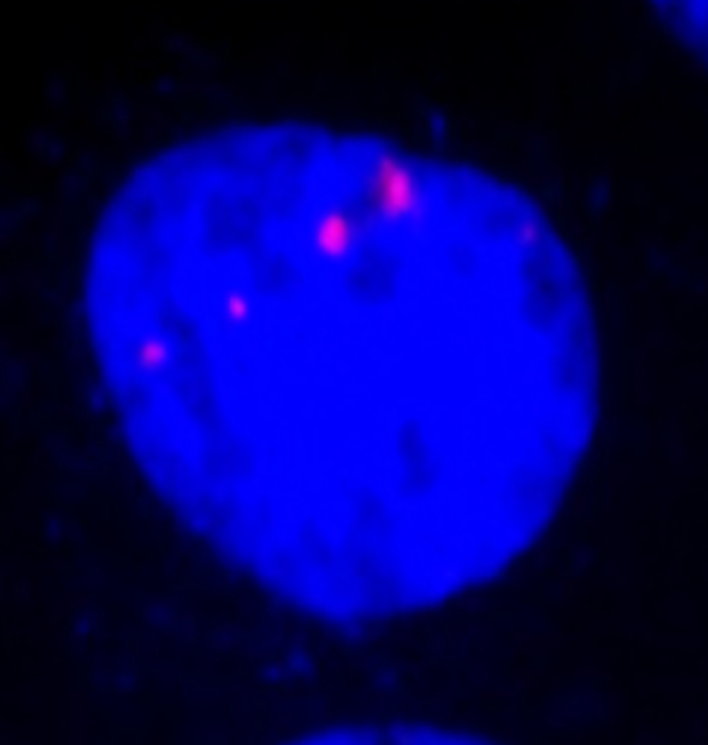

Supplement: Supplementary file 13 — Figure EV2 Source Data [file 44318_2026_783_MOESM13_ESM.zip › Figure EV2/Figure EV2C/WT_Merged.tif]

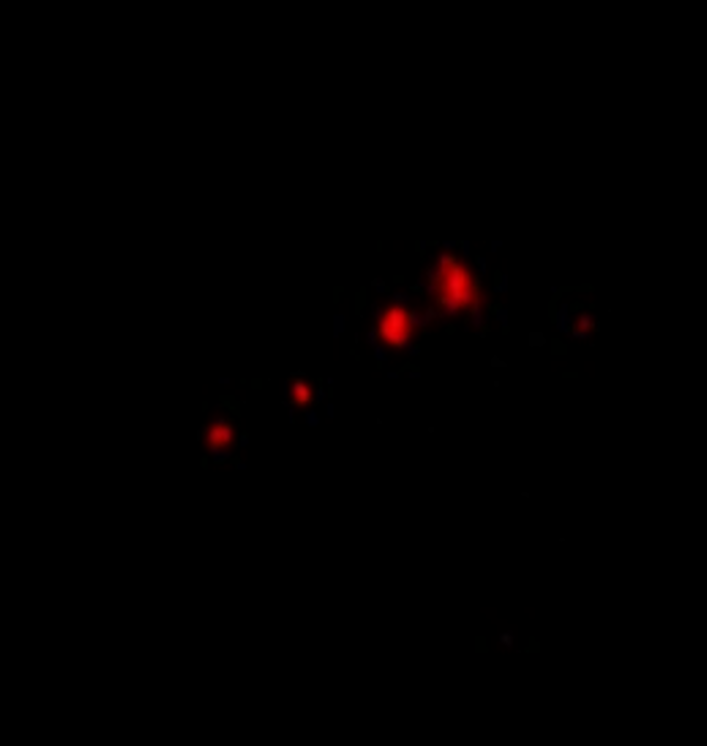

Supplement: Supplementary file 13 — Figure EV2 Source Data [file 44318_2026_783_MOESM13_ESM.zip › Figure EV2/Figure EV2C/WT_PLA.tif]

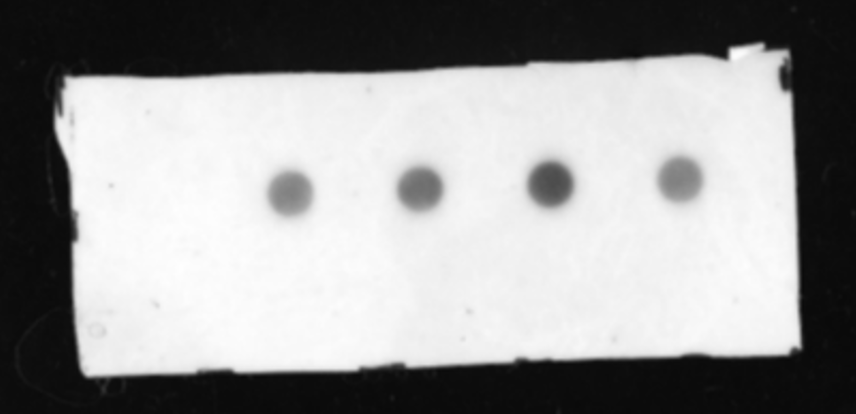

Supplement: Supplementary file 13 — Figure EV2 Source Data [file 44318_2026_783_MOESM13_ESM.zip › Figure EV2/Figure EV2F/Biotin.tif]

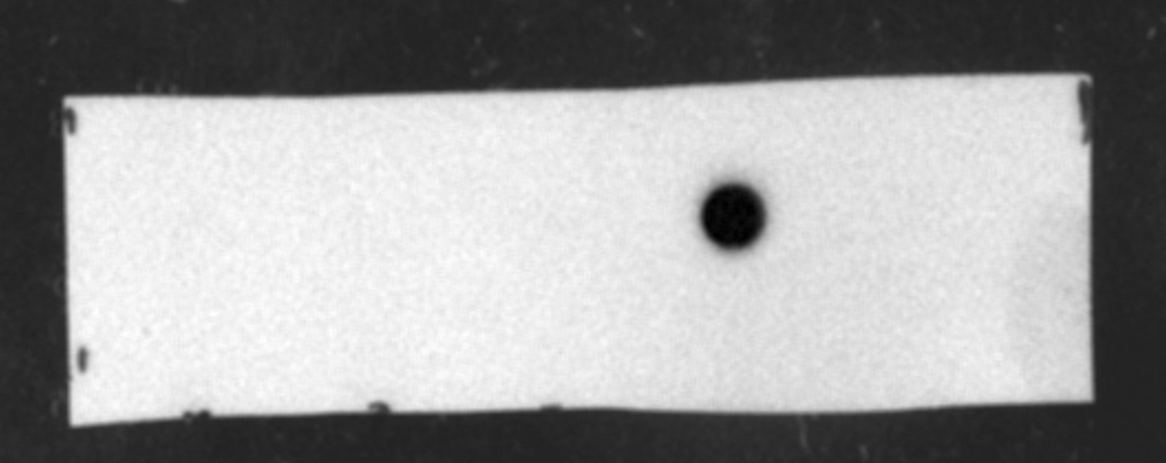

Supplement: Supplementary file 13 — Figure EV2 Source Data [file 44318_2026_783_MOESM13_ESM.zip › Figure EV2/Figure EV2F/Phosphoserine.tif]

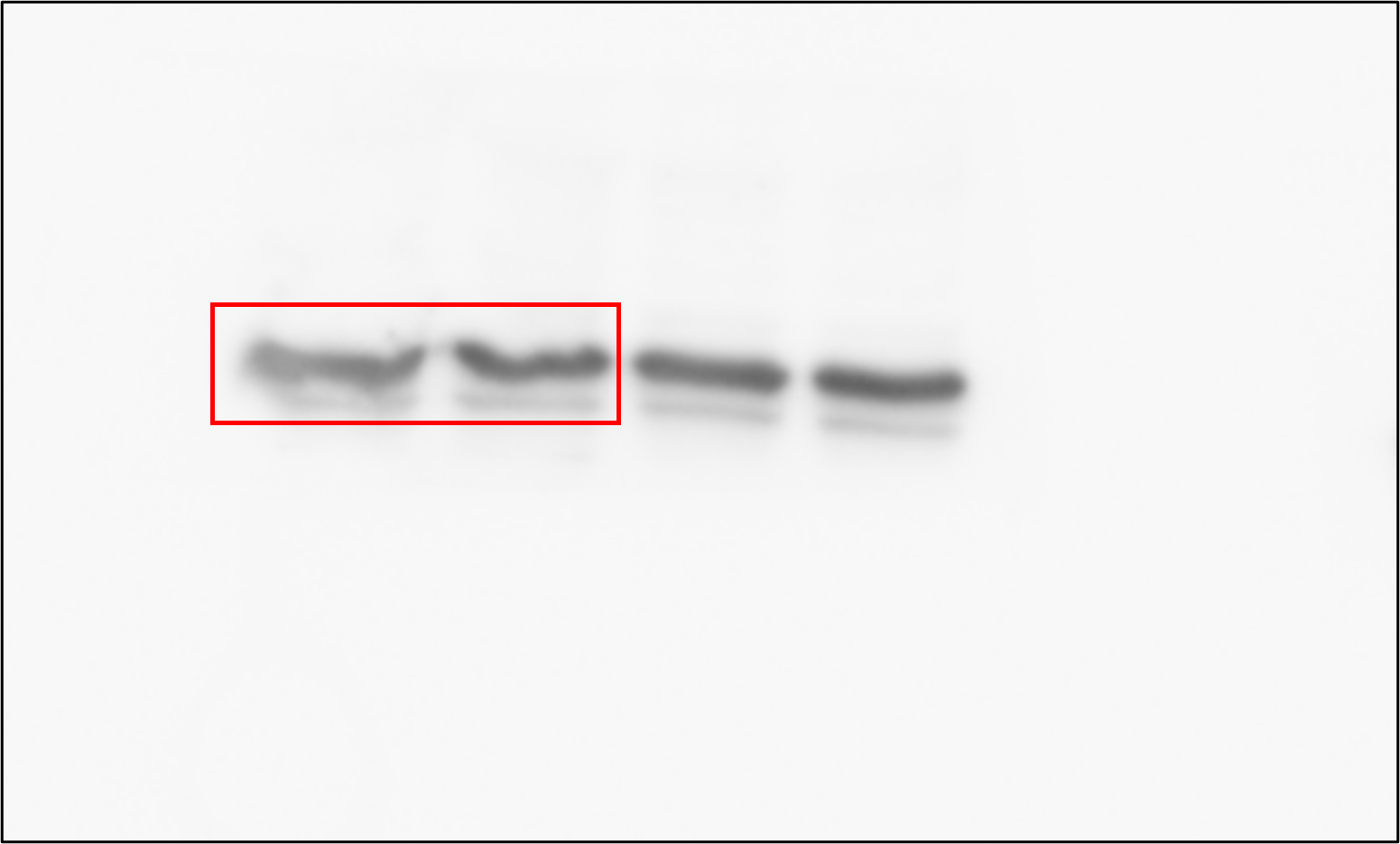

Supplement: Supplementary file 14 — Figure EV3 Source Data [file 44318_2026_783_MOESM14_ESM.zip › Figure EV3/Figure EV3A/GAPDH.tif]

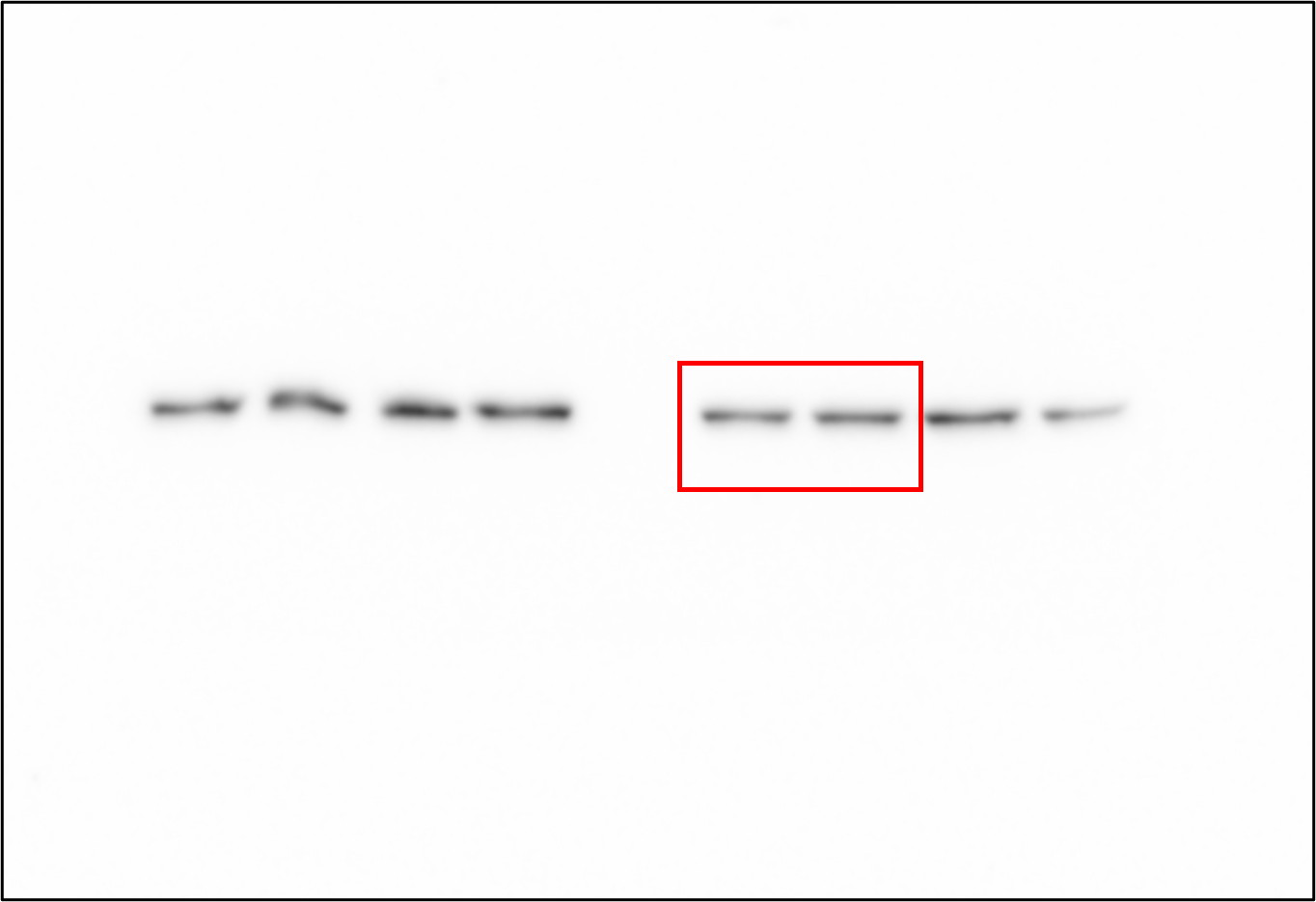

Supplement: Supplementary file 14 — Figure EV3 Source Data [file 44318_2026_783_MOESM14_ESM.zip › Figure EV3/Figure EV3A/GFP.tif]

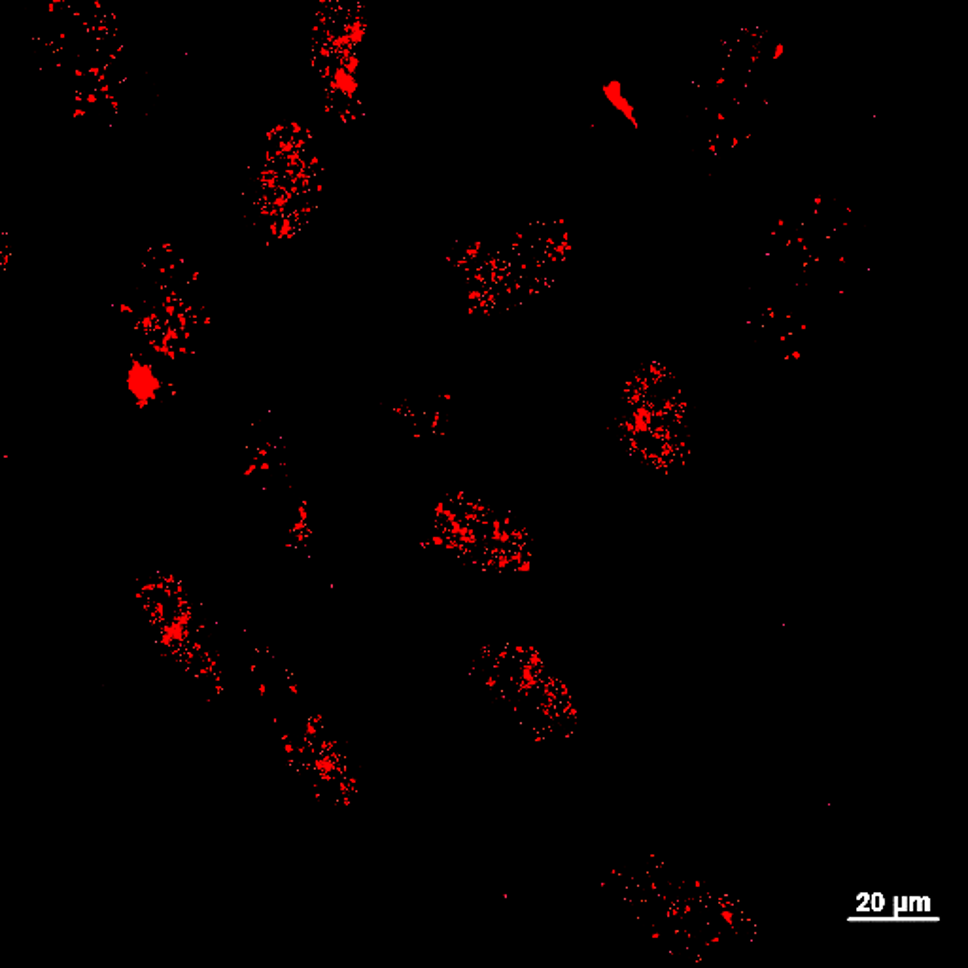

Supplement: Supplementary file 14 — Figure EV3 Source Data [file 44318_2026_783_MOESM14_ESM.zip › Figure EV3/Figure EV3B/S320A_53BP1.tif]

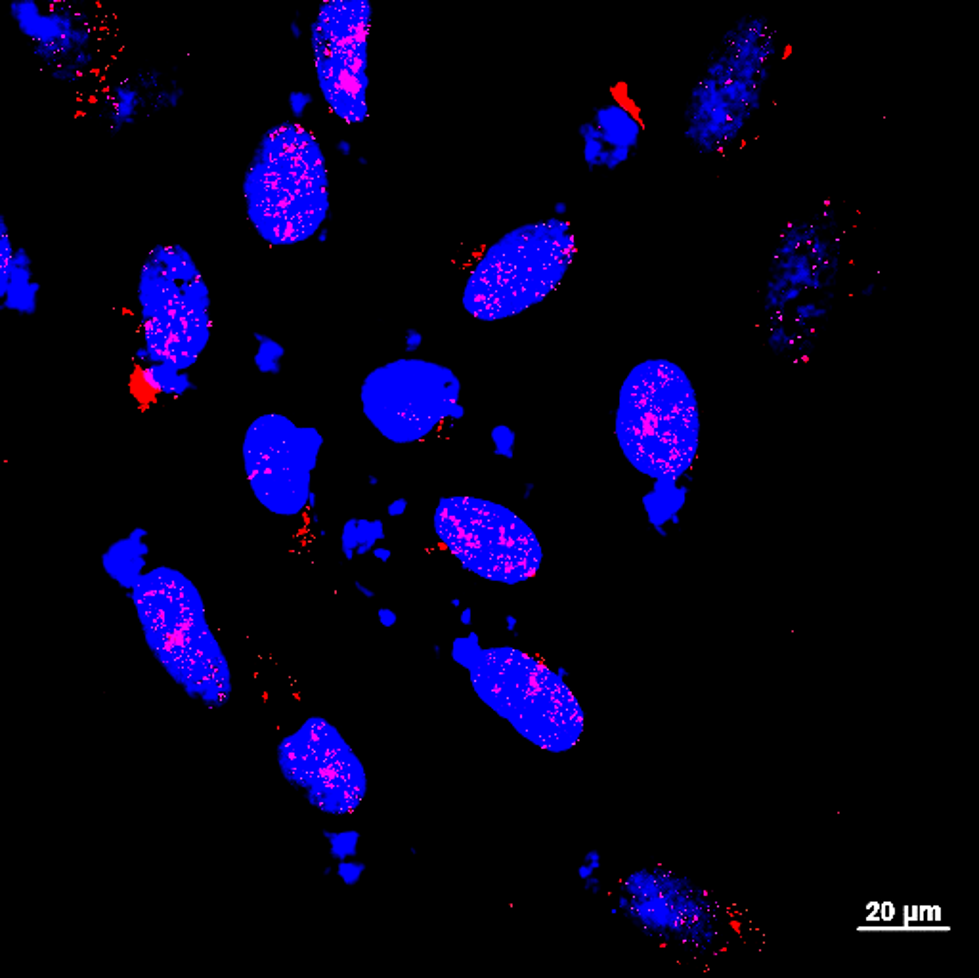

Supplement: Supplementary file 14 — Figure EV3 Source Data [file 44318_2026_783_MOESM14_ESM.zip › Figure EV3/Figure EV3B/S320A_Merged.tif]

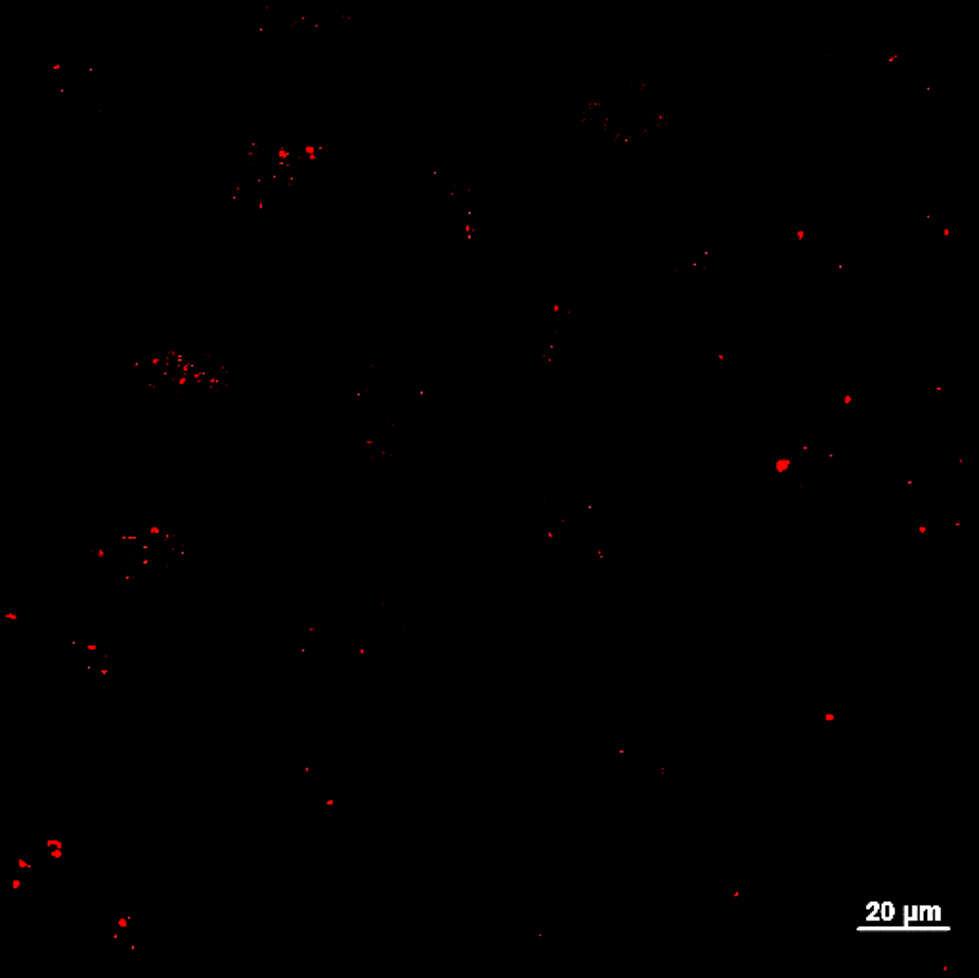

Supplement: Supplementary file 14 — Figure EV3 Source Data [file 44318_2026_783_MOESM14_ESM.zip › Figure EV3/Figure EV3B/WT_53BP1.tif]

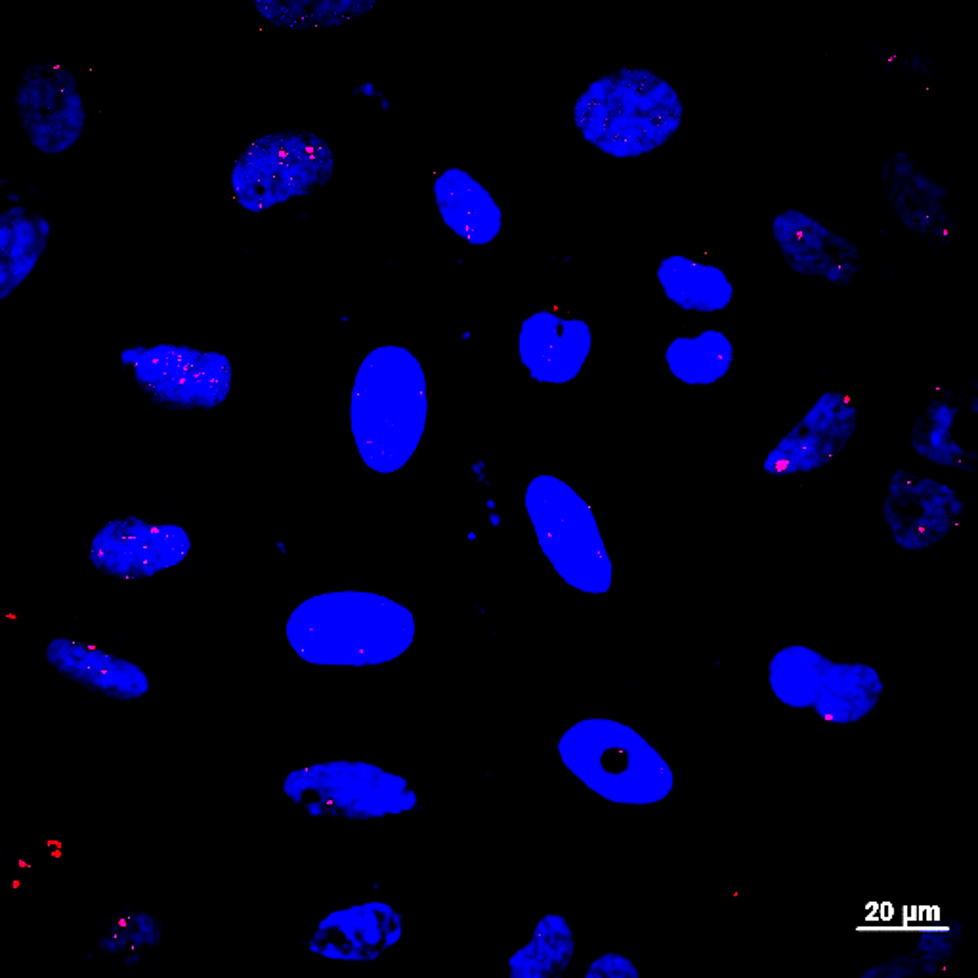

Supplement: Supplementary file 14 — Figure EV3 Source Data [file 44318_2026_783_MOESM14_ESM.zip › Figure EV3/Figure EV3B/WT_Merged.tif]

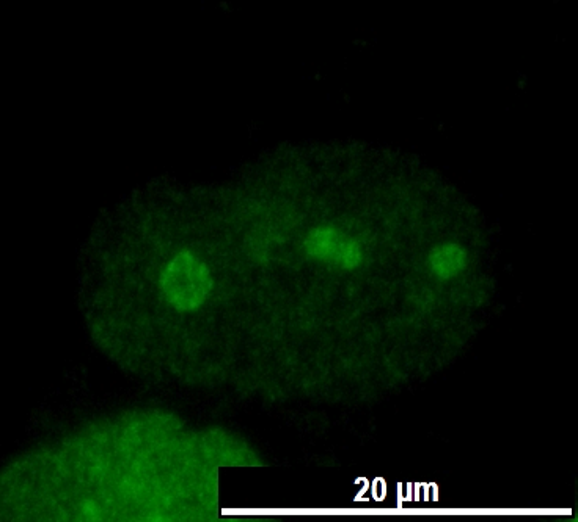

Supplement: Supplementary file 14 — Figure EV3 Source Data [file 44318_2026_783_MOESM14_ESM.zip › Figure EV3/Figure EV3C/S320A_EGFP.tif]

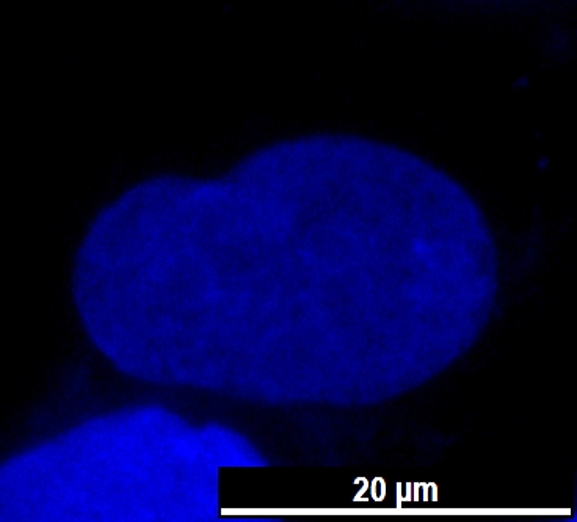

Supplement: Supplementary file 14 — Figure EV3 Source Data [file 44318_2026_783_MOESM14_ESM.zip › Figure EV3/Figure EV3C/S320A_Hoechst.tif]

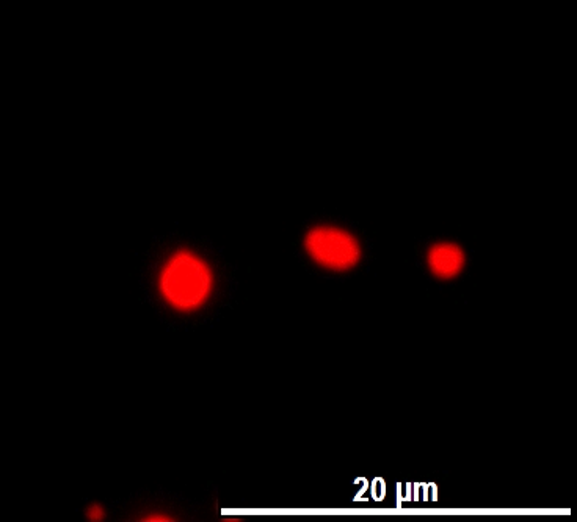

Supplement: Supplementary file 14 — Figure EV3 Source Data [file 44318_2026_783_MOESM14_ESM.zip › Figure EV3/Figure EV3C/S320A_mCherry.tif]

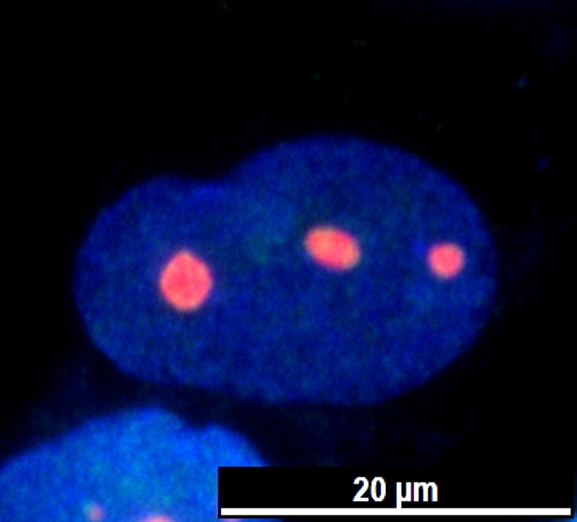

Supplement: Supplementary file 14 — Figure EV3 Source Data [file 44318_2026_783_MOESM14_ESM.zip › Figure EV3/Figure EV3C/S320A_Merged.tif]

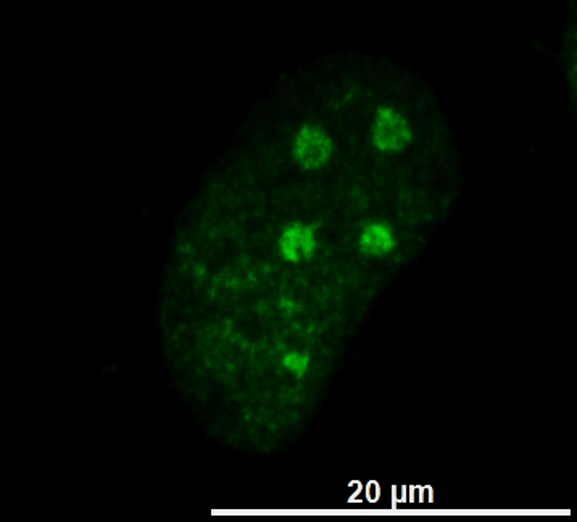

Supplement: Supplementary file 14 — Figure EV3 Source Data [file 44318_2026_783_MOESM14_ESM.zip › Figure EV3/Figure EV3C/WT_EGFP.tif]

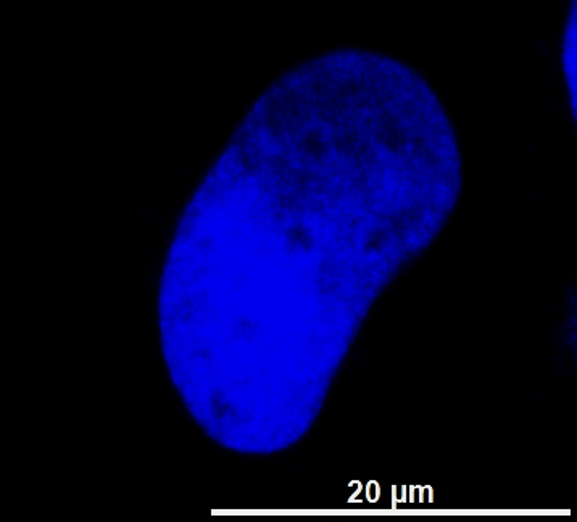

Supplement: Supplementary file 14 — Figure EV3 Source Data [file 44318_2026_783_MOESM14_ESM.zip › Figure EV3/Figure EV3C/WT_Hoechst.tif]

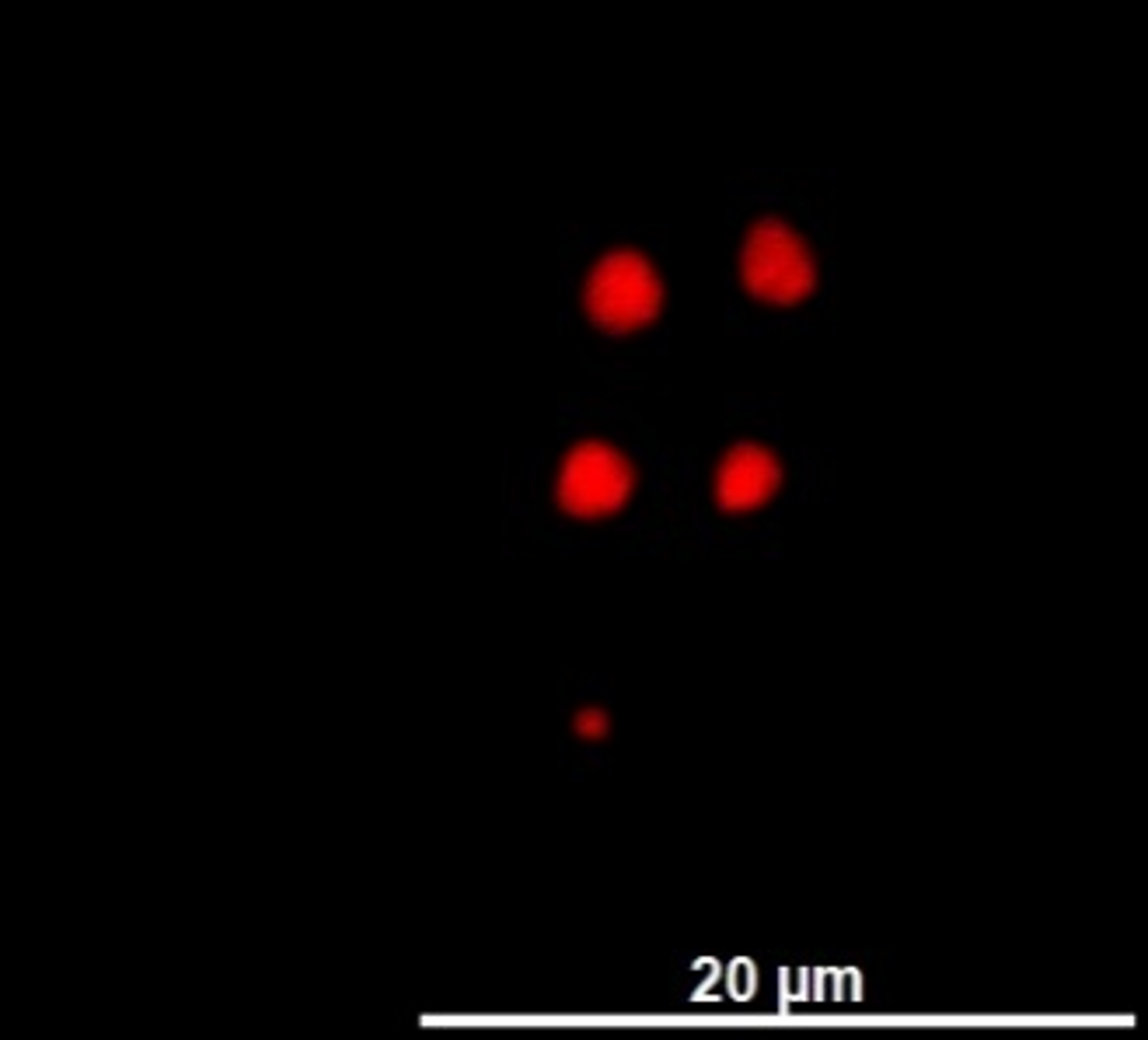

Supplement: Supplementary file 14 — Figure EV3 Source Data [file 44318_2026_783_MOESM14_ESM.zip › Figure EV3/Figure EV3C/WT_mCherry.tif]

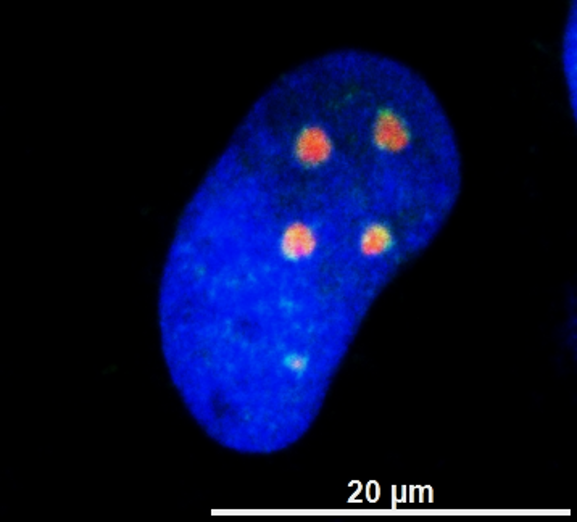

Supplement: Supplementary file 14 — Figure EV3 Source Data [file 44318_2026_783_MOESM14_ESM.zip › Figure EV3/Figure EV3C/WT_Merged.tif]

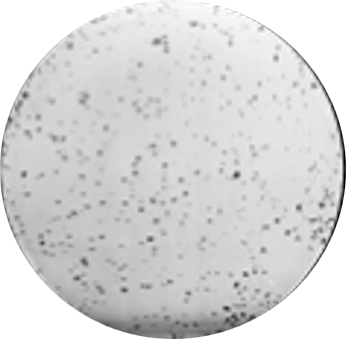

Supplement: Supplementary file 14 — Figure EV3 Source Data [file 44318_2026_783_MOESM14_ESM.zip › Figure EV3/Figure EV3G/S320A.tif]

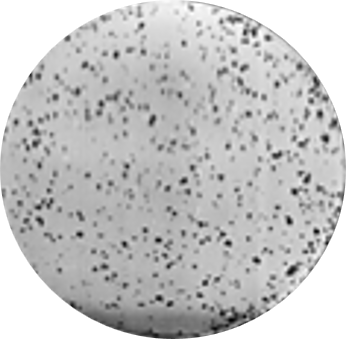

Supplement: Supplementary file 14 — Figure EV3 Source Data [file 44318_2026_783_MOESM14_ESM.zip › Figure EV3/Figure EV3G/WT.tif]

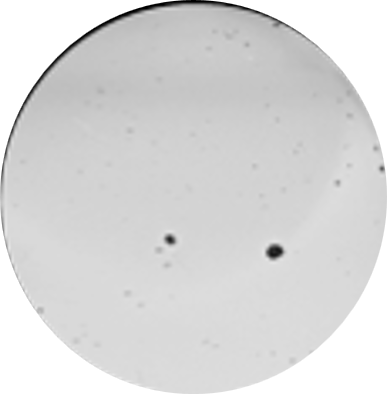

Supplement: Supplementary file 14 — Figure EV3 Source Data [file 44318_2026_783_MOESM14_ESM.zip › Figure EV3/Figure EV3I/S320A_CPT.tif]

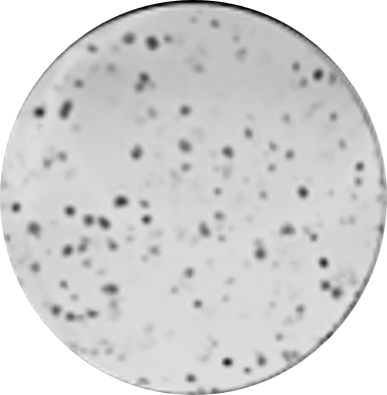

Supplement: Supplementary file 14 — Figure EV3 Source Data [file 44318_2026_783_MOESM14_ESM.zip › Figure EV3/Figure EV3I/S320A_Unreated.tif]

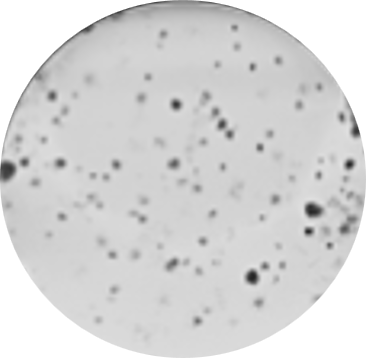

Supplement: Supplementary file 14 — Figure EV3 Source Data [file 44318_2026_783_MOESM14_ESM.zip › Figure EV3/Figure EV3I/WT_CPT.tif]

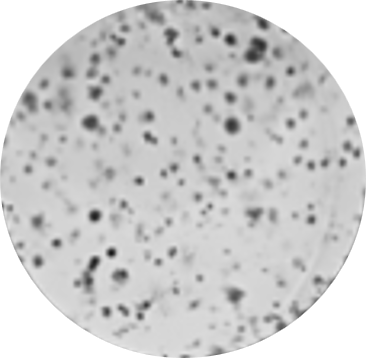

Supplement: Supplementary file 14 — Figure EV3 Source Data [file 44318_2026_783_MOESM14_ESM.zip › Figure EV3/Figure EV3I/WT_Untreated.tif]
