# Supplementary material for: A CHK1-mediated phosphorylation switch suppresses human Topoisomerase 1-associated genomic instability
Source: EMBO J. 2026 May 13;45(12):4220–56. doi: 10.1038/s44318-026-00783-3 (PMC13270093; doi:10.1038/s44318-026-00783-3)
Supplement: Supplementary file 15 — Expanded View Figures [file 44318_2026_783_MOESM15_ESM.pdf]

Expanded View Figures

Figure EV1. Replication-independent stabilization of TOP1ccs in response to CHK1*i*.

(**A–D**) Representative immunofluorescence microscopy images of TOP1cc levels in U2-OS cells subjected to CHK1*i*-treatment in the absence (**A**) or presence of replication inhibition. U2-OS cells were subjected to serum starvation (0.5% fetal bovine serum/FBS, 48 h) (**B**), Aphidicolin (APH, 200 nM, 16 h) (**C**), or CDC7i (PHA, 5 µM, 4 h) (**D**), followed by 250 nM CHK1*i* treatment for 2 h. Cellular TOP1cc levels were detected using anti-TOP1cc monoclonal antibody. Scale bars: 10 µm for (**A–D**). (**E**) Quantitative representation of the replicates shown in (**A–D**). ****$P < 0.0001$. Error bars represent median ± interquartile range. Significance was determined using the Kruskal–Wallis test with Dunn's post hoc analysis. (**F**) Quantification across three replicates of (**A–D**). *P* values are: <0.0001 (Untreated: No CHK1*i vs.* CHK1*i*), <0.0001 (0.5% FBS: No CHK1*i vs.* CHK1*i*), <0.0001 (APH: No CHK1*i vs.* CHK1*i*), <0.0001 (PHA: No CHK1*i vs.* CHK1*i*), 0.176245 (Untreated+CHK1*i vs.* 0.5% FBS+ CHK1*i*), 0.265347 (Untreated +CHK1*i vs.* APH+ CHK1*i*), 0.314577 (Untreated+CHK1*i vs.* PHA+CHK1*i*). 200 cells were scored per condition. Error bars represent SEM. Significance was determined using Kruskal–Wallis test with Dunn's post hoc analysis. Source data are available online for this figure.

 © The Author(s)

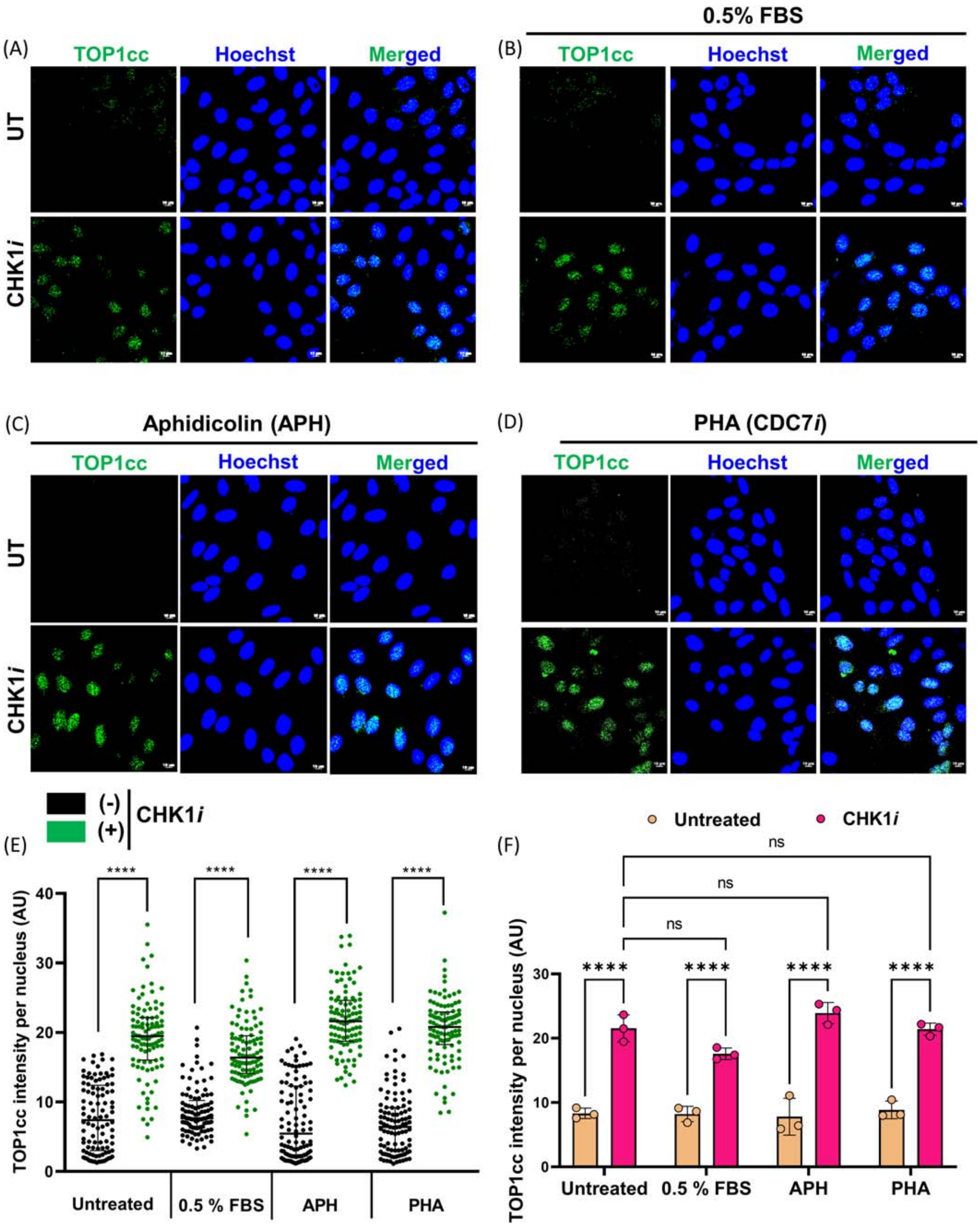
0.5% FBS
(A) TOP1cc Hoechst Merged
(B) TOP1cc Hoechst Merged
UT
CHK1i
(C) Aphidicolin (APH)
(D) PHA (CDC7i)
TOP1cc Hoechst Merged
TOP1cc Hoechst Merged
UT
CHK1i
(-) (+) CHK1i
(E)
TOP1cc intensity per nucleus (AU)
Untreated 0.5 % FBS APH PHA
****
(F)
Untreated CHK1i
ns
TOP1cc intensity per nucleus (AU)
Untreated 0.5 % FBS APH PHA
****

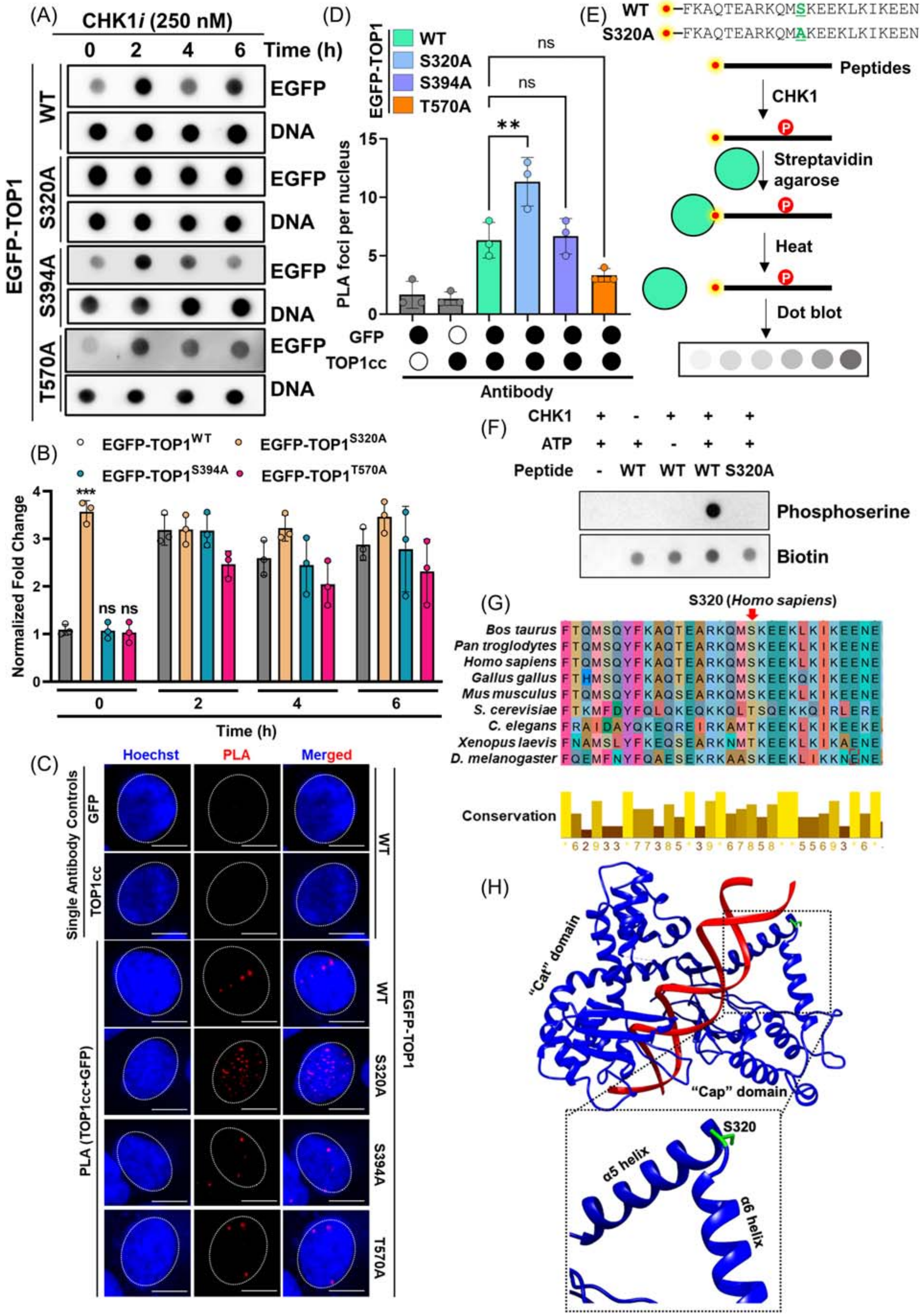
(A)
CHK1i (250 nM)
0 2 4 6 Time (h)
EGFP-TOP1
WT
S320A
S394A
T570A
EGFP
DNA
(B)
EGFP-TOP1WT
EGFP-TOP1S320A
EGFP-TOP1S394A
EGFP-TOP1T570A
Normalized Fold Change
***
ns ns
Time (h)
(C)
Hoechst PLA Merged
Single Antibody Controls
GFP
TOP1cc
PLA (TOP1cc+GFP)
WT
S320A
S394A
T570A
EGFP-TOP1
(D)
EGFP-TOP1
WT
S320A
S394A
T570A
ns
ns
**
PLA foci per nucleus
GFP
TOP1cc
Antibody
(E)
WT FKAQTEARKQMSKEEKLKIKEEN
S320A FKAQTEARKQMAKEEKLKIKEEN
Peptides
CHK1
Streptavidin agarose
Heat
Dot blot
(F)
CHK1 + - + + +
ATP + + - + +
Peptide - WT WT WT S320A
Phosphoserine
Biotin
(G)
S320 (Homo sapiens)
Bos taurus FTQMSQYFKAQTEARKQMSKEEKLKIKEENE
Pan troglodytes FTQMSQYFKAQTEARKQMSKEEKLKIKEENE
Homo sapiens FTQMSQYFKAQTEARKQMSKEEKLKIKEENE
Gallus gallus FTHMSQYFKAQTEARKQMSKEEKQKIKEENE
Mus musculus FTQMSQYFKAQSEARKQMSKEEKLKIKEENE
S. cerevisiae FTKMFDYFQLQKEQKKQLTSQEKKQIRLERE
C. elegans FRAIDAYQKEQREIRKAMTKEEKLKIKEEKE
Xenopus laevis FNAMSLYFKEQSEARKNMTKEEKLKIKAENE
D. melanogaster FQEMFNYFQAESEKRKAASKEEKLIKKNENE
Conservation
(H)
"Cat" domain
"Cap" domain
S320
α5 helix
α6 helix

 © The Author(s)

Figure EV2. Characterization of S320 phosphorylation as a regulator of TOP1 dynamics.

(**A**, **B**) Representative RADAR assay in U2-OS cells transfected with EGFP-TOP1^{WT}, EGFP-TOP1^{S320A}, EGFP-TOP1^{S394A}, or EGFP-TOP1^{T570A} without depletion of endogenous TOP1. Cells were transfected with plasmids expressing EGFP-TOP1^{WT}, EGFP-TOP1^{S320A}, EGFP-TOP1^{S394A}, or EGFP-TOP1^{T570A} followed by treatment with CHK1*i* (250 nM) for 2, 4, or 6 h, 48 h post transfection. Dot blots were probed with anti-GFP antibody. *P* values (0 h) are: 0.007925 (EGFP-TOP1^{WT} *vs.* EGFP-TOP1^{S320A}), 0.966818 (EGFP-TOP1^{WT} *vs.* EGFP-TOP1^{S394A}), and 0.076058 (EGFP-TOP1^{WT} *vs.* EGFP-TOP1^{T570A}) (data from three independent experiments; significance was determined using two-way ANOVA with Tukey test). Error bars represent SD. (**C**, **D**) Representative Proximity Ligation Assay (PLA) images (and quantification across three replicates) of EGFP and TOP1cc in U2-OS cells transfected with EGFP-TOP1^{WT}, EGFP-TOP1^{S320A}, EGFP-TOP1^{S394A}, or EGFP-TOP1^{T570A}. Cells were transfected, followed by PLA with anti-GFP and anti-TOP1cc antibody 48 h post transfection. Nuclei are marked with white dotted lines. Scale bars: 10 µm). *P* values are: 0.0011598 (EGFP-TOP1^{WT} *vs.* EGFP-TOP1^{S320A}), 0.114290 (EGFP-TOP1^{WT} *vs.* EGFP-TOP1^{S394A}), and 0.084425 (EGFP-TOP1^{WT} *vs.* EGFP-TOP1^{T570A}) (data from three independent experiments with 200 cells per condition; significance was determined using Kruskal–Wallis test with Dunn's post hoc analysis). Error bars represent SEM. (**E**, **F**) In vitro kinase assay with recombinant human CHK1 and biotinylated peptides encompassing TOP1 S320 and its phosphoresistant mutant S320A. (**F**) Multiple sequence alignment of TOP1 orthologs from nine eukaryotic species, highlighting conservation of Serine 320. WT and S320A peptides (amino acid highlighted in green) were biotinylated at their N-termini (red). In vitro kinase reactions were performed with recombinant CHK1 and relevant peptides, followed by purification of peptides *via* streptavidin agarose beads (green). Peptides were then dissociated from the beads and subjected to a dot blot. (**G**) Structural context of Serine 320 in the TOP1-DNA complex (PDB ID:1A31). (**H**) Structural context of Serine 320 in TOP1 protein (PDB ID: 1A31). Positioning of Serine 320 relative to α5 and α6 "nose-cone" helices is shown in inset. Source data are available online for this figure.

© The Author(s)

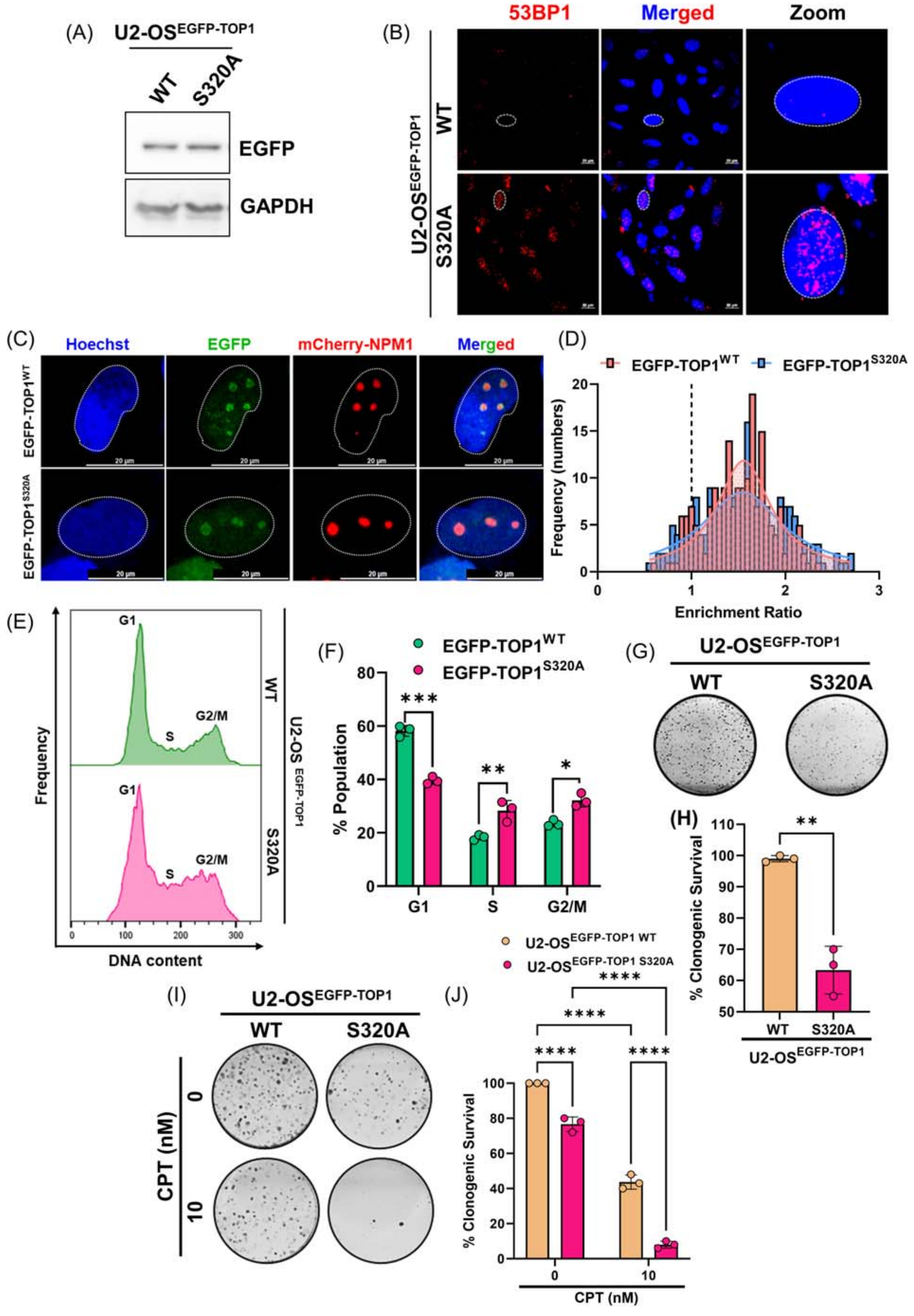
(A)
U2-OS^EGFP-TOP1
WT
S320A
EGFP
GAPDH
(B)
53BP1
Merged
Zoom
U2-OS^EGFP-TOP1
WT
S320A
(C)
Hoechst
EGFP
mCherry-NPM1
Merged
EGFP-TOP1^WT
EGFP-TOP1^S320A
20 µm
(D)
EGFP-TOP1^WT
EGFP-TOP1^S320A
Frequency (numbers)
Enrichment Ratio
(E)
Frequency
DNA content
G1
S
G2/M
WT
S320A
U2-OS EGFP-TOP1
(F)
EGFP-TOP1^WT
EGFP-TOP1^S320A
% Population
G1
S
G2/M
(G)
U2-OS^EGFP-TOP1
WT
S320A
(H)
% Clonogenic Survival
WT
S320A
U2-OS^EGFP-TOP1
(I)
U2-OS^EGFP-TOP1
WT
S320A
CPT (nM)
0
10
(J)
U2-OS^EGFP-TOP1 WT
U2-OS^EGFP-TOP1 S320A
% Clonogenic Survival
CPT (nM)

Figure EV3. Characterization of U2-OS cells stably expressing EGFP-TOP1^{WT} and EGFP-TOP1^{S320A}.

(**A**) Immunoblot showing expression of EGFP-TOP1^{WT} and EGFP-TOP1^{S320A} in U2-OS$^{EGFP\text{-}TOP1WT}$ and U2-OS$^{EGFP\text{-}TOP1S320A}$ stable cells, respectively. (**B**) Representative immunofluorescent microscopic images of 53BP1 levels in U2-OS$^{EGFP\text{-}TOP1WT}$ and U2-OS$^{EGFP\text{-}TOP1S320A}$ cells. See Fig. 7C for quantification. Nuclei are marked with white dotted lines. Scale bars: 10 µm. (**C**) Nuclear distribution of EGFP-TOP1^{WT} and EGFP-TOP1^{S320A} in U2-OS$^{EGFP\text{-}TOP1WT}$ and U2-OS$^{EGFP\text{-}TOP1S320A}$ cells, respectively. U2-OS$^{EGFP\text{-}TOP1WT}$ and U2-OS$^{EGFP\text{-}TOP1S320A}$ cells were transfected with mCherry-NPM1, followed by live cell imaging. Nuclei are marked with white dotted lines. Scale bars: 20 µm. (**D**) Quantification of the experiment shown in (**C**). Refer to the "Methods" section for details on the calculation of the enrichment ratio. Two hundred cells were acquired per condition. (**E**, **F**) Flow cytometric analysis (and quantification thereof) of cell cycle distribution of U2-OS$^{EGFP\text{-}TOP1WT}$ and U2-OS$^{EGFP\text{-}TOP1S320A}$ cells. *$P = 0.012348$, **$P = 0.005529$, ***$P = 0.000197$ (data from three independent experiments; significance was determined using two-way ANOVA with Tukey test). Error bars represent SD. (**G**, **H**) Clonogenic survival of U2-OS$^{EGFP\text{-}TOP1WT}$ and U2-OS$^{EGFP\text{-}TOP1S320A}$ cells. U2-OS$^{EGFP\text{-}TOP1WT}$ and U2-OS$^{EGFP\text{-}TOP1S320A}$ cells were seeded in six-well plates, followed by enumeration of resultant colonies post 7–8 days of incubation. Percentage survival was calculated with respect to the number of colonies in U2-OS$^{EGFP\text{-}TOP1WT}$ cells. **$P = 0.001311$ (data from three independent experiments; significance was determined using unpaired *t* test). (**I**, **J**) Sensitivity of U2-OS$^{EGFP\text{-}TOP1WT}$ and U2-OS$^{EGFP\text{-}TOP1S320A}$ cells toward CPT. U2-OS$^{EGFP\text{-}TOP1WT}$ and U2-OS$^{EGFP\text{-}TOP1S320A}$ cells were seeded in six-well plates, followed by treatment with CPT (10 nM) for 16 h post seeding. Resultant colonies were enumerated after 7–8 days of incubation. ****$P < 0.0001$ (data from three independent experiments; significance was determined using two-way ANOVA with Tukey test). Error bars represent SD. Source data are available online for this figure.

© The Author(s)
