# Supplementary material for: A CHK1-mediated phosphorylation switch suppresses human Topoisomerase 1-associated genomic instability
Source: EMBO J. 2026 May 13;45(12):4220–56. doi: 10.1038/s44318-026-00783-3 (PMC13270093; doi:10.1038/s44318-026-00783-3)
Supplement: Supplementary file 2 — Peer Review File [file 44318_2026_783_MOESM2_ESM.pdf]

THE EMBO JOURNAL

A CHK1-mediated phosphorylation switch suppresses human Topoisomerase 1-associated genomic instability

Ananda Guha Majumdar, Nitish Chauhan, Pooja Gupta, Mahesh Subramanian, and Birija Patro

Corresponding author(s): Birija Patro (bisank@barc.gov.in) , Mahesh Subramanian (maheshs@barc.gov.in)

Review Timeline:

Submission Date:	27th Aug 25
Editorial Decision:	26th Sep 25
Revision Received:	1st Feb 26
Editorial Decision:	2nd Mar 26
Revision Received:	20th Mar 26
Editorial Decision:	30th Mar 26
Revision Received:	3rd Apr 26
Accepted:	9th Apr 26

Editor: Hartmut Vodermaier

Transaction Report:

(Note: With the exception of the correction of typographical or spelling errors that could be a source of ambiguity, letters and reports are not edited. Depending on transfer agreements, referee reports obtained elsewhere may or may not be included in this compilation. Referee reports are anonymous unless the Referee chooses to sign their reports.)

Prof. Birija Sankar Patro
Bhabha Atomic Research Centre
Bio-Organic Division
Mod. Lab
Trombay
Mumbai, Maharashtra 400085
India

26th Sep 2025

Re: EMBOJ-2025-122198
A CHK1-orchestrated phosphorylation switch suppresses Topoisomerase 1-associated genomic instability

Dear Birija,

Thank you again for submitting your manuscript on CHK1 regulation of TOP1ccs to The EMBO Journal. Three expert referees have now assessed it and provided the comments copied below. As you will see, the referees appreciate the potential interest of your findings and acknowledge your use of various complementary experimental approaches. At the same time, they however also raise several important technical and presentational issues that would need to be addressed prior to publication. Should you be able to address these various points to the referees' satisfaction, we would be happy to pursue a revised manuscript further for publication in our journal. Please be reminded, however, that our single-major-revision-round policy makes it important to diligently respond to each referee point at the time of resubmission; therefore, please do not hesitate to contact me early on in case you would like to clarify any of the referees' points, or discuss plans for how to best answer them. We would also be open to extending the revision deadline if that should be helpful. Our scooping protection policy ensures that competing manuscripts published while your work is under revision will not have a negative effect on our final decision.

Further information on preparing, formatting and uploading a revised manuscript can be found below and in our Guide to Authors. Thank you again for the opportunity to consider this work for The EMBO Journal, and I look forward to receiving your revised manuscript in due time.

With kind regards,

Hartmut

Hartmut Vodermaier, PhD
Senior Editor, The EMBO Journal
h.vodermaier@embojournal.org

*** PLEASE NOTE: All revised manuscript are subject to initial checks for completeness and adherence to our formatting guidelines. Revisions may be returned to the authors and delayed in their editorial re-evaluation if they fail to comply to the following requirements (see also our Guide to Authors for further information):

1) Every manuscript requires a Data Availability section (even if only stating that no deposited datasets are included). Primary datasets or computer code produced in the current study have to be deposited in appropriate public repositories prior to resubmission, and reviewer access details provided in case that public access is not yet allowed. Further information: embopress.org/page/journal/14602075/authorguide#dataavailability

2) Each figure legend must specify
- size of the scale bars that are mandatory for all micrograph panels
- the statistical test used to generate error bars and P-values
- the type error bars (e.g., S.E.M., S.D.)
- the number (n) and nature (biological or technical replicate) of independent experiments underlying each data point
- Figures may not include error bars for experiments with n<3; scatter plots showing individual data points should be used instead.

3) Revised manuscript text (including main tables, and figure legends for main and EV figures) has to be submitted as editable text file (e.g., .docx format). We encourage highlighting of changes (e.g., via text color) for the referees' reference.

4) Each main and each Expanded View (EV) figure should be uploaded as individual production-quality files (preferably in .eps,

.tif, .jpg formats). For suggestions on figure preparation/layout, please refer to our Figure Preparation Guidelines: http://bit.ly/EMBOPressFigurePreparationGuideline

5) Point-by-point response letters should include the original referee comments in full together with your detailed responses to them (and to specific editor requests if applicable), and also be uploaded as editable (e.g., .docx) text files.

6) Please complete our Author Checklist, and make sure that information entered into the checklist is also reflected in the manuscript; the checklist will be available to readers as part of the Review Process File. A download link is found at the top of our Guide to Authors: embopress.org/page/journal/14602075/authorguide

7) All authors listed as (co-)corresponding need to deposit, in their respective author profiles in our submission system, a unique ORCiD identifier linked to their name. Please see our Guide to Authors for detailed instructions.

8) Please note that supplementary information at EMBO Press has been superseded by the 'Expanded View' for inclusion of additional figures, tables, movies or datasets; with up to five EV Figures being typeset and directly accessible in the HTML version of the article. For details and guidance, please refer to: embopress.org/page/journal/14602075/authorguide#expandedview

9) To facilitate reproducibilty and cross-laboratory adoption of methodologies, please structure the Materials & Methods section as outlined in our guide to authors, including a completed Reagents and Tools Table that can be downloaded from our author guidelines as well (https://www.embopress.org/page/journal/14602075/authorguide#structuredmethods).

10) Digital image enhancement is acceptable practice, as long as it accurately represents the original data and conforms to community standards. If a figure has been subjected to significant electronic manipulation, this must be clearly noted in the figure legend and/or the 'Materials and Methods' section. The editors reserve the right to request original versions of figures and the original images that were used to assemble the figure. Finally, we generally encourage uploading of numerical as well as gel/blot image source data; for details see: embopress.org/page/journal/14602075/authorguide#sourcedata

At EMBO Press we ask authors to provide source data for the main manuscript figures. You will receive a separate email with instructions for providing source data with your revised manuscript, including how to upload and organize the files.

Further information is available in our Guide For Authors: https://www.embopress.org/page/journal/14602075/authorguide

In the interest of ensuring the conceptual advance provided by the work, we recommend submitting a revision within 3 months (25th Dec 2025). Please discuss the revision progress ahead of this time with the editor if you require more time to complete the revisions. Use the link below to submit your revision:

Link Not Available

Referee #1:

In this manuscript, the authors identify that CHK1 phosphorylates topoisomerase I (TOP1) on Ser320 under physiological conditions, which is critical for proper TOP1 catalytic activity, notably the religation reaction. They further show that defective TOP1 Ser320 phosphorylation leads to the accumulation of cellular TOP1 cleavage complexes (TOP1ccs), and to the subsequent induction of DNA double-strand breaks caused by replication-transcription conflicts. This is a compelling study that provides significant insights into the mechanism by which TOP1 activity (which is essential for many DNA transactions), is regulated, and is of general interest. Although the experiments are generally convincing, some points should be addressed to further support the conclusions and to improve the manuscript.

Major concerns

1. This study strongly relies on the use of CHK1 inhibitors, which can have non-specific effects. To exclude the possibility that such non-specific effects contribute to the observed increase in TOP1cc levels, the authors should test whether CHK1 depletion (e.g., by siRNA or CRISPR) increases TOP1ccs, and whether under these conditions CHK1 inhibitors have no additional effect. In addition, TOP1 can be trapped under physiological conditions by DNA alterations. Therefore, can it be excluded that CHK1 inhibition induces DNA damage, which then contributes to the observed increase in TOP1cc levels?

2. To directly demonstrate that CHK1 phosphorylates TOP1 at Ser320, the in vitro kinase assay shown in Fig. 4G should also

include the TOP1-S320A mutant.

3. Additional information and analyses should be provided to clarify the reproducibility of some experiments:
- Dot blot experiments to assess TOP1ccs and R-loops in the manuscript should be quantified relative to the dsDNA signal and tested for statistical significance.
- It is not indicated whether the single-cell analyses of the immunofluorescence data (e.g., in the main figures: Figs. 1G, 2B, 4F, 5F, 7B,C,E, and 8B,D,F) are presented as a mix of cells from independent experiments (which could lead to misleading results) or from a representative single-cell experiment. A representative single-cell experiment should be presented and the statistical significance of the data should be tested on the mean of independent experiments.
- The number of biological replicates should be indicated in the figure legend for each experiment.

4. Fig. 3 is confusing. It is unclear why the authors analyze the effect of CHK1 inhibition on CPT-induced post-translational modifications of TOP1 (ubiquitylation, sumoylation, PARylation) and TOP1 degradation, when the key question would be the effect of CHK1 inhibition alone on these events. CPT would be useful only as a positive control. To properly analyze these effects, the duration of CHK1 inhibitor treatment should be extended under conditions that clearly increase TOP1cc levels (e.g., 6 h with 250 nM SCH). Fig. 3A also raises concerns. As mentioned in the legend, if an alkaline lysis method was indeed used to analyze TOP1 by WB, the disappearance of TOP1 upon a 1 h-CPT treatment would not reflect TOP1 degradation, but rather the induction of TOP1ccs (using alkaline extracts, band depletion occurs when TOP1 is bound to chromatin as TOP1ccs). This would be consistent with numerous reports showing that CPT indeed induces TOP1ccs after 1 h but that TOP1 degradation requires more time (typically 4 to 6 h). This also raises questions about the CHK1 inhibitor conditions, which do not show a decrease in TOP1 (Fig 3A), even though under these conditions TOP1ccs are induced (as detected in the loading control of DUST assays in Fig. 3F).

Minor concerns

1. To provide a better understanding of the role of CHK1-mediated TOP1 phosphorylation in TOP1 catalytic activity, the authors should perform in vitro experiments to determine whether the TOP1 S320A mutant affects the TOP1 cleavage reaction, in addition to its effect on religation.

2. Although the increase in γH2AX in TOP1-S320A cells compared to TOP1-WT cells appears more pronounced in EdU^+ cells, EdU^- cells also show an increase (Fig. 7D,E). Therefore, the experiment in Fig. 8A,B using transcription inhibitors should also be performed in EdU^+ vs EdU^- cells.

3. The Methods section is insufficiently detailed and should be carefully revised. As illustrative examples, the antibodies and cell extract preparation used for WB are not indicated (for cell extracts, this information is crucial to interpret Fig. 3A, as mentioned above); it is not indicated which antibody was used for RADAR (anti-TOP1 or anti-TOP1cc); for the single-cell experiments, the number of cells analyzed and the details of the quantification procedure are often missing.

4. In Fig. 7D,E, γH2AX is analyzed in the green channel but the cells express EGFP-TOP1 constructs, which should also emit green fluorescence. This raises concern about signal overlap.

5. The authors should discuss how CHK1-mediated phosphorylation of TOP1 promotes TOP1 catalytic activity, e.g., in terms of TOP1 conformation or interaction with other proteins?

Referee #2:

This manuscript titled 'A CHK1-orchestrated phosphorylation switch suppresses human Topoisomerase 1- associated genomic instability' by Majumdar et al. describes how CHK1 regulates TOP1 cleavage complex formation under physiological conditions. In a series of experiments, they demonstrate that CHK1 has a prominent role in preventing TOP1ccs under physiological conditions. They start by screening relevant DDR-associated kinases, pick up CHK1 from the screen (one of the top hits) and demonstrate stabilisation of TOP1cc with two distinct CHK1 inhibitors. In order to rule out secondary consequences arising from CHK1 inhibition, they provide experiments that rule out a direct influence from replication, transcription or associated pathways that degrade TOP1. Physical interaction by IP followed by an in vitro kinase assay helps them further strengthen their claim. They then identify specific amino acid residues within TOP1, generate mutants and directly demonstrate the impact of TOP1-Ser320 in DNA relaxation and the TOP1 catalysis cycle. They wrap up the story by demonstrating the impact of TOP1-Ser320 in genome stability by a series of elegant experiments from the replication perspective, comprising of EdU incorporation assay, single molecule DNA fibre assay and metaphase spreads and further back it up from the transcription side by providing all the relevant experiments.
The manuscript is well structured, and multiple techniques/methodologies are employed to demonstrate their findings.
Taken together, the work uncovers a previously unrecognised mechanism by which CHK1 phosphorylation of TOP1 at Serine residue 320 regulates TOP1 catalytic activity, maintains genome stability, ensuring proper replication dynamics, preventing

transcription-replication conflicts, and limiting chromosomal instability.
However, this work has several limitations and needs to be revised before supporting its publication.

A major shortcoming of this study is the failure to discuss the role of ATR, the kinase upstream of Chk1, considering that it is also found in their screen, and CHK1 is literally inactive without ATR. This can be easily visible in their Figure 4G, where phosphorylation of TOP1 is weak. Is this an ATR-CHK1-dependent process or only a CHK1-dependent process? This is essential to address.
Additionally, some statements are not visible in their results. E.g. In Figure 3E, they claim that the CHK1 inhibitor does not affect the ubiquitination status, but I see that it does, as the ubiquitin signal/smear is strongly reduced after CPT and CHK1 inhibitor treatment when compared to CPT treatment alone. The quantification of results in several independent experiments will help them to draw their conclusion.
They also did not consider the fact that CHK1inh might affect TOP1ccs (local/chromatin fraction) degradation, but not global TOP1 degradation.
Also, some aspects of chromosomal degradation of TOP1ccs are omitted from their investigation (Wss1/SPRTN; Cdc48 PMID: 24998930, PMID: 32152270).
Also, the statement that we do not know how cells prevent Top1ccs under physiological conditions is not correct. Several papers show this. For instance: PMID: 32152270

The ectopically expressing TOP1 wild-type (TOP1wt) and TOP1 Ser320Ala (TOP1S320A) cell lines should be characterised in terms of TOP1 localisation and cell cycle distribution and kinetics.

Additional Comments
Introduction
Relevant publications need to be cited when discussing the removal of TOP1cc (e.g., Wss1/SPRTN)
Also, when discussing the action of WRN on CHK1 activation, the author cites articles that do not describe the actual mechanism. A small paragraph about canonical CHK1 activation through polymerase-helicase uncoupling upon TOP1cc induction or fork stalling needs to be added.
What do the author mean by basal CHK1 activity? How this is activity regulated? I would assume that this is independent of long-stretches of ssDNA related to fork stalling. Please discuss non-canonical activation of CHK1 that is linked with DPCs and explain why such activity is relevant for normal physiological processes and how this is relevant to this manuscript.

Results
Figure1
Better gel for 1E needed
1F Complementary Western blot (pATM, pKAP1 or pCHK2) is needed to demonstrate that the experimental condition does not lead to DSB formation. 1 µM CPT is well known for DSB induction, which will activate ATM and DNA-PKcs. Demonstrate the same with 100 or 200 nM CPT instead.
It is important to supplement these (TOP1 mobility) data with ssDNA staining under non-denaturing conditions or by total RPA staining. It will help to clarify the status of the canonical signal for CHK1 activation i.e., ssDNA. Does inhibition of CHK1 lead to more ssDNA? There must be clarity.

Figure 2
C-H instead of values at the bottom of the gels, the authors must provide line graphs which are easy to read.
Figure 3
A-B Explanation regarding the differences in methodology with respect to alkaline lysis and RADAR assay will provide more clarity to the general readers.
A What is the justification for using 10 and 25 µM CPT. A Western blot is needed to show the status of pATM, pKAP1 and pCHK2 under the same conditions.
C-E a schematic of the assay will help the general readers about the experimental setup.
G High concentration of CPT is used.
It will be ideal to do a Western analyses with all CPT conditions (dose and time) and demonstrate that it does not induce DSBs or activate associated kinases.

Figure 5
D Ideally, these experiments should have been performed after depletion of endogenous TOP1. Line graphs have to be provided

Figure 6
Data presented with different font sizes is difficult to read. The authors must fix these, which are common throughout the manuscript and present these figures in a clear and consistent manner.
The concentration of plasmid in the relaxation assay is missing. Please comment on why Proteinase K has not been used before running the reaction products on a gel. In the methodology, please mention why post-staining is performed for the general readers

Figure 7
A Western blot for stable expression is needed
Font sizes need to be uniform
High-resolution images for metaphase chromosomes are needed

Figure 8
A-B does not say anything about DSB; please stain DSB markers (53BP1) or rephrase

Figure 9
A How do you rule out DSB induction and ATM activation under 2 µM CPT for 2hrs. The appearance of DSBs will change the overall interpretation.

Additional unclear points throughout the manuscript:
1. In supplementary Figure 10 A, it appears that the GFP signal indicating the expression of S320A is not present in all cells. Corresponding IF experiments (Fig. 7 A, D) should include GPF signal and non-single cell assays, like the fibre assay could be used following FACS sorting of GFP-positive cells.
2. In the experiments involving ectopic and transient expression of TOP1 wild-type (TOP1wt) and S320A further evidence of expression in multiple cells is required
3. Clearly indicate the number of events/cells analyzed per condition and experiment (Fig. 4E, F, Fig. 5E, F, Fig. 7A-C, Fig. 8A,B).
4. Figure 3H is confusing. The effect of CtIP on intensity of TOP1cc foci in dotblot (3H) does not correspond to the quantification graph (3I).
5. The entire set of experiment is performed in cancer cell lines (U2OS, PANC-1, A549, HCT116, and MDA-MB-231). To substantiate the claim that the observed effects are not cell type-specific, it is essential to demonstrate them in non-transformed human cell lines.
6. While the study focuses on phospho-resistant mutants, it would be equally informative to explore the complementary approach of constitutive phosphorylation. For instance, employing a phospho-mimetic mutant such as TOP1S320E could provide valuable insights into TOP1 mobility in FRAP, catalytic activity (religation assay), and genome stability.
Minor Comments
1. In Figure 1 legend "At least 200 nuclei were scored per sample." Is repeated.
2. TOP1ccs stands for Topoisomerase 1 cleavage complexes, not for the TOP1-DNA covalent complex, although this is indeed a TOP1-DNA covalent complex. (Page 1, line 10 and 11)
3. (Page 17, lines 396 - 400) The possibility of cofactors recognizing the phosphate group on TOP1 and initiating degradation is not mentioned in the discussion; only conformational differences are referenced.
4. Throughout the Materials and Methods section, time units are inconsistently abbreviated (e.g., minutes/min, hours/h). Ensure consistency.
5. Figure 8 (C) R-loops in the cytoplasm with RNase H (-) may need a different image; (F) a clearer image for wild-type and RNase H (-) would enhance clarity. The difference in density is not as strong as suggested by the statistics provided.
6. Remove "250 nM" behind CHK1i in Fig. 3G.
7. Fig. 2B the figure legend indicates that cells were treated with CHKi1 for 4 and 6h, figure itself says 2 and 4h.
Typos
1. Page 2, Line 927: ... formation of transient a TOP1-DNA covalent complex...
2. Page 20, Line 489: ... various therapeutics cannot not be attributed...
3. Page 34, Line 817: ... periodic voxtexing...

Referee #3:

In this manuscript by Majumdar et al, the authors identify TOP1 Serine-320 phosphorylation by CHK1 as a regulatory mechanism that controls TOP1 re-ligation and maintains steady state TOP1cc levels in the cells. Furthermore, the authors show that the ectopic overexpression on the phospho-mutant impairs replication and results in genome instability. The authors suggest that impairment of this mechanism results fork slowdown and also replication transcription conflicts. This study is well done, and the authors have used multiple different approaches to demonstrate the phenomenon and critical role of this phosphorylation of TOP1 in maintain genome stability. I have few suggestions which I think would make the conclusions even more robust.
Comments:
1. The authors have used a constitutive overexpression system of the TOP1 phospho-mutant for all their experiments. I propose that the perform the most critical experiments with an siRNA for TOP1 to downregulate endogenous TOP1 and then use a siRNA resistant phospho-mutant overexpression to.
2. If the model that the authors propose were to be true, i.e. TOP1 cc mediated fork slowdown and transcription inhibition caused replication transcription conflicts, transient inhibition of transcription or RNAaseH overexpression should be able to rescue the genome instability that they observed upon phospho0mutant overexpression. The authors should be careful about the transient transcription inhibitors as they can also cause cell cycle arrest and should include controls to demonstrate that transcription is inhibited and not the replication process. Furthermore the authors should also show that RNAase H overexpression in these contexts also reduces R-loop formation.

Point wise Response to Referees:

Referee #1

In this manuscript, the authors identify that CHK1 phosphorylates topoisomerase I (TOP1) on Ser320 under physiological conditions, which is critical for proper TOP1 catalytic activity, notably the religation reaction. They further show that defective TOP1 Ser320 phosphorylation leads to the accumulation of cellular TOP1 cleavage complexes (TOP1ccs), and to the subsequent induction of DNA double-strand breaks caused by replication-transcription conflicts. This is a compelling study that provides significant insights into the mechanism by which TOP1 activity (which is essential for many DNA transactions), is regulated, and is of general interest. Although the experiments are generally convincing, some points should be addressed to further support the conclusions and to improve the manuscript.

Response: We thank the Reviewer for finding the experiments are largely convincing and for his/her insightful comments for improvement the manuscript. We have tried our best to address his/her queries diligently, as mentioned below.

Major Comments

1. (A) This study strongly relies on the use of CHK1 inhibitors, which can have non-specific effects. To exclude the possibility that such non-specific effects contribute to the observed increase in TOP1cc levels, the authors should test whether CHK1 depletion (e.g., by siRNA or CRISPR) increases TOP1ccs, and whether under these conditions CHK1 inhibitors have no additional effect.

   (B) In addition, TOP1 can be trapped under physiological conditions by DNA alterations. Therefore, can it be excluded that CHK1 inhibition induces DNA damage, which then contributes to the observed increase in TOP1cc levels?

   Response: We thank the reviewer for the valuable suggestions.

   (A) As suggested by the reviewer, CHK1 was depleted by CHK1 siRNA, while non-specific siRNA was used as control, followed by treatment with CHK1 inhibitor (SCH). Our results reveal that (1) silencing of CHK1 results in constitutive stabilization of TOP1ccs on the genome and (2) CHK1 inhibition under siCHK1 condition does not trigger any additional

TOP1cc stabilization. The data is now included in the revised manuscript (Fig. 1G, H; Appendix Fig. S3I).

(B) In order to address if CHK1*i*-induced DNA damage triggers TOP1cc trapping, we measured γH2AX levels in U2-OS cells treated with 250 nM SCH or 50 nM UCN-01 for 2, 4 or 6 h (4 Gy IR was used as a positive control). Our results reveal that CHK1 inhibition under our experimental conditions do not induce significant DNA damage. These results are included in the revised manuscript (Appendix Fig. S4).

2. To directly demonstrate that CHK1 phosphorylates TOP1 at Ser320, the in vitro kinase assay shown in Fig. 4G should also include the TOP1-S320A mutant.

Response: We truly appreciate this insightful suggestion of the reviewer. Inclusion of the TOP1-S320A mutant in the in vitro kinase assay would indeed be informative. We have tried our best to purify TOP1-WT and TOP1-S320A in bacterial systems, however, we could not succeed to get the desired expressions of these proteins due to toxicity associated with human TOP1 expression in bacterial systems. Currently, we do not have infrastructures for eukaryotic systems (e.g., baculovirus/yeast), which is considered to be better systems for human TOP1 purification. Nevertheless, we have addressed the reviewer's central concern by performing following complementary cell-based experiments that directly test whether CHK1 phosphorylates TOP1 at Serine-320 *in cellulo*.

(A) We transfected U2-OS cells with EGFP-$TOP1^{WT}$ and EGFP-$TOP1^{S320A}$, followed by immunoprecipitation with GFP-trap magnetic agarose under untreated on CHK1*i*-treated conditions. This was followed by immunoblotting with anti-phosphoserine antibody. Our results (Fig. 5H, I) revealed multiple key observations:

i. CHK1*i* drastically reduces serine phosphorylation on EGFP-$TOP1^{WT}$, indicating that CHK1 does indeed phosphorylate TOP1 inside cells.

ii. EGFP-$TOP1^{S320A}$ displayed significantly reduced serine phosphorylation (compared to EGFP-$TOP1^{WT}$) under untreated conditions, revealing that TOP1 is indeed phosphorylated at Serine 320 position inside cells.

iii. While CHK1*i* drastically reduces serine phosphorylation on EGFP-$TOP1^{WT}$, it has minimal effect on EGFP-$TOP1^{S320A}$ phosphorylation. This suggests that Serine 320 is the principal CHK1-targeted site on TOP1.

Taken together, these results demonstrate that TOP1 is phosphorylated by CHK1 at Serine 320 position inside cells.

(B) In order to further establish TOP1 S320 as a CHK1 substrate, we synthesized a biotinylated peptide encompassing TOP1 S320, and its corresponding mutant peptide bearing S320A mutation. We performed *in vitro* kinase assay with CHK1 and these peptides. Peptides were then isolated using streptavidin-agarose beads, subjected to dot blotting and then detected with anti-phosphoserine antibody. Our results (Fig. EV2E, F) reveal that CHK1 robustly phosphorylates the peptide encompassing TOP1 S320, and this phosphorylation is completely abolished in the S320A mutant, thus establishing that TOP1 S320 is indeed a target of CHK1-mediated phosphorylation.

3. Additional information and analyses should be provided to clarify the reproducibility of some experiments:

(A) Dot blot experiments to assess TOP1ccs and R-loops in the manuscript should be quantified relative to the dsDNA signal and tested for statistical significance.

(B) It is not indicated whether the single-cell analyses of the immunofluorescence data (e.g., in the main figures: Figs. 1G, 2B, 4F, 5F, 7B,C,E, and 8B,D,F) are presented as a mix of cells from independent experiments (which could lead to misleading results) or from a representative single-cell experiment. A representative single-cell experiment should be presented and the statistical significance of the data should be tested on the mean of independent experiments.

(c) The number of biological replicates should be indicated in the figure legend for each experiment.

Response: We sincerely acknowledge the reviewer's concern.

(A) In the original manuscript, normalized grey intensity values (normalized to dsDNA signal) ± SD were provided underneath each blot. However, statistical significance was not provided with each data. Hence, in order to address the reviewer's concern, we have now calculated normalized fold change (with respect to dsDNA signal and untreated samples where applicable), and plotted line graphs (or bar graphs where applicable) with statistical analyses at relevant places.

(B) We thank the reviewer for the valuable suggestion. The authors would like to clarify that the representative photomicrographs of single cell experiments are generated from samples

belonging to a single replicate. The quantifications were derived from the replicate used to generate the representative image. As per the reviewer's suggestion, graphs have now been plotted (along with statistical analyses) with means of three independent replicates. In the revised manuscript, both graphs are included (main figures contain the representative replicate, while appendix figures contain data from all three replicates) for each single cell experiment (contained in main figures, expanded view figures as well as appendix figures), and clarifications as to the source of the data have been provided in corresponding figure legends.

(C) Information pertaining to the number of biological replicates has been introduced into all figure legends in the revised manuscript as suggested by the reviewer.

4. (A) Fig. 3 is confusing. It is unclear why the authors analyze the effect of CHK1 inhibition on CPT-induced post-translational modifications of TOP1 (ubiquitylation, sumoylation, PARylation) and TOP1 degradation, when the key question would be the effect of CHK1 inhibition alone on these events. CPT would be useful only as a positive control.

   (B) To properly analyze these effects, the duration of CHK1 inhibitor treatment should be extended under conditions that clearly increase TOP1cc levels (e.g., 6 h with 250 nM SCH).

   (C) Fig. 3A also raises concerns. As mentioned in the legend, if an alkaline lysis method was indeed used to analyze TOP1 by WB, the disappearance of TOP1 upon a 1 h-CPT treatment would not reflect TOP1 degradation, but rather the induction of TOP1ccs (using alkaline extracts, band depletion occurs when TOP1 is bound to chromatin as TOP1ccs). This would be consistent with numerous reports showing that CPT indeed induces TOP1ccs after 1 h but that TOP1 degradation requires more time (typically 4 to 6 h). This also raises questions about the CHK1 inhibitor conditions, which do not show a decrease in TOP1 (Fig 3A), even though under these conditions TOP1ccs are induced (as detected in the loading control of DUST assays in Fig. 3F).

   Response: We thank the reviewer for raising these pertinent questions.

   (A) Cellular TOP1cc levels are maintained through a fine balance between TOP1cc formation (a characteristic of the TOP1 catalytic cycle or orchestrated by the presence of a drug such as CPT) and TOP1cc degradation (either drug-induced degradation or basal degradative pathways such as SPRTN). In the original manuscript, the experimental question we attempted to answer in Figure 3 was whether CHK1 regulates cellular TOP1/TOP1cc

degradation machinery, resulting in CHK1*i*-mediated TOP1cc stabilization on the genome. In order to do that, we took advantage of the fact that CPT-mediated TOP1cc stabilization is followed by rapid TOP1cc degradation and global TOP1 downregulation. Hence, we hypothesized that if CHK1 indeed regulates the cellular TOP1cc/TOP1 degradation machinery, then CHK1*i* should interfere with degradation of TOP1ccs stabilized by CPT. Accordingly, global TOP1 levels as well as TOP1cc levels were measured in CPT-treated U2-OS cells with or without pre-treatment with CHK1*i*. CHK1*i* was found to be incapable of preventing CPT-mediated TOP1cc degradation/global TOP1 degradation, thus revealing that CHK1 does not regulate the cellular TOP1cc degradation machinery.

(B) As suggested by the reviewer, we have performed DUST assays for SUMOylation, Ubiquitination and PARylation under CHK1*i* treatment (250 nM) for 2, 4 and 6 h. The data is now included in the revised manuscript (Figure 3G). Our findings suggest that CHK1*i* treatment does not induce degradative PTMs on TOP1ccs.

(C) We would like to clarify respectfully that we have employed the TOP1 downregulation assay (Lin *et al*, 2008), which is distinct from the TOP1 band depletion assay (Desai *et al*, 1997). In TOP1 downregulation assay, alkaline lysates are neutralized followed by digestion of genomic DNA with Micrococcal nuclease or Benzonase in order to release trapped TOP1ccs (this was also mentioned in Method section in the original manuscript). The TOP1 downregulation assay is used for absolute measurement of global TOP1 levels as opposed to the band depletion assay, which is used to measure soluble TOP1 levels. Further, we have deliberately used high CPT doses in order to trigger rapid TOP1 degradation (for global TOP1 measurement). In our experimental system, we have observed that while TOP1 degradation begins ~4-6 h post 1 µM CPT treatment (Guha Majumdar *et al*, 2023), treatments beyond 10 µM concentration trigger rapid degradation as early as 1 h. Our experimental results are in agreement with other reports demonstrating rapid TOP1 degradation beginning as early as 1 h upon treatment with 20 or 25 µM CPT (Lin *et al*, 2009; Sun *et al*, 2020). This is also consistent with rapid ubiquitination of TOP1ccs, which begins as early as 30 min post exposure to CPT (Sun *et al*, 2020). Interestingly, CHK1 inhibitor, under our experimental conditions, induces TOP1cc but not their degradation. To avoid confusion, we have now mentioned that "the alkaline assay was performed along with micrococcal nuclease or Benzonase" in the result section itself.

Minor Comments

1. To provide a better understanding of the role of CHK1-mediated TOP1 phosphorylation in TOP1 catalytic activity, the authors should perform *in vitro* experiments to determine whether the TOP1 S320A mutant affects the TOP1 cleavage reaction, in addition to its effect on religation.

   Response: We thank the reviewer for the valuable suggestion. As suggested by the reviewer, cleavage reactions were performed with immunoprecipitated EGFP-TOP1^{WT} and EGFP-TOP1^{S320A}, and the resulting data has now been included in the revised manuscript (Figure 6H, I). Our experiments reveal that EGFP-TOP1^{WT} and EGFP-TOP1^{S320A} are equally competent in the cleavage reaction, and their kinetic differences lie in the religation step of catalysis (Fig. 6J, K).

2. Although the increase in γH2AX in TOP1-S320A cells compared to TOP1-WT cells appears more pronounced in EdU$^+$ cells, EdU$^-$ cells also show an increase (Fig. 7D,E). Therefore, the experiment in Fig. 8A,B using transcription inhibitors should also be performed in EdU$^+$ vs EdU$^-$ cells.

   Response: We thank the reviewer for the insightful observation. According to results presented in the original manuscript, there is indeed statistically significant elevation in γH2AX as well as 53BP1 in cells expressing EGFP-TOP1^{S320A} compared to cells expressing EGFP-TOP1^{WT} (statistical analysis now included in Figure 7E in the revised manuscript). Hence, as per the reviewer's suggestion, we have treated U2-OS cells stably expressing EGFP-TOP1^{S320A} with DRB and measured rescue in γH2AX levels in EdU$^+$ vs EdU$^-$ cells. Our results (Appendix Fig. S12C, D) reveal that transcription inhibition results in statistically significant reduction in γH2AX levels in EdU$^+$ as well as EdU$^-$ cells.

3. The Methods section is insufficiently detailed and should be carefully revised. As illustrative examples, the antibodies and cell extract preparation used for WB are not indicated (for cell extracts, this information is crucial to interpret Fig. 3A, as mentioned above); it is not indicated which antibody was used for RADAR (anti-TOP1 or anti-TOP1cc); for the single-cell experiments, the number of cells analyzed and the details of the quantification procedure are often missing.

Response: We thank the reviewer for the suggestion and apologize for this lapse in the original manuscript. As suggested by the reviewer, the methods section has now been thoroughly revised and details of antibodies used for western blotting and RADAR assay have been included.

The authors would like to clarify that the alkaline lysis method was described in detail in the methods section of the original manuscript under "Assay for TOP1 downregulation". In order to improve readability, the relevant section has now been renamed as "Assay for TOP1 downregulation (alkaline lysis method)".

As suggested, the details of quantification procedure are now included in the methods section in the revised manuscript. The number of events analysed in single cell experiments are mentioned in corresponding figure legends.

4. In Fig. 7D,E, γH2AX is analyzed in the green channel but the cells express EGFP-TOP1 constructs, which should also emit green fluorescence. This raises concern about signal overlap.

   Response: We thank the reviewer for the pertinent question. Our experimental system reduces background EGFP-specific signal in a two-fold manner: (i) We have observed drastic reduction of EGFP signal after 4% paraformaldehyde fixation at pH 8, which is performed prior to all our immunofluorescence assays. (ii) In order to further reduce the background signal from EGFP, we perform pre-extraction of cells with 0.5% Triton X-100 in PBS for 7 min on ice prior to fixation. This results in extensive loss of soluble EGFP-TOP1/TOP1 from nuclei. The combined effect of these two factors results in barely detectable background EGFP-specific fluorescence compared to the intense fluorescence signal obtained with AlexaFluor-tagged secondary antibodies. We apologize for the lack of sufficient detail pertaining to these experiments in the methods section. These details have now been included in the revised manuscript.

5. The authors should discuss how CHK1-mediated phosphorylation of TOP1 promotes TOP1 catalytic activity, e.g., in terms of TOP1 conformation or interaction with other proteins?

   Response: We thank the reviewer for the suggestion. The authors would like to clarify that the putative mechanism through which CHK1-mediated phosphorylation might regulate TOP1 catalytic activity was discussed in the original manuscript. We would like to draw the

reviewer's attention to the following excerpt which was a part of the discussion section of the original manuscript:

> "Recent theoretical as well as molecular dynamics (MD) simulation-based calculations have highlighted the critical impact of serine phosphorylation on bending propensities of protein backbones. Given its strategic positioning at the loop between the α5 and α6 "nose-cone" helices, phosphorylation of S320 might be implicated in their relative movement, and in turn, their interaction with the DNA substrate. The indispensable role played by the relative dynamics of the "nose-cone" helices has been evidenced by localized non-isomorphism between multiple crystal structures in this region, and theoretically predicted by MD simulations. These studies provide a putative structural basis for our observations."

In connection to the likelihood of other cofactors recognizing phosphorylated TOP1 S320 to promote its catalytic activity, the authors would like to submit that the possibilities are indeed multitudinous. However, since the authors have not experimentally approached the subject, a detailed discussion of the same might risk indulging in conjecture. Hence, the authors have not ventured into an incisive deliberation of the same. However, to include the possibility of cofactors recognizing TOP1 phosphorylation and augmenting its catalytic activity, the authors have included the following statement in the discussion section of the revised manuscript:

"Further, the possibility to other cofactors recognizing phosphorylation on TOP1 Serine 320 to promote its catalytic activity may not be entirely improbable, especially in the light of a seminal study which demonstrated a critical role played by RNA Pol II in regulating TOP1 catalytic activity. However, such a conjecture remains open to investigation"

Referee #2

This manuscript titled 'A CHK1-orchestrated phosphorylation switch suppresses human

Topoisomerase 1- associated genomic instability' by Majumdar et al. describes how CHK1 regulates TOP1 cleavage complex formation under physiological conditions. In a series of experiments, they demonstrate that CHK1 has a prominent role in preventing TOP1ccs under physiological conditions. They start by screening relevant DDR-associated kinases, pick up CHK1 from the screen (one of the top hits) and demonstrate stabilisation of TOP1cc with two distinct CHK1 inhibitors. In order to rule out secondary consequences arising from CHK1 inhibition, they provide experiments that rule out a direct influence from replication, transcription or associated pathways that degrade TOP1. Physical interaction by IP followed by an in vitro kinase assay helps them further strengthen their claim. They then identify specific amino acid residues within TOP1, generate mutants and directly demonstrate the impact of TOP1-Ser320 in DNA relaxation and the TOP1 catalysis cycle. They wrap up the story by demonstrating the impact of TOP1-Ser320 in genome stability by a series of elegant experiments from the replication perspective, comprising of EdU incorporation assay, single molecule DNA fibre assay and metaphase spreads and further back it up from the transcription side by providing all the relevant experiments.

The manuscript is well structured, and multiple techniques/methodologies are employed to demonstrate their findings. Taken together, the work uncovers a previously unrecognised mechanism by which CHK1 phosphorylation of TOP1 at Serine residue 320 regulates TOP1 catalytic activity, maintains genome stability, ensuring proper replication dynamics, preventing transcription-replication conflicts, and limiting chromosomal instability. However, this work has several limitations and needs to be revised before supporting its publication.

Response: We thank the Reviewer for acknowledging the multiple complementary techniques in the manuscript and also for appreciating the well-structured nature of the manuscript. We are also grateful to the Reviewer for recognizing that the study uncovers a previously unrecognized mechanism whereby CHK1-mediated phosphorylation of TOP1 at serine 320 regulates TOP1 catalytic activity, maintains genome stability, ensures proper

replication dynamics, prevents transcription–replication conflicts, and limits chromosomal instability.

We further appreciate the insightful and pertinent queries raised by the Reviewer, which have significantly strengthened the quality and clarity of the revised manuscript.

Major Comments

1. A major shortcoming of this study is the failure to discuss the role of ATR, the kinase upstream of Chk1, considering that it is also found in their screen, and CHK1 is literally inactive without ATR. This can be easily visible in their Figure 4G, where phosphorylation of TOP1 is weak. Is this an ATR-CHK1-dependent process or only a CHK1-dependent process? This is essential to address.

Response: We thank the reviewer for raising a very pertinent as well as interesting question, one which had also intrigued the authors while conducting the study. As very correctly stated by the Reviewer, CHK1 is heavily dependent on ATR for activation upon replication stress or DNA damage. Since both ATR and CHK1 have emerged positively in our RADAR screen data, we considered the possibility that TOP1 might be directly and parallelly regulated by (i) ATR alone, (ii) ATR-CHK1 and/or (iii) CHK1 alone. To dissect the role of ATR and/or CHK1 in the regulation of TOP1cc, we have carried out several fresh experiments, as mentioned below:

(A) siRNA-mediated transient genetic depletion of CHK1 is sufficient to trap TOP1ccs on the genome, and CHK1*i* treatment under this condition does not induce further stabilization of TOP1ccs (Please refer to Fig. 1H of revised manuscript). This observation robustly demonstrates the direct involvement of CHK1 in regulation of TOP1 dynamics while ruling out a significant role of ATR alone in the same process.

(B) Interestingly, pharmacological ATR inhibition in the background of siCHK1 fails to enhance TOP1cc stabilization (Fig. 1H and Appendix Fig. S3I in the revised manuscript), which strongly suggests that TOP1cc trapping due to ATR inhibition seen in the RADAR screen is largely due to ATR-inhibition-associated loss of basal CHK1 activity.

Above two key observations strongly suggest a pivotal role of ATR-CHK1 axis in TOP1 dynamics and non-viability of other possibilities *i.e.,* ATR or CHK1 alone in the process.

As rigorously demonstrated by several groups, ATR is responsible for maintenance of basal cellular CHK1 activity even in the absence of exogenous replication stress (Saldivar *et*

al, 2018; Michelena *et al*, 2019; Moiseeva *et al*, 2019; Lebrec *et al*, 2022). When considered in conjunction with our observations, the authors believe that while ATR is absolutely indispensable of maintenance of basal CHK1 kinase activity in cells, CHK1 remains the "effector kinase" responsible for our observations with respect to the maintenance of TOP1 dynamics. Considering these observations, the authors have maintained throughout the manuscript that this is a CHK1-dependent pathway.

In order the clarify the position of ATR in the larger scheme of this pathway at cellular level, the authors have now included a discussion on the role of ATR in maintenance of basal CHK1 activity in the introduction section and discussed the newly reported data (with siCHK1) at length in the revised manuscript.

2. Additionally, some statements are not visible in their results. E.g. In Figure 3E, they claim that the CHK1 inhibitor does not affect the ubiquitination status, but I see that it does, as the ubiquitin signal/smear is strongly reduced after CPT and CHK1 inhibitor treatment when compared to CPT treatment alone. The quantification of results in several independent experiments will help them to draw their conclusion.

   Response: We thank the reviewer for the insightful observation and appreciate the concern. In order to address the same, we have reviewed our raw blots, and performed densitometric quantification and statistical analyses with the same. The quantitative analyses are now included in the manuscript (Fig. 3D). Upon statistical analyses of data from 3 replicates, we have established that CHK1*i* treatment does not significantly rescue CPT-induced TOP1cc ubiquitylation, in line with the observation reported in the original manuscript.

3. They also did not consider the fact that CHK1inh might affect TOP1ccs (local/chromatin fraction) degradation, but not global TOP1 degradation.

   Response: To the best of our understanding, the Reviewer has expressed a concern that the authors have not explored the possibility that CHK1*i* might influence only TOP1cc degradation from the genomic DNA but not global TOP1 degradation.

   We would like to bring it to the reviewer's attention that in the original manuscript, Fig. 3A addressed the possibility of the influence of CHK1*i* on global TOP1 degradation

using modified alkaline lysis assay, where cell extract was treated with Micrococcal nuclease and Benzonase. The data from this experiment clearly revealed that CHK1*i* treatment does not influence global TOP1 degradation.

In Fig. 3B, we had demonstrated using RADAR assay, that CHK1*i* pre-treatment does not prevent CPT-induced TOP1cc degradation from chromatin, thus negating the influence of CHK1*i* on local (chromatin fraction) TOP1 degradation. We apologize if the same was not described in sufficient detail in the original manuscript. We hope this addresses the concern raised by the reviewer.

4. Also, some aspects of chromosomal degradation of TOP1ccs are omitted from their investigation (Wss1/SPRTN; Cdc48 PMID: 24998930, PMID:32152270).

   Response: We thank the reviewer for pointing out the lapse with respect to the two vital TOP1cc-degradation pathways in the original manuscript. As suggested by the Reviewer, we have performed experiments involving siRNA-mediated silencing of SPRTN and pharmacological inhibition of p97 (CB-5083). Experiments performed under these conditions reveal that SPRTN and p97 do indeed have the capability to partially target CHK1*i*-induced TOP1ccs (Fig. 3I, J).

5. Also, the statement that we do not know how cells prevent Top1ccs under physiological conditions is not correct. Several papers show this. For instance: PMID: 32152270.

   Response: We apologize for the lapse on our part and thank the reviewer for pointing out the mistake in phrasing. We have made corresponding changes in the introduction section of our manuscript to incorporate the relevant literature.

6. The ectopically expressing TOP1 wild-type (TOP1wt) and TOP1 Ser320Ala (TOP1S320A) cell lines should be characterized in terms of TOP1 localization and cell cycle distribution and kinetics.

   Response: We thank the reviewer for the vital suggestion. We have now performed live cell imaging of cells expressing EGFP-TOP1^{WT} and EGFP-TOP1^{S320A}. Our results reveal nuclear localization of both variants, with a strong propensity of nucleolar accumulation (visualized through co-localization with mCherry-NPM1) (Fig. EV3C,D). We have also performed cell cycle analysis of U2-OS cells with stable ectopic expression of EGFP-

TOP1^{WT} and EGFP-TOP1^{S320A}. Our results reveal that ectopic expression EGFP-TOP1^{S320A} induces slightly longer S and G2/M phases of the cell cycle (Fig. EV3E,F).

Additional Comments

1. Introduction

(A) Relevant publications need to be cited when discussing the removal of TOP1cc (e.g., Wss1/SPRTN). (B) Also, when discussing the action of WRN on CHK1 activation, the author cites articles that do not describe the actual mechanism. A small paragraph about canonical CHK1 activation through polymerase-helicase uncoupling upon TOP1cc induction or fork stalling needs to be added. (C) What do the author mean by basal CHK1 activity? How this is activity regulated? I would assume that this is independent of long-stretches of ssDNA related to fork stalling. Please discuss non-canonical activation of CHK1 that is linked with DPCs and explain why such activity is relevant for normal physiological processes and how this is relevant to this manuscript.

Response: We thank the reviewer for the valuable suggestions.

(A) The relevant literature (which was cited without separate mention in the original manuscript) has now been briefly discussed in the revised manuscript.

(B) As suggested, canonical and TOP1cc-induced CHK1 activation have now been briefly discussed in the introduction section of the revised manuscript.

(C) The authors would like to clarify that basal CHK1 activity refers to the baseline, constitutive kinase activity of CHK1 which is maintained in cells during unperturbed growth even in the absence of replication stress, as demonstrated by several groups (Saldivar *et al*, 2018; Michelena *et al*, 2019; Moiseeva *et al*, 2019; Lebrec *et al*, 2022). The mechanism of ATR/CHK1 activation under unperturbed replication remains an actively investigated question. However, it is proposed that basal ATR/CHK1 activation may be initiated by short stretches of single stranded DNA generated by the action of the CMG helicase or at unligated Okazaki fragments. As highlighted in our study, basal level of CHK1 activation is necessary to regulate the TOP1 dynamics for facilitating the removal of topological constrains for smooth progression of replication forks and transcription machinery and maintaining genomic stability in response to inherently stable TOP1cc for longer time. A

short discussion of the same has now been included in the introduction section of the revised manuscript.

2. (A) Figure1: Better gel for 1E needed

(B) 1F Complementary Western blot (pATM, pKAP1 or pCHK2) is needed to demonstrate that the experimental condition does not lead to DSB formation. (D) 1 µM CPT is well known for DSB induction, which will activate ATM and DNA-PKcs. Demonstrate the same with 100 or 200 nM CPT instead. (C) It is important to supplement these (TOP1 mobility) data with ssDNA staining under non-denaturing conditions or by total RPA staining. It will help to clarify the status of the canonical signal for CHK1 activation i.e., ssDNA. Does inhibition of CHK1 lead to more ssDNA? There must be clarity.

Response: We thank the reviewer for these pertinent questions.

(A) As per the reviewer's suggestion, the western blot corresponding to Figure 1E (depicting TOP1 levels in cells treated with CHK1 inhibitors SCH and UCN-01) of the original manuscript has been repeated and has now been included in the revised manuscript as Appendix Fig. S3D.

(B) As suggested by the reviewer, we have performed western blot for phospho-KAP1 (S824) in U2-OS cells treated with 250 nM SCH for 6 h. Results Showed that that the experimental conditions do not induce DSBs. The data has been included in the revised manuscript (Appendix Fig. S3J). Further, we have also immunofluorescence-based detection of γH2AX levels under these conditions, which led us to further corroborate that CHK1 inhibition conditions employed in our experiments do not induce DNA damage in U2-OS cells (Appendix Fig. S4). The relevant findings have been described at length in the revised manuscript.

(C) As suggested by the reviewer, TOP1cc stabilization has been demonstrated with 100 nM CPT (1 h treatment). The data is included in Figure 1E in the revised manuscript.

(D) As per the reviewer's suggestion, we have treated U2-OS cells with SCH (250 nM, 6 h) and UCN-01 (50 nM, 6 h), followed by immunofluorescent detection of phospho-RPA (S33) and total RPA. Our results reveal that there is no significant induction of ssDNA as a result of CHK1 inhibition conditions employed in our study. In control experiment, treatment with CPT (100 nM) led to significant increase in phospho-RPA (S33) level, suggesting ssDNA

generation in the CPT treatment. The data is included in the revised manuscript (Appendix Fig. S5).

3. Figure 2, C-H instead of values at the bottom of the gels, the authors must provide line graphs which are easy to read.

   Response: As per the reviewer's suggestion, quantification values provided in the original manuscript have now been replaced with line graphs and statistical analyses.

4. (A) Figure 3 A-B Explanation regarding the differences in methodology with respect to alkaline lysis and RADAR assay will provide more clarity to the general readers.

   (B) What is the justification for using 10 and 25 μM CPT. A Western blot is needed to show the status of pATM, pKAP1 and pCHK2 under the same conditions.

   (C) C-E a schematic of the assay will help the general readers about the experimental setup.

   (D) G High concentration of CPT is used. It will be ideal to do a Western analyses with all CPT conditions (dose and time) and demonstrate that it does not induce DSBs or activate associated kinases.

   Response:

   (A) We thank the reviewer for the valuable suggestion for facilitation of readability for general readers. As suggested, we have included a schematic workflow of the alkaline lysis method in the revised manuscript (Appendix Figure S6F). Schematic workflow of RADAR assay was presented in Figure 1A of original manuscript. The same has been retained in the revised manuscript.

   (B) Cellular TOP1cc levels are maintained through a fine balance between TOP1cc formation (a characteristic of the TOP1 catalytic cycle or orchestrated by the presence of a drug such as CPT) and TOP1cc degradation (either drug-induced degradation or basal degradative pathways such as SPRTN). In the original manuscript, the experimental question we attempted to answer in Fig. 3 was whether CHK1 regulates cellular TOP1/TOP1cc degradation machinery, resulting in CHK1*i*-mediated TOP1cc stabilization on the genome. In order to do that, we took advantage of the fact that CPT-mediated TOP1cc stabilization is followed by rapid TOP1cc degradation and global TOP1 downregulation. Hence, we hypothesized that if CHK1 indeed regulates the cellular TOP1cc/TOP1 degradation machinery, then CHK1*i* should interfere with degradation of TOP1ccs stabilized by CPT.

Hence, in Fig. 3, CPT treatment was solely used as a model system for triggering TOP1 degradation. The concentration used in these experiments were chosen based on the standard concentrations used for triggering TOP1 degradation in several reports from different laboratories (Sun *et al*, 2020; Lin *et al*, 2009; Desai *et al*, 1997; Sun *et al*, 2021; Zhang *et al*, 2022). The authors would like to introduce a gentle clarification that CPT-induced DNA damage (which is copiously induced at these concentrations as widely reported by us and others (Guha Majumdar *et al*, 2023) or activation of associated kinases, is unlikely to have a bearing on our experimental analysis, as CPT was merely used a standard tool for inducing TOP1 degradation.

(C) As suggested by the reviewer, schematic workflows have now been provided alongside the relevant figures in the revised manuscript.

(D) The authors request the reviewer to please refer to the clarification provided above in (B), concerning the usage of micromolar concentrations of CPT.

5. Figure 5D Ideally, these experiments should have been performed after depletion of endogenous TOP1. Line graphs have to be provided.

Response: We thank the reviewer for the valuable suggestion. As suggested, we have performed siRNA-mediated depletion of TOP1, followed by ectopic expression of TOP1 mutants. Specific siRNA is used to target towards 3'UTR of human TOP1, which principally depletes endogenous TOP1 but not ectopically expressing TOP1 (without 3'UTR). The new data along with the corresponding graph and statistical analyses has now been included in the revised manuscript (Fig. 5D).

6. Figure 6

(A) Data presented with different font sizes is difficult to read. The authors must fix these, which are common throughout the manuscript and present these figures in a clear and consistent manner. The concentration of plasmid in the relaxation assay is missing. (B) Please comment on why Proteinase K has not been used before running the reaction products on a gel. (C) In the methodology, please mention why post-staining is performed for the general readers.

Response: (A) We apologize for this issue in the original manuscript. As suggested, font sizes are enhanced in all the figures for better readability. The concentration of plasmid

used for plasmid relaxation assays (200 ng/reaction) has now been incorporated in the methods section of the revised manuscript.

(B) Plasmid relaxation assays were performed as per standard protocols reported elsewhere (Nitiss *et al*, 2012, 2021). Proteinase K digestion is not performed in case of plasmid relaxation assay as a standard practice due to the following factors:

i. Since *in vitro* plasmid relaxation assay is an equilibrium assay (and not a suicide assay such as the cleavage and religation assays) which measures the final products of relaxation of supercoiled DNA by TOP1 and does not take into account the population of TOP1ccs, Proteinase K digestion is not performed to release TOP1cc-bound plasmids. However, Proteinase K digestion is performed in TOP1 cleavage and religation assays to release TOP1cc-bound intermediates.

ii. Since products of plasmid relaxation assay are resolved on agarose gels with significantly larger pore size (and hence the ability to accommodate large complexes) compared to urea-PAGE gels (used for cleavage and religation assays), the TOP1cc-bound plasmids are likely to participate in the electrophoretic process and migrate in sync with the nicked plasmid fraction (owing to the negligible molecular mass of TOP1 as compared to the large mass of plasmid DNA bound to TOP1).

(C) We thank the reviewer for the valuable suggestion. The reason for using post-staining (*"Since EtBr induces positive supercoiling in covalently closed circular DNA, post-staining was performed in order to avoid EtBr-induced topological perturbations in TOP1-relaxed plasmid intermediates."*) has now been included in the methods section of the revised manuscript.

7. Figure 7

(A) A Western blot for stable expression is needed. (B) Font sizes need to be uniform

(C) High-resolution images for metaphase chromosomes are needed.

Response: We thank the reviewer for the suggestions.

(A) As suggested by the reviewer, a western blot for stable expression of EGFP-TOP1^{WT} and EGFP-TOP1^{S320A} was been included in the revised manuscript (Fig. EV3A).

(B) As per the reviewer's suggestion, font sizes have been made uniform in Fig. 7.

(C) We acknowledge the lack of clarity in the metaphase spread photomicrographs in the original manuscript. Hence, the images have been replaced with two larger, high

resolution metaphase spreads (one each for EGFP-TOP1^{WT} and EGFP-TOP1^{S320A}) in the revised manuscript (Fig. 7J).

8. Figure 8

A-B does not say anything about DSB; please stain DSB markers (53BP1) or rephrase.

Response: We thank the reviewer for pointing out the lapse on our part. The relevant section has been rephrased to "DNA damage" in place of "DSB".

9. Figure 9

A How do you rule out DSB induction and ATM activation under 2 µM CPT for 2hrs. The appearance of DSBs will change the overall interpretation.

Response: We thank the reviewer for the pertinent question. The authors would like to clarify that the aim of the experiment in Fig. 9 was to evaluate the effect of CHK1*i* on genome-wide transcription vis-à-vis gene length. In this experiment, CPT was only used as a positive control to recapitulate the observation already reported by other groups (Veloso *et al*, 2013; Baranello *et al*, 2016). This was solely done in order to establish the validity of our experimental protocol. The mechanistic basis for the selective suppression of long genes by CPT has been intensely worked out (Veloso *et al*, 2013; Baranello *et al*, 2016), and hence, this experimental condition was solely used as a positive control.

Additional unclear points throughout the manuscript:

1. In supplementary Figure 10 A, it appears that the GFP signal indicating the expression of S320A is not present in all cells. Corresponding IF experiments (Fig. 7 A, D) should include GPF signal and non-single cell assays, like the fibre assay could be used following FACS sorting of GFP-positive cells.

Response: We thank the reviewer for the insight, and we apologize for the lack of clarity of the image which was provided earlier. As per our microscopic analysis, 100% of U2-OS$^{EGFP\text{-}TOP1\ WT}$ and U2-OS$^{EGFP\text{-}TOP1\ S320A}$ cells have EGFP expression. In order to improve clarity, Supplementary Figure 10 A has been replaced with an image captured at 63X magnification (as opposed to 20x magnification provided earlier). Additionally, EGFP fluorescence

quantification has been performed across 500 cells each to demonstrate equivalent EGFP expression in U2-OS$^{EGFP\text{-}TOP1\ WT}$ and U2-OS$^{EGFP\text{-}TOP1\ S320A}$ cells (Appendix Fig. 10A).

In our experimental system, we have observed loss of EGFP signal upon fixation and subsequent immunofluorescence staining protocol. However, due to single colony selection and EGFP expression across 100% cells, EGFP signal acquisition was not attempted in experiments pertaining to Fig. 7A, D.

We thank the reviewer for the valuable suggestion. In single molecule DNA fiber analysis, cells were trypsinized, lysed on glass slides, followed by gravity-assisted DNA fiber spreading. Since cells are not fixed during the workflow (which would prevent fiber spreading), FACS sorting was not possible to perform in order to avoid replication-fork perturbations during the sorting process. However, we performed microscopy-assisted estimation of transfection efficiency (10 fields covering a minimum of 300 cells per sample), whereby ~80 % cells were found to be positive for EGFP signal (Appendix Fig. S11). In addition, during acquisition of DNA fibers, analysis was performed on spatially distant fibers in order to eliminate signal bias from untransfected cells.

2. In the experiments involving ectopic and transient expression of TOP1 wild-type (TOP1wt) and S320A further evidence of expression in multiple cells is required.

   Response: In order to provide evidence for ectopic expression across multiple cells in experiments performed in Figure 6 and Figure 7, we have provided microscopy images for EGFP expression (Appendix Fig. S9 and Appendix Fig. S11, respectively).

3. Clearly indicate the number of events/cells analyzed per condition and experiment (Fig. 4E, F, Fig. 5E, F, Fig. 7A-C, Fig. 8A, B).

   Response: As suggested by the Reviewer, the number of cells analysed in single cell experiments has now been described in the corresponding figure legends in the revised manuscript.

4. Figure 3H is confusing. The effect of CtIP on intensity of TOP1cc foci in dotblot (3H) does not correspond to the quantification graph (3I).

Response: We apologize for the quality of the blot in the original manuscript. The dot blot with siCTIP has now been replaced with another replicate which better represents the quantification data (Fig. 3I).

5. The entire set of experiment is performed in cancer cell lines (U2OS, PANC-1, A549, HCT116, and MDA-MB-231). To substantiate the claim that the observed effects are not cell type-specific, it is essential to demonstrate them in non-transformed human cell lines.

Response: We thank the reviewer for this observation and appreciate the concern. In the original manuscript, CHK1*i*-induced TOP1cc stabilization was demonstrated in primary mouse splenic cells. However, in order to replicate the effect in non-transformed human cells, we have performed the experiment in WI-38 cells. Our results with WI-38 (human fetal lung) (Appendix Fig. S3G) corroborate our earlier findings presented in the original manuscript.

6. While the study focuses on phospho-resistant mutants, it would be equally informative to explore the complementary approach of constitutive phosphorylation. For instance, employing a phospho-mimetic mutant such as TOP1S320E could provide valuable insights into TOP1 mobility in FRAP, catalytic activity (religation assay), and genome stability.

Response: We thank the reviewer for the valuable suggestion. While conducting the study, we generated $TOP1^{S320D}$ as well as $TOP1^{S320E}$ as candidate phosphomimetic variants. However, we did not obtain encouraging results with either of these mutants. A careful review of literature revealed that several groups have reported that phosphomimetic mutants often fail to faithfully recapitulate the effect of phosphorylation events. These effects often stem from failure of cofactors to bind to the mutated site, or the non-equivalence of negative charge of a phosphate group (typically -1.5) and aspartate or glutamate (-1) at physiological pH (Dephoure *et al*, 2013; Pérez-Mejías *et al*, 2020). For example, the effect of phosphomimetic mutants in 14-3-3ζ has been intensely studied. Kozeleková and coworkers demonstrated that S58D/E phosphomimetics fail to recapitulate S58 phosphorylation in 14-3-3ζ due to adverse effects on these mutations on the oligomerization status and subcellular localization of the protein (Kozeleková *et al*, 2022). Similarly Somale and coworkers have reported the failure of activation loop

phosphomimetic mutants of RSK to produce constitutive activation of RSK due to failure in forming a critical salt bridge required for activation (Somale *et al*, 2020). In another study, Chino and coworkers demonstrated that CK2-mediated phosphorylation on the autophagy receptor TEX264 cannot be phenotypically recapitulated by phosphomimetic mutations, due to the failure to form multiple hydrogen bonds which are involved in the interaction between phosphorylated TEX264 and ATG8 (Chino *et al*, 2022). These and several other studies (Rrustemi *et al*, 2024; Ng *et al*, 2010) have led us to believe that TOP1 S320 phosphorylation is likely to induce unique alterations in the relative mobility between the α5 and α6 "nose-cone" helices (discussed in detail in the original manuscript), which may be dependent on ionic interactions which cannot be faithfully recapitulated by aspartate or glutamate at physiological pH.

Minor Comments

1. In Figure 1 legend "At least 200 nuclei were scored per sample." Is repeated.

Response: As suggested by the reviewer, the repetition has been corrected in the revised manuscript.

2. TOP1ccs stands for Topoisomerase 1 cleavage complexes, not for the TOP1-DNA covalent complex, although this is indeed a TOP1-DNA covalent complex. (Page 1, line 10 and 11)

Response: We thank the reviewer for the suggestion. We have incorporated the suggested change in the revised manuscript.

3. (Page 17, lines 396 - 400) The possibility of cofactors recognizing the phosphate group on TOP1 and initiating degradation is not mentioned in the discussion; only conformational differences are referenced.

Response: We thank the reviewer for the insightful question. The authors would like to clarify that the authors propose that phosphorylation of S320 of TOP1 affects its interaction with the DNA substrate. This proposition is based on available crystal structure data (which shows intimate interactions between the "nose-cone" helices around S320 and the DNA substrate), and several molecular dynamics simulations which demonstrate a critical role of the relative mobility of these helices in determining the interaction of TOP1 with its substrate. We would like to draw the reviewer's attention to the following excerpt from the

discussion section of the original manuscript:

"Recent theoretical as well as molecular dynamics (MD) simulation-based calculations have highlighted the critical impact of serine phosphorylation on bending propensities of protein backbones. Given its strategic positioning at the loop between the α5 and α6 "nose-cone" helices, phosphorylation of S320 might be implicated in their relative movement, and in turn, their interaction with the DNA substrate. The indispensable role played by the relative dynamics of the "nose-cone" helices has been evidenced by localized non-isomorphism between multiple crystal structures in this region, and theoretically predicted by MD simulations. These studies provide a putative structural basis for our observations."

The experimental observations made by the authors do not suggest an apparent possibility of cofactors recognizing the phosphate group on S320 and initiating degradation of TOP1 or TOP1ccs. This is backed by the fact that CHK1 inhibition does not alter global TOP1 levels (Appendix Fig. S3D). Further, results reported in Fig. 3 strongly suggest that while CHK1*i*-stabilized TOP1ccs may be partially targeted by CTIP, SPRTN and p97, CHK1-mediated phosphorylation itself may not play a prominent role in degradation of TOP1 or TOP1ccs (Fig. 3A-D). Collectively, our results point towards a direct role played by CHK1-mediated phosphorylation of TOP1 S320 in regulation of TOP1 catalytic cycle (Figure 6 and Supplementary Figure 9). Hence, the relevant possibilities which are supported by our experimental observations have been discussed in the manuscript.

4. Throughout the Materials and Methods section, time units are inconsistently abbreviated (e.g., minutes/min, hours/h). Ensure consistency.

Response: We thank the reviewer for the suggestion. We have revised the methods section and ensured consistency in time units (h, min and s).

5. Figure 8

(C) R-loops in the cytoplasm with RNase H (-) may need a different image;

(F) a clearer image for wild-type and RNase H (-) would enhance clarity. The difference in density is not as strong as suggested by the statistics provided.

Response: (C) In order to address the reviewer's concern, the entire panel pertaining to Fig. 8C has been replaced with clearer images.

(F) As per the reviewer's suggestion, Figure 8F has been replaced with a fresh dot blot, and the corresponding statistical analysis has been provided alongside.

Remove "250 nM" behind CHK1i in Fig. 3G.

Response: The necessary change has been incorporated as per the reviewer's suggestion.

Fig. 2B the figure legend indicates that cells were treated with CHKi1 for 4 and 6h, figure itself says 2 and 4h.

Response: We apologize for the lapse in the original manuscript. For the experiment, cells were treated for 4 and 6 h, which has now been corrected in the revised manuscript.

9. Typos

1. Page 2, Line 927: ... formation of transient a TOP1-DNA covalent complex...
2. Page 20, Line 489: ... various therapeutics cannot not be attributed...
3. Page 34, Line 817: ... periodic voxtexing...

Response: The authors apologize for the typos in the original manuscript. The whole manuscript has now been proofread, and typos (including the ones pointed out by the reviewer) have been resolved in the revised manuscript.

Referee #3

In this manuscript by Majumdar et al, the authors identify TOP1 Serine-320 phosphorylation by CHK1 as a regulatory mechanism that controls TOP1 re-ligation and maintains steady state TOP1cc levels in the cells. Furthermore, the authors show that the ectopic overexpression on the phospho-mutant impairs replication and results in genome instability. The authors suggest that impairment of this mechanism results fork slowdown and also replication transcription conflicts. This study is well done, and the authors have used multiple different approaches to demonstrate the phenomenon and critical role of this phosphorylation of TOP1 in maintain genome stability. I have few suggestions which I think would make the conclusions even more robust.

Response: We thank the Reviewer for acknowledging multiple different approaches employed in the manuscript to demonstrate the phenomenon and critical role of this

phosphorylation of TOP1 in maintain genome stability. We are also grateful for his/her insightful comments for the improving the manuscript. We have tried our best to address his/her queries diligently, as mentioned below.

Comments:

1. The authors have used a constitutive overexpression system of the TOP1 phospho-mutant for all their experiments. I propose that they perform the most critical experiments with an siRNA for TOP1 to downregulate endogenous TOP1 and then use a siRNA resistant phospho-mutant overexpression.

   Response: We thank the reviewer for the valuable suggestion. As suggested by the Reviewer, we have performed siRNA-mediated depletion of TOP1, followed by ectopic expression of TOP1 mutants. Specific siRNA is used to target towards 3'UTR of human TOP1, which principally depletes endogenous TOP1 but not ectopically expressing TOP1 (without 3'UTR). The new data along with the corresponding graph and statistical analyses has now been included in the revised manuscript (Fig. 5D).

2. If the model that the authors propose were to be true, i.e. TOP1 cc mediated fork slowdown and transcription inhibition caused replication transcription conflicts, (A) transient inhibition of transcription or RNAaseH overexpression should be able to rescue the genome instability that they observed upon phospho-mutant overexpression. (B) The authors should be careful about the transient transcription inhibitors as they can also cause cell cycle arrest and should include controls to demonstrate that transcription is inhibited and not the replication process. (C) Furthermore the authors should also show that RNAase H overexpression in these contexts also reduces R-loop formation.

   Response: We thank the reviewer for raising these very pertinent questions. In order to address these, we have performed the following experiments as suggested:

   (A) We have employed DRB-mediated transient transcription inhibition in U2-OS cells stably expressing EGFP-TOP1^{S320A}, followed by preparation of metaphase spreads. Interestingly, transient inhibition of transcription for 16 h does indeed result in partial yet statistically significant rescue of genomic instability (Fig. 8J, K), thus supporting the Reviewer's interpretation.

(B) In order to rule out DRB-mediated replication perturbations, we measured EdU incorporation in U2-OS cells expressing EGFP-TOP1^{S320A} post DRB treatment for 8 or 16 h. Our results (Supplementary Figure 13A, B) reveal that under our experimental conditions, DRB treatment does not introduce significant replication perturbations.

(C) Since we have used the transient transcription inhibition model, we have employed dot blot to measure R-loop levels in U2-OS cells expressing EGFP-TOP1^{S320A} in response to DRB treatment. Our results revealed that DRB treatment for 16 h does indeed result in significant reduction in R-loop levels (Appendix Fig. S13C, D).

References

Baranello L, Wojtowicz D, Cui K, Devaiah BN, Chung H-J, Chan-Salis KY, Guha R, Wilson K, Zhang X, Zhang H, *et al* (2016) RNA Polymerase II Regulates Topoisomerase 1 Activity to Favor Efficient Transcription. *Cell* 165: 357–371

Chino H, Yamasaki A, Ode KL, Ueda HR, Noda NN & Mizushima N (2022) Phosphorylation by casein kinase 2 enhances the interaction between ER-phagy receptor TEX264 and ATG8 proteins. *EMBO Rep* 23: EMBR202254801

Dephoure N, Gould KL, Gygi SP & Kellogg DR (2013) Mapping and analysis of phosphorylation sites: a quick guide for cell biologists. *MBoC* 24: 535–542

Desai SD, Liu LF, Vazquez-Abad D & D'Arpa P (1997) Ubiquitin-dependent Destruction of Topoisomerase I Is Stimulated by the Antitumor Drug Camptothecin *. *Journal of Biological Chemistry* 272: 24159–24164

Guha Majumdar A, Shree S, Das A, Kumar BK, Dey P, Subramanian M & Patro BS (2023) Design, synthesis and development of a dual inhibitor of Topoisomerase 1 and poly (ADP-ribose) polymerase 1 for efficient killing of cancer cells. *European Journal of Medicinal Chemistry* 258: 115598

Kozeleková A, Náplavová A, Brom T, Gašparik N, Šimek J, Houser J & Hritz J (2022) Phosphorylated and Phosphomimicking Variants May Differ—A Case Study of 14-3-3 Protein. *Front Chem* 10

Lebrec V, Poteau M, Morretton J-P & Gavet O (2022) Chk1 dynamics in G2 phase upon replication stress predict daughter cell outcome. *Developmental Cell* 57: 638-653.e5

Lin C-P, Ban Y, Lyu YL, Desai SD & Liu LF (2008) A Ubiquitin-Proteasome Pathway for the Repair of Topoisomerase I-DNA Covalent Complexes. *Journal of Biological Chemistry* 283: 21074–21083

Lin C-P, Ban Y, Lyu YL & Liu LF (2009) Proteasome-dependent processing of topoisomerase I-DNA adducts into DNA double strand breaks at arrested replication forks. *J Biol Chem* 284: 28084–28092

Michelena J, Gatti M, Teloni F, Imhof R & Altmeyer M (2019) Basal CHK1 activity safeguards its stability to maintain intrinsic S-phase checkpoint functions. *J Cell Biol* 218: 2865–2875

Moiseeva TN, Yin Y, Calderon MJ, Qian C, Schamus-Haynes S, Sugitani N, Osmanbeyoglu HU, Rothenberg E, Watkins SC & Bakkenist CJ (2019) An ATR and CHK1 kinase signaling mechanism that limits origin firing during unperturbed DNA replication. *Proc Natl Acad Sci USA* 116: 13374–13383

Ng Y-W, Raghunathan D, Chan PM, Baskaran Y, Smith DJ, Lee C-H, Verma C & Manser E (2010) Why an A-Loop Phospho-Mimetic Fails to Activate PAK1: Understanding an Inaccessible Kinase State by Molecular Dynamics Simulations. *Structure* 18: 879–890

Nitiss JL, Kiianitsa K, Sun Y, Nitiss KC & Maizels N (2021) Topoisomerase Assays. *Curr Protoc* 1: e250

Nitiss JL, Soans E, Rogojina A, Seth A & Mishina M (2012) Topoisomerase Assays. *Curr Protoc Pharmacol* CHAPTER: Unit3.3

Pérez-Mejías G, Velázquez-Cruz A, Guerra-Castellano A, Baños-Jaime B, Díaz-Quintana A, González-Arzola K, Ángel De la Rosa M & Díaz-Moreno I (2020) Exploring protein phosphorylation by combining computational approaches and biochemical methods. *Comput Struct Biotechnol J* 18: 1852–1863

Rrustemi T, Meyer K, Roske Y, Uyar B, Akalin A, Imami K, Ishihama Y, Daumke O & Selbach M (2024) Pathogenic mutations of human phosphorylation sites affect protein–protein interactions. *Nat Commun* 15: 3146

Saldivar JC, Hamperl S, Bocek MJ, Chung M, Bass TE, Cisneros-Soberanis F, Samejima K, Xie L, Paulson JR, Earnshaw WC, *et al* (2018) An intrinsic S/G2 checkpoint enforced by ATR. *Science* 361: 806–810

Somale D, Di Nardo G, di Blasio L, Puliafito A, Vara-Messler M, Chiaverina G, Palmiero M, Monica V, Gilardi G, Primo L, *et al* (2020) Activation of RSK by phosphomimetic substitution in the activation loop is prevented by structural constraints. *Sci Rep* 10: 591

Sun Y, Chen J, Huang SN, Su YP, Wang W, Agama K, Saha S, Jenkins LM, Pascal JM & Pommier Y (2021) PARylation prevents the proteasomal degradation of topoisomerase I DNA-protein crosslinks and induces their deubiquitylation. *Nat Commun* 12: 5010

Sun Y, Miller Jenkins LM, Su YP, Nitiss KC, Nitiss JL & Pommier Y (2020) A conserved SUMO pathway repairs topoisomerase DNA-protein cross-links by engaging ubiquitin-mediated proteasomal degradation. *Sci Adv* 6: eaba6290

Veloso A, Biewen B, Paulsen MT, Berg N, Lima LC de A, Prasad J, Bedi K, Magnuson B, Wilson TE & Ljungman M (2013) Genome-Wide Transcriptional Effects of the Anti-Cancer Agent Camptothecin. *PLOS ONE* 8: e78190

Zhang H, Xiong Y, Su D, Wang C, Srivastava M, Tang M, Feng X, Huang M, Chen Z & Chen J (2022) TDP1-independent pathways in the process and repair of TOP1-induced DNA damage. *Nat Commun* 13: 4240

Prof. Birija Sankar Patro
Bhabha Atomic Research Centre
Bio-Organic Division
Mod. Lab
Trombay
Mumbai, Maharashtra 400085
India

2nd Mar 2026

Re: EMBOJ-2025-122198R
A CHK1-orchestrated phosphorylation switch suppresses Topoisomerase 1-associated genomic instability

Dear Dr. Patro,

Thank you for submitting your revised manuscript on TOP1 regulation by Chk1 to The EMBO Journal. Two of the original referees have now assessed it once more, and were overall satisfied with the revisions, with only some minor text changes still suggested by referee 2.
Before proceeding further with the manuscript, we however need to also clarify several issues that came up during our routine pre-acceptance image checks. From our analyses, it looks like some duplicate images are displayed between Figure 1H and Figures 3I and 5D - despite being labelled as showing distinct conditions. We will require conclusive clarification of these issues, including provision of supporting source data, and careful revision of these figures as applicable.

In addition, there are also a number of other editorial points that would still need to be addressed at this stage:

- Please adjust the order of the manuscript sections, and also make sure to use the correct section headers:
Single title page with complete author information, Abstract, Introduction, Results, Discussion, Methods, Data Availability, Acknowledgements, Disclosure and Competing Interests Statement, References, Main Figure Legends, Tables, Expanded Figure Legends.

- I would suggest making the title more explicit and clear by replacing the vague term '-orchestrated' with something like '-mediated' or '-dependent'.

- As we are switching from a free-text author contribution statement towards a more formal statement based on Contributor Role Taxonomy (CRediT) terms, please remove the present Author Contribution section and instead specify each author's contribution(s) directly in the Author Information page of our submission system during upload of the final manuscript. See https://casrai.org/credit/ for more information.

- Please carefully go through the reference list and make sure that all references have their complete citation information - including year, journal name & volume, and page/elocator numbers - such information is currently missing for several of them. Also, please adjust the format for citation of preprints as specified in our author guidelines:
The citation in the text should be: "(preprint: [author name1] et al, [year])"
The citation in the reference list: "[author name1], [author name2], ... , [author name10] (et al), [year] [article title]. bioRxiv doi: [doi xxx] (preprint)"

- Please make sure to fully complete our author checklist, as responses in the header and several other sections are still missing.

- Please move all funding information (currently in a separate section) into the Acknowledgements section, and make sure that it is fully congruent with the information entered into the submission system.

- Please provide suggestions for a short 'blurb' text prefacing and summing up the conceptual aspect of the study in two sentences (max. 250 characters), followed by 3-5 one-sentence 'bullet points' with brief factual statements of key results of the paper; they will form the basis of an editor-written 'Synopsis' accompanying the online version of the article. Please also upload a synopsis image, which can be used as a "visual title" for the synopsis section of your paper. The image should be ideally in JPG format, and please make sure that it remains in the modest dimensions of (exactly) 550 pixels wide and between 300-500 pixels high.

- Thank you for providing the required Source Data. Unfortunately, we would require this data to be split up by figure panel, instead of numerical data for several panels being combined into a single XLSX file. Instead, rather combine numerical and graphical data for a given panel in one subfolder each. Also, please double-check that all Source Data listed in the Source Data

checklist are included (e.g., the mentioned SD for Fig 1J does not seem to be present).

- Please provide the Appendix as PDF, and make sure to insert a page break before each figure/table item. Also, please carefully double-check the contents, there seem to be 13 Appendix figures, but only 12 are listed on its title page, while 15 Appendix figures are referenced in the manuscript text.

- Please update the format of the "Data Availability" section, which should only mention data deposited in external repositories. Please include a general access link to each of the utilized databases (e.g. PRIDE), plus the respective accession codes for the various datasets. Please also remove referee access information now, and ensure that all datasets become publicly accessible at this point.

- During routine pre-acceptance checks, our data editors have raised the following queries regarding figures, data, and legends; I would appreciate if you briefly answered to them in the cover letter of your final submission, and made the requested text modifications with changes/additions highlighted via the "Track changes" option, to facilitate our final checking":
1. Please note that n=2 in figures 6I, K, 7I (clarify with them!); 9A, C, D; EV2 B, D (not applicable!)
2. Although 'n' is provided, please describe the nature of entity for 'n' in the legends of figures 2D, F, H; 3D, H, J; 6I, K
3. Please note that the exact p values are not provided in the legends of figures 1F, K, L; 3J; 4F, 5G, I; 6B, D, F, K; 7B, C, E, H, I, K; 8B, D, G, I, K; EV1 E, F; EV2 B, D; EV3 F, H, J.
4. Please note that the dashed borders are not defined in the legend of figure 7A, 8A, H; EV2 C, EV3 B, C. This needs to be rectified.
5. Please note that the red dotted squares are not defined in the legend of figure 8J. This needs to be rectified.

I am therefore returning the manuscript to you once more for revision, hoping that you will be able to satisfactorily address these remaining issues. Please do not hesitate to contact me should you have any questions in this regard.

With kind regards,

Hartmut

Hartmut Vodermaier, PhD
Senior Editor, The EMBO Journal
h.vodermaier@embojournal.org

*** PLEASE NOTE: All revised manuscript are subject to initial checks for completeness and adherence to our formatting guidelines. Revisions may be returned to the authors and delayed in their editorial re-evaluation if they fail to comply to the following requirements. As a first step please read our guidelines for revised submissions:
https://link.springer.com/journal/44318/submission-guidelines#cms-Revised-submissions

1) Every manuscript requires a Data Availability section (even if only stating that no deposited datasets are included). Primary datasets or computer code produced in the current study have to be deposited in appropriate public repositories prior to resubmission, and reviewer access details provided in case that public access is not yet allowed.

2) Each figure legend must specify
- size of the scale bars that are mandatory for all micrograph panels
- the statistical test used to generate error bars and P-values
- the type error bars (e.g., S.E.M., S.D.)
- the number (n) and nature (biological or technical replicate) of independent experiments underlying each data point
- Figures may not include error bars for experiments with n<3; scatter plots showing individual data points should be used instead.

3) Revised manuscript text (including main tables, and figure legends for main and EV figures) has to be submitted as editable text file (e.g., .docx format). We encourage highlighting of changes (e.g., via text color) for the referees' reference.

4) Each main and each Expanded View (EV) figure should be uploaded as individual production-quality files (preferably in .eps, .tif, .jpg formats). For suggestions on figure preparation/layout, please refer to our Figure Preparation Guidelines:
https://media.springernature.com/original/springer-cms/rest/v1/content/27825798/data/v1

5) Point-by-point response letters should include the original referee comments in full together with your detailed responses to them (and to specific editor requests if applicable), and also be uploaded as editable (e.g., .docx) text files.

6) Please complete our Author Checklist, and make sure that information entered into the checklist is also reflected in the

manuscript; the checklist will be available to readers as part of the Review Process File.

7) All authors listed as (co-)corresponding need to deposit, in their respective author profiles in our submission system, a unique ORCiD identifier linked to their name. Please see our Guide to Authors for detailed instructions.

8) Please note that supplementary information at EMBO Press has been superseded by the 'Expanded View' for inclusion of additional figures, tables, movies or datasets; with up to five EV Figures being typeset and directly accessible in the HTML version of the article.

9) To facilitate reproducibilty and cross-laboratory adoption of methodologies, please structure the Materials & Methods section as outlined in our guide to authors, including a completed Reagents and Tools Table.

10) Digital image enhancement is acceptable practice, as long as it accurately represents the original data and conforms to community standards. If a figure has been subjected to significant electronic manipulation, this must be clearly noted in the figure legend and/or the 'Materials and Methods' section. The editors reserve the right to request original versions of figures and the original images that were used to assemble the figure. Finally, we generally encourage uploading of numerical as well as gel/blot image source data.

At EMBO Press we ask authors to provide source data for the main manuscript figures. You will receive a separate email with instructions for providing source data with your revised manuscript, including how to upload and organize the files.

In the interest of ensuring the conceptual advance provided by the work, we recommend submitting a revision within 3 months (31st May 2026). Please discuss the revision progress ahead of this time with the editor if you require more time to complete the revisions. Use the link below to submit your revision:

Link Not Available

Referee #1:

The authors have thoroughly addressed all my comments. The revised manuscript is improved and of significant interest to a broad scientific audience.

Referee #2:

The revised manuscript has been improved, and the authors have addressed the majority of my comments.

They have now experimentally confirmed and discussed the previous finding that p97-SPRTN regulates steady-state TOP1ccs under unperturbed conditions. However, although they provide an extensive discussion, they do not consider the possibility that SPRTN may also indirectly regulate TOP1cc persistence by directly regulating CHK1 activation under unperturbed conditions (PMID: 31316063).

In the abstract, they state that the mechanism of TOP1cc regulation escapes proteasomal control: "We further demonstrate a distinct mechanism of TOP1cc stabilization which escapes recognition by the proteasomal, nuclease, and autophagic machineries." However, the persistence of TOP1ccs under CHK1 inhibition is heavily regulated by CtIP, SPRTN, and p97 (Fig. 3I, J). This point should be emphasized more clearly in the manuscript, as these proteins very likely regulate TOP1cc removal via protease activity (SPRTN), segregase activity (p97), or nuclease activity (CtIP) following CHK1 S320 phosphorylation (Fig. 3I, J). This should also be incorporated into their model (Fig. 9E).

==

Point-by-point Response to Referees

Referee #1:

The authors have thoroughly addressed all my comments. The revised manuscript is improved and of significant interest to a broad scientific audience.

Response: We thank the Referee for his/her insightful comments to improve the manuscript.

Referee #2:

The revised manuscript has been improved, and the authors have addressed the majority of my comments.

Response: We thank the Referee for his/her insightful comments to improve the manuscript.

1. They have now experimentally confirmed and discussed the previous finding that p97-SPRTN regulates steady-state TOP1ccs under unperturbed conditions. However, although they provide an extensive discussion, they do not consider the possibility that SPRTN may also indirectly regulate TOP1cc persistence by directly regulating CHK1 activation under unperturbed conditions (PMID: 31316063).

Response: We thank the reviewer for suggesting a pertinent and intriguing possibility. We have now included a reference to the same in the discussion section of the revised manuscript.

2. In the abstract, they state that the mechanism of TOP1cc regulation escapes proteasomal control: "We further demonstrate a distinct mechanism of TOP1cc stabilization which escapes recognition by the proteasomal, nuclease, and autophagic machineries." However, the persistence of TOP1ccs under CHK1 inhibition is heavily regulated by CtIP, SPRTN, and p97 (Fig. 3I, J). This point should be emphasized more clearly in the manuscript, as these proteins very likely regulate TOP1cc removal via protease activity (SPRTN), segregase activity (p97), or nuclease activity (CtIP) following CHK1 S320 phosphorylation (Fig. 3I,J). This should also be incorporated into their model (Fig. 9E).

Response: We thank the reviewer for the insightful suggestion. We have now modified the abstract in the revised manuscript to include the targeting of CHK1*i*-stabilized TOP1ccs by CtIP, SPRTN and p97 and also discussed in the manuscript. Further, the model has been updated in a commensurate manner.

Prof. Birija Sankar Patro
Bhabha Atomic Research Centre
India

30th Mar 2026

Re: EMBOJ-2025-122198R1
A CHK1-mediated phosphorylation switch suppresses human Topoisomerase 1-associated genomic instability

Dear Dr. Patro,

Thank you for submitting your re-revised manuscript for our consideration. After carefully going through all final changes, I unfortunately noticed a few remaining issues that will require further modifications:

- For various figure panels, statistic representations have been applied to samples where N<3, and calculation of mean, SD etc. is therefore not meaningful: this is the case for Figure 3J; 6I; 6K; 7I
=> for these panels, please switch to plotting individual data points instead of error bars

- The reference list still needs to be adjusted as specified in our Guide to Authors (https://link.springer.com/journal/44318/submission-guidelines#cms-Reference-guidelines). For each entry, we absolutely need: Journal name - Journal volume - Page number(s) or eLocator
We DO NOT need issue number (i.e., the number in brackets between volume number and page number), and we do not need DOIs or URLs (except for preprints, or the rare cases of online-ahead-of-print publications that do not have a formal citation yet). Please make sure to remove these parts from all entries.
Moreover, please carefully check all submissions once more for completeness, as some of them (e.g. Hidmi et al, Zhang & Hunter) still seem to lack full citation information. You may need to double-check in different places, e.g., PubMed, when entries are not unambiguous on certain sites.

I am therefore returning the manuscript to you once more, in order to allow you to make these final adjustments Please do not hesitate to contact me should you have any questions in this regard.

With kind regards,

Hartmut

Hartmut Vodermaier, PhD
Senior Editor, The EMBO Journal
h.vodermaier@embojournal.org

*** PLEASE NOTE: All revised manuscript are subject to initial checks for completeness and adherence to our formatting guidelines. Revisions may be returned to the authors and delayed in their editorial re-evaluation if they fail to comply to the following requirements. As a first step please read our guidelines for revised submissions: https://link.springer.com/journal/44318/submission-guidelines#cms-Revised-submissions

1) Every manuscript requires a Data Availability section (even if only stating that no deposited datasets are included). Primary datasets or computer code produced in the current study have to be deposited in appropriate public repositories prior to resubmission, and reviewer access details provided in case that public access is not yet allowed.

2) Each figure legend must specify
- size of the scale bars that are mandatory for all micrograph panels
- the statistical test used to generate error bars and P-values
- the type error bars (e.g., S.E.M., S.D.)
- the number (n) and nature (biological or technical replicate) of independent experiments underlying each data point
- Figures may not include error bars for experiments with n<3; scatter plots showing individual data points should be used instead.

3) Revised manuscript text (including main tables, and figure legends for main and EV figures) has to be submitted as editable text file (e.g., .docx format). We encourage highlighting of changes (e.g., via text color) for the referees' reference.

4) Each main and each Expanded View (EV) figure should be uploaded as individual production-quality files (preferably in .eps, .tif, .jpg formats). For suggestions on figure preparation/layout, please refer to our Figure Preparation Guidelines: https://media.springernature.com/original/springer-cms/rest/v1/content/27825798/data/v1

5) Point-by-point response letters should include the original referee comments in full together with your detailed responses to them (and to specific editor requests if applicable), and also be uploaded as editable (e.g., .docx) text files.

6) Please complete our Author Checklist, and make sure that information entered into the checklist is also reflected in the manuscript; the checklist will be available to readers as part of the Review Process File.

7) All authors listed as (co-)corresponding need to deposit, in their respective author profiles in our submission system, a unique ORCiD identifier linked to their name. Please see our Guide to Authors for detailed instructions.

8) Please note that supplementary information at EMBO Press has been superseded by the 'Expanded View' for inclusion of additional figures, tables, movies or datasets; with up to five EV Figures being typeset and directly accessible in the HTML version of the article.

9) To facilitate reproducibilty and cross-laboratory adoption of methodologies, please structure the Materials & Methods section as outlined in our guide to authors, including a completed Reagents and Tools Table.

10) Digital image enhancement is acceptable practice, as long as it accurately represents the original data and conforms to community standards. If a figure has been subjected to significant electronic manipulation, this must be clearly noted in the figure legend and/or the 'Materials and Methods' section. The editors reserve the right to request original versions of figures and the original images that were used to assemble the figure. Finally, we generally encourage uploading of numerical as well as gel/blot image source data.

At EMBO Press we ask authors to provide source data for the main manuscript figures. You will receive a separate email with instructions for providing source data with your revised manuscript, including how to upload and organize the files.

In the interest of ensuring the conceptual advance provided by the work, we recommend submitting a revision within 3 months (28th Jun 2026). Please discuss the revision progress ahead of this time with the editor if you require more time to complete the revisions. Use the link below to submit your revision:

Link Not Available

==

Point-by-point Response to Editorial comments:

1. For various figure panels, statistic representations have been applied to samples where N<3, and calculation of mean, SD etc. is therefore not meaningful: this is the case for Figure 3J; 6I; 6K; 7I
=> for these panels, please switch to plotting individual data points instead of error bars.
Response: We thank the editor for the clarification regarding data representation. In addition to Figures 3J, 6I, 6K and 7I, Figures 6B, 6D and 6F were also derived from two independent experiments. As suggested, individual data points have been plotted in Figures 3J, 6B, 6D, 6F, 6I, 6K and 7I. The authors would like to mention that in order to avoid complication of data representation, Figures 6B, 6D, 6F, 6I, 6K and 7I, which were originally line graphs, have now been converted to column graphs with individual data points. Further, statistical tests have been removed from Figures 6B, 6D, 6F, 6K and 7I, as suggested. The figure legends have also been appropriately modified.

2. The reference list still needs to be adjusted as specified in our Guide to Authors (https://link.springer.com/journal/44318/submission-guidelines#cms-Reference-guidelines). For each entry, we absolutely need: Journal name - Journal volume - Page number(s) or eLocator
We DO NOT need issue number (i.e., the number in brackets between volume number and page number), and we do not need DOIs or URLs (except for preprints, or the rare cases of online-ahead-of-print publications that do not have a formal citation yet). Please make sure to remove these parts from all entries.
Response: The references have now been formatted according to the syntax provided in **https://link.springer.com/journal/44318/submission-guidelines#cms-Reference-guidelines**.

3. Moreover, please carefully check all submissions once more for completeness, as some of them (e.g. Hidmi et al, Zhang & Hunter) still seem to lack full citation information. You may need to double-check in different places, e.g., PubMed, when entries are not unambiguous on certain sites.
Response: The mentioned references have been double-checked, and formatted according to the prescribed syntax.

EMBO Press Author Checklist

Corresponding Author Name: Birija Sankar Patro
Journal Submitted to: The EMBO Journal
Manuscript Number: EMBOJ-2025-122198

USEFUL LINKS FOR COMPLETING THIS FORM
The EMBO Journal - Author Guidelines
EMBO Reports - Author Guidelines
Molecular Systems Biology - Author Guidelines
EMBO Molecular Medicine - Author Guidelines

Reporting Checklist for Life Science Articles (updated January
This checklist is adapted from Materials Design Analysis Reporting (MDAR) Checklist for Authors. MDAR establishes a minimum set of requirements in transparent reporting in the life sciences (see Statement of Task: 10.31222/osf.io/9sm4x). Please follow the journal's guidelines in preparing your
Please note that a copy of this checklist will be published alongside your article.

Abridged guidelines for figures
1. Data
The data shown in figures should satisfy the following conditions:
- ➞ the data were obtained and processed according to the field's best practice and are presented to reflect the results of the experiments in an accurate and unbiased manner.
- ➞ ideally, figure panels should include only measurements that are directly comparable to each other and obtained with the same assay.
- ➞ plots include clearly labeled error bars for independent experiments and sample sizes. Unless justified, error bars should not be shown for technical
- ➞ if n<5, the individual data points from each experiment should be plotted. Any statistical test employed should be justified.
- ➞ Source Data should be included to report the data underlying figures according to the guidelines set out in the authorship guidelines on Data

2. Captions
Each figure caption should contain the following information, for each panel where they are relevant:
- ➞ a specification of the experimental system investigated (eg cell line, species name).
- ➞ the assay(s) and method(s) used to carry out the reported observations and measurements.
- ➞ an explicit mention of the biological and chemical entity(ies) that are being measured.
- ➞ an explicit mention of the biological and chemical entity(ies) that are altered/varied/perturbed in a controlled manner.
- ➞ the exact sample size (n) for each experimental group/condition, given as a number, not a range;
- ➞ a description of the sample collection allowing the reader to understand whether the samples represent technical or biological replicates (including how many animals, litters, cultures, etc.).
- ➞ a statement of how many times the experiment shown was independently replicated in the laboratory.
- ➞ definitions of statistical methods and measures:
  - common tests, such as t-test (please specify whether paired vs. unpaired), simple χ2 tests, Wilcoxon and Mann-Whitney tests, can be unambiguously identified by name only, but more complex techniques should be described in the methods section;
  - are tests one-sided or two-sided?
  - are there adjustments for multiple comparisons?
  - exact statistical test results, e.g., P values = x but not P values < x;
  - definition of 'center values' as median or average;
  - definition of error bars as s.d. or s.e.m.

Please complete ALL of the questions below.
Select "Not Applicable" only when the requested information is not relevant for your study.

Materials

Newly Created Materials	Information included in the manuscript?	In which section is the information available? (Reagents and Tools Table, Materials and Methods, Figures, Data Availability Section)
New materials and reagents need to be available; do any restrictions apply?	Yes	Reagents and Tools Table

Antibodies	Information included in the manuscript?	In which section is the information available? (Reagents and Tools Table, Materials and Methods, Figures, Data Availability Section)
For **antibodies** provide the following information:<br>- Commercial antibodies: RRID (if possible) or supplier name, catalogue number and or/clone number<br>- Non-commercial: RRID or citation	Yes	Reagents and Tools Table

DNA and RNA sequences	Information included in the manuscript?	In which section is the information available? (Reagents and Tools Table, Materials and Methods, Figures, Data Availability Section)
**Short novel DNA or RNA including primers, probes**: provide the sequences.	Yes	Reagents and Tools Table. Primer sequences are also provided in Appendix.

Cell materials	Information included in the manuscript?	In which section is the information available? (Reagents and Tools Table, Materials and Methods, Figures, Data Availability Section)
**Cell lines:** Provide species information, strain. Provide accession number in repository **OR** supplier name, catalog number, clone number, and/**OR** RRID.	Yes	Reagents and Tools Table
**Primary cultures:** Provide species, strain, sex of origin, genetic modification status.	Not Applicable	Not Applicable
Report if the cell lines were recently **authenticated** (e.g., by STR profiling) and tested for mycoplasma contamination.	Not Applicable	Not Applicable

Experimental animals	Information included in the manuscript?	In which section is the information available? (Reagents and Tools Table, Materials and Methods, Figures, Data Availability Section)
**Laboratory animals or Model organisms:** Provide species, strain, sex, age, genetic modification status. Provide accession number in repository **OR** supplier name, catalog number, clone number, **OR** RRID.	Not Applicable	Methods
**Animal observed in or captured from the field:** Provide species, sex, and age where possible.	Not Applicable	Not Applicable
Please detail **housing and husbandry conditions**.	Not Applicable	Not Applicable

Plants and microbes	Information included in the manuscript?	In which section is the information available? (Reagents and Tools Table, Materials and Methods, Figures, Data Availability Section)
**Plants:** provide species and strain, ecotype and cultivar where relevant, unique accession number if available, and source (including location for collected wild specimens).	Not Applicable	Not Applicable
**Microbes:** provide species and strain, unique accession number if available, and source.	Yes	Reagents and Tools Table

Human research participants	Information included in the manuscript?	In which section is the information available? (Reagents and Tools Table, Materials and Methods, Figures, Data Availability Section)
If collected and within the bounds of privacy constraints report on age, sex and gender or ethnicity for all study participants.	Not Applicable	Not Applicable

Core facilities	Information included in the manuscript?	In which section is the information available? (Reagents and Tools Table, Materials and Methods, Figures, Data Availability Section)
If your work benefited from core facilities, was their service mentioned in the acknowledgments section?	Not Applicable	Not Applicable

Design

Study protocol	Information included in the manuscript?	In which section is the information available? (Reagents and Tools Table, Materials and Methods, Figures, Data Availability Section)
If study protocol has been **pre-registered, provide DOI in the manuscript**. For clinical trials, provide the trial registration number **OR** cite DOI.	Not Applicable	Not Applicable
Report the **clinical trial registration number** (at ClinicalTrials.gov or equivalent), where applicable.	Not Applicable	Not Applicable

Laboratory protocol	Information included in the manuscript?	In which section is the information available? (Reagents and Tools Table, Materials and Methods, Figures, Data Availability Section)
Provide DOI OR other citation details if **external detailed step-by-step protocols** are available.	Not Applicable	Not Applicable

Experimental study design and statistics	Information included in the manuscript?	In which section is the information available? (Reagents and Tools Table, Materials and Methods, Figures, Data Availability Section)
Include a statement about **sample size** estimate even if no statistical methods were used.	Yes	Methods
Were any steps taken to minimize the effects of subjective bias when allocating animals/samples to treatment (e.g. **randomization procedure**)? If yes, have they been described?	Not Applicable	Not Applicable
Include a statement about **blinding** even if no blinding was done.	Not Applicable	Not Applicable
Describe **inclusion/exclusion criteria** if samples or animals were excluded from the analysis. Were the criteria pre-established?<br><br>If sample or data points were omitted from analysis, report if this was due to attrition or intentional exclusion and provide justification.	Not Applicable	Not Applicable
For every figure, are **statistical tests** justified as appropriate? Do the data meet the assumptions of the tests (e.g., normal distribution)? Describe any methods used to assess it. Is there an estimate of variation within each group of data? Is the variance similar between the groups that are being statistically compared?	Yes	Methods; Figure legends

Sample definition and in-laboratory replication	Information included in the manuscript?	In which section is the information available? (Reagents and Tools Table, Materials and Methods, Figures, Data Availability Section)
In the figure legends: state number of times the experiment was **replicated** in laboratory.	Yes	Figure Legends
In the figure legends: define whether data describe **technical or biological replicates**.	Yes	Figure Legends

Ethics

Ethics	Information included in the manuscript?	In which section is the information available? (Reagents and Tools Table, Materials and Methods, Figures, Data Availability Section)
Studies involving **human participants**: State details of **authority granting ethics approval** (IRB or equivalent committee(s), provide reference number for approval.	Not Applicable	Not Applicable
Studies involving **human participants**: Include a statement confirming that **informed consent** was obtained from all subjects and that the experiments conformed to the principles set out in the WMA Declaration of Helsinki and the Department of Health and Human Services Belmont Report.	Not Applicable	Not Applicable
Studies involving **human participants:** For publication of **patient photos**, include a statement confirming that consent to publish was obtained.	Not Applicable	Not Applicable
Studies involving experimental **animals**: State details of **authority granting ethics approva**l (IRB or equivalent committee(s), provide reference number for approval. Include a statement of compliance with ethical regulations.	Yes	Methods
Studies involving **specimen and field samples:** State if relevant **permits** obtained, provide details of authority approving study; if none were required, explain why.	Not Applicable	Not Applicable

Dual Use Research of Concern (DURC)	Information included in the manuscript?	In which section is the information available? (Reagents and Tools Table, Materials and Methods, Figures, Data Availability Section)
Could your study fall under dual use research restrictions? Please check biosecurity documents and list of **select agents and toxins** (CDC): https://www.selectagents.gov/sat/list.htm	Not Applicable	Not Applicable
If you used a select agent, is the security level of the lab appropriate and reported in the manuscript?	Not Applicable	Not Applicable
If a study is subject to dual use research of concern regulations, is the name of the **authority granting approval and reference number** for the regulatory approval provided in the manuscript?	Not Applicable	Not Applicable

Reporting

The MDAR framework recommends adoption of discipline-specific guidelines, established and endorsed through community initiatives. Journals have their own policy about requiring specific guidelines and recommendations to complement MDAR.

Adherence to community standards	Information included in the manuscript?	In which section is the information available? (Reagents and Tools Table, Materials and Methods, Figures, Data Availability Section)
State if relevant guidelines or checklists (e.g., **ICMJE, MIBBI, ARRIVE, PRISMA**) have been followed or provided.	Not Applicable	Not Applicable
For **tumor marker prognostic studies,** we recommend that you follow the **REMARK** reporting guidelines (see link list at top right). See author guidelines, under 'Reporting Guidelines'. Please confirm you have followed these guidelines.	Not Applicable	Not Applicable
For **phase II and III randomized controlled trials**, please refer to the **CONSORT** flow diagram (see link list at top right) and submit the CONSORT checklist (see link list at top right) with your submission. See author guidelines, under 'Reporting Guidelines'. Please confirm you have submitted this list.	Not Applicable	Not Applicable

Data Availability

Data availability	Information included in the manuscript?	In which section is the information available? (Reagents and Tools Table, Materials and Methods, Figures, Data Availability Section)
Have **primary datasets** been deposited according to the journal's guidelines (see 'Data Deposition' section) and the respective accession numbers provided in the Data Availability Section?	Yes	Data Availability Section
Were **human clinical and genomic datasets** deposited in a public access-controlled repository in accordance to ethical obligations to the patients and to the applicable consent agreement?	Not Applicable	Not Applicable
Are **computational models** that are central and integral to a study available without restrictions in a machine-readable form? Were the relevant accession numbers or links provided?	Not Applicable	Not Applicable
If publicly available data were reused, provide the respective **data citations in the reference list**.	Not Applicable	Not Applicable
